# Neighbor Preferences of Amino Acids and Context-Dependent Effects of Amino Acid Substitutions in Human, Mouse, and Dog

**DOI:** 10.3390/ijms150915963

**Published:** 2014-09-10

**Authors:** Mingchuan Fu, Zhuoran Huang, Yuanhui Mao, Shiheng Tao

**Affiliations:** 1 College of Life Sciences and State Key Laboratory of Crop Stress Biology in Arid Areas, Northwest A&F University, Yangling 712100, China; E-Mails: fmcsky@nwsuaf.edu.cn (M.F.); sdrzh@nwsuaf.edu.cn (Z.H.); maoyuanhui@nwsuaf.edu.cn (Y.M.); 2 Bioinformatics Center, Northwest A&F University, Yangling 712100, China

**Keywords:** protein, neighborhood, substitution, context-dependence, secondary structure

## Abstract

Amino acids show apparent propensities toward their neighbors. In addition to preferences of amino acids for their neighborhood context, amino acid substitutions are also considered to be context-dependent. However, context-dependence patterns of amino acid substitutions still remain poorly understood. Using relative entropy, we investigated the neighbor preferences of 20 amino acids and the context-dependent effects of amino acid substitutions with protein sequences in human, mouse, and dog. For 20 amino acids, the highest relative entropy was mostly observed at the nearest adjacent site of either *N*- or *C*-terminus except C and G. C showed the highest relative entropy at the third flanking site and periodic pattern was detected at G flanking sites. Furthermore, neighbor preference patterns of amino acids varied greatly in different secondary structures. We then comprehensively investigated the context-dependent effects of amino acid substitutions. Our results showed that nearly half of 380 substitution types were evidently context dependent, and the context-dependent patterns relied on protein secondary structures. Among 20 amino acids, P elicited the greatest effect on amino acid substitutions. The underlying mechanisms of context-dependent effects of amino acid substitutions were possibly mutation bias at a DNA level and natural selection. Our findings may improve secondary structure prediction algorithms and protein design; moreover, this study provided useful information to develop empirical models of protein evolution that consider dependence between residues.

## 1. Introduction

Amino acid sequences are necessary to allow proteins to fold into their native conformations [1]. As such, protein sequence patterns should be characterized to understand protein structure, function, and stability. Previous studies revealed that amino acid compositions vary in secondary structures [2,3,4,5]. For example, M, A, K, E, and L are helix-preferred amino acids, whereas P and G likely disrupt helices. Likewise, V, I, T, F, W, and Y show high propensities for β-strand, whereas P, G, A, and E are poor β-strand-forming residues. In addition to amino acid preferences for different secondary structures, preferences for particular residue pairs in protein sequences have also been discovered. These preferred residue pairings are found in α-helices [6,7,8,9,10,11], parallel/antiparallel β-sheets [12,13,14], loops [15], and protein inter-domain linkers [16]. Such residue pairs are related to secondary structure formation and protein stabilization. In this work, the neighbor preferences of 20 amino acids were investigated and the neighbor preference patterns among different secondary structures were compared. This research may provide new insights into neighbor preferences of amino acids; furthermore, this study may improve secondary structure prediction algorithms and protein design.

Inspired by the research on neighbor preferences of amino acids, we further want to investigate whether or not amino acid substitutions also prefer neighborhood context and the specific context-dependence pattern of each substitution type. Thus, the second issue addressed in this study is to assess the context-dependent effects of amino acid substitutions. Nucleotide mutations are context dependent, and the most important mutation bias is the CpG effect [17,18,19,20]. Based on these empirical studies, several context-dependent evolutionary models for mammals have been established [21,22,23,24]. However, similar studies on amino acid substitutions are still very few. Understanding amino acid substitution patterns and constructing explicit protein evolution models are critical to phylogenetic analyses. Common protein evolution models, whether theoretical [25,26] or empirical [27,28,29,30], are usually constructed on the basis of the assumption that all protein sites evolve at the same rate and independent of other sites (*i.e.*, amino acid substitutions occurring at one site are independent from amino acids at other sites). Several models have been proposed to relax the assumption of equal evolutionary rates at all sites [31,32,33,34,35,36]; by contrast, the assumption of site-independence has been maintained. It is now widely agreed that the site-independence assumption is simplistic and biologically unrealistic. To relax such assumptions, researchers have developed some more elaborate models allowing for dependence between residues in recent years. Such models can be classified as knowledge-based (informational) models [37,38,39] and physics-based models [40,41]. However, these mechanistic models inaccurately describe protein thermodynamics and appear outperformed by some sophisticated site-independent models [42]. An alternative approach that may overcome the shortcomings of these mechanistic models is to use phenomenological models, which attempt to fit the results of sequence change without understanding the underlying biological process [43]. Wang *et al.* [44] first assessed the context-dependent effects of amino acid substitutions by using a dataset of 45,173 orthologous proteins. They found that amino acid substitutions are neighbor dependent, and the patterns of neighbor-dependence are similar between *N*- and *C*-termini. However, the specific context-dependence pattern of each amino acid substitution type has not been assessed. Moreover, context-dependence patterns in different protein secondary structures have not been considered in their research. In this work, the context-dependent effect of each amino acid substitution type and the context-dependence patterns in different secondary structures were comprehensively investigated. Our findings provided very useful information for further development of protein evolution empirical models that consider site dependence.

Relative entropy [45], also called Kullback-Leibler divergence or information gain, provides a measurement of the distance between two probability distributions *P* and *Q*. In general, *P* represents the observed probability distribution of a dataset and *Q* represents the expected or theoretical probability distribution. Applying relative entropy, we aimed to investigate the neighbor preference patterns of 20 amino acids and the context-dependent effects of amino acid substitutions.

## 2. Results

### 2.1. Neighbor Preferences of Amino Acids

**Figure 1:**
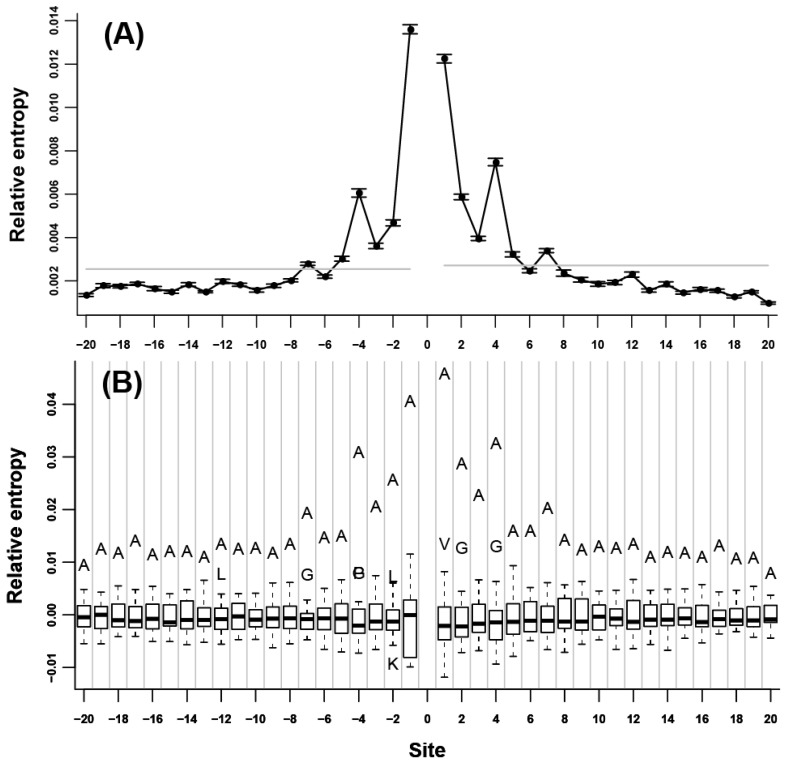
Neighbor preference pattern of the amino acid A. (**A**) Relative entropies of the neighboring sites of A of *N*- and *C*-termini. The vertical coordinate represents the value of relative entropy, and the horizontal coordinate represents the relative distance to the 0 site. The flanking sites of *N*-terminus are indicated by negative numbers and the flanking sites of *C*-terminus are indicated by positive numbers. The gray lines are the thresholds representing 0.001 significance level. The standard deviation of the relative entropy in each site is indicated by black error bars; (**B**) Boxplot of the 20 relative entropies calculated by 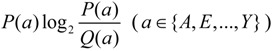 at each flanking site of the amino acid A. The bold line in each box represents the median of the 20 values. The top and bottom lines of each box indicate the upper and lower quartiles, respectively. The upper and lower whiskers represent the largest data point which was less than the sum of the upper quartile plus 1.5 times the interquartile range (IQR), and the lowest data point which was greater than the lower quartile minus 1.5 IQR, respectively. In order to determine which amino acids were apparently preferred (or not preferred) in the neighboring sites, the outliers were represented by the corresponding one letter codes of amino acids.

**Figure 2:**
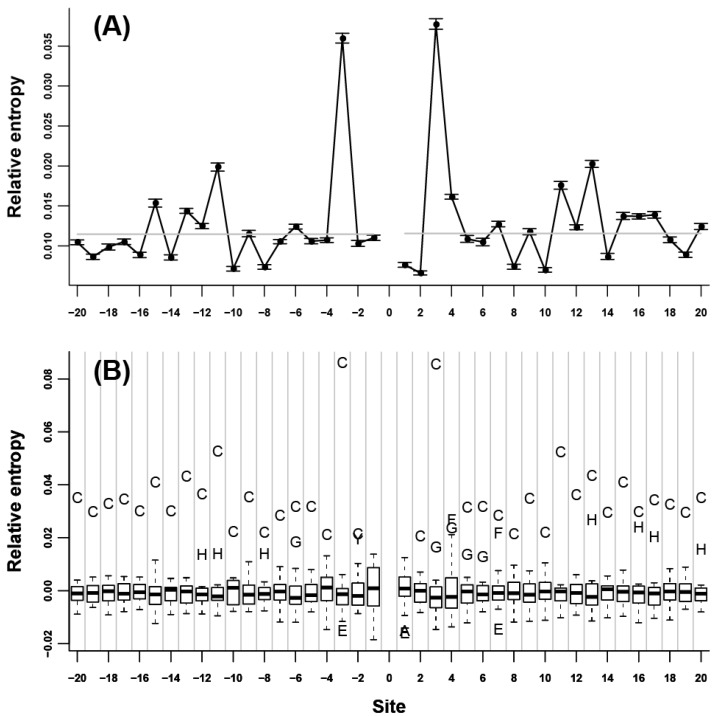
Neighbor preference pattern of the amino acid C. (**A**) Relative entropies of the neighboring sites of C of *N*- and *C*-termini; (**B**) Boxplot of the 20 relative entropies at each flanking site of the amino acid C.

**Figure 3:**
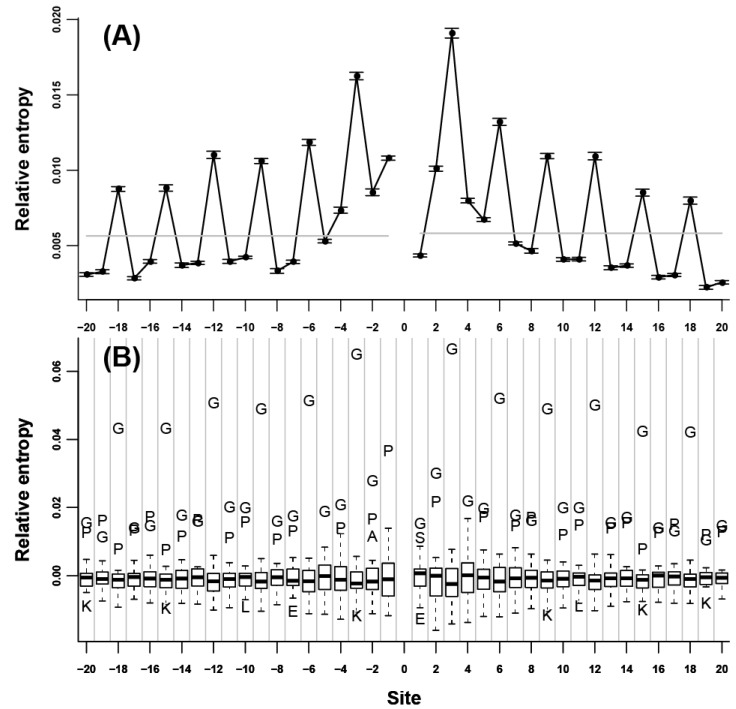
Neighbor preference pattern of the amino acid G. (**A**) Relative entropies of the neighboring sites of G of *N*- and *C*-termini; (**B**) Boxplot of the 20 relative entropies at each flanking site of the amino acid G.

We initially assessed the neighbor preference pattern of each amino acid type. Our results showed that all of the 20 amino acids were remarkably neighbor-preferred (Figure S1). The highest relative entropy was mainly observed at the nearest adjacent site of either *N*- or *C*-terminus, indicating that neighbor preferences were the strongest for the two immediate flanking sites; this value subsequently decreased when the distance to the 0 site increased. Relative entropy decreased rapidly in the nearest 5 to 7 flanking sites; afterward, relative entropy decreased very slowly and became not significant (Figure 1A). C and G are two exceptions. For C (Figure 2A), the highest relative entropies appeared at the third flanking sites of both *N*- and *C*-termini. Without these sites, relative entropies were relatively low. For G (Figure 3A), the relative entropies of *N*- and *C*-terminal flanking sites showed an evident periodic change, that is, relative entropies were remarkably high in all of the 3*n* (*n* = ±1, ±2, ±3…) flanking sites; other sites showed low values. The peaks at the 3*n* flanking sites decreased gradually when the distance to the 0 site increased. To determine the specific amino acids responsible for the high relative entropy at one flanking site, we further investigated the 20 relative entropies calculated using 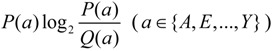 at each flanking site of the 20 amino acids (Figure 1B, Figure 2B, and Figure 3B). We found that for more than half of the amino acids (A, L, V, P, G, S, T, Q, C, H, K, R, D, and E), the corresponding residues tended to show high propensity at the neighboring sites (e.g., in Figure 1B, amino acid A was a outlier which was above the upper whisker of the boxplot in each flanking site, which demonstrated that amino acid A was a type of preferred amino acid in the neighboring sites of amino acid A).

### 2.2. Neighbor Preferences of Amino Acids in Different Protein Secondary Structures

**Figure 4:**
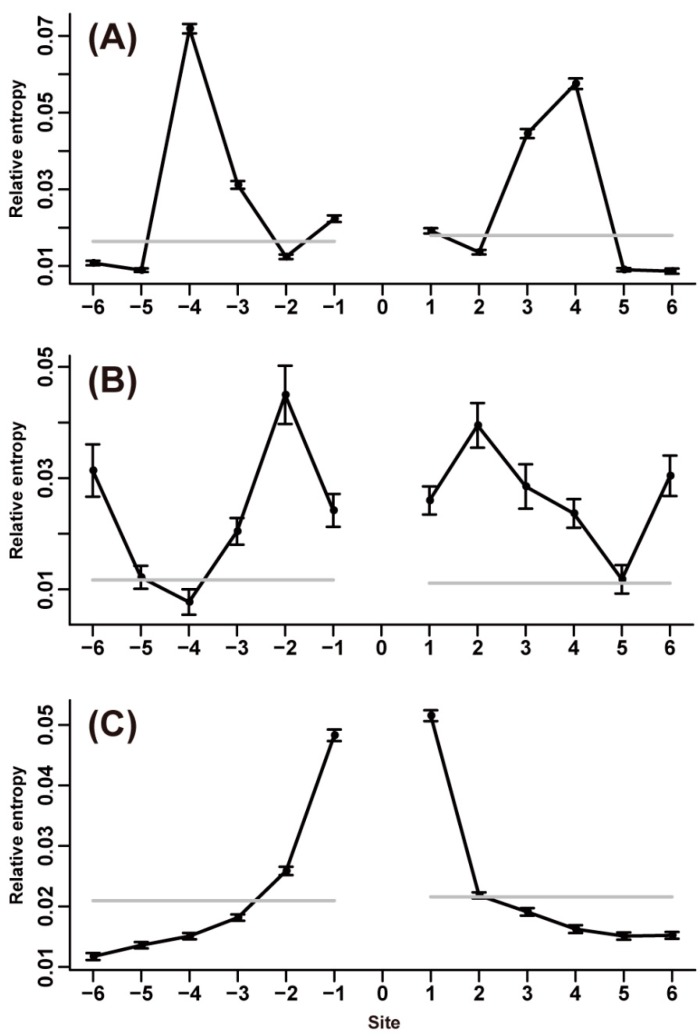
Neighbor preference patterns of V, T, and E in different secondary structures. (**A**) Neighbor preference pattern of the amino acid V in α-helix; (**B**) Neighbor preference pattern of the amino acid T in β-strand; and (**C**) Neighbor preference pattern of the amino acid E in coil. (Boxplots of these amino acids can be found in Figures S2–S4).

Considering the limitation of the average sequence length of each secondary structure (α-helix, β-strand and coil), we extracted six instead of twenty flanking sites for each type of amino acid. Neighbor preference patterns in the three secondary structures were different. In α-helix, the highest relative entropy was commonly seen at the third or fourth flanking site for 14 of the 20 amino acids (F, A, V, L, I, M, W, C, Y, Q, R, K, E, and D; Figure 4A; Figure S2). The majority of these amino acids favorably interacted with residues at spacing *i*, *i* + 3 or *i*, *i* + 4 of the same kind or similar polarity, that is, nonpolar/nonpolar or polar/polar pairings were predominant for the *i*, *i* + 3 and *i*, *i* + 4 combinations. The exceptions were C and Y, which showed high propensity for the residues of opposite polarity. L was frequently found at the third or fourth neighboring sites of nonpolar amino acids. Charged amino acids preferred to interact with residues at spacing *i*, *i* + 3 or *i*, *i* + 4 of opposing charge (e.g., R-E, K-E, and K-D pairings). Some exceptional amino acid pairings, such as L-V, L-I, L-L, F-L, A-A, Q-Q, and E-R, at spacing *i*, *i* + 4 observed in our study have also been documented in other studies [7,11]. For the six other amino acids (P, G, T, N, S, and H), the highest relative entropy was mainly observed at the second flanking site of *N*- or *C*-terminus (except P). In contrast to *i*, *i* + 3 and *i*, *i* + 4 pairings, the exceptional *i*, *i* + 2 amino acid combinations were all polar/nonpolar pairings except G. L was the most preferred residue at the ±2 sites of these amino acids.

Studies have investigated residue pairing preferences on adjacent β-strands [12,13,14]. In contrast to these studies, our study investigated the neighbor preferences of amino acids along the same strand. Although intimate interactions between residues in one strand, which is almost fully extended, are rare because of a large C_α_-C_α_ distance [13], evident neighbor preferences were still found. For the amino acids in β-strand, the highest relative entropy commonly appeared at the immediate or second adjacent site (Figure 4B; Figure S3). The preferred *i*, *i* + 1 pairings were mainly polar/nonpolar combinations (e.g., V-S, I-Q, N-I, F-H, F-S, D-I, and V-E); by contrast, polar/polar or nonpolar/nonpolar combinations were predominant for *i*, *i* + 2 pairings (e.g., G-I, V-G, N-T, and N-S). V and I were frequently found at neighboring sites of other amino acids. Although G is a poor β-strand-forming residue, G showed moderately high propensity at neighboring sites of other amino acids, such as L, V, I, and F.

In coil, all of the amino acids showed evident neighbor preferences; the highest relative entropy was mainly observed at the nearest flanking site (Figure 4C; Figure S4). For C and G, the highest relative entropies were detected at the third flanking sites of both *N*- and *C*-termini. In addition, both residues showed apparently high relative entropies at ±6 sites. More than half of the amino acids in coil (A, P, G, S, Q, C, Y, H, K, R, D, and E) showed high propensity for the same type of residues. P occurred frequently at neighboring sites of almost all of the amino acids.

### 2.3. Context-Dependent Effects of Amino Acid Substitutions

**Figure 5:**
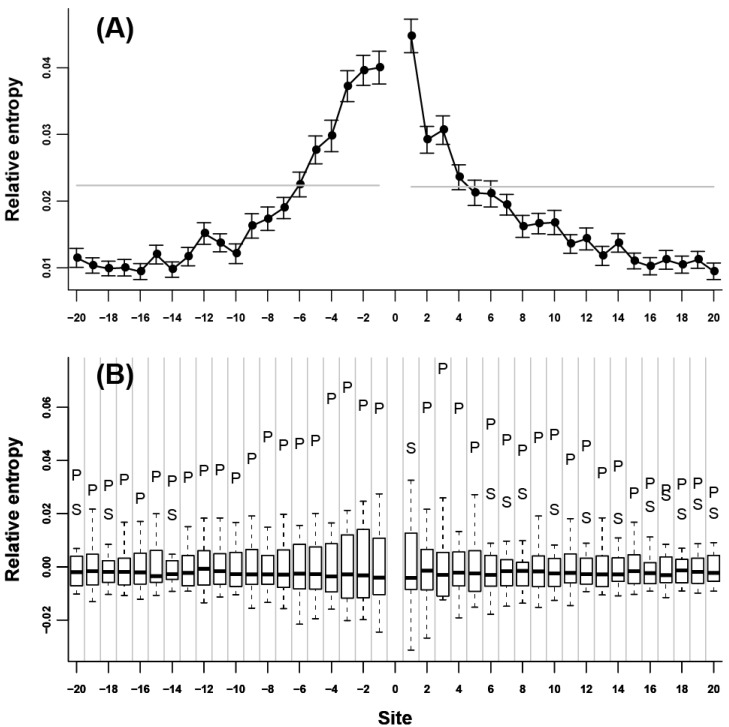
Context-dependence pattern of *S*→*P* substitution. (**A**) Relative entropies of the neighboring sites of the *S*→*P* substitution of *N*- and *C*-termini; (**B**) Boxplot of the 20 relative entropies at each flanking site of the *S*→*P* substitution.

To investigate whether or not amino acid substitutions are dependent on neighborhood context, we then assessed the pattern of context-dependent effects of amino acid substitutions. Our results showed that nearly half of the 380 amino acid substitution types were remarkably context dependent, and the highest relative entropies were mainly observed at the two nearest flanking sites (Figure 5A; Figure S5). Among the 20 amino acids, P, E, S, A, and G were frequently found at neighboring sites of amino acid substitutions, particularly substitutions between nonpolar residues or between nonpolar and polar residues. For substitutions between two polar amino acids, P, E, S, K, and Q appeared to be the preferred neighbors. A common characteristic of the amino acid substitutions was that when they substituted to P, E, S, A, G or K, the neighboring sites tended to show high propensity for the amino acid type as the post-substituted one (Figure 5B). Considering the self-preference of these amino acids (P, E, S, A, G and K), we found that a residue with a specific neighborhood context in proteins was possibly substituted in another residue, which exhibited a high propensity of this neighborhood context. Among 20 amino acids, P was the most preferred residue at neighboring sites of many substitution types. For some substitution types, this high propensity of P could extend to nearly all of the flanking sites (Figure 5B). Although P was not the most heterogeneous amino acid at neighboring sites in some other cases, P showed relatively high frequency in association with other amino acids.

### 2.4. Context-Dependent Effects of Amino Acid Substitutions in Different Protein Secondary Structures

Some amino acid substitution types in each secondary structure were excluded from this research because of limited data size. In β-strand, only two substitution types were considered and neither of them showed an evident context-dependent effect.

**Figure 6:**
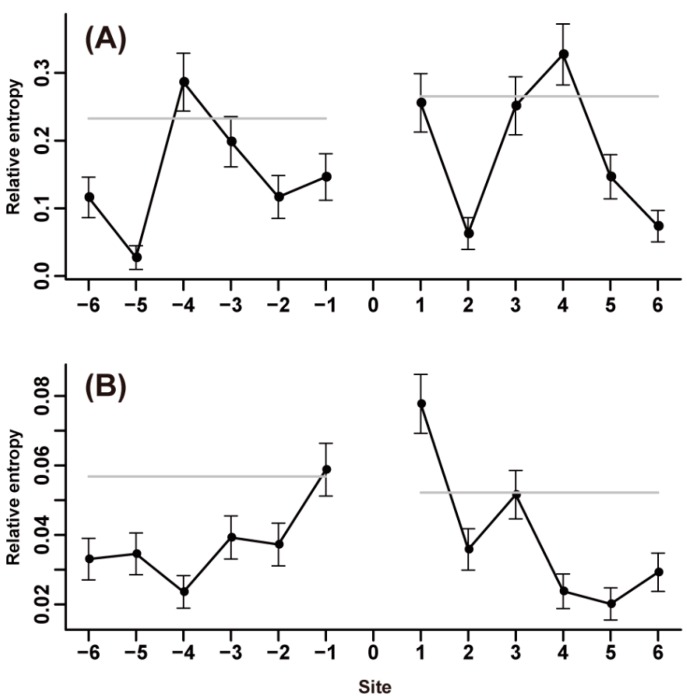
Context-dependence pattern of *G*→*E* substitution in α-helix and coil. (**A**) Context-dependence pattern of the *G*→*E* substitution in α-helix; (**B**) Context-dependence pattern of the *G*→*E* substitution in coil. (Boxplots of this amino acid substitution type in α-helix and coil can be found in Figures S6 and S7).

In α-helix, 43 out of the 96 considered substitution types showed evident context-dependent effects (Figure S6). Similar to the neighbor preference patterns of amino acids in α-helix, the highest relative entropy of most amino acid substitution types was observed at the third or fourth flanking site (Figure 6A). Notably, the ±3 or ±4 sites of these amino acid substitutions always showed high propensity for one or more residues of E, Q, K, and R. Eight substitution types showed the highest relative entropy at the second flanking site, and L was the most preferred neighbor at the ±2 sites in most cases. Interestingly, all of these eight substitution types (except *L*→*V*) were the ones substituted to G, T, N, or S (e.g., *A*→*G*, *I*→*T*, *K*→*N*, and *A*→*S*) showing the highest neighbor preference for the ±2 sites in α-helix. In addition, some substitution types showed the greatest heterogeneity at the nearest flanking sites (particularly +1 site); most of these substitutions involved L, I, or V (e.g., *I*→*M*, *L*→*F*, and *T*→*V*).

In coil, 163 substitution types were considered. Among these substitutions, 40 showed evident context-dependent effects (Figure S7). In these substitution types, the highest relative entropy mostly appeared at the nearest flanking sites (Figure 6B). Among the 20 amino acids, P occurred most frequently at the neighboring sites of amino acid substitutions. Furthermore, A, S, E, and G showed high propensities at neighboring sites. Substitutions to A or E showed similar propensities of neighborhood context as the corresponding post-substituted residue.

## 3. Discussion

In this research, more than half of the 20 amino acids showed propensity toward the amino acid types as themselves at the neighboring sites. This self-preference is consistent with that in previous studies and is likely caused by replication slippage and nucleotide mutation bias [46,47,48]. Self-clustering of amino acids contributes to the emergence of novel proteins and protein-protein interaction networks [49,50]. I, M, F, W, N, and Y do not show evident self-preference pattern, suggesting that tandem repeats of these residues are not favored in proteins [51].

The exceptional neighbor preference patterns of C and G are structurally and functionally important. Miseta and Csutora [52] initially revealed that two Cs are frequently found with the separation of two other residues in various proteins of several species. They suggested that this result is attributed to the first and fourth residues in α-helices or β-turns that are closest to each other. Contrary to previous studies, this exceptional pattern of C was only observed in coils in our study. The periodic occurrence of G was observed in this study. Periodic patterns play a role in structural packing and atom interactions [51]. Previous studies showed that G at every third position is essential for the formation of a collagen triple helix [53]. Using Pfam [54], we further grouped the proteins according to the presence or absence of the collagen triple-helix domain. The result showed that a remarkable periodic occurrence of G occurred in the collagen proteins; by contrast, no such pattern was observed in the other proteins (Figure S8). Hence, the periodic occurrence of G observed in this study resulted from the influence of collagen proteins.

Cellular location greatly influences protein structure and function. The same type of amino acid may behave distinctively in different protein environments. To assess whether or not exceptional neighbor preference patterns of C and G are identical in different protein environments, we further investigated the neighbor preferences of these two amino acids in different cellular locations (results of C are shown in Figure S9, results of G are shown in Figure S10). In nuclear proteins, the relative entropies were strikingly high at the ±3 sites of C. In cytosol proteins, the ±3 sites of C also showed relatively high relative entropies. For G in proteins located in the cytosol, endoplasmic reticulum, extracellular environment, lysosome, and mitochondria, the pattern of periodic change was observed. No such pattern was observed in other protein environments. We also repeated this work by investigating the protein sequences with corresponding GO [55] entries (about one-third of the dataset) which were retrieved by DAVID [56,57]. The results showed that the neighbor preference patterns of C and G in different cellular locations were similar between these two datasets (Figure S11). These findings revealed that neighbor preference patterns for both amino acids greatly differed in various protein environments.

The neighbor preference patterns of amino acids in different secondary structures are necessary to maintain the corresponding structural conformation. Distinct neighbor preference patterns of amino acids were found in α-helix, β-strand, and coil. In α-helix, the highest relative entropy occurred at the third or fourth flanking sites in the majority of the amino acids. This result is mainly attributed to the residue pairs at spacing *i*, *i* + 3 or *i*, *i* + 4 appearing on the same side of α-helix; this spatial proximity induces the two side chains of these pairs to favorably interact and stabilize helices by salt bridges, hydrogen bonds, or hydrophobic interactions of particular amino acid combinations [7,11]. In particular, H-bonding between residues at spacing *i*, *i* + 4 contributes greatly to α-helix stabilization. The high propensity for nonpolar/nonpolar or polar/polar combinations of *i*, *i* + 3 and *i*, *i* + 4 residue pairings is mainly attributed to the amphiphilic nature of α-helix, which contains one hydrophobic side and one hydrophilic side. The exceptional *i*, *i* + 4 polar/nonpolar pairings observed in this study indicate that the interactions between some residues of opposite polarity may be necessary to stabilize a α-helix structure [8]. The preferred *i*, *i* + 2 pairings were predominant polar/nonpolar combinations mainly because the residues at spacing *i*, *i* + 2 are on the opposite side of α-helix.

One characteristic of the neighbor preference pattern in β-strand is the high propensity for polar/nonpolar combinations of *i*, *i* + 1 pairings and polar/polar or nonpolar/nonpolar combinations of residues at spacing *i*, *i* + 2. This observation is reasonable because the alternating pattern of nonpolar and polar residues is a general characteristic of β-strands and is necessary to determine β-strand structure [58,59].

In coil, P is frequently found at neighboring sites of almost all of the amino acids. P is the sole imino acid among 20 amino acids. The amino nitrogen of P is bonded to two alkyl groups rather than one alkyl group; therefore, no amide hydrogen can be donated to form H-bonding. This unique characteristic allows P to break α-helix and β-strand conformations and lead to form irregular secondary structures [15]. Consequently, P was the preferred neighbor of residues in coils.

In this work, the context-dependent effects of amino acid substitutions were comprehensively investigated. The underlying mechanisms of the context-dependent effects remain unclear. Two possible reasons are nucleotide mutation bias and natural selection. Misawa and Kikuno [60] found that approximately 14% of synonymous and nonsynonymous substitutions in human genes are caused by CpG hypermutations [61]. Considering that a nonsynonymous substitution is involved in the CpG effect (e.g., a nonsynonymous substitution from Val to Ile, *i.e.*, *GTT*→*ATT*, *GTC*→*ATC* or *GTA*→*ATA*, provided that the third position of the 5'-adjacent codon is C), it may be retained rather than purified if such substitution does not apparently change protein structure and function. Thus, some amino acid substitutions possibly occur in specific neighborhood contexts because of mutation bias. Another reason is natural selection. One notable finding in this work is that amino acid substitutions likely showed similar propensities of neighborhood context to those of post-substituted residue, particularly in the substitutions to P, E, S, A, G, and K in all of the proteins, to G, T, N, and S in α-helices, and to A and E in coils. This result indicated that one amino acid with a specific neighborhood context in proteins was possibly substituted by the amino acid with the propensity of such neighborhood context. This characteristic of amino acid substitutions is reasonable because natural selection favors the maintenance of protein local structures and functions.

## 4. Experimental Section

### 4.1. Neighbor Preferences of Amino Acids

We investigated the neighbor preference pattern of each amino acid type by using the protein sequences of human (*Homo sapiens*), mouse (*Mus musculus*), and dog (*Canis lupus familiaris*) downloaded at Ensembl database [62,63]. A total of 20 flanking sites of *N*- and *C*-termini of each amino acid were extracted. The sites containing less than 20 flanking sites of either *N*- or *C*-terminus were excluded from the research. We extracted six instead of twenty flanking sites when neighbor preference patterns were investigated in different secondary structures because of the limitation of the average sequence length of protein secondary structures.

### 4.2. Context-Dependent Effects of Amino Acid Substitutions

The orthologous information of mammals was downloaded from OrthoDB database [64,65], which provides the hierarchical catalog of orthologs, including 252 eukaryotic species and 1115 bacteria. The OrthoDB database can provide the corresponding Ensembl Protein IDs of each species in each orthologous group. We extracted the orthologous groups of human, mouse, and dog (a total of 11,007 orthologous groups), and downloaded the corresponding protein sequences from the Ensembl database.

We aligned the orthologous sequences of human, mouse, and dog by using clustalw [66] with default parameters. Using the PHYLIP [67] format files produced by clustalw as input files, the codeml program in the PAML (Phylogenetic Analysis by Maximum Likelihood) package [68] was then used to reconstruct the ancestral sequences. The control file for the codeml program was codeml.ctl. The empirical model of jones.dat (the parameter aaRatefile in codeml.ctl) was used in this study. The parameters “seqfile” and “outfile” were changed for each orthologous (alignment) group by a simple Perl script. A phylogenetic tree being constructed by the taxonomy tools of NCBI [69] was used (Figure S12). The average accuracies of the two ancestral sequences were >96% and 98% (PAML calculates the posterior probability of each site in ancestral node by using maximum likelihood method [70], which can be used as the measurement of the accuracy of the site. The overall accuracies of the ancestral sequences for each orthologous group could be found in the rst output file, which was produced by the codeml program).

There were four branches along the phylogenetic tree in total (Figure S12). In each branch, there were two nodes, which represented the descendant node and the corresponding ancestral node. To infer the amino acid substitutions along one branch, pair-wise comparisons between the sequences of the two nodes were conducted (the rst files produced by the codeml program could give us the summary of amino acid substitutions along each branch). At last, above 1,000,000 substitutions were inferred along the tree. To assess the context-dependent effects of amino acid substitutions, we extracted 20 flanking sites of *N*- and *C*-termini from each substitution. We excluded substitution sites with <20 flanking sites. Six flanking sites were extracted to investigate context dependence patterns of amino acid substitutions in different secondary structures.

### 4.3. Calculation of Relative Entropy

The relative entropy [45] of an amino acid *a* at one particular flanking site of one type of amino acids or substitutions was calculated as follows:

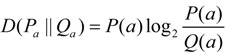
(1)
where P(a) represents the observed frequency of amino acid *a* at a given flanking site and Q(a) represents the background or expected frequency of amino acid *a* at the site.

We define the relative entropy of one given flanking site as the sum of the relative entropies of the 20 amino acids at the site, as expressed in the following equation:

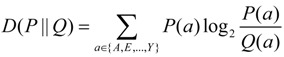
(2)


This summation is always nonnegative and is equal to zero if and only if for all of the 20 amino acids at one flanking site.

To investigate the neighbor preferences of amino acids, we assigned the background distribution of amino acids as the observed frequencies of the 20 amino acids in the corresponding dataset (all of the protein sequences, sequences in one secondary structure, or in one cellular location). To analyze the context-dependent effects of amino acid substitutions and prevent the neighbor preferences of amino acids from providing biased results, we calculated the background distribution of amino acids as the distribution of the 20 amino acids at each flanking site of one particular amino acid type in the corresponding dataset. For example, the background distribution of amino acids at each flanking site in *A*→*D* substitution corresponded to the amino acid distribution at the corresponding flanking site of A.

To estimate thresholds, we randomly shuffled the 20 (or 6) sites of all *N*- or *C*-terminal flanking sequences of one type of amino acids or substitutions and then recalculated the relative entropy of each flanking site [71]. This process was repeated 500 times, and 10,000 (20 × 500) simulated relative entropies were obtained. We chose the tenth-highest value as threshold representing the 0.001 significance level. For investigations on different protein secondary structures, we selected the third-highest value among 3000 (6 × 500) simulated relative entropies as threshold. The thresholds of the two sides commonly showed differences because the data sizes of *N*- and *C*-terminal flanking sequences were usually distinct. Through bootstrap samplings of the dataset for 100 times, the standard deviations of relative entropies were calculated.

### 4.4. Prediction of Protein Secondary Structures

We predicted the secondary structures of protein sequences by using the Jpred server [72]. Jpred is a protein secondary structure prediction server incorporating the Jnet algorithm [73]. The Jnet method was developed by seven-fold cross-validated training on the dataset derived from SCOP [74] database at the superfamily level. The server can update in sync with the major updates of SCOP and UniProt [75], which makes it maintain high-accuracy predictions. Now, the average accuracy of this server is >81%. This server predicts three categories of secondary structures (α-helix, β-strand, and coil) of a protein sequence. In this study, we used Advanced Jpred [76] for batch submission of the protein sequences. The input files were in FASTA format (a format begins with a single description line, then followed by sequence lines), with each sequence being given a unique name.

### 4.5. Prediction of Protein Cellular Locations

We predicted the cellular locations of our dataset by using WoLF PSORT [77,78]. The server can convert amino acid sequences into numerical localization features based on known sorting signal motifs and several other sequence features. A wrapper method is used to select the most relevant features. The dataset of WoLF PSORT is comprised of fungi, plant and animal. The information of cellular location used in the server is obtained from UniProt [75] and GO [55] databases. At present, the average prediction accuracy of the server is >80%. This server can be used to predict approximately 11 cellular locations (e.g., cytosol, extracellular, nuclear, and plasma membrane) in animal sequences.

## 5. Conclusions

In this work, amino acids were evidently neighbor preferred and the amino acid substitutions were apparently context dependent. These findings could be exploited in the improvement of secondary structure prediction algorithms and further development of protein evolution models. Further studies should be conducted to investigate these neighbor preference patterns in more species and proteins with different functions. Further studies could also be performed to construct a context-dependent model of protein evolution incorporating the results of this work.

## Supplementary Materials

Supplementary materials can be found at http://www.mdpi.com/1422-0067/15/9/15963/s1.

## Supplementary Files

Supplementary File 1
