# Supplementary material for: Neighbor Preferences of Amino Acids and Context-Dependent Effects of Amino Acid Substitutions in Human, Mouse, and Dog
Source: Int J Mol Sci. 2014 Sep 10;15(9):15963–80. doi: 10.3390/ijms150915963 (PMC4200849; doi:10.3390/ijms150915963)

Supplementary Information

Figure S1. Neighbor preference patterns of the 20 amino acids.

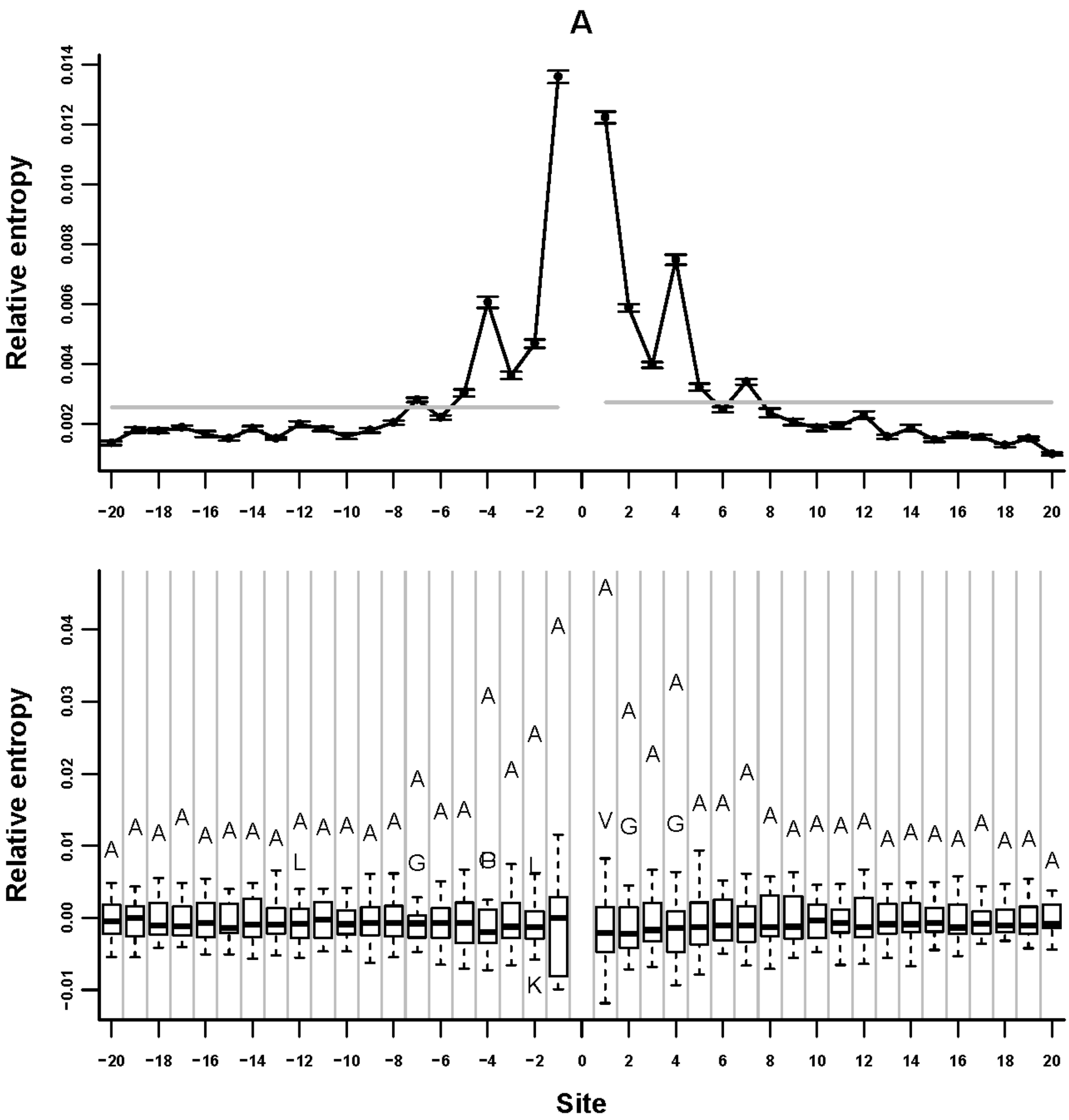

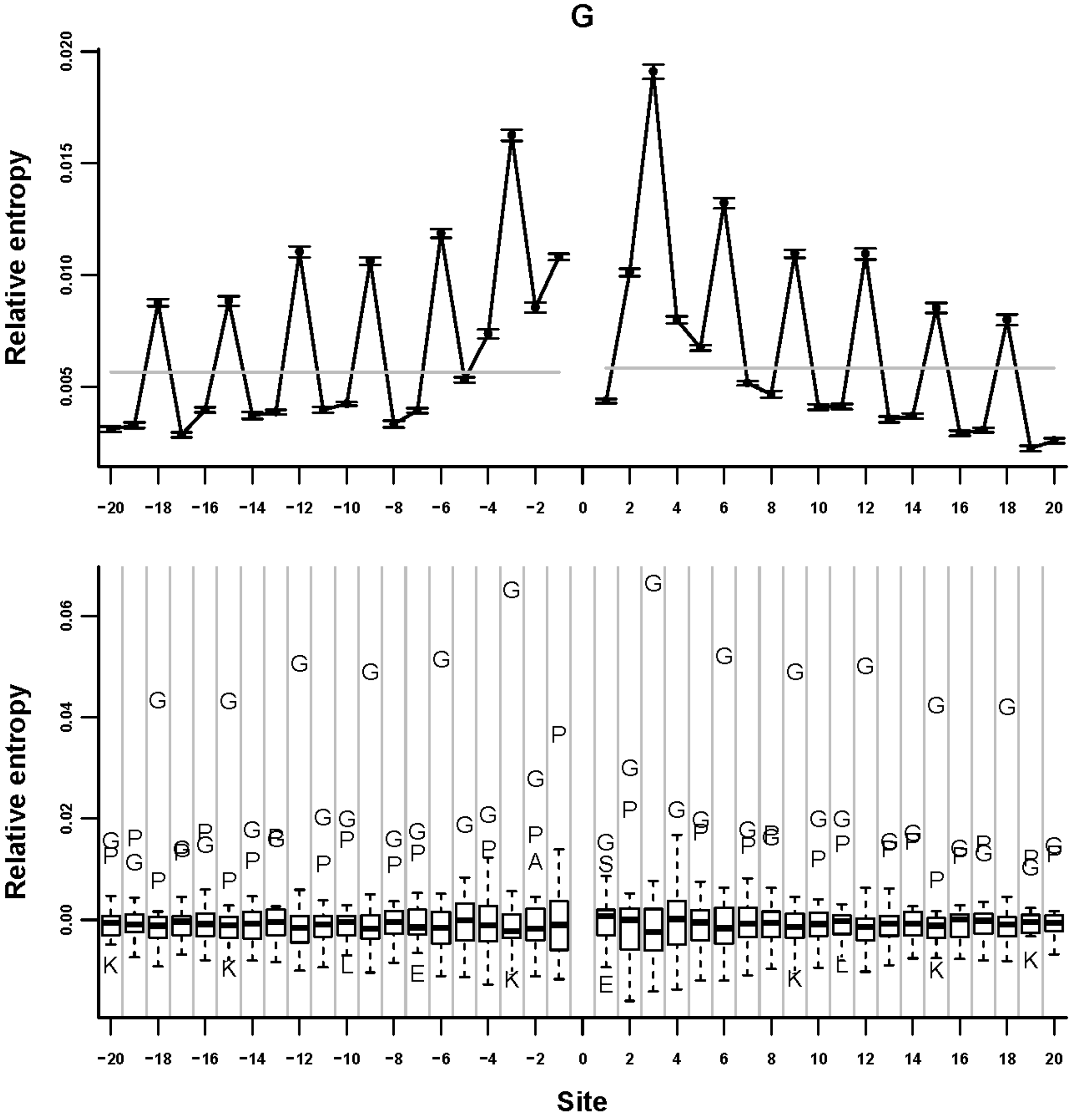
G
Relative entropy
0.005
0.010
0.015
0.020
−20 −18 −16 −14 −12 −10 −8 −6 −4 −2 0 2 4 6 8 10 12 14 16 18 20
Relative entropy
0.00
0.02
0.04
0.06
−20 −18 −16 −14 −12 −10 −8 −6 −4 −2 0 2 4 6 8 10 12 14 16 18 20
Site

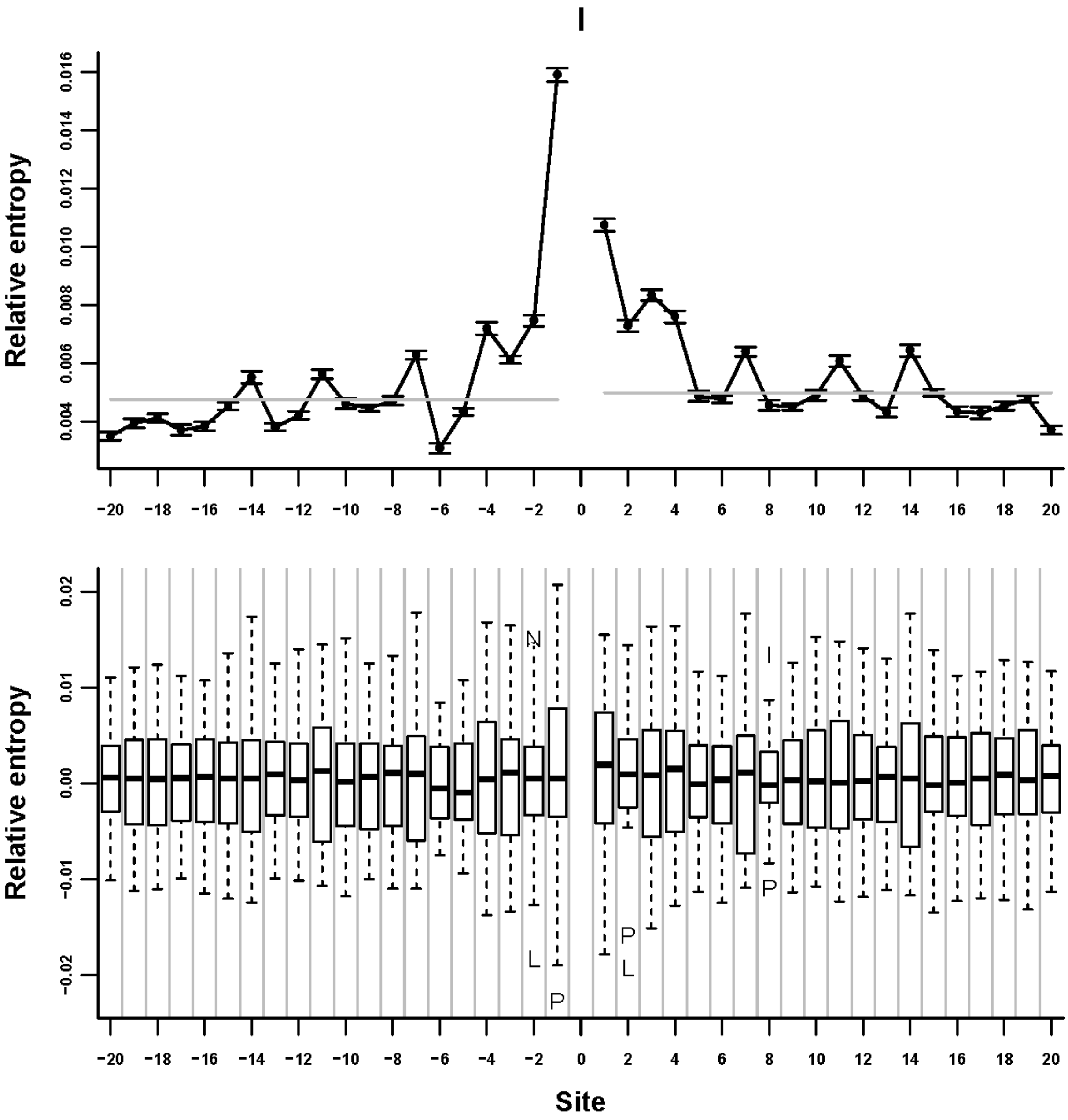
I
Relative entropy
0.004
0.006
0.008
0.010
0.012
0.014
0.016
-20 -18 -16 -14 -12 -10 -8 -6 -4 -2 0 2 4 6 8 10 12 14 16 18 20
Relative entropy
-0.02
-0.01
0.00
0.01
0.02
N
L
P
P
L
I
P
-20 -18 -16 -14 -12 -10 -8 -6 -4 -2 0 2 4 6 8 10 12 14 16 18 20
Site

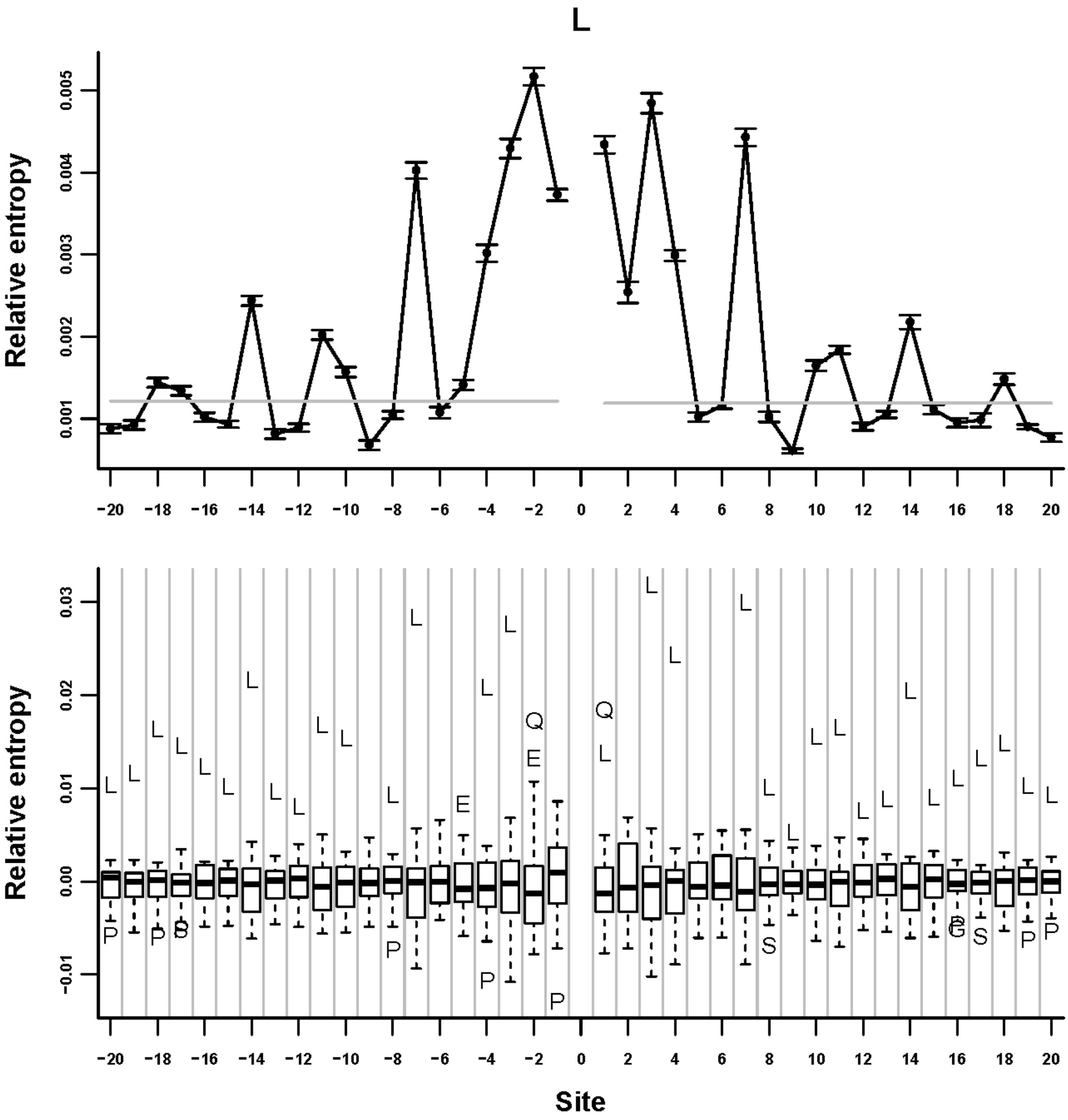
L
Relative entropy
0.001
0.002
0.003
0.004
0.005
-20 -18 -16 -14 -12 -10 -8 -6 -4 -2 0 2 4 6 8 10 12 14 16 18 20
Relative entropy
-0.01
0.00
0.01
0.02
0.03
Site

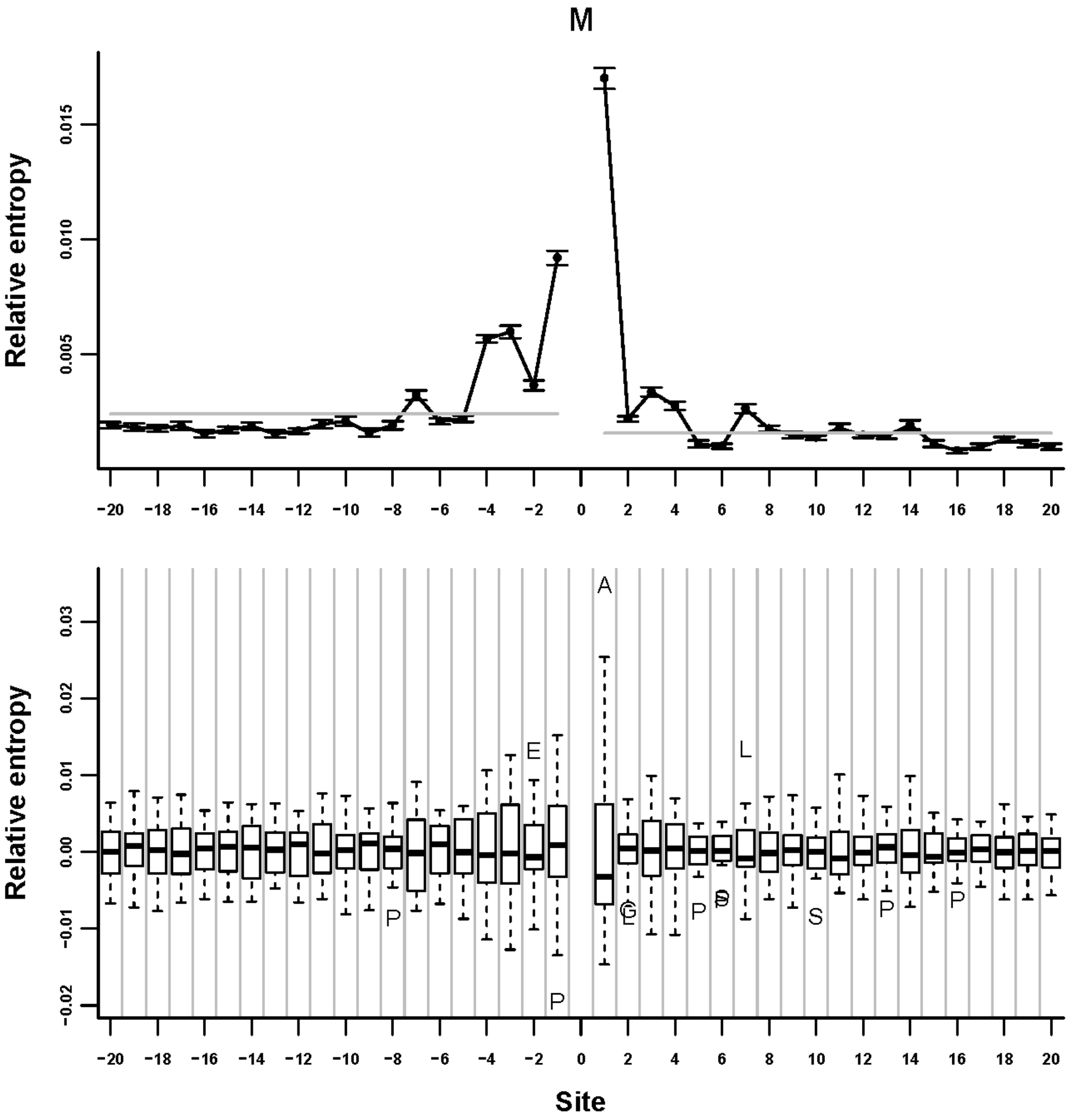
M
Relative entropy
0.005
0.010
0.015
-20 -18 -16 -14 -12 -10 -8 -6 -4 -2 0 2 4 6 8 10 12 14 16 18 20
Relative entropy
-0.02
-0.01
0.00
0.01
0.02
0.03
A
E
L
P
G
E
P
S
P
S
P
P
P
-20 -18 -16 -14 -12 -10 -8 -6 -4 -2 0 2 4 6 8 10 12 14 16 18 20
Site

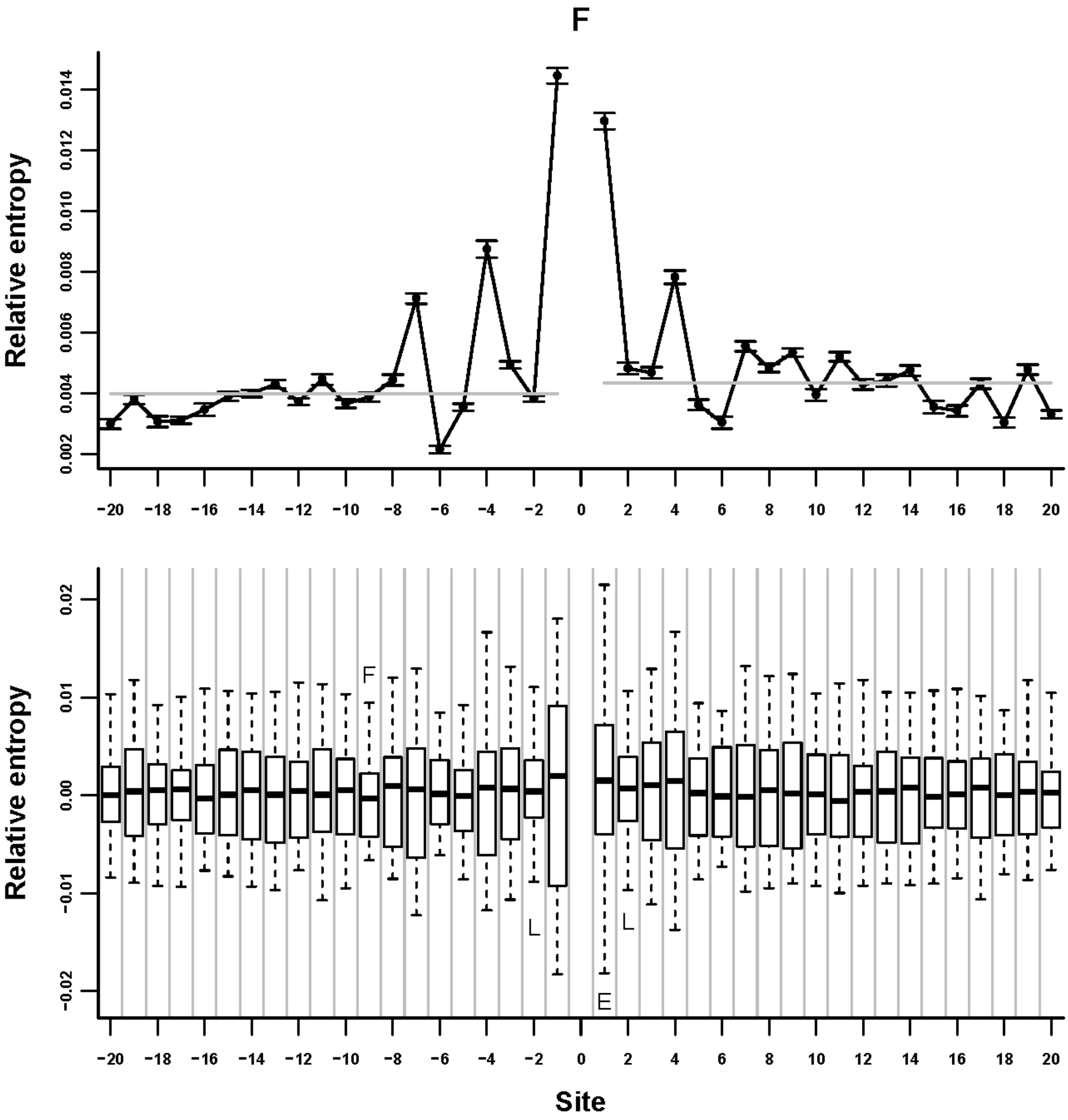
F
Relative entropy
0.002
0.004
0.006
0.008
0.010
0.012
0.014
-20 -18 -16 -14 -12 -10 -8 -6 -4 -2 0 2 4 6 8 10 12 14 16 18 20
Relative entropy
0.02
0.01
0.00
-0.01
-0.02
F
L
L
E
-20 -18 -16 -14 -12 -10 -8 -6 -4 -2 0 2 4 6 8 10 12 14 16 18 20
Site

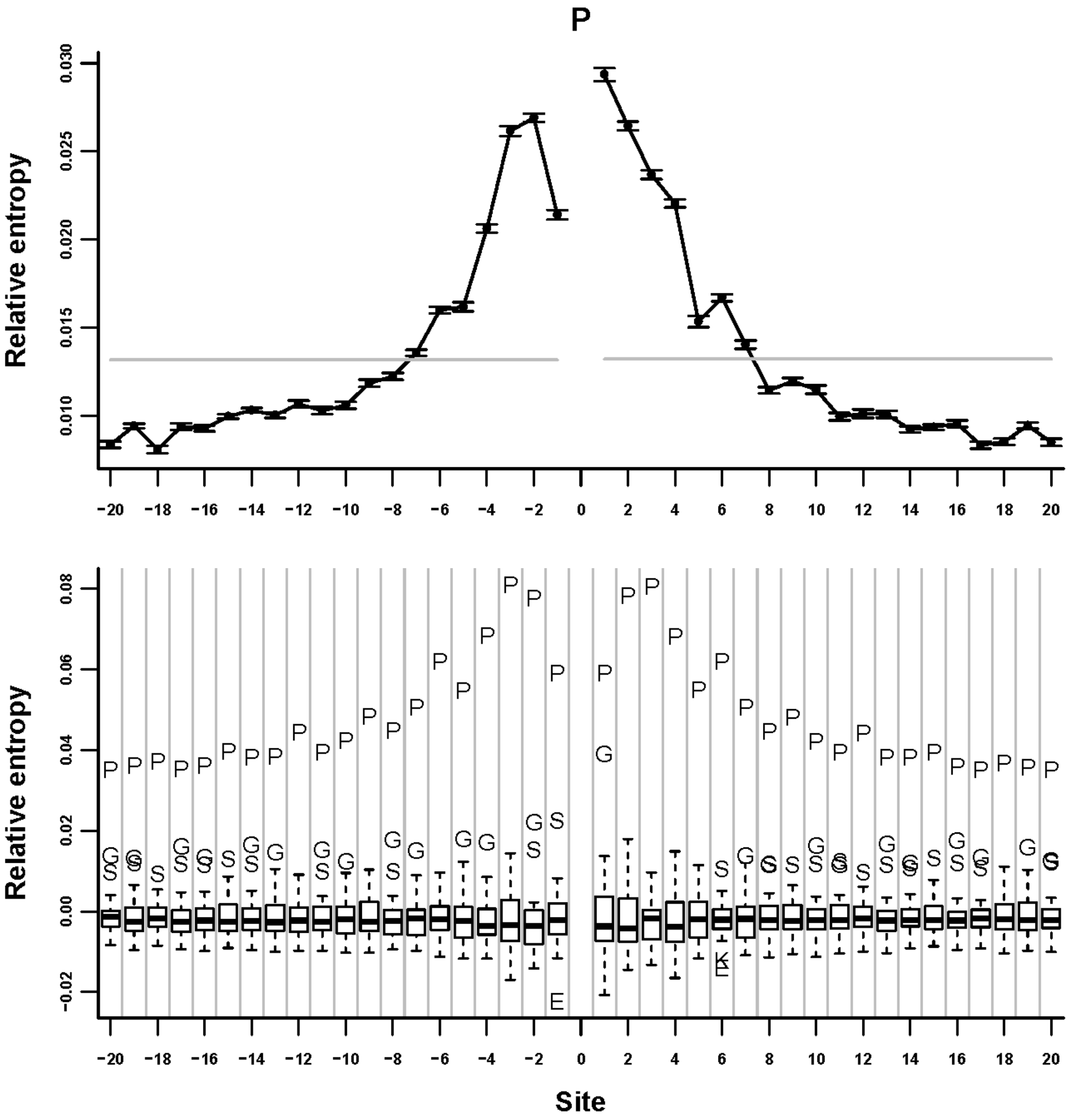
P
Relative entropy
Relative entropy
Site

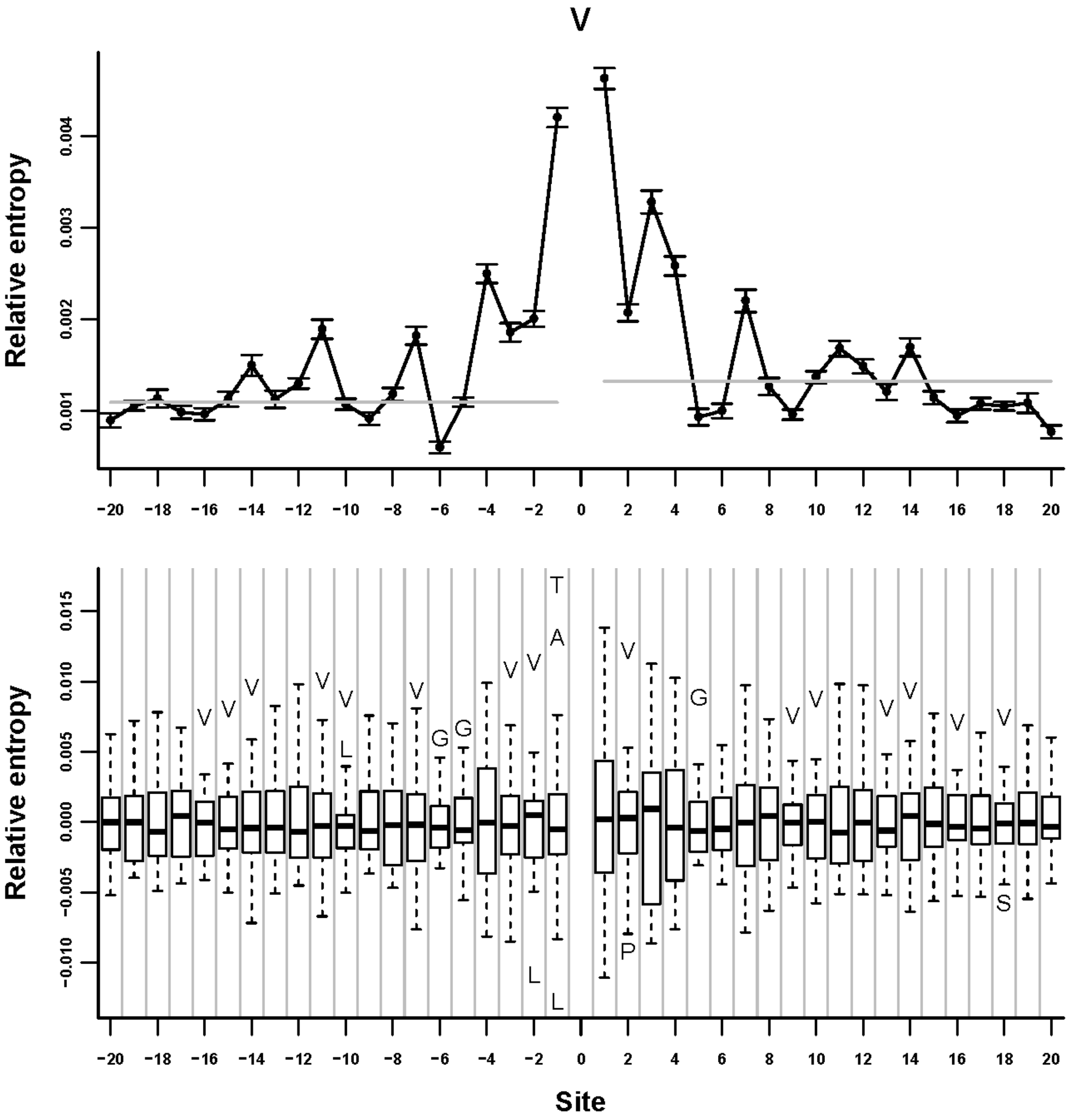
V
Relative entropy
0.001
0.002
0.003
0.004
−20
−18
−16
−14
−12
−10
−8
−6
−4
−2
0
2
4
6
8
10
12
14
16
18
20
Relative entropy
−0.010
−0.005
0.000
0.005
0.010
0.015
Site
T
A
L
L
P
S
G
G
G
L

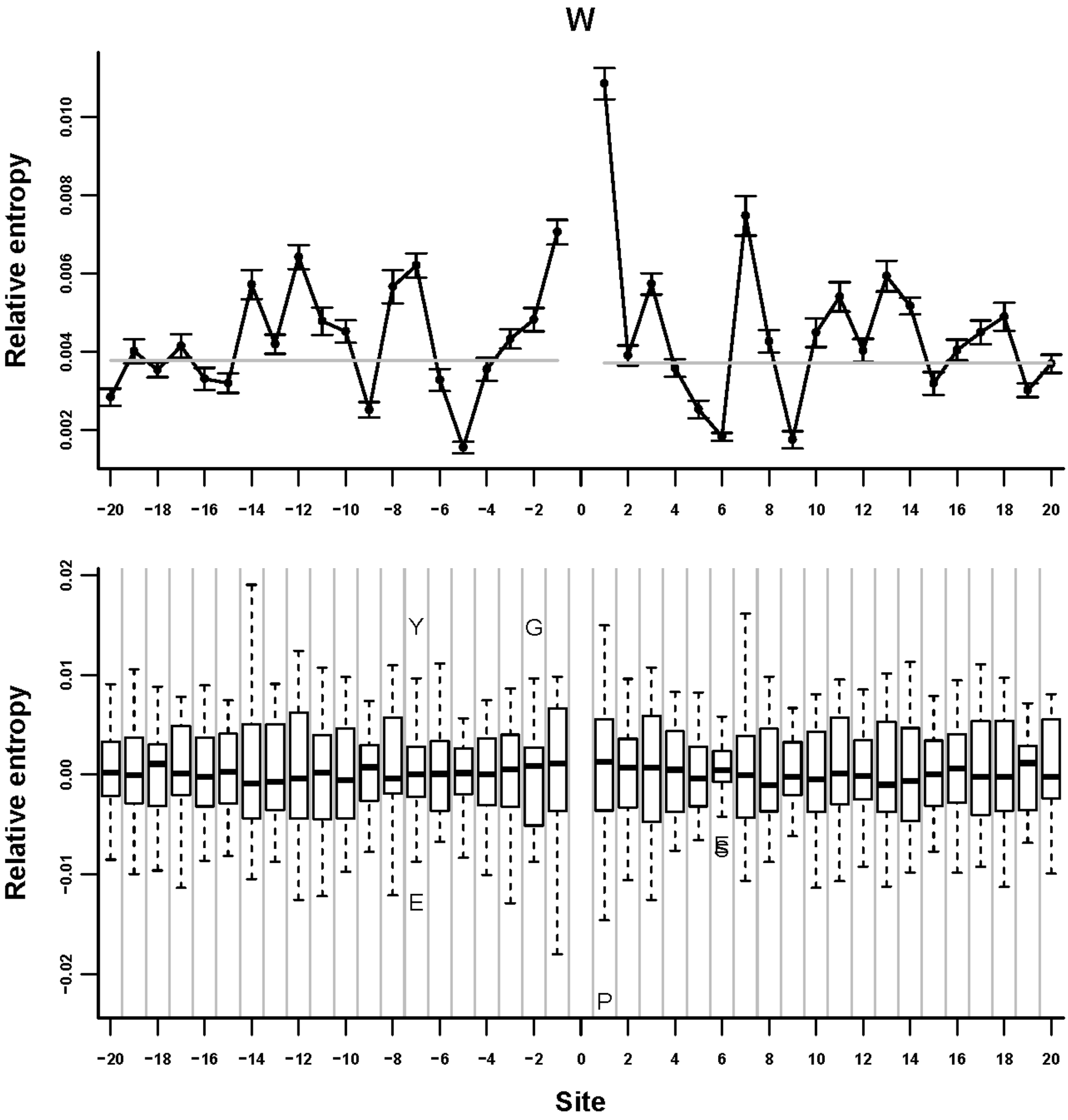
W
Relative entropy
0.002
0.004
0.006
0.008
0.010
-20 -18 -16 -14 -12 -10 -8 -6 -4 -2 0 2 4 6 8 10 12 14 16 18 20
Relative entropy
-0.02
-0.01
0.00
0.01
0.02
Y
G
E
E
S
P
-20 -18 -16 -14 -12 -10 -8 -6 -4 -2 0 2 4 6 8 10 12 14 16 18 20
Site

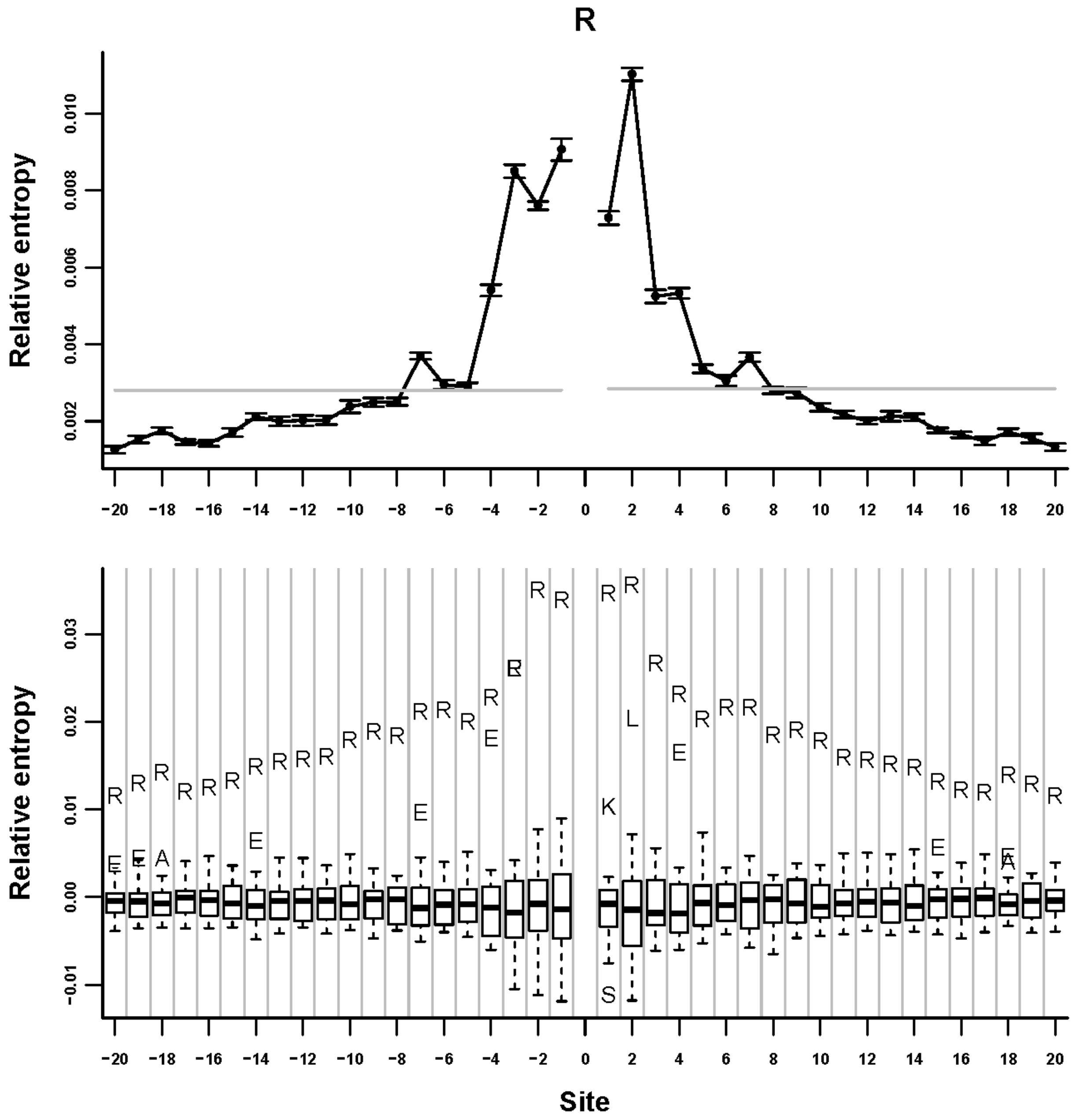
R
Relative entropy
Site

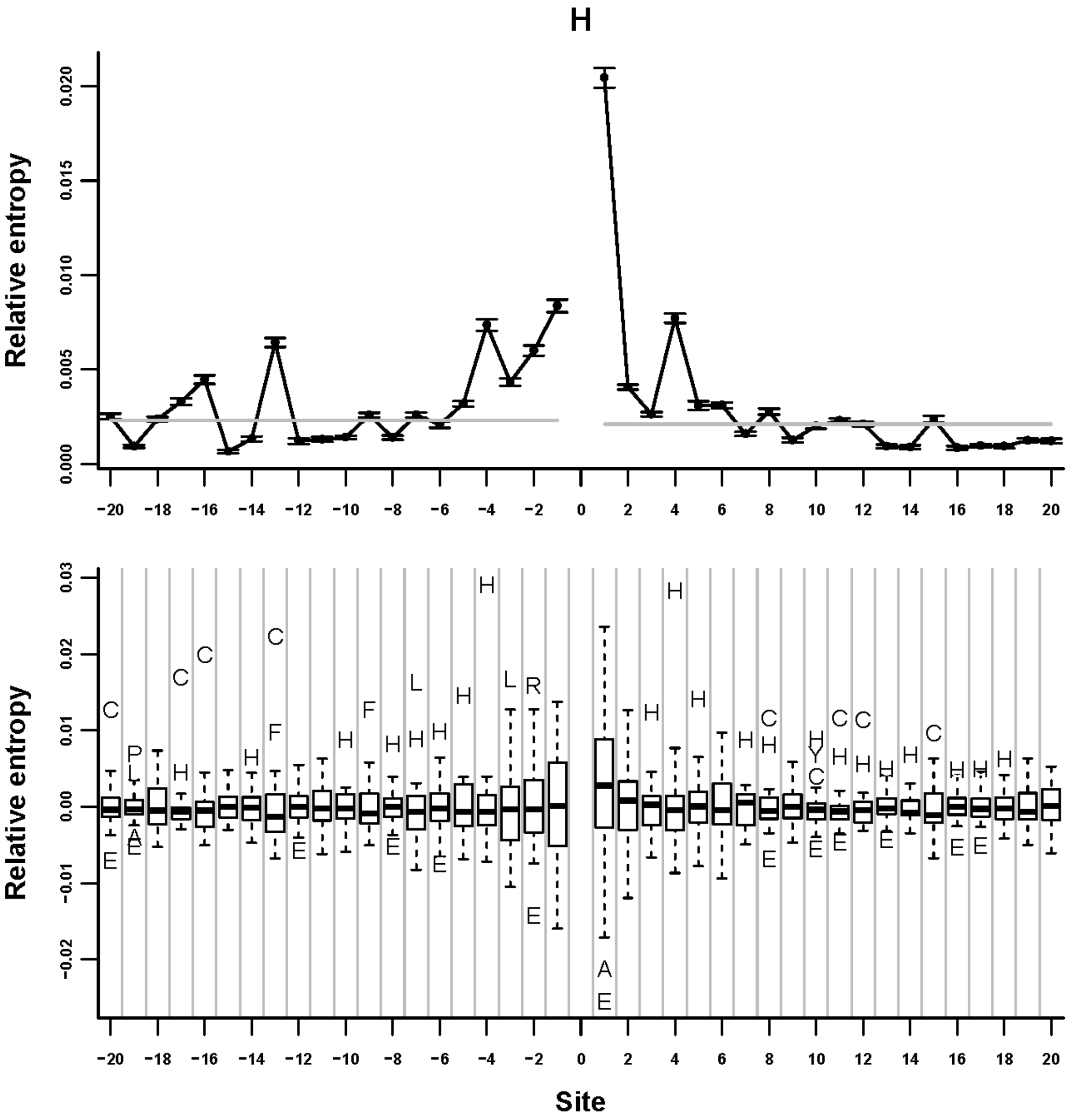
H
Relative entropy
0.000
0.005
0.010
0.015
0.020
-20 -18 -16 -14 -12 -10 -8 -6 -4 -2 0 2 4 6 8 10 12 14 16 18 20
Relative entropy
-0.02
-0.01
0.00
0.01
0.02
0.03
Site

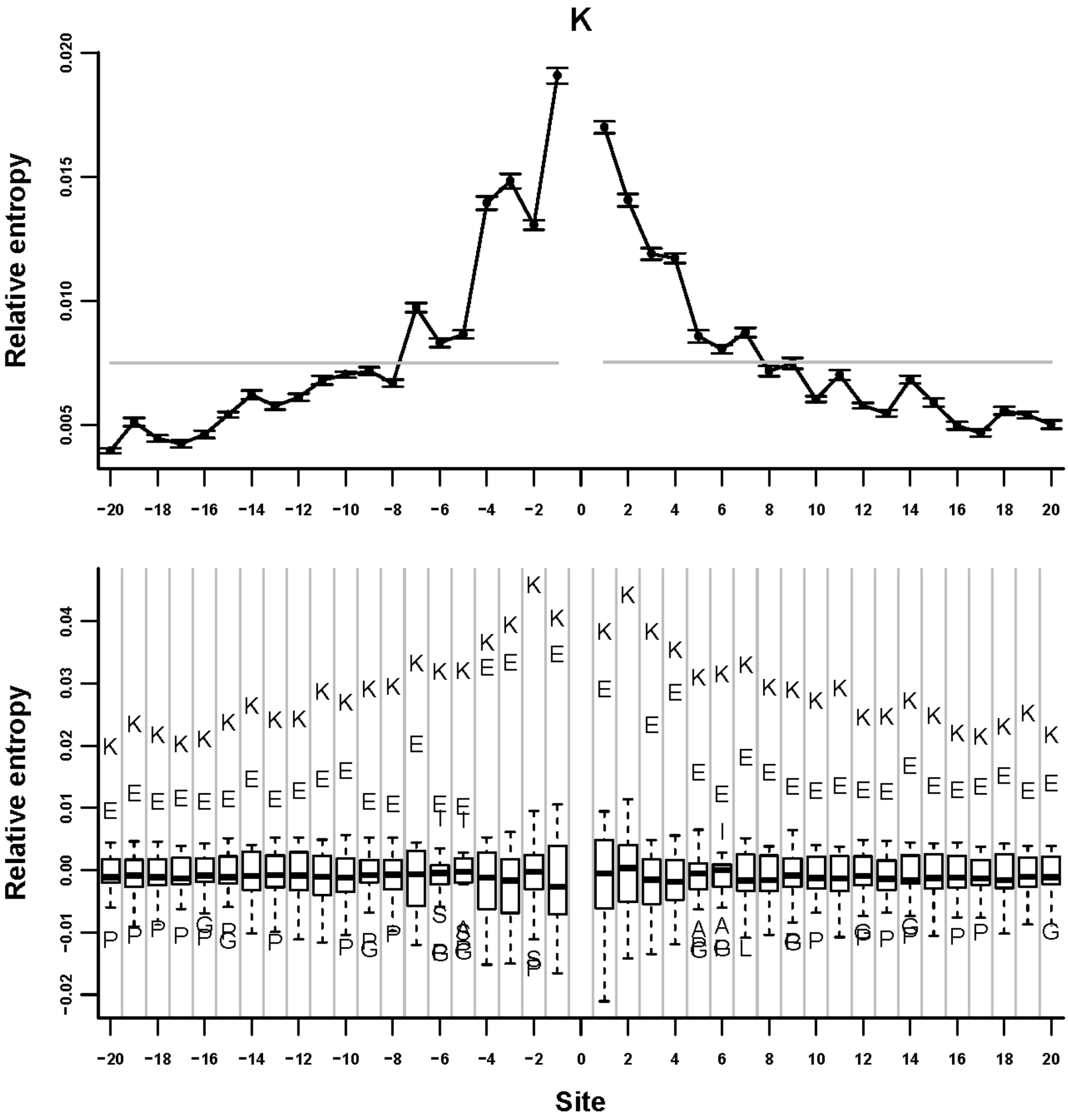
K
Relative entropy
Relative entropy
Site

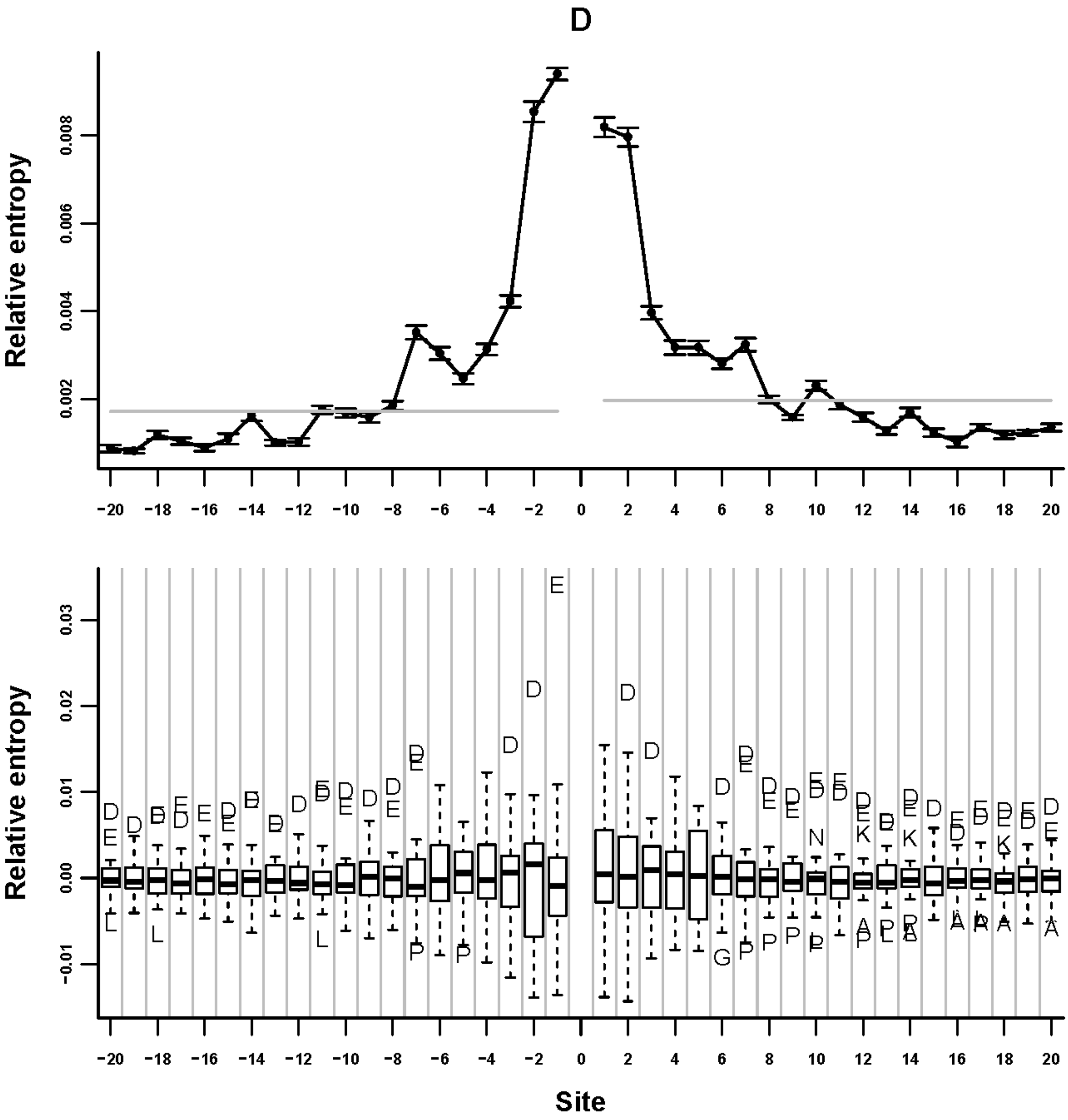
D
Relative entropy
Relative entropy
Site

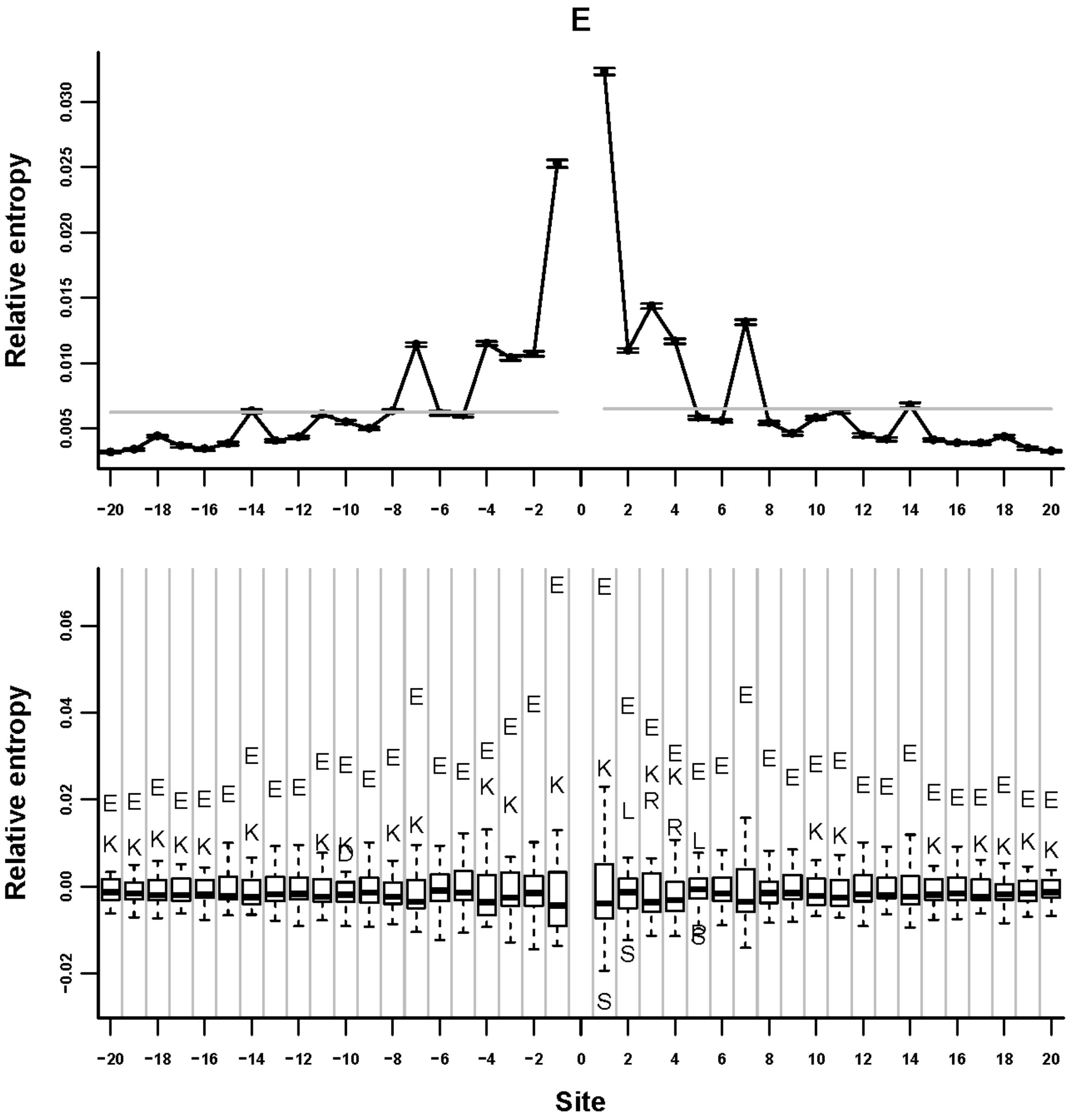
E
Relative entropy
Relative entropy
Site

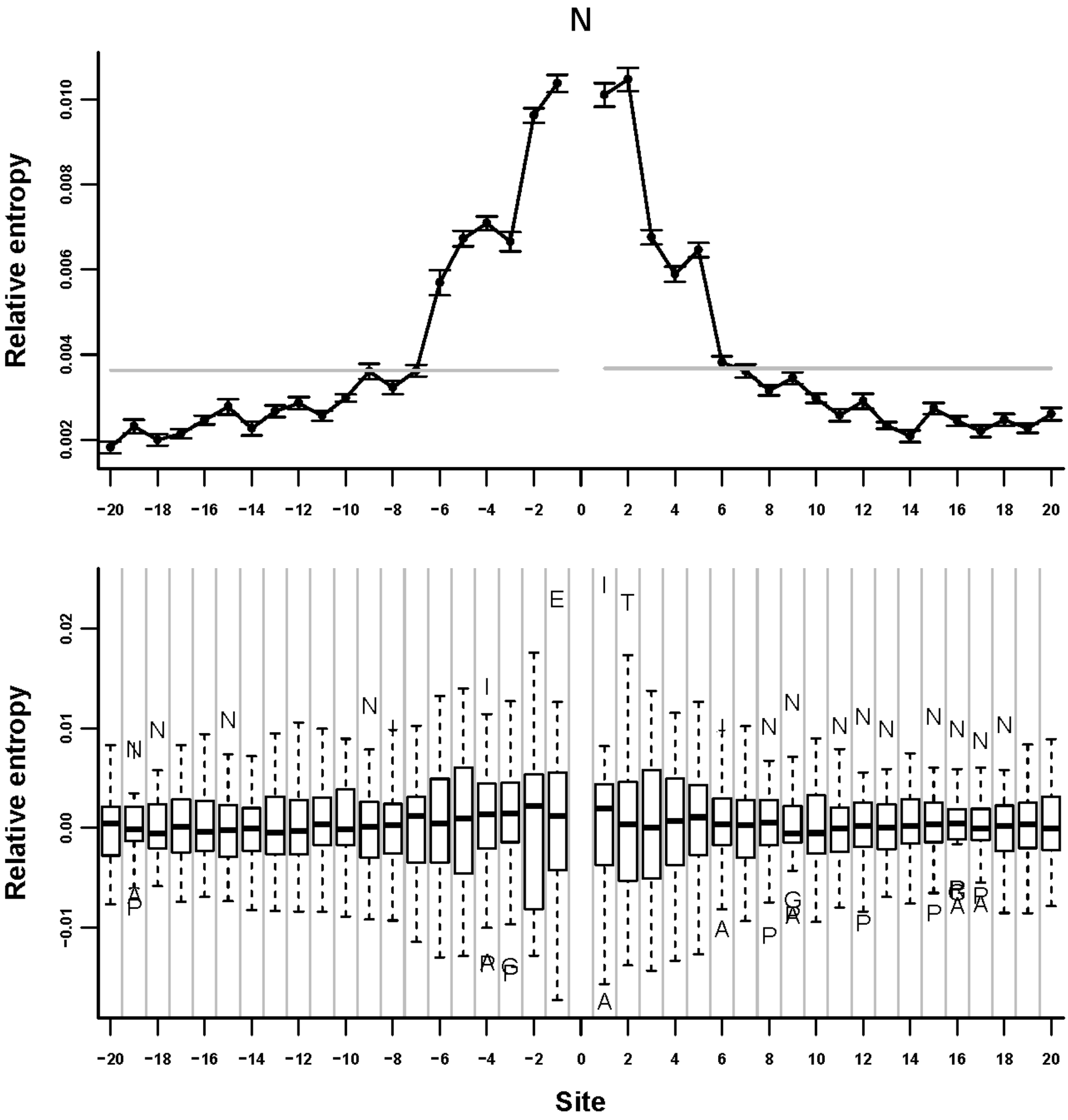
N
Relative entropy
Relative entropy
Site

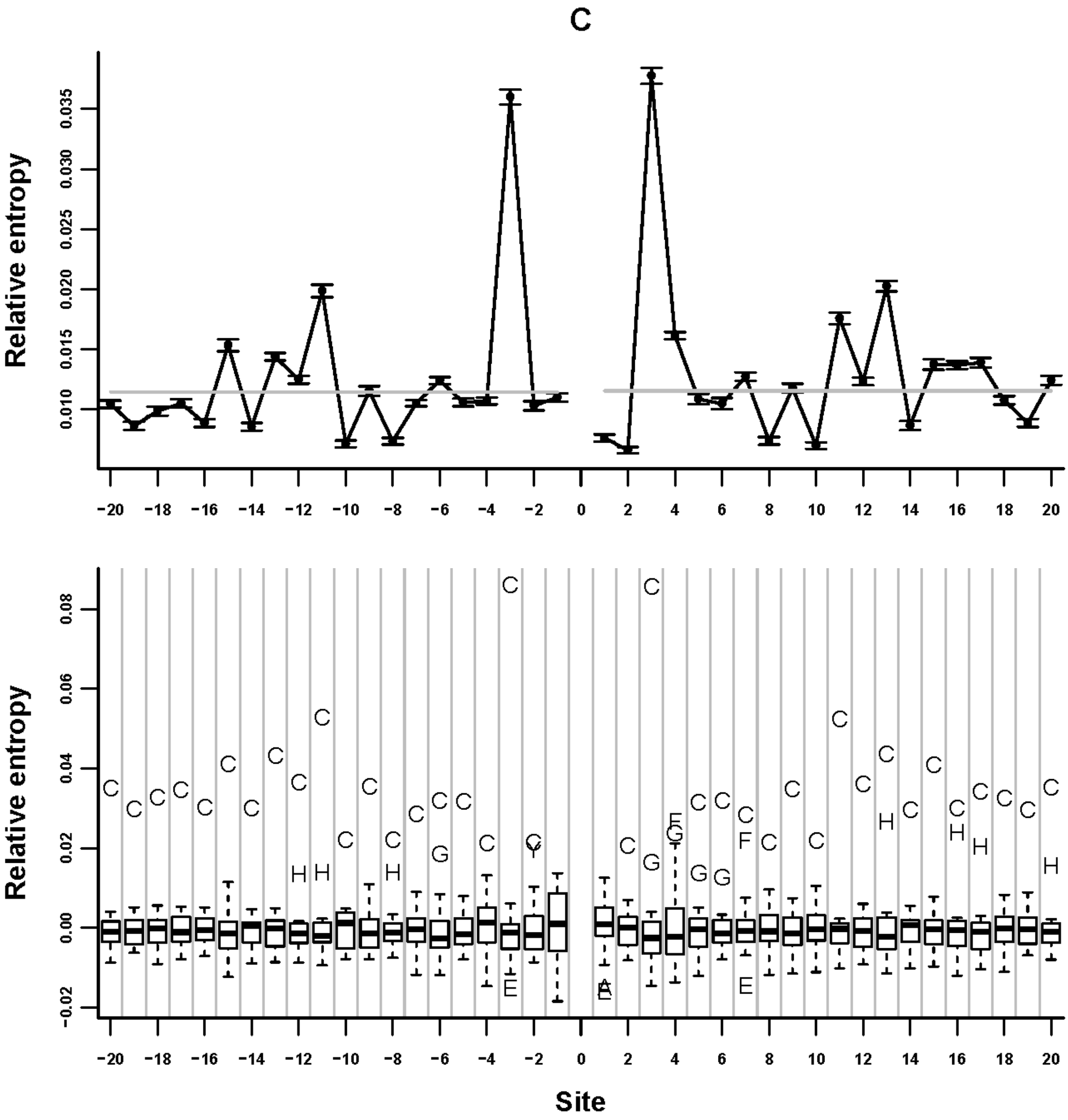
C
Relative entropy
0.010
0.015
0.020
0.025
0.030
0.035
0.00
0.02
0.04
0.06
0.08
-0.02
−20 −18 −16 −14 −12 −10 −8 −6 −4 −2 0 2 4 6 8 10 12 14 16 18 20
Site

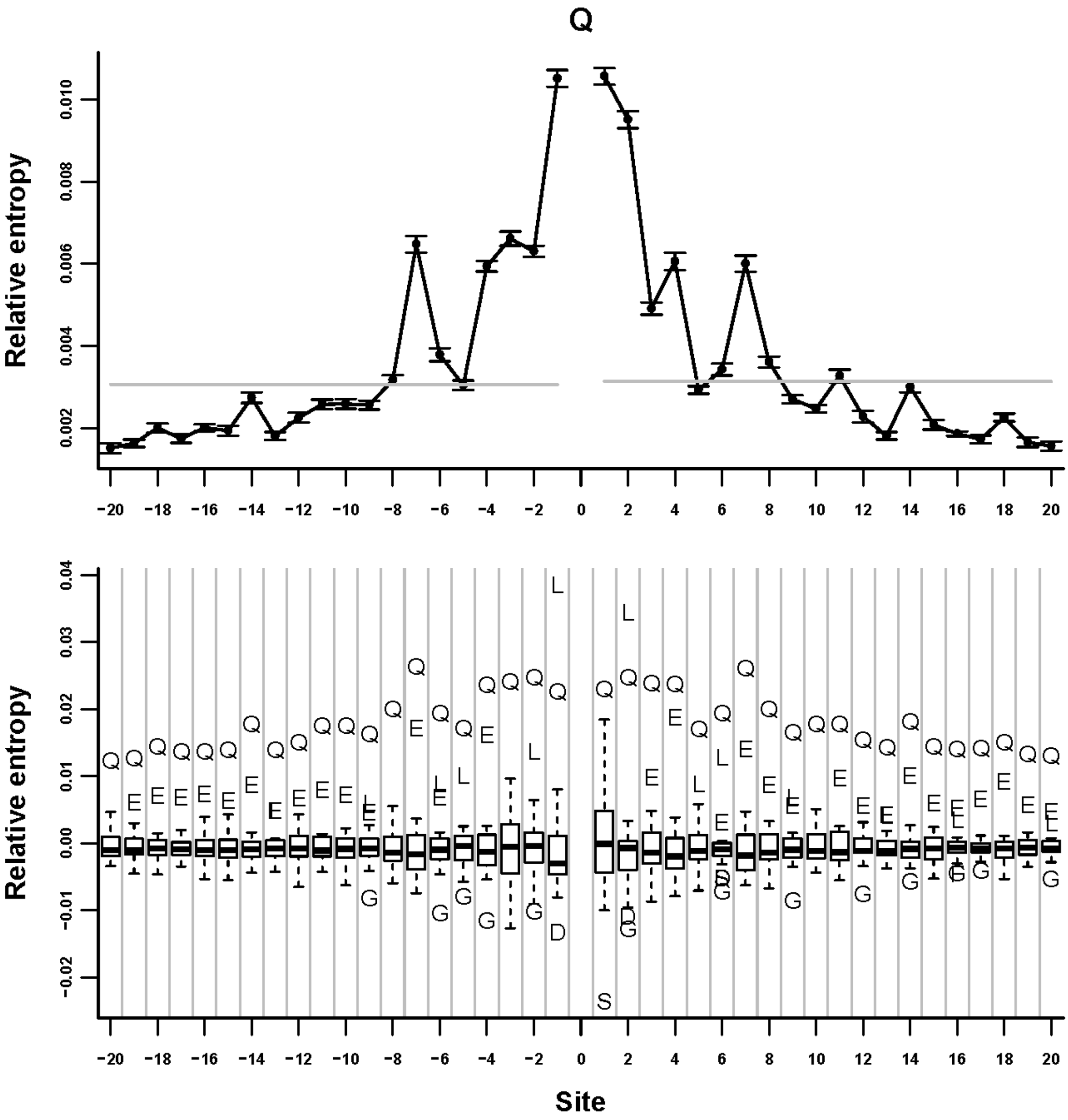
Q
Relative entropy
Site

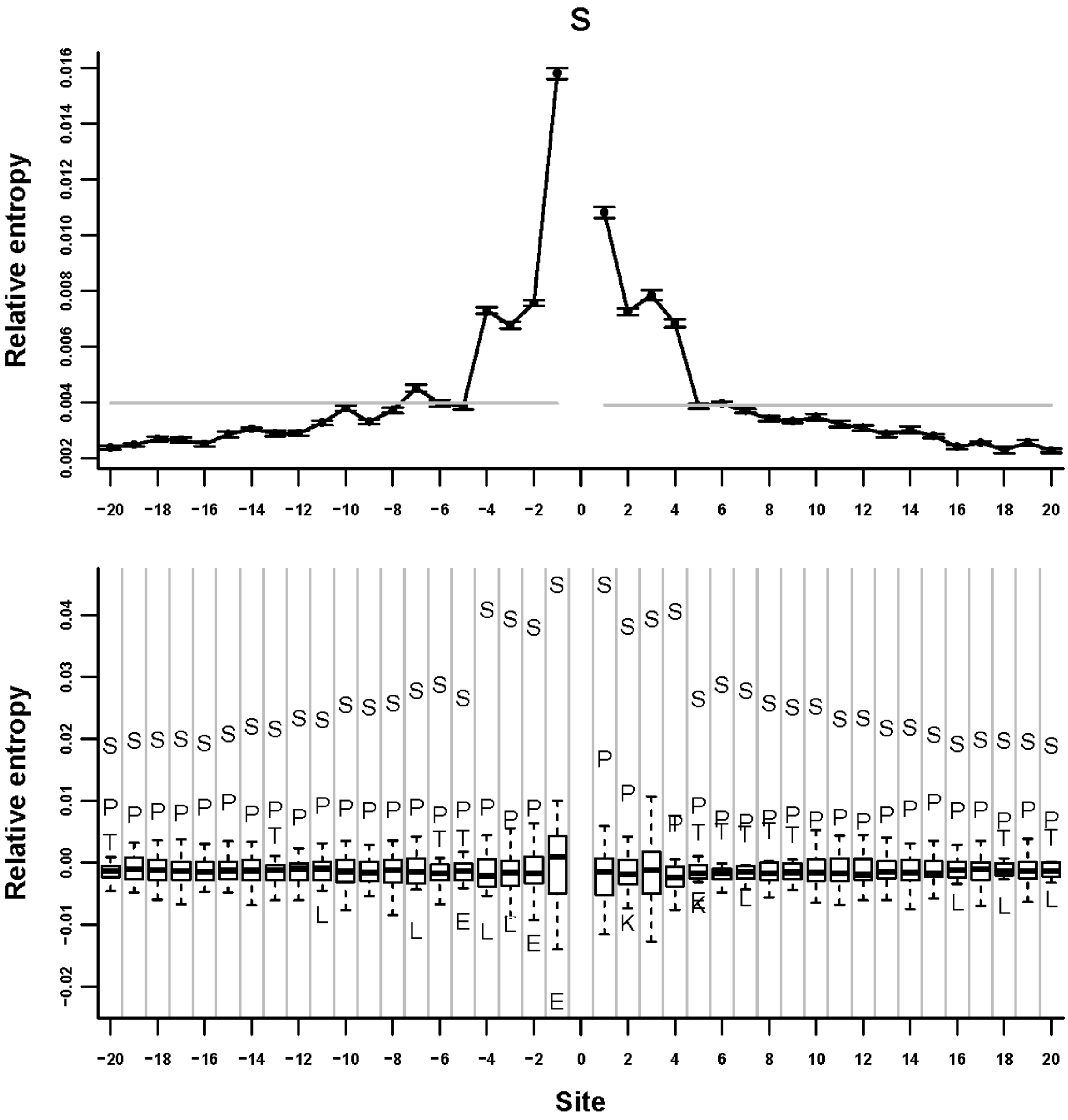
S
Relative entropy
Relative entropy
Site

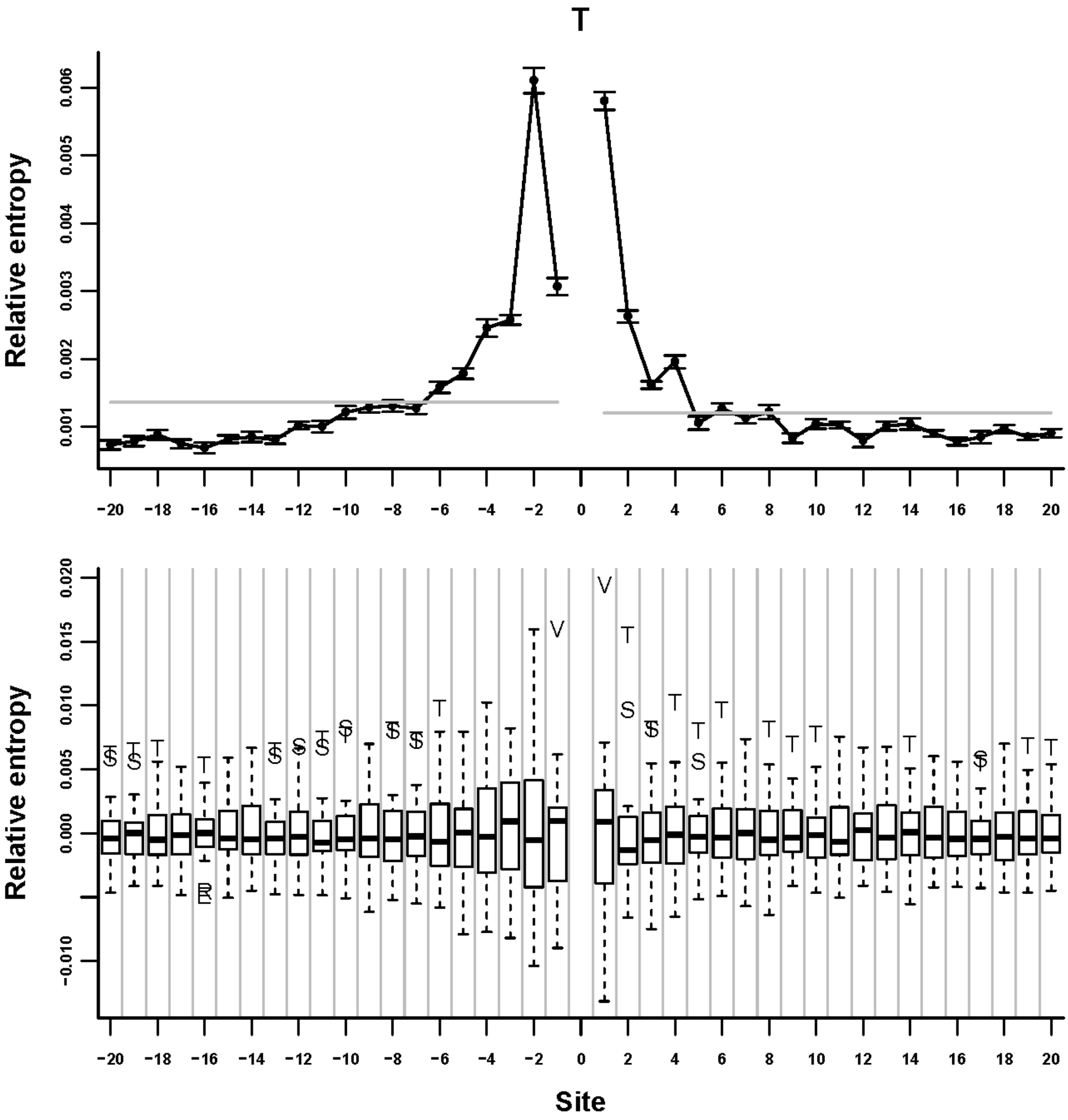
T
Relative entropy
Site
V
T
S
R
E

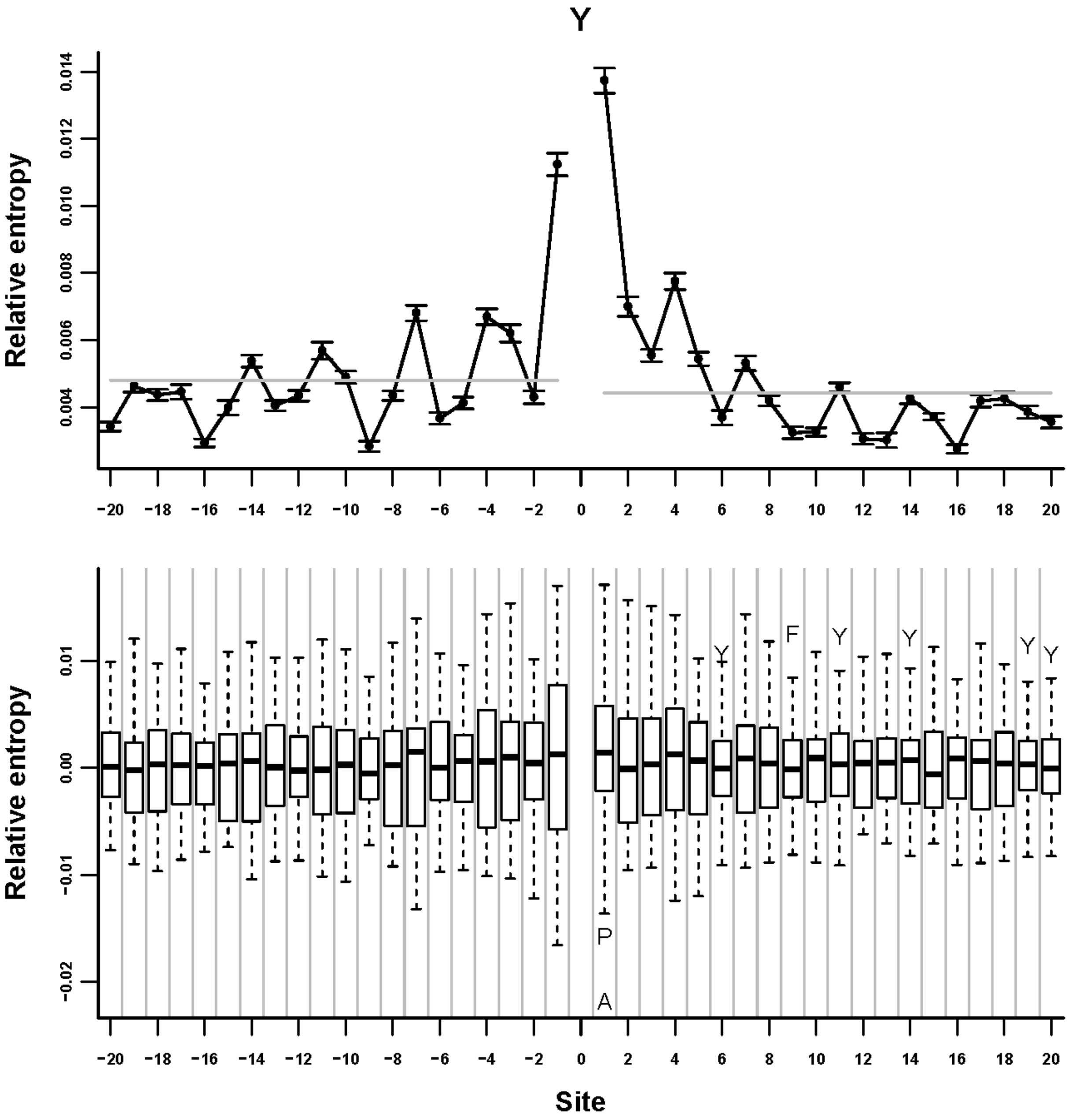
Y
Relative entropy
0.004
0.006
0.008
0.010
0.012
0.014
-20 -18 -16 -14 -12 -10 -8 -6 -4 -2 0 2 4 6 8 10 12 14 16 18 20
Relative entropy
-0.02
-0.01
0.00
0.01
Y F Y Y Y Y
P
A
-20 -18 -16 -14 -12 -10 -8 -6 -4 -2 0 2 4 6 8 10 12 14 16 18 20
Site

Figure S2. Neighbor preference patterns of the 20 amino acids in α-helix.

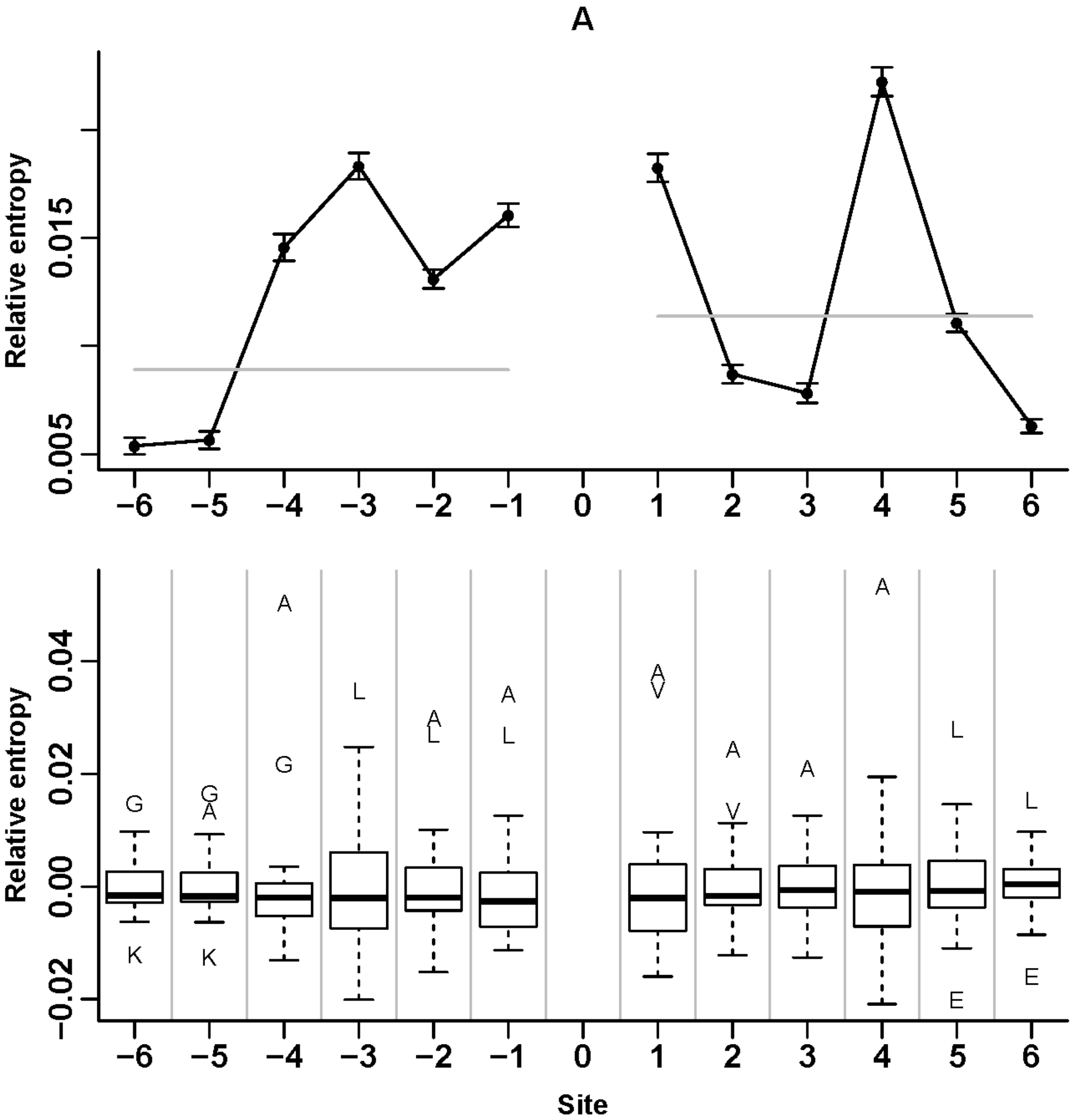

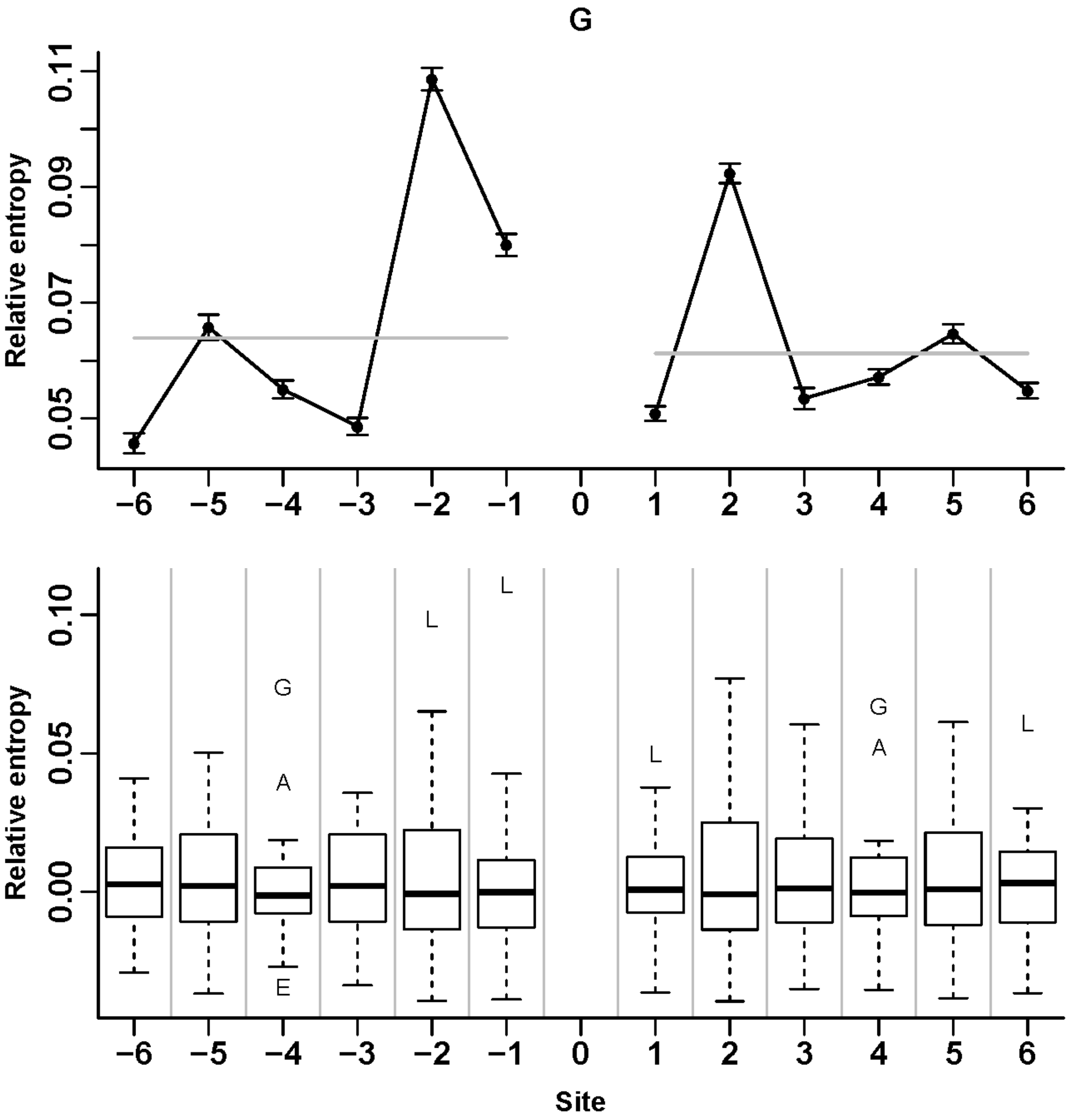
G
Relative entropy
0.05
0.07
0.09
0.11
−6
−5
−4
−3
−2
−1
0
1
2
3
4
5
6
Relative entropy
0.00
0.05
0.10
G
A
E
L
L
L
G
A
L
−6
−5
−4
−3
−2
−1
0
1
2
3
4
5
6
Site

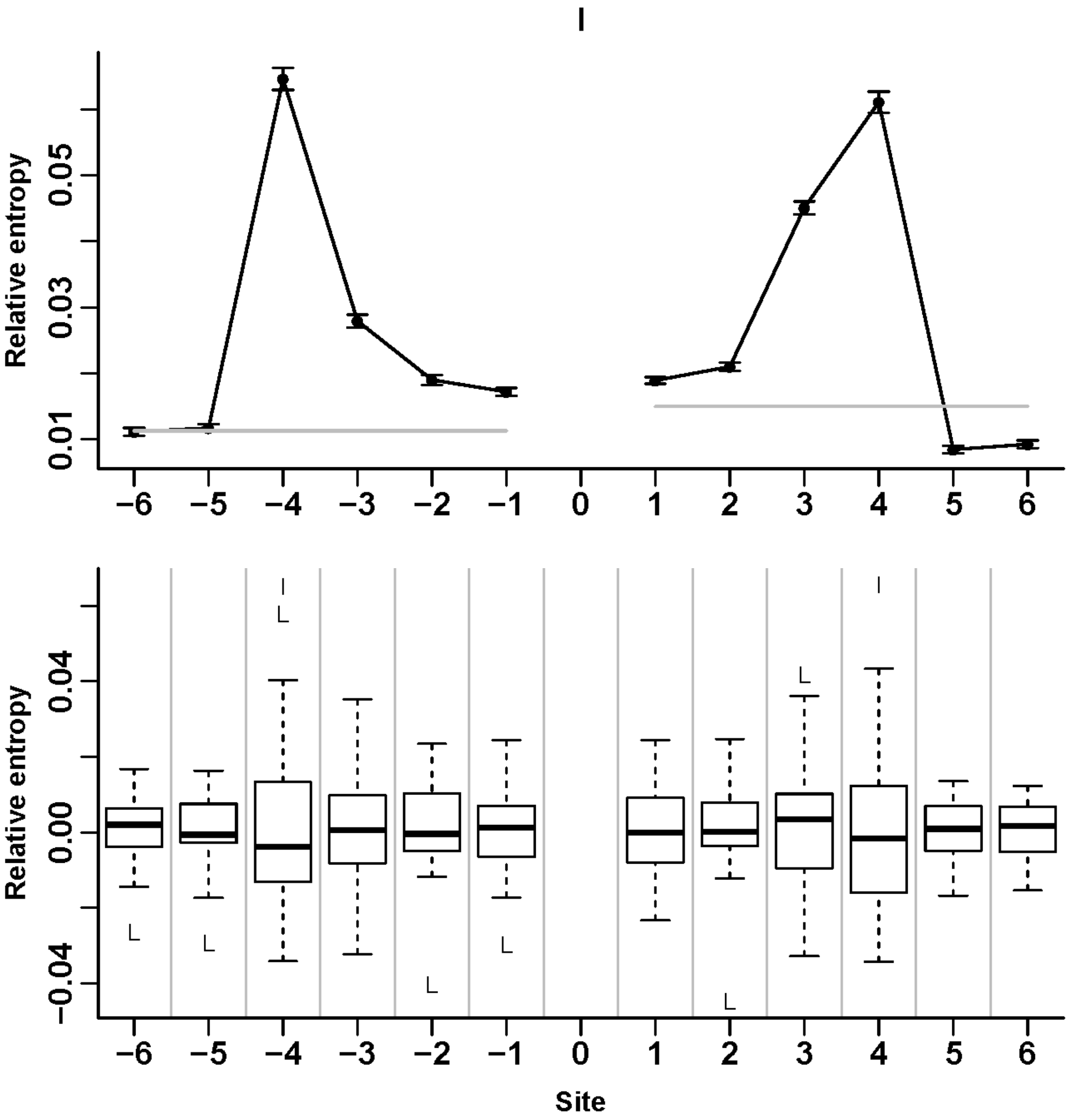
I
Relative entropy
0.01
0.03
0.05
−6
−5
−4
−3
−2
−1
0
1
2
3
4
5
6
Relative entropy
−0.04
0.00
0.04
I
L
L
L
L
L
L
L
L
I
Site

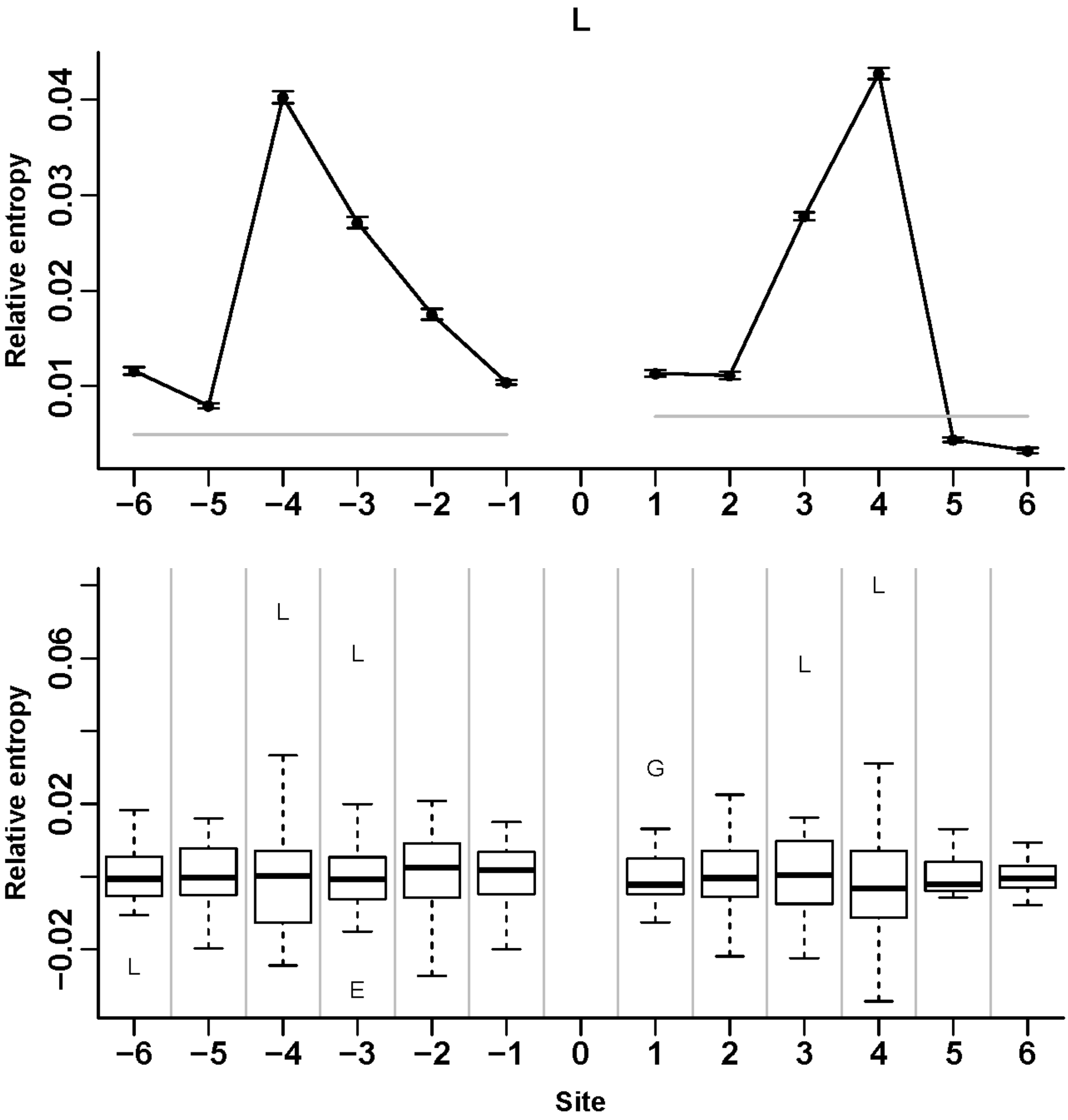
L
Relative entropy
0.01
0.02
0.03
0.04
−6
−5
−4
−3
−2
−1
0
1
2
3
4
5
6
Relative entropy
−0.02
0.02
0.06
L
L
L
E
G
L
L
Site

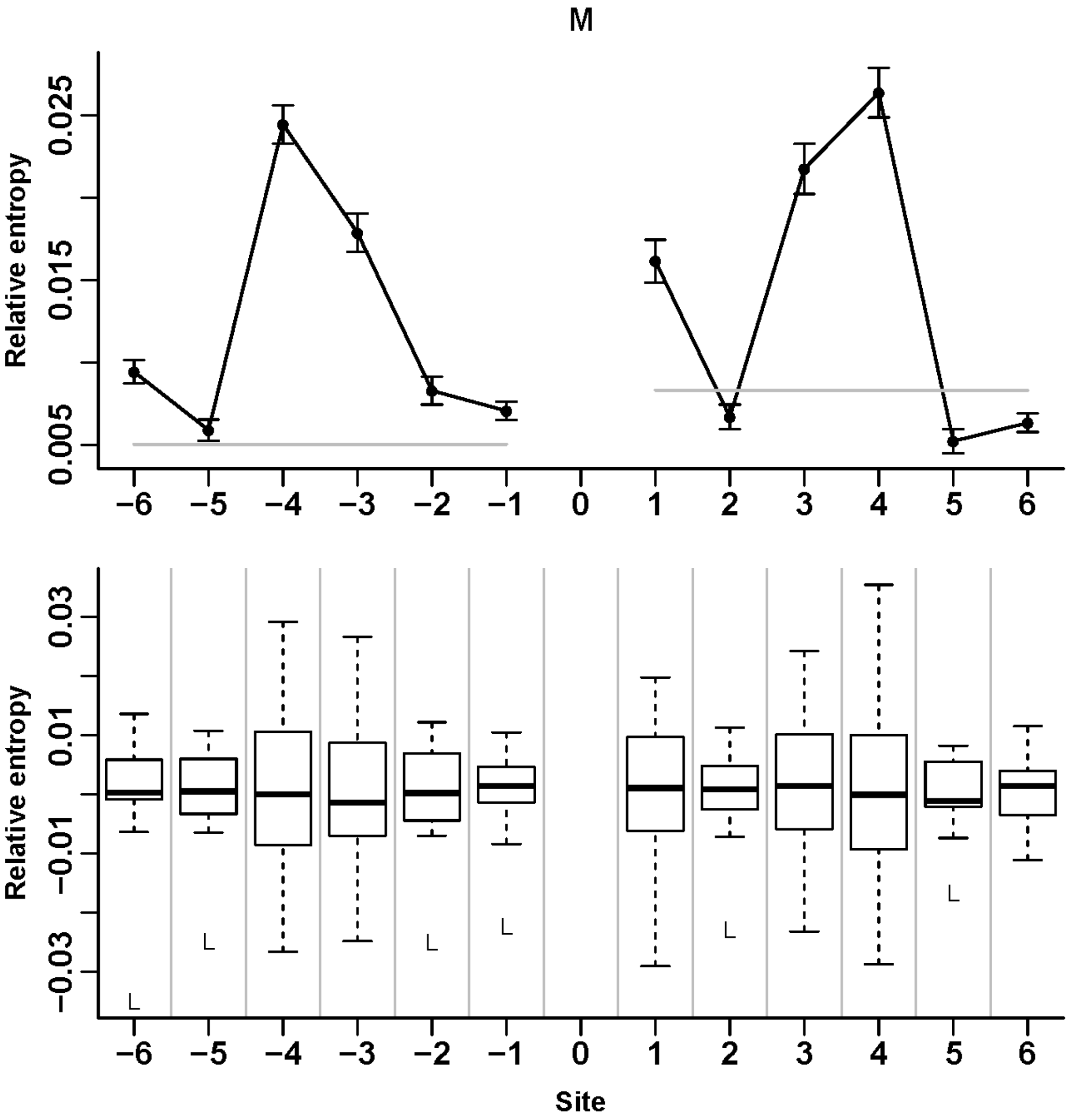
M
Relative entropy
0.005
0.015
0.025
−6
−5
−4
−3
−2
−1
0
1
2
3
4
5
6
Relative entropy
−0.03
−0.01
0.01
0.03
Site

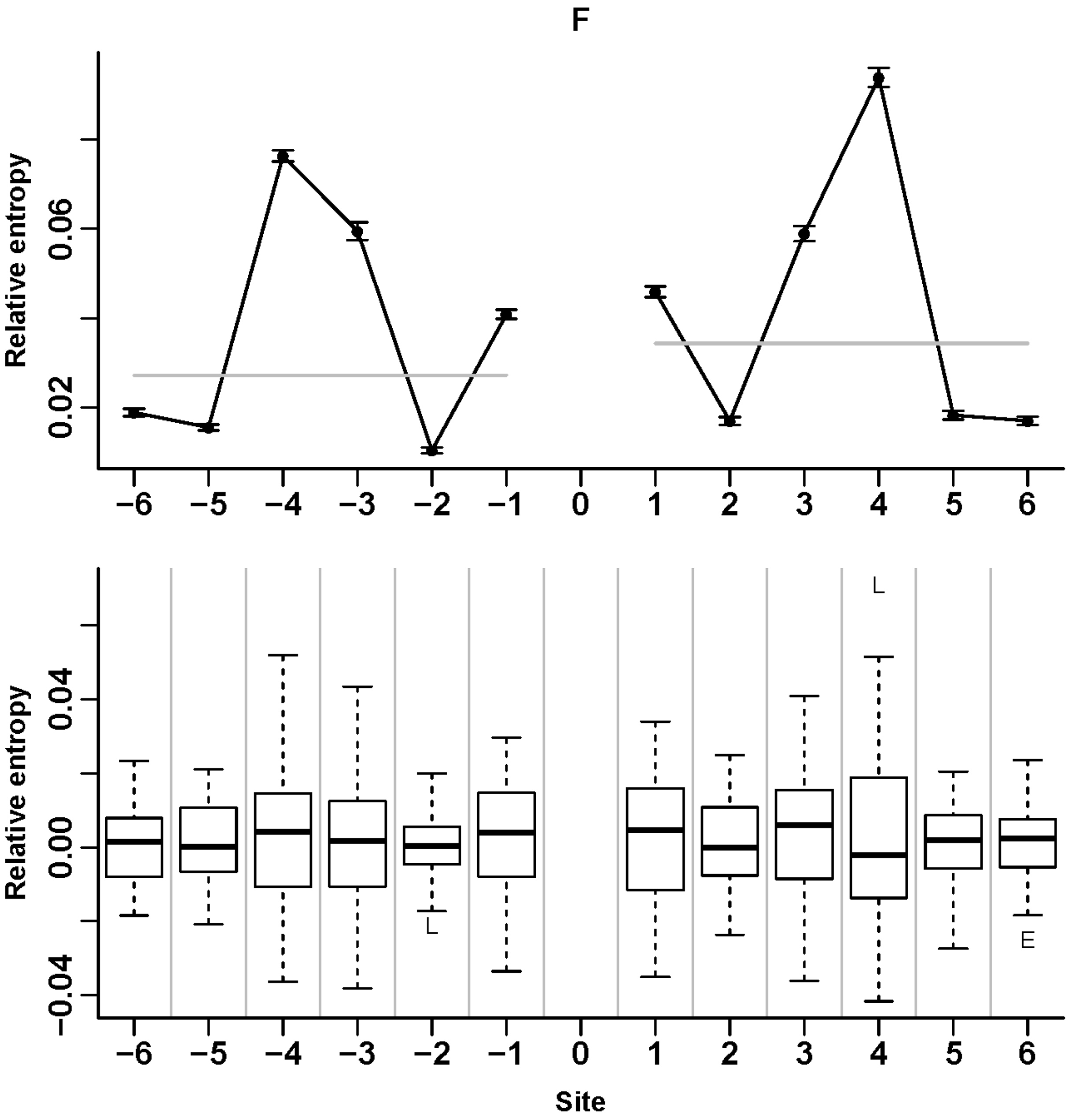
F
Relative entropy
0.02
0.06
−6
−5
−4
−3
−2
−1
0
1
2
3
4
5
6
Relative entropy
−0.04
0.00
0.04
L
L
E
Site

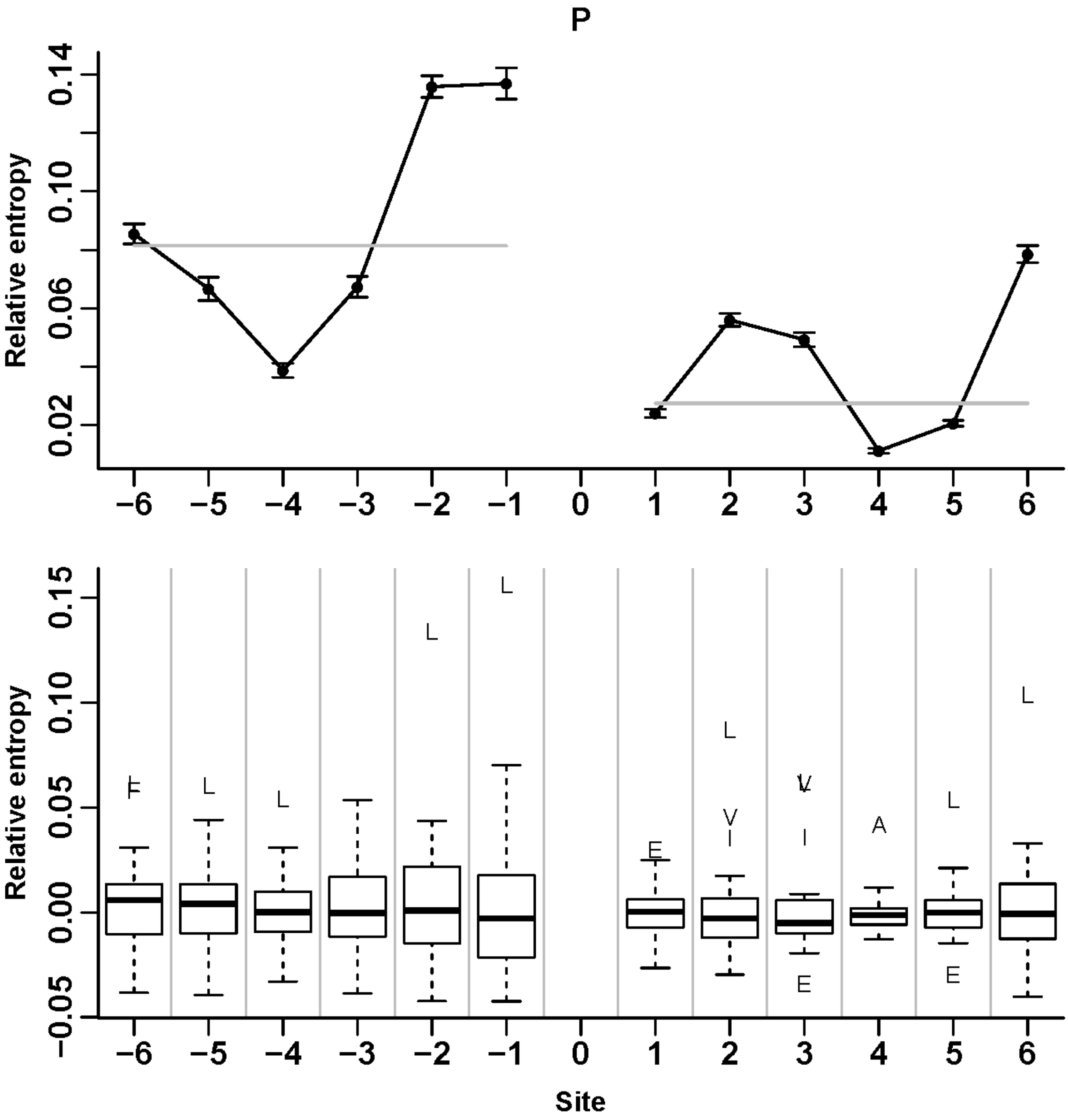
P
Relative entropy
0.02
0.06
0.10
0.14
−6
−5
−4
−3
−2
−1
0
1
2
3
4
5
6
Relative entropy
−0.05
0.00
0.05
0.10
0.15
L
L
L
F
L
L
E
L
V
I
V
I
E
A
L
E
L
Site

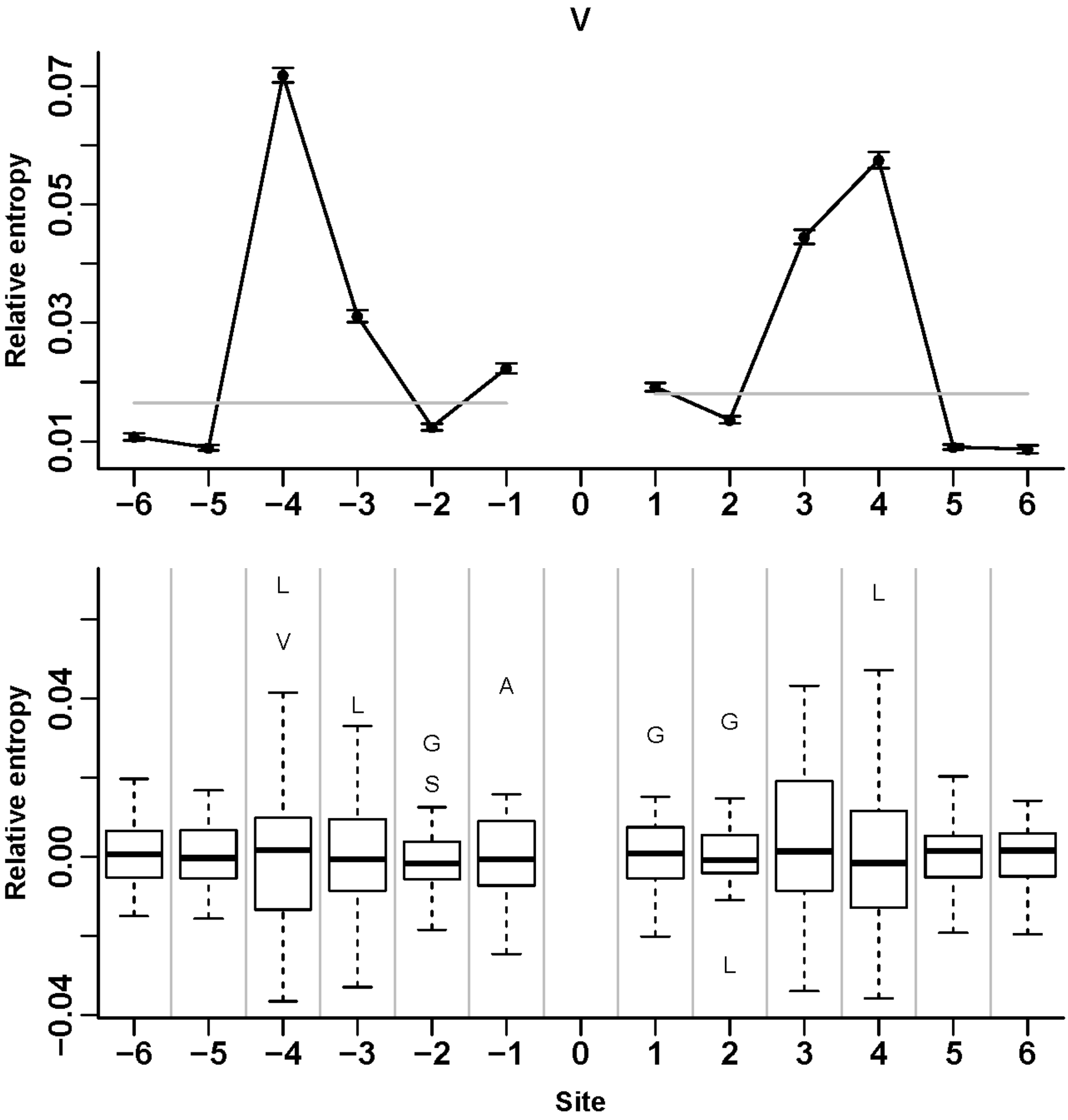
V
Relative entropy
0.07
0.05
0.03
0.01
−6
−5
−4
−3
−2
−1
0
1
2
3
4
5
6
L
V
L
A
G
S
G
G
L
L
0.04
0.00
−0.04
Site

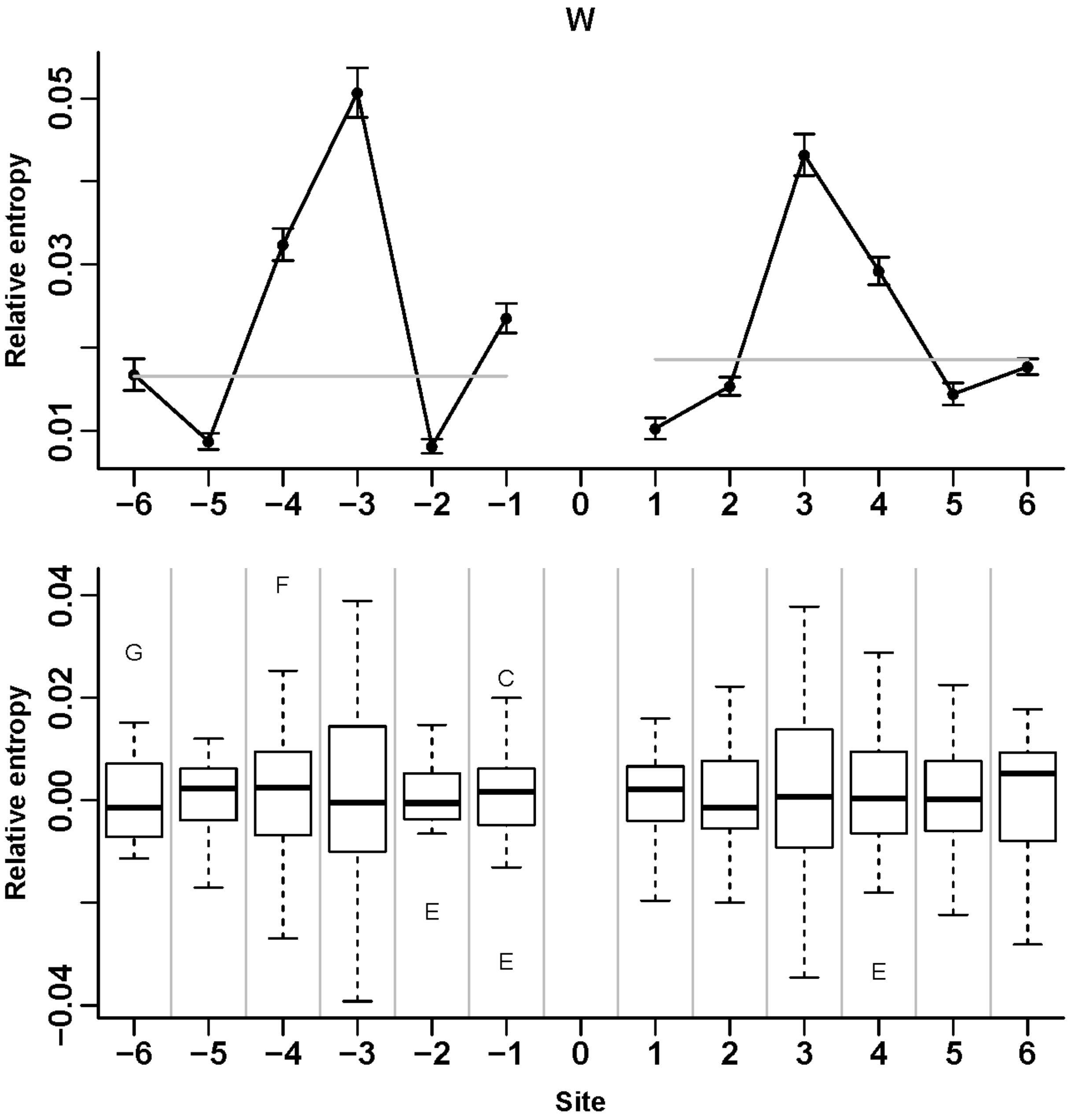
W
Relative entropy
0.01
0.03
0.05
−6
−5
−4
−3
−2
−1
0
1
2
3
4
5
6
Relative entropy
−0.04
0.00
0.02
0.04
G
F
C
E
E
E
Site

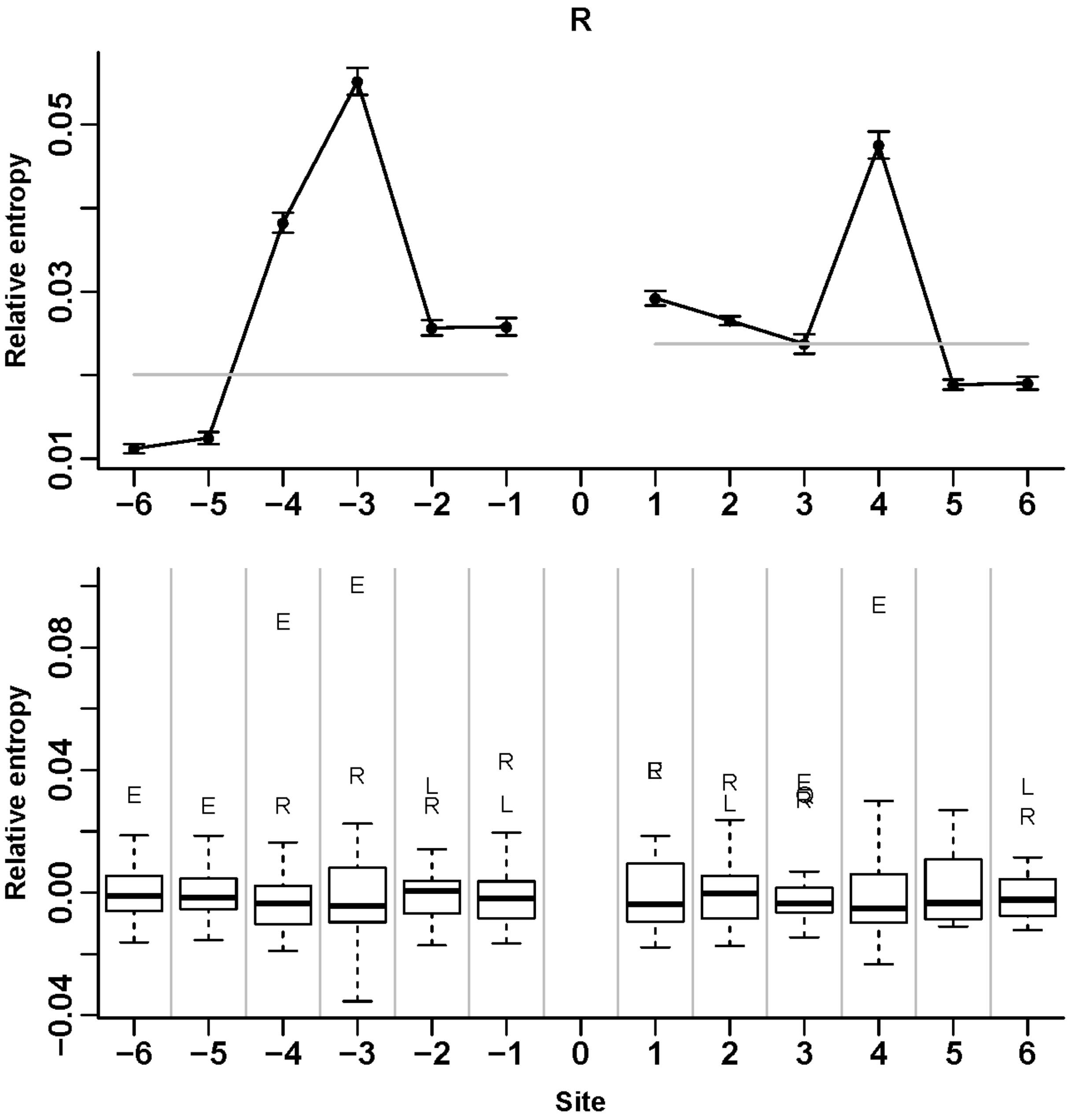
R
Relative entropy
0.01
0.03
0.05
−6
−5
−4
−3
−2
−1
0
1
2
3
4
5
6
Relative entropy
−0.04
0.00
0.04
0.08
Site

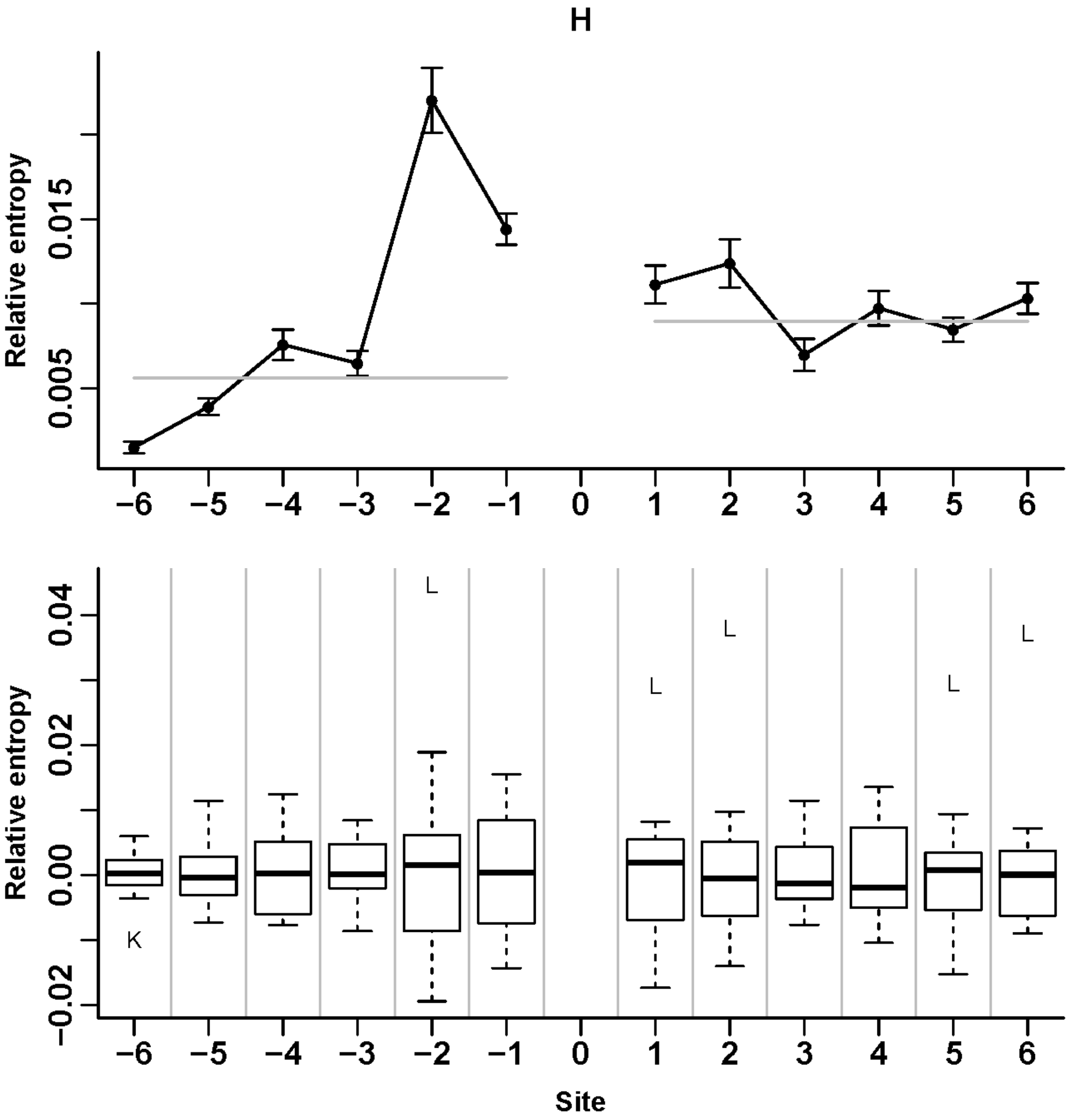
H
Relative entropy
0.005
0.015
−6
−5
−4
−3
−2
−1
0
1
2
3
4
5
6
Relative entropy
−0.02
0.00
0.02
0.04
K
L
L
L
L
L
L
Site

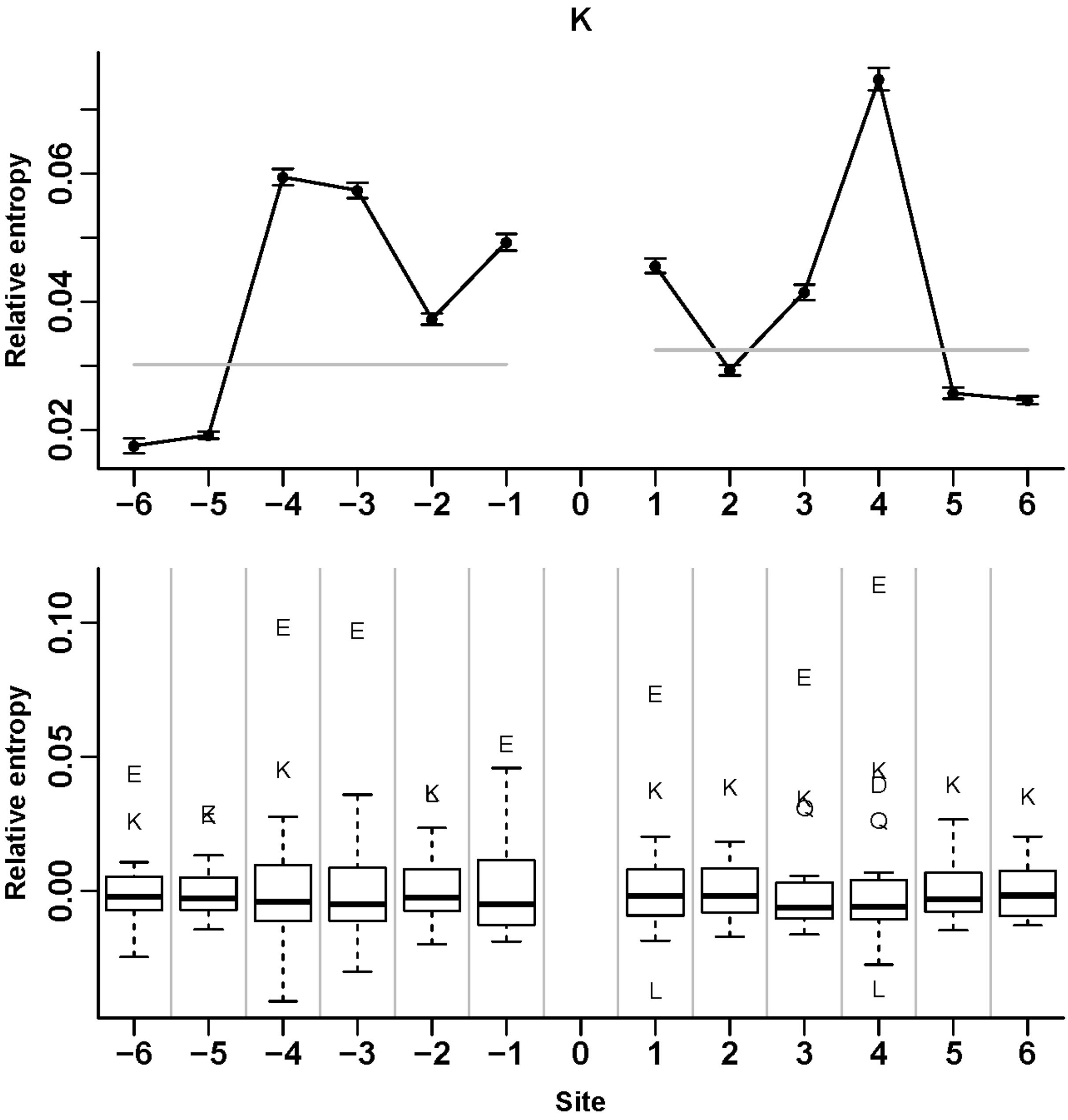
K
Relative entropy
0.02
0.04
0.06
0.00
0.05
0.10
−6 −5 −4 −3 −2 −1 0 1 2 3 4 5 6
Site
E
K
L
D
Q

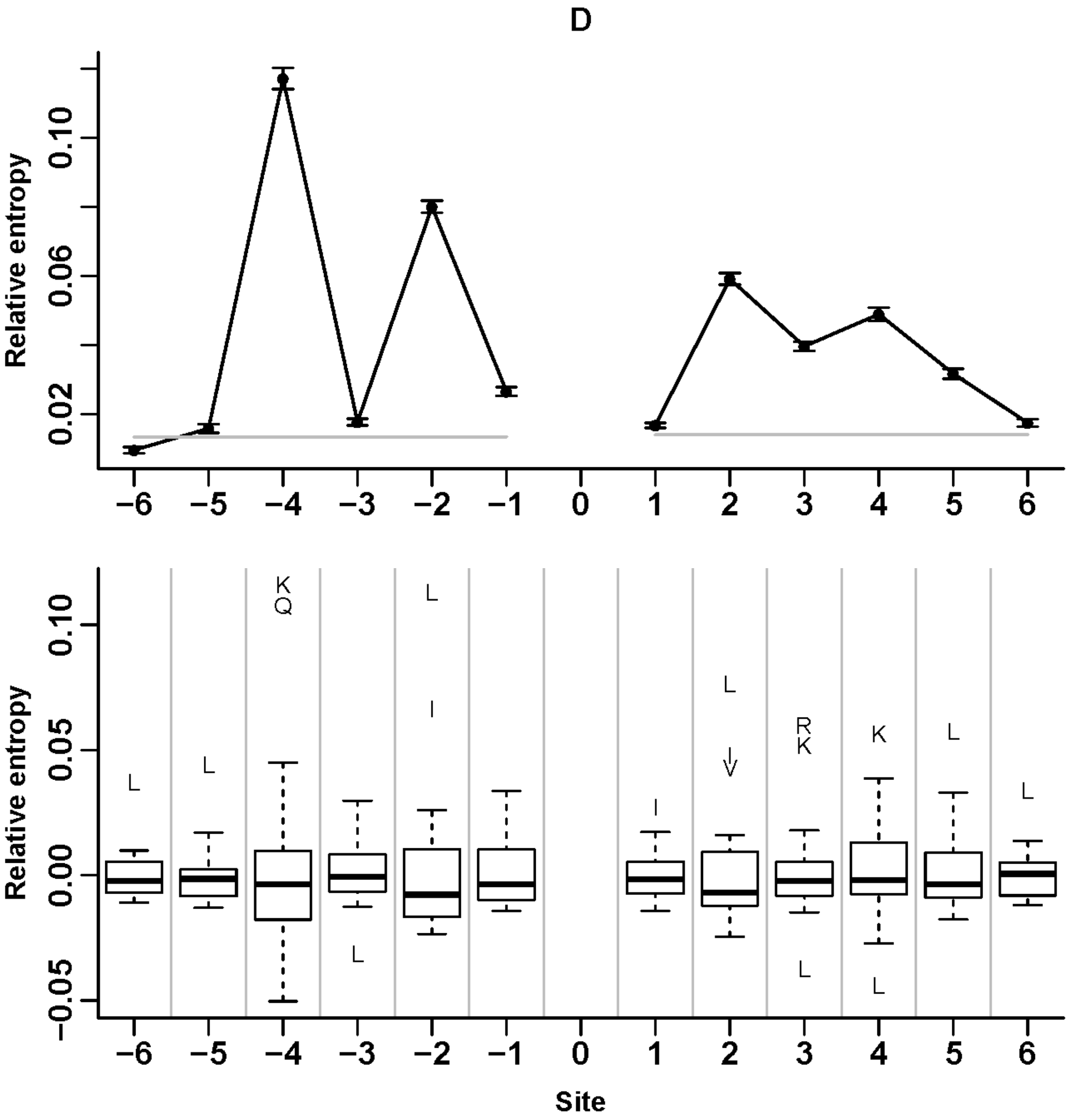
D
Relative entropy
0.02
0.06
0.10
−6
−5
−4
−3
−2
−1
0
1
2
3
4
5
6
Relative entropy
−0.05
0.00
0.05
0.10
L
L
K
Q
L
L
I
I
L
I
V
R
K
L
K
L
L
L
Site

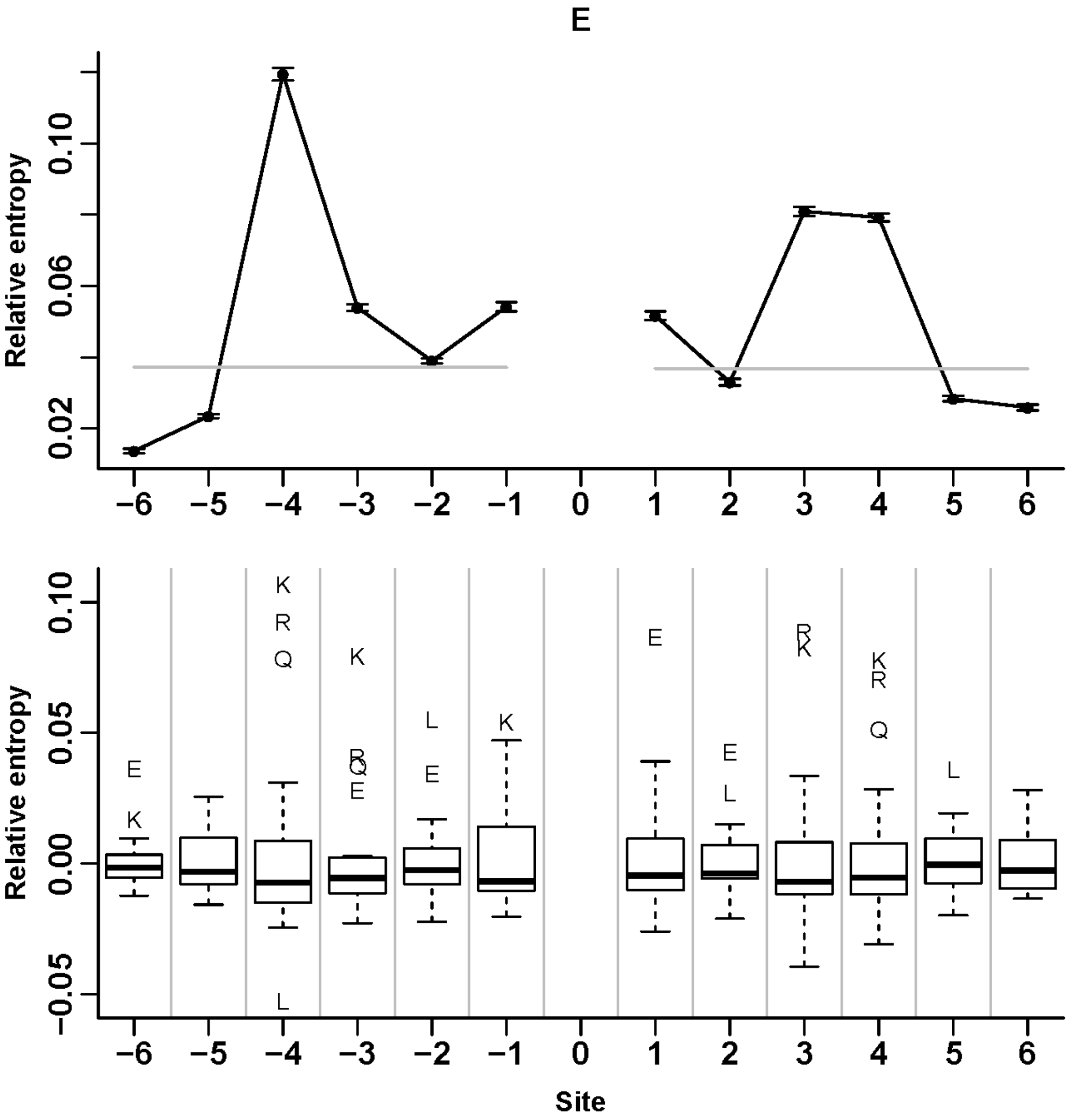
E
Relative entropy
0.02
0.06
0.10
−6
−5
−4
−3
−2
−1
0
1
2
3
4
5
6
Relative entropy
−0.05
0.00
0.05
0.10
E
K
K
R
Q
L
K
R
Q
E
L
E
K
E
E
L
R
K
K
R
Q
L
Site

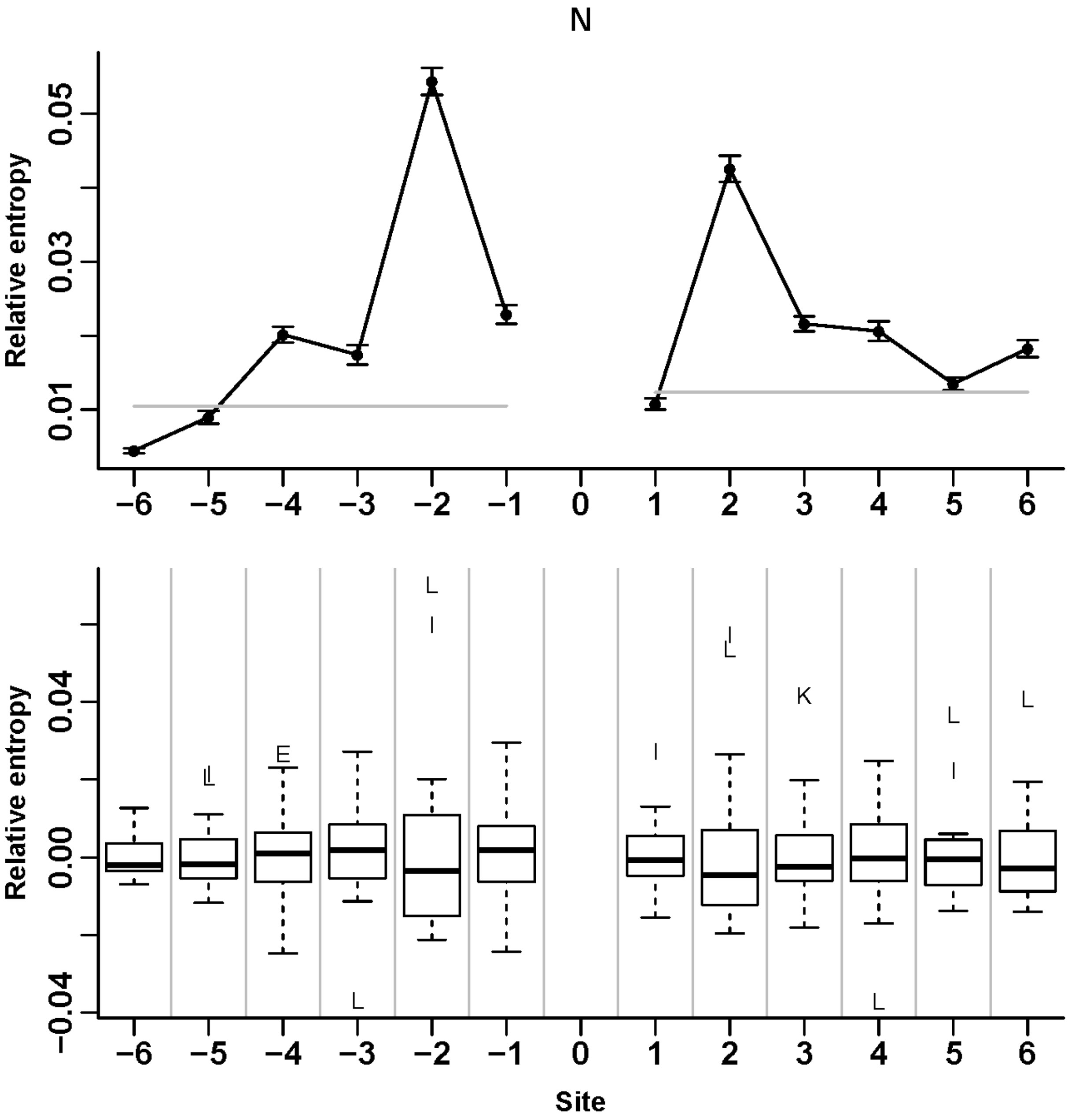
N
Relative entropy
0.01
0.03
0.05
−6
−5
−4
−3
−2
−1
0
1
2
3
4
5
6
Relative entropy
−0.04
0.00
0.04
L
I
E
L
L
I
I
L
I
K
L
L
I
L
L
Site

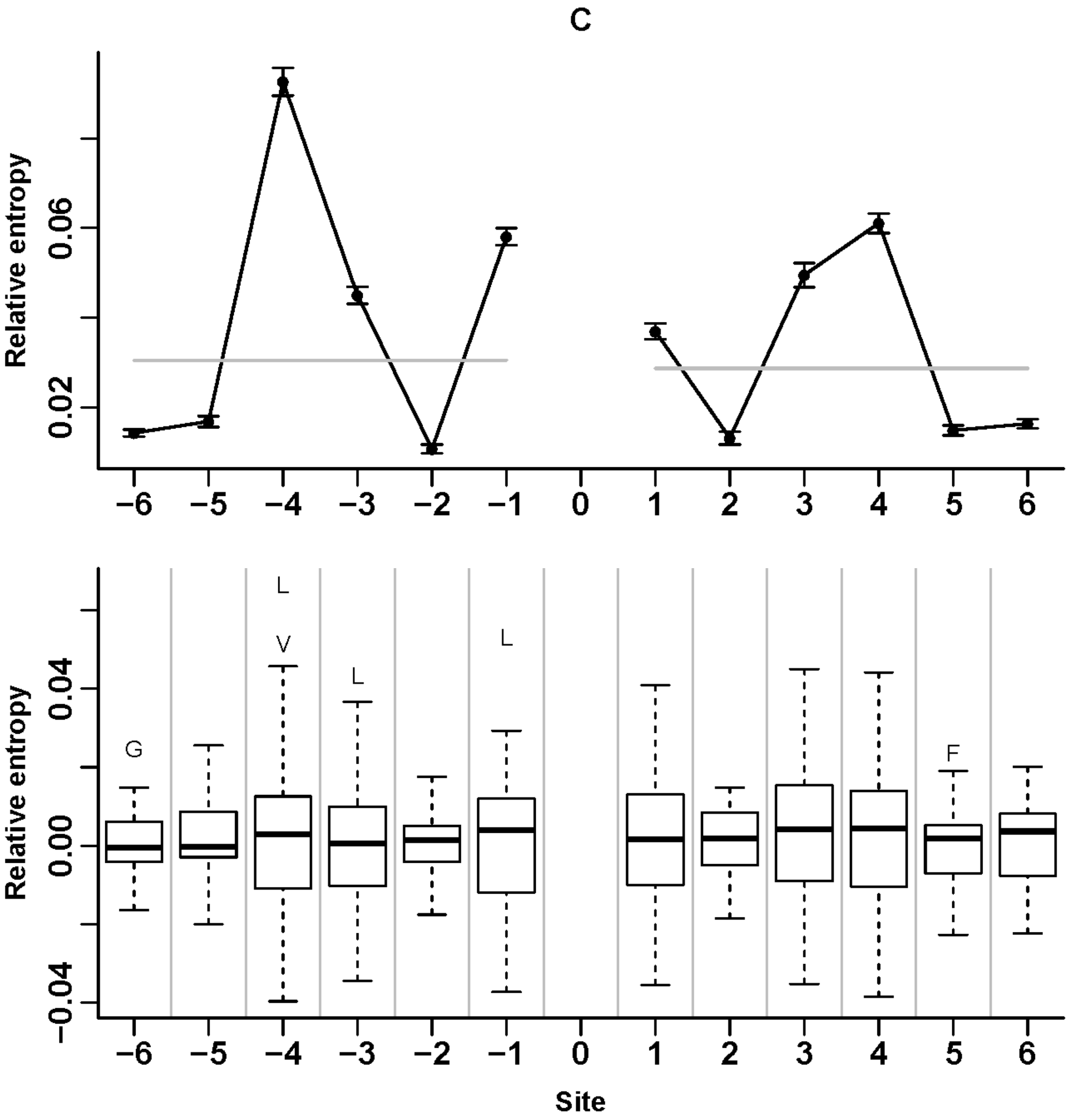
C
Relative entropy
0.02
0.06
−6 −5 −4 −3 −2 −1 0 1 2 3 4 5 6
Relative entropy
−0.04
0.00
0.04
G
L
V
L
L
F
−6 −5 −4 −3 −2 −1 0 1 2 3 4 5 6
Site

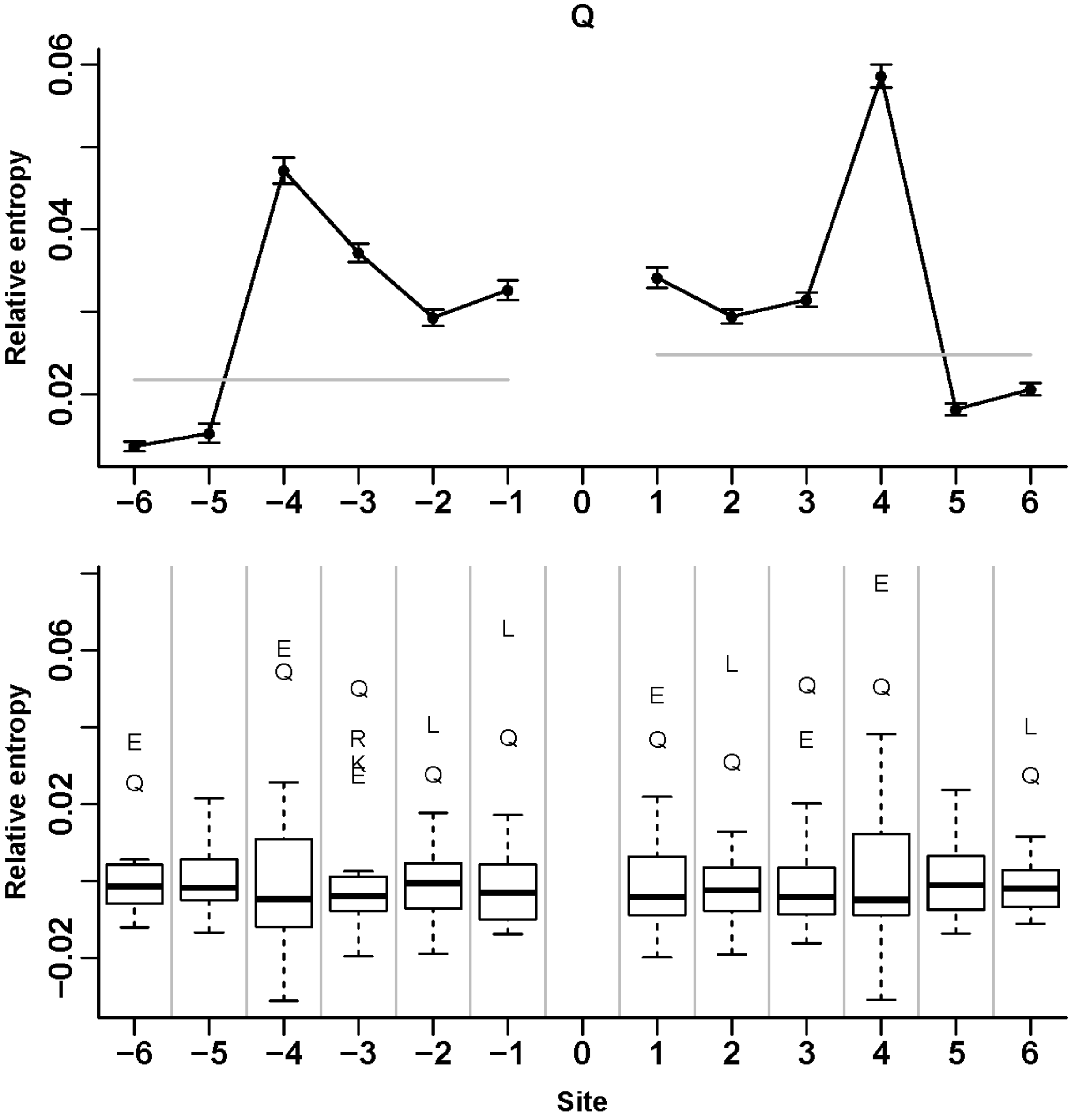
Q
Relative entropy
0.02
0.04
0.06
−6
−5
−4
−3
−2
−1
0
1
2
3
4
5
6
Relative entropy
−0.02
0.02
0.06
E
Q
E
Q
Q
R
K
E
L
Q
L
Q
E
Q
L
Q
Q
E
E
Q
L
Q
Site

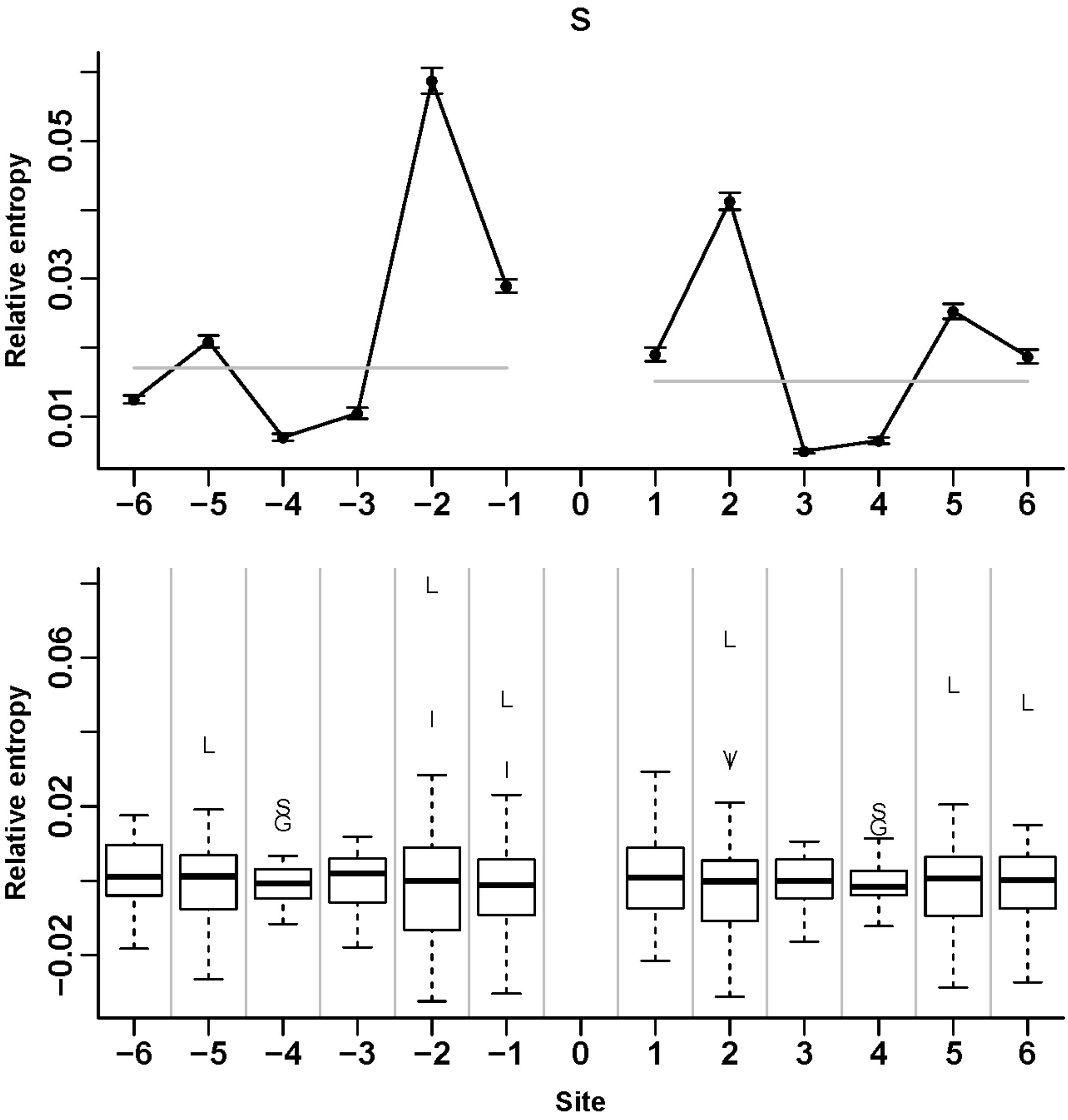
S
Relative entropy
0.01
0.03
0.05
−6
−5
−4
−3
−2
−1
0
1
2
3
4
5
6
Relative entropy
−0.02
0.02
0.06
L
I
Site

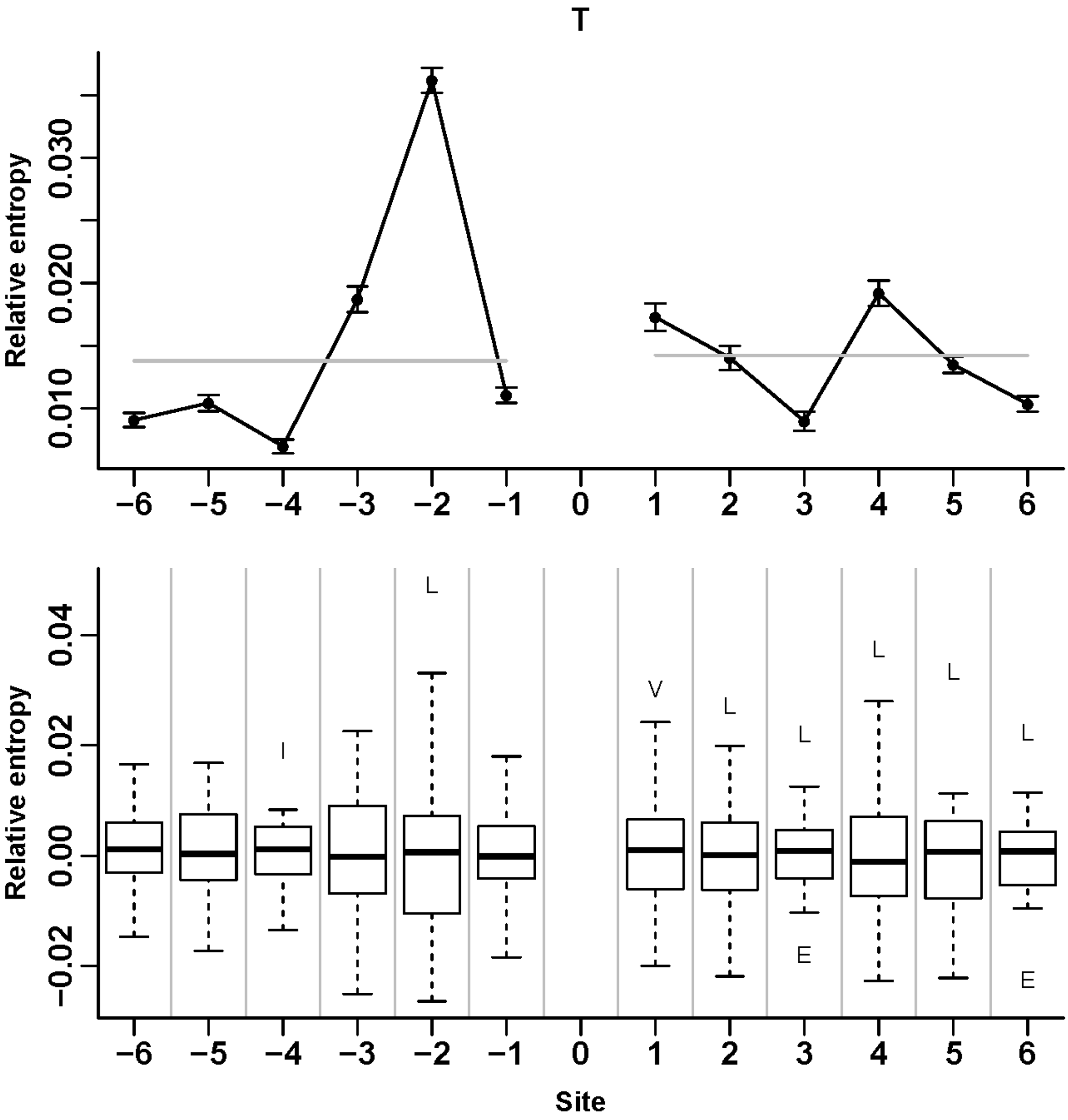
T
Relative entropy
0.010
0.020
0.030
−6
−5
−4
−3
−2
−1
0
1
2
3
4
5
6
L
I
V
L
L
L
L
L
E
E
Relative entropy
−0.02
0.00
0.02
0.04
Site

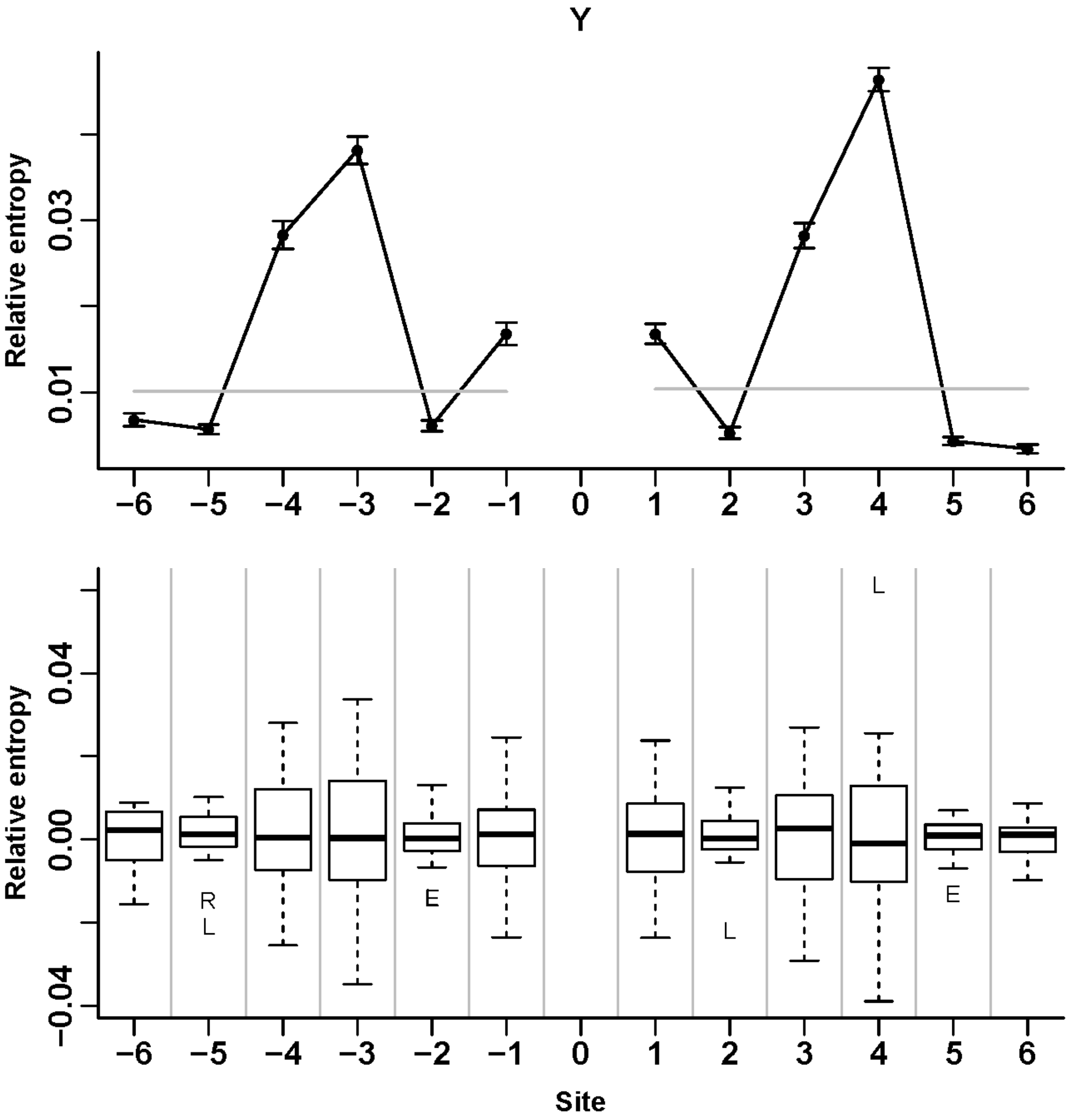
Y
Relative entropy
0.01
0.03
−6
−5
−4
−3
−2
−1
0
1
2
3
4
5
6
Relative entropy
−0.04
0.00
0.04
R
L
E
L
L
E
Site

Figure S3. Neighbor preference patterns of the 20 amino acids in β-strand.

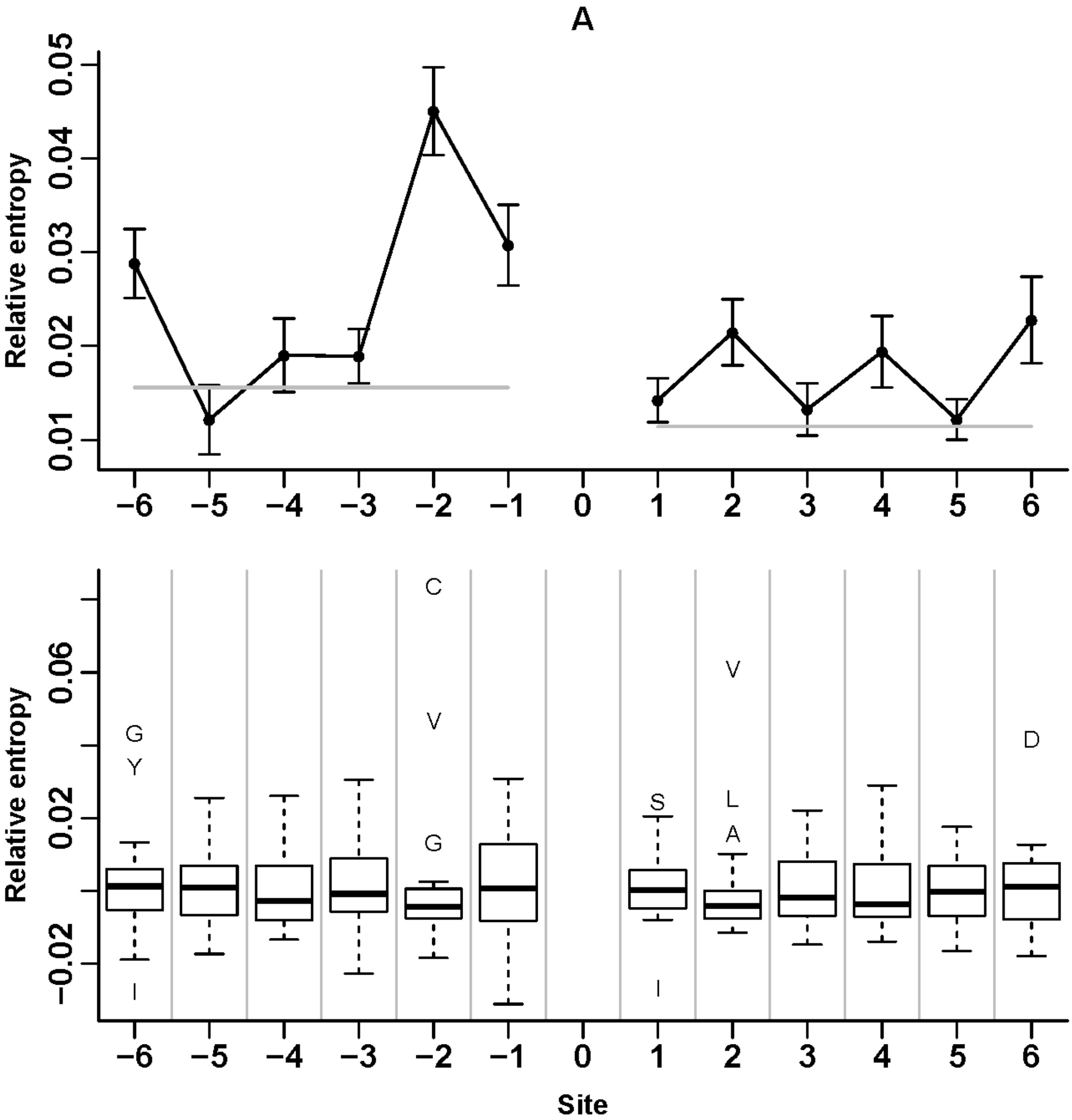

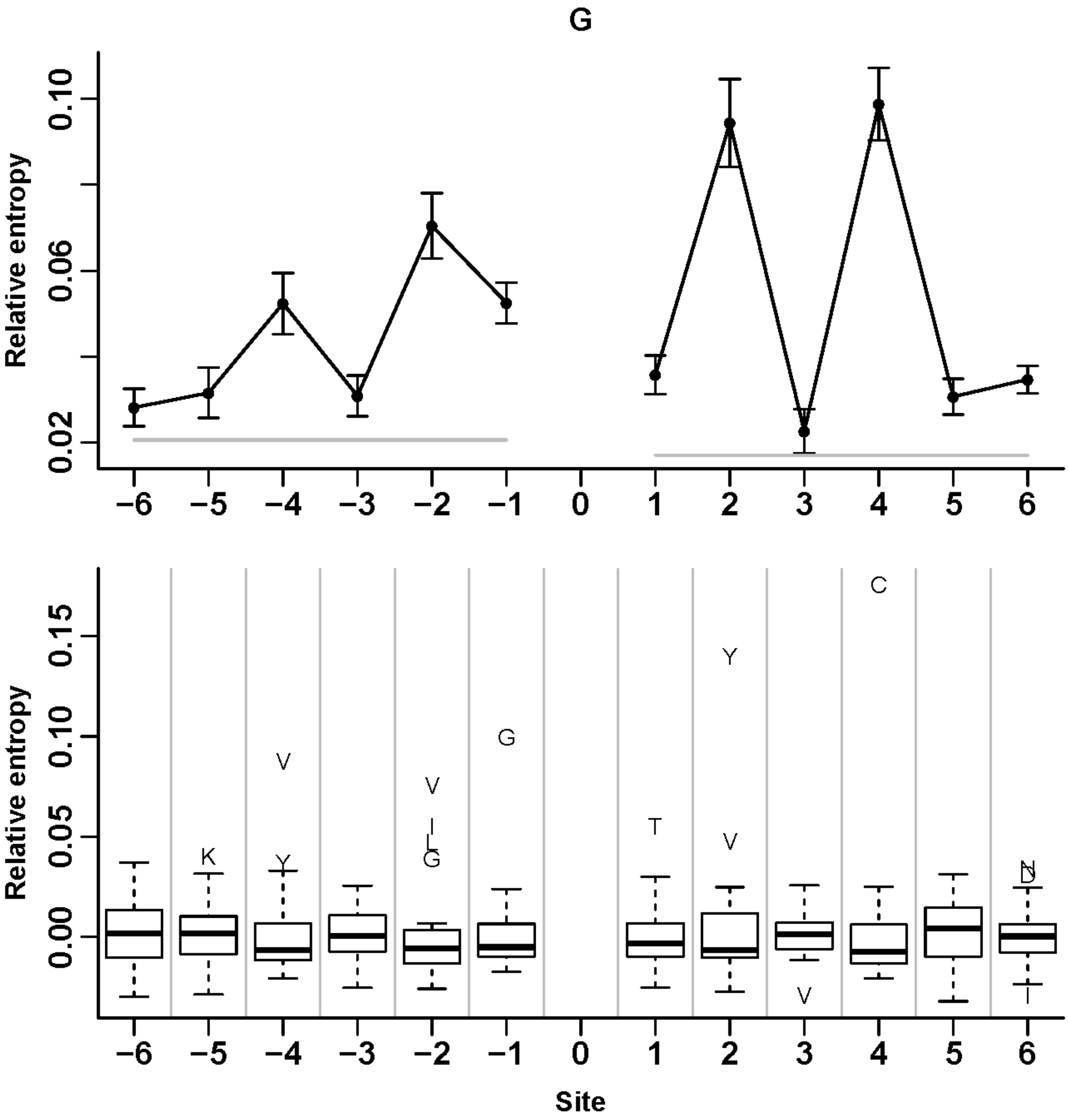
G
Relative entropy
0.02
0.06
0.10
−6
−5
−4
−3
−2
−1
0
1
2
3
4
5
6
Relative entropy
0.00
0.05
0.10
0.15
K
V
Y
V
I
L
G
G
T
Y
V
V
C
N
D
I
Site

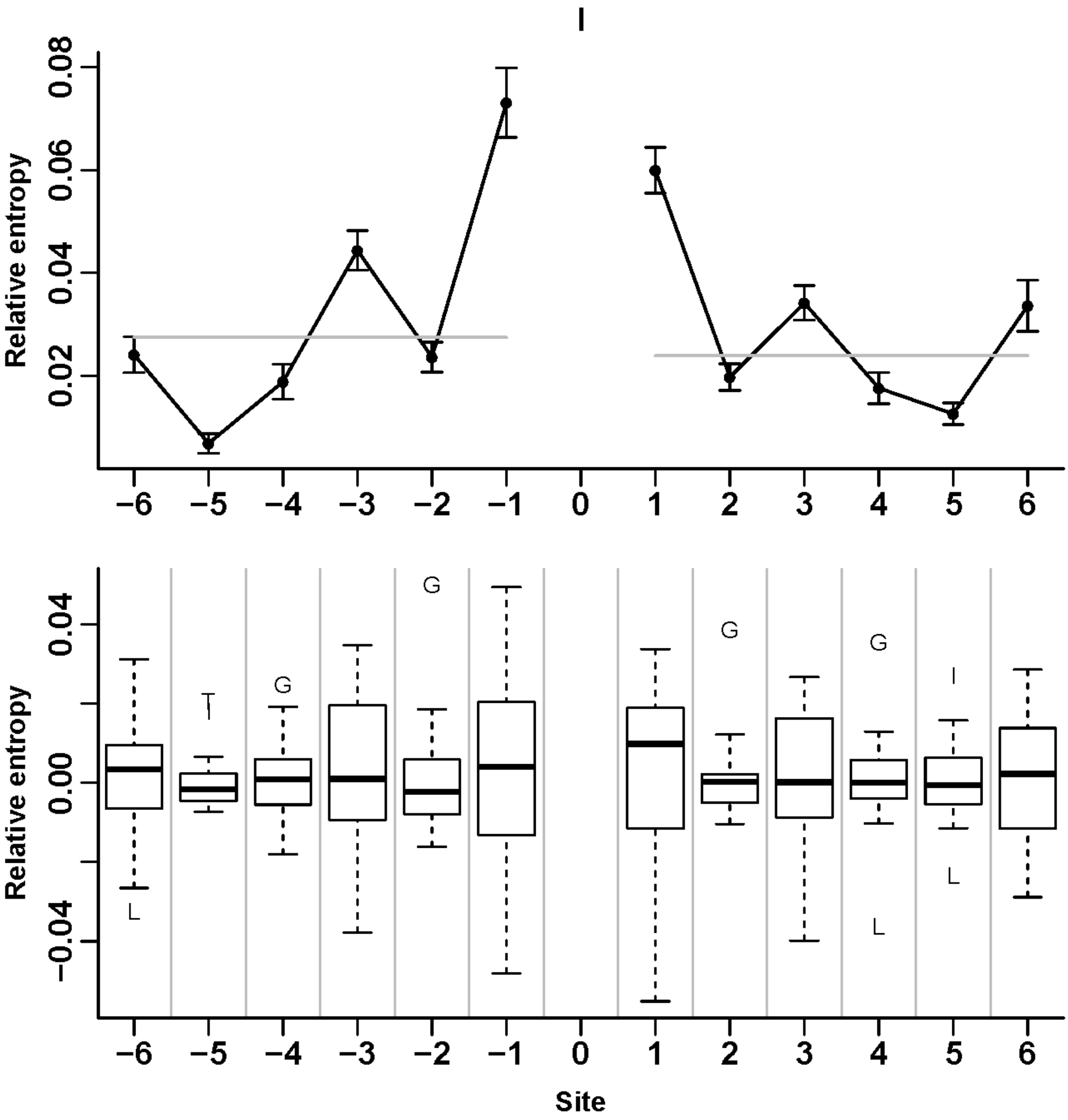
I
Relative entropy
0.08
0.06
0.04
0.02
−6 −5 −4 −3 −2 −1 0 1 2 3 4 5 6
Relative entropy
0.04
0.00
−0.04
G
G
G
T
G
I
L
L
L
−6 −5 −4 −3 −2 −1 0 1 2 3 4 5 6
Site

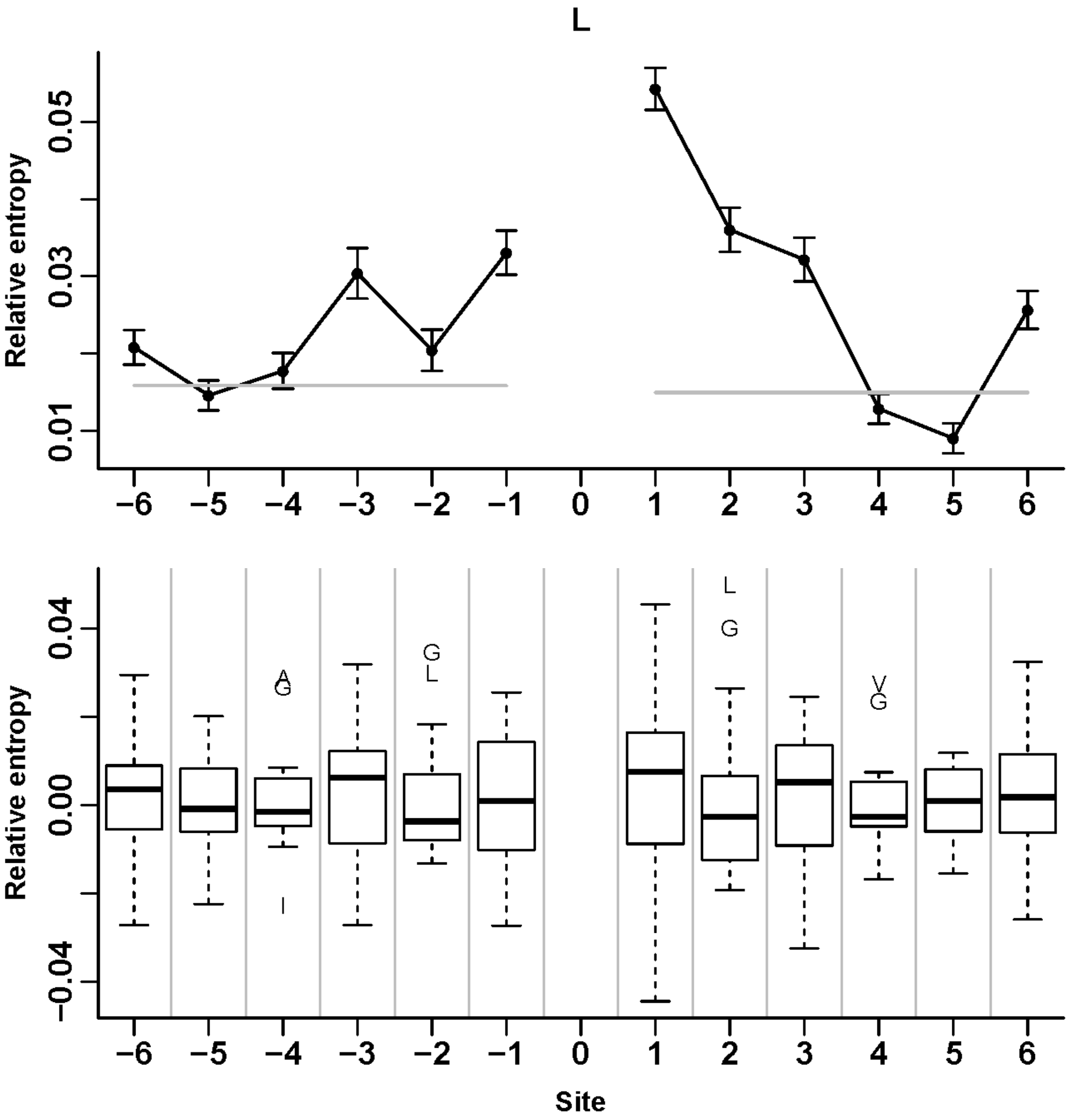
L
Relative entropy
0.01
0.03
0.05
−6
−5
−4
−3
−2
−1
0
1
2
3
4
5
6
Relative entropy
−0.04
0.00
0.04
A
G
I
G
L
L
G
V
G
Site

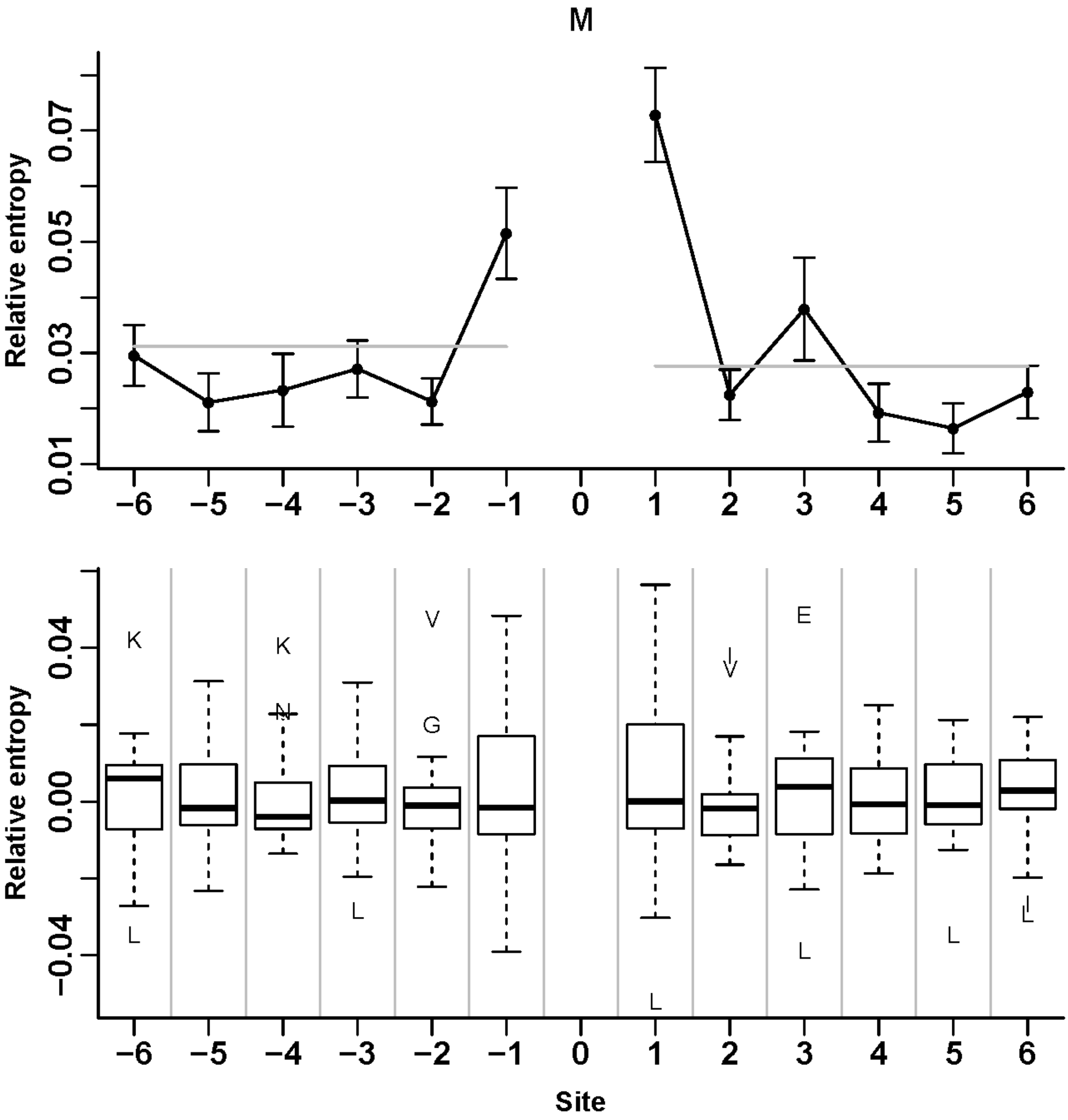
M
Relative entropy
0.01
0.03
0.05
0.07
−6
−5
−4
−3
−2
−1
0
1
2
3
4
5
6
Relative entropy
−0.04
0.00
0.04
K
L
K
N
L
V
G
L
E
L
L
I
V
L
I
L
Site

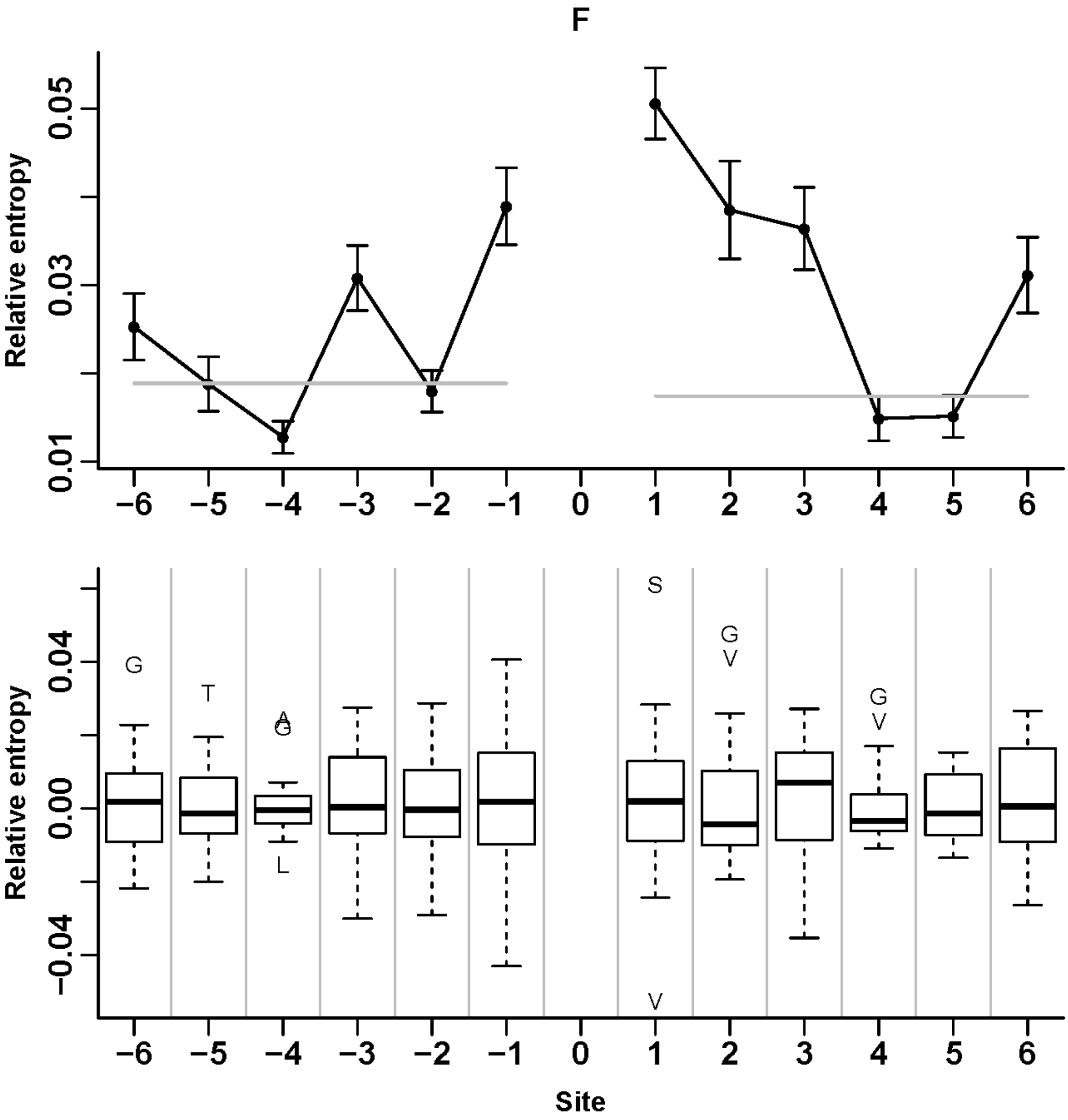
F
Relative entropy
0.01
0.03
0.05
−6
−5
−4
−3
−2
−1
0
1
2
3
4
5
6
Relative entropy
−0.04
0.00
0.04
G
T
A
G
L
S
G
V
G
V
V
Site

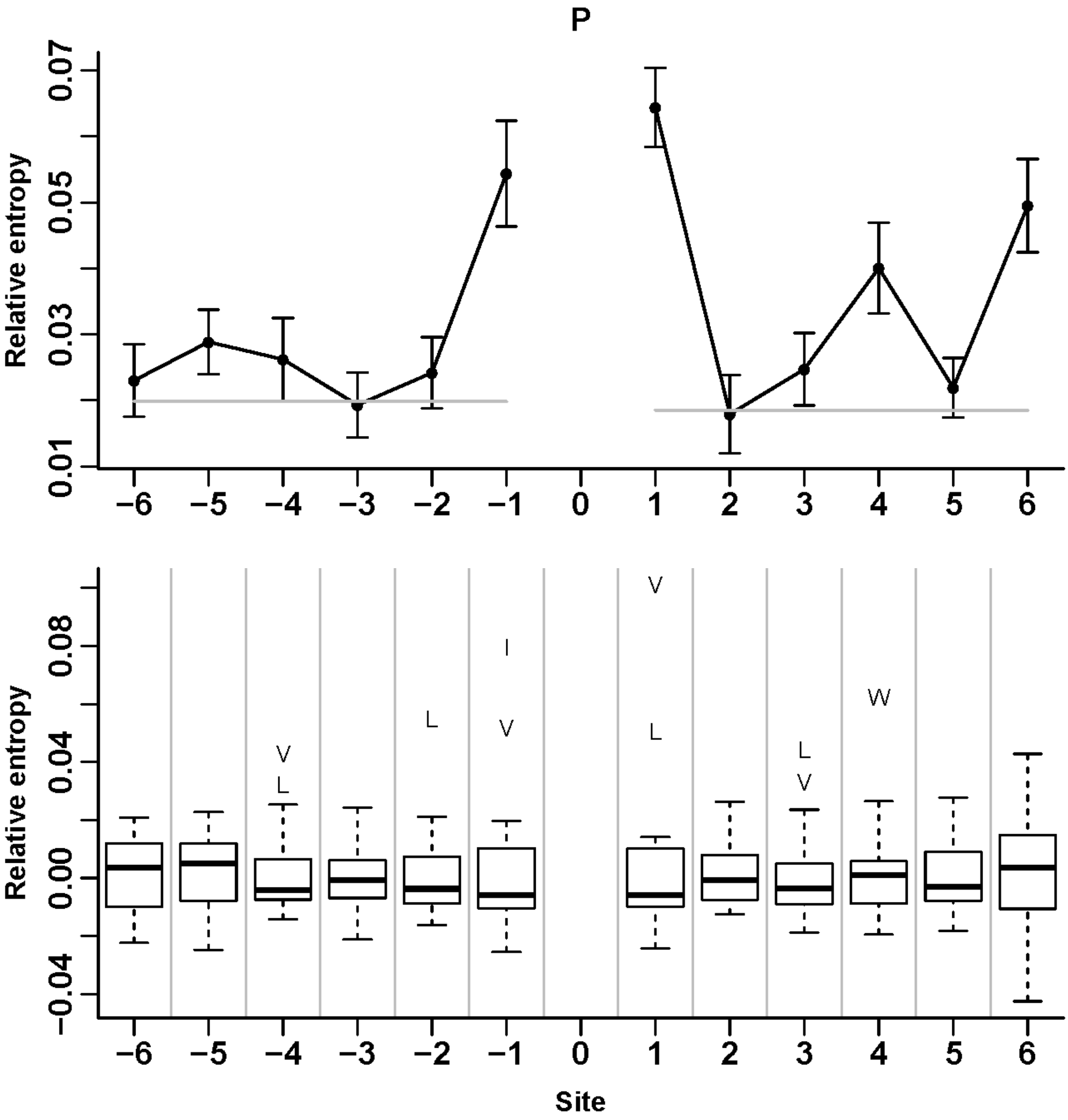
P
Relative entropy
0.01
0.03
0.05
0.07
−6
−5
−4
−3
−2
−1
0
1
2
3
4
5
6
Relative entropy
−0.04
0.00
0.04
0.08
V
L
L
I
V
V
L
L
V
W
Site

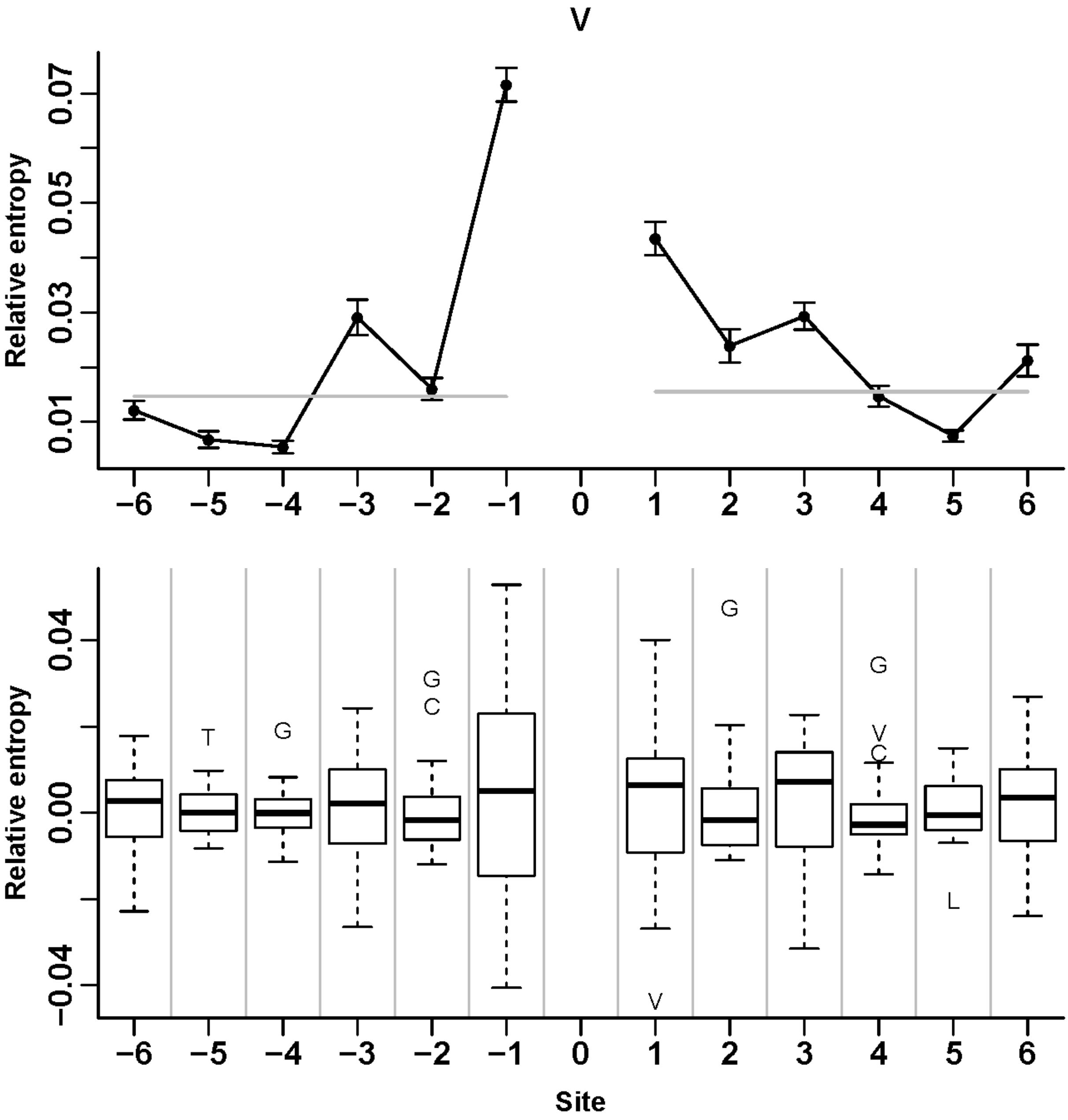
V
Relative entropy
0.01
0.03
0.05
0.07
−6
−5
−4
−3
−2
−1
0
1
2
3
4
5
6
Relative entropy
−0.04
0.00
0.04
T
G
G
C
G
G
V
C
L
V
Site

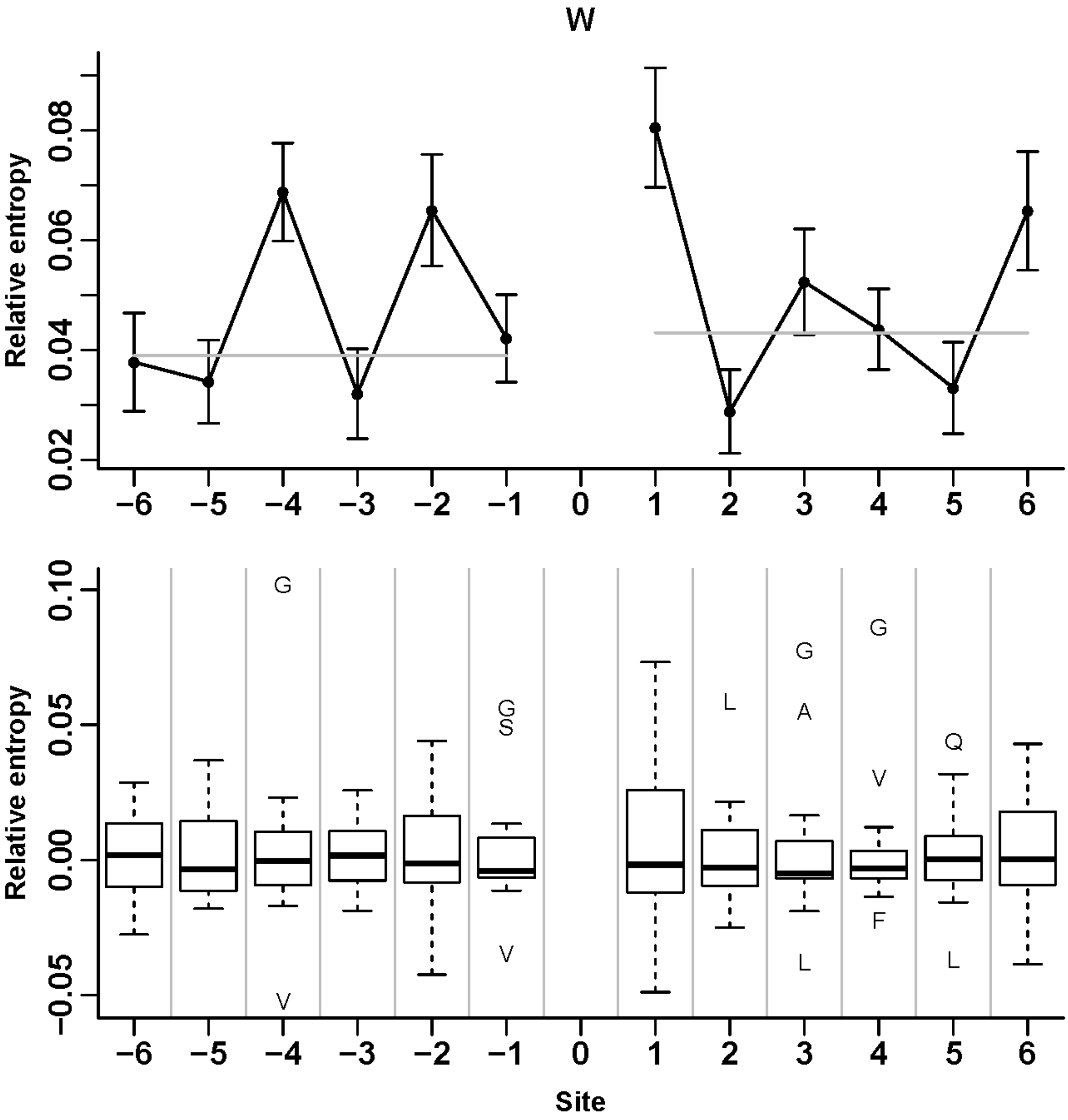
W
Relative entropy
0.02
0.04
0.06
0.08
−6
−5
−4
−3
−2
−1
0
1
2
3
4
5
6
Relative entropy
−0.05
0.00
0.05
0.10
G
V
G
S
V
L
G
A
L
G
V
F
Q
L
Site

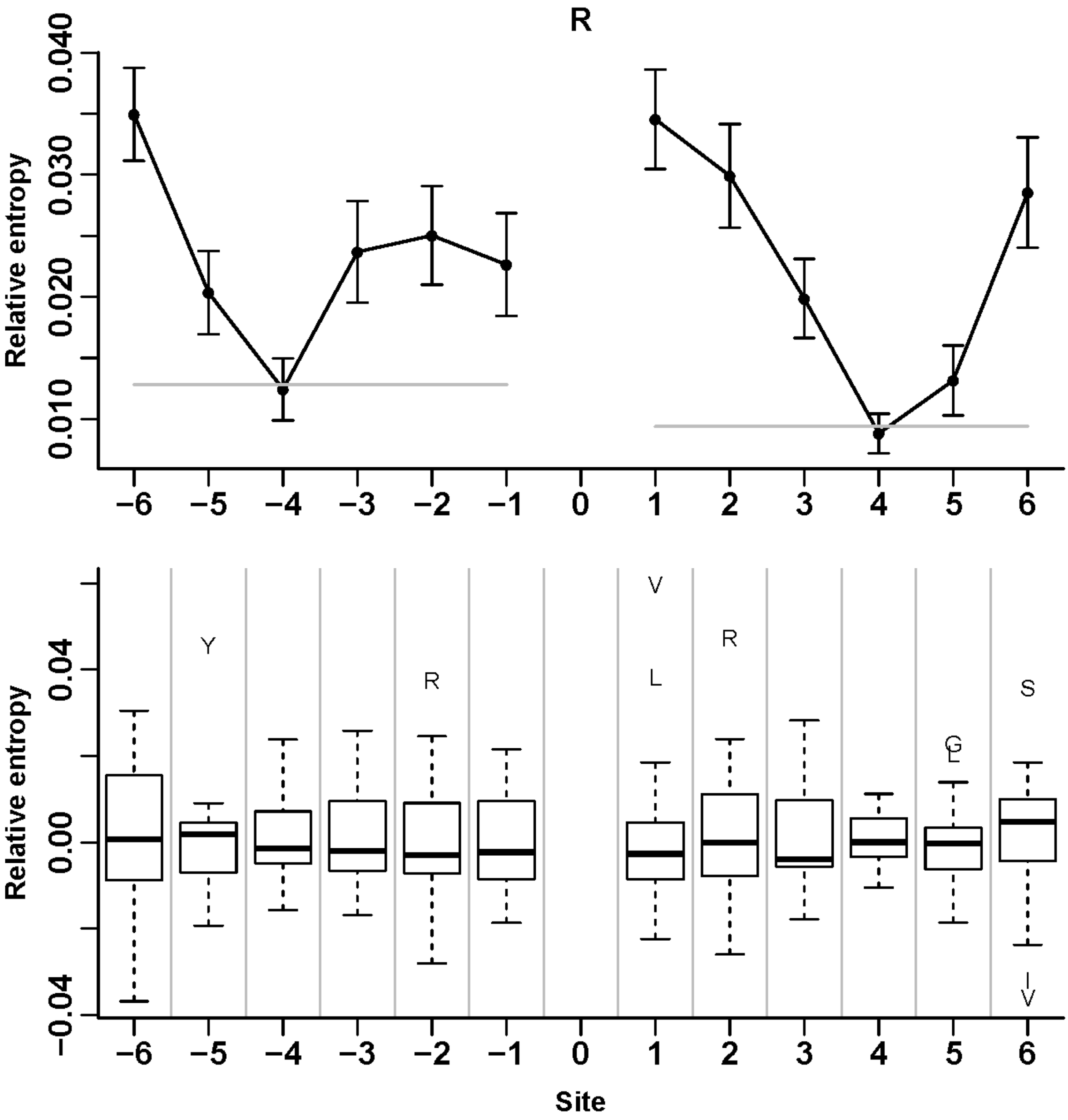
R
Relative entropy
0.010
0.020
0.030
0.040
−6
−5
−4
−3
−2
−1
0
1
2
3
4
5
6
Relative entropy
−0.04
0.00
0.04
Y
R
V
L
R
G
L
S
I
V
Site

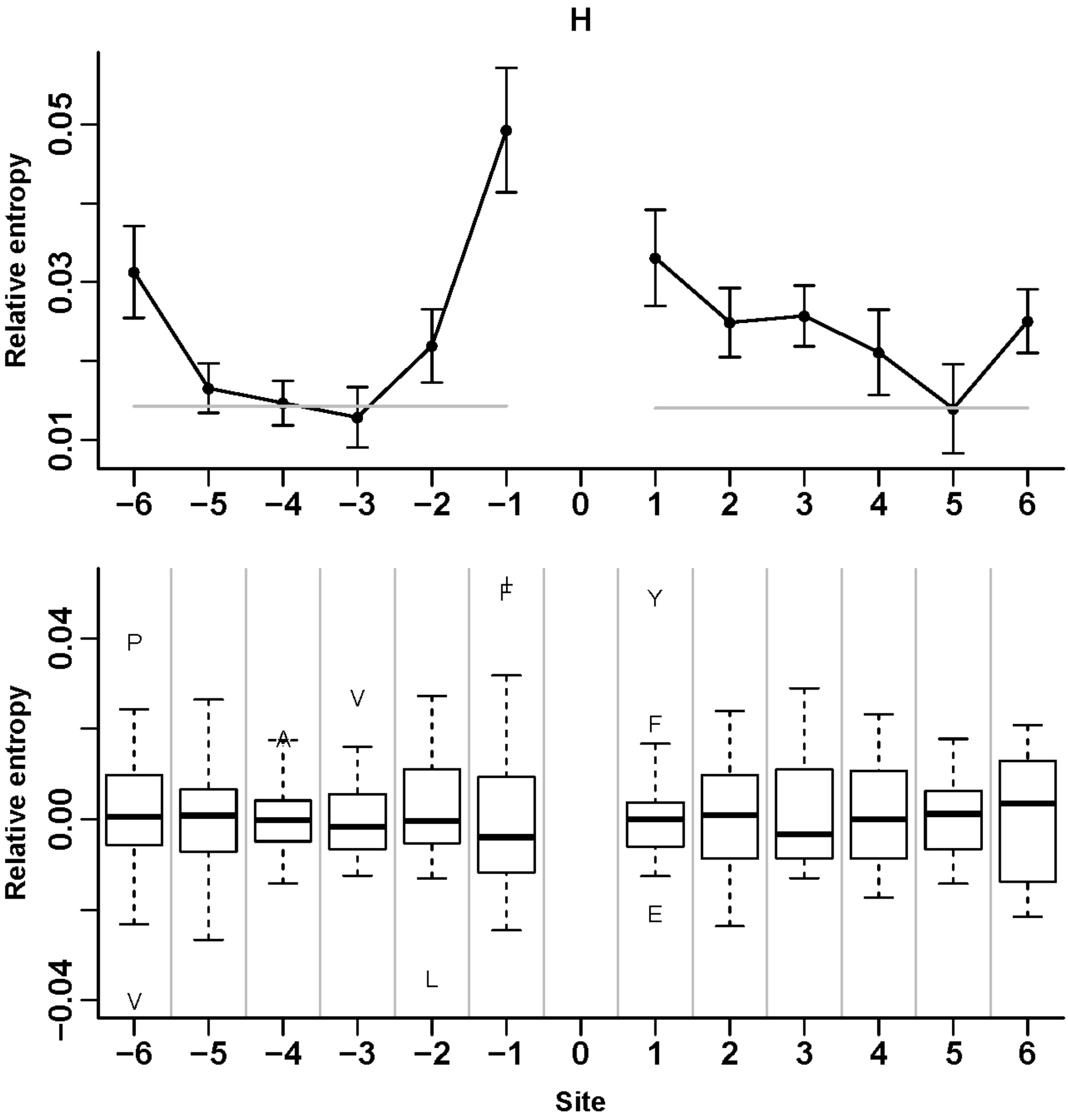
H
Relative entropy
0.01
0.03
0.05
−6
−5
−4
−3
−2
−1
0
1
2
3
4
5
6
Relative entropy
−0.04
0.00
0.04
P
V
A
V
L
F
Y
F
E
Site

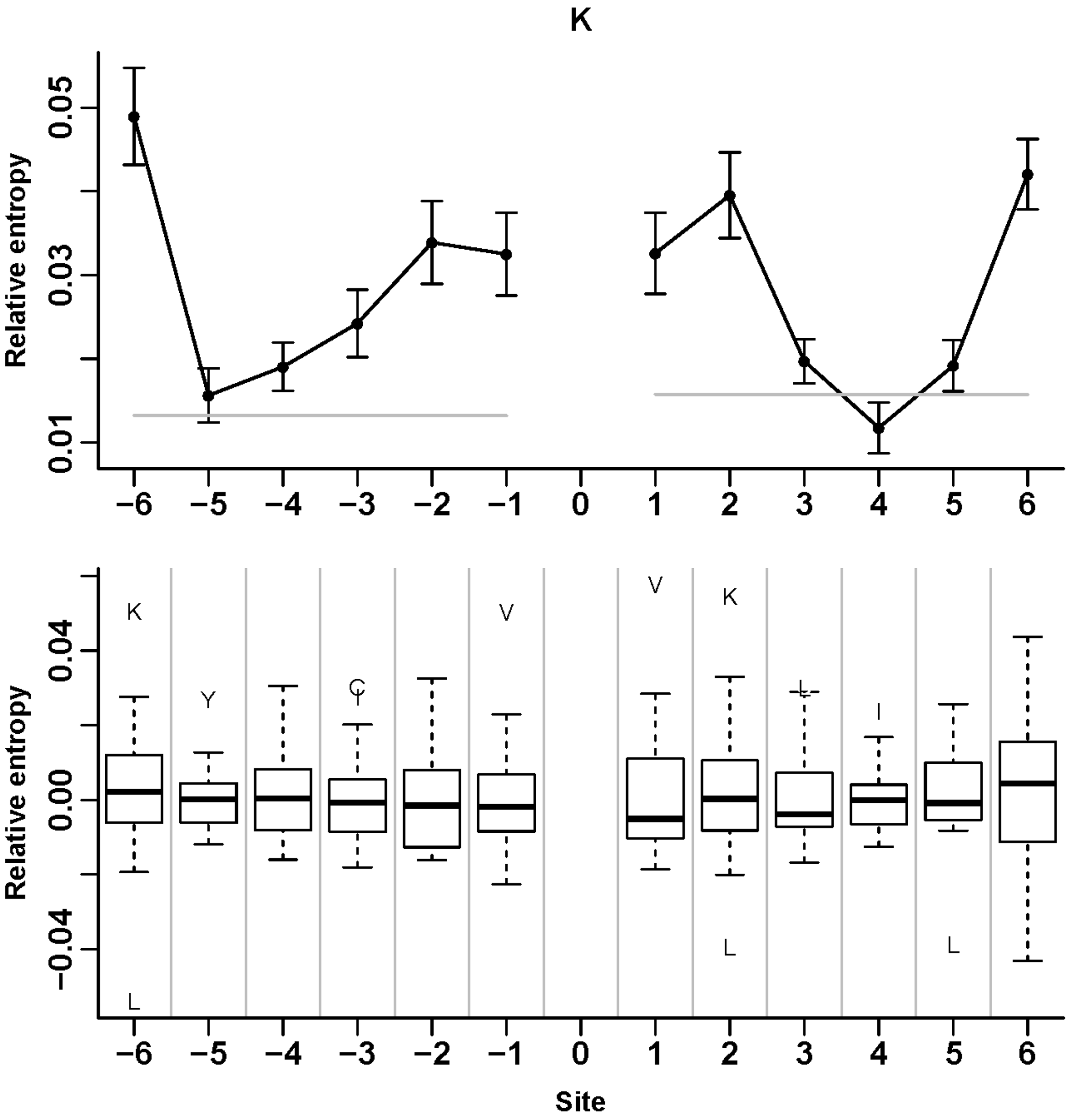
K
Relative entropy
0.01
0.03
0.05
−6
−5
−4
−3
−2
−1
0
1
2
3
4
5
6
Relative entropy
−0.04
0.00
0.04
K
Y
C
V
V
K
L
I
L
L
L
Site

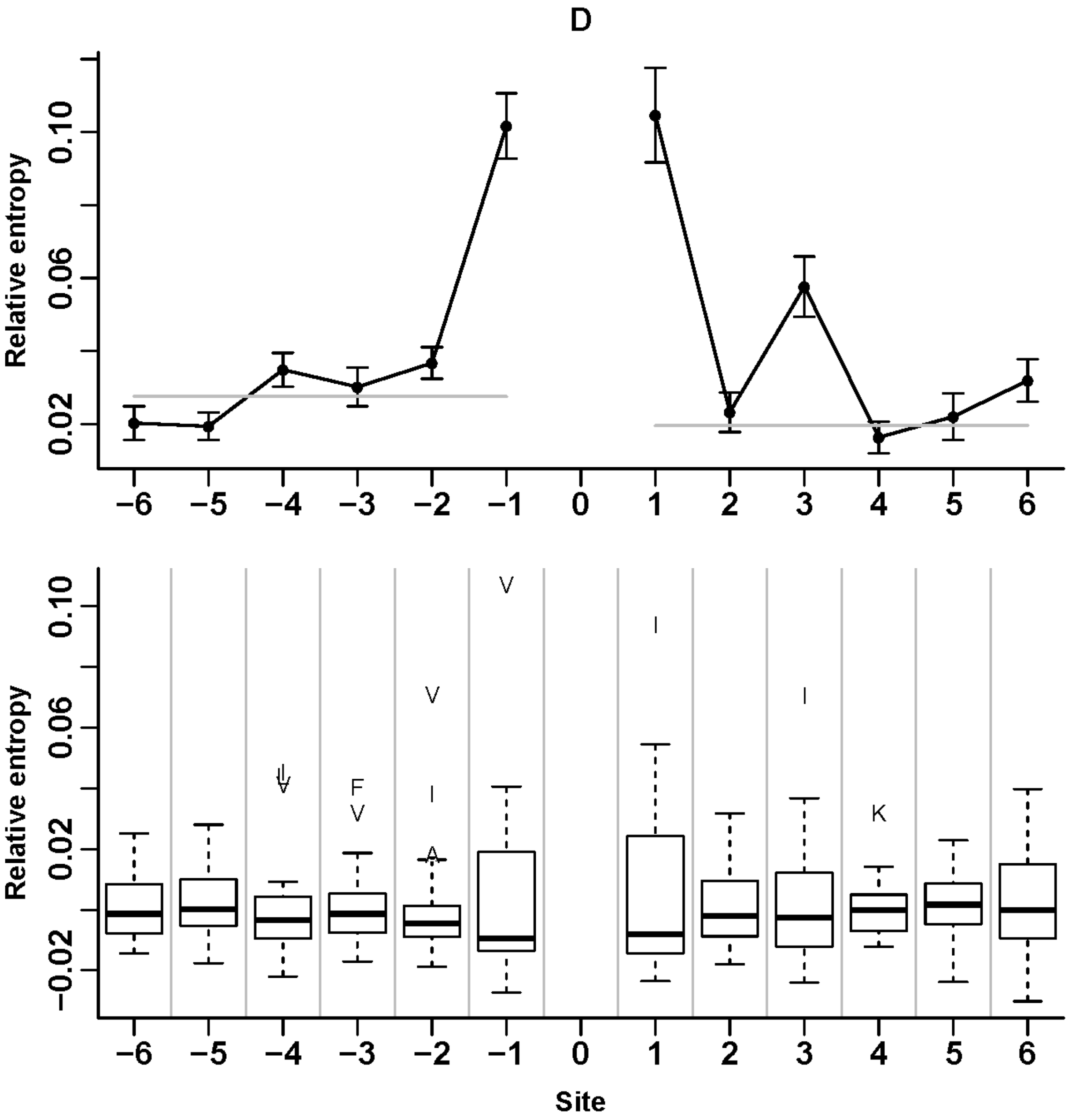
D
Relative entropy
0.02
0.06
0.10
−6
−5
−4
−3
−2
−1
0
1
2
3
4
5
6
Relative entropy
−0.02
0.02
0.06
0.10
V
I
V
I
F
V
I
A
K
Site

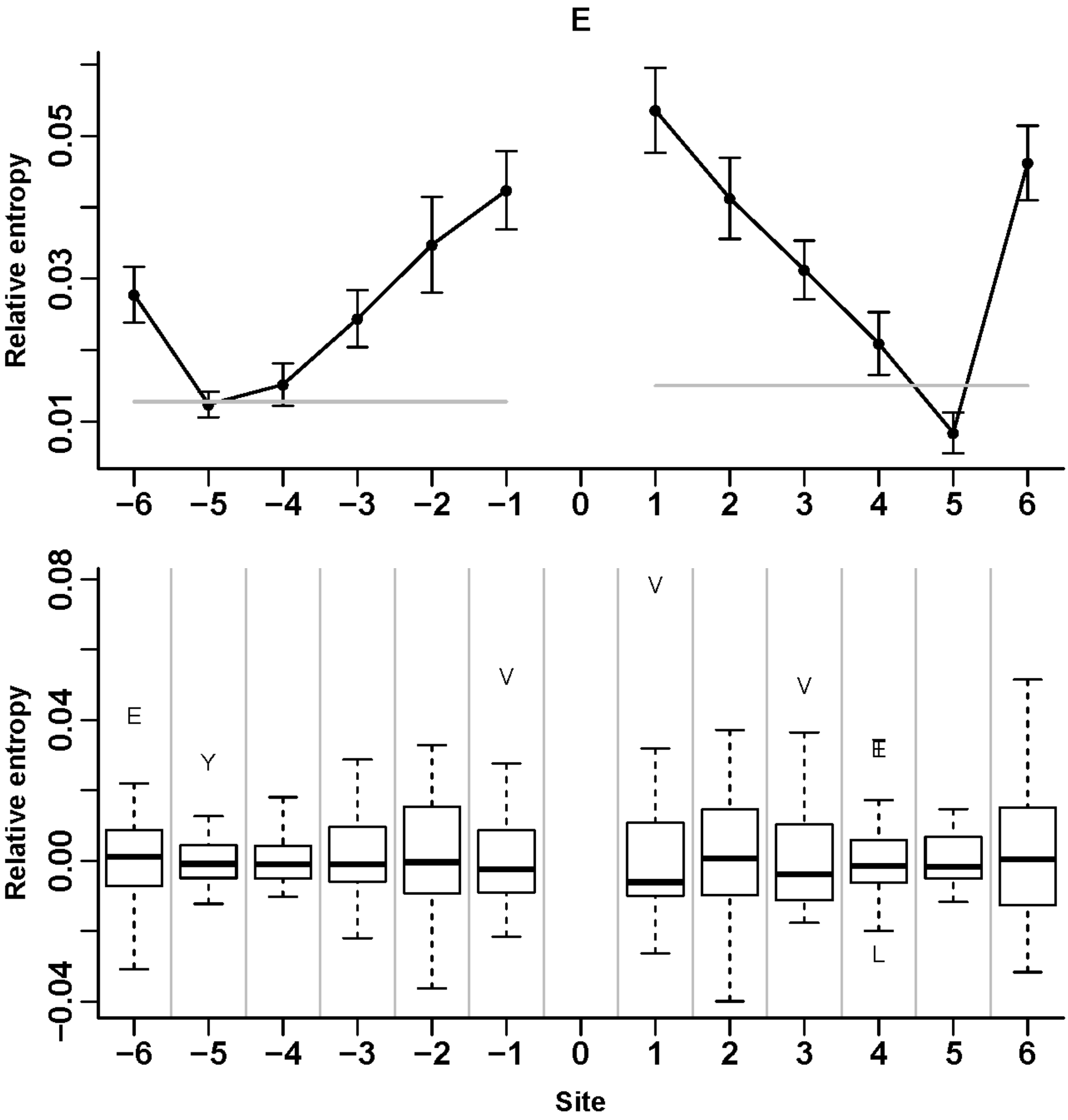
E
Relative entropy
0.01
0.03
0.05
−6
−5
−4
−3
−2
−1
0
1
2
3
4
5
6
Relative entropy
−0.04
0.00
0.04
0.08
E
Y
V
V
V
E
L
Site

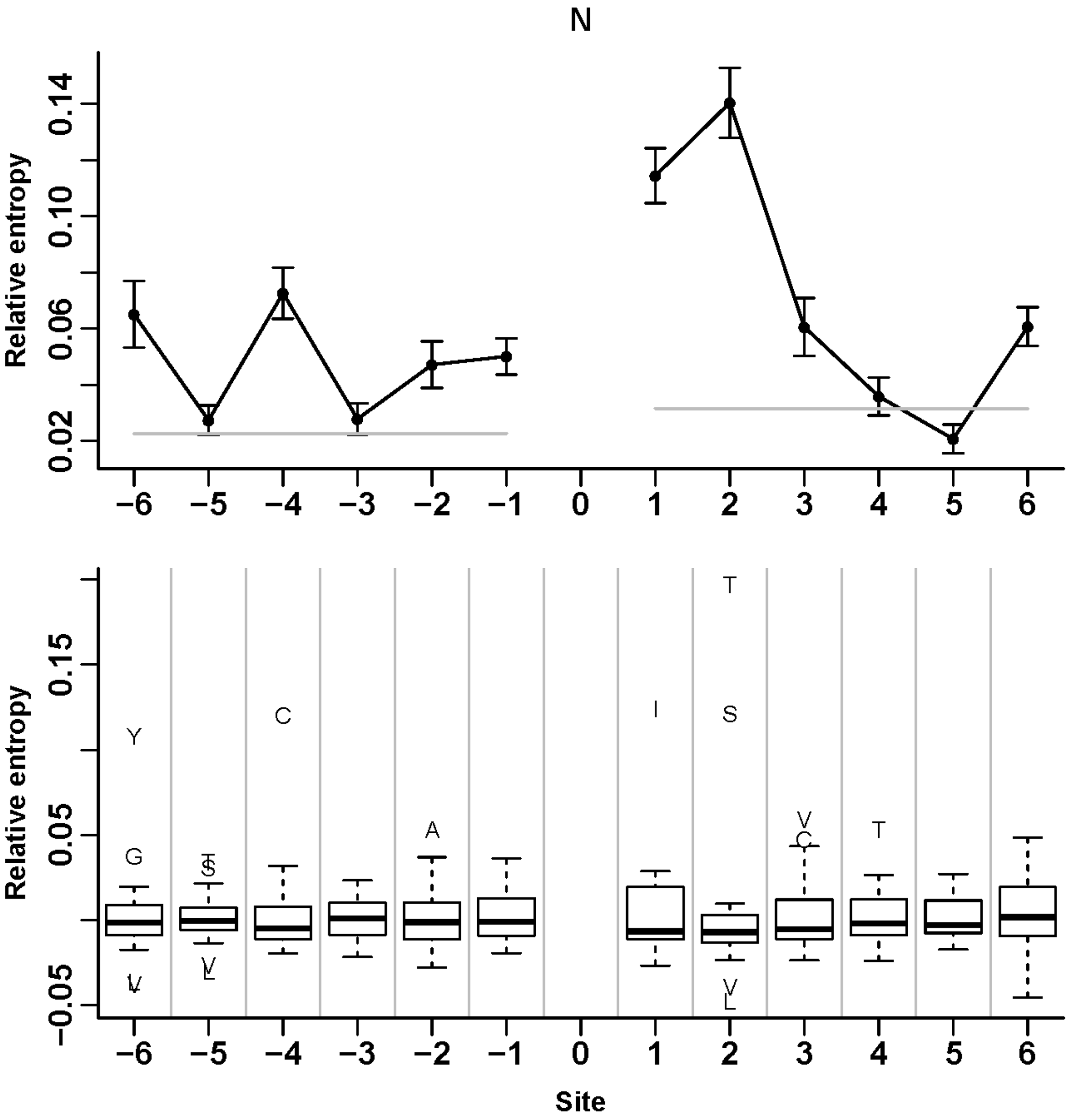
N
Relative entropy
0.02
0.06
0.10
0.14
−6
−5
−4
−3
−2
−1
0
1
2
3
4
5
6
Relative entropy
−0.05
0.05
0.15
Y
G
C
A
I
T
S
V
C
T
V
L
Site

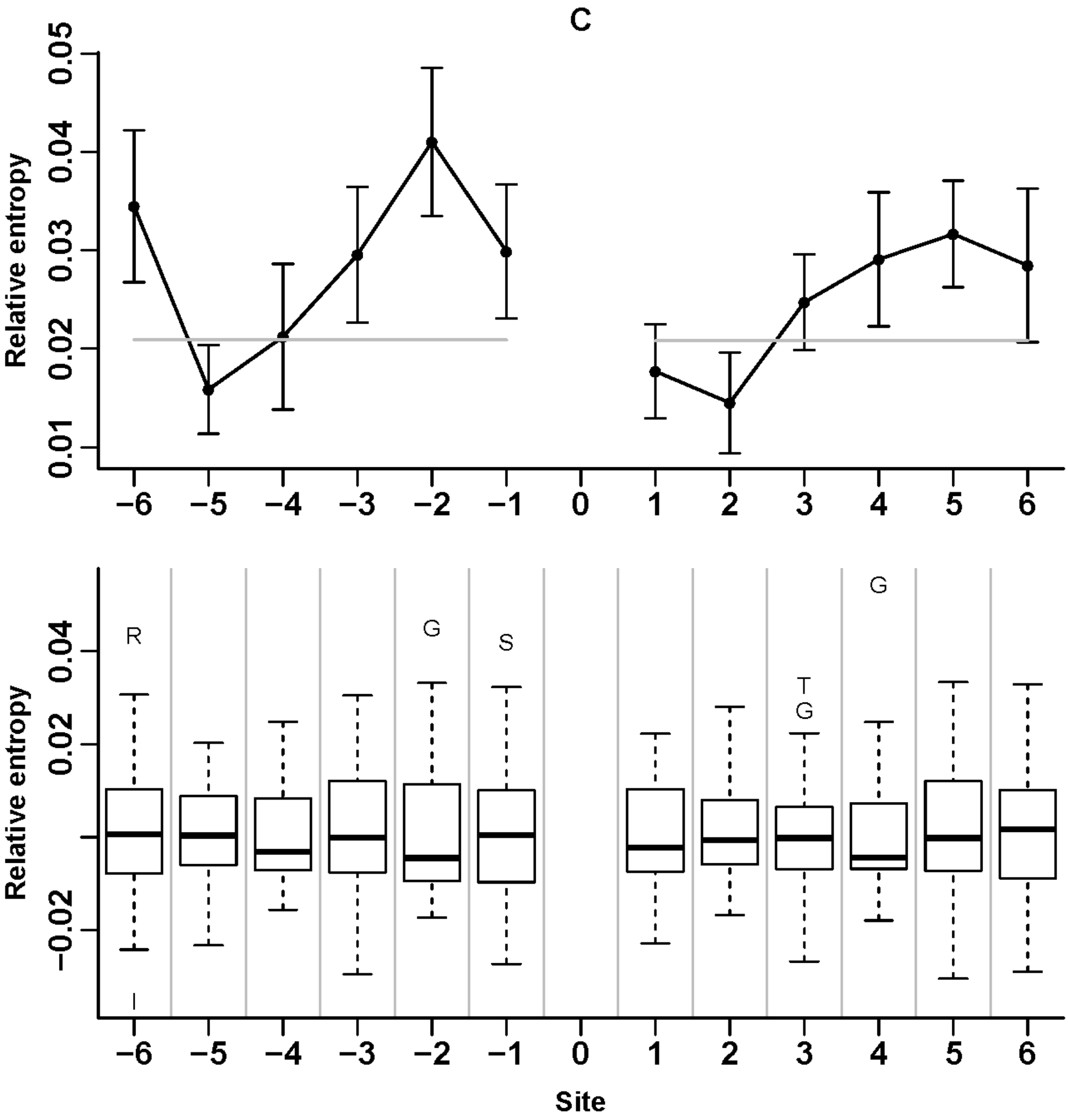
C
Relative entropy
0.01
0.02
0.03
0.04
0.05
−6
−5
−4
−3
−2
−1
0
1
2
3
4
5
6
Relative entropy
−0.02
0.02
0.04
R
I
G
S
T
G
G
Site

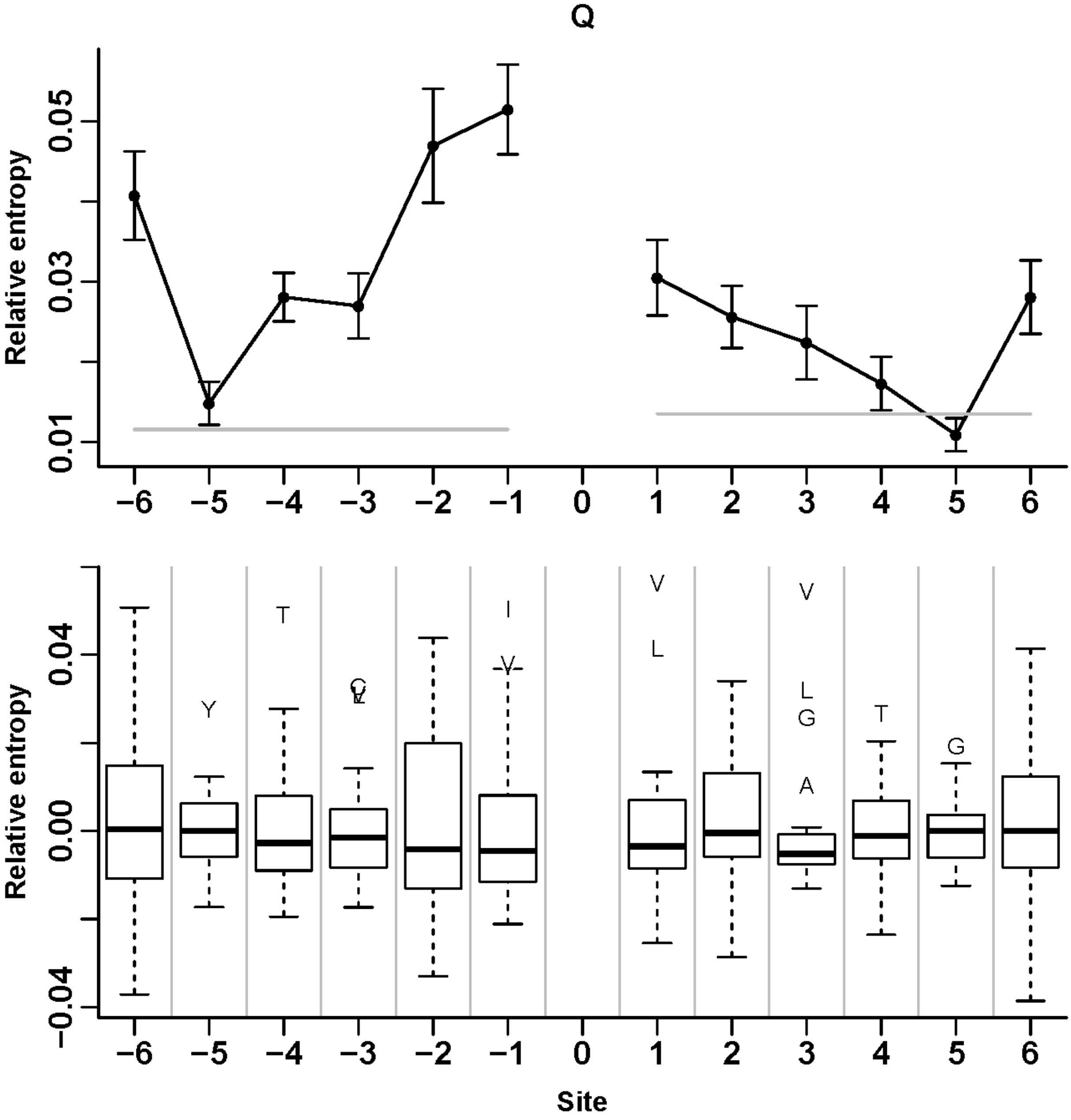
Q
Relative entropy
0.01
0.03
0.05
−6
−5
−4
−3
−2
−1
0
1
2
3
4
5
6
Y
T
C
V
I
V
V
L
V
L
G
A
T
G
−0.04
0.00
0.04
Site

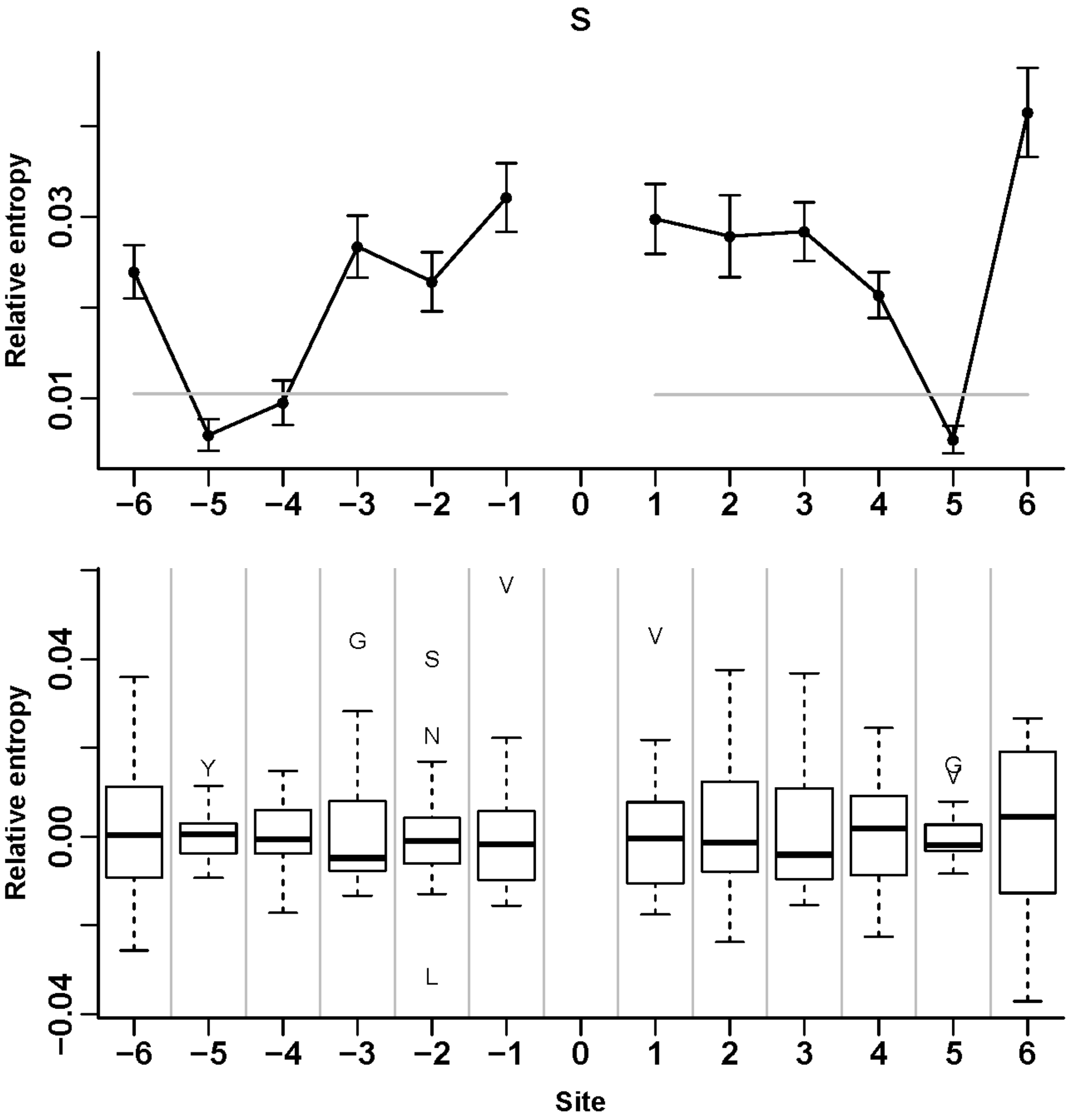
S
Relative entropy
0.01
0.03
−6
−5
−4
−3
−2
−1
0
1
2
3
4
5
6
Relative entropy
−0.04
0.00
0.04
Y
G
S
N
L
V
V
G
V
Site

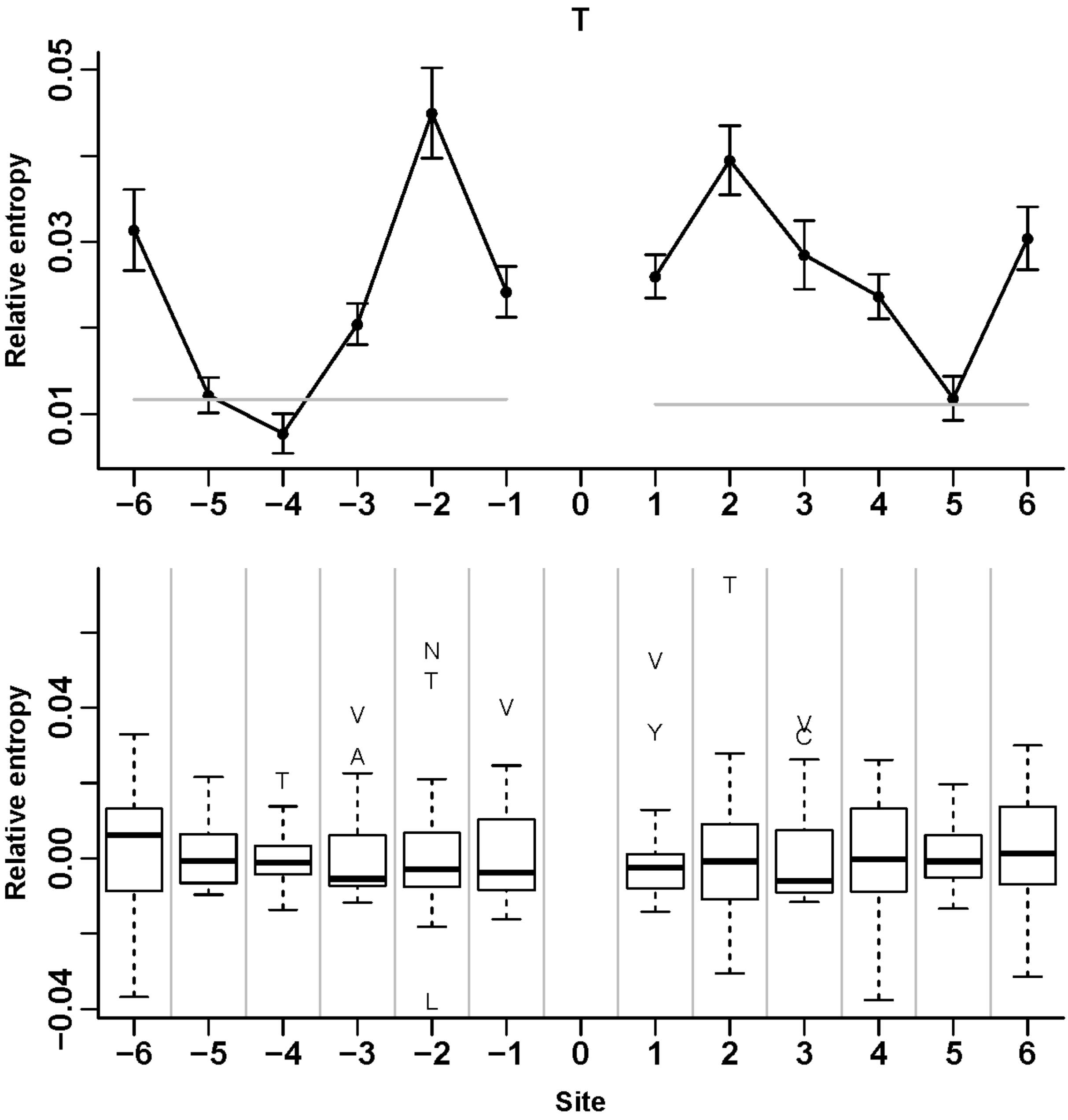
T
Relative entropy
0.05
0.03
0.01
−6
−5
−4
−3
−2
−1
0
1
2
3
4
5
6
Relative entropy
0.04
0.00
−0.04
T
V
A
N
T
L
V
V
Y
T
V
C
Site

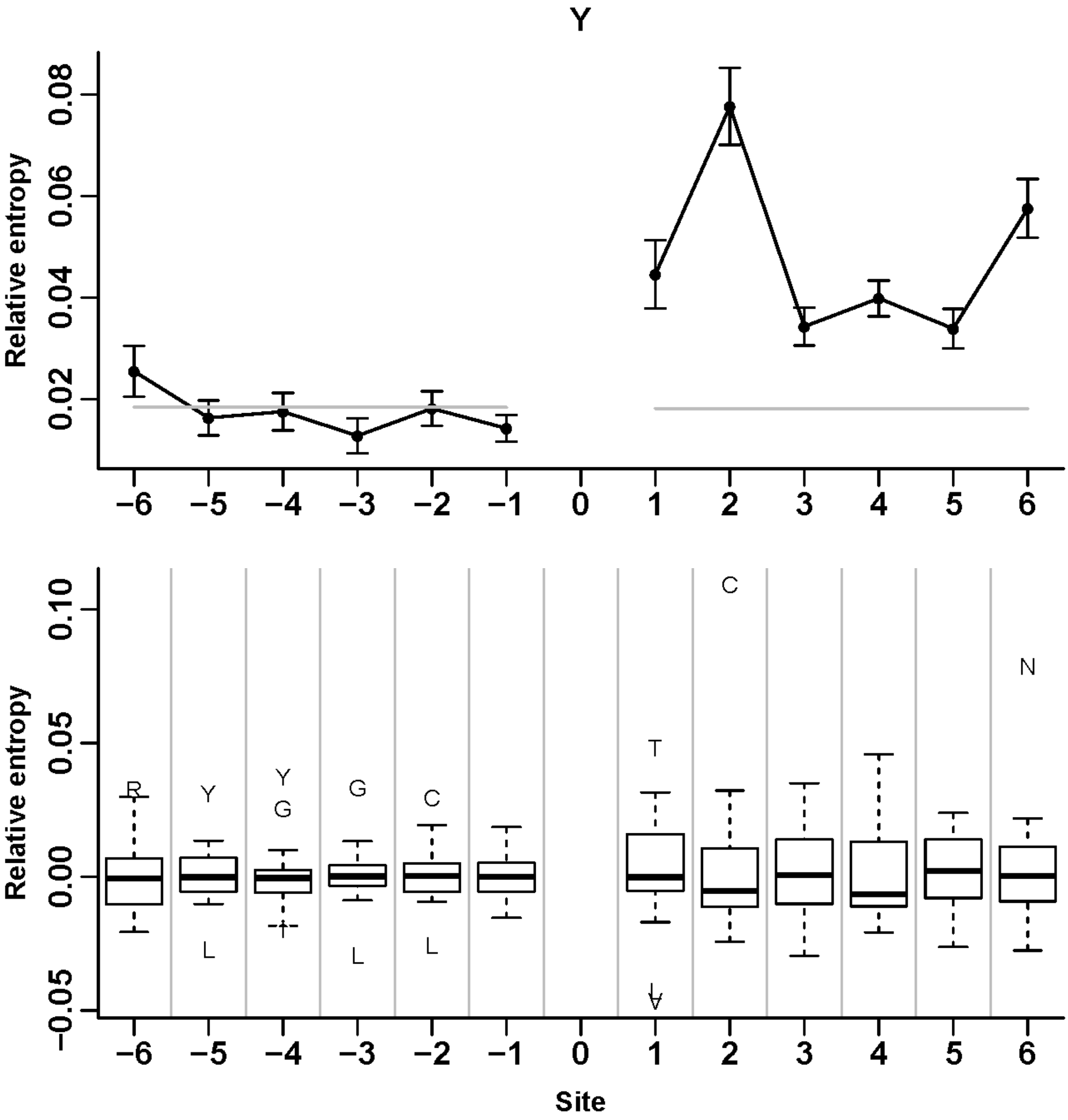

Y
Relative entropy
0.02
0.04
0.06
0.08
−6 −5 −4 −3 −2 −1 0 1 2 3 4 5 6
Relative entropy
−0.05
0.00
0.05
0.10
R Y Y G G C T C N
L T L L V
−6 −5 −4 −3 −2 −1 0 1 2 3 4 5 6
Site

Figure S4. Neighbor preference patterns of the 20 amino acids in coil.

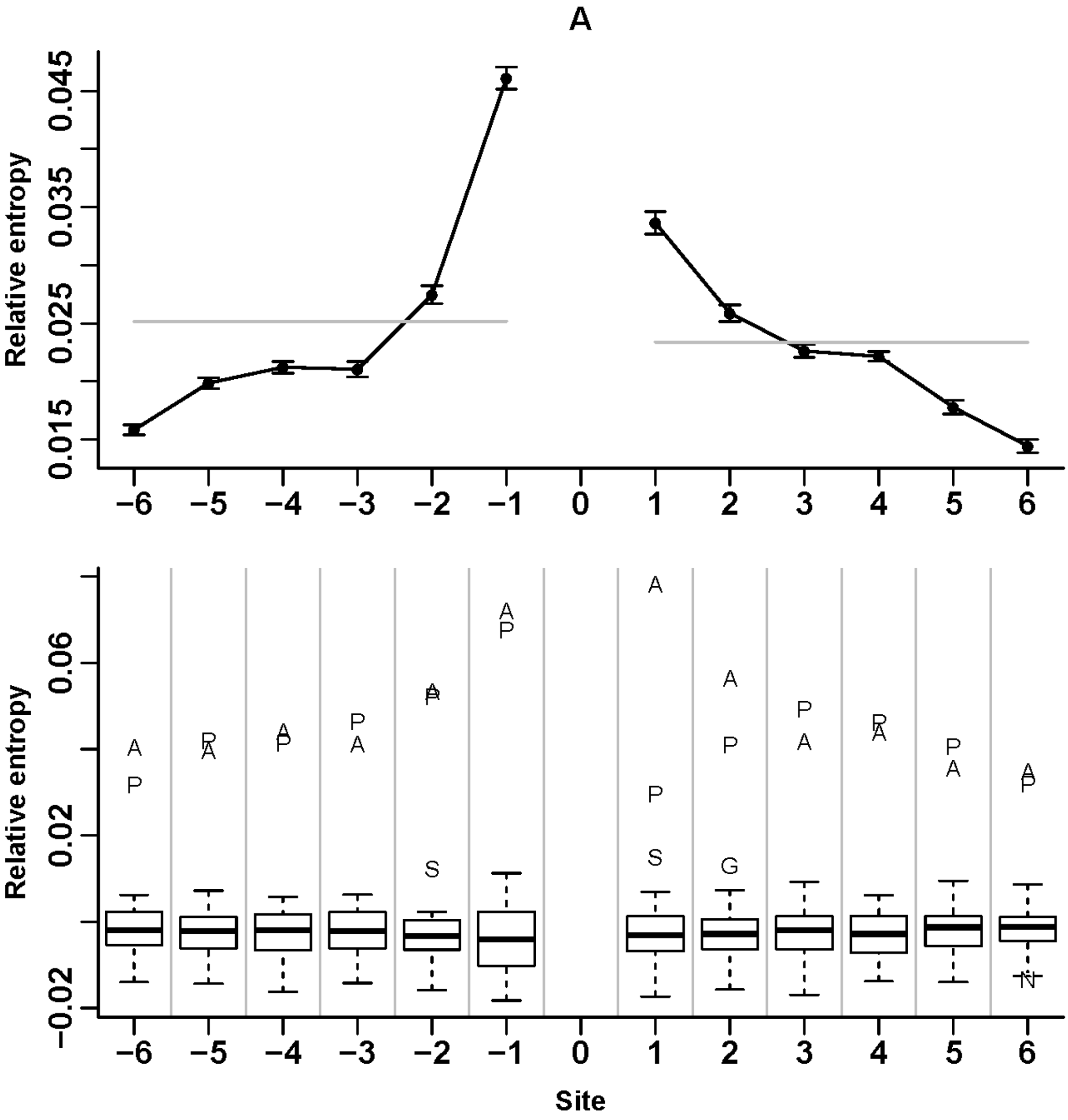

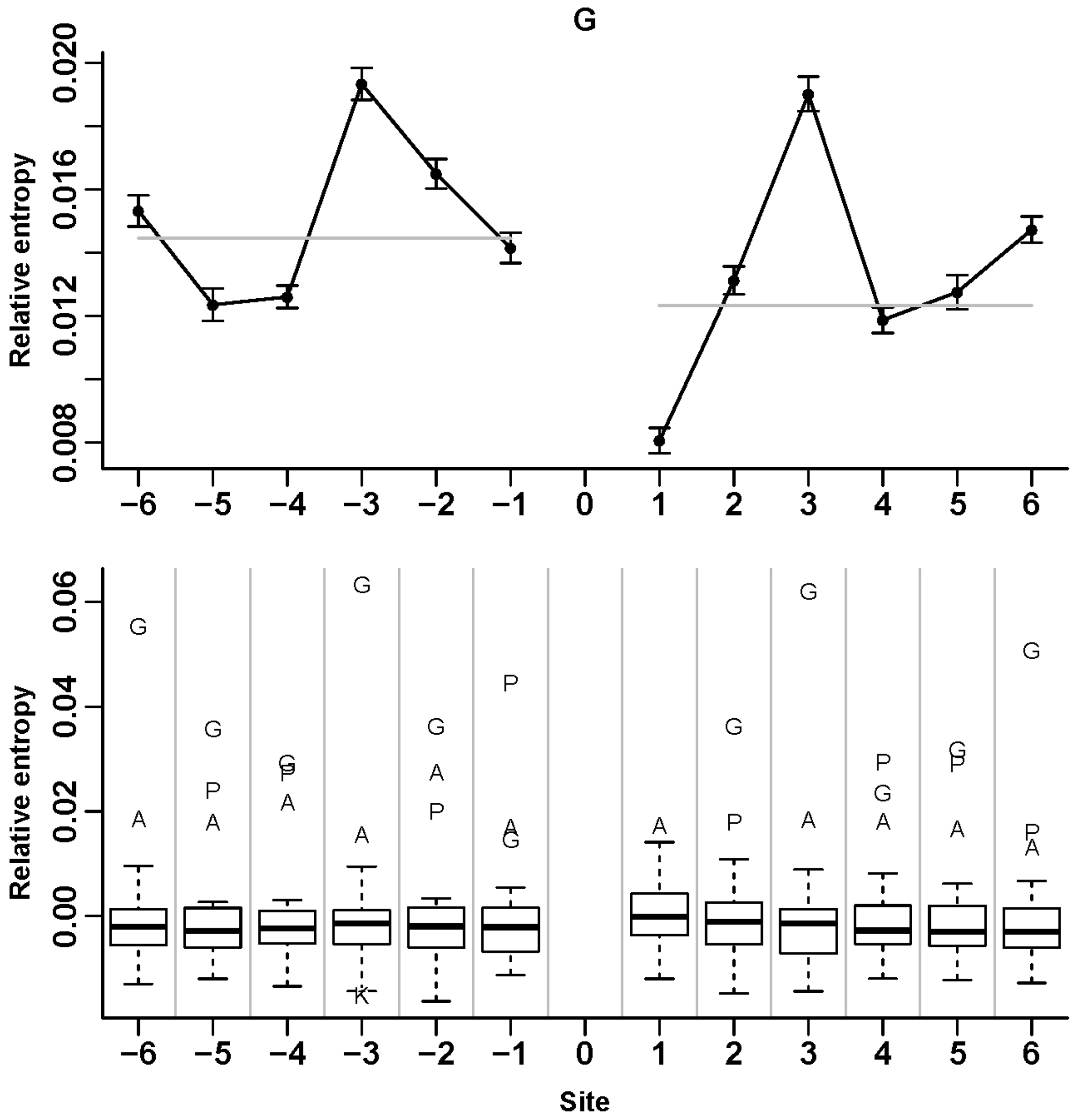
G
Relative entropy
0.008
0.012
0.016
0.020
−6 −5 −4 −3 −2 −1 0 1 2 3 4 5 6
Relative entropy
0.00
0.02
0.04
0.06
Site

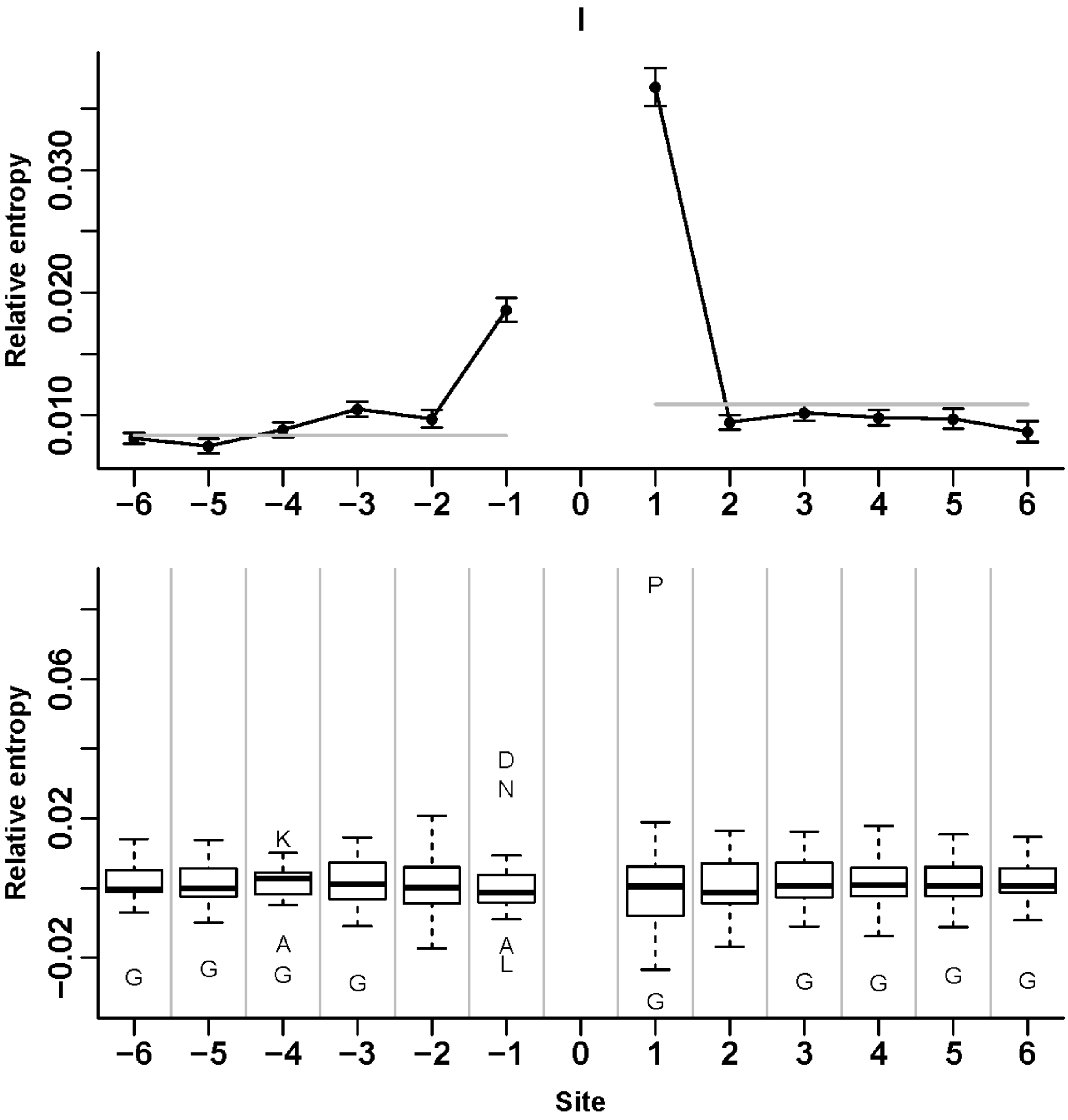
I
Relative entropy
0.010
0.020
0.030
−6
−5
−4
−3
−2
−1
0
1
2
3
4
5
6
Relative entropy
−0.02
0.02
0.06
P
D
N
K
A
A
L
G
G
G
G
G
G
G
G
G
G
Site

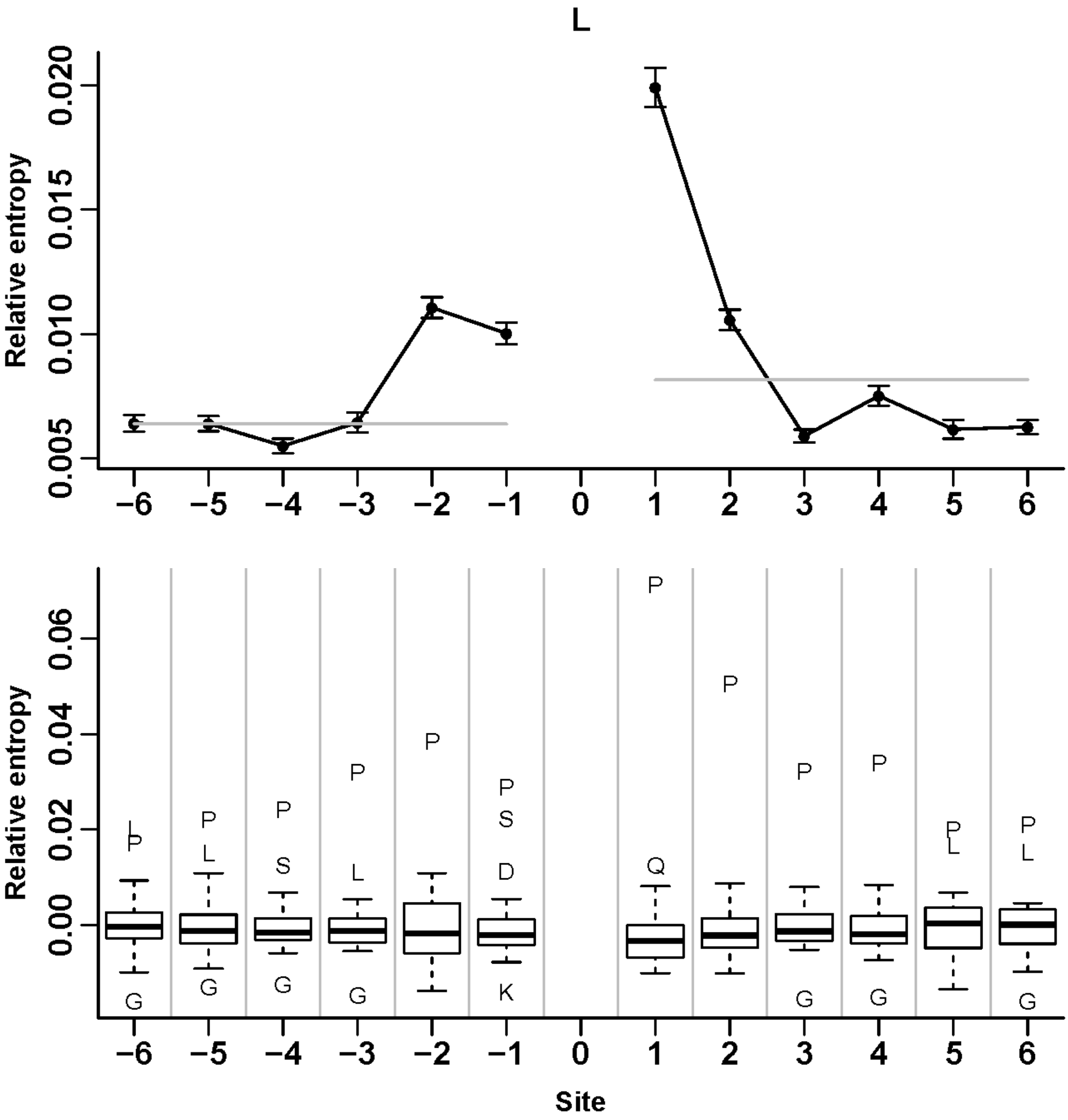
L
Relative entropy
0.005
0.010
0.015
0.020
−6
−5
−4
−3
−2
−1
0
1
2
3
4
5
6
Relative entropy
0.00
0.02
0.04
0.06
Site

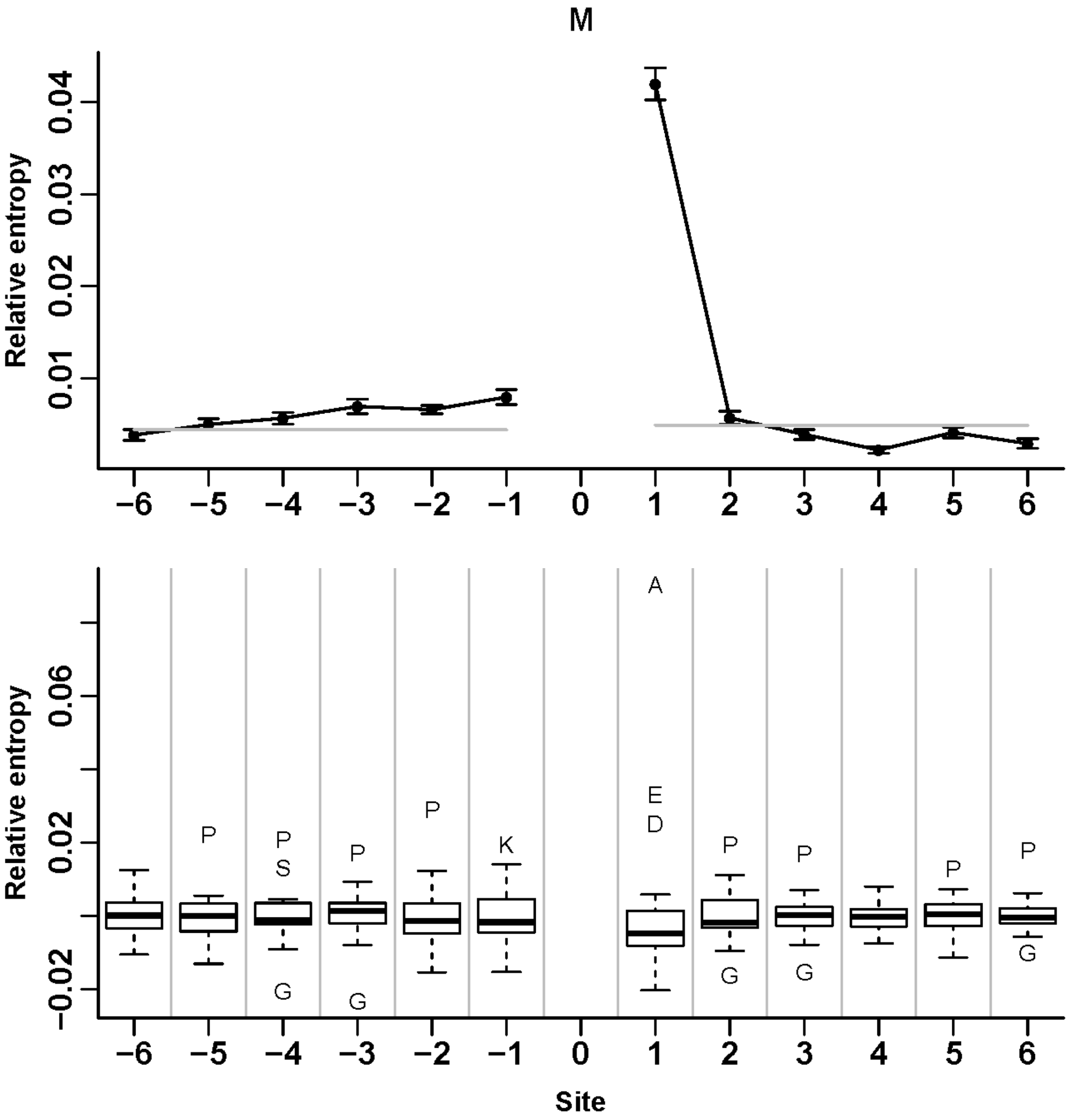
M
Relative entropy
0.01
0.02
0.03
0.04
−6
−5
−4
−3
−2
−1
0
1
2
3
4
5
6
Relative entropy
−0.02
0.02
0.06
A
E
D
P
P
S
G
P
G
P
K
P
G
P
G
P
P
G
Site

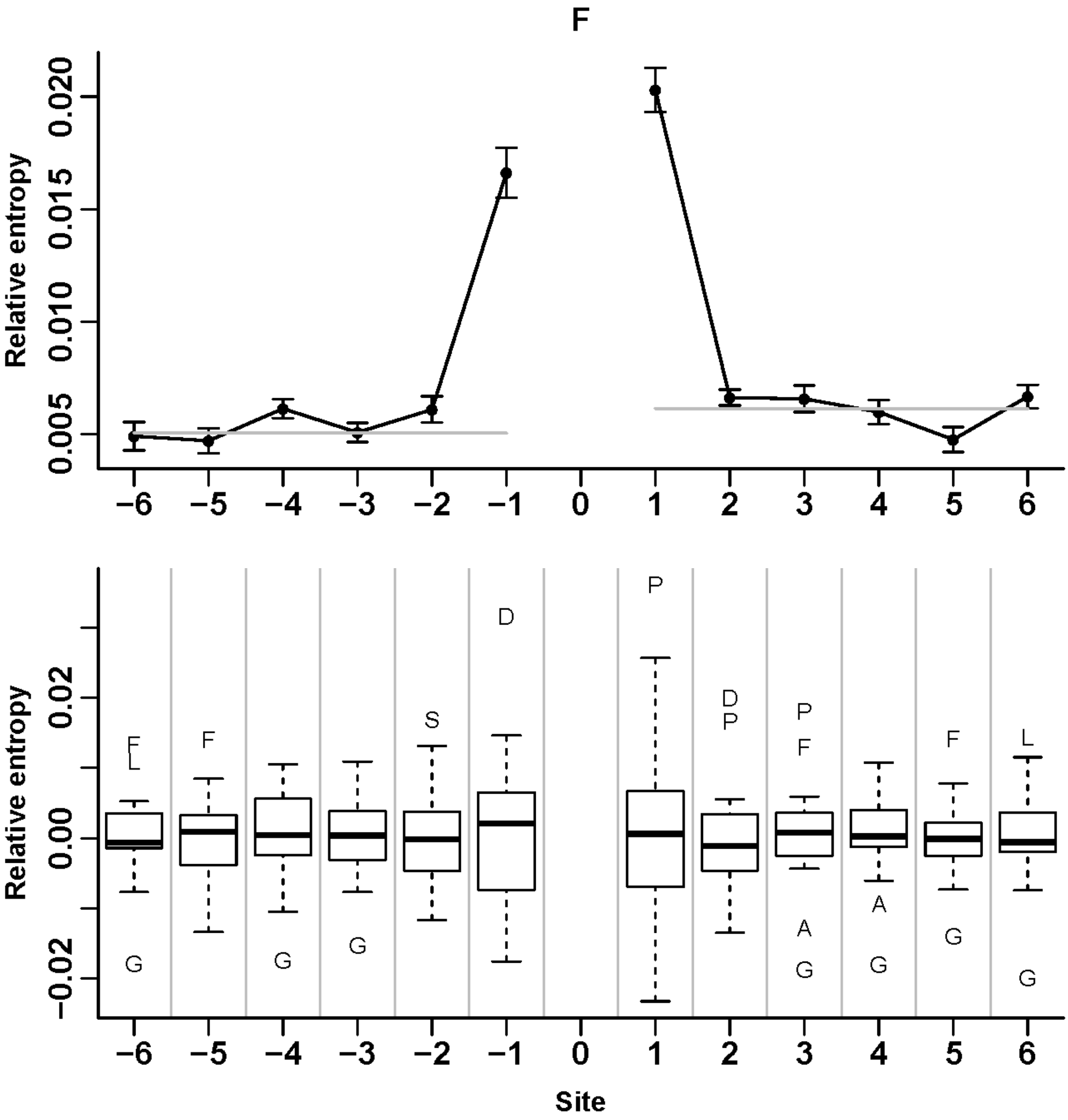
F
Relative entropy
0.005
0.010
0.015
0.020
−6
−5
−4
−3
−2
−1
0
1
2
3
4
5
6
Relative entropy
−0.02
0.00
0.02
F
L
G
F
G
G
S
D
P
D
P
P
F
A
G
A
G
F
G
L
G
Site

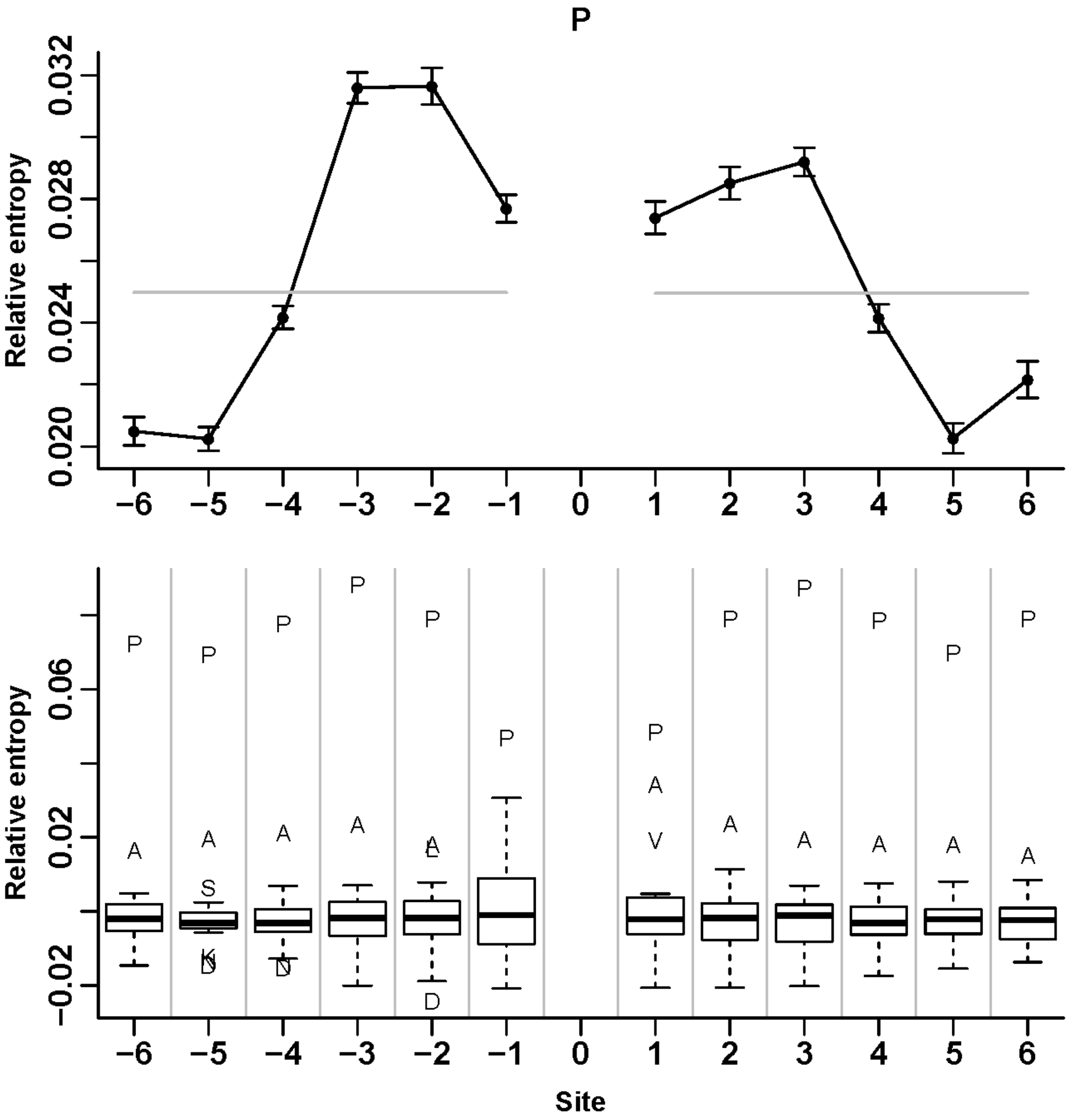
P
Relative entropy
0.020
0.024
0.028
0.032
−6
−5
−4
−3
−2
−1
0
1
2
3
4
5
6
Relative entropy
−0.02
0.02
0.06
Site

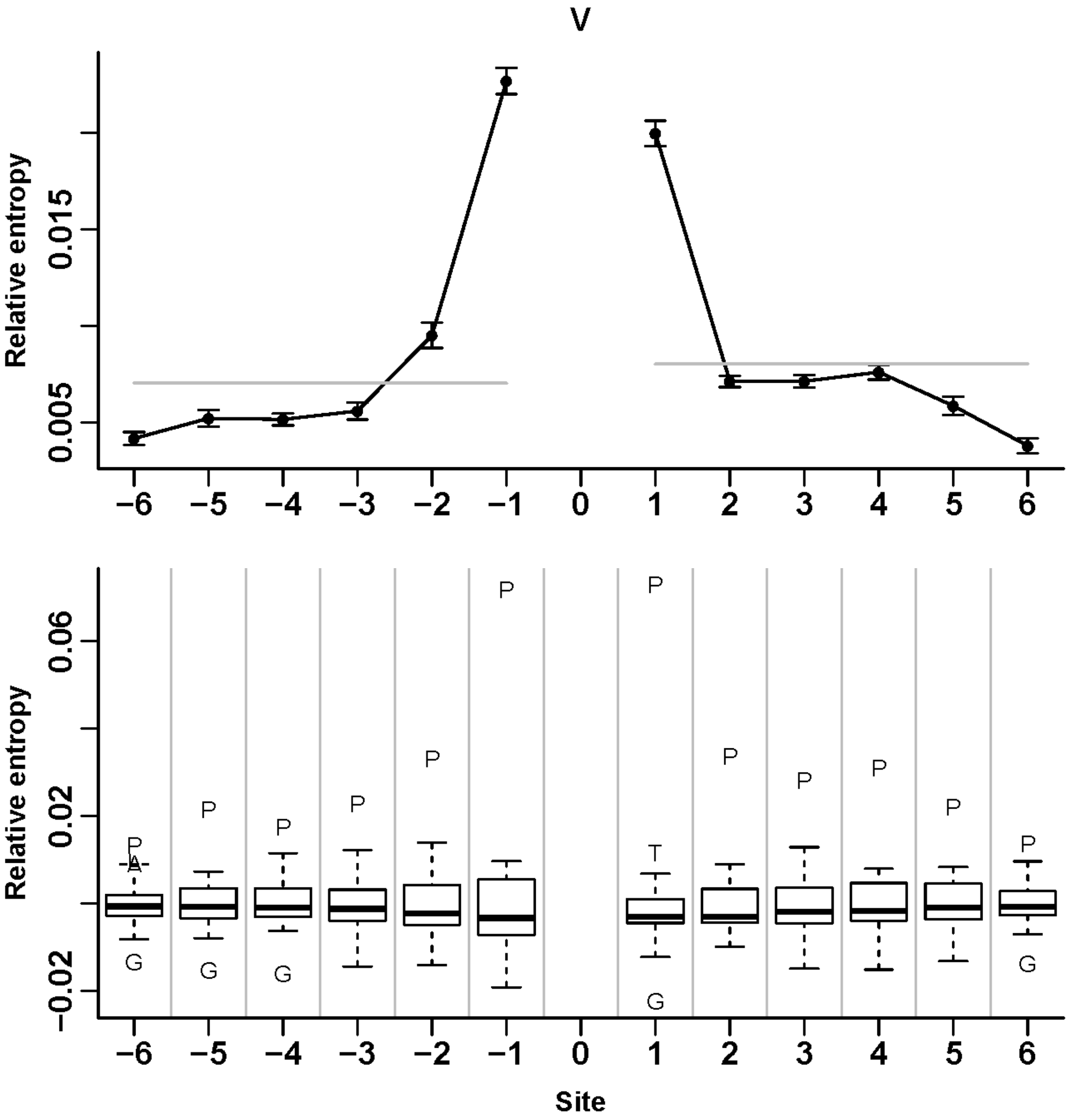
V
Relative entropy
0.005
0.015
−6
−5
−4
−3
−2
−1
0
1
2
3
4
5
6
Relative entropy
−0.02
0.02
0.06
P
A
G
T
Site

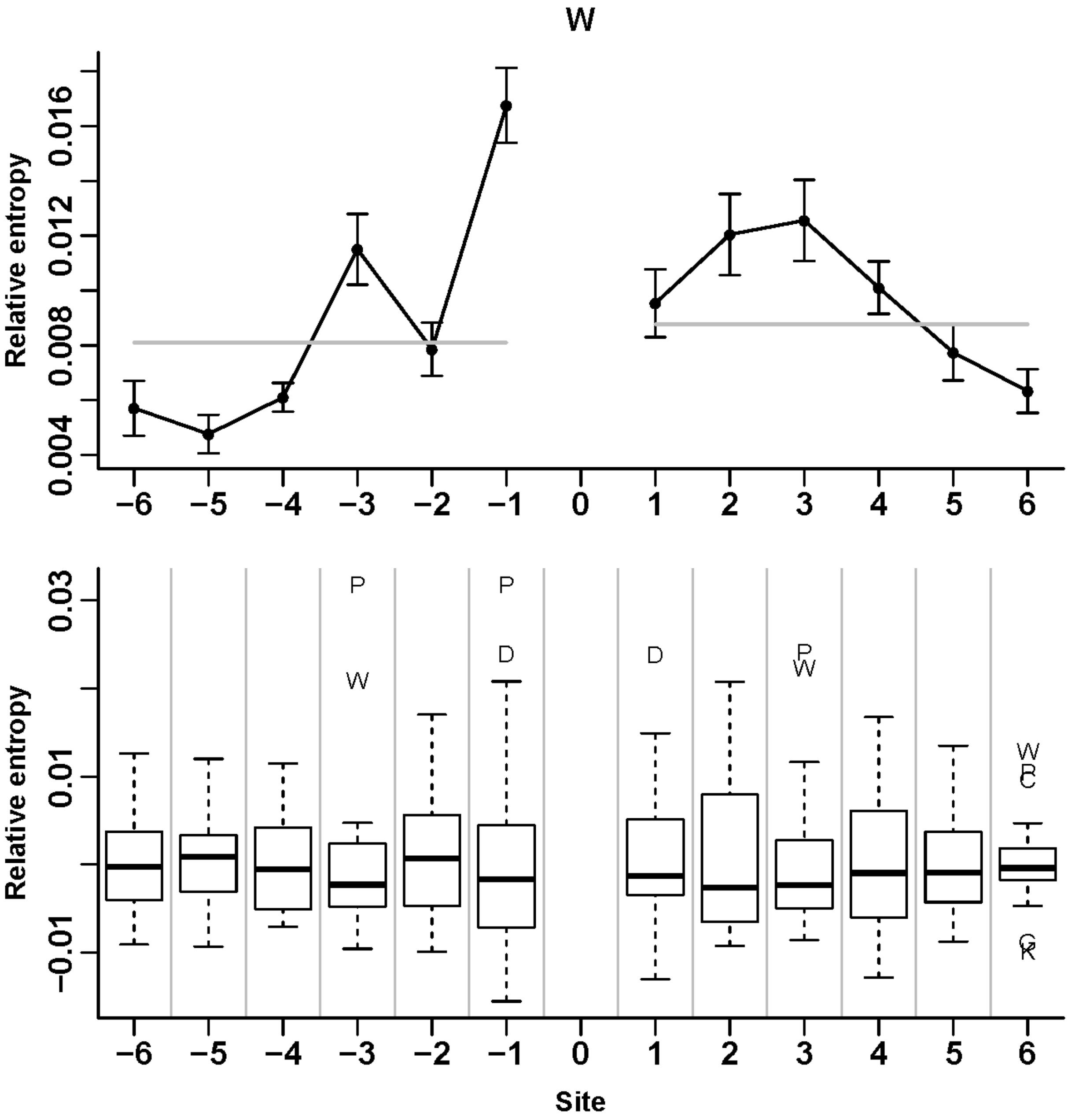
W
Relative entropy
0.004
0.008
0.012
0.016
−6
−5
−4
−3
−2
−1
0
1
2
3
4
5
6
P
W
P
D
D
P
W
W
P
C
C
K
Relative entropy
−0.01
0.01
0.03
Site

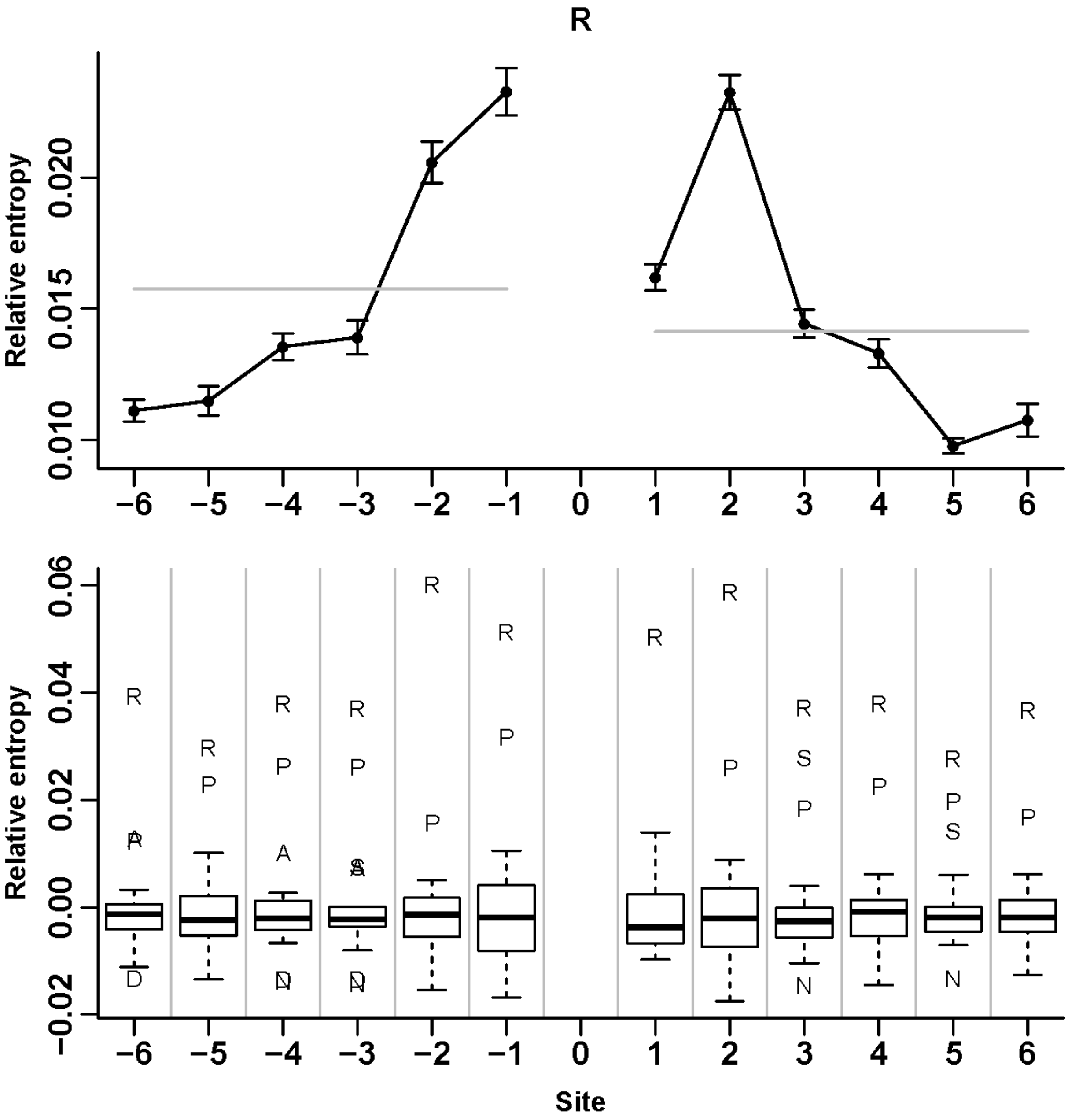
R
Relative entropy
0.010
0.015
0.020
−6
−5
−4
−3
−2
−1
0
1
2
3
4
5
6
Relative entropy
−0.02
0.00
0.02
0.04
0.06
Site

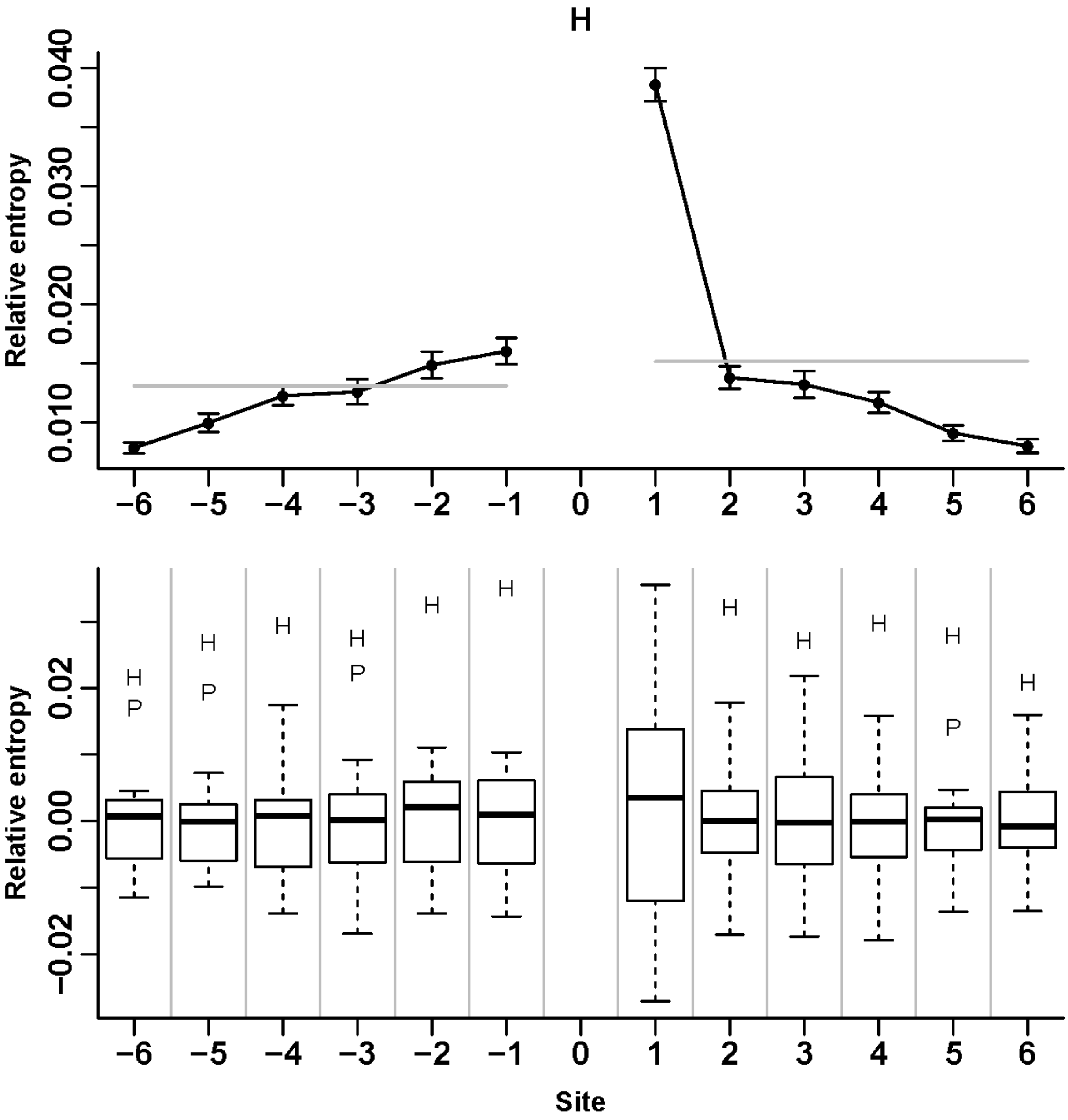
H
Relative entropy
0.010
0.020
0.030
0.040
−6
−5
−4
−3
−2
−1
0
1
2
3
4
5
6
Relative entropy
−0.02
0.00
0.02
H
P
Site

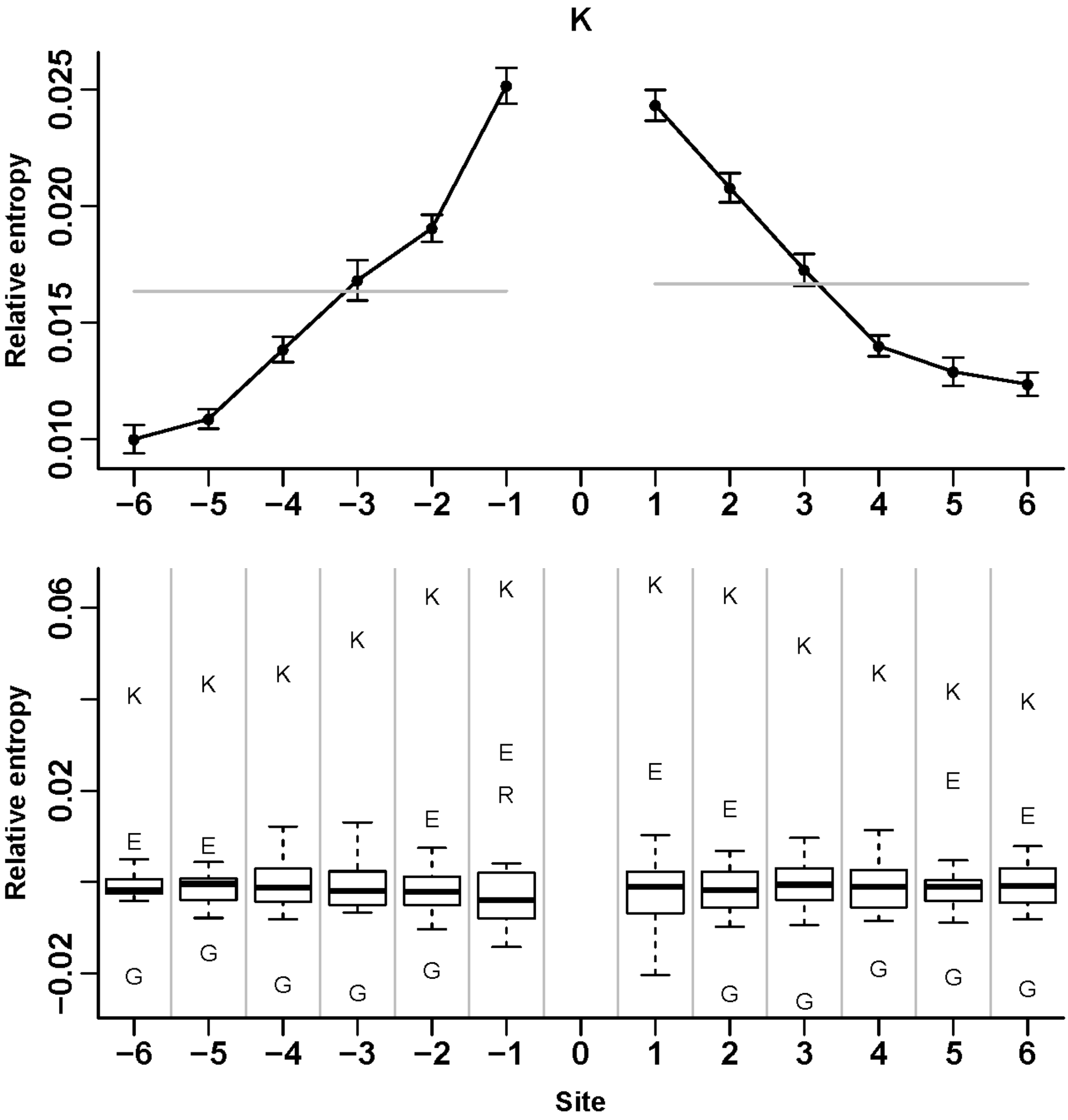
K
Relative entropy
0.010
0.015
0.020
0.025
−6
−5
−4
−3
−2
−1
0
1
2
3
4
5
6
Relative entropy
−0.02
0.02
0.06
K
E
G
R
Site

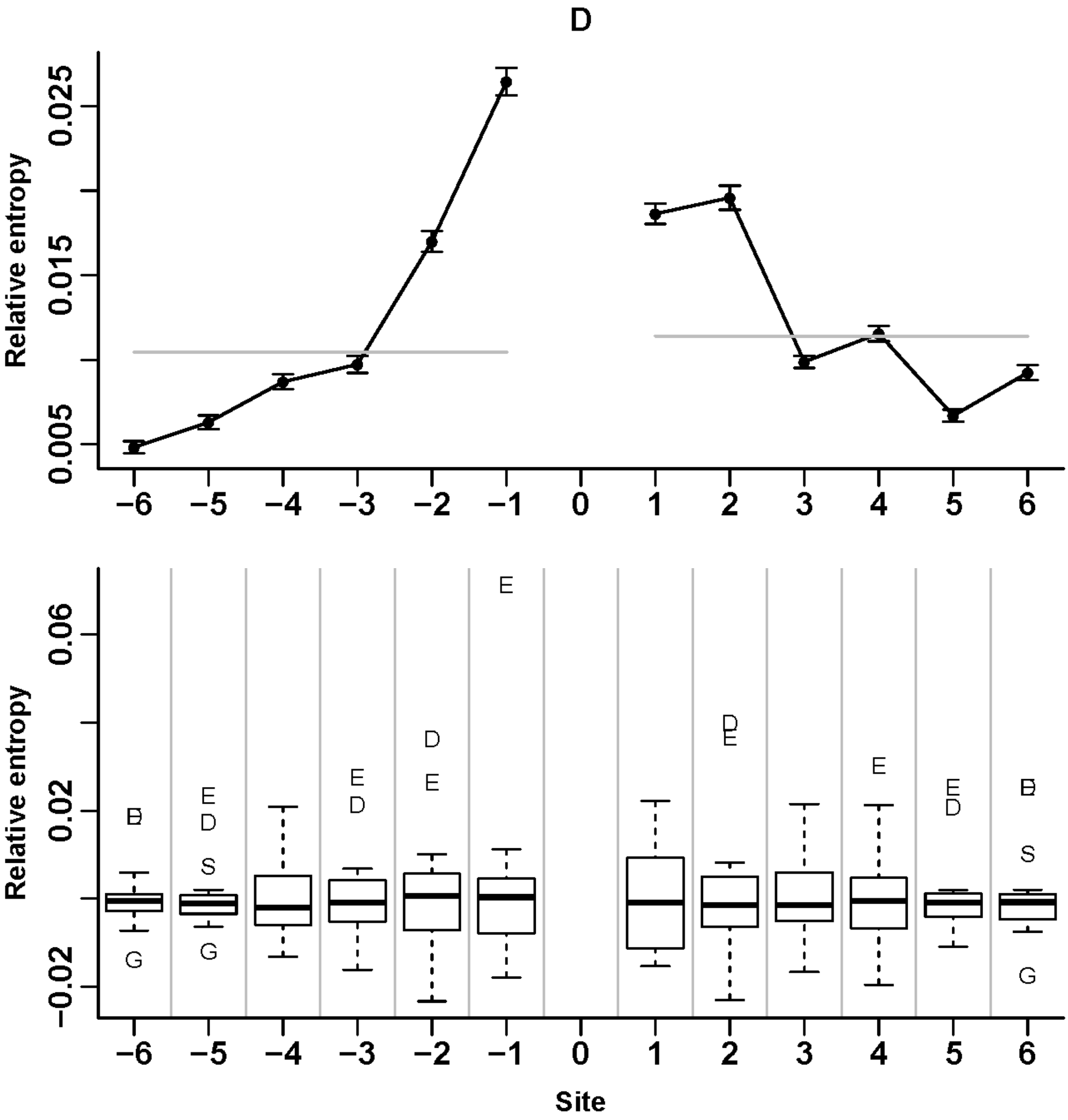
D
Relative entropy
0.005
0.015
0.025
−6
−5
−4
−3
−2
−1
0
1
2
3
4
5
6
Relative entropy
−0.02
0.02
0.06
E
D
E
D
E
D
E
S
G
G
S
G
Site

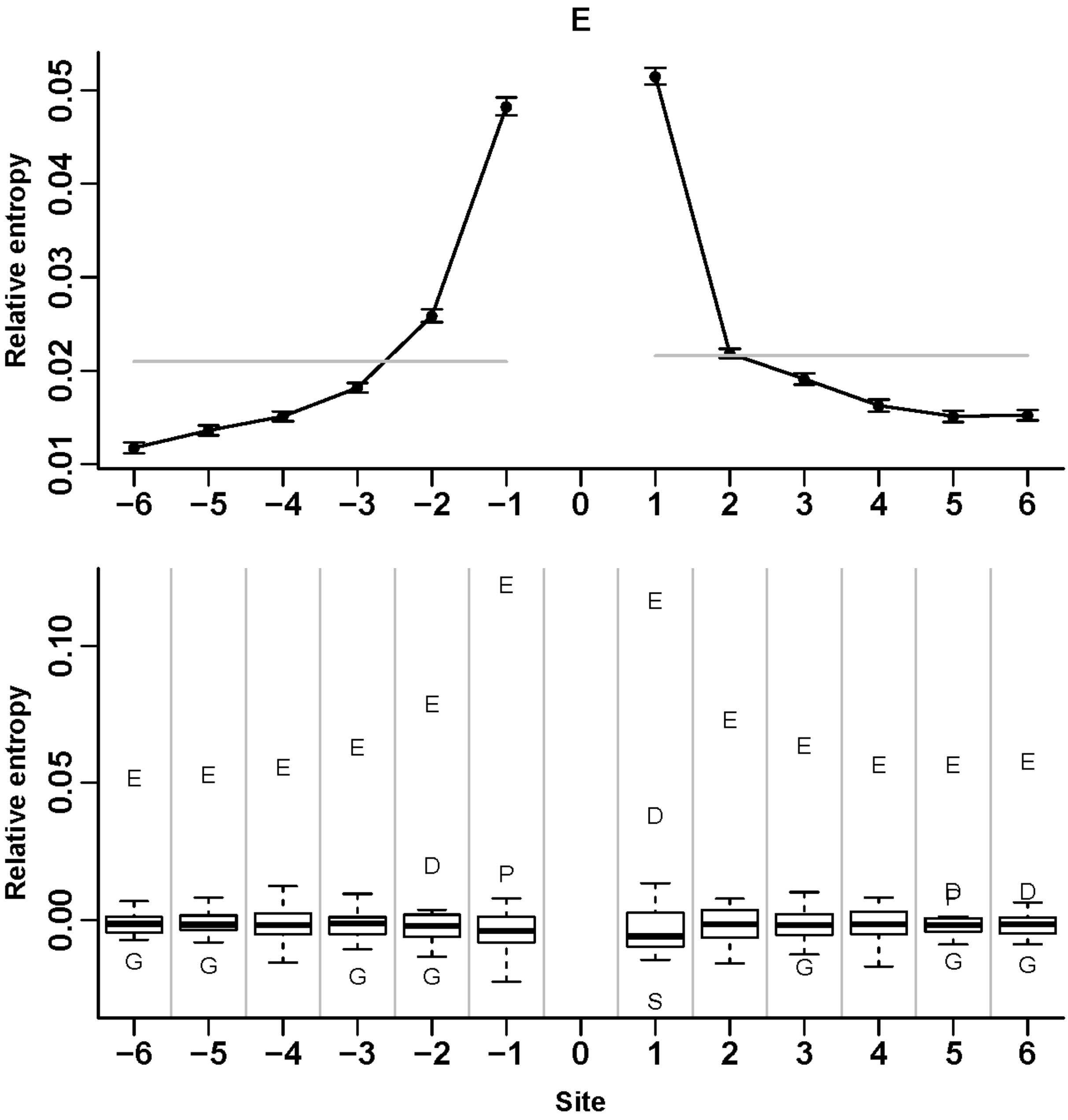
E
Relative entropy
0.01
0.02
0.03
0.04
0.05
−6 −5 −4 −3 −2 −1 0 1 2 3 4 5 6
Relative entropy
0.00
0.05
0.10
E E E E E E E E E E E E
G G G G G G
D D D
P P P
S
−6 −5 −4 −3 −2 −1 0 1 2 3 4 5 6
Site

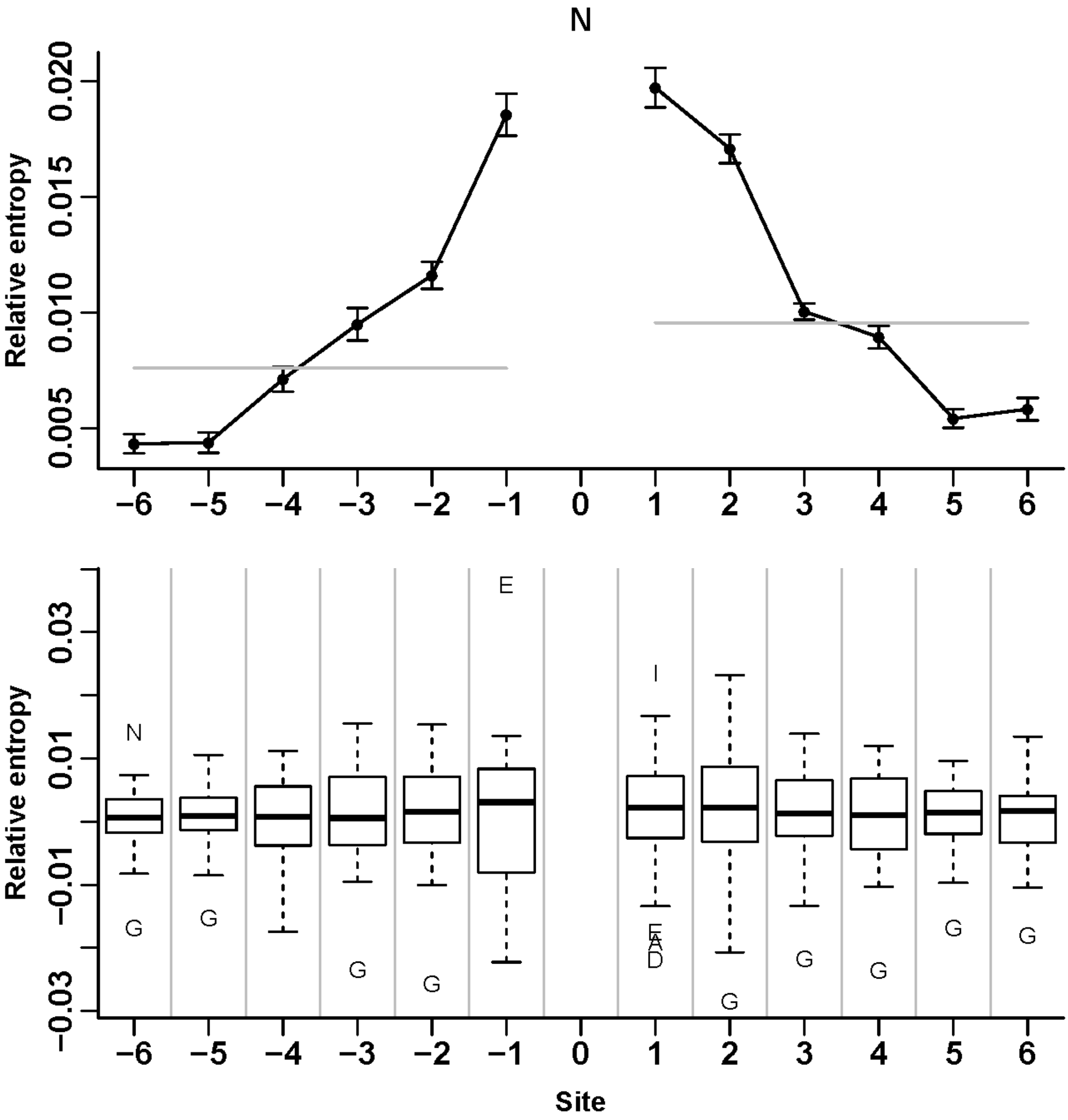
N
Relative entropy
0.005
0.010
0.015
0.020
−6
−5
−4
−3
−2
−1
0
1
2
3
4
5
6
Relative entropy
−0.03
−0.01
0.01
0.03
E
I
N
G
G
G
G
G
G
G
G
G
G
D
Site

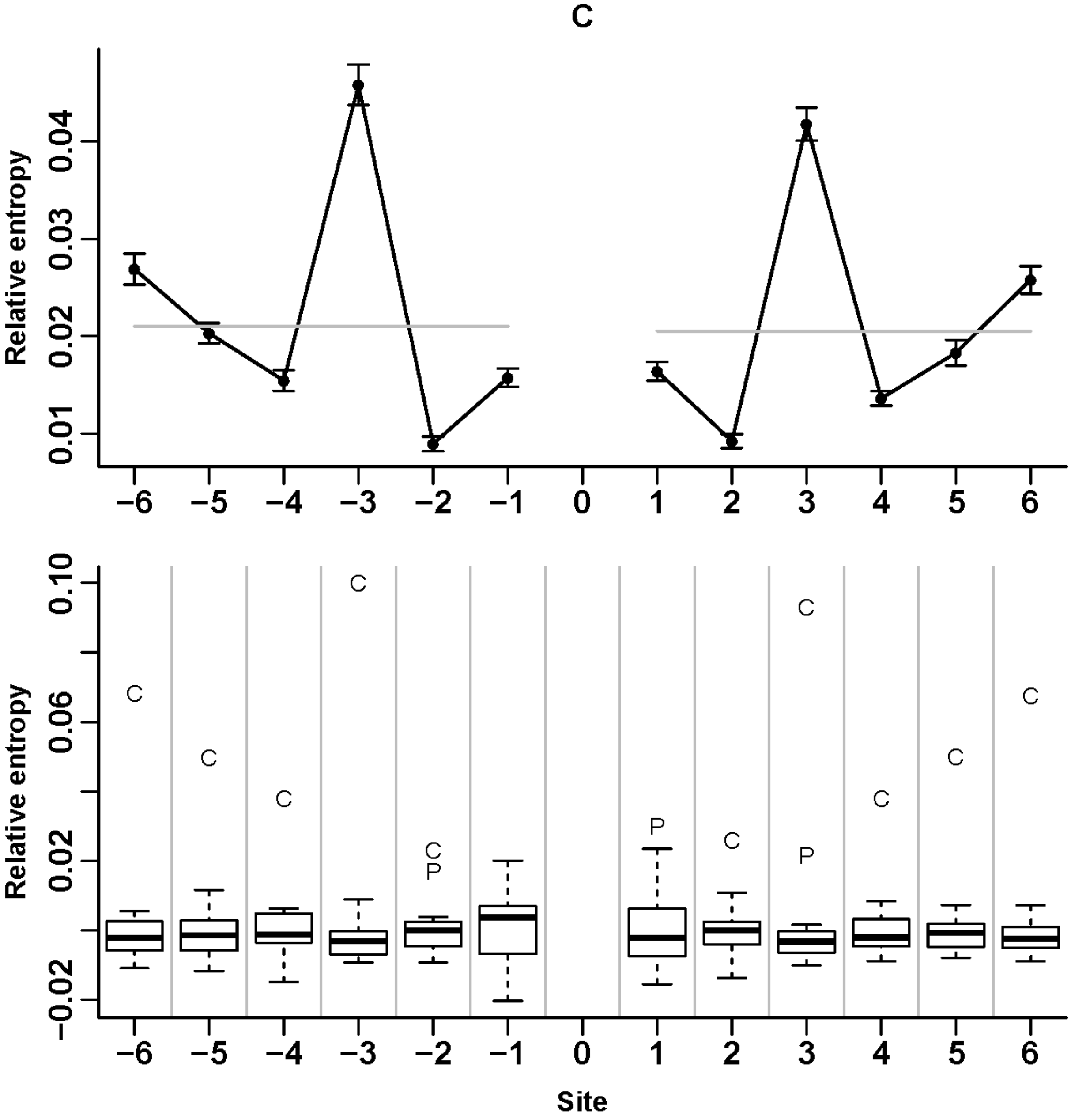
C
Relative entropy
0.01
0.02
0.03
0.04
−6
−5
−4
−3
−2
−1
0
1
2
3
4
5
6
Relative entropy
−0.02
0.02
0.06
0.10
C
P
Site

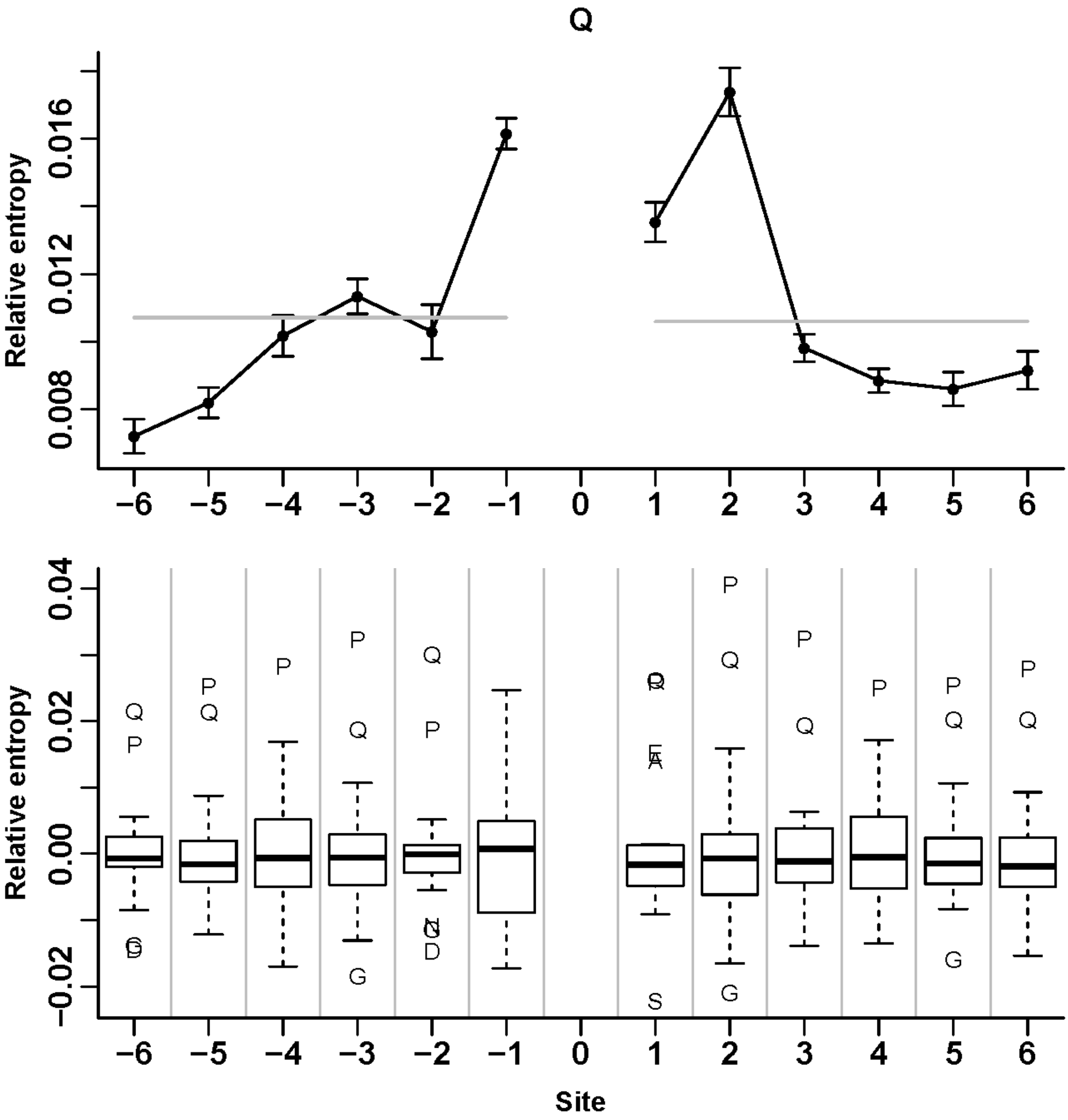
Q
Relative entropy
0.008
0.012
0.016
−6
−5
−4
−3
−2
−1
0
1
2
3
4
5
6
Relative entropy
−0.02
0.00
0.02
0.04
Q
P
G
D
P
Q
P
P
Q
G
Q
P
N
G
D
Q
R
E
A
S
P
Q
G
P
Q
P
P
Q
G
P
Q
Site

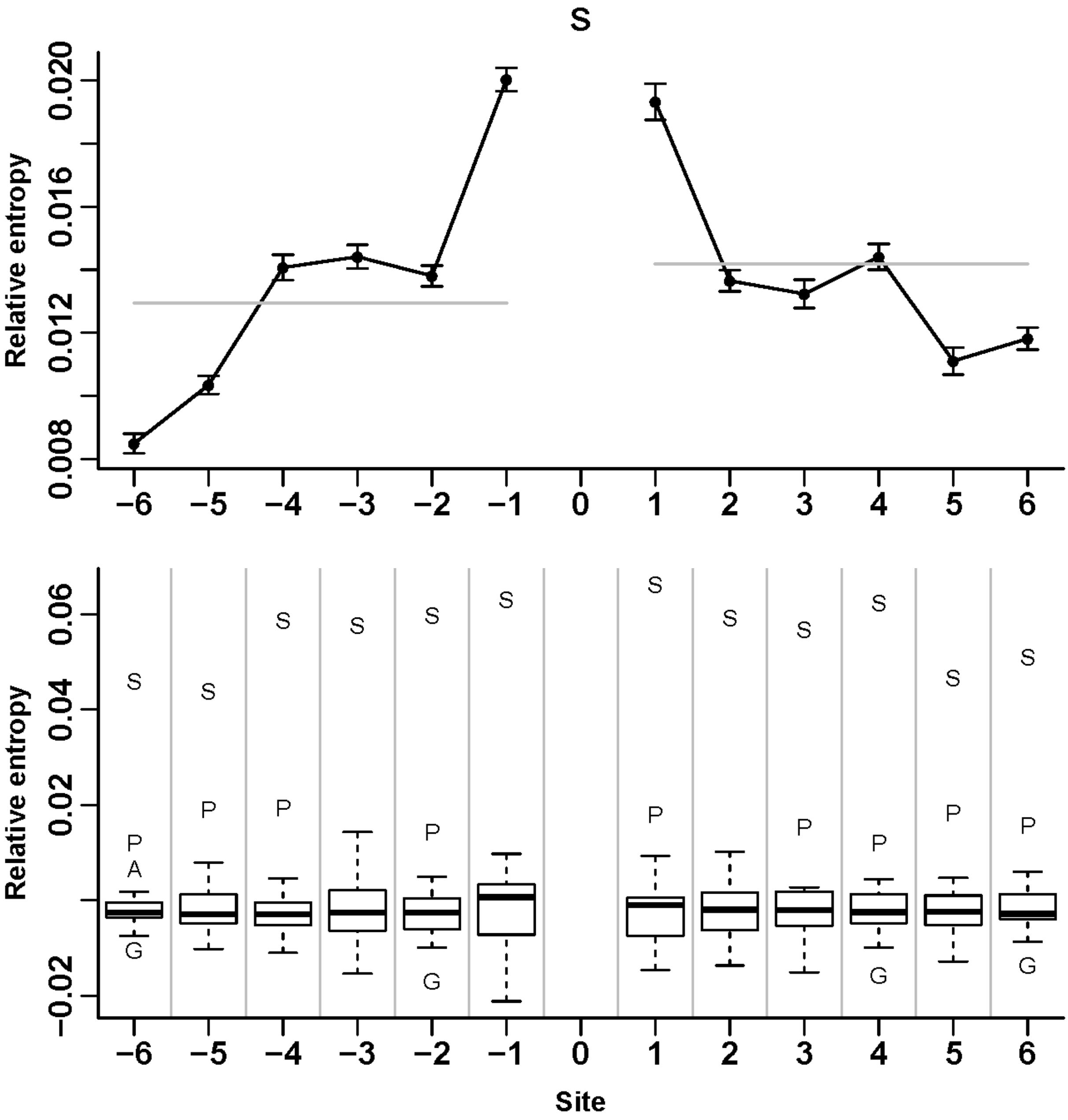
S
Relative entropy
0.008
0.012
0.016
0.020
−6
−5
−4
−3
−2
−1
0
1
2
3
4
5
6
Relative entropy
−0.02
0.02
0.04
0.06
S
P
A
G
Site

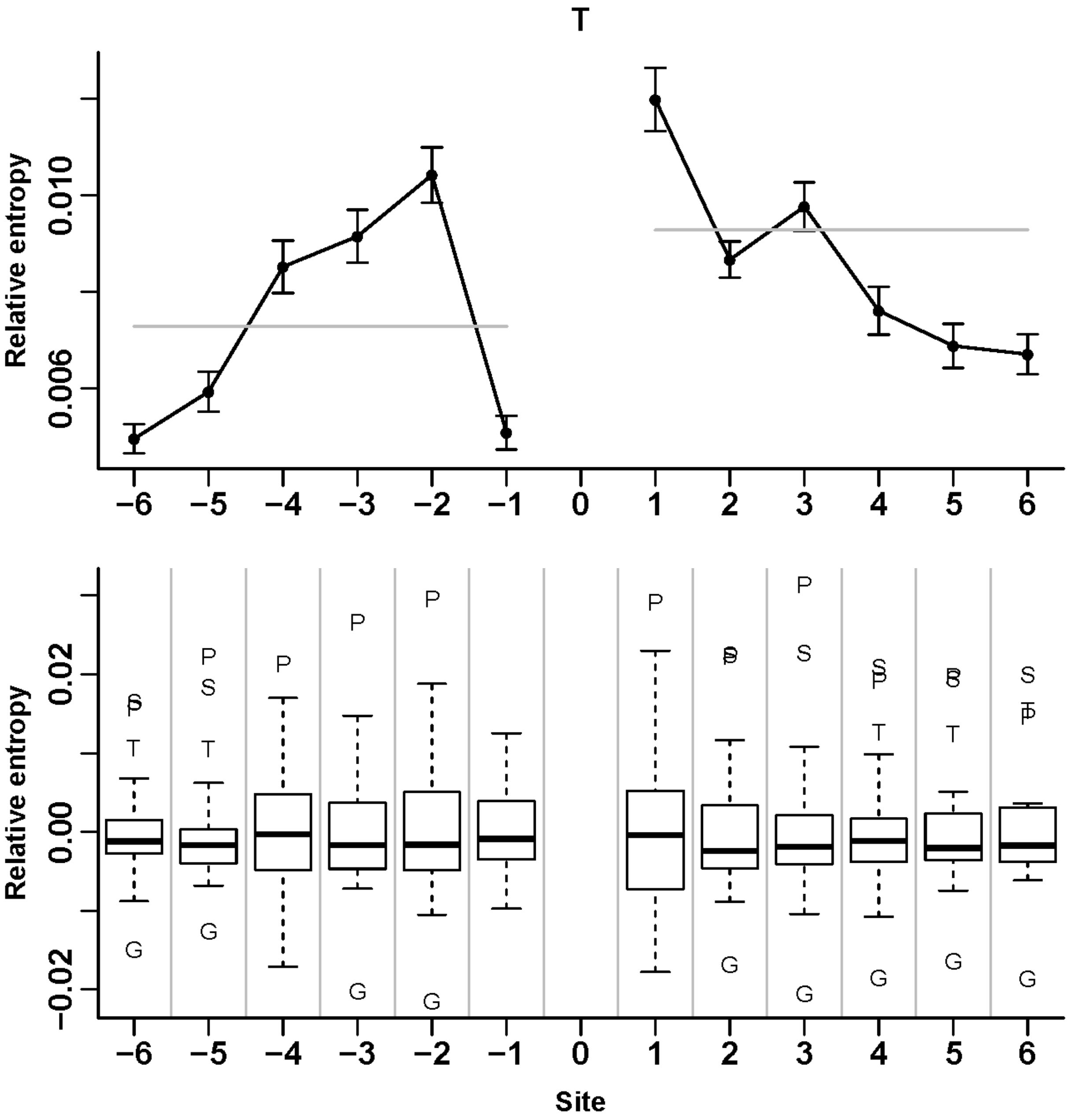
T
Relative entropy
0.006
0.010
−6 −5 −4 −3 −2 −1 0 1 2 3 4 5 6
Relative entropy
−0.02
0.00
0.02
P S T G
Site

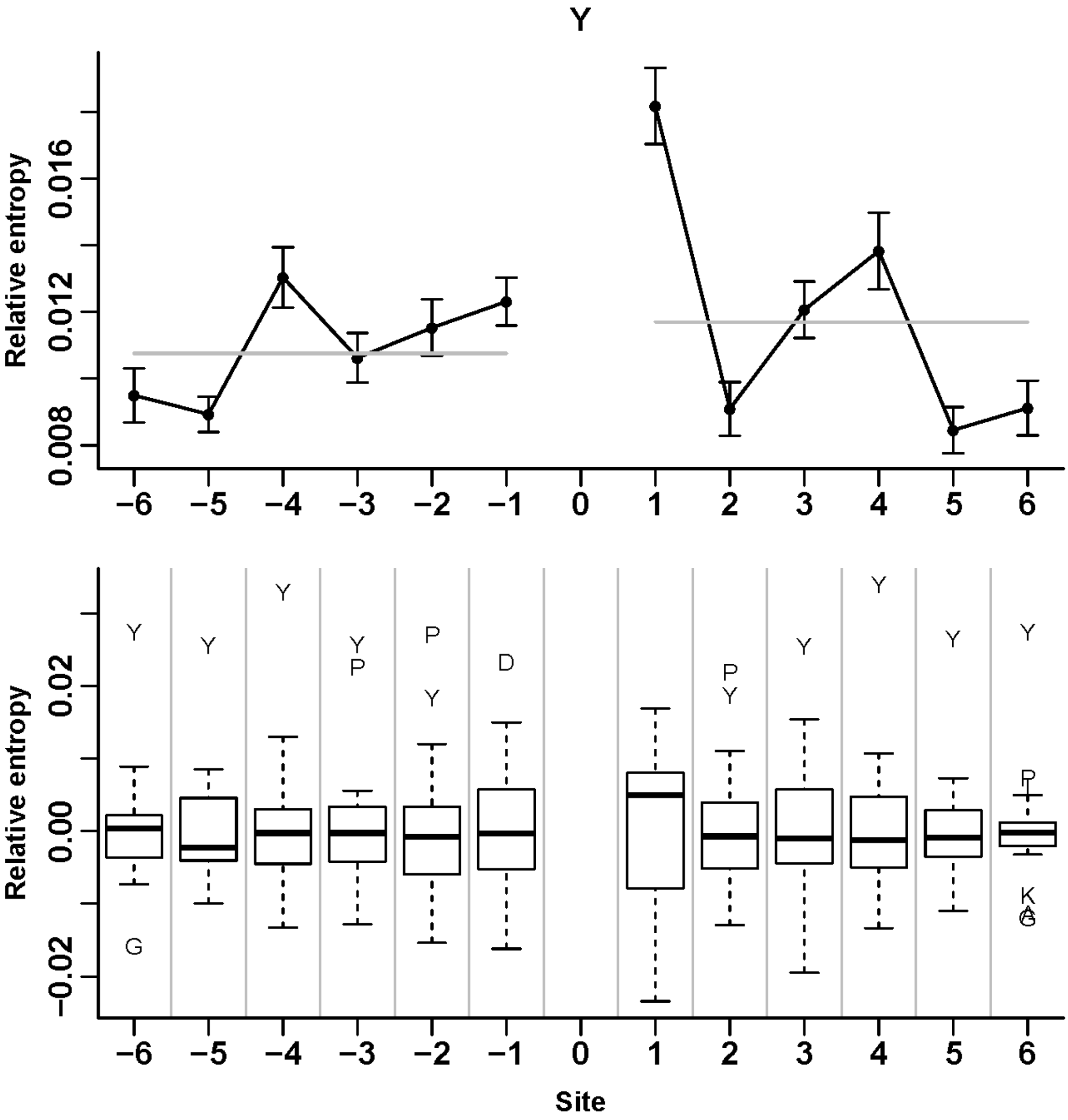
Y
Relative entropy
0.008
0.012
0.016
−6
−5
−4
−3
−2
−1
0
1
2
3
4
5
6
Relative entropy
−0.02
0.00
0.02
Y
Y
Y
Y
P
P
Y
D
P
Y
Y
Y
Y
Y
G
P
K
A
G
Site

Figure S5. Context-dependence patterns of the amino acid substitutions.

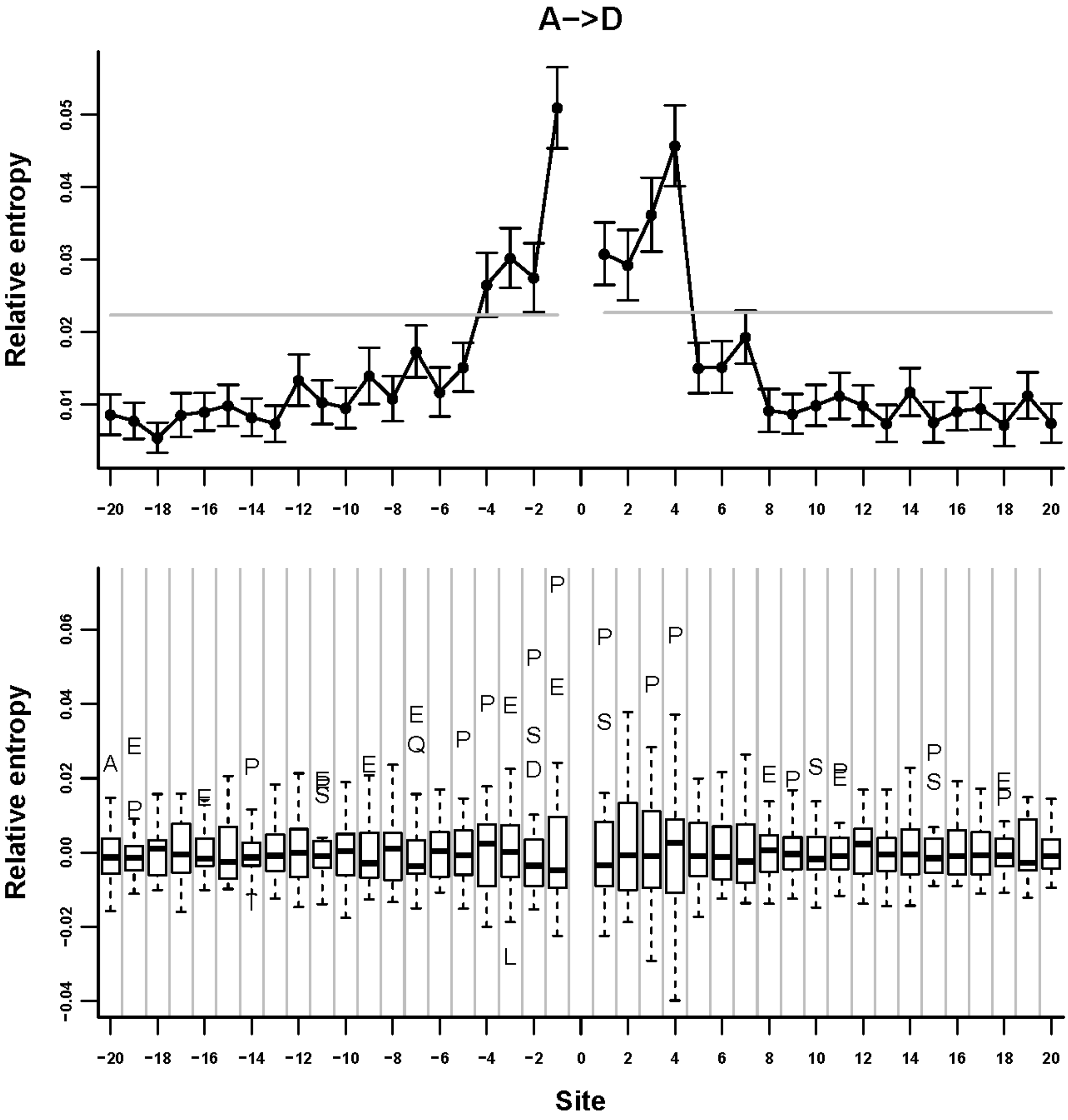

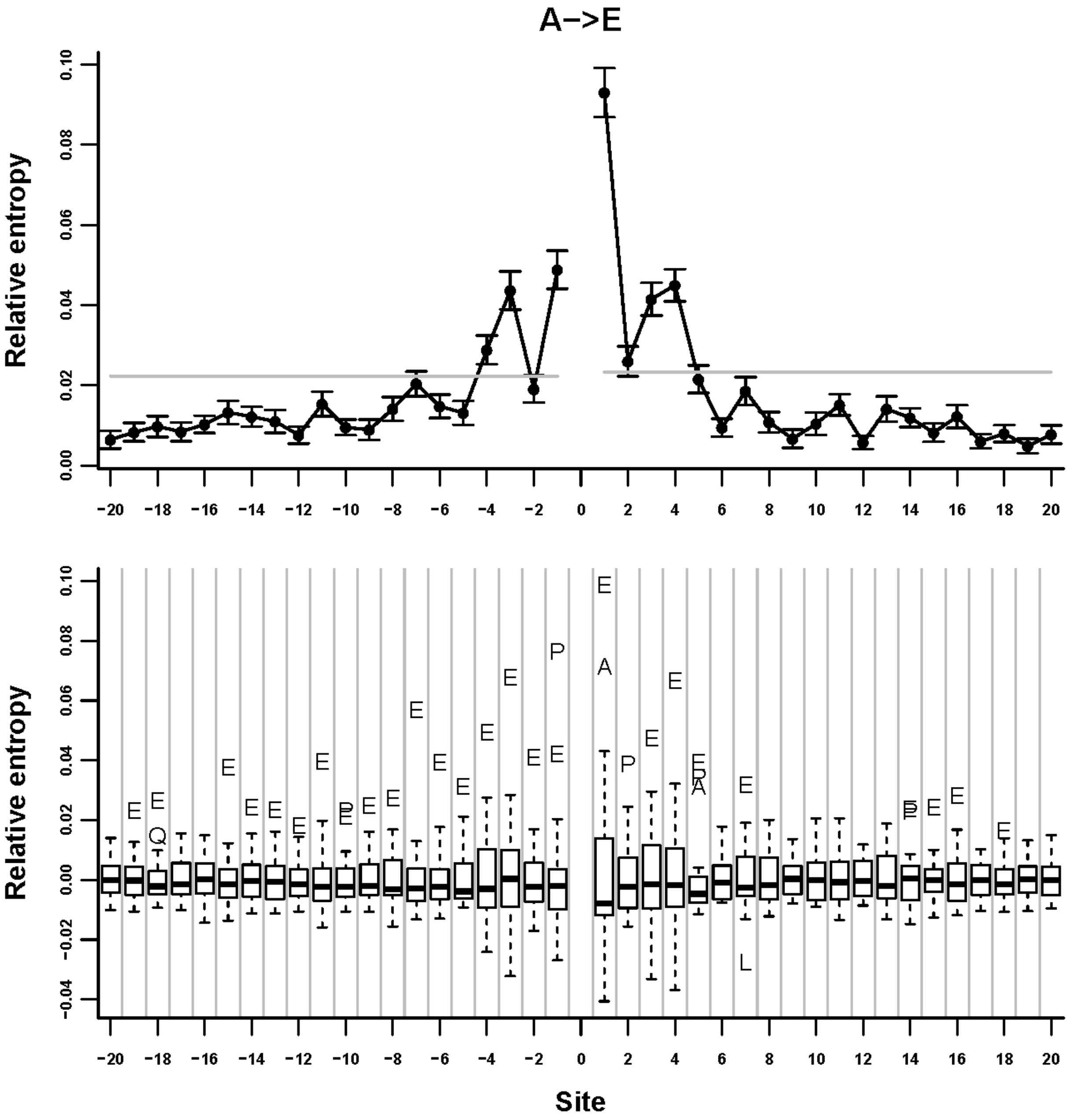
A−>E
Relative entropy
Relative entropy
Site

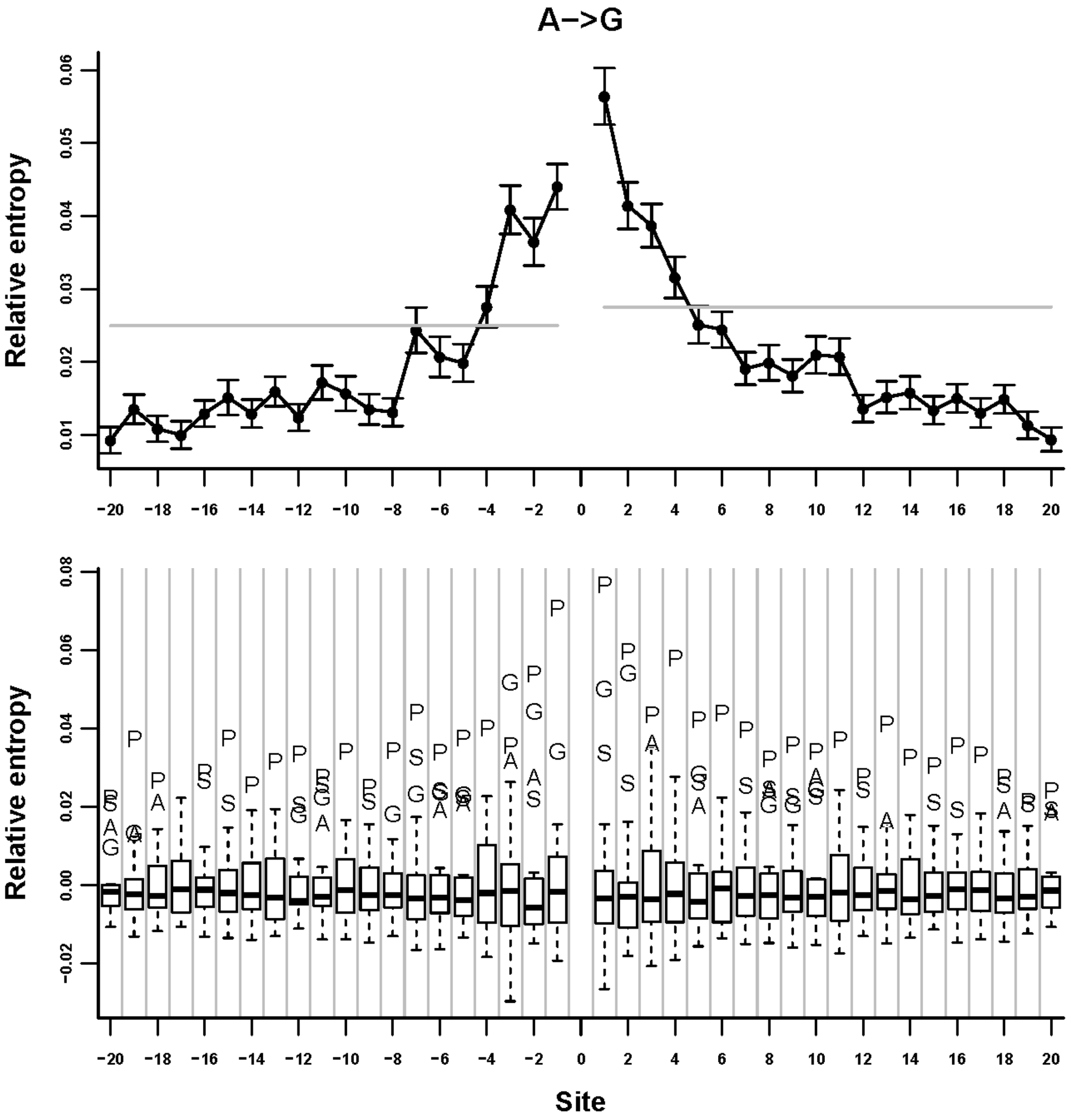
A−>G
Relative entropy
Relative entropy
Site

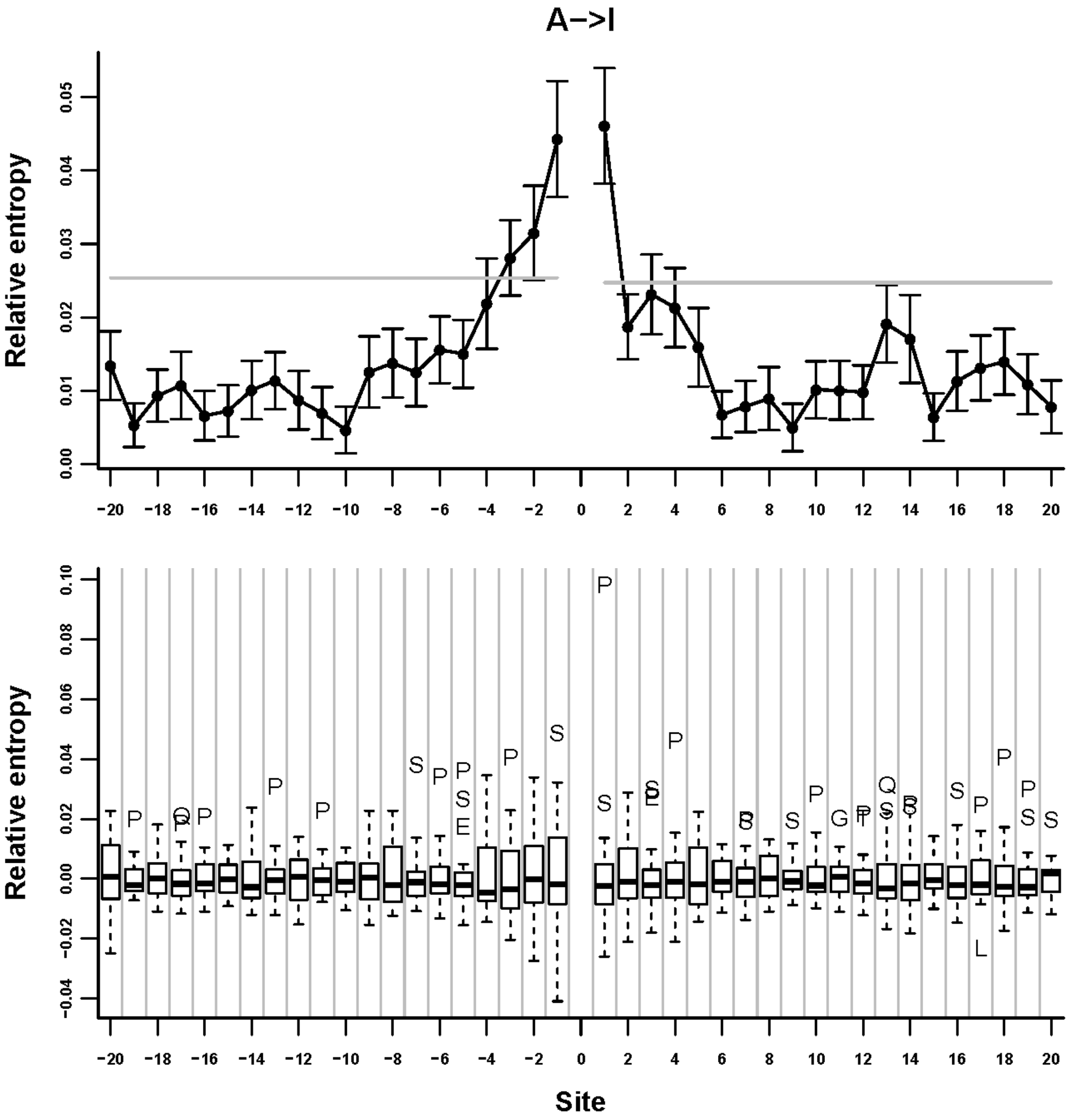
A−>I
Relative entropy
Relative entropy
Site

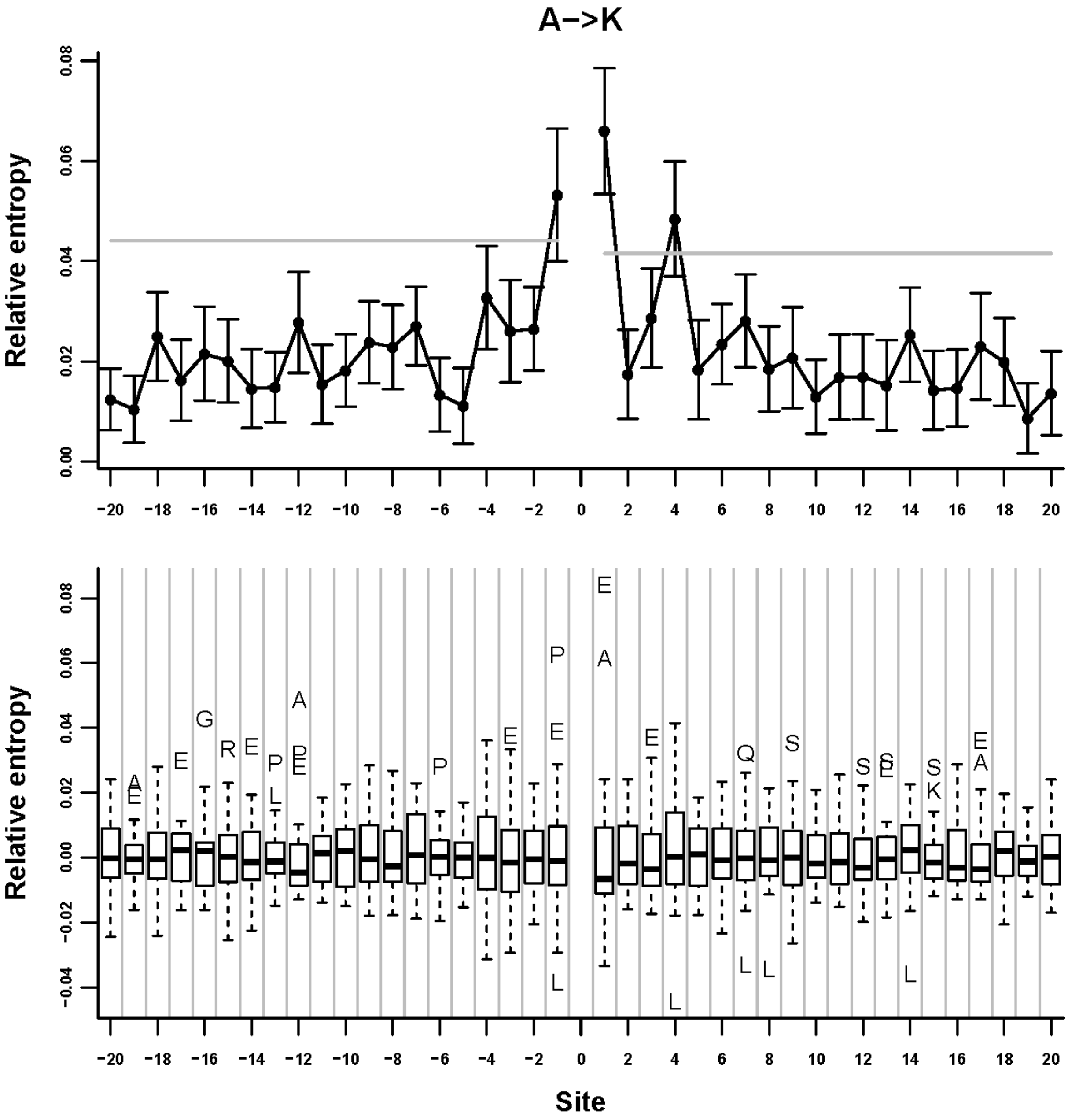
A−>K
Relative entropy
Relative entropy
Site

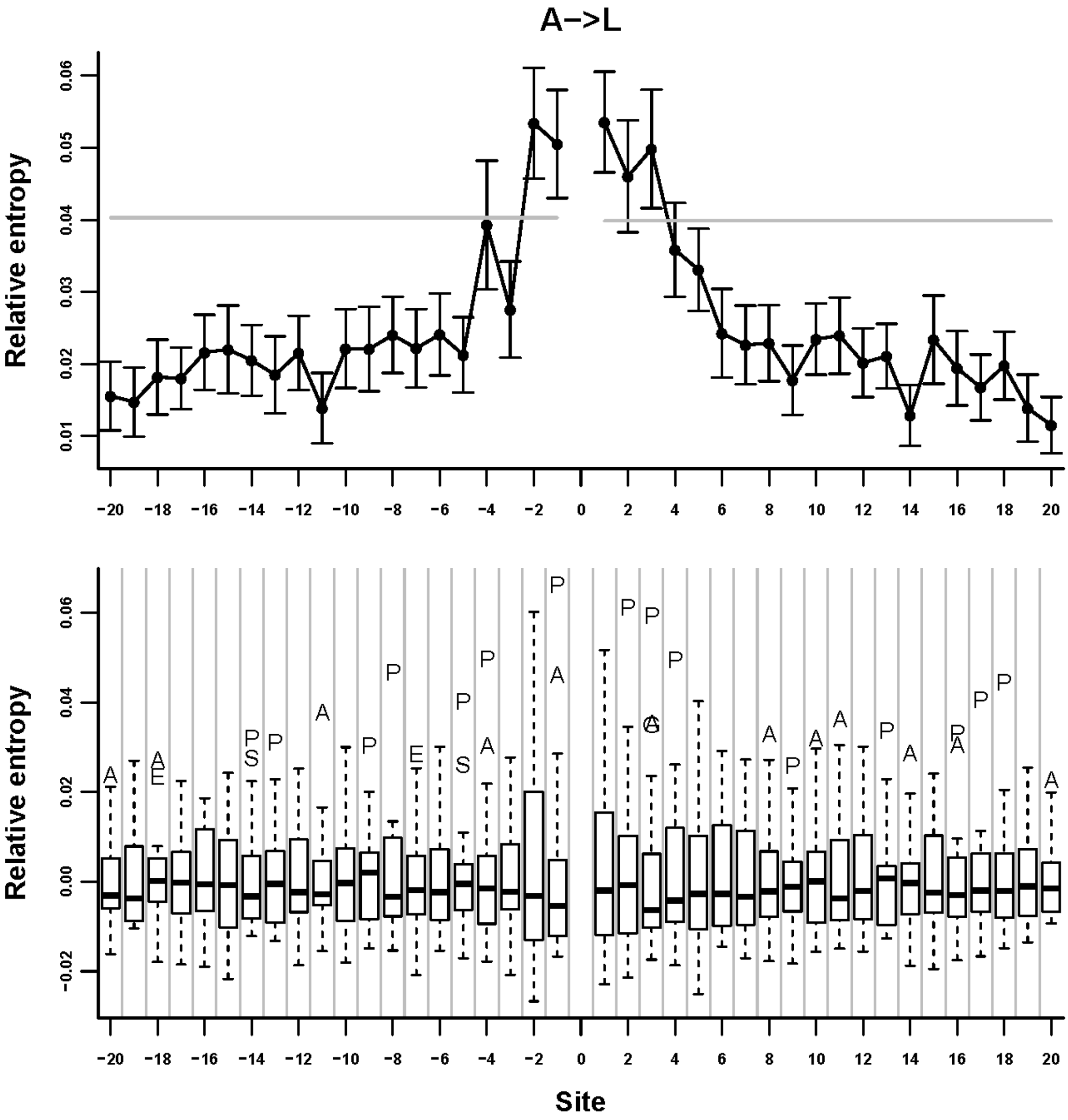
A−>L
Relative entropy
Relative entropy
Site

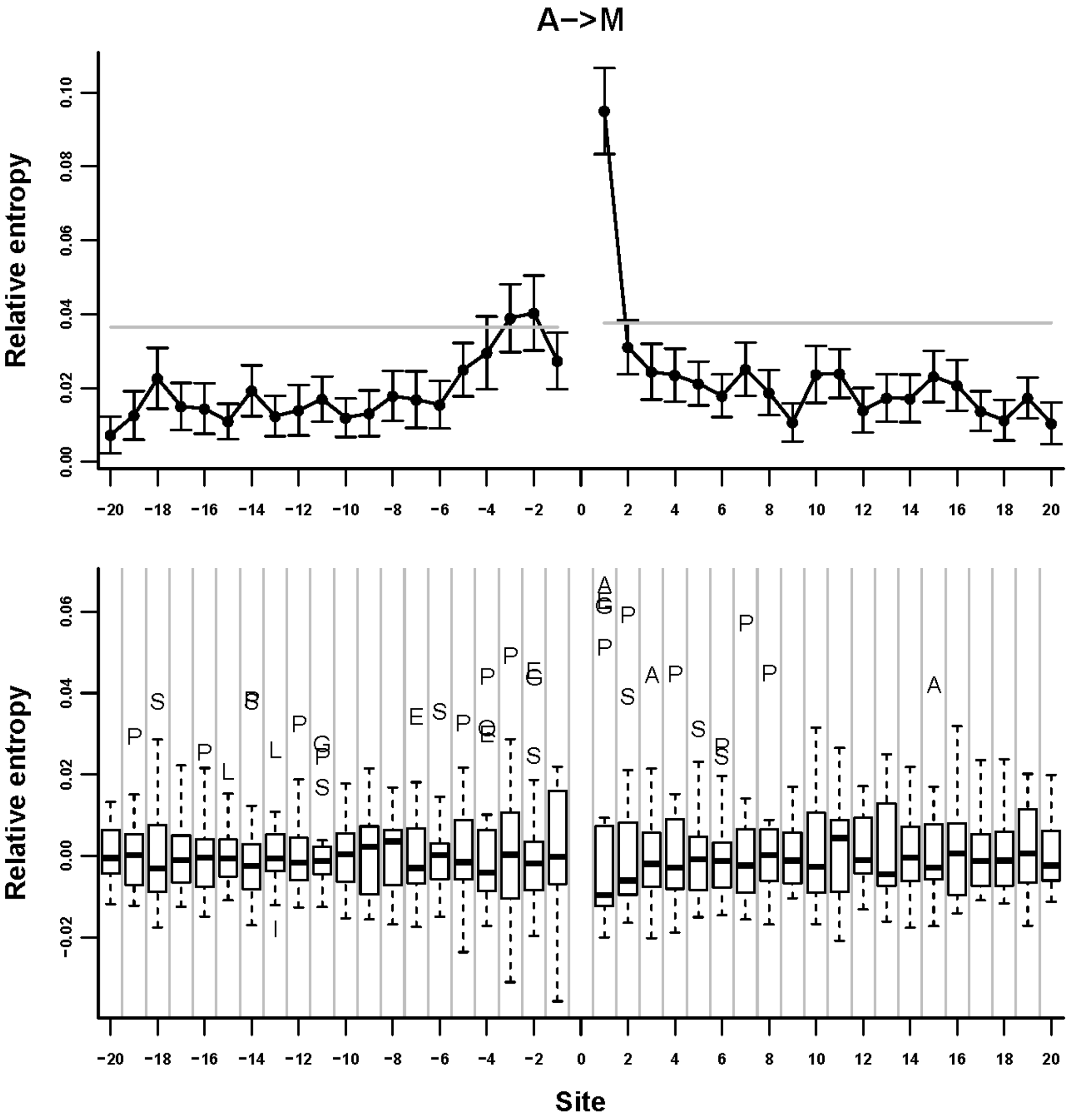
A−>M
Relative entropy
Relative entropy
Site

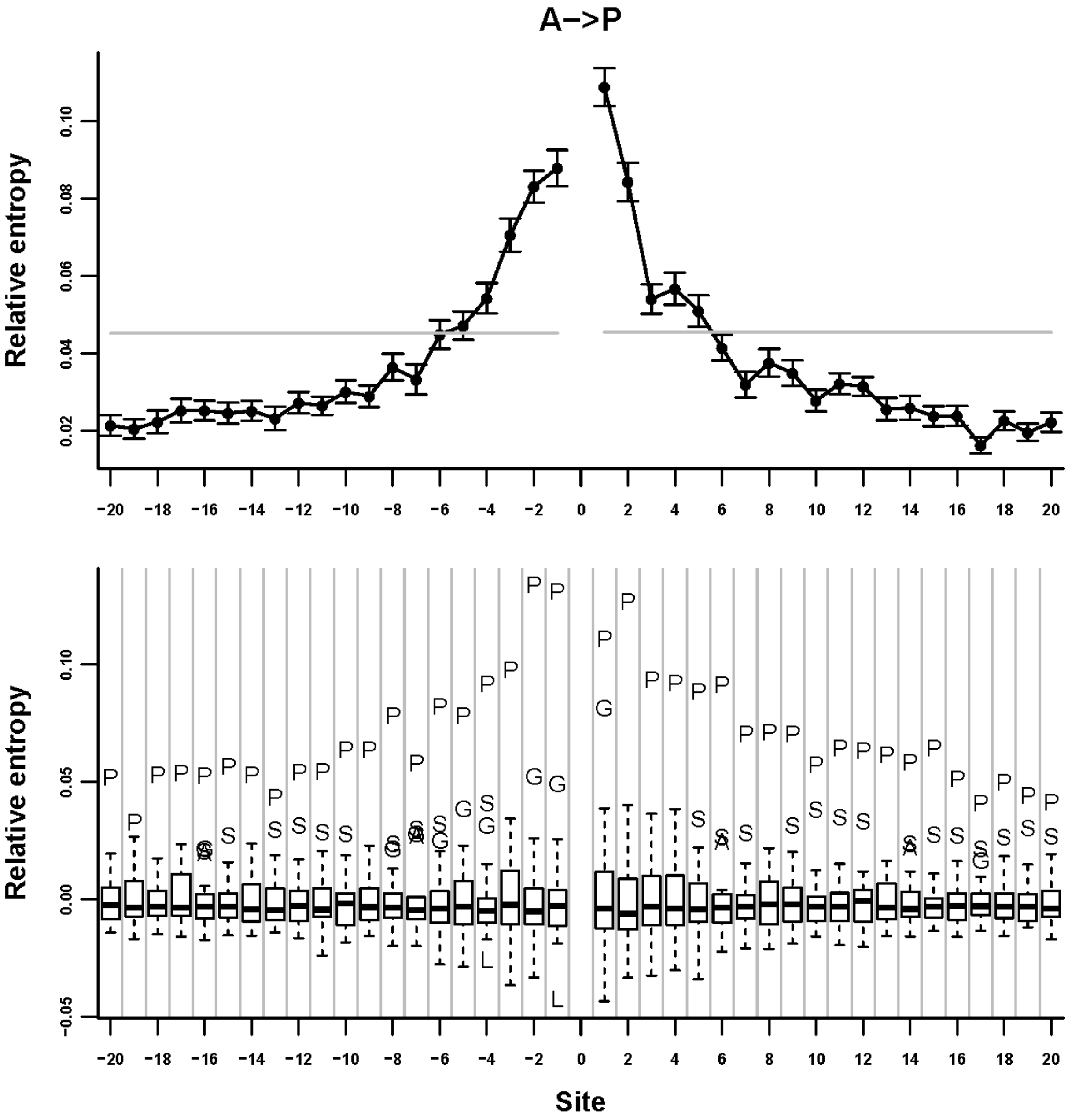
A−>P
Relative entropy
0.02
0.04
0.06
0.08
0.10
−20
−18
−16
−14
−12
−10
−8
−6
−4
−2
0
2
4
6
8
10
12
14
16
18
20
Relative entropy
−0.05
0.00
0.05
0.10
Site

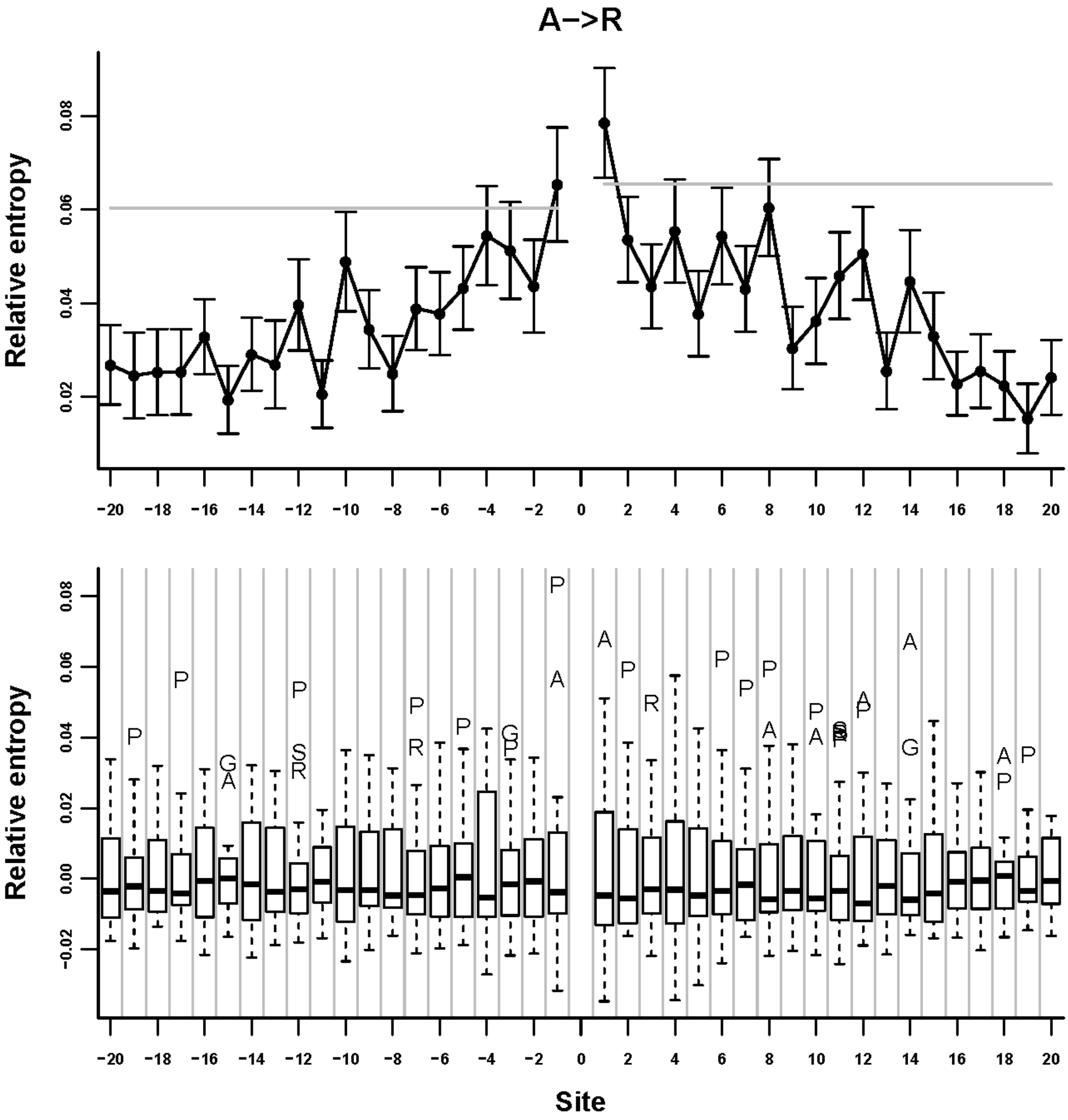
A−>R
Relative entropy
0.02
0.04
0.06
0.08
−20 −18 −16 −14 −12 −10 −8 −6 −4 −2 0 2 4 6 8 10 12 14 16 18 20
Relative entropy
-0.02
0.00
0.02
0.04
0.06
0.08
Site

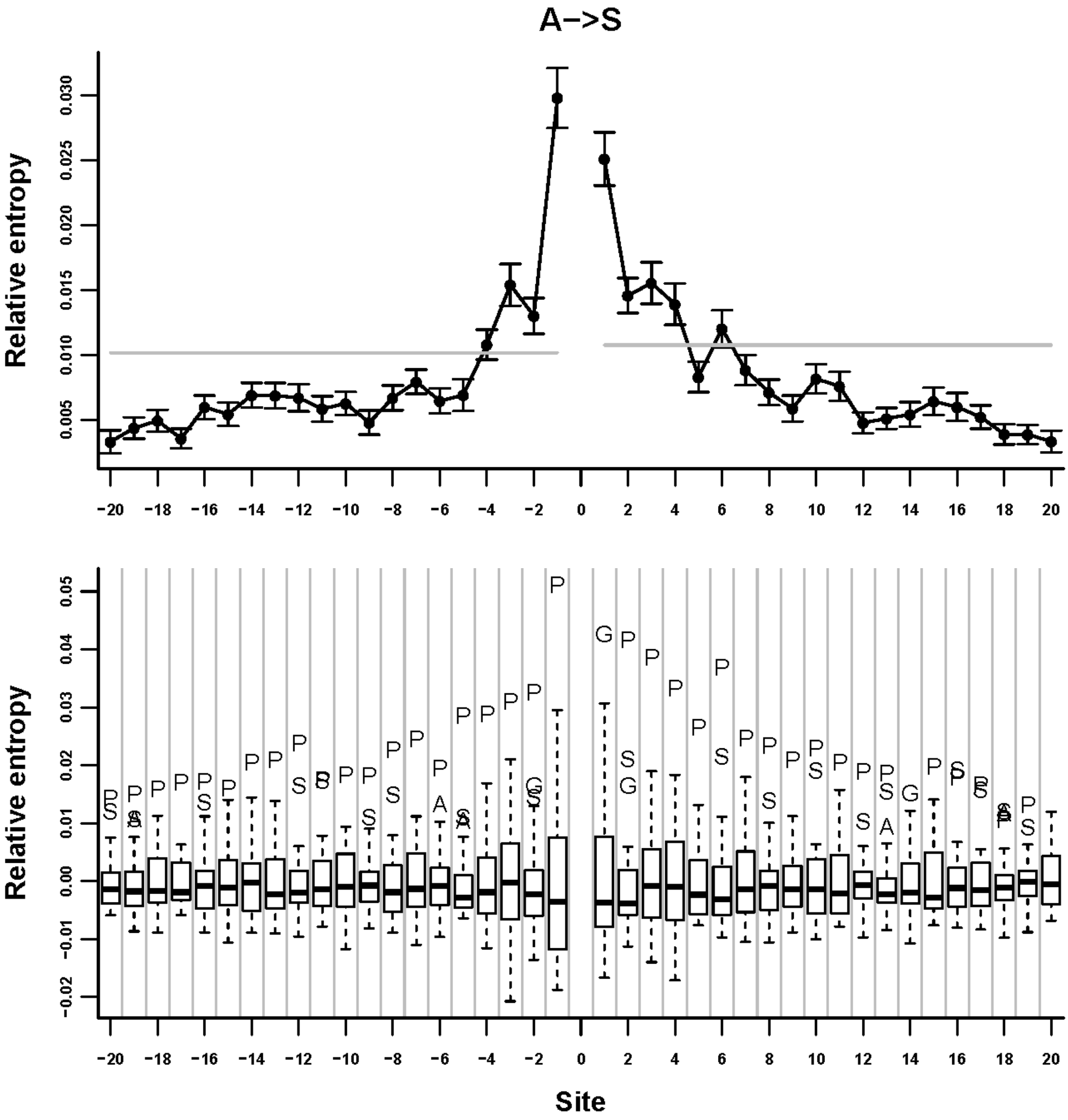
A->S
Relative entropy
Relative entropy
Site

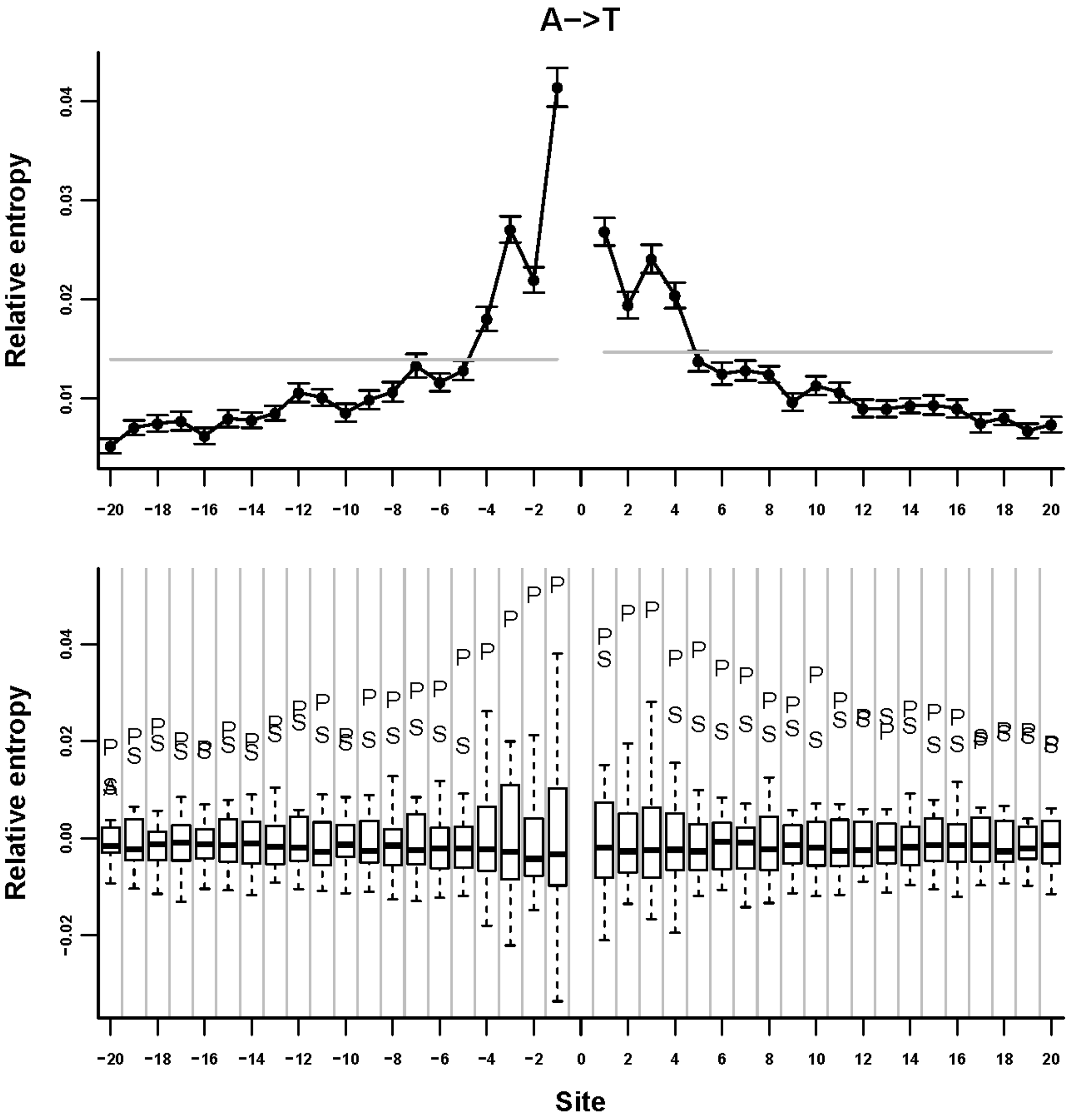
A−>T
Relative entropy
Relative entropy
Site

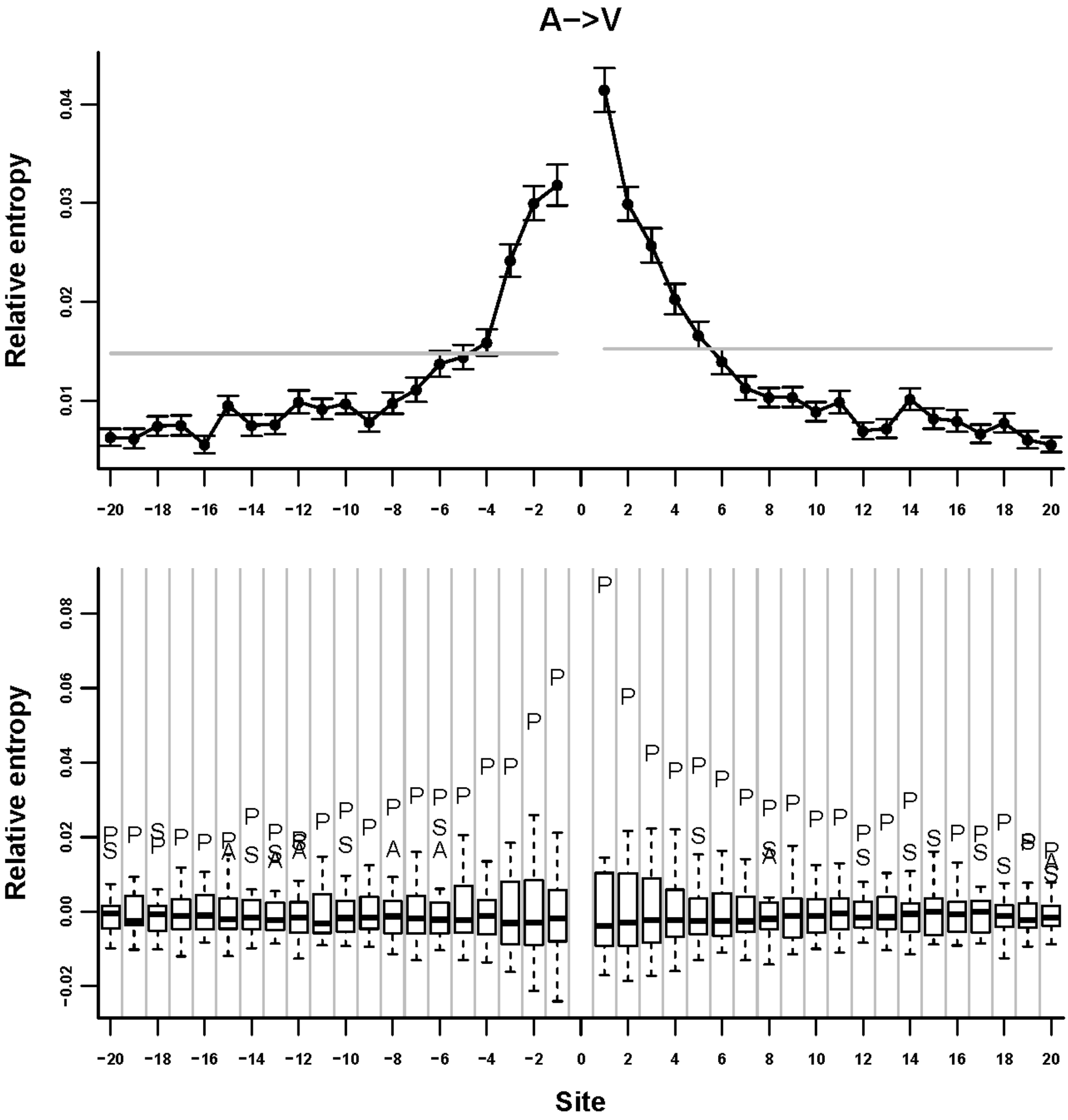
A−>V
Relative entropy
Relative entropy
Site

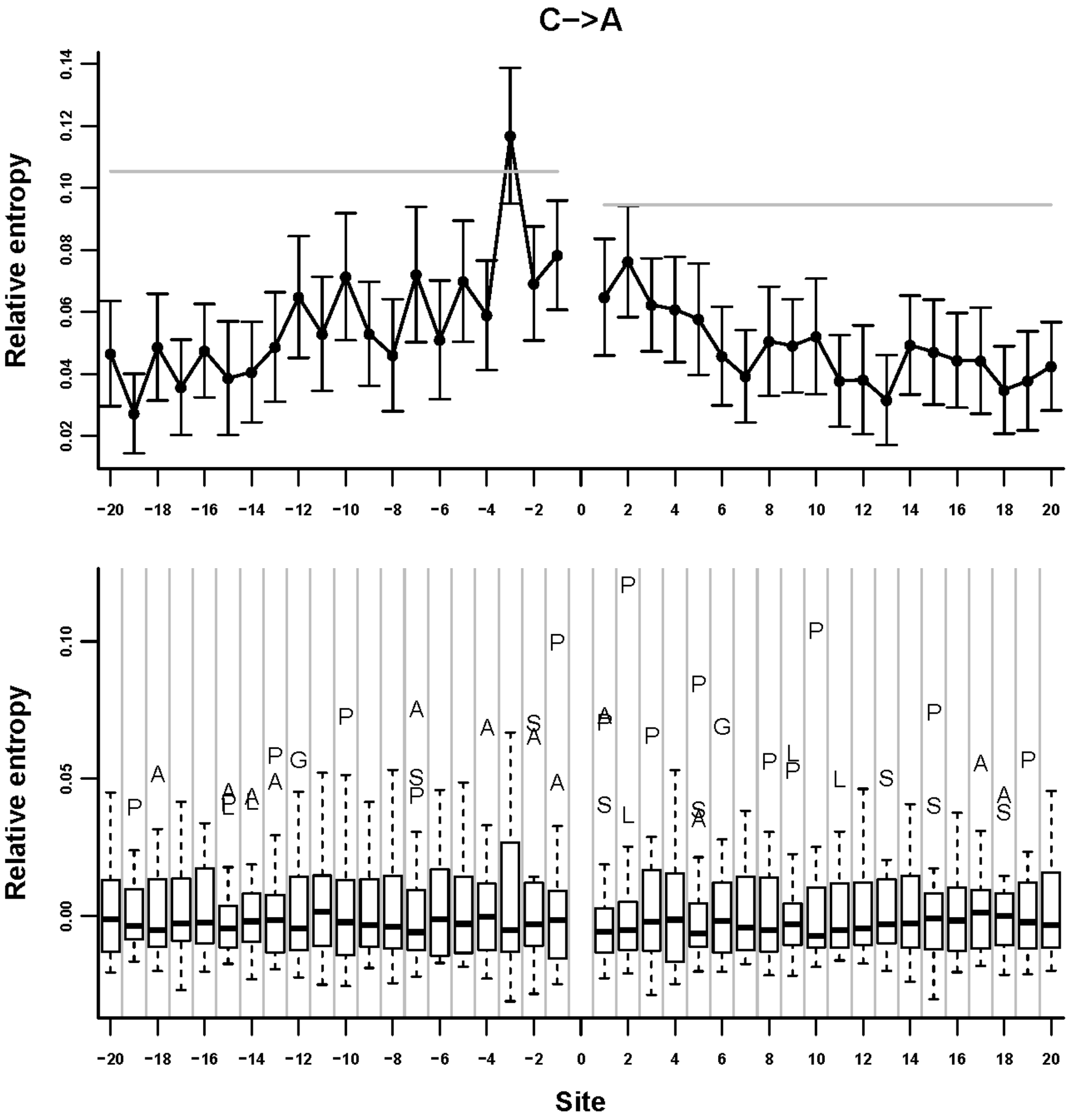
C−>A
Relative entropy
0.02
0.04
0.06
0.08
0.10
0.12
0.14
−20 −18 −16 −14 −12 −10 −8 −6 −4 −2 0 2 4 6 8 10 12 14 16 18 20
Relative entropy
0.00
0.05
0.10
Site

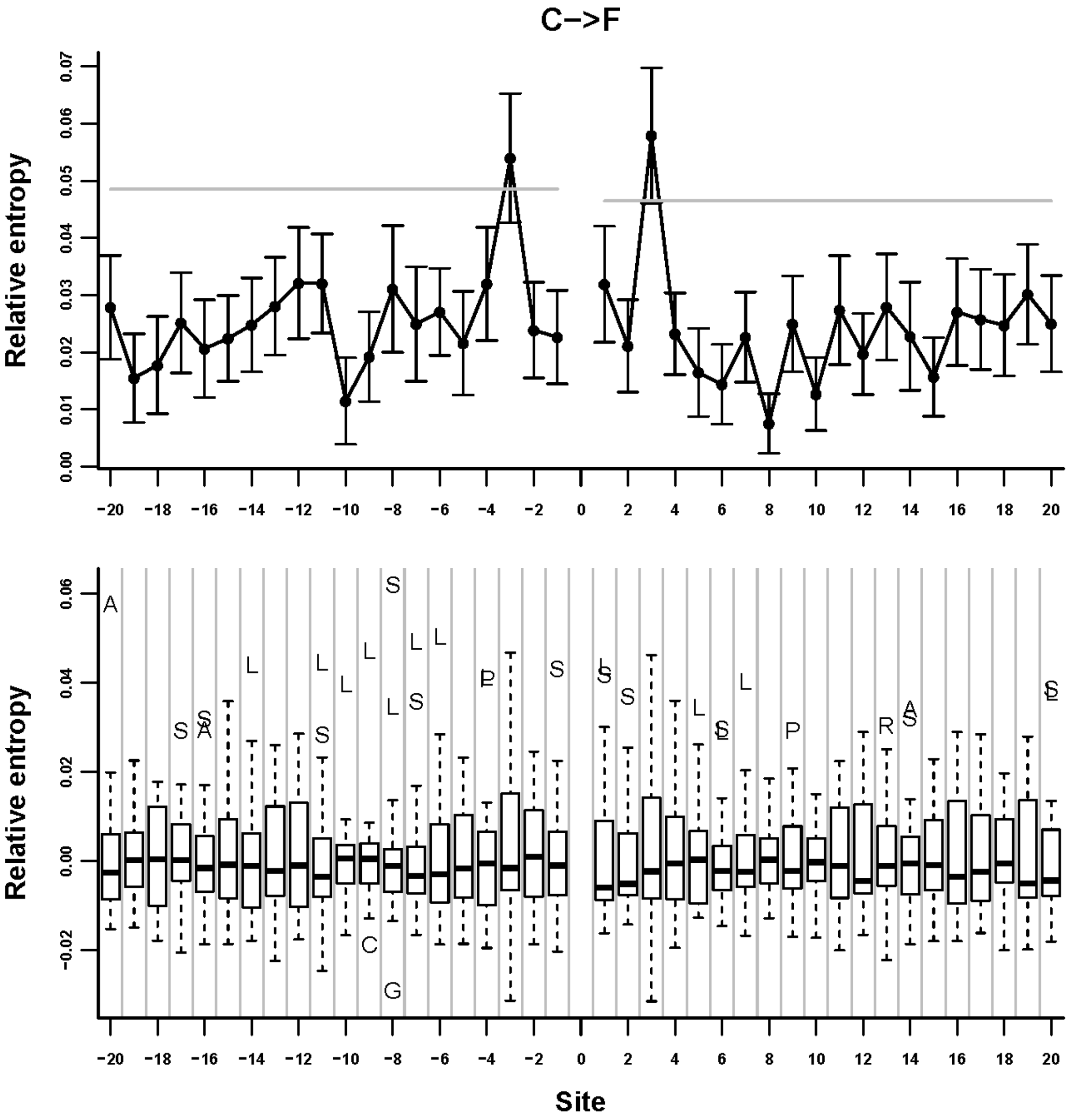
C−>F
Relative entropy
Relative entropy
Site

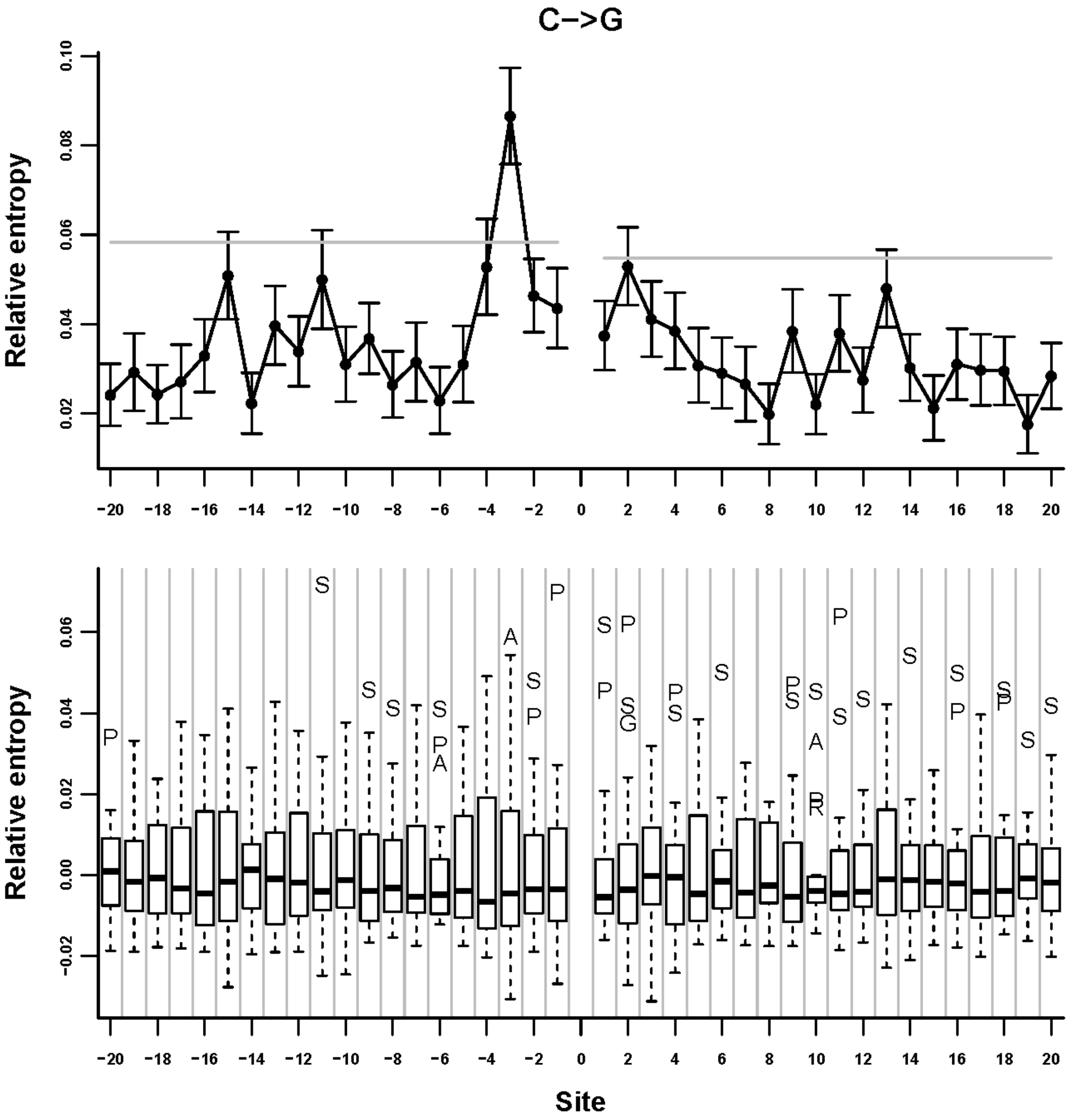
C−>G
Relative entropy
Relative entropy
Site

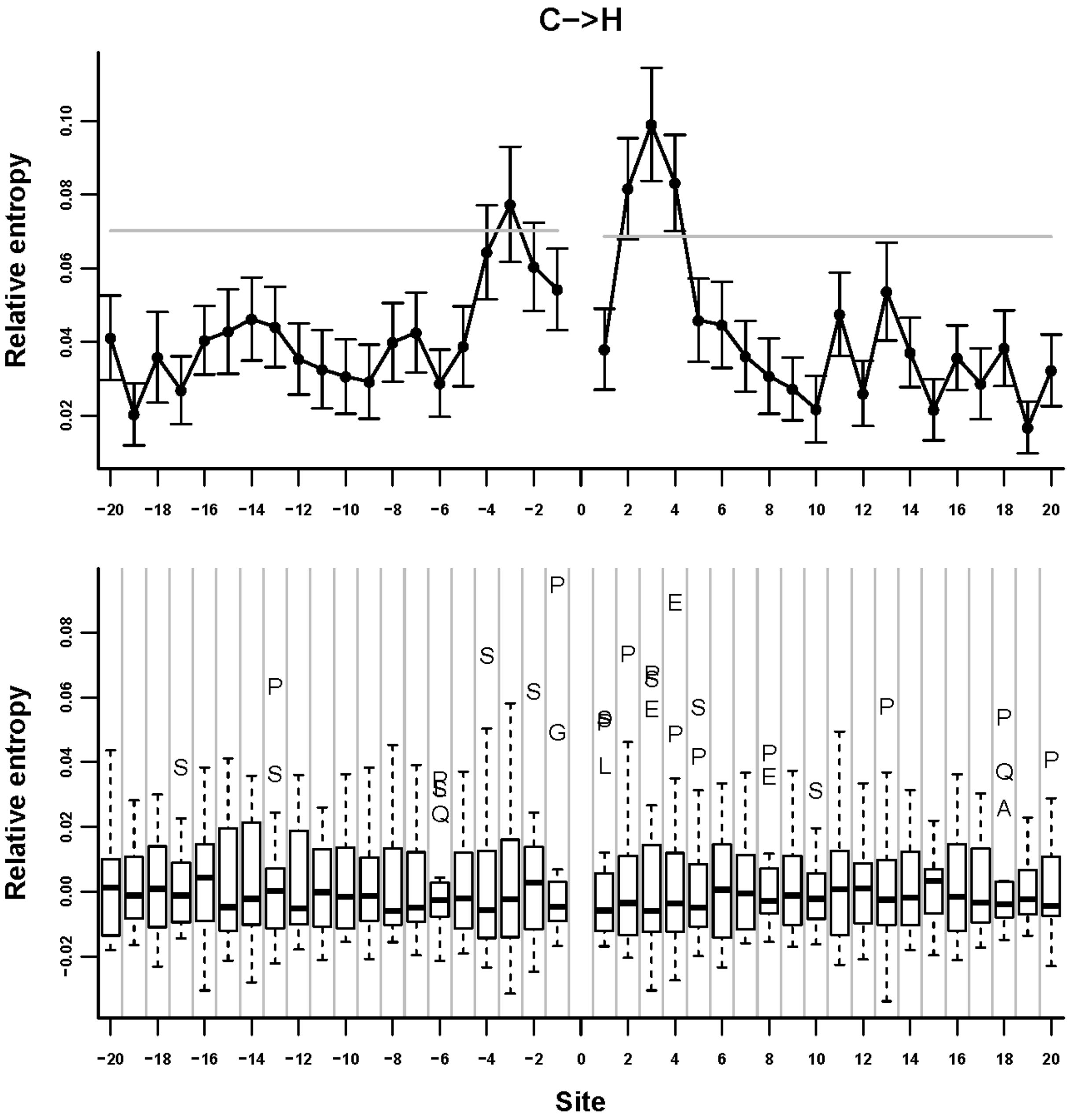
C−>H
Relative entropy
Relative entropy
Site

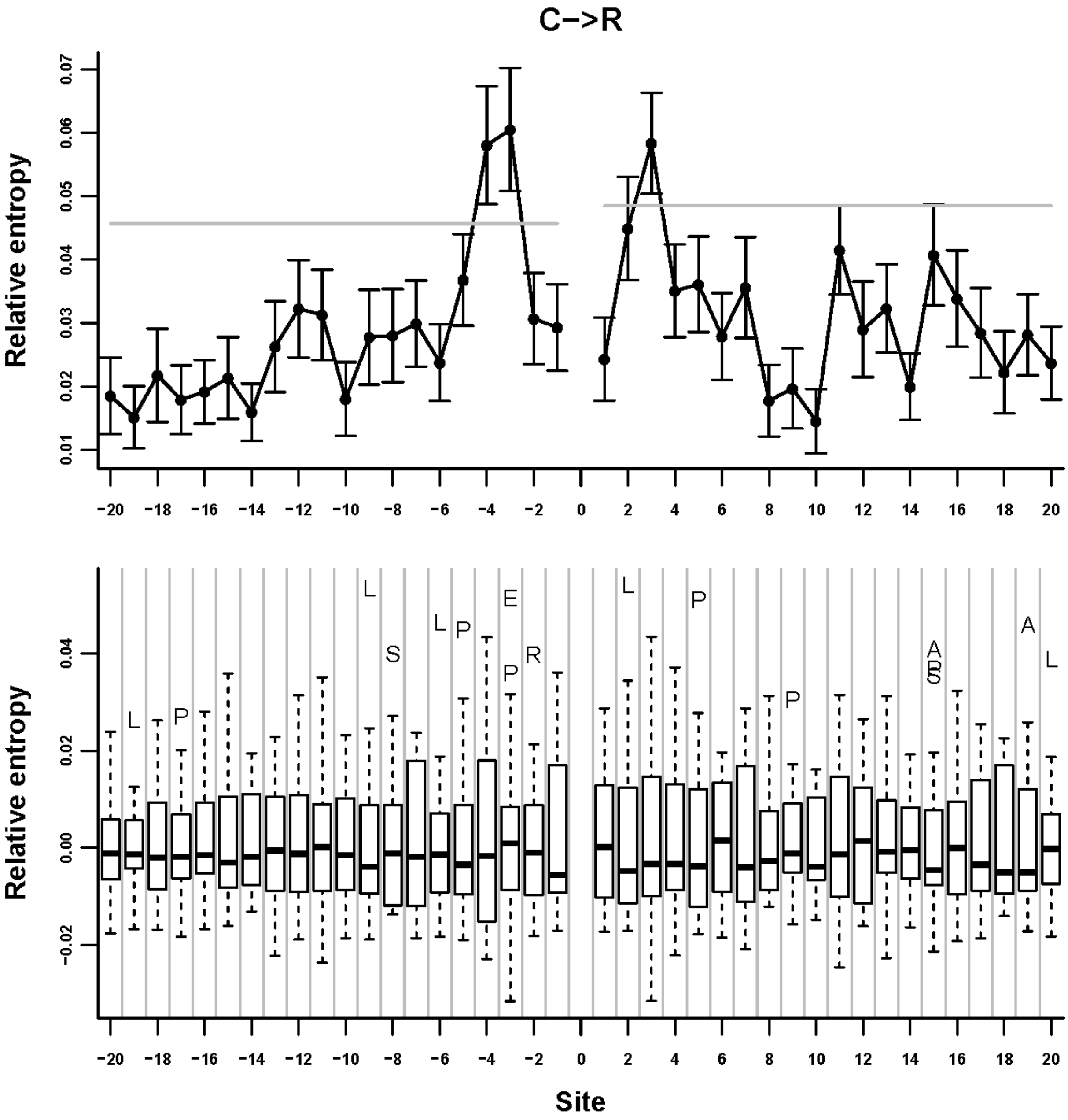
C−>R
Relative entropy
Relative entropy
Site
L
P
L
S
L
P
E
P
R
L
P
P
A
P
S
A
L

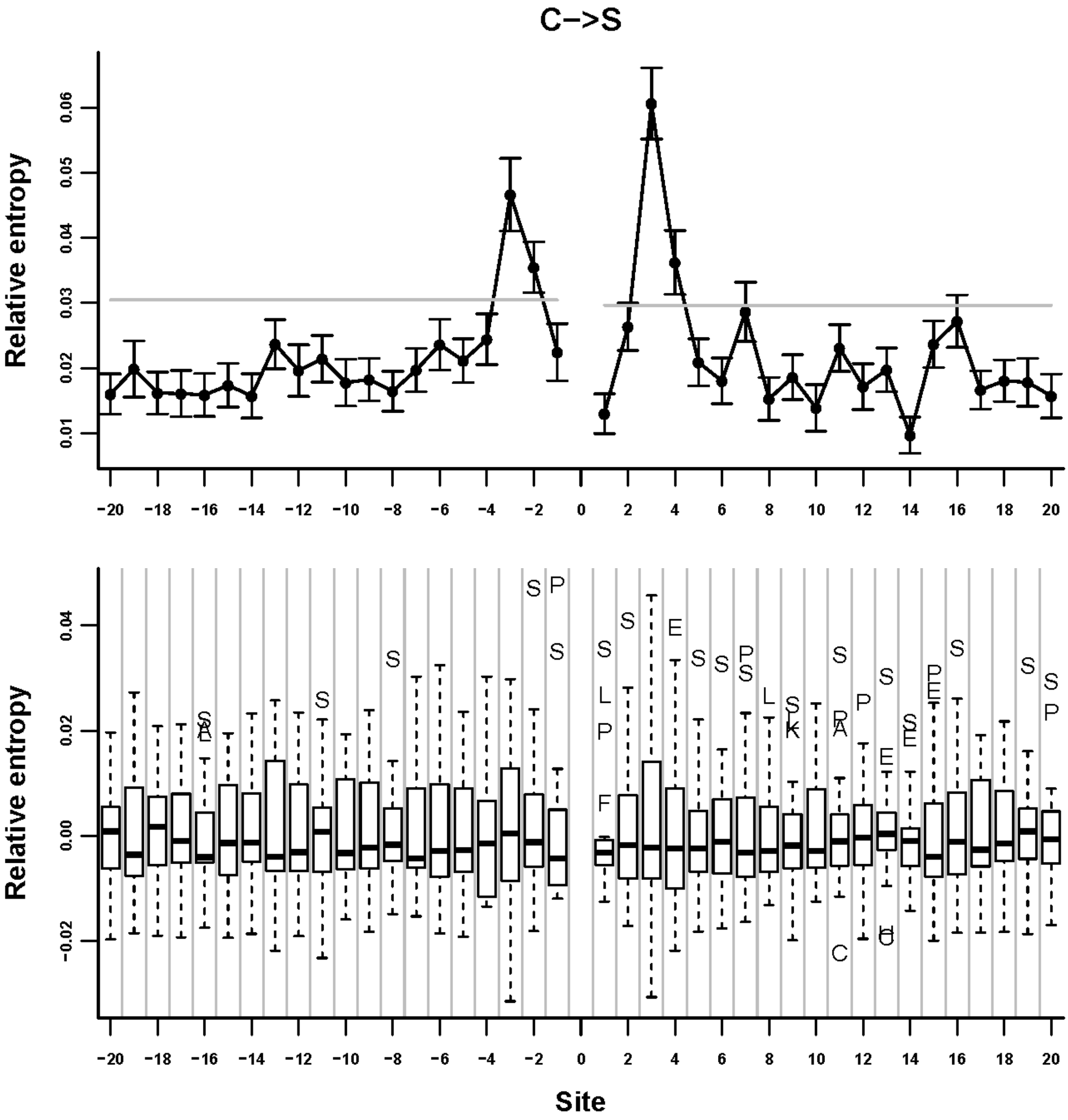
C−>S
Relative entropy
Relative entropy
Site

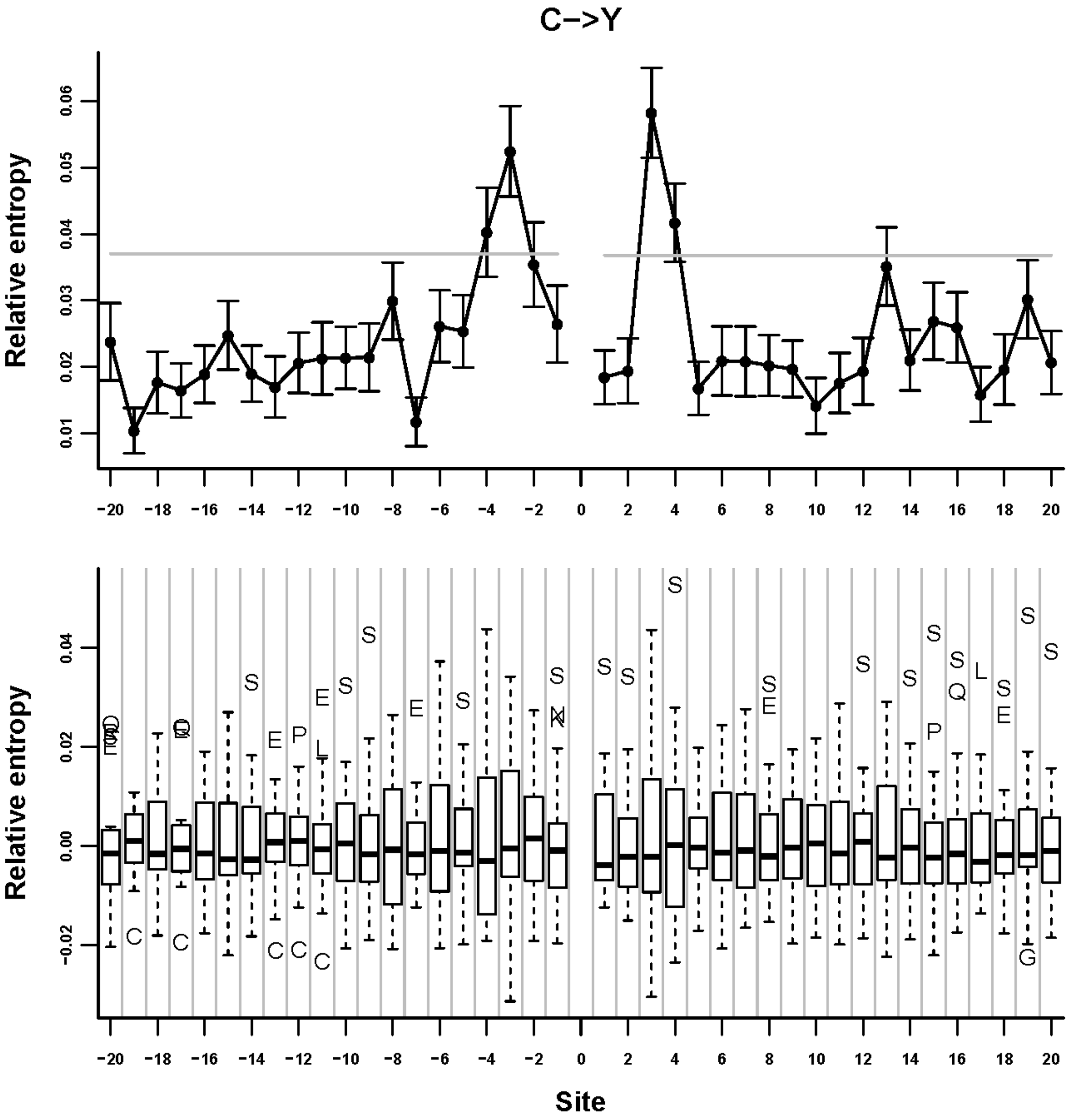
C−>Y
Relative entropy
0.01
0.02
0.03
0.04
0.05
0.06
−20 −18 −16 −14 −12 −10 −8 −6 −4 −2 0 2 4 6 8 10 12 14 16 18 20
Relative entropy
−0.02
0.00
0.02
0.04
−20 −18 −16 −14 −12 −10 −8 −6 −4 −2 0 2 4 6 8 10 12 14 16 18 20
Site

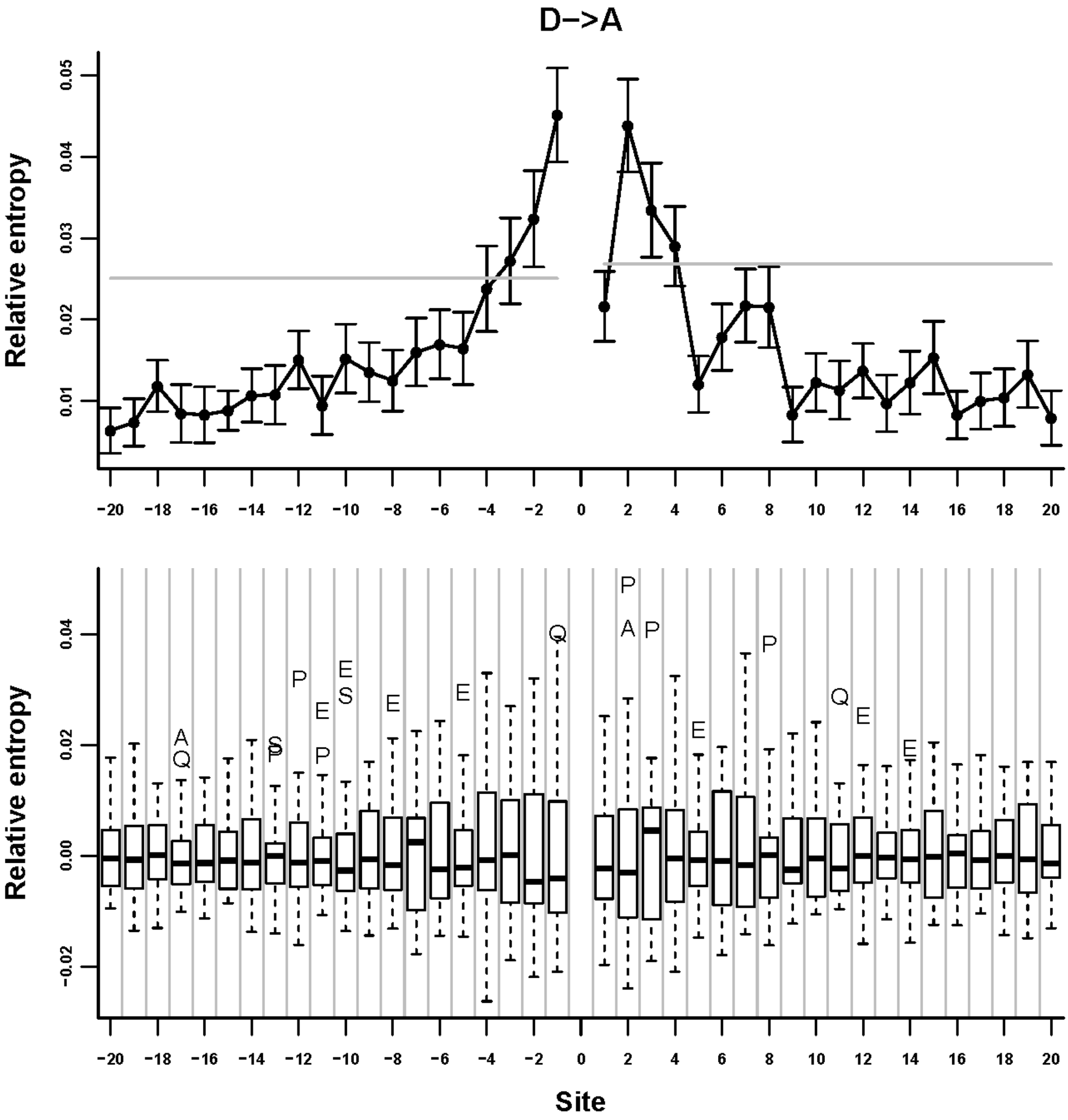

D->A
Relative entropy
0.01
0.02
0.03
0.04
0.05
-20 -18 -16 -14 -12 -10 -8 -6 -4 -2 0 2 4 6 8 10 12 14 16 18 20
Relative entropy
-0.02
0.00
0.02
0.04
A
Q
S
P
P
E
P
E
S
E
E
Q
P
A
P
E
P
Q
E
E
-20 -18 -16 -14 -12 -10 -8 -6 -4 -2 0 2 4 6 8 10 12 14 16 18 20
Site

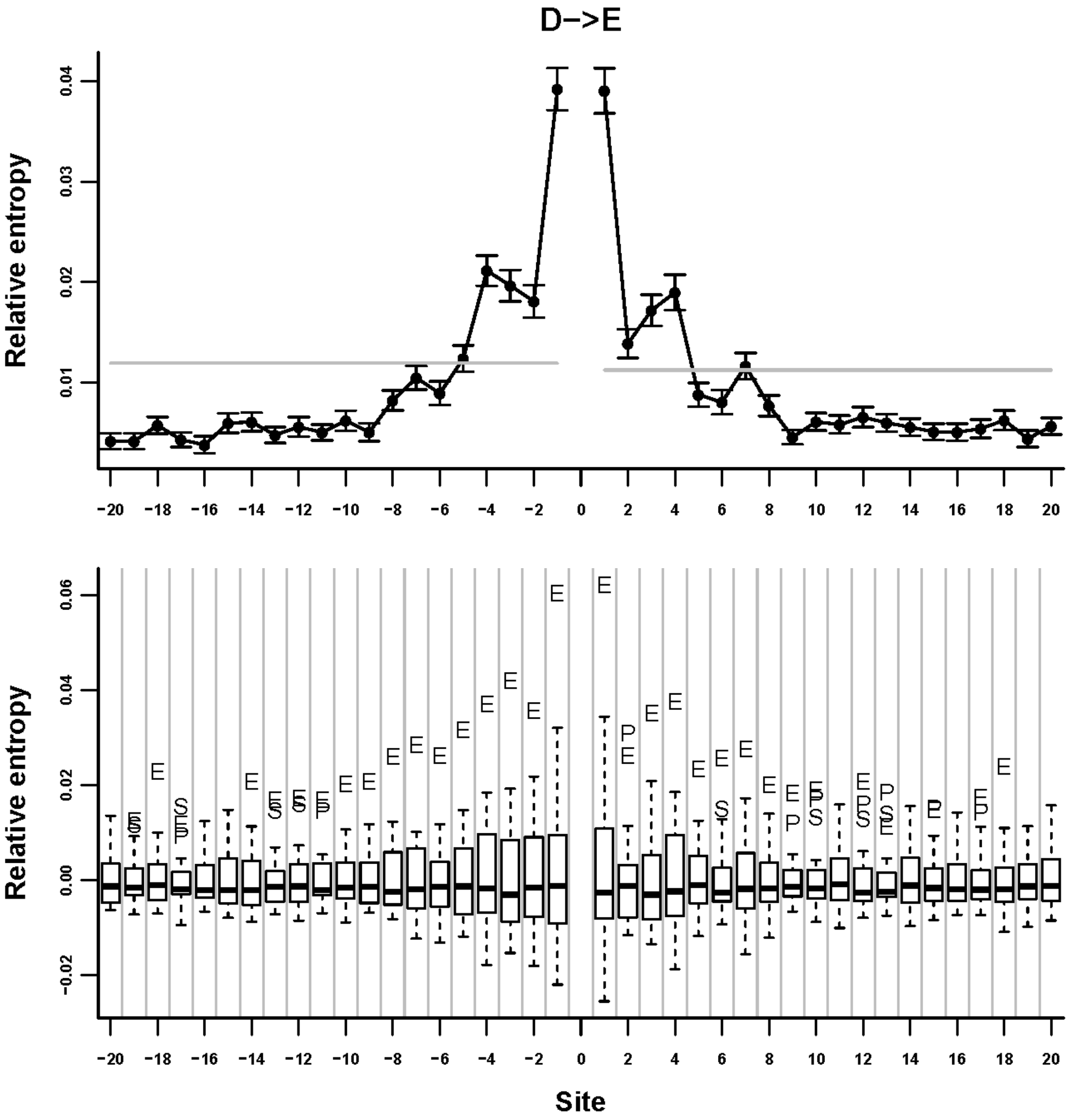
D−>E
Relative entropy
Relative entropy
Site

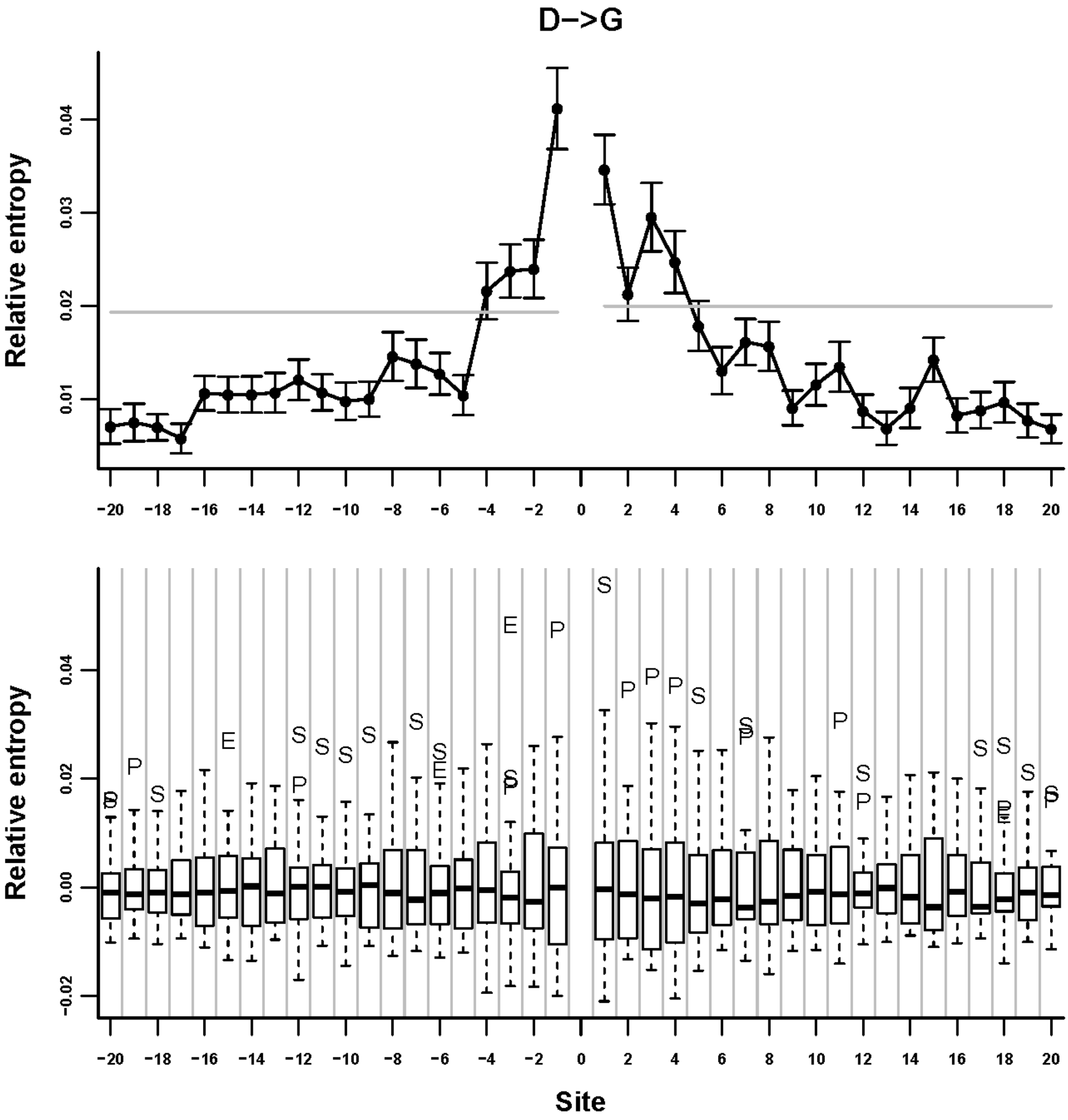
D->G
Relative entropy
Relative entropy
Site

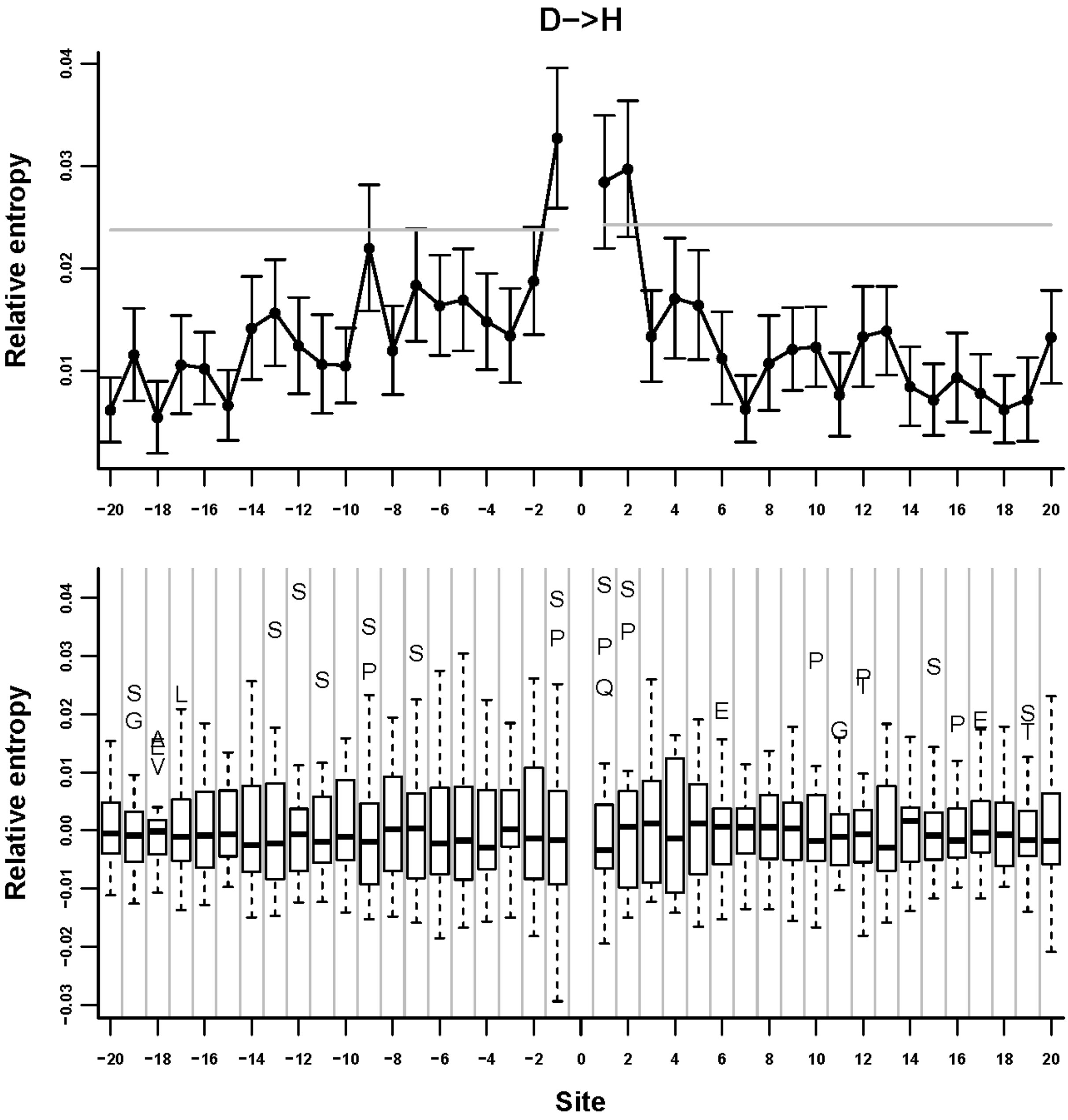
D−>H
Relative entropy
Relative entropy
Site
S
G
L
S
S
S
S
P
S
S
P
S
P
Q
S
P
E
P
G
P
T
S
P
E
S
T

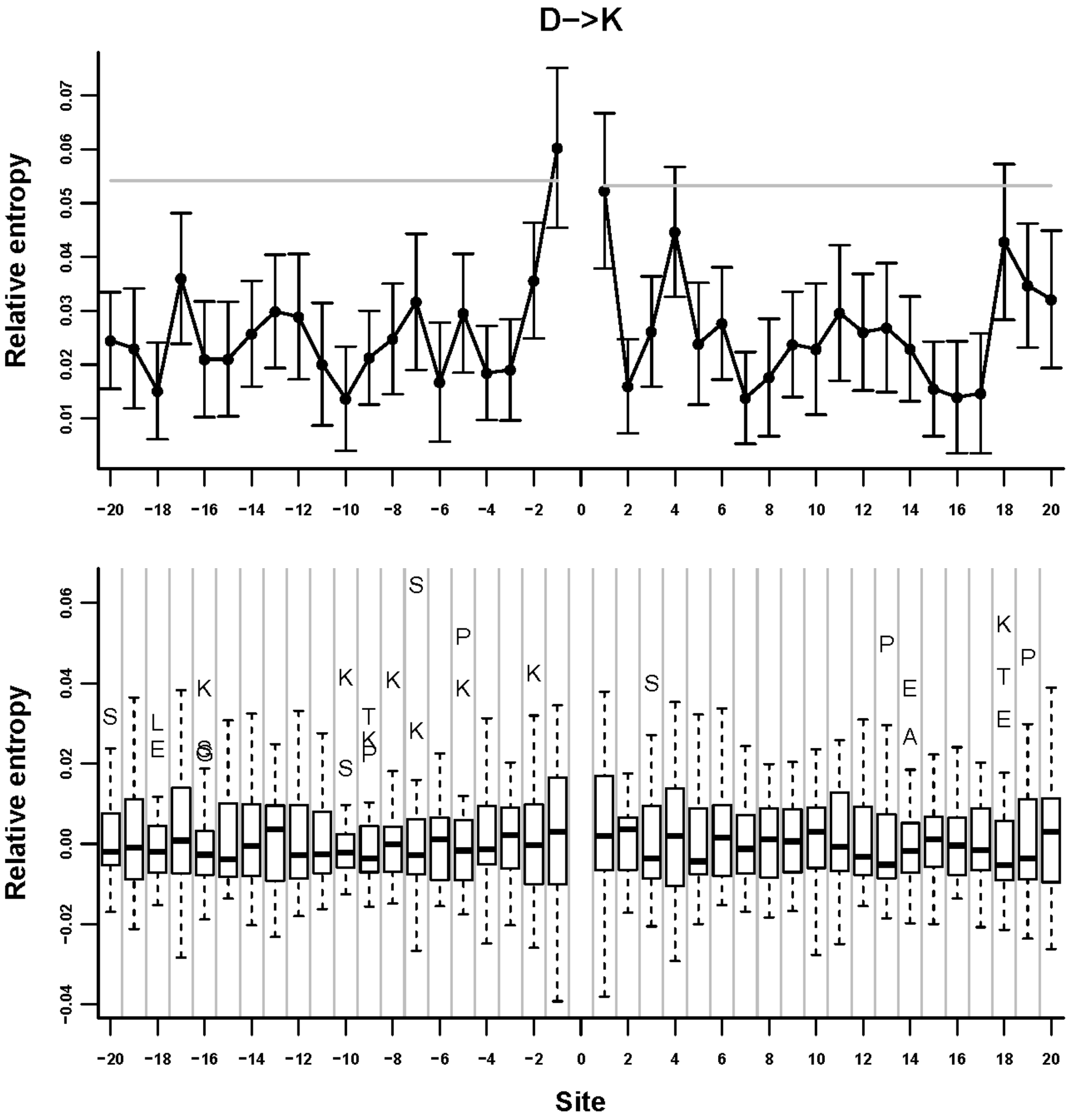
D->K
Relative entropy
Relative entropy
Site
S
L
E
K
S
G
K
S
T
K
P
K
S
K
P
K
K
S
P
E
A
K
T
E
P

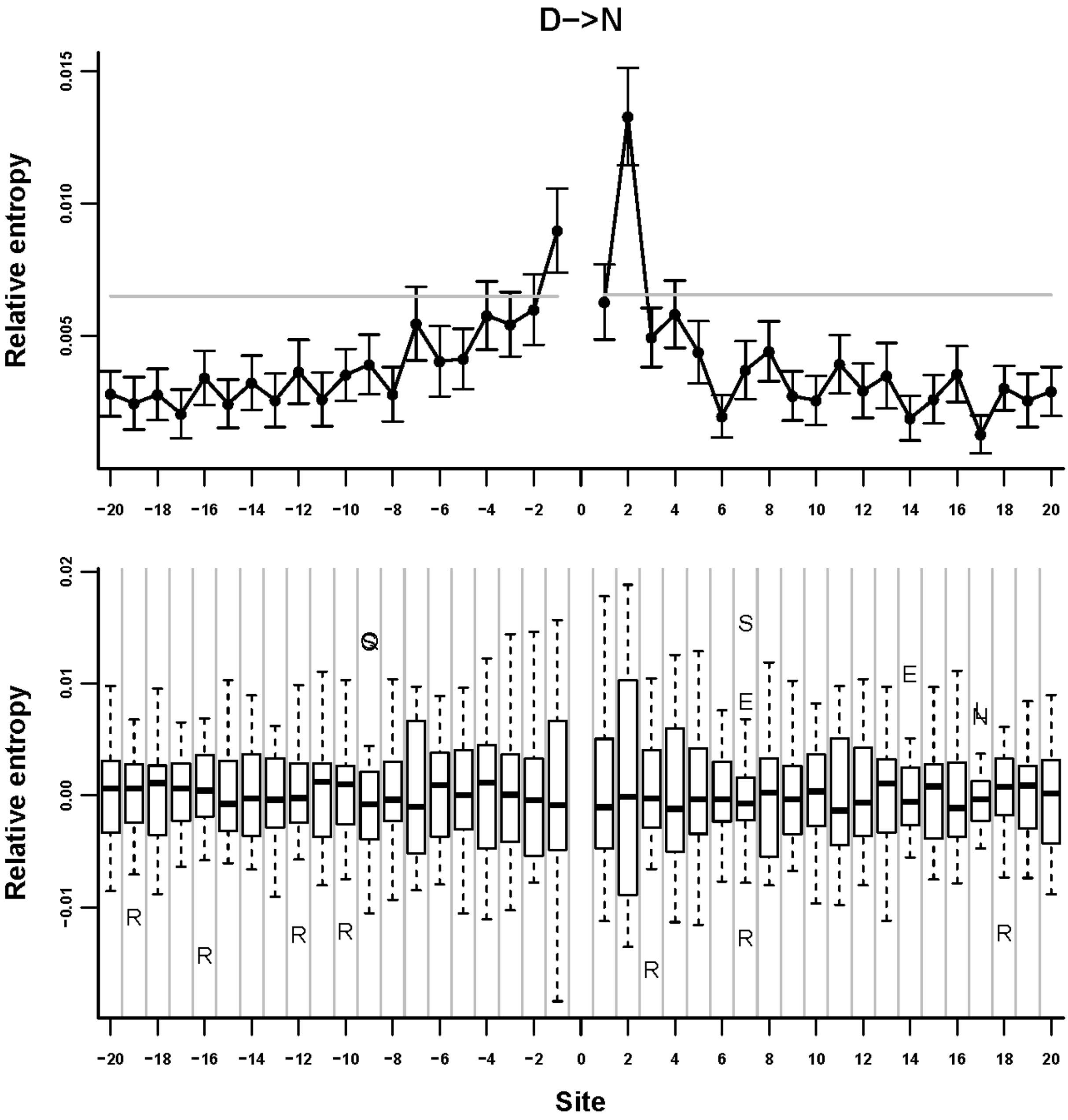
D−>N
Relative entropy
0.015
0.010
0.005
−20 −18 −16 −14 −12 −10 −8 −6 −4 −2 0 2 4 6 8 10 12 14 16 18 20
Relative entropy
0.02
0.01
0.00
-0.01
S
Q
E
E
N
R
R
R
R
R
R
R
R
−20 −18 −16 −14 −12 −10 −8 −6 −4 −2 0 2 4 6 8 10 12 14 16 18 20
Site

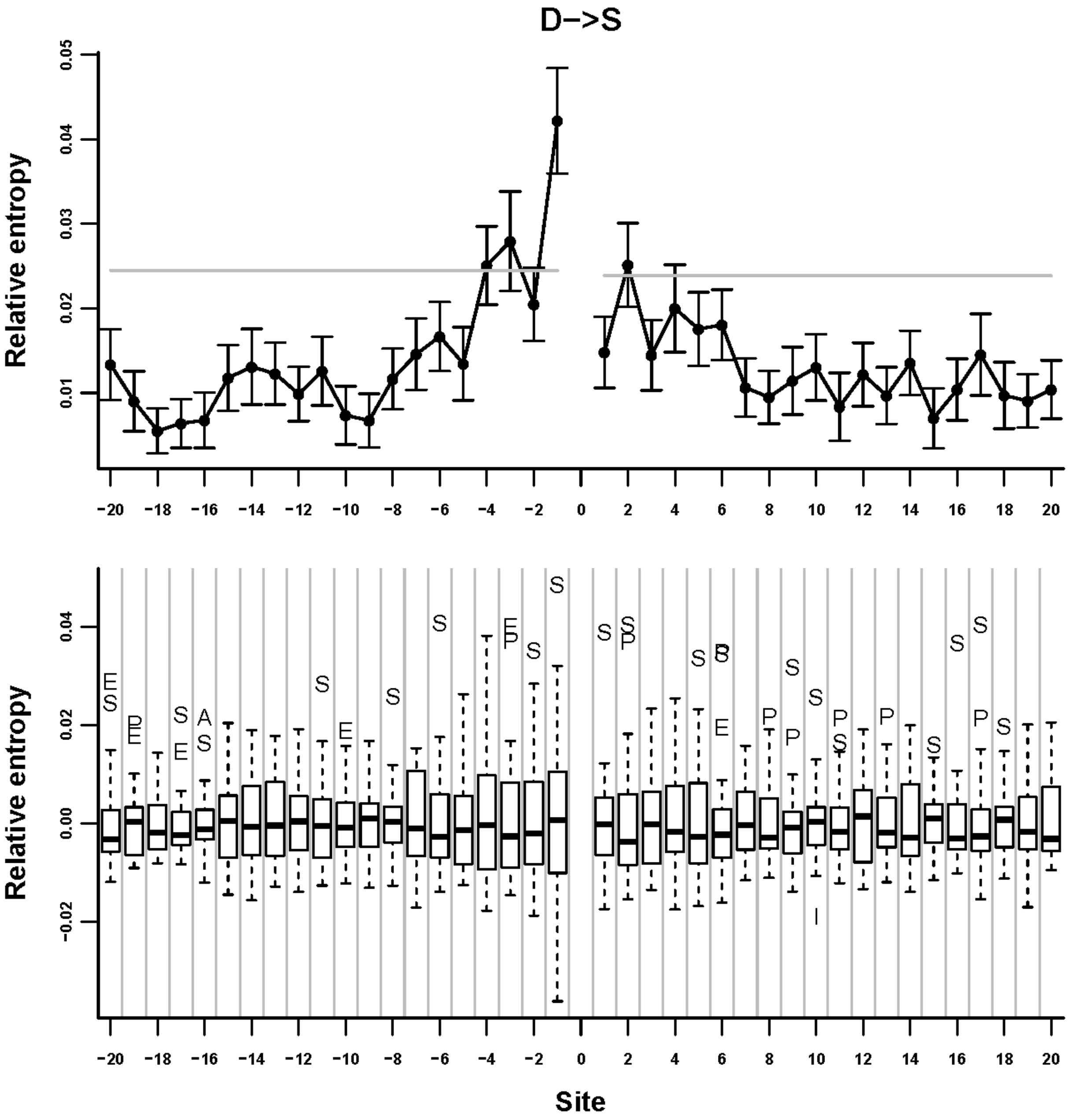
D->S
Relative entropy
Relative entropy
Site

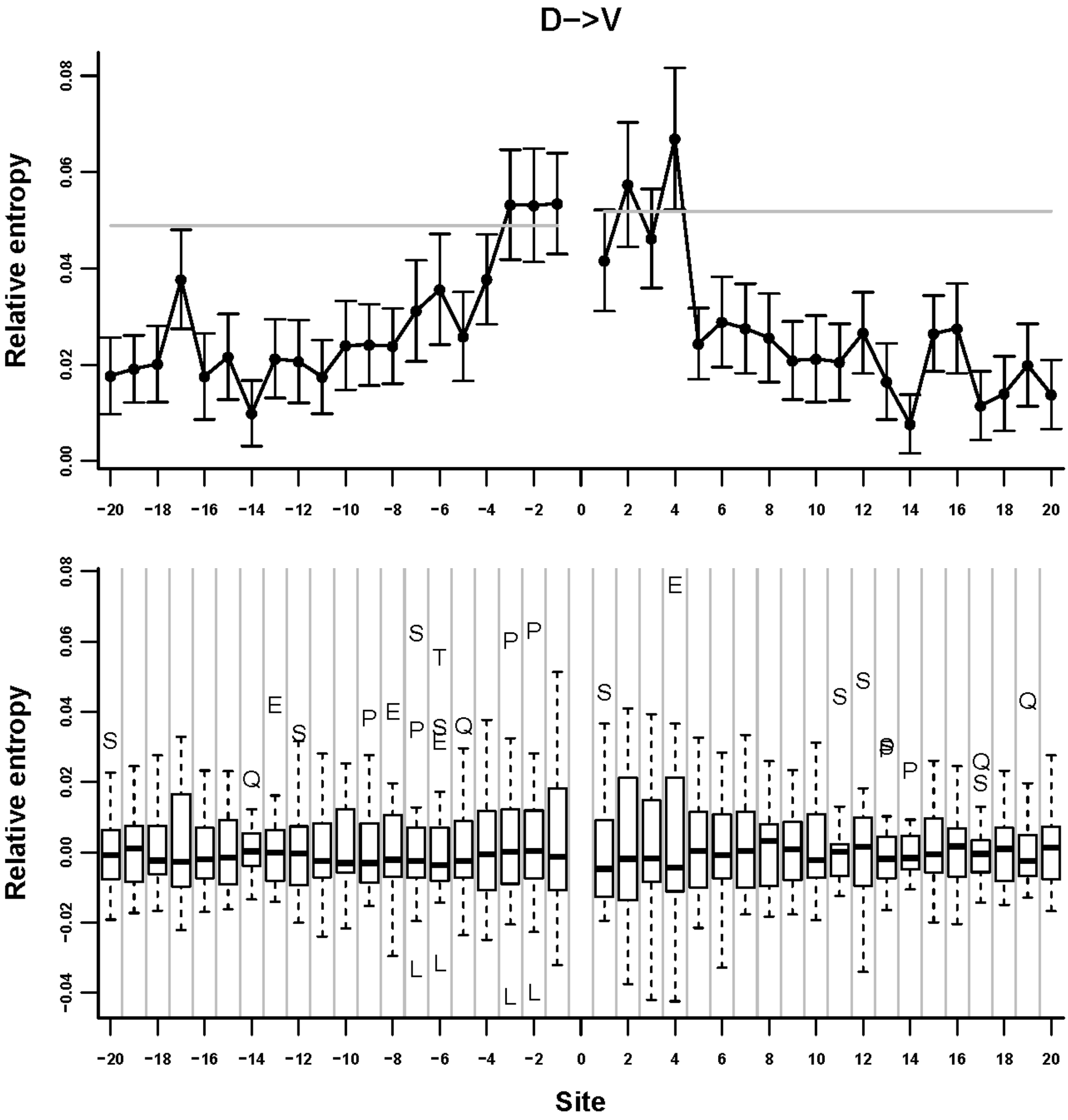
D->V
Relative entropy
Relative entropy
Site

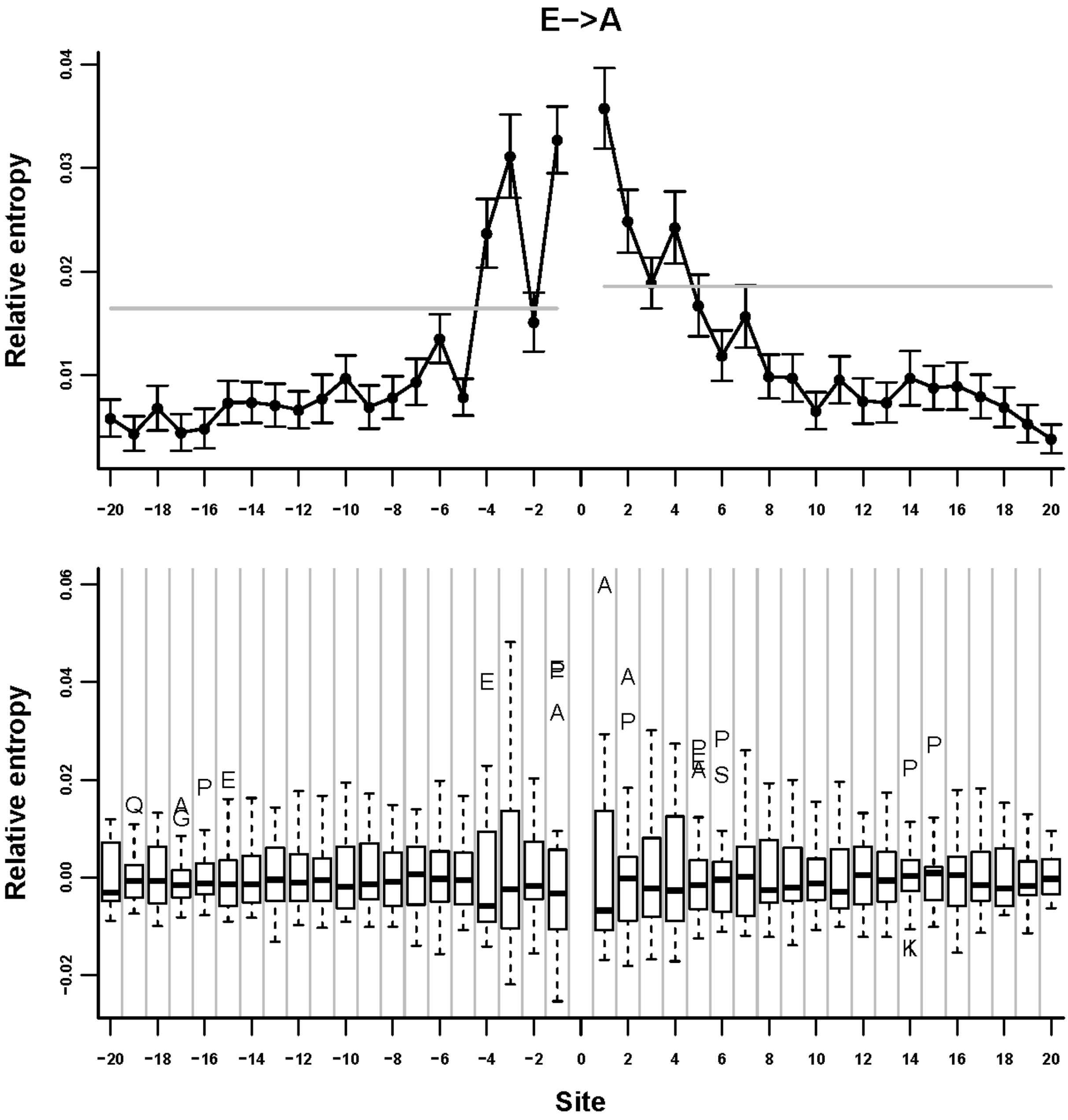
E−>A
Relative entropy
0.01
0.02
0.03
0.04
−20
−18
−16
−14
−12
−10
−8
−6
−4
−2
0
2
4
6
8
10
12
14
16
18
20
Relative entropy
−0.02
0.00
0.02
0.04
0.06
Site

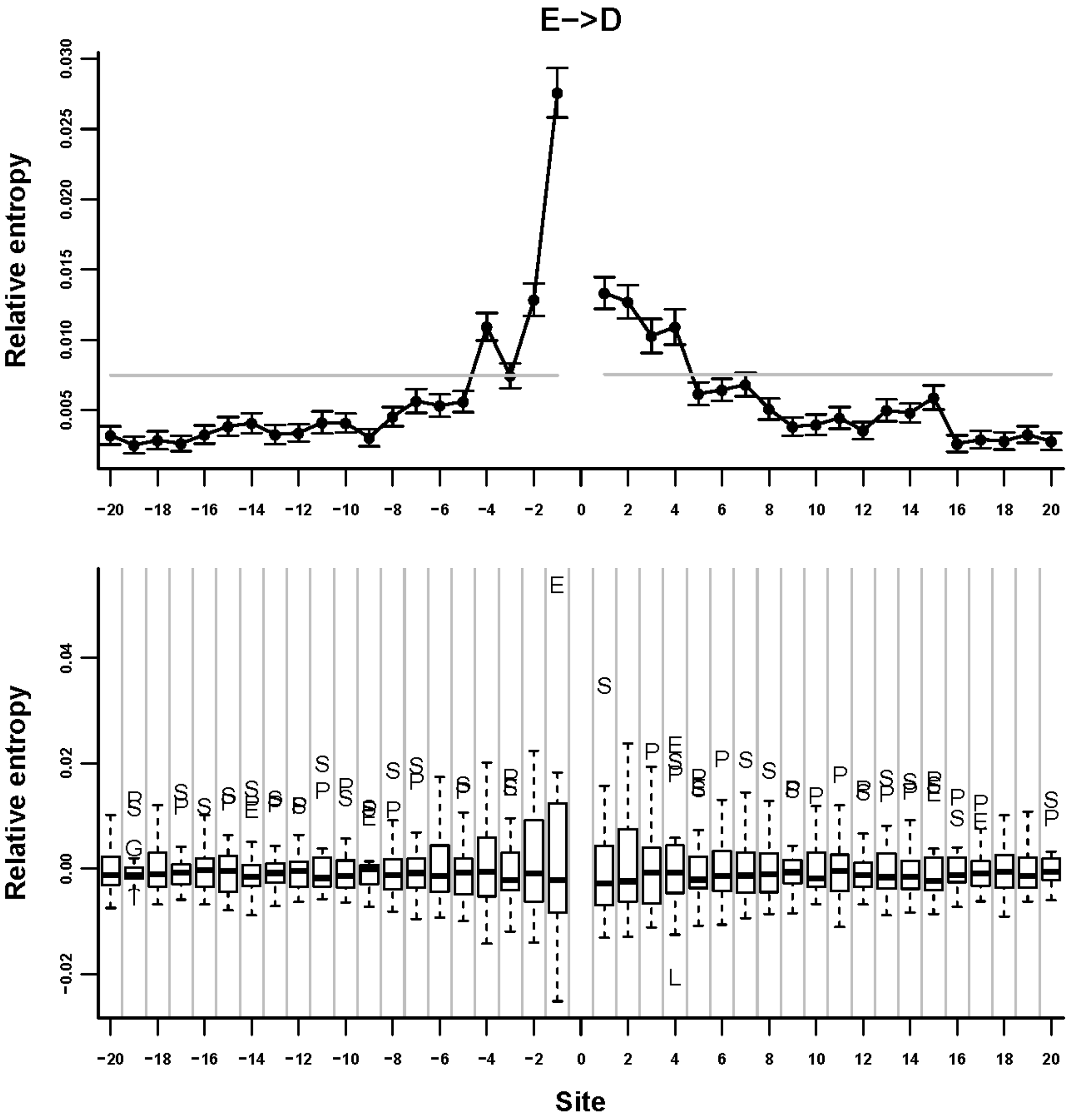
E−>D
Relative entropy
Relative entropy
Site

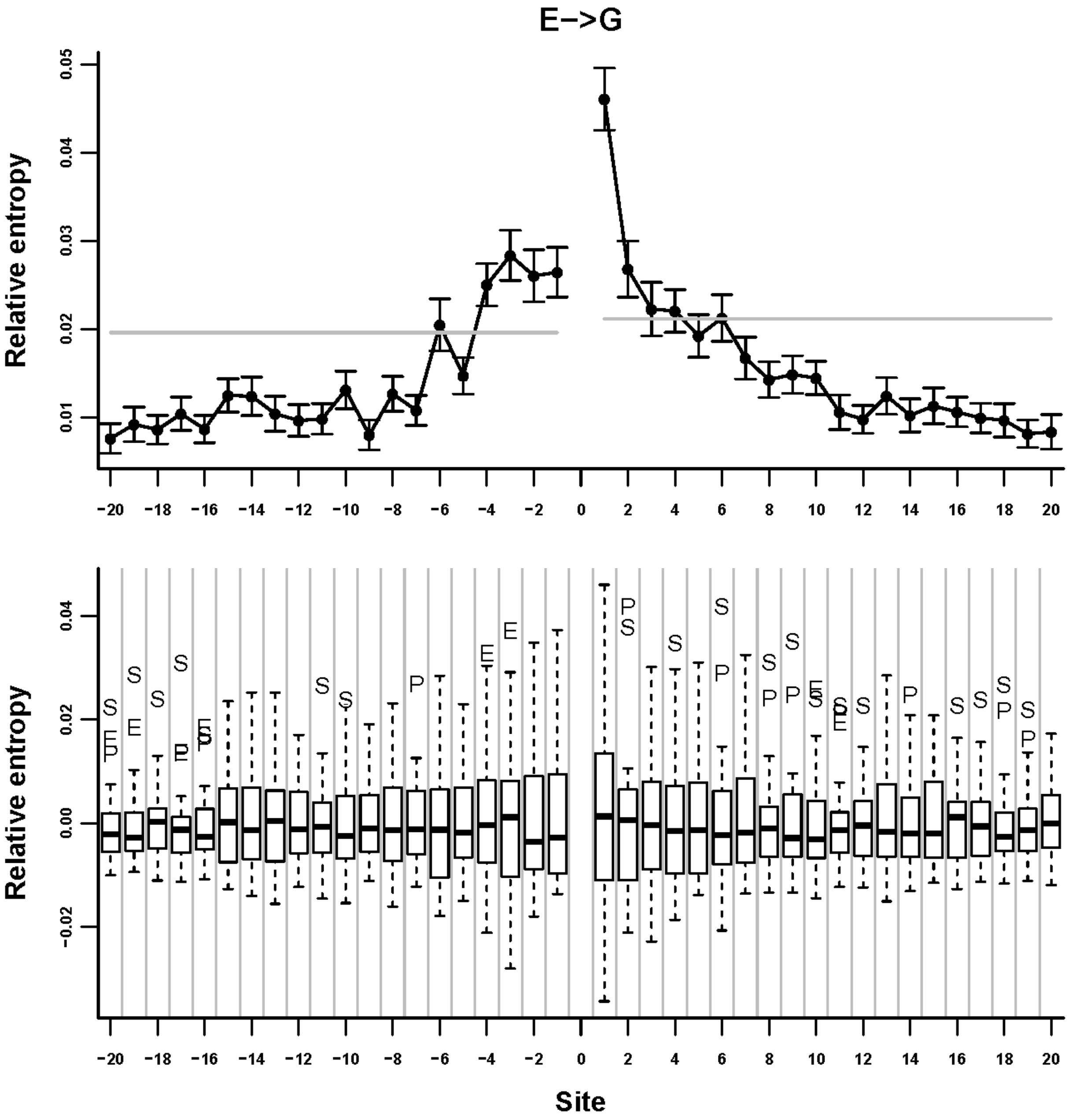
E−>G
Relative entropy
0.01
0.02
0.03
0.04
0.05
−20 −18 −16 −14 −12 −10 −8 −6 −4 −2 0 2 4 6 8 10 12 14 16 18 20
Relative entropy
−0.02
0.00
0.02
0.04
−20 −18 −16 −14 −12 −10 −8 −6 −4 −2 0 2 4 6 8 10 12 14 16 18 20
Site

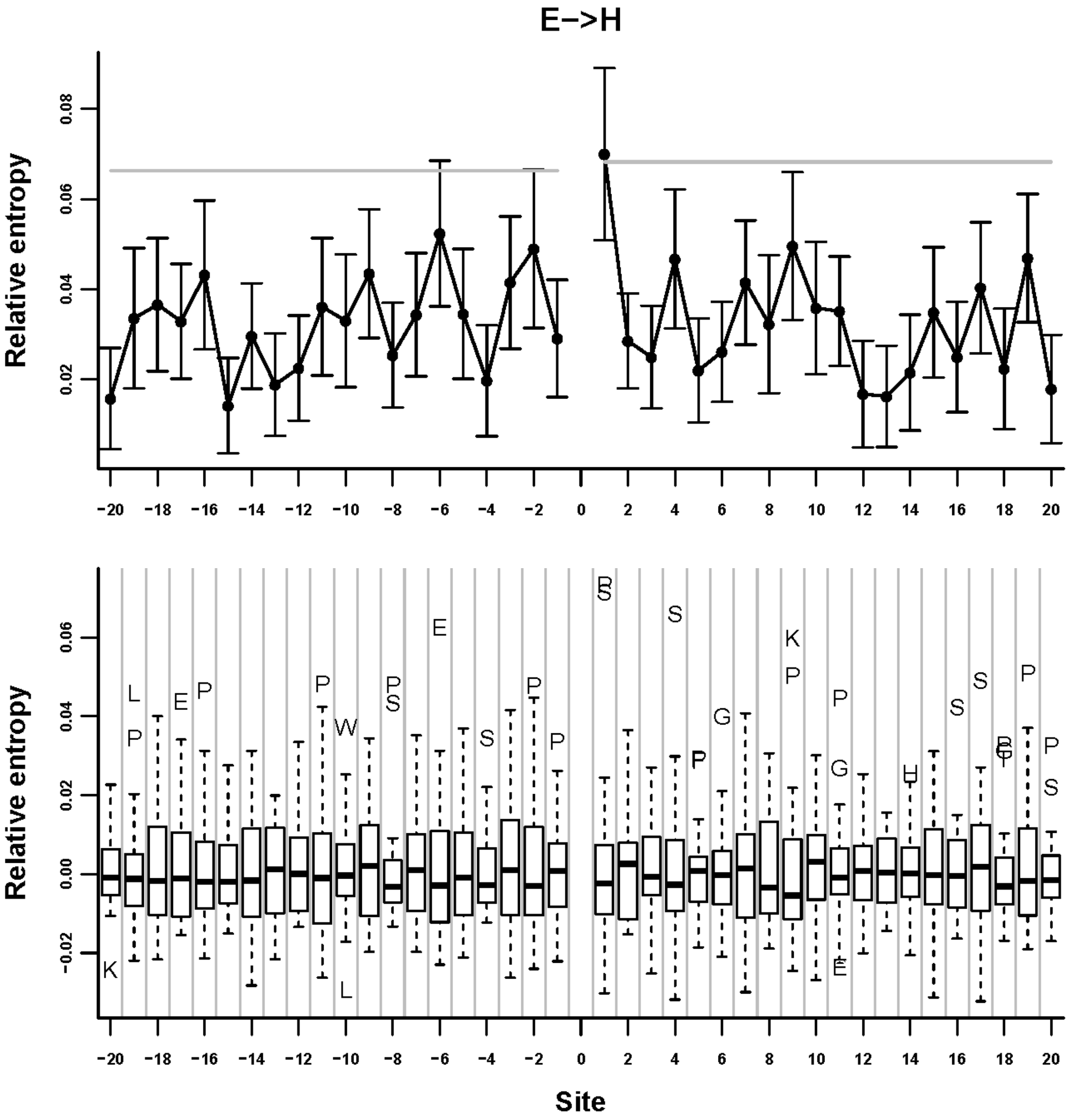
E−>H
Relative entropy
Relative entropy
Site

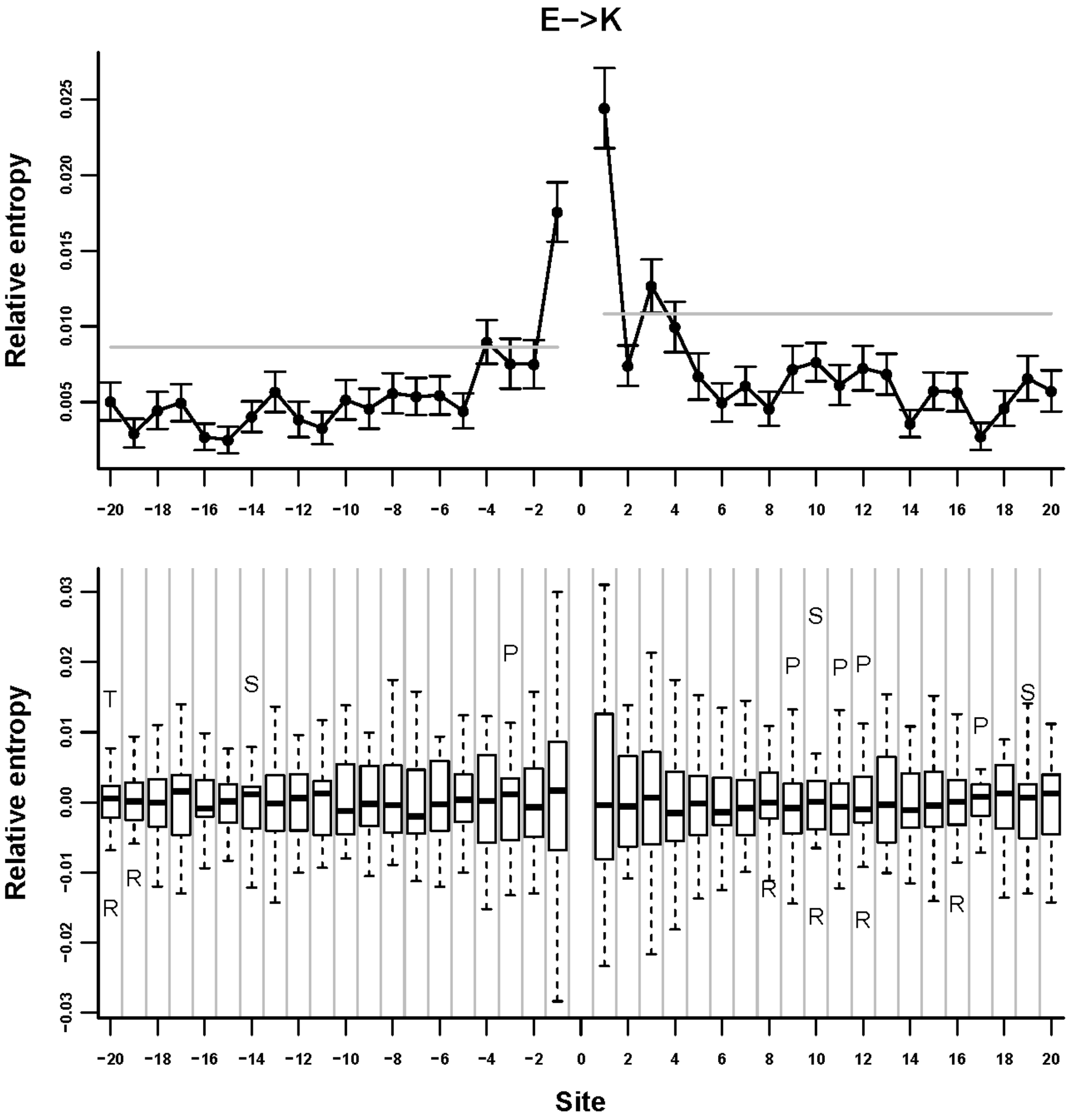
E−>K
Relative entropy
0.005
0.010
0.015
0.020
0.025
−20 −18 −16 −14 −12 −10 −8 −6 −4 −2 0 2 4 6 8 10 12 14 16 18 20
Relative entropy
−0.03
−0.02
−0.01
0.00
0.01
0.02
0.03
T
R
R
S
P
P
S
P
P
S
P
R
R
R
R
Site

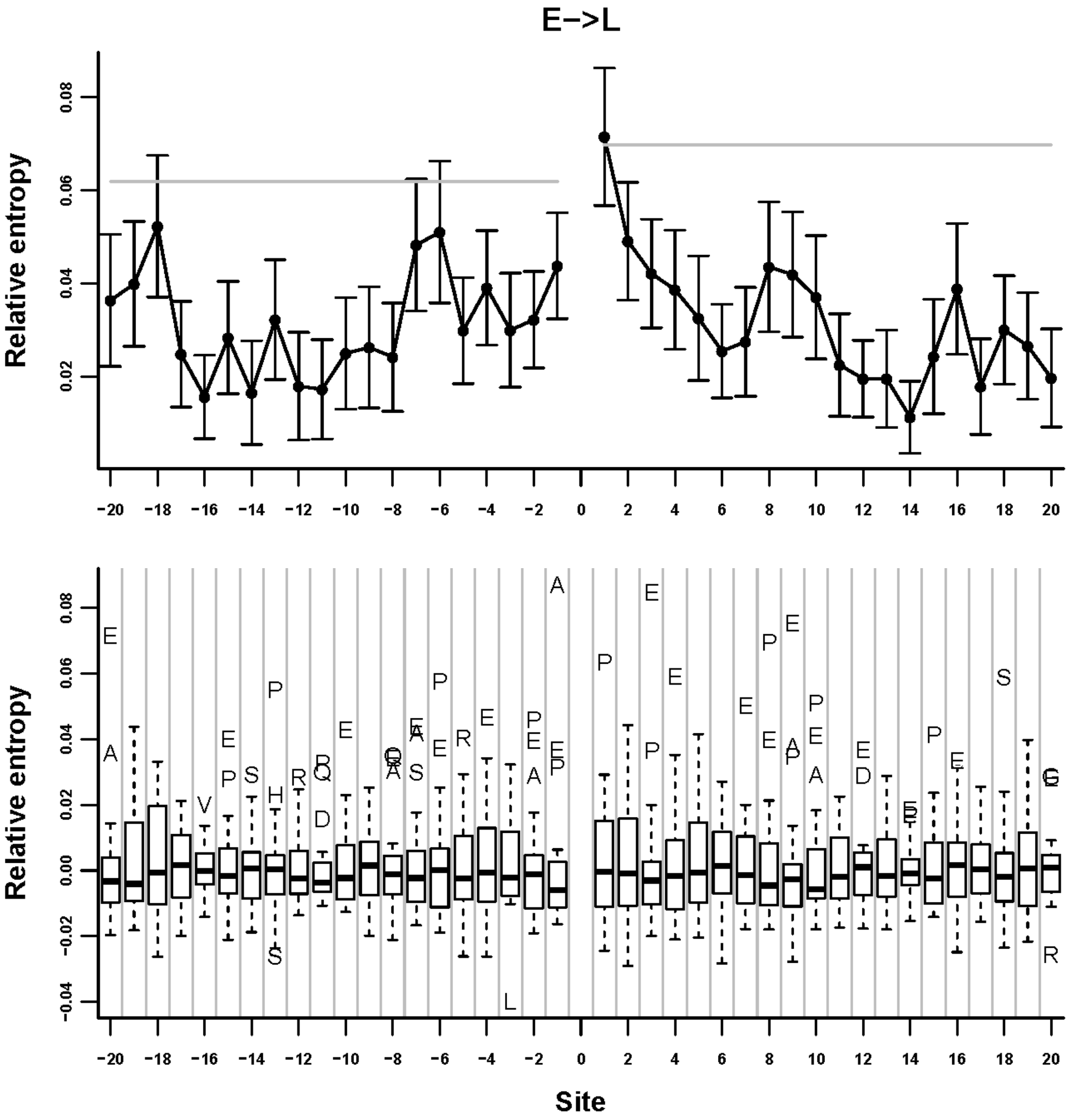
E−>L
Relative entropy
Relative entropy
Site

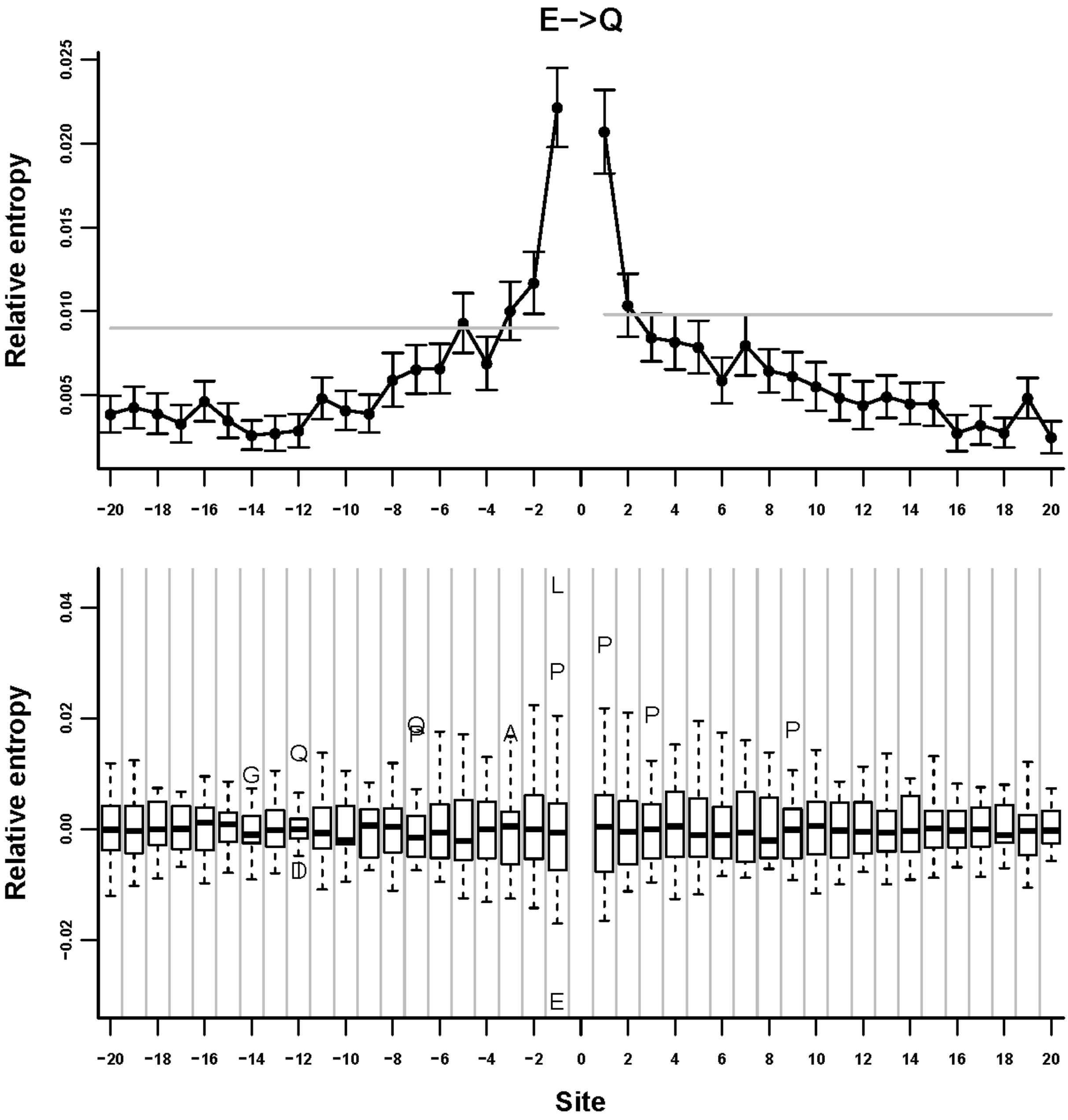
E−>Q
Relative entropy
0.025
0.020
0.015
0.010
0.005
−20 −18 −16 −14 −12 −10 −8 −6 −4 −2 0 2 4 6 8 10 12 14 16 18 20
Relative entropy
0.04
0.02
0.00
−0.02
L
P
P
P
P
P
Q
G
Q
A
D
E
−20 −18 −16 −14 −12 −10 −8 −6 −4 −2 0 2 4 6 8 10 12 14 16 18 20
Site

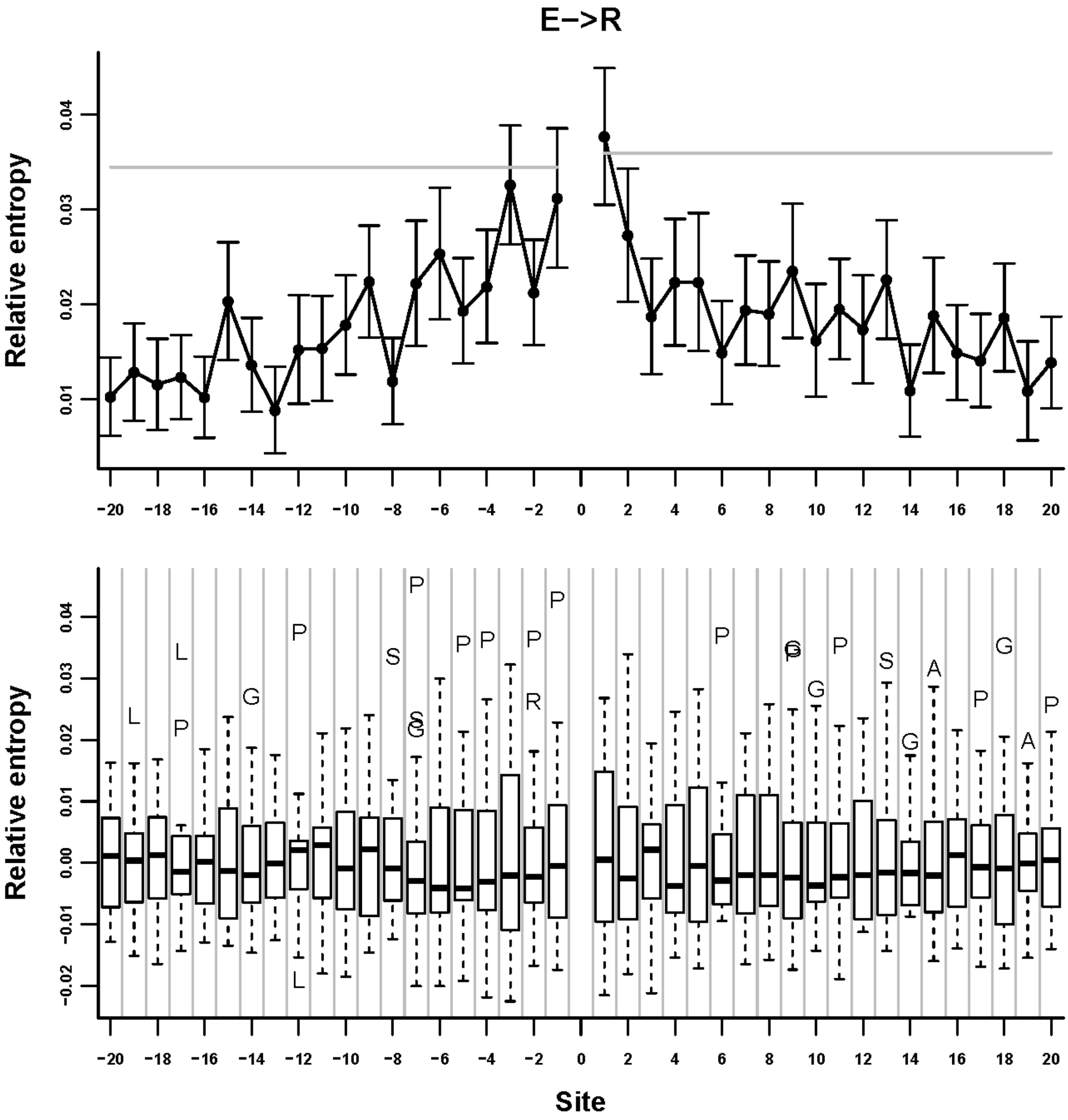
E−>R
Relative entropy
0.01
0.02
0.03
0.04
−20
−18
−16
−14
−12
−10
−8
−6
−4
−2
0
2
4
6
8
10
12
14
16
18
20
Relative entropy
−0.02
−0.01
0.00
0.01
0.02
0.03
0.04
Site

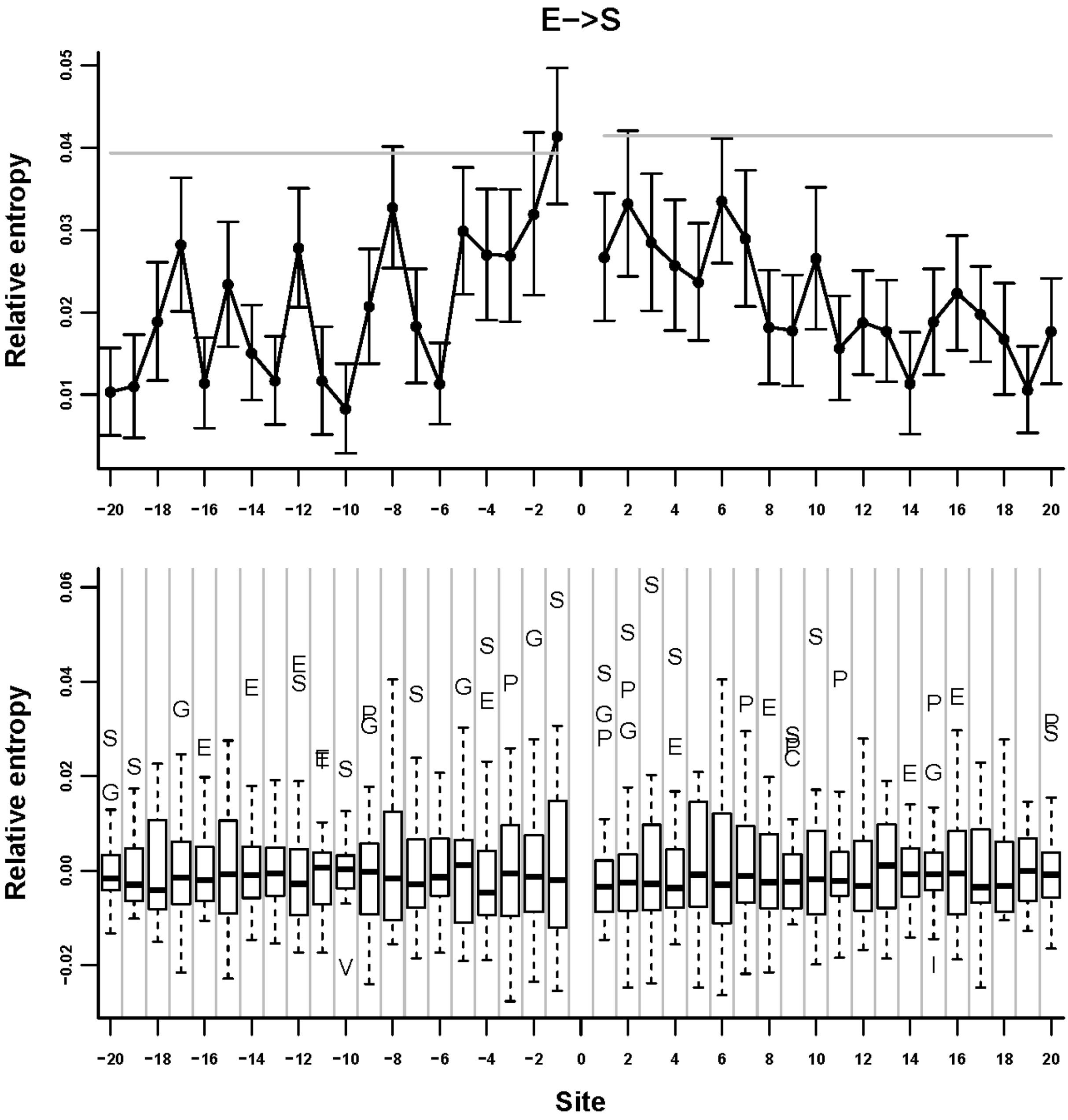
E−>S
Relative entropy
Relative entropy
Site

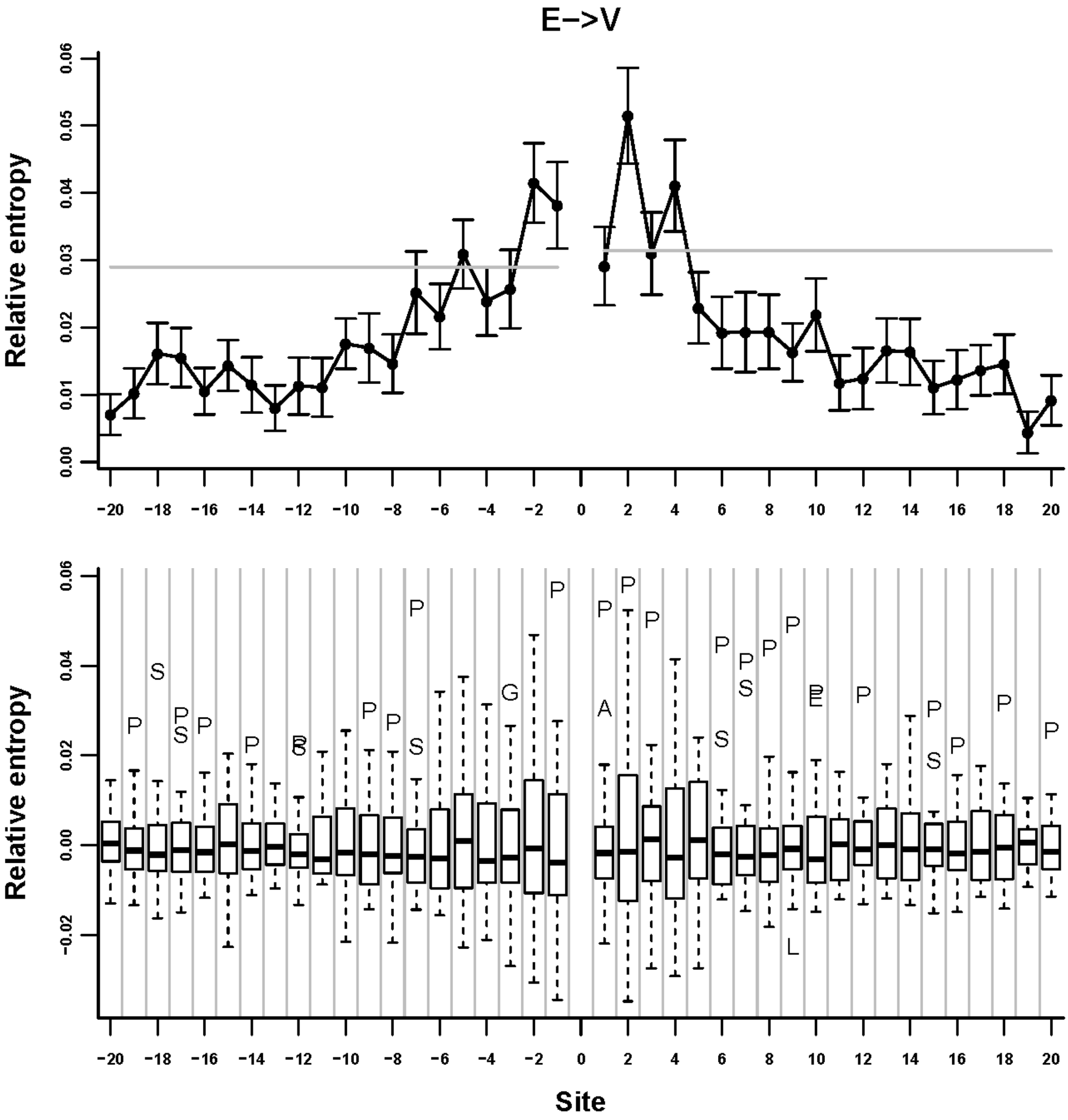
E−>V
Relative entropy
Site

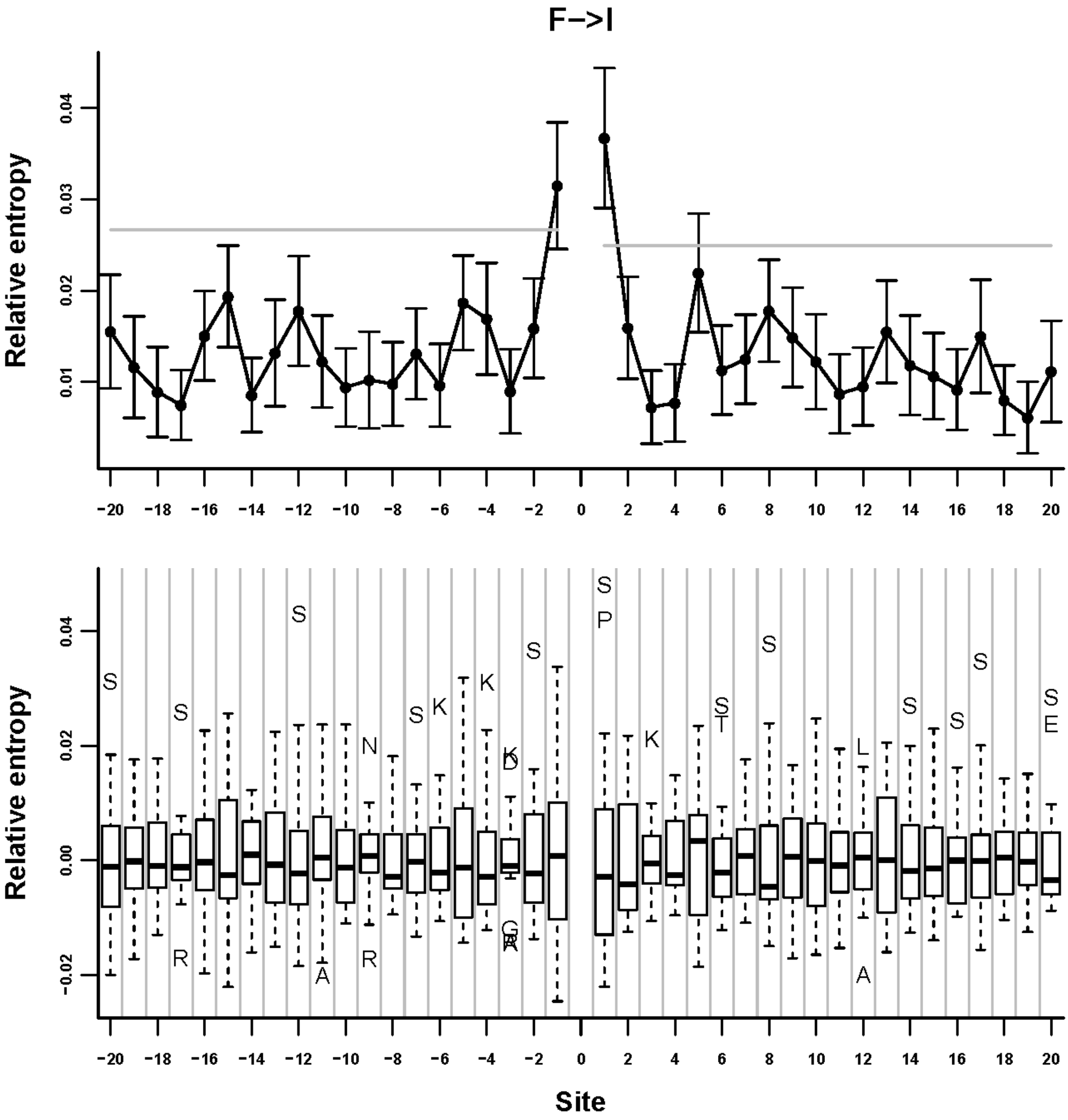
F->I
Relative entropy
Site
S
P
K
L
A
N
R
T
E
D
G

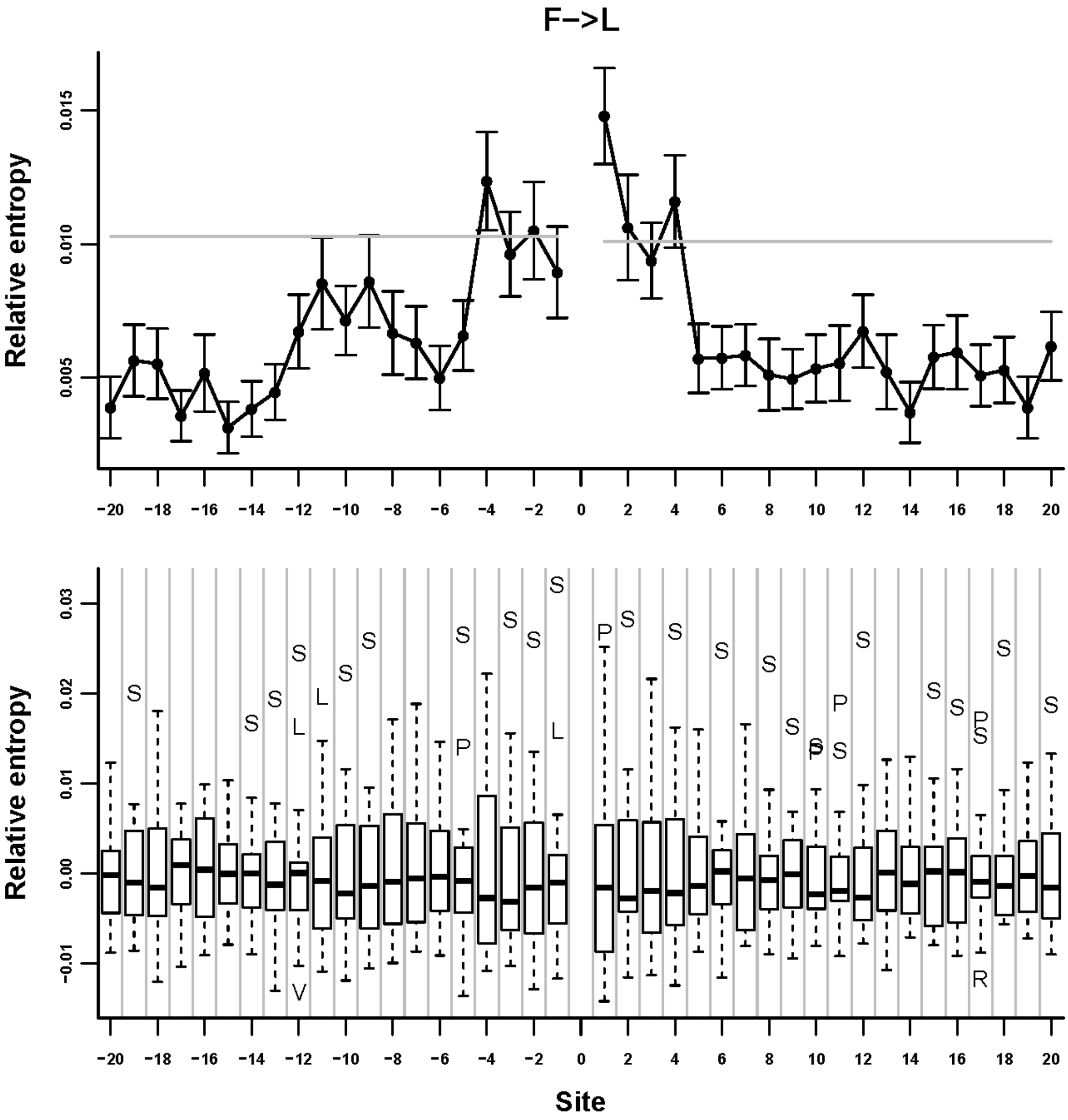
F−>L
Relative entropy
Relative entropy
Site

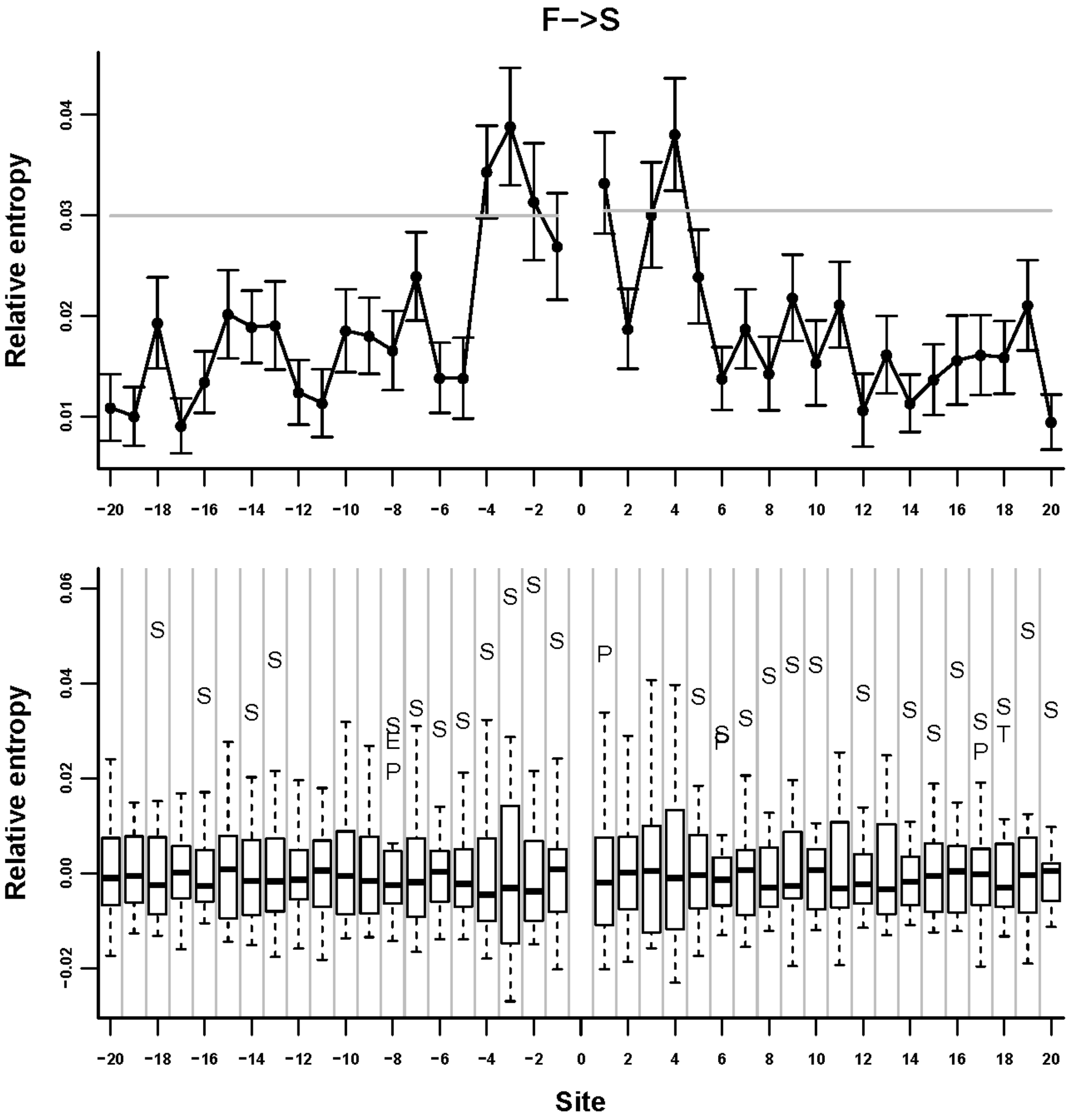
F−>S
Relative entropy
Relative entropy
Site

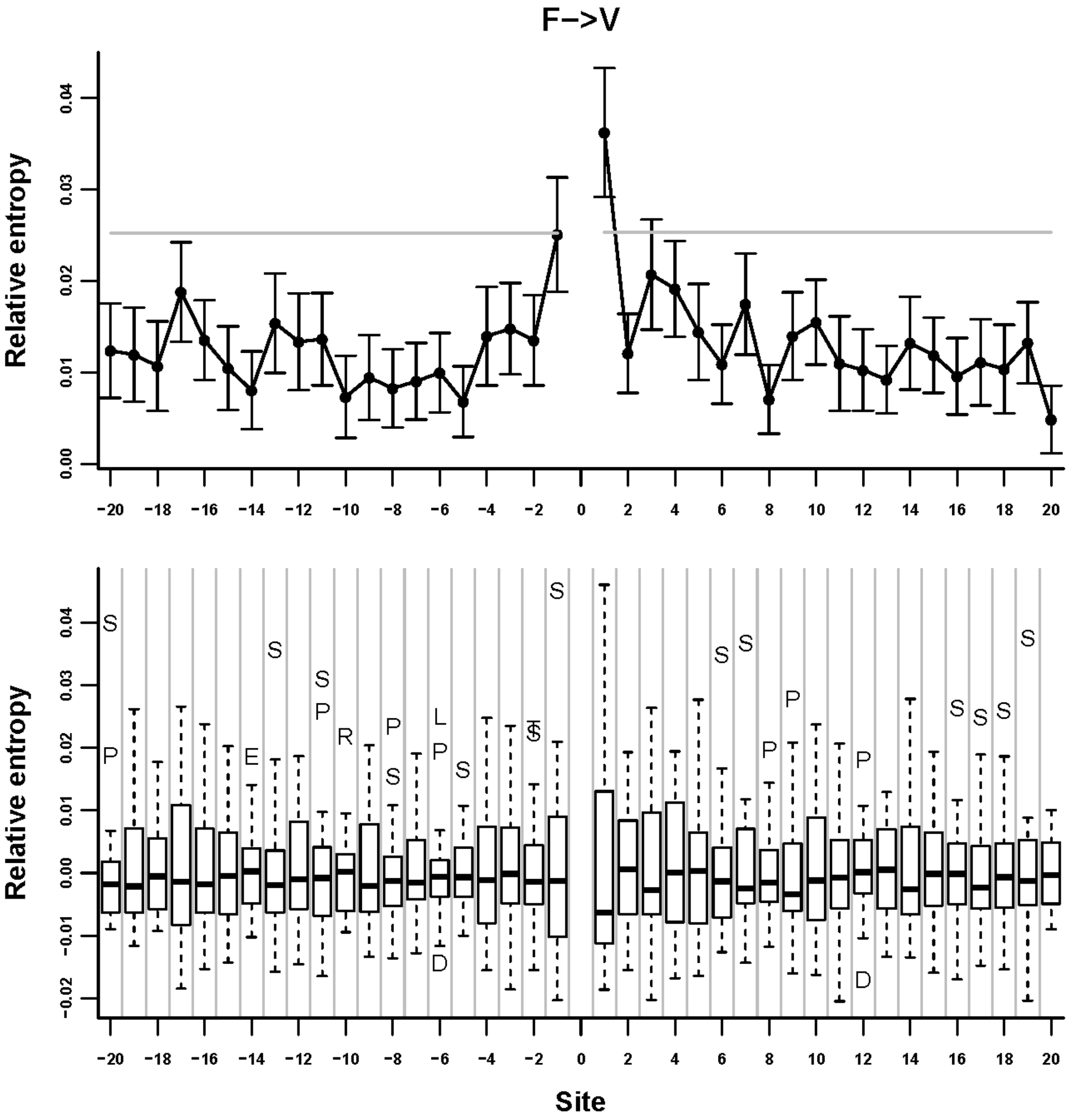
F−>V
Relative entropy
Relative entropy
Site

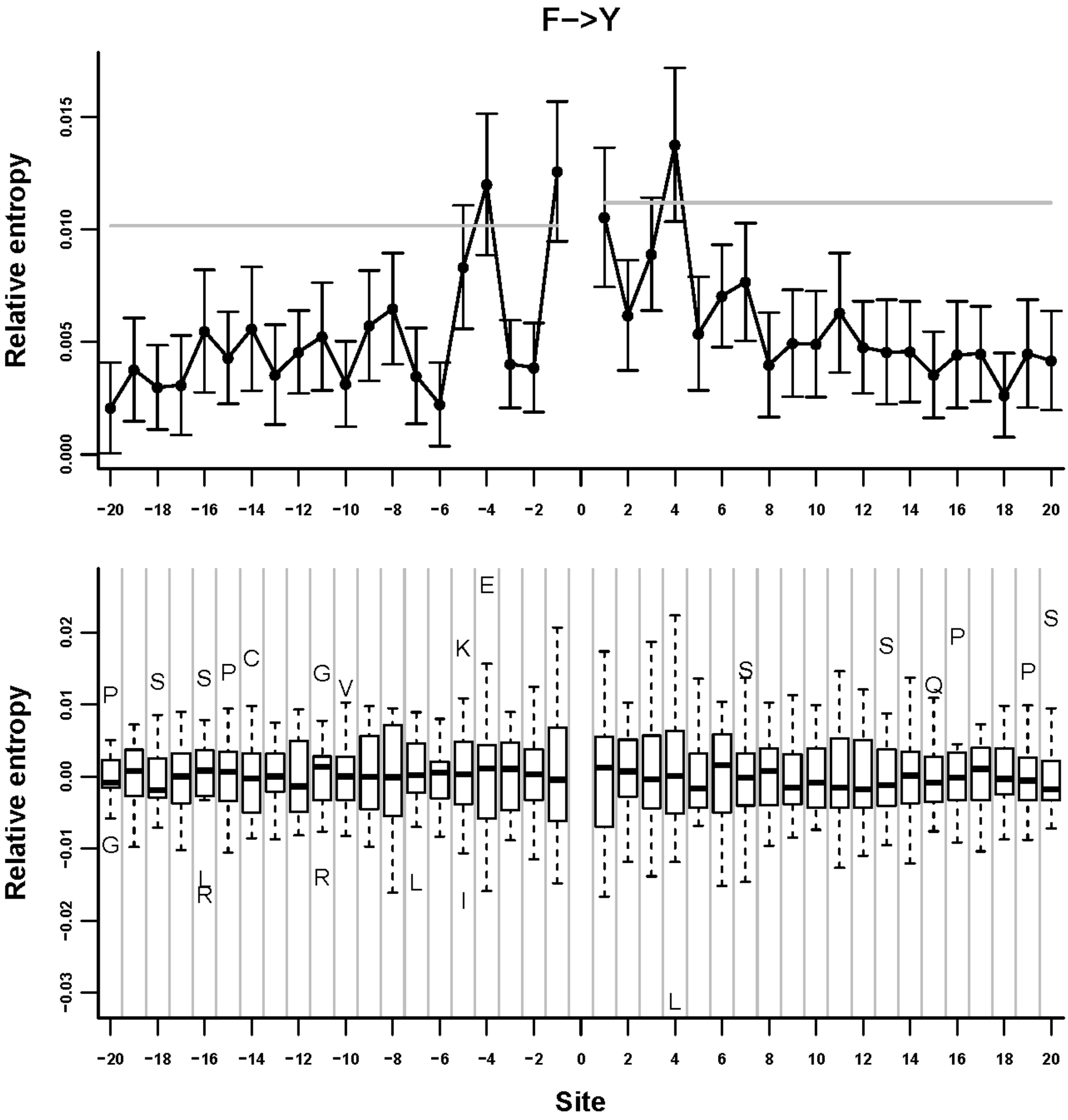
F−>Y
Relative entropy
Site
P
G
S
S
L
R
P
C
G
R
V
L
K
I
E
L
S
S
P
Q
P
S

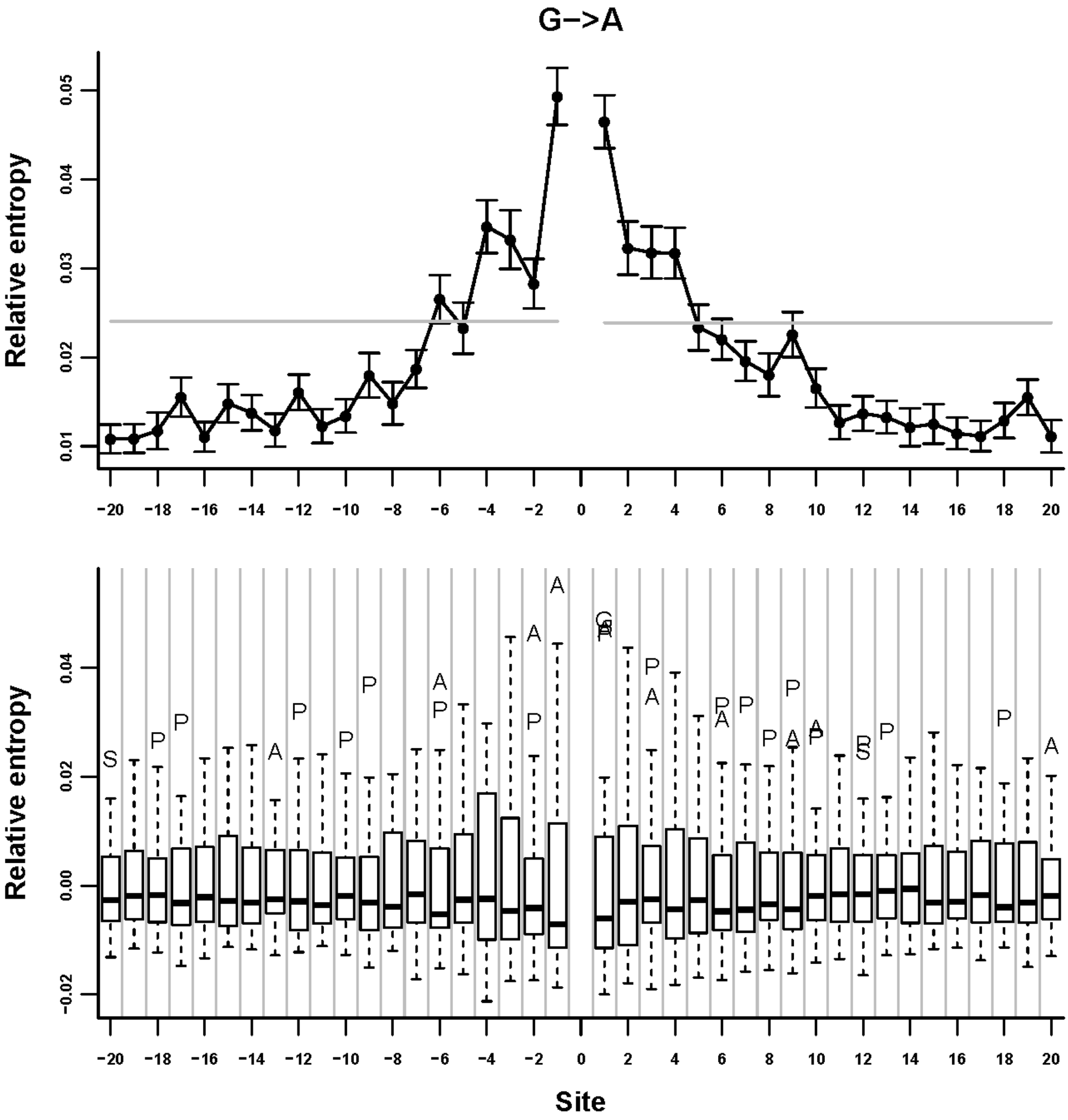
G−>A
Relative entropy
Site

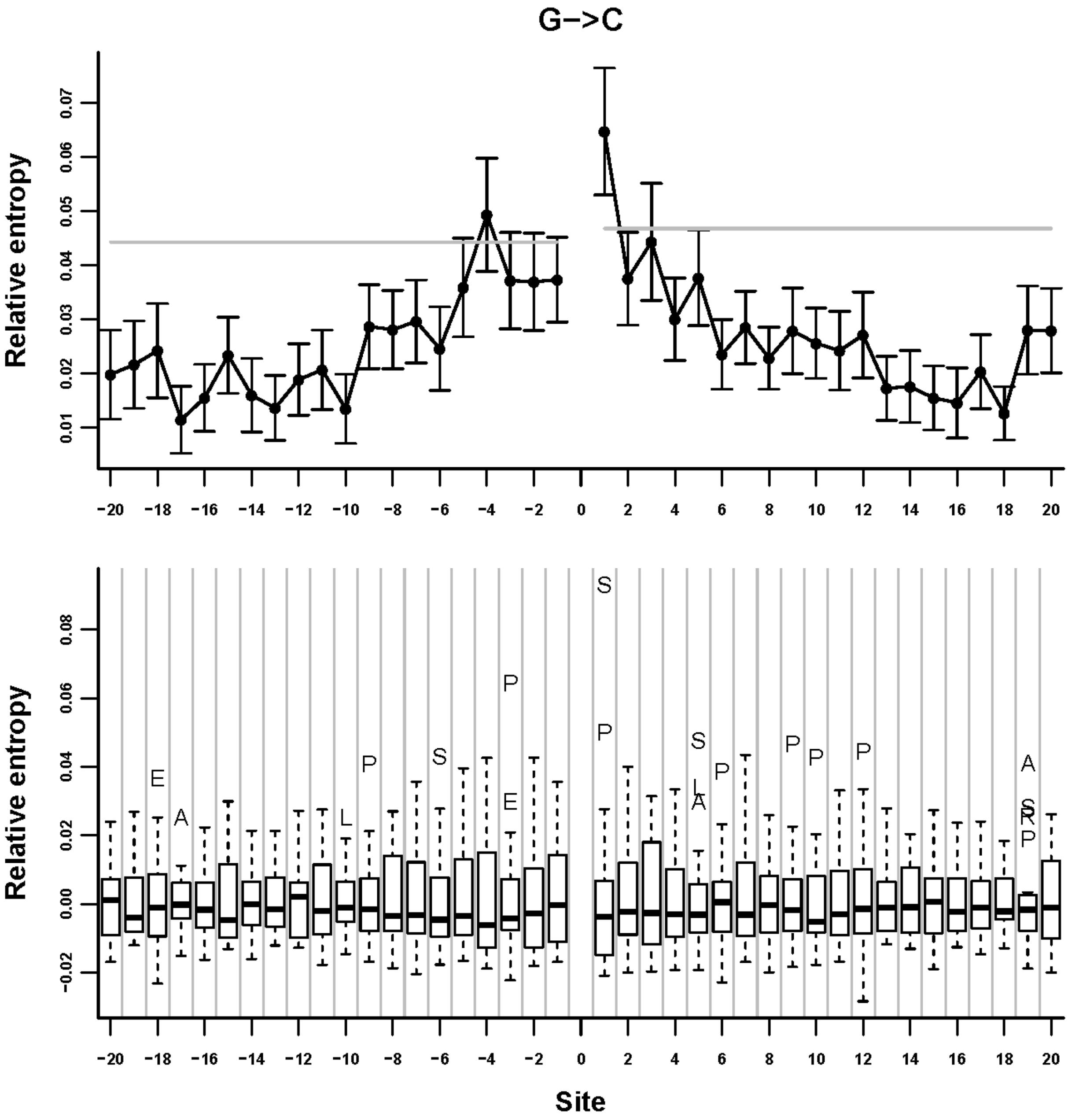
G−>C
Relative entropy
Relative entropy
Site
S
P
P
E
E
A
P
L
S
S
L
A
P
P
P
P
A
S
R
P

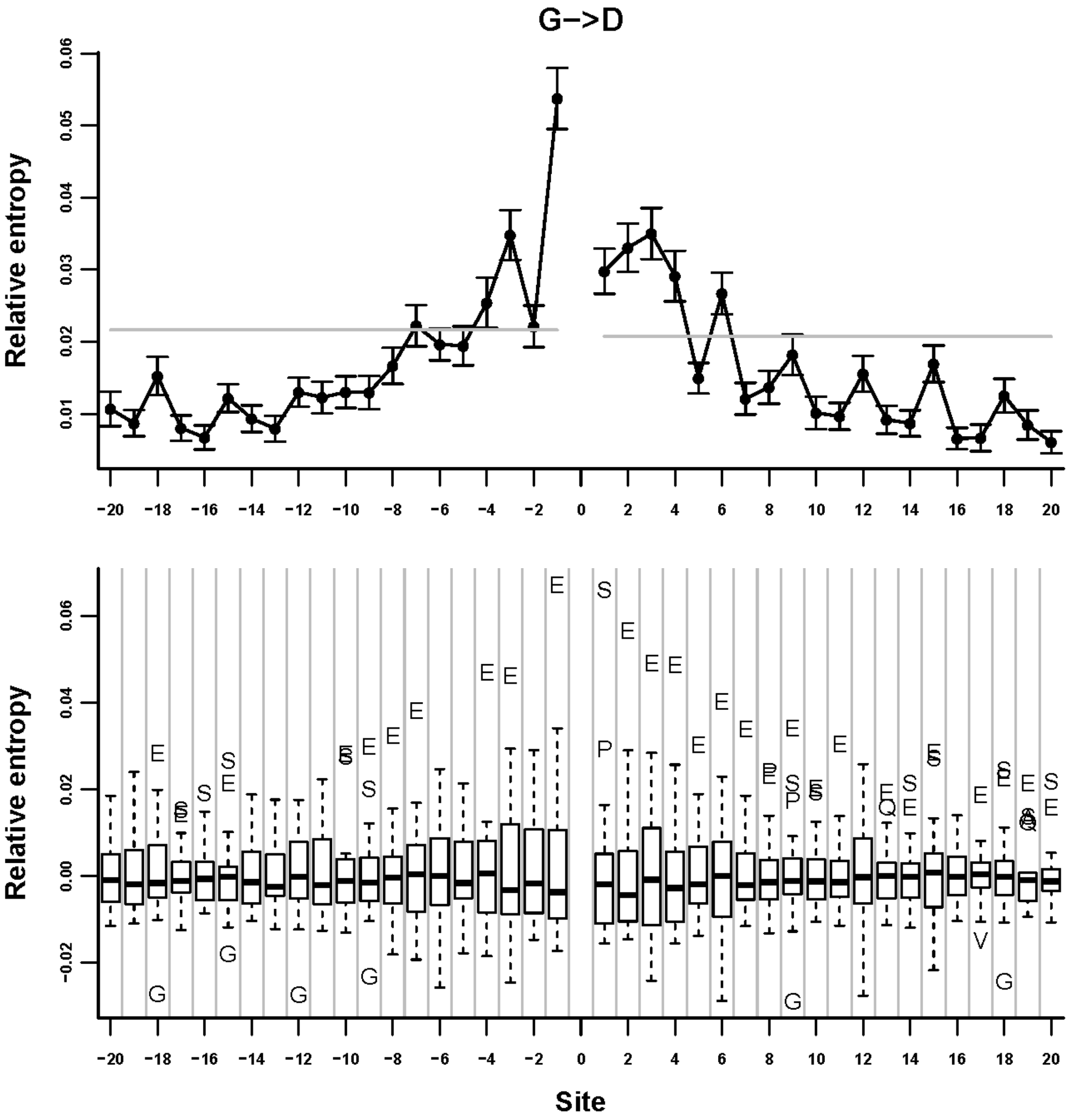
G−>D
Relative entropy
Relative entropy
Site

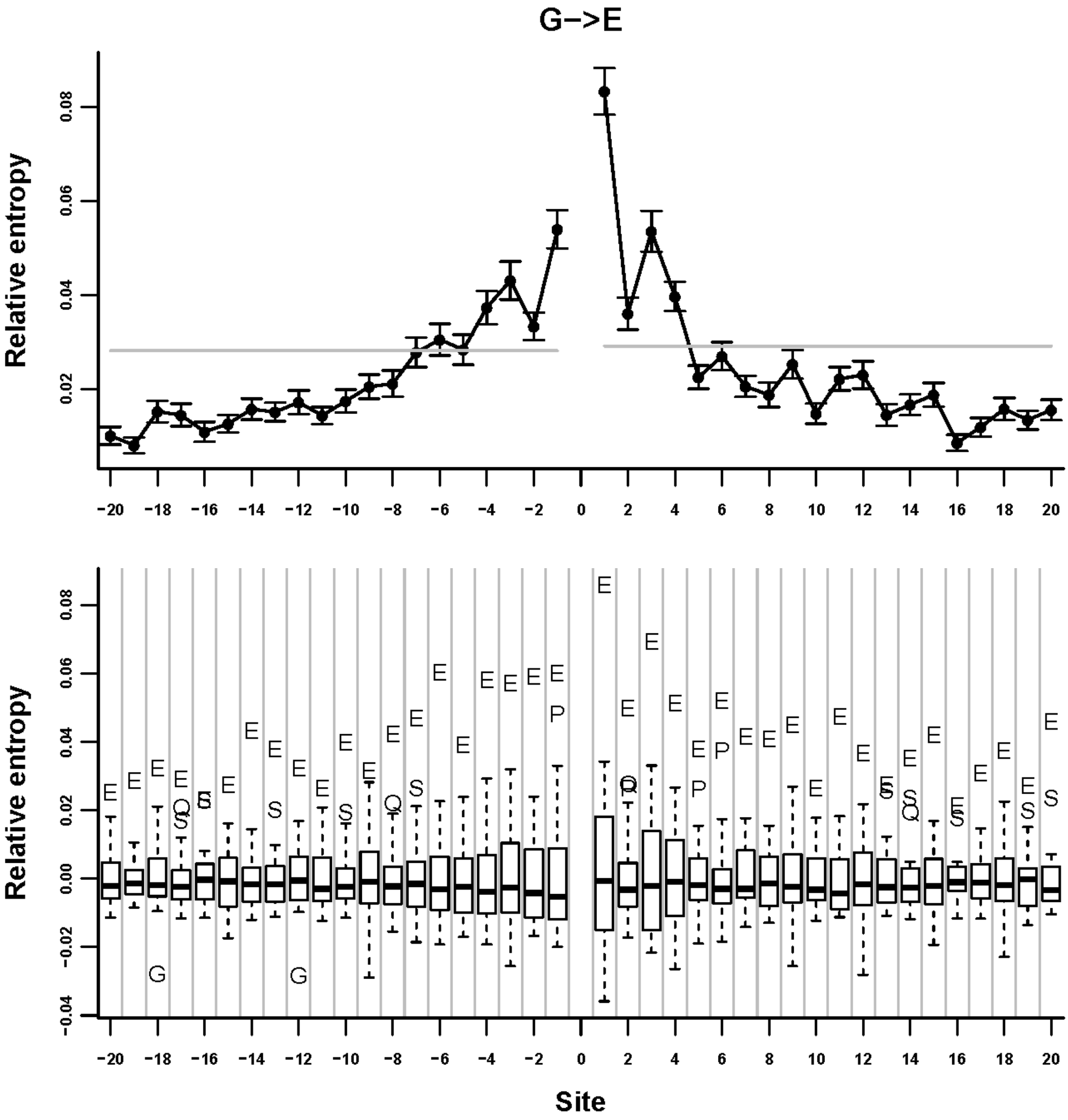
G−>E
Relative entropy
Relative entropy
Site

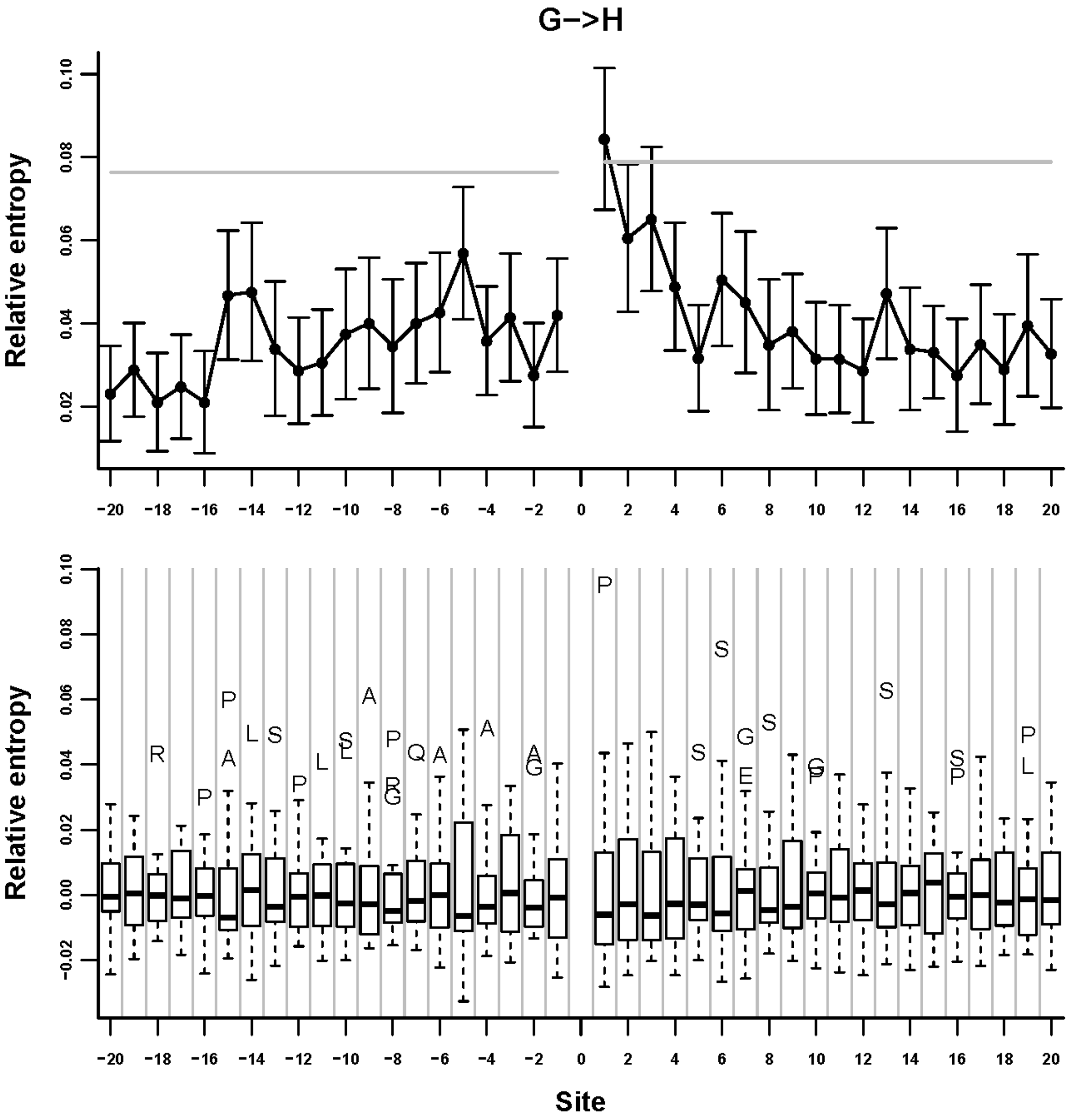
G−>H
Relative entropy
Relative entropy
Site

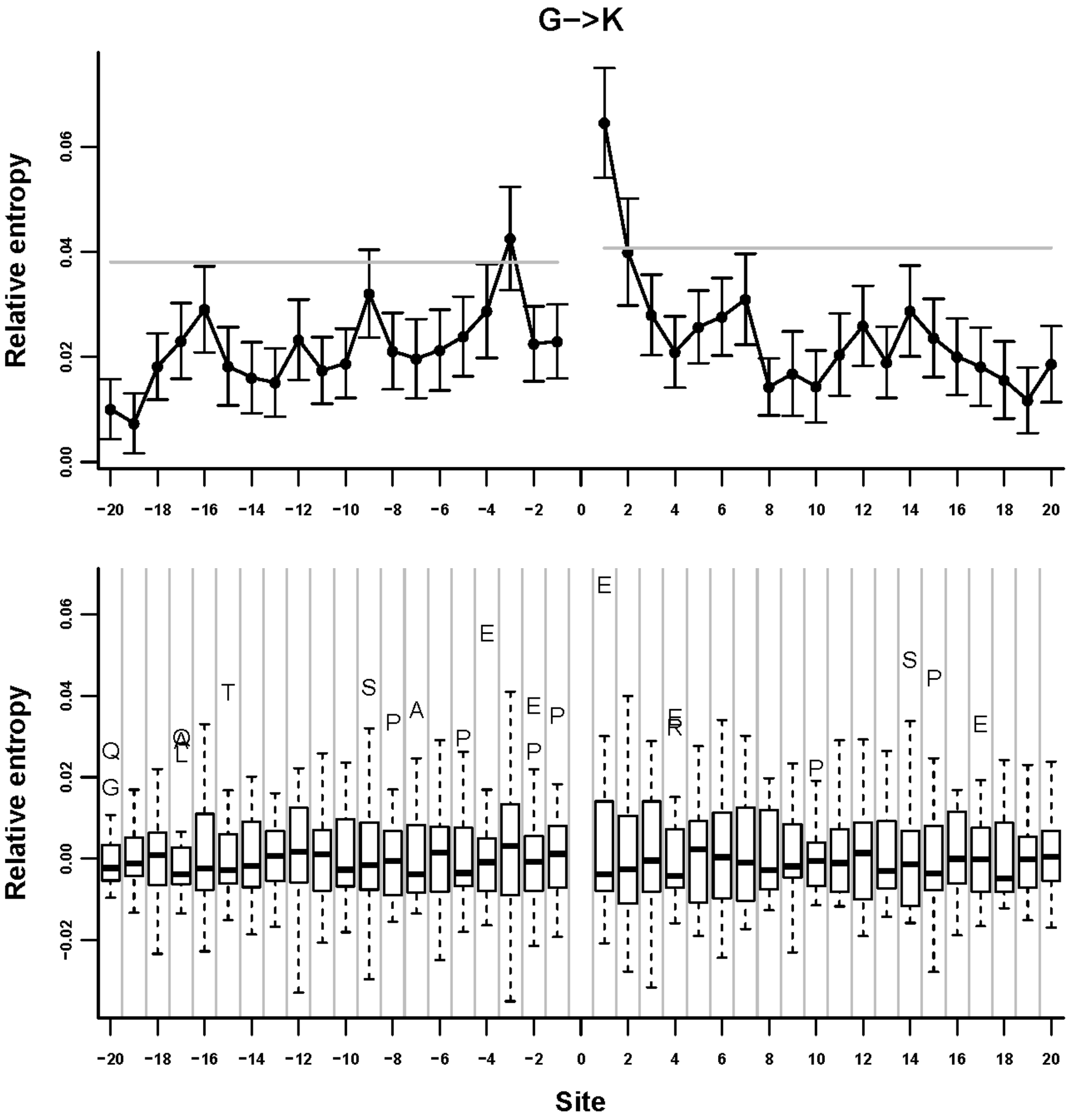
G−>K
Relative entropy
0.00
0.02
0.04
0.06
−20
−18
−16
−14
−12
−10
−8
−6
−4
−2
0
2
4
6
8
10
12
14
16
18
20
Site
−0.02
Q
G
A
L
T
S
P
A
E
E
P
P
E
R
P
S
P
E

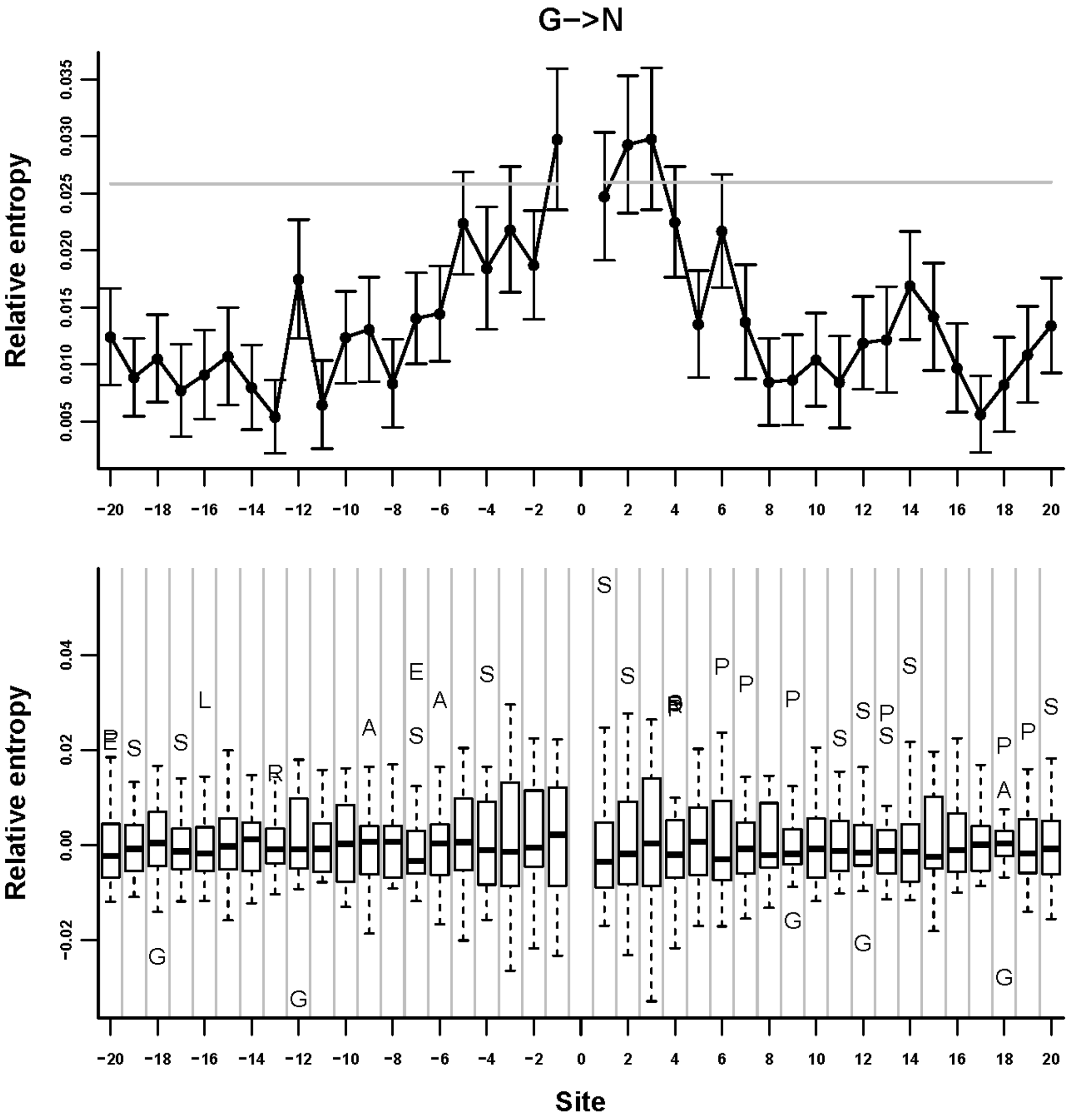
G−>N
Relative entropy
Relative entropy
Site

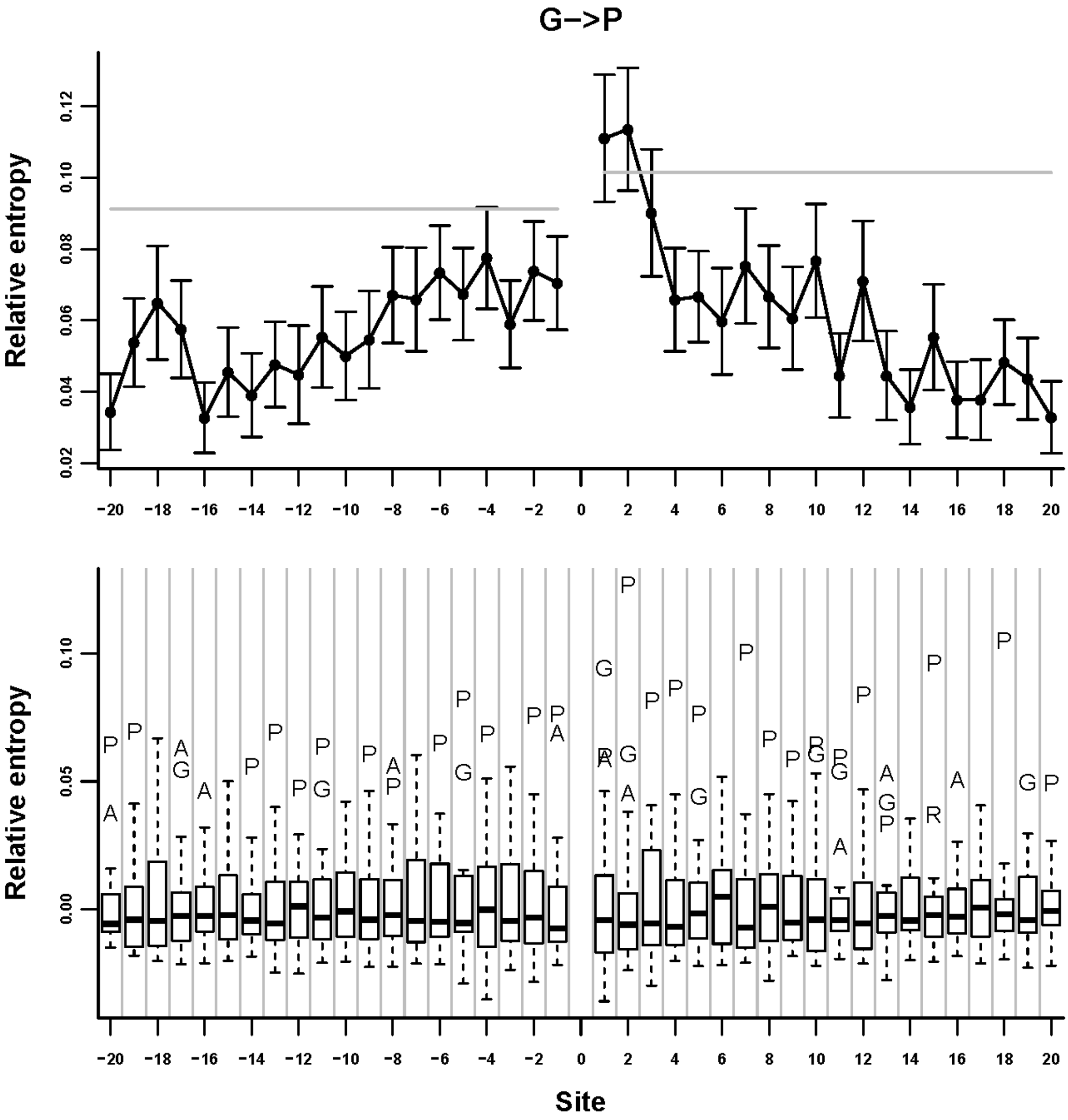
G−>P
Relative entropy
Relative entropy
Site

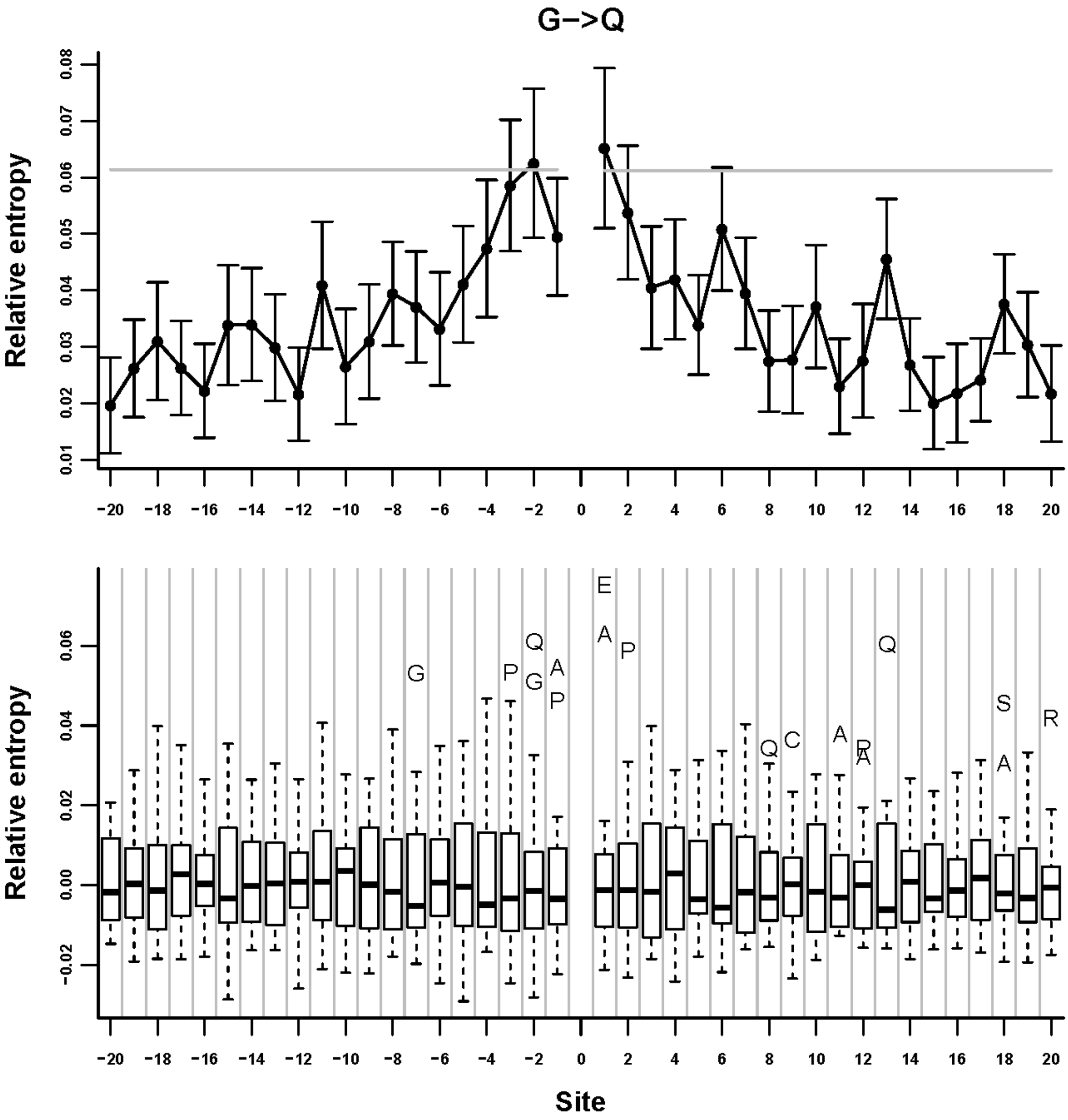
G−>Q
Relative entropy
Relative entropy
Site
G
P
Q
G
A
P
E
A
P
Q
Q
C
A
R
A
S
A
R

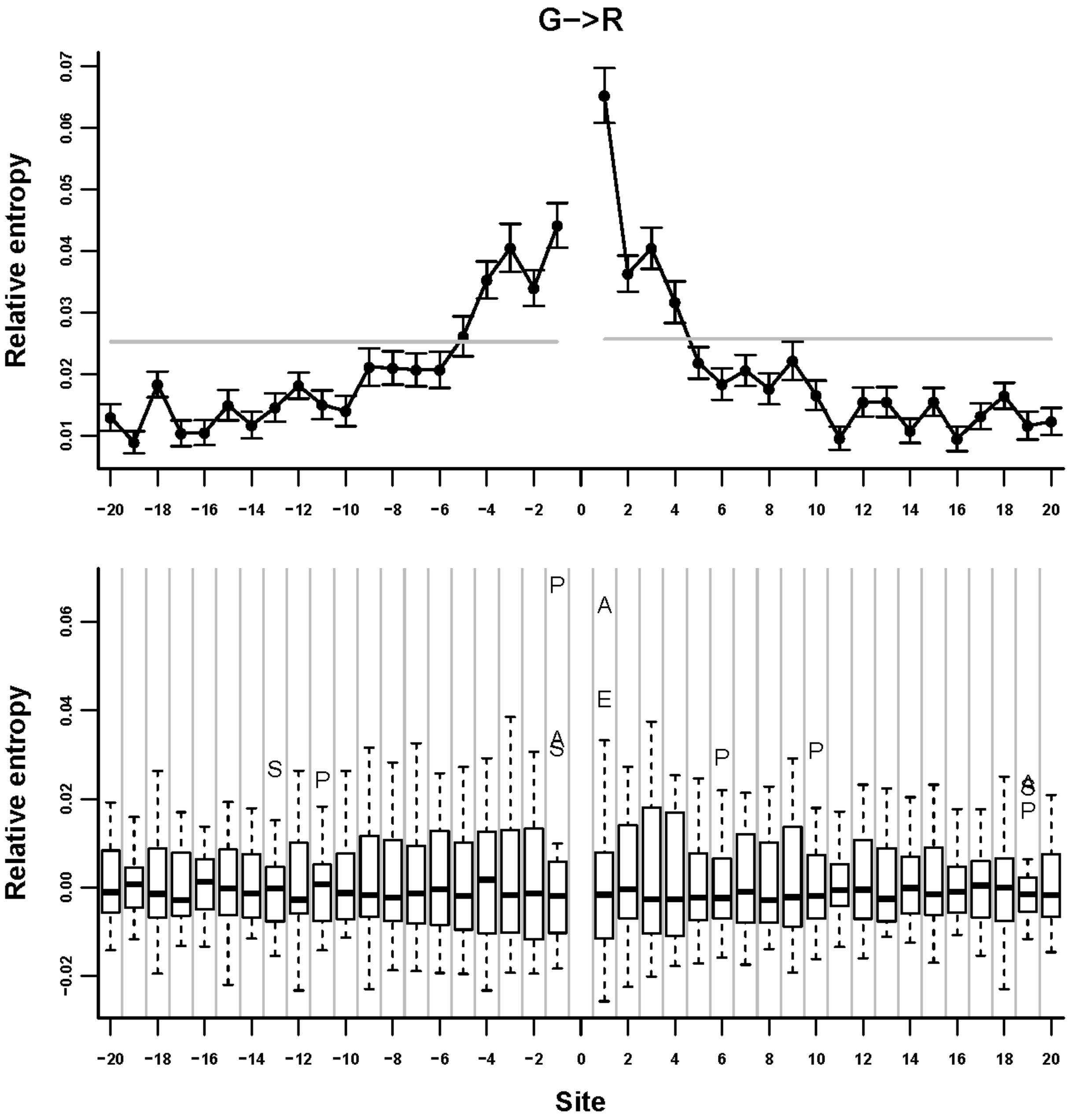
G->R
Relative entropy
0.01
0.02
0.03
0.04
0.05
0.06
0.07
-20 -18 -16 -14 -12 -10 -8 -6 -4 -2 0 2 4 6 8 10 12 14 16 18 20
Relative entropy
-0.02
0.00
0.02
0.04
0.06
P
A
E
A
S
S
P
P
P
A
S
P
-20 -18 -16 -14 -12 -10 -8 -6 -4 -2 0 2 4 6 8 10 12 14 16 18 20
Site

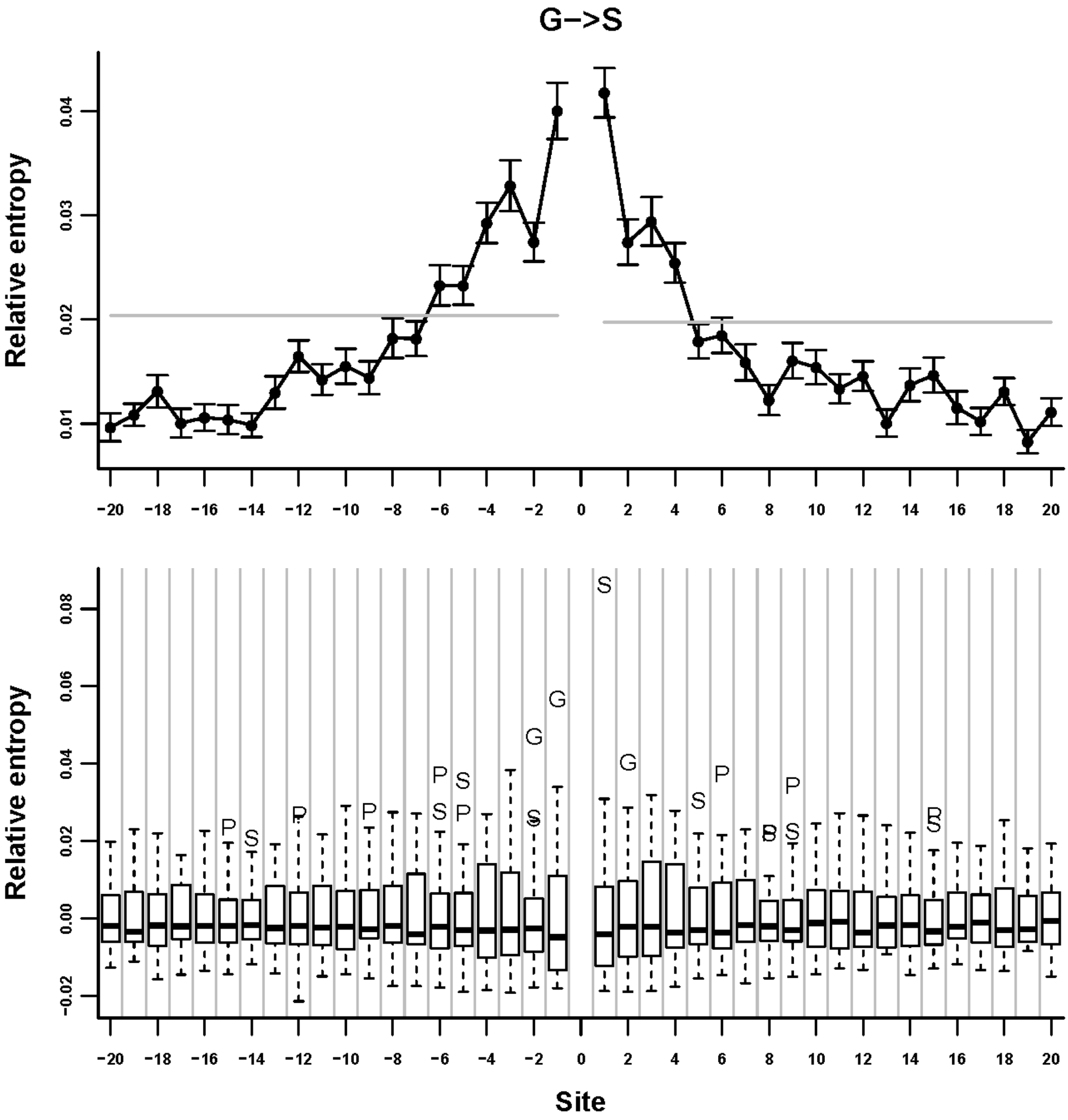

G−>S
Relative entropy
0.01
0.02
0.03
0.04
−20
−18
−16
−14
−12
−10
−8
−6
−4
−2
0
2
4
6
8
10
12
14
16
18
20
Relative entropy
−0.02
0.00
0.02
0.04
0.06
0.08
Site

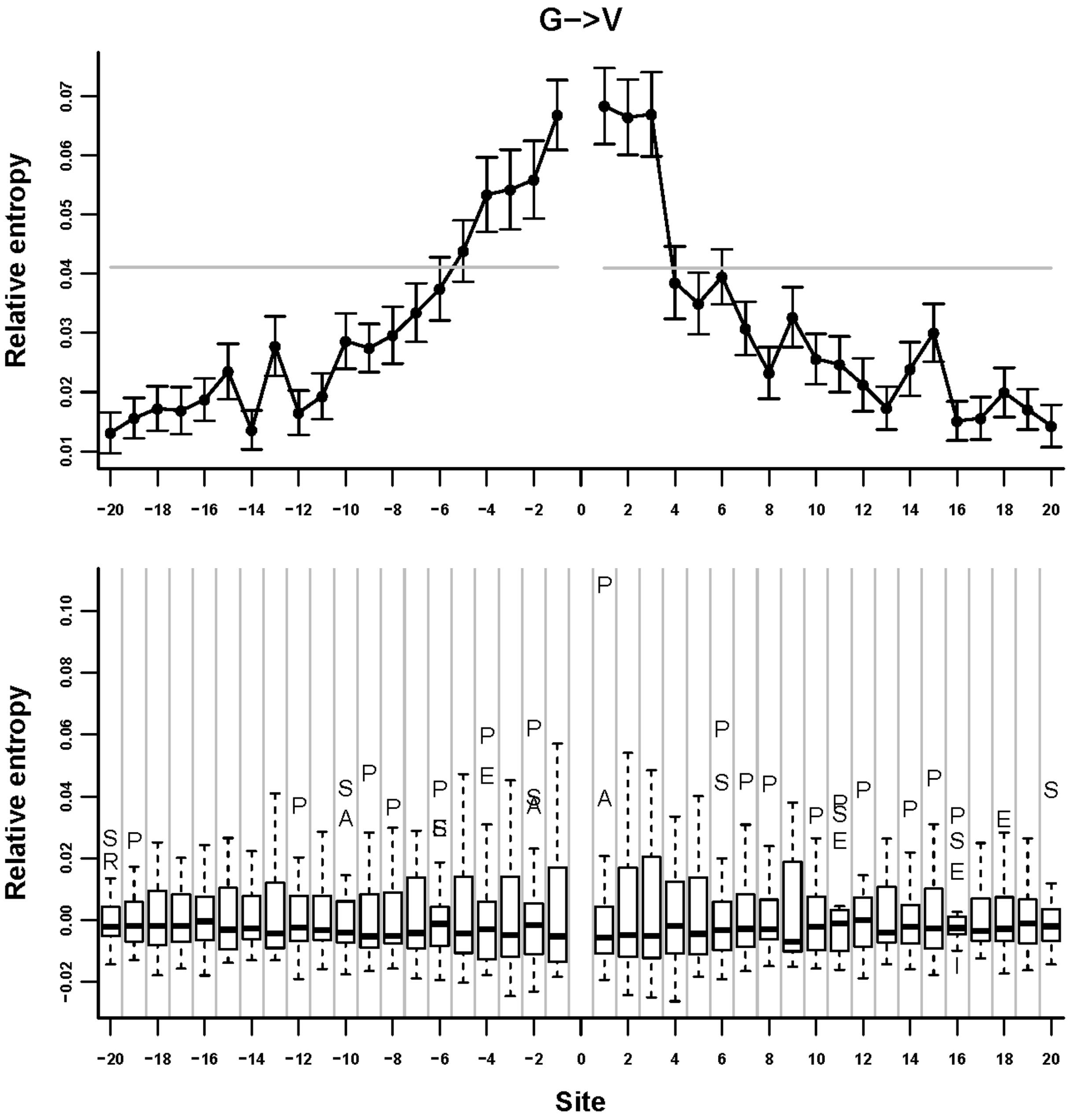
G->V
Relative entropy
Relative entropy
Site

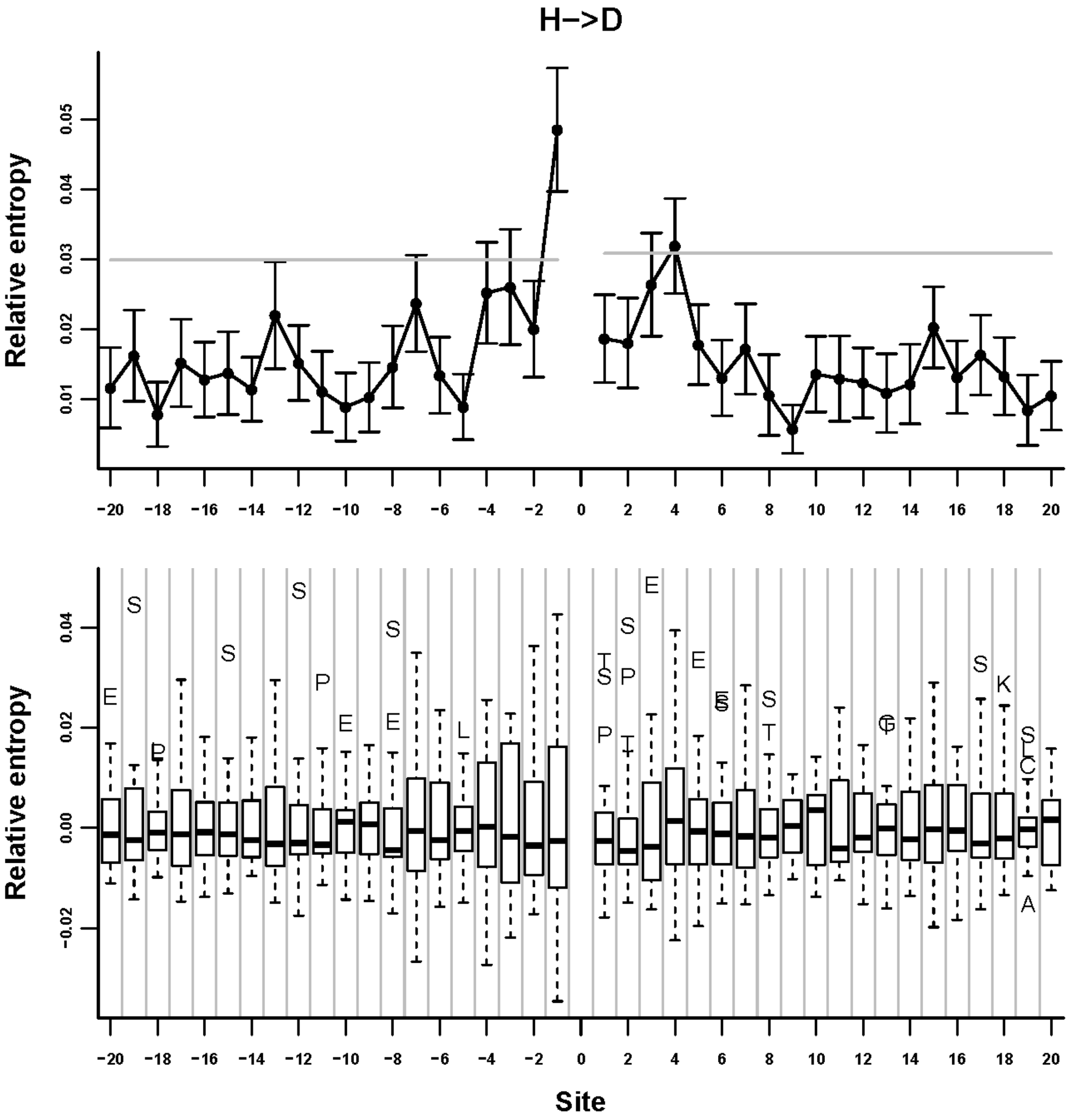
H−>D
Relative entropy
Relative entropy
Site

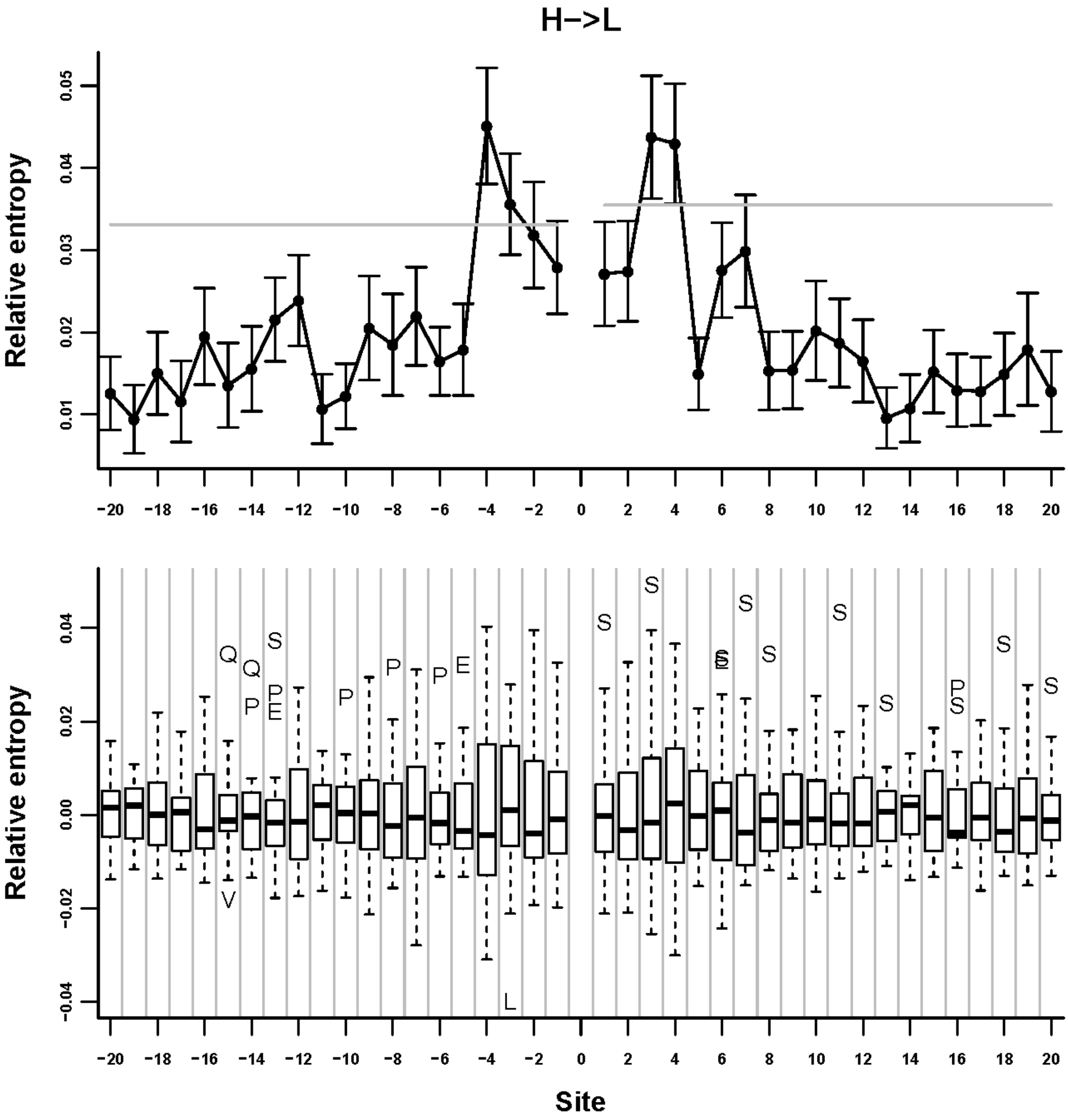
H−>L
Relative entropy
0.01
0.02
0.03
0.04
0.05
−20 −18 −16 −14 −12 −10 −8 −6 −4 −2 0 2 4 6 8 10 12 14 16 18 20
Relative entropy
−0.04
−0.02
0.00
0.02
0.04
Site

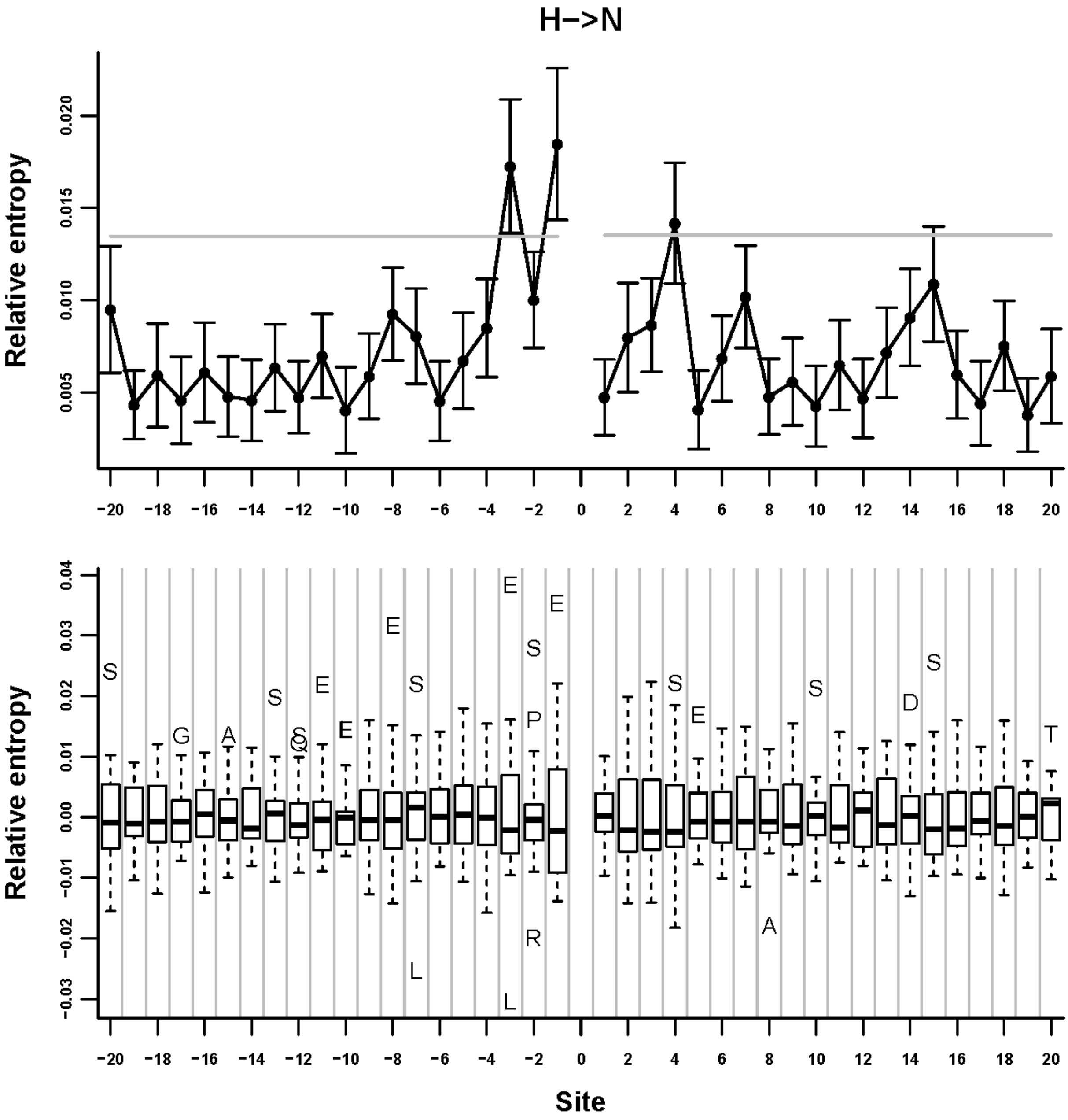
H−>N
Relative entropy
0.005
0.010
0.015
0.020
−20
−18
−16
−14
−12
−10
−8
−6
−4
−2
0
2
4
6
8
10
12
14
16
18
20
Relative entropy
Site

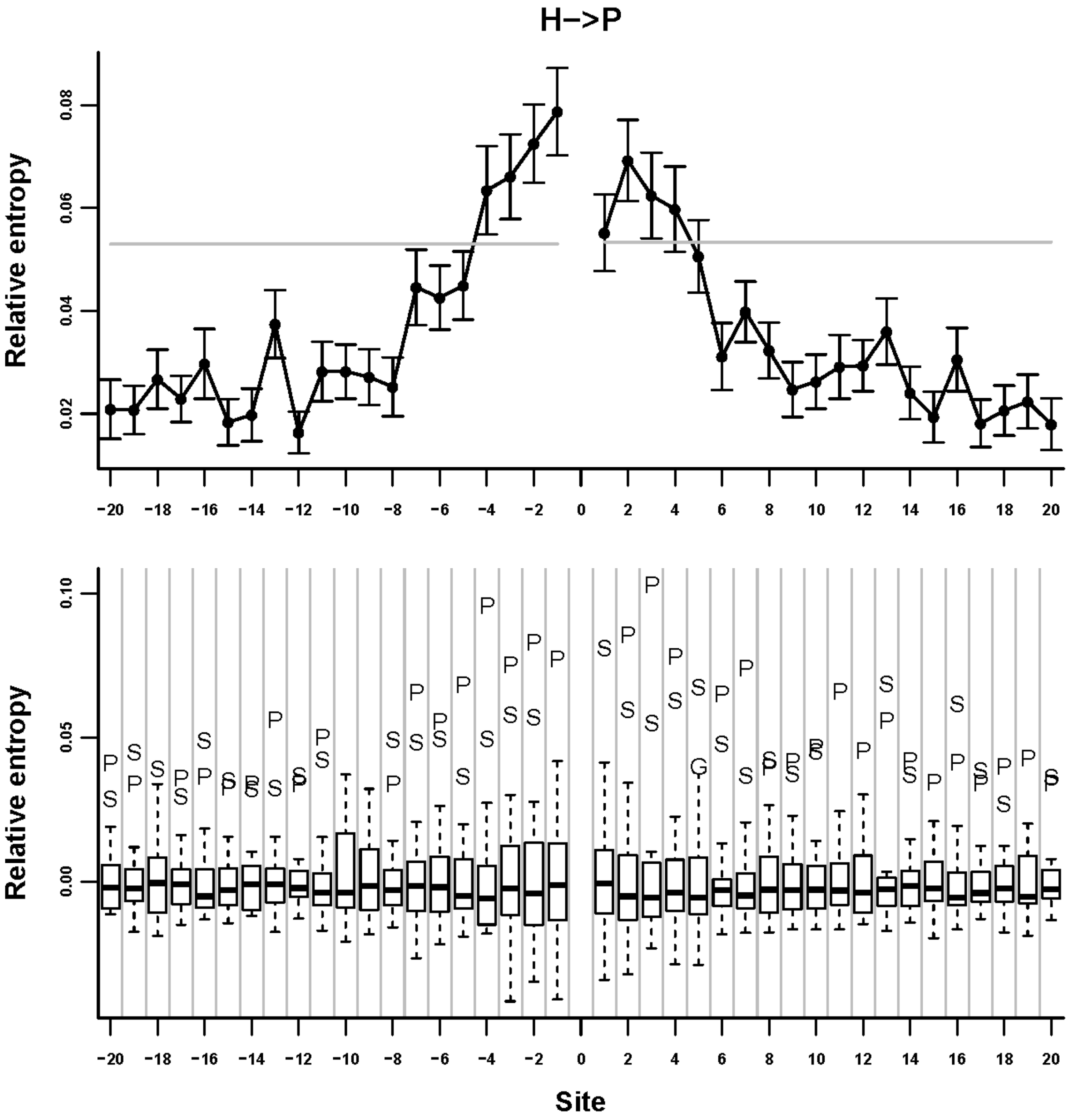
H−>P
Relative entropy
0.02
0.04
0.06
0.08
−20
−18
−16
−14
−12
−10
−8
−6
−4
−2
0
2
4
6
8
10
12
14
16
18
20
Relative entropy
0.00
0.05
0.10
Site

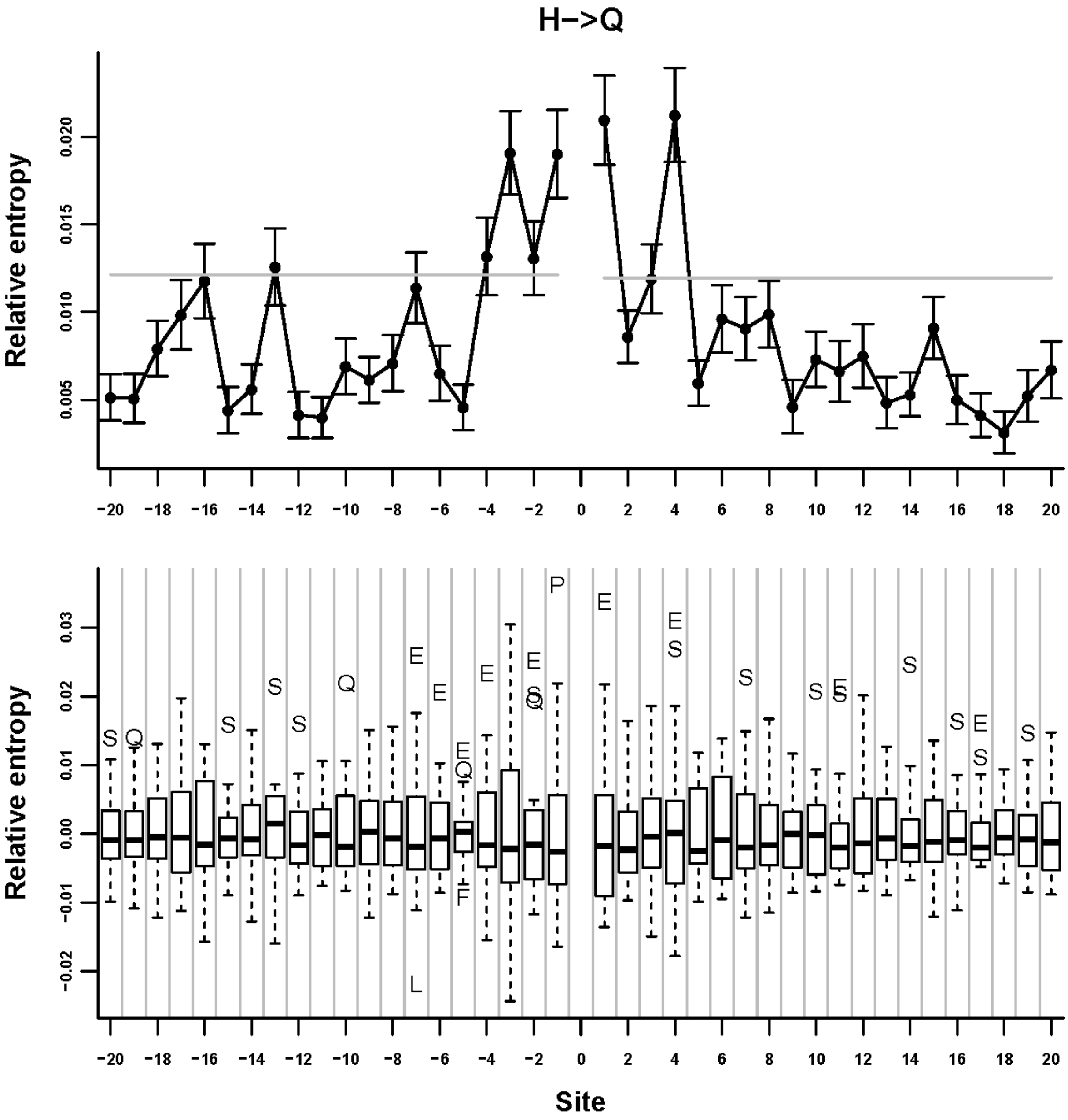

H->Q
Relative entropy
Relative entropy
Site

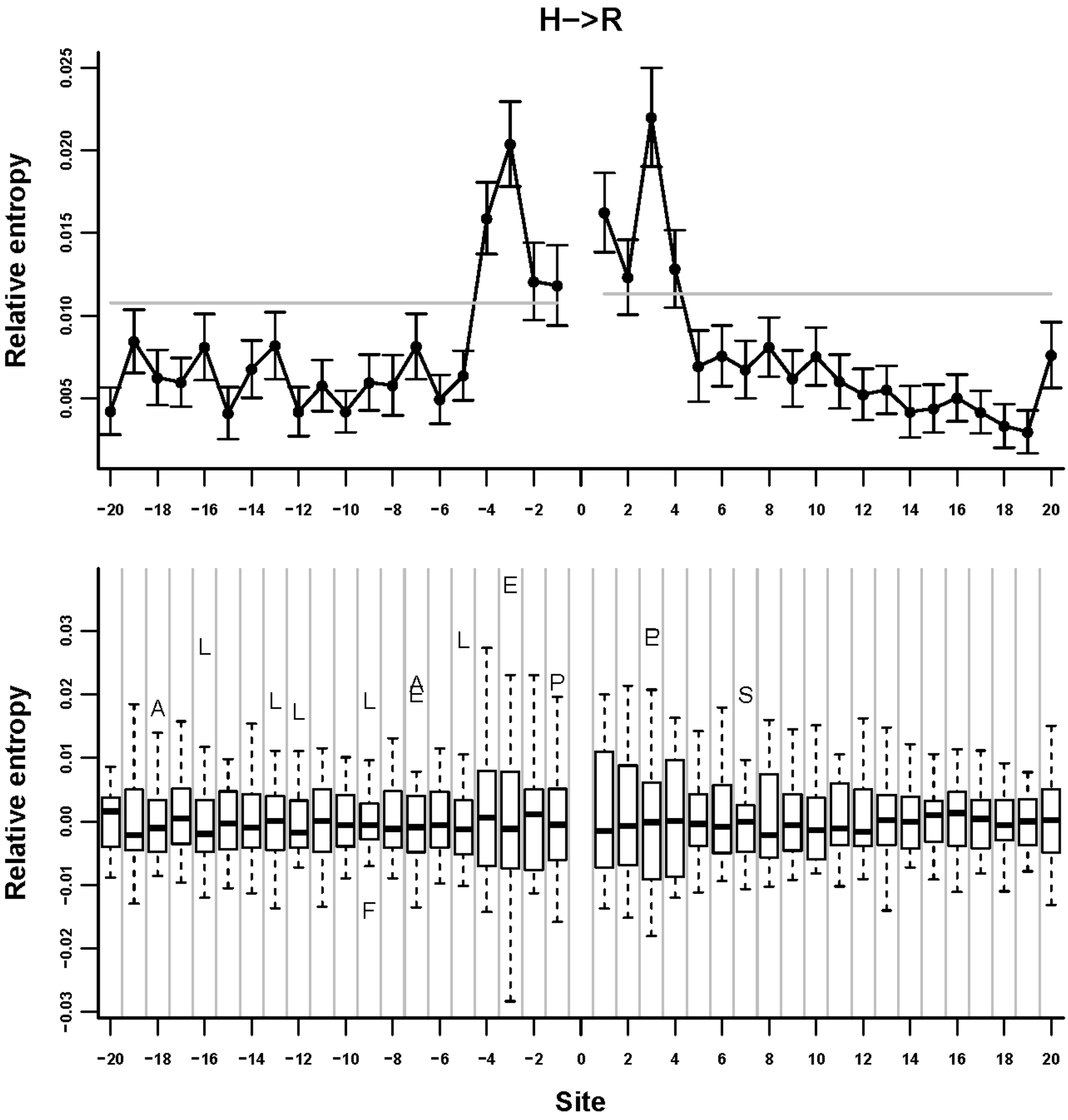
H−>R
Relative entropy
0.025
0.020
0.015
0.010
0.005
−20 −18 −16 −14 −12 −10 −8 −6 −4 −2 0 2 4 6 8 10 12 14 16 18 20
Relative entropy
0.03
0.02
0.01
0.00
−0.01
−0.02
−0.03
A L L L L A E L E P P S F
−20 −18 −16 −14 −12 −10 −8 −6 −4 −2 0 2 4 6 8 10 12 14 16 18 20
Site

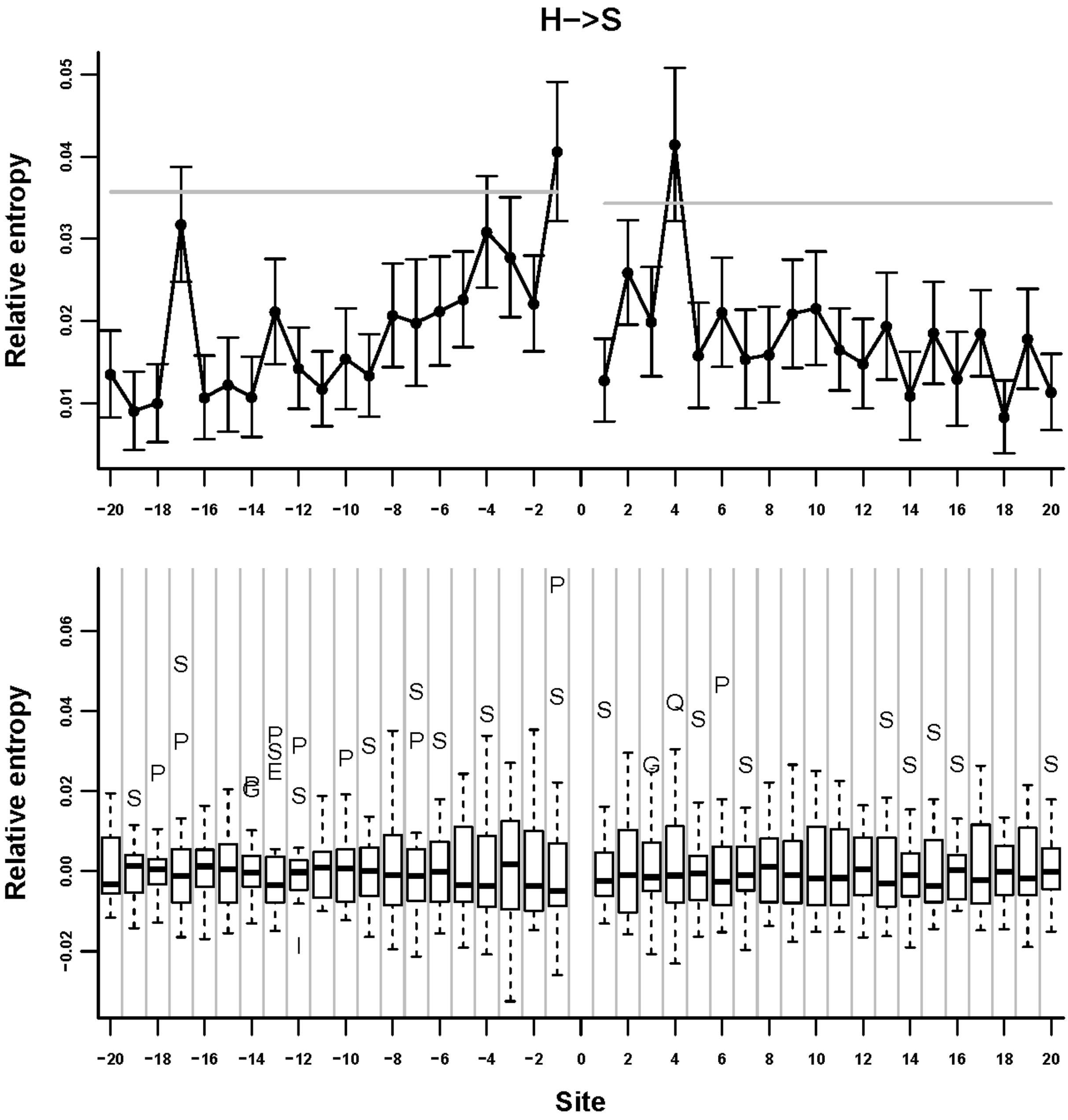
H−>S
Relative entropy
Relative entropy
Site

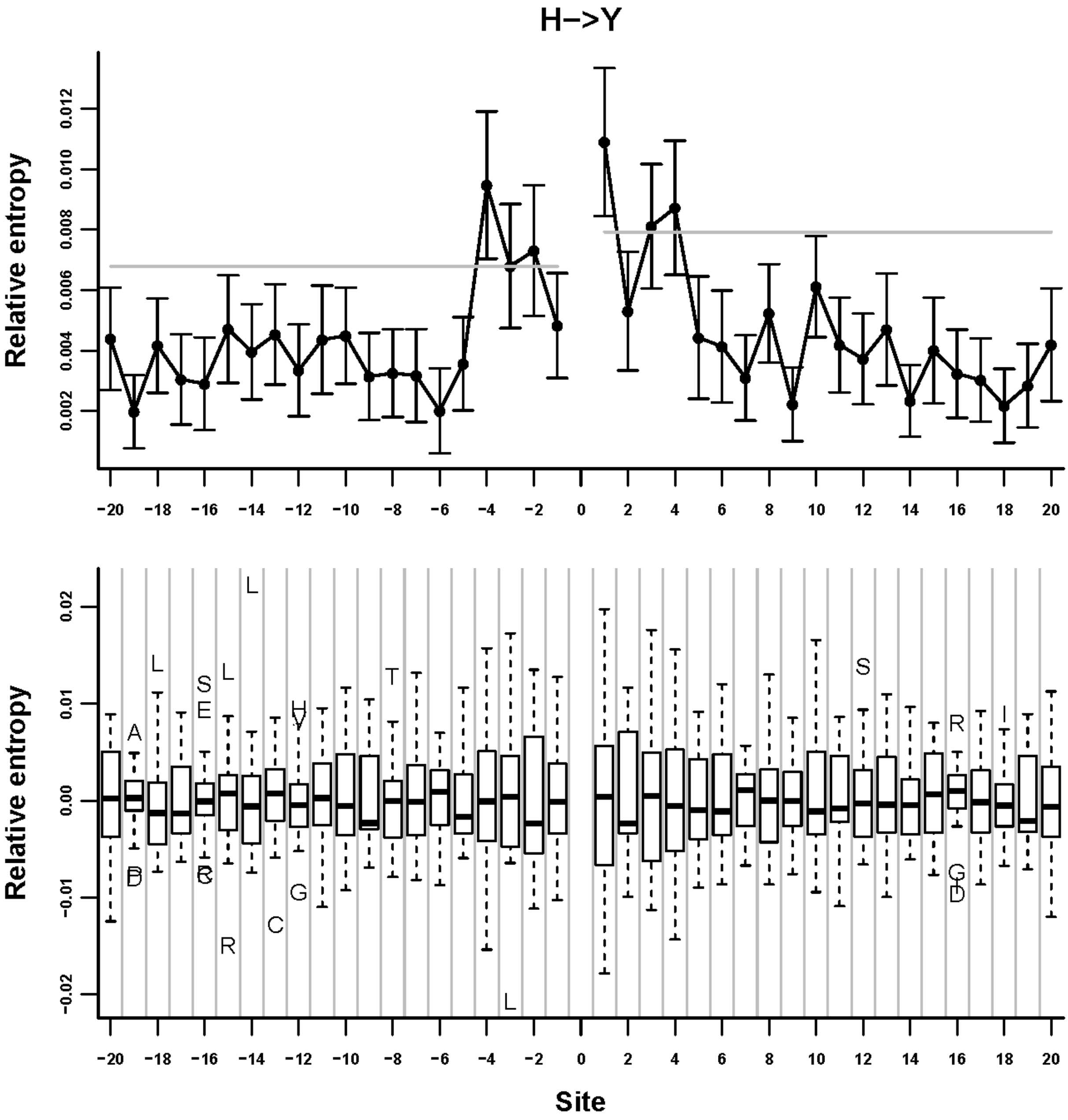
H−>Y
Relative entropy
Site

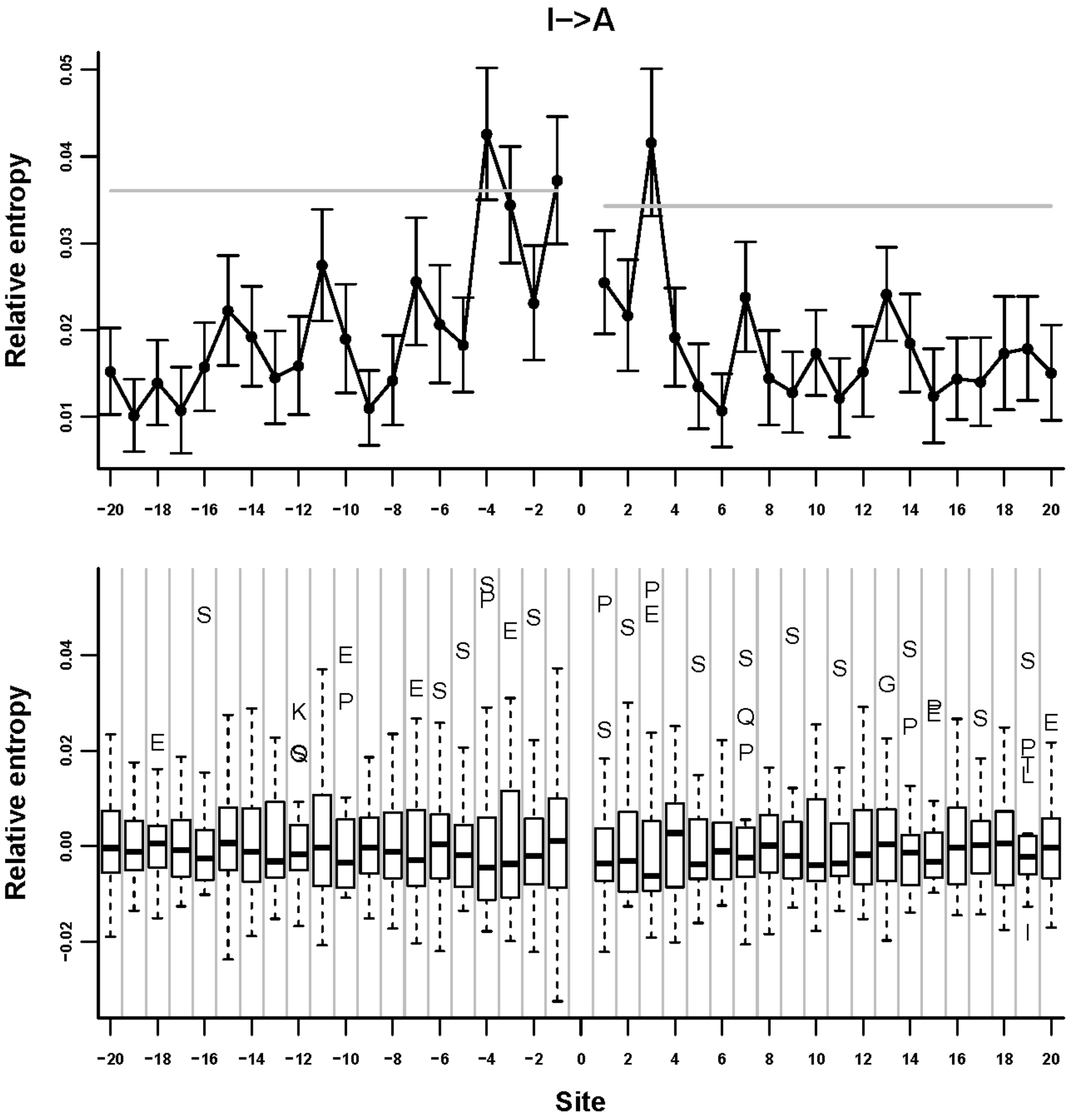
I–>A
Relative entropy
Relative entropy
Site

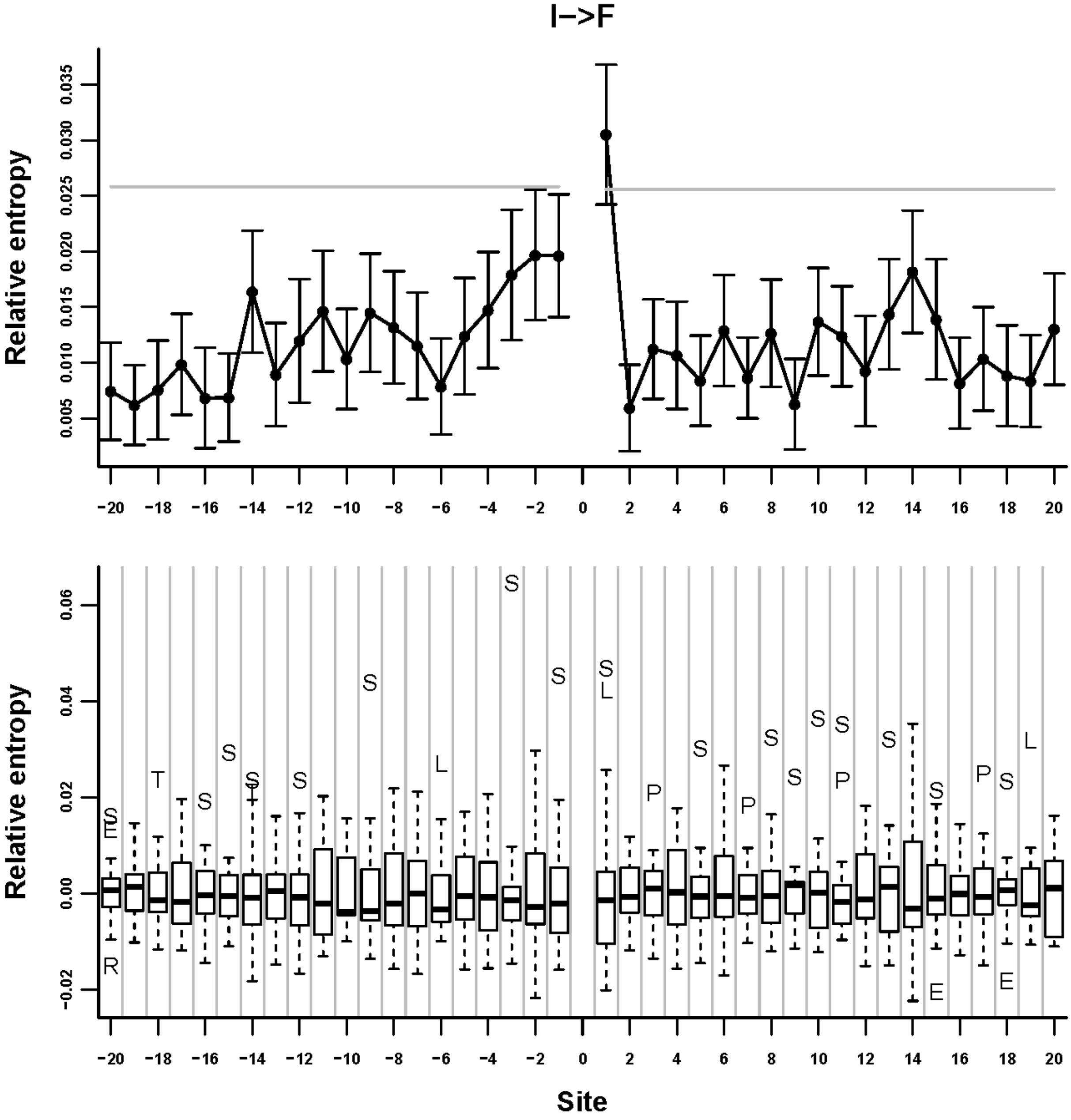
I->F
Relative entropy
0.005
0.010
0.015
0.020
0.025
0.030
0.035
−20 −18 −16 −14 −12 −10 −8 −6 −4 −2 0 2 4 6 8 10 12 14 16 18 20
Relative entropy
−0.02
0.00
0.02
0.04
0.06
Site

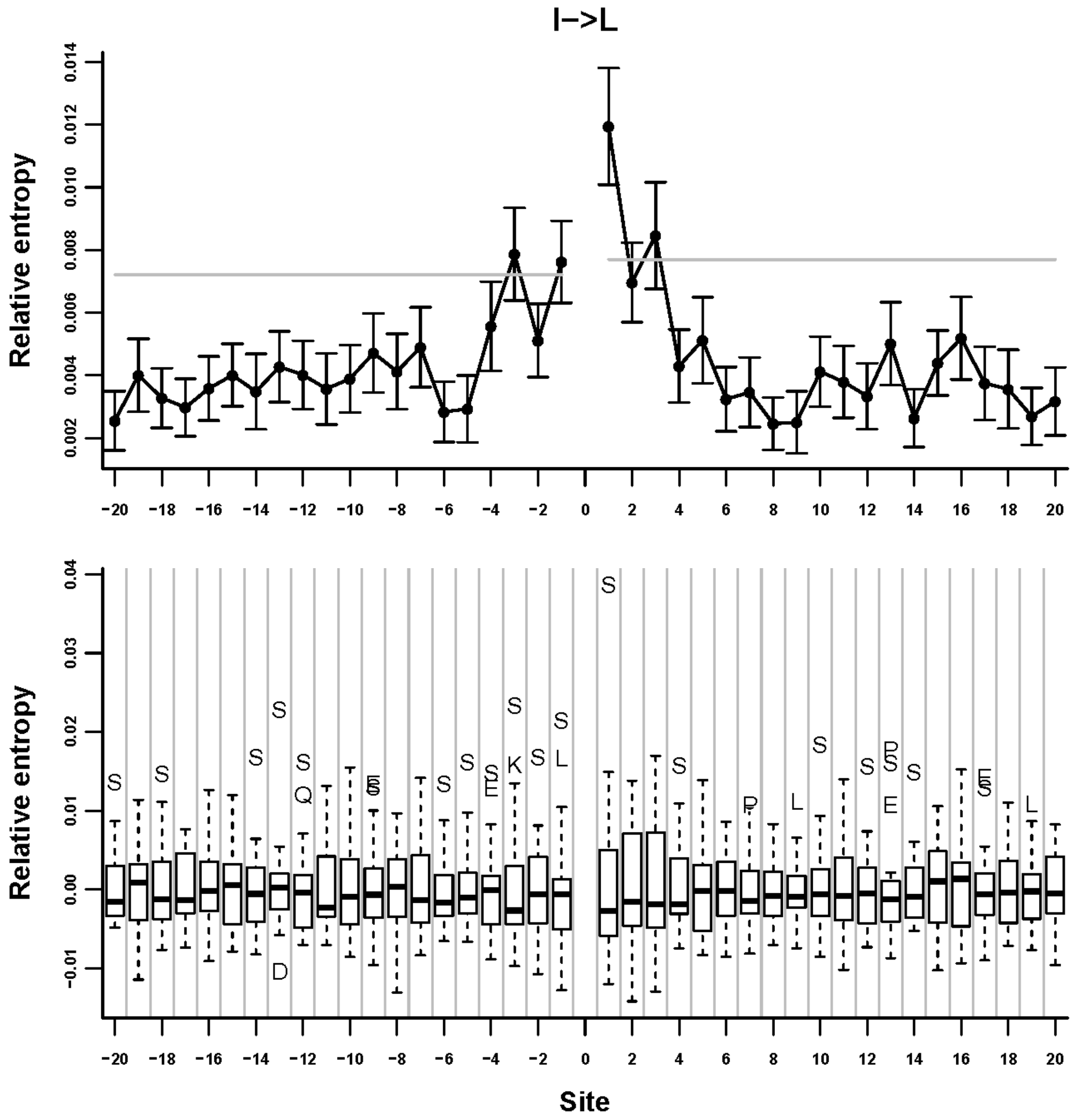
I−>L
Relative entropy
Relative entropy
Site

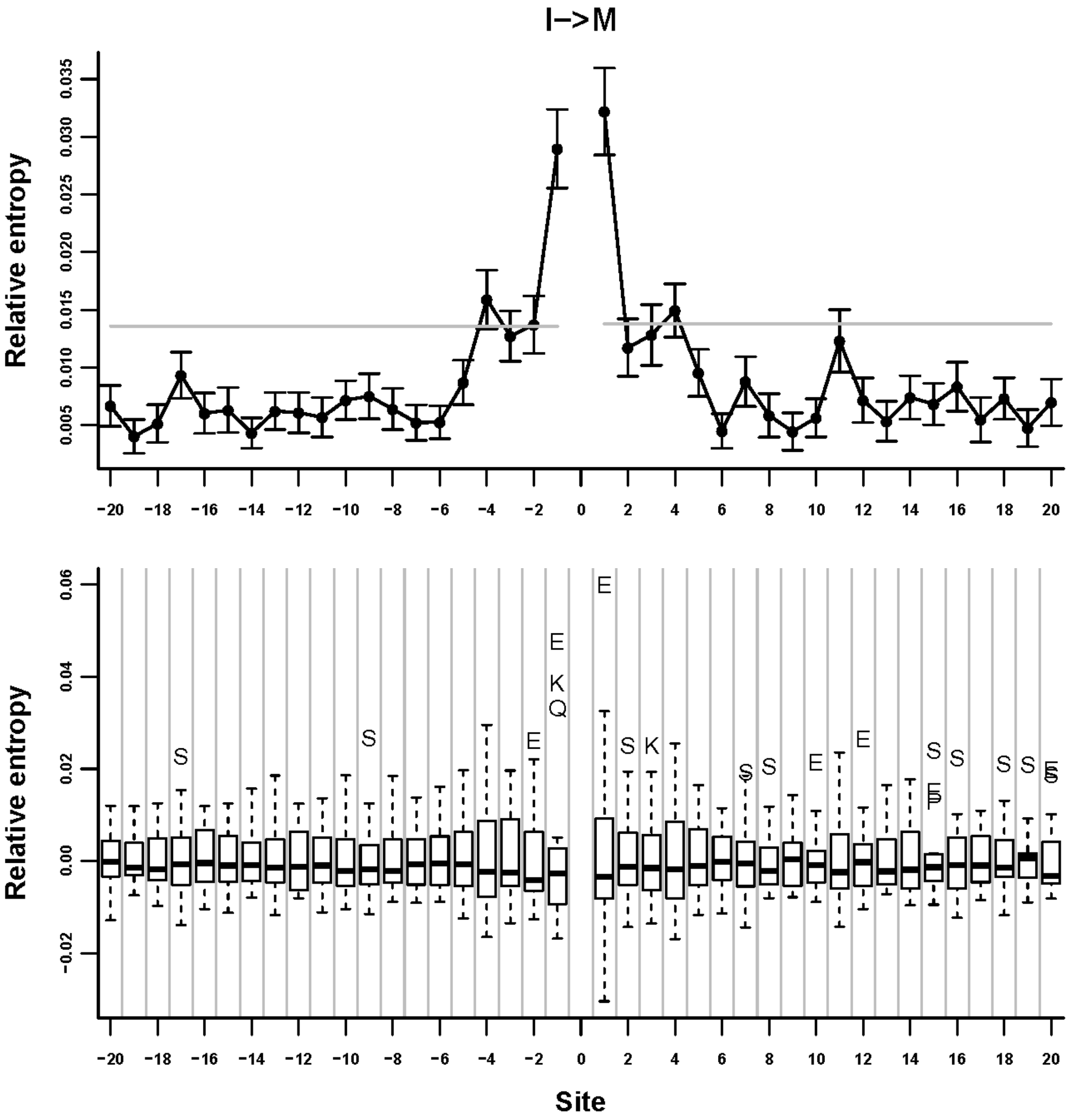
I−>M
Relative entropy
Relative entropy
Site

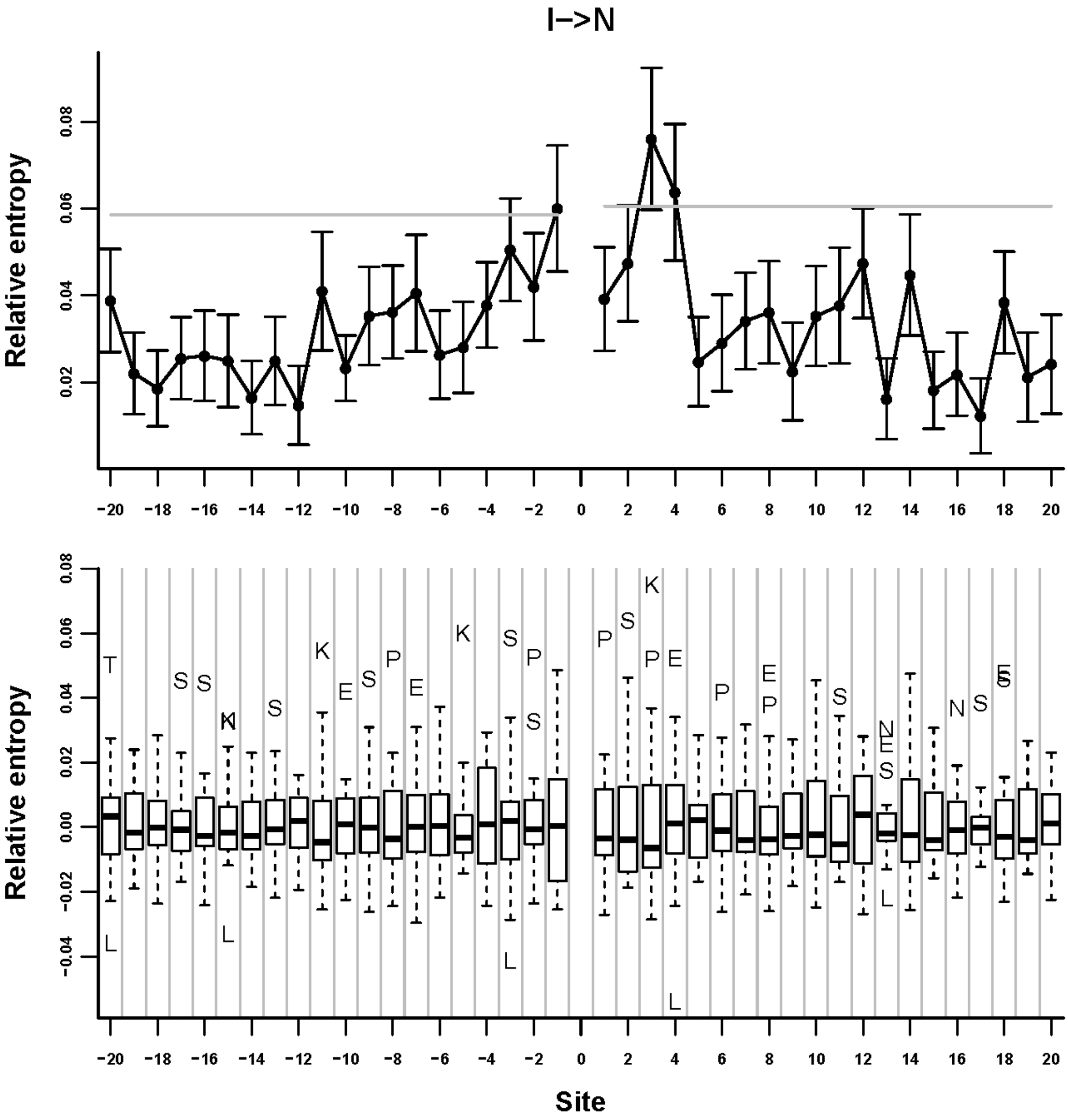
I->N
Relative entropy
Relative entropy
Site

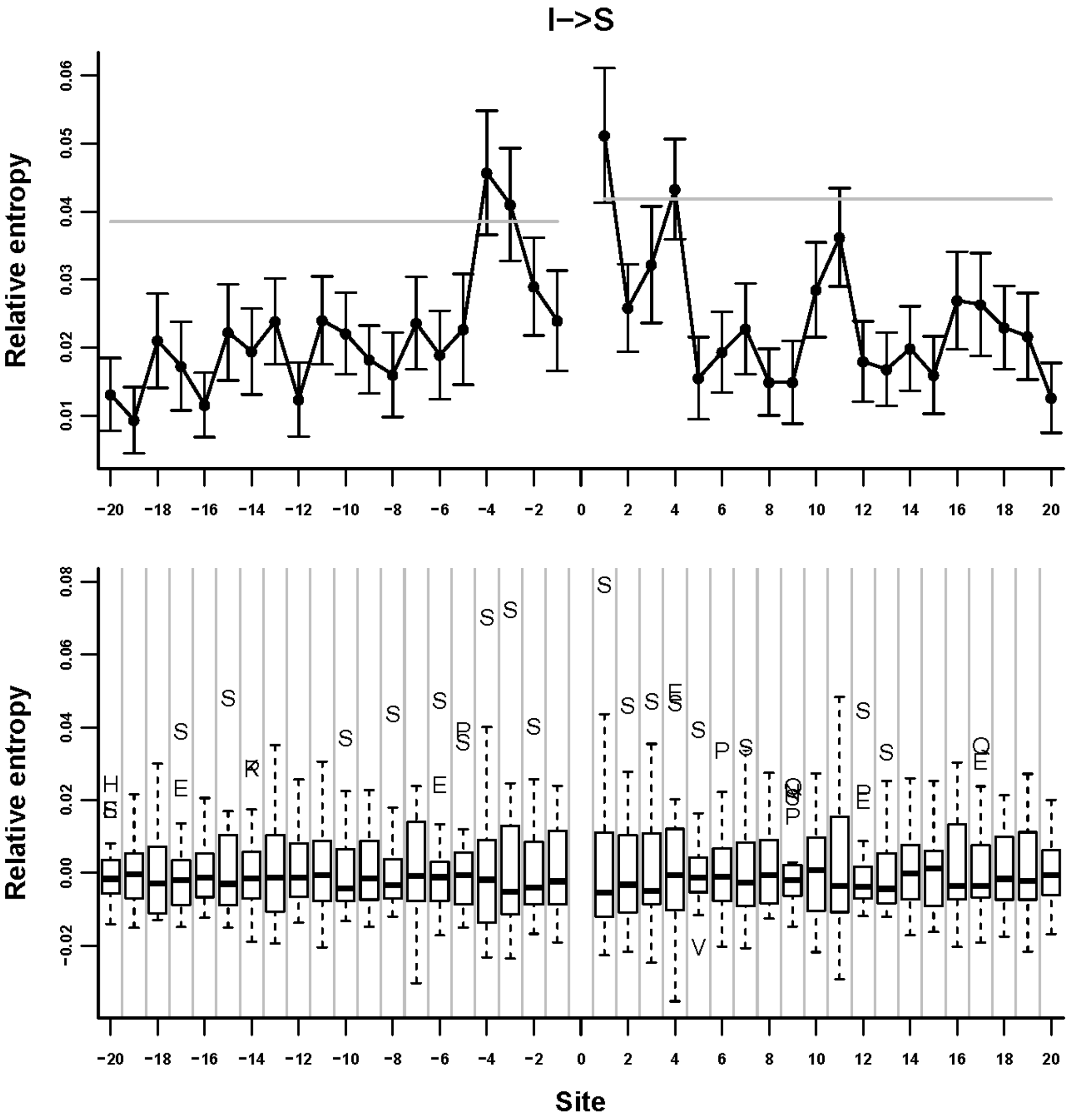
I->S
Relative entropy
Relative entropy
Site

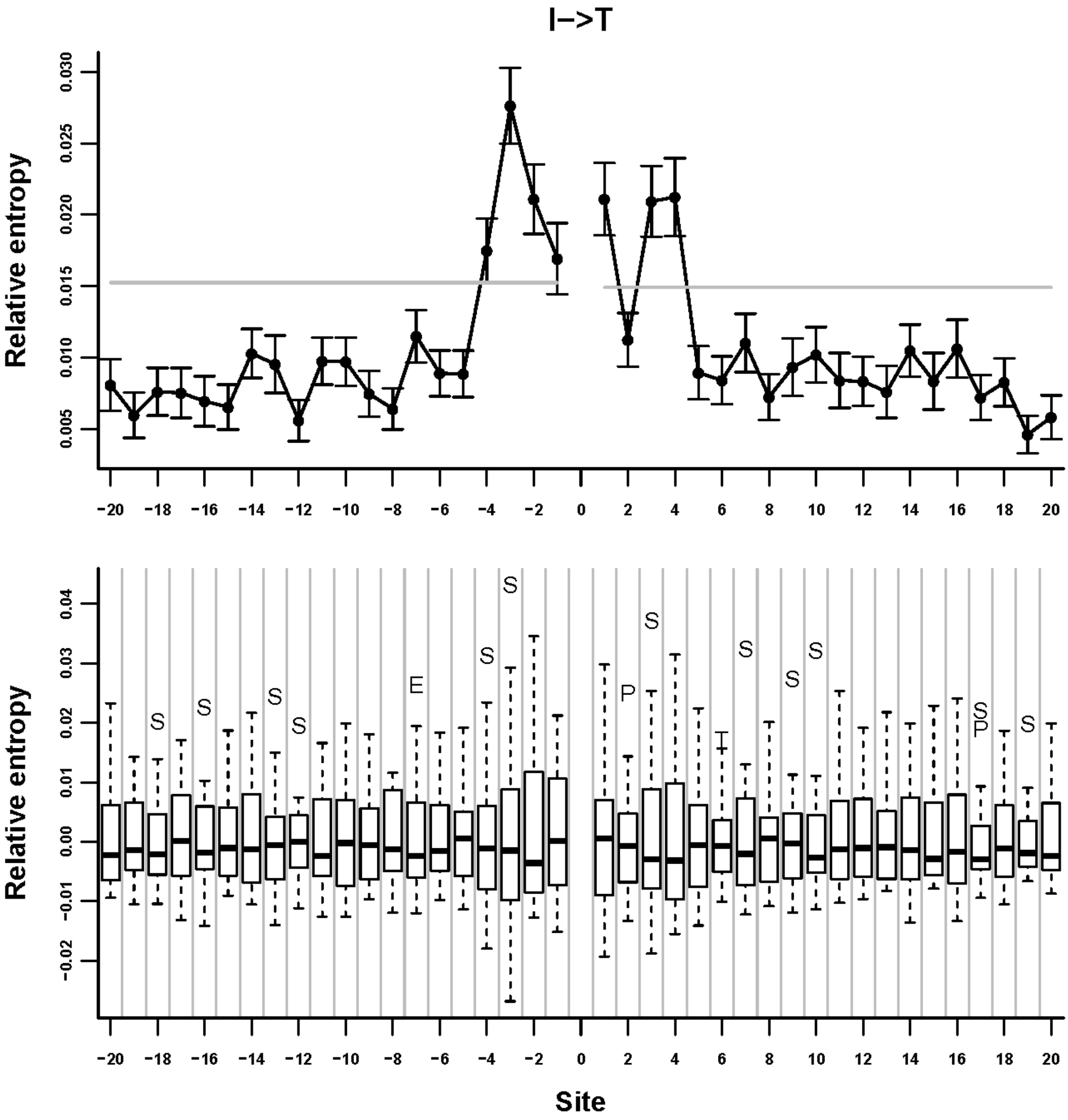
I->T
Relative entropy
Relative entropy
Site
S
S
S
S
E
S
S
P
S
S
S
S
S
P
S

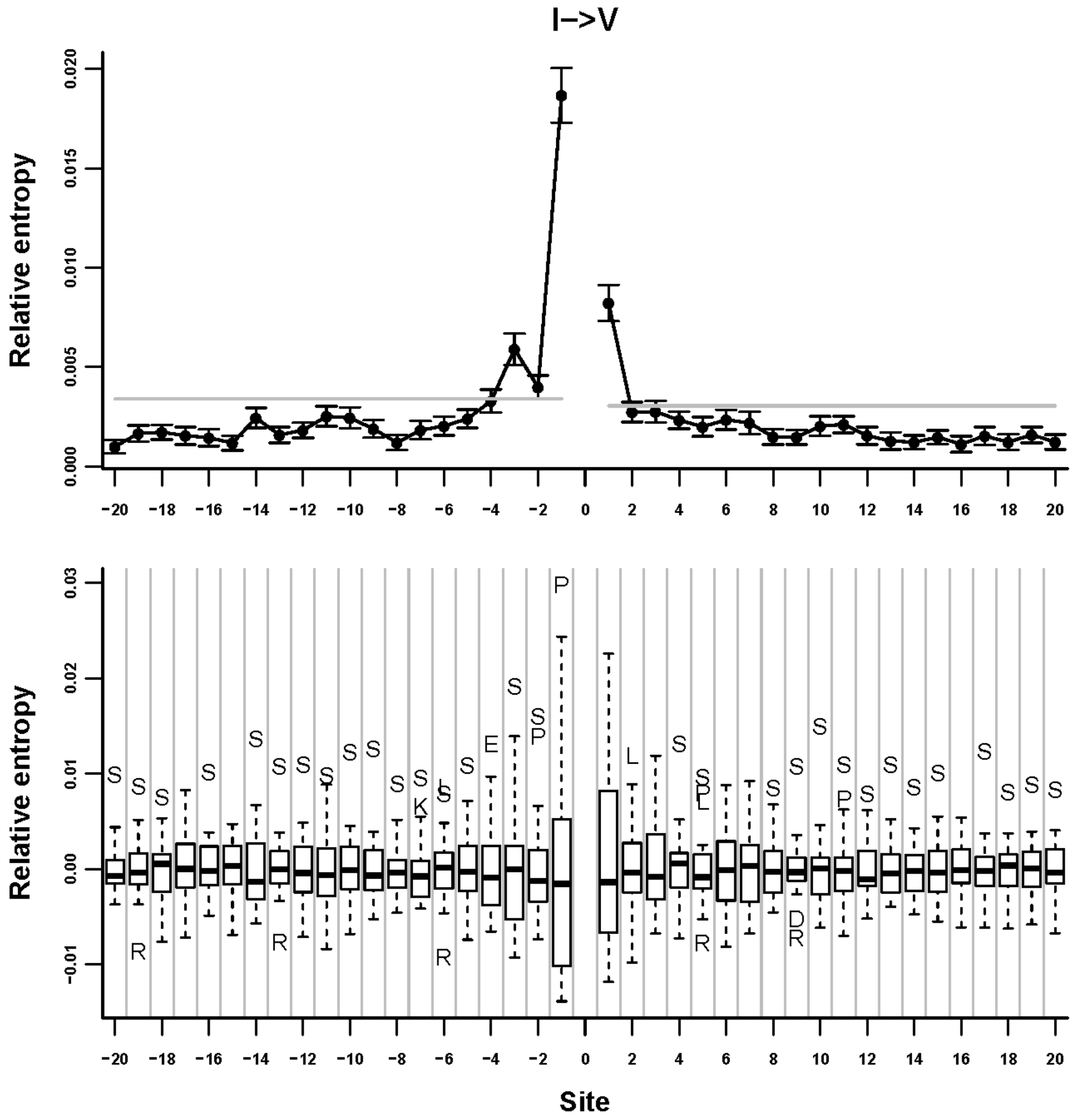
I->V
Relative entropy
Relative entropy
Site

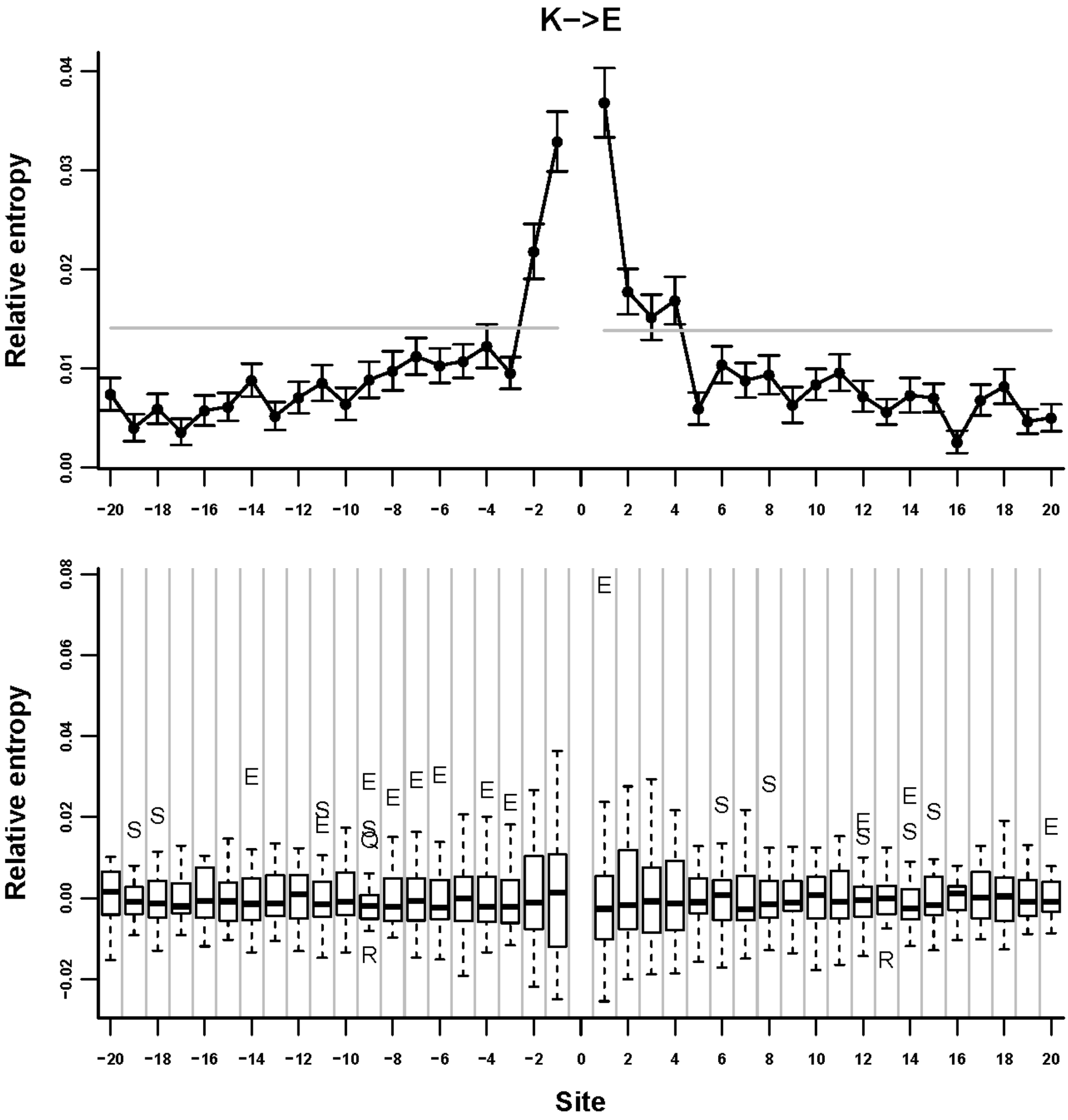

K−>E
Relative entropy
0.00
0.01
0.02
0.03
0.04
−20
−18
−16
−14
−12
−10
−8
−6
−4
−2
0
2
4
6
8
10
12
14
16
18
20
Relative entropy
−0.02
0.00
0.02
0.04
0.06
0.08
Site

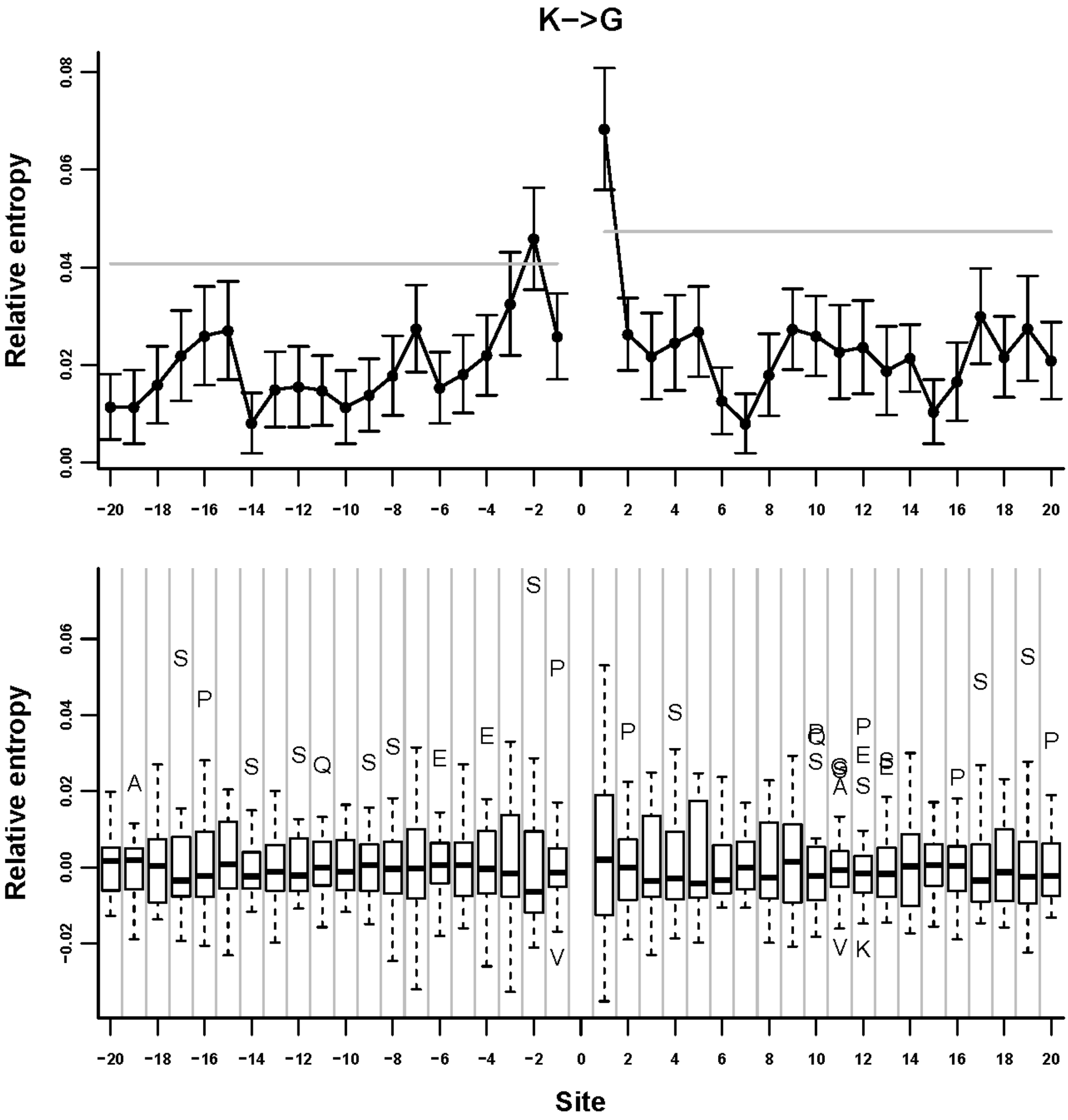
K->G
Relative entropy
Relative entropy
Site

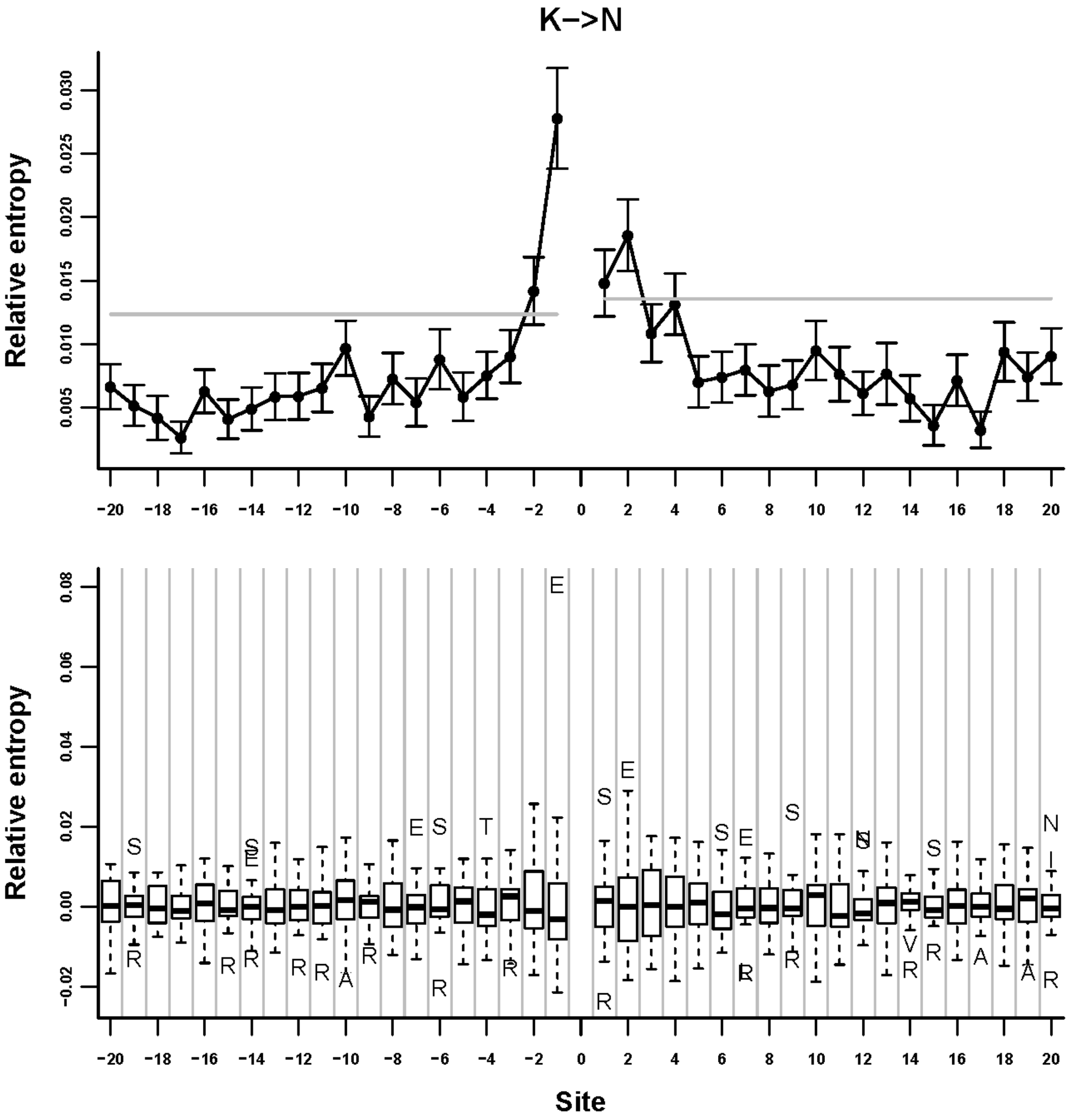
K−>N
Relative entropy
Relative entropy
Site

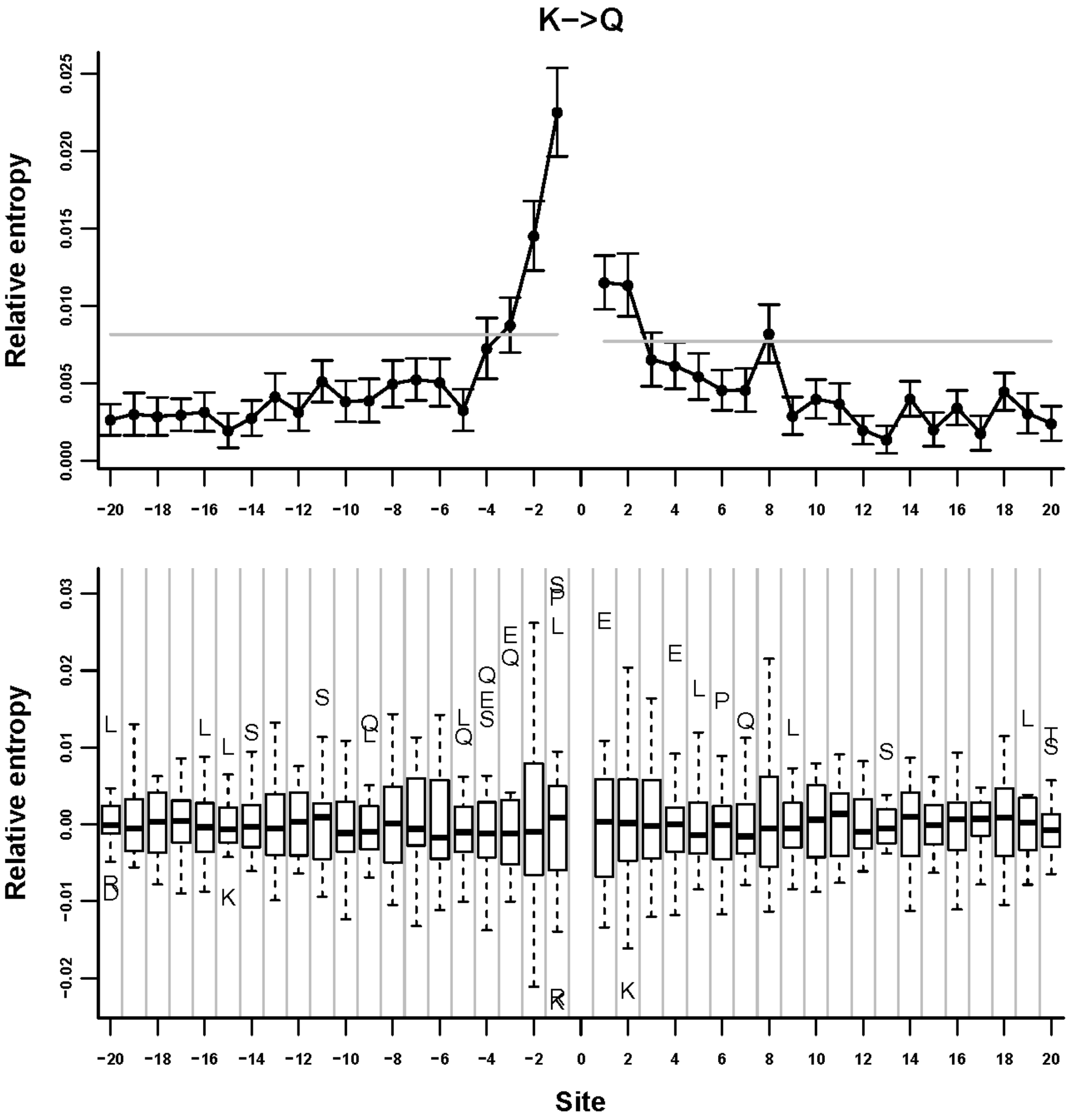
K−>Q
Relative entropy
Site

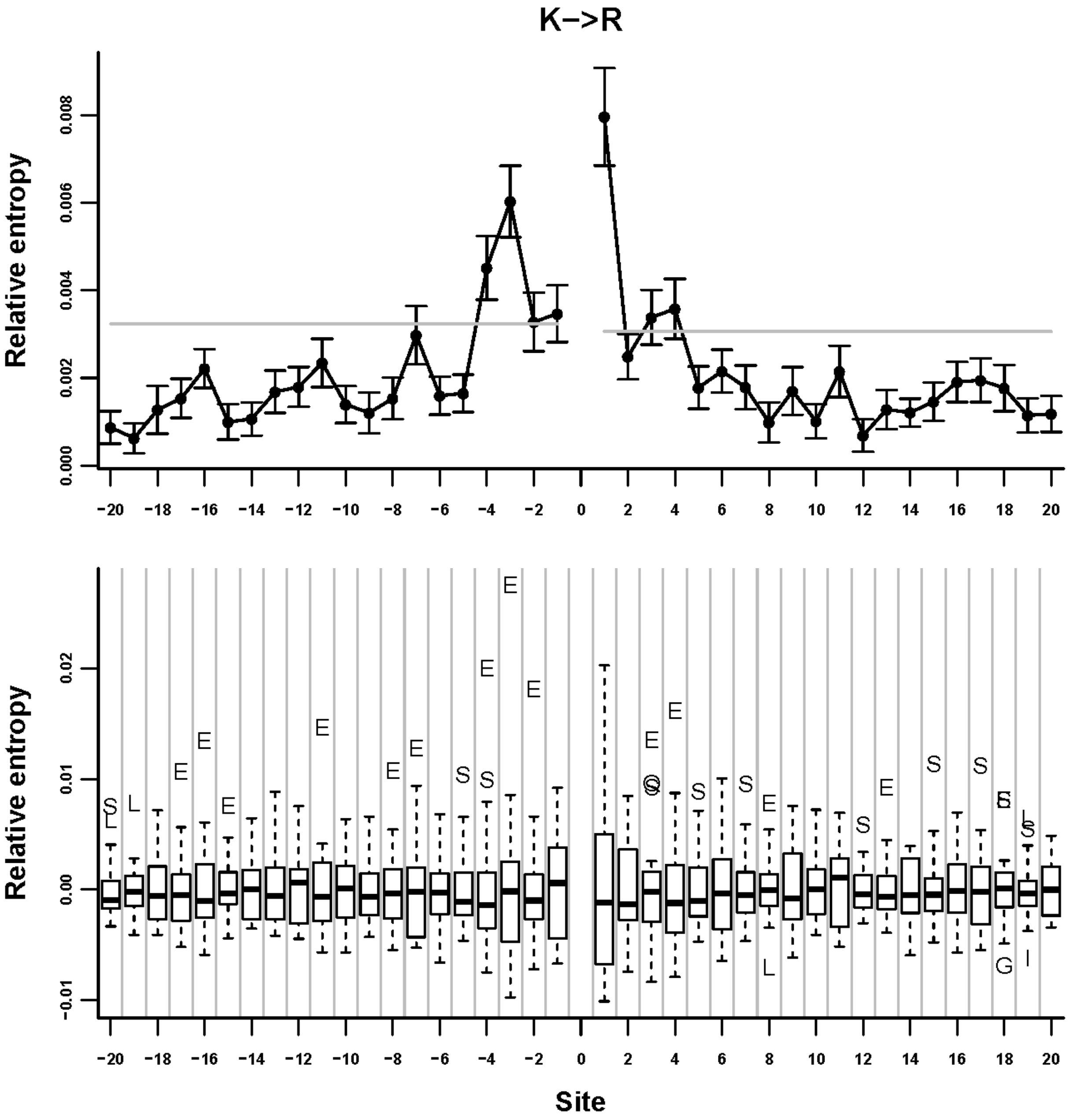
K−>R
Relative entropy
Site

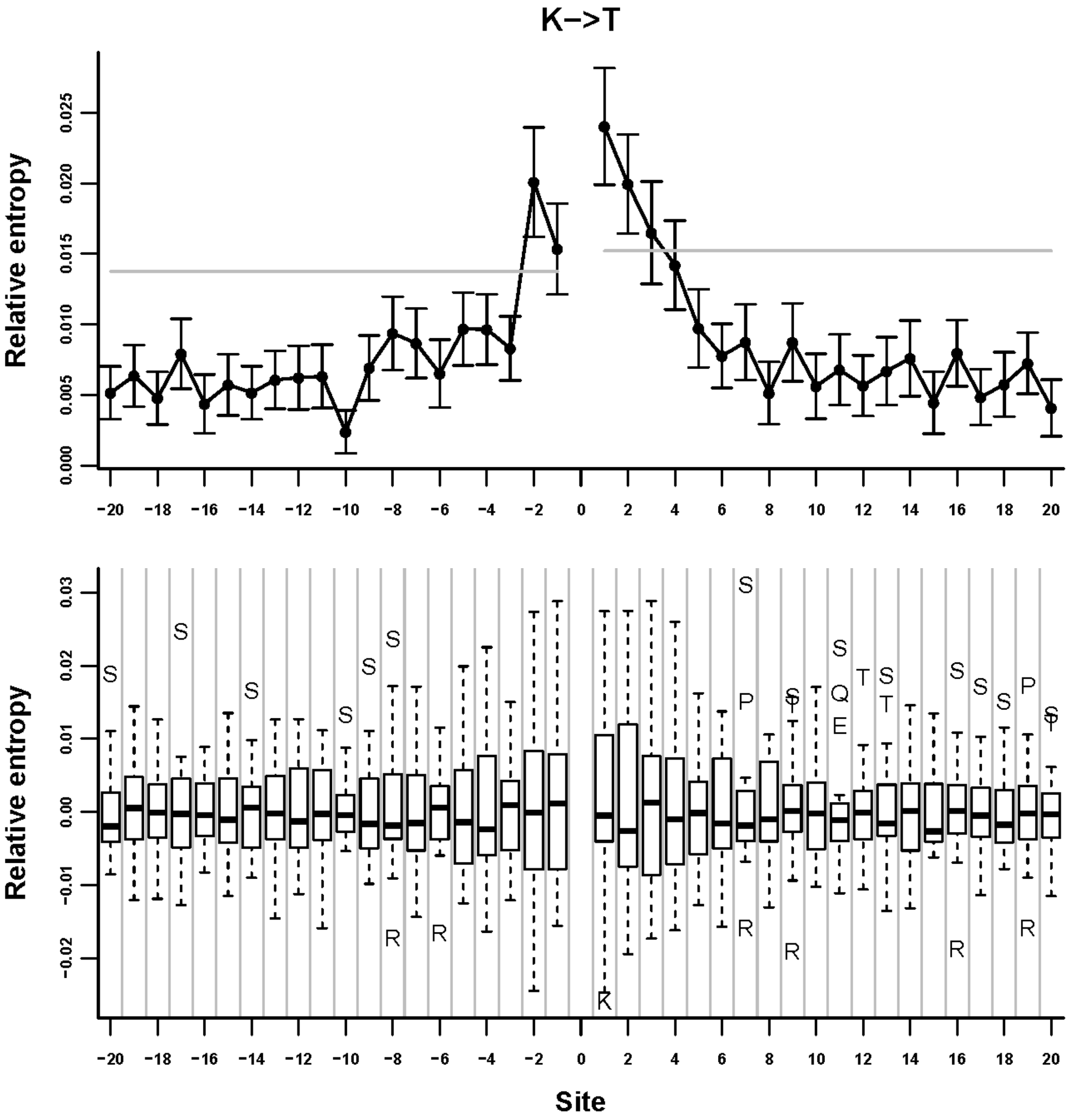
K−>T
Relative entropy
Relative entropy
Site

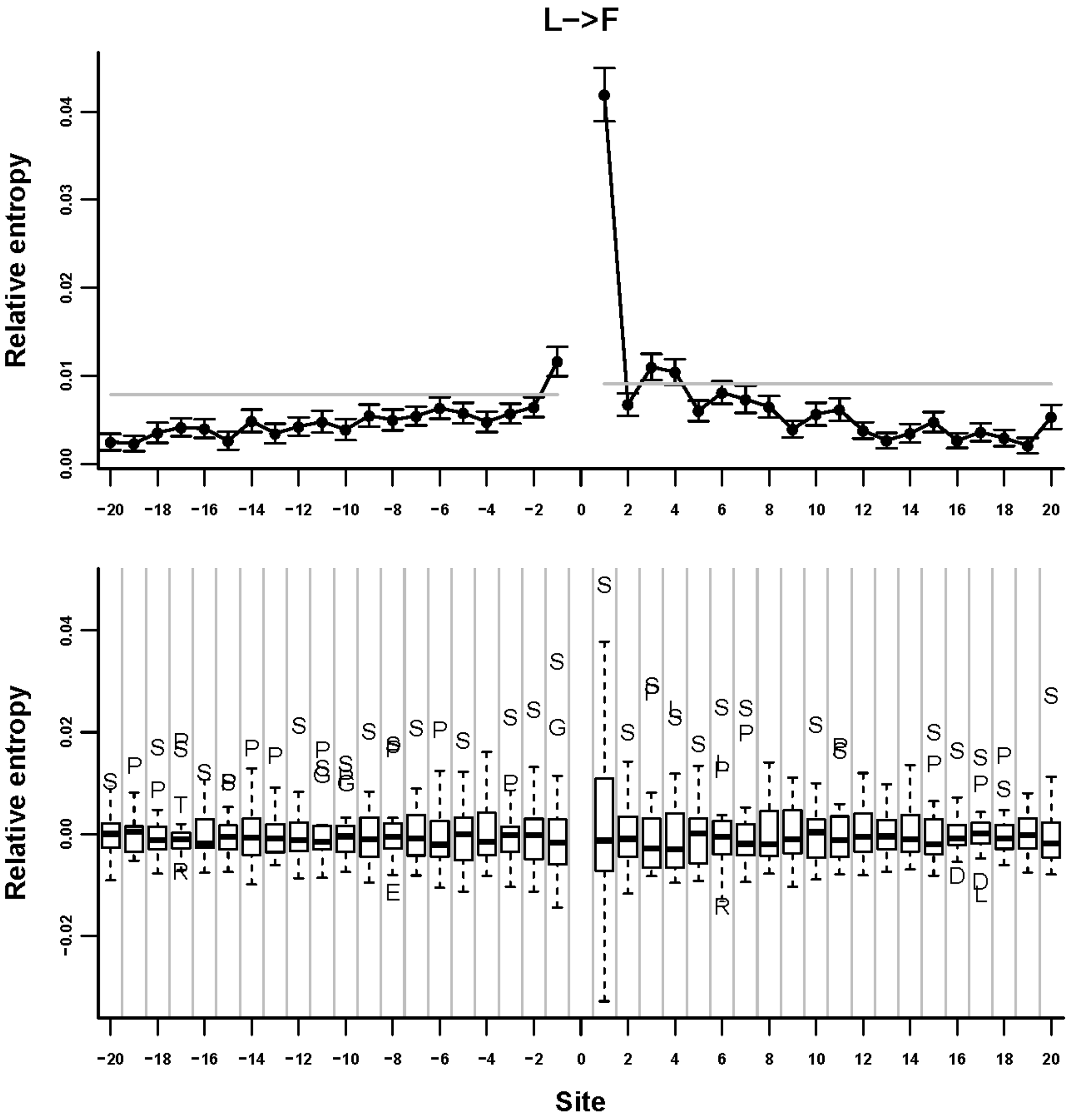
L−>F
Relative entropy
Site

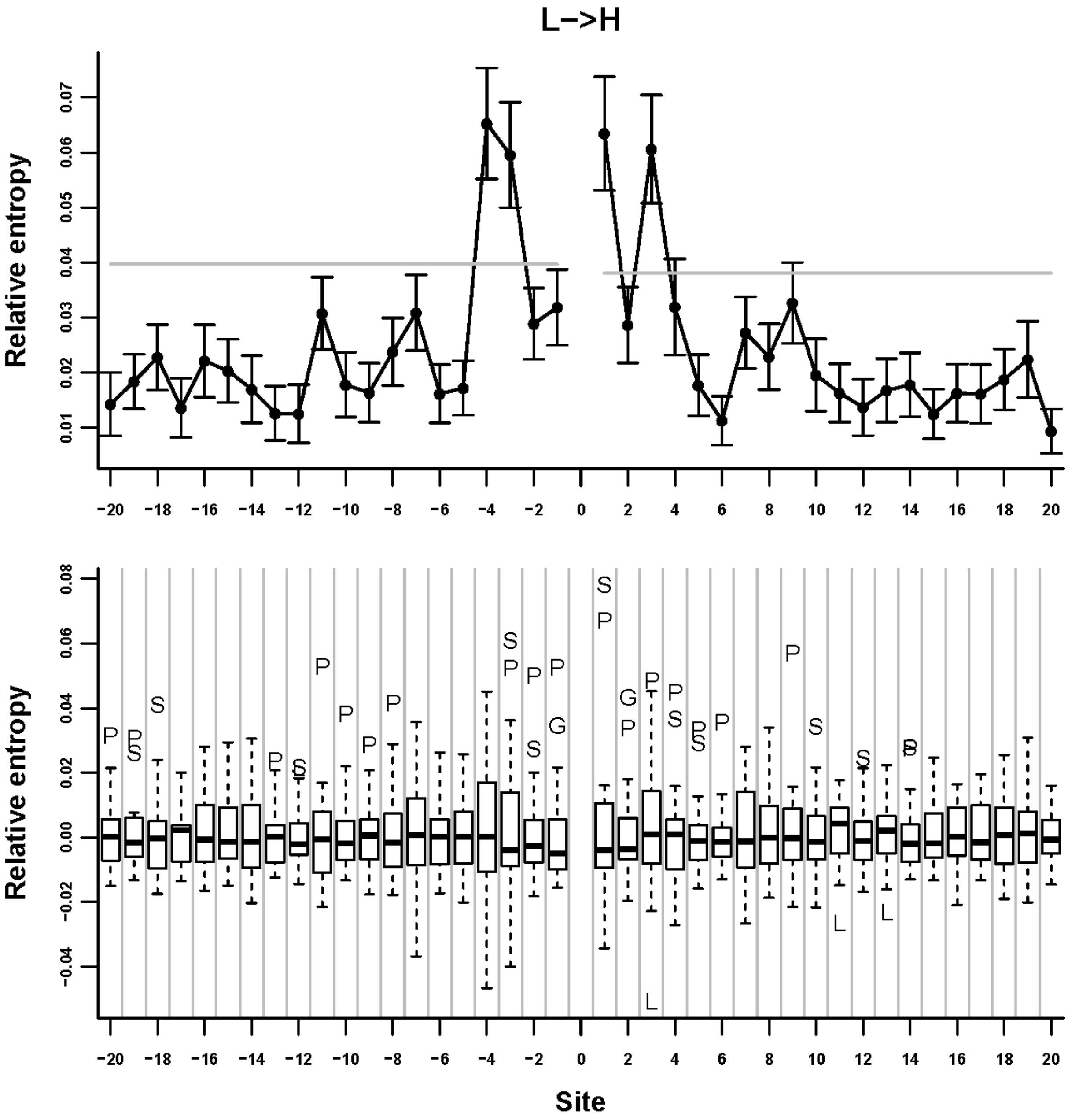
L−>H
Relative entropy
Relative entropy
Site

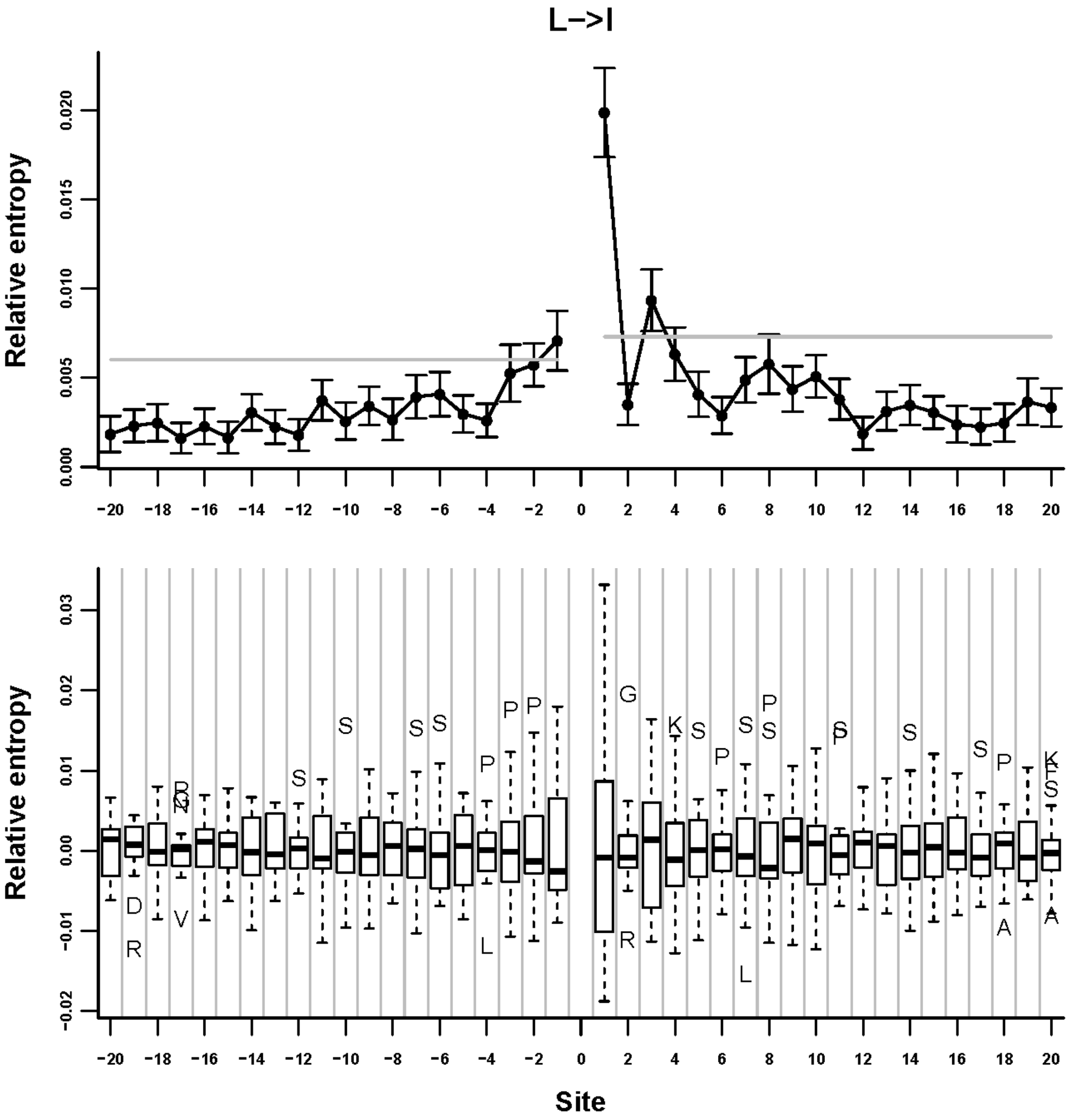
L−>I
Relative entropy
Site

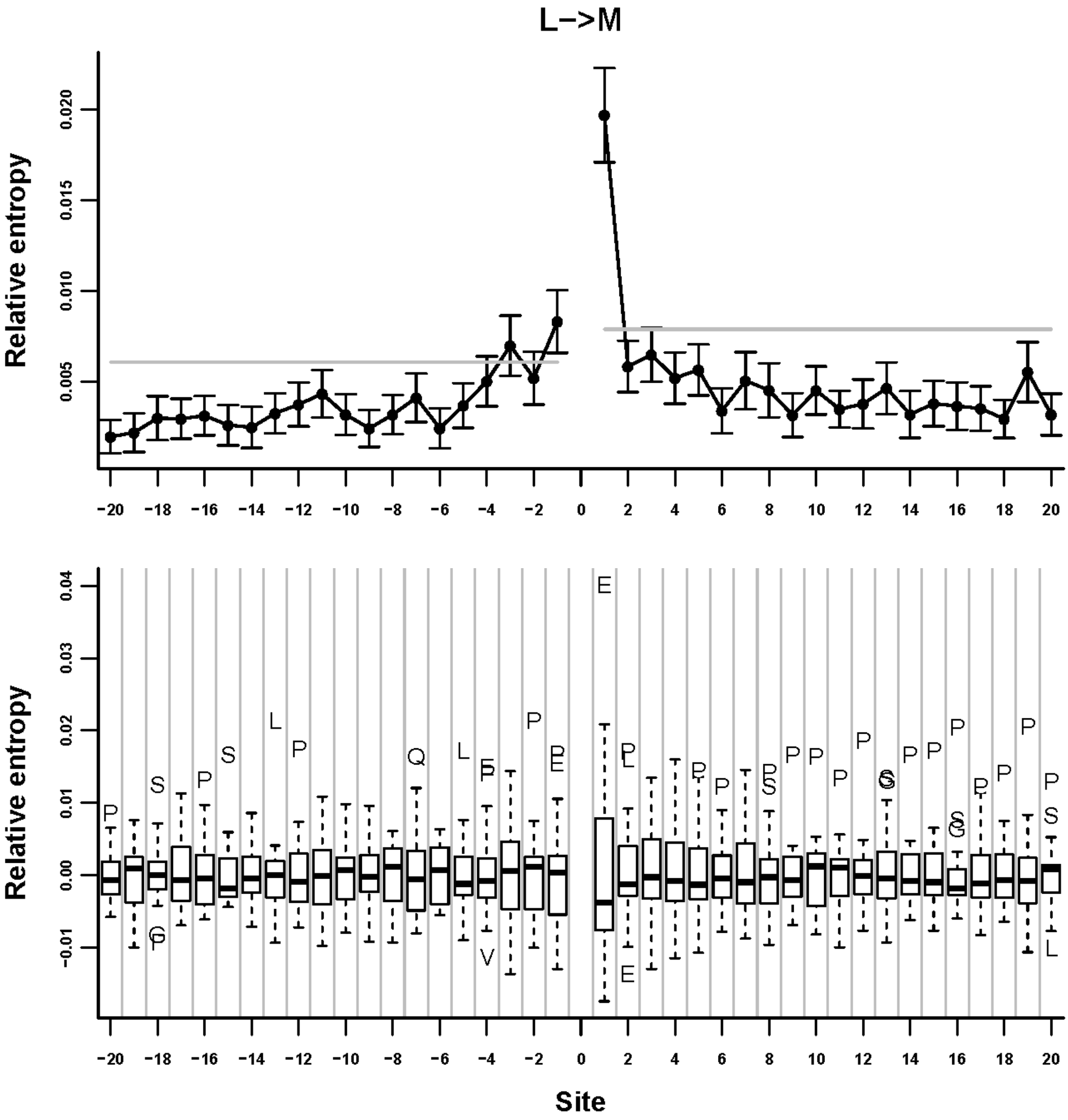
L−>M
Relative entropy
Relative entropy
Site

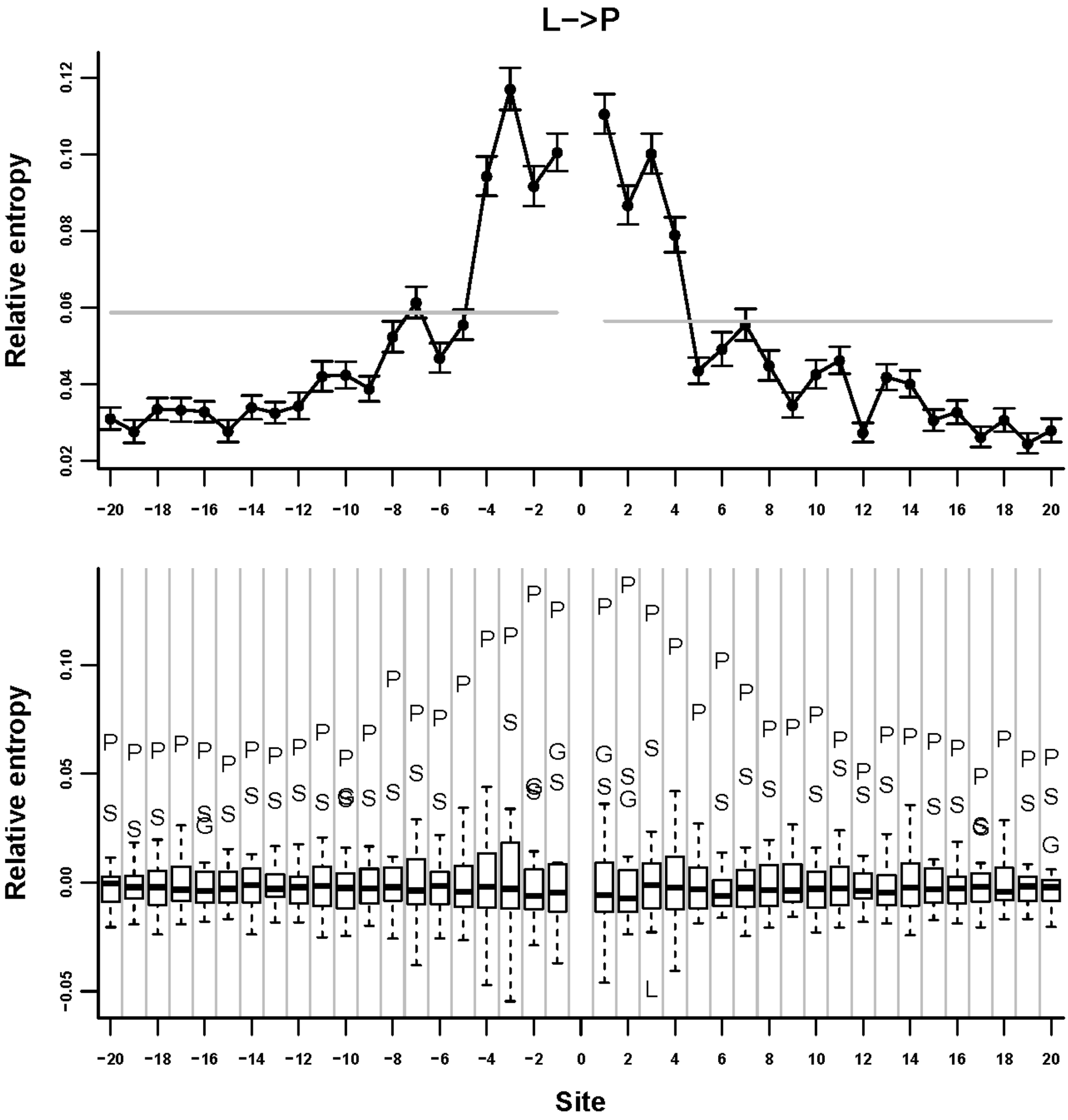
L->P
Relative entropy
Site

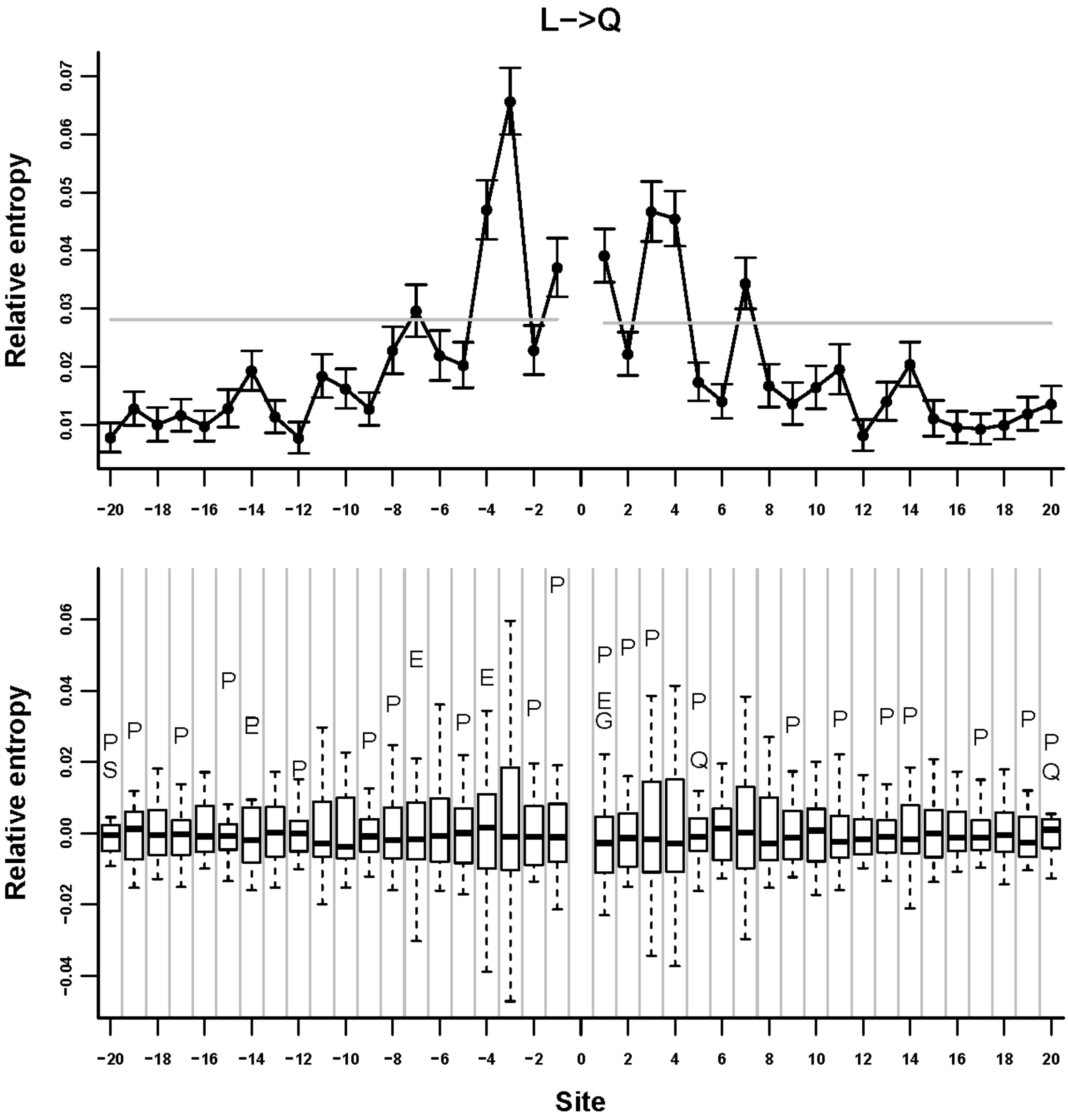
L−>Q
Relative entropy
Relative entropy
Site

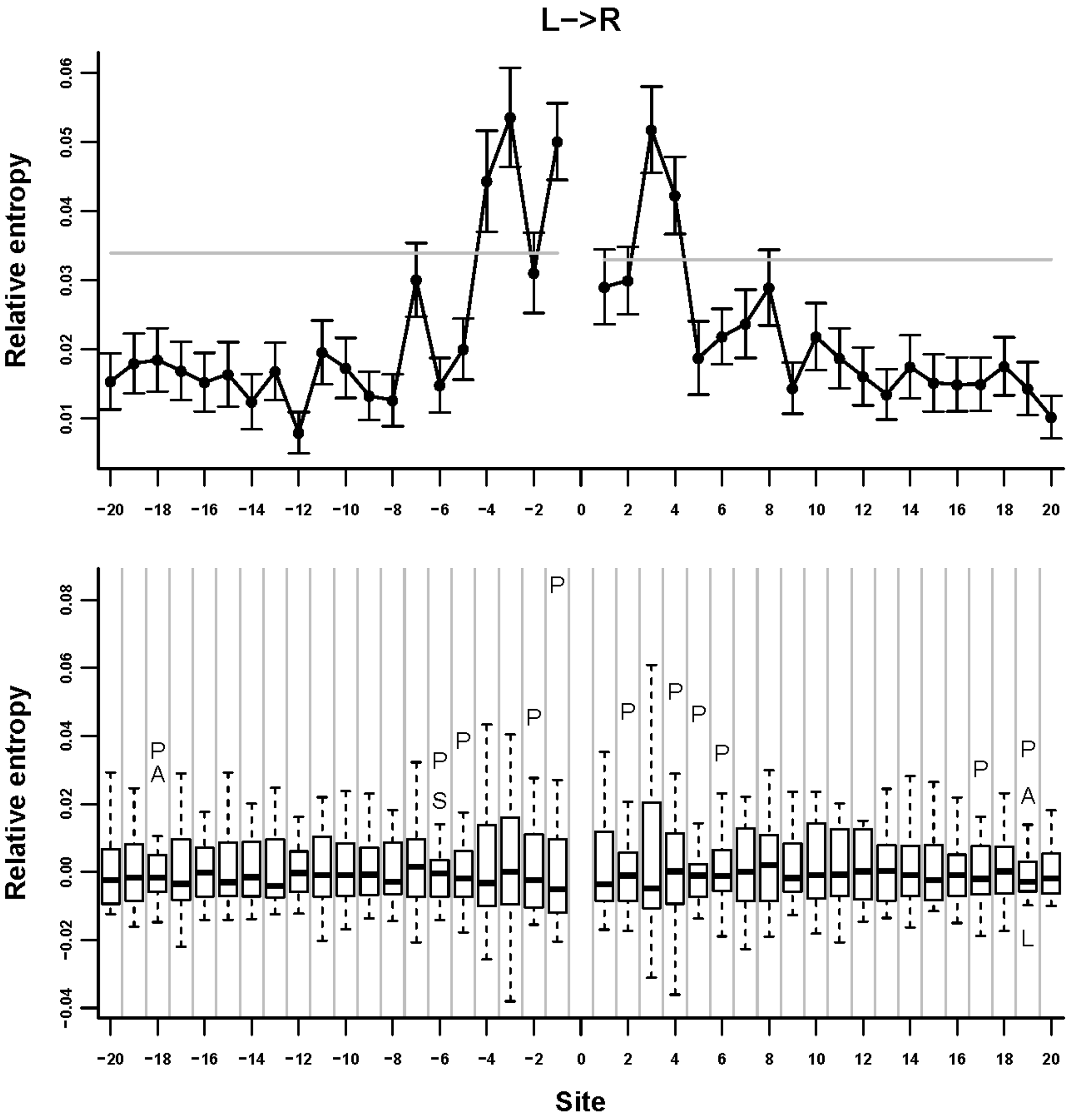

L−>R
Relative entropy
Site

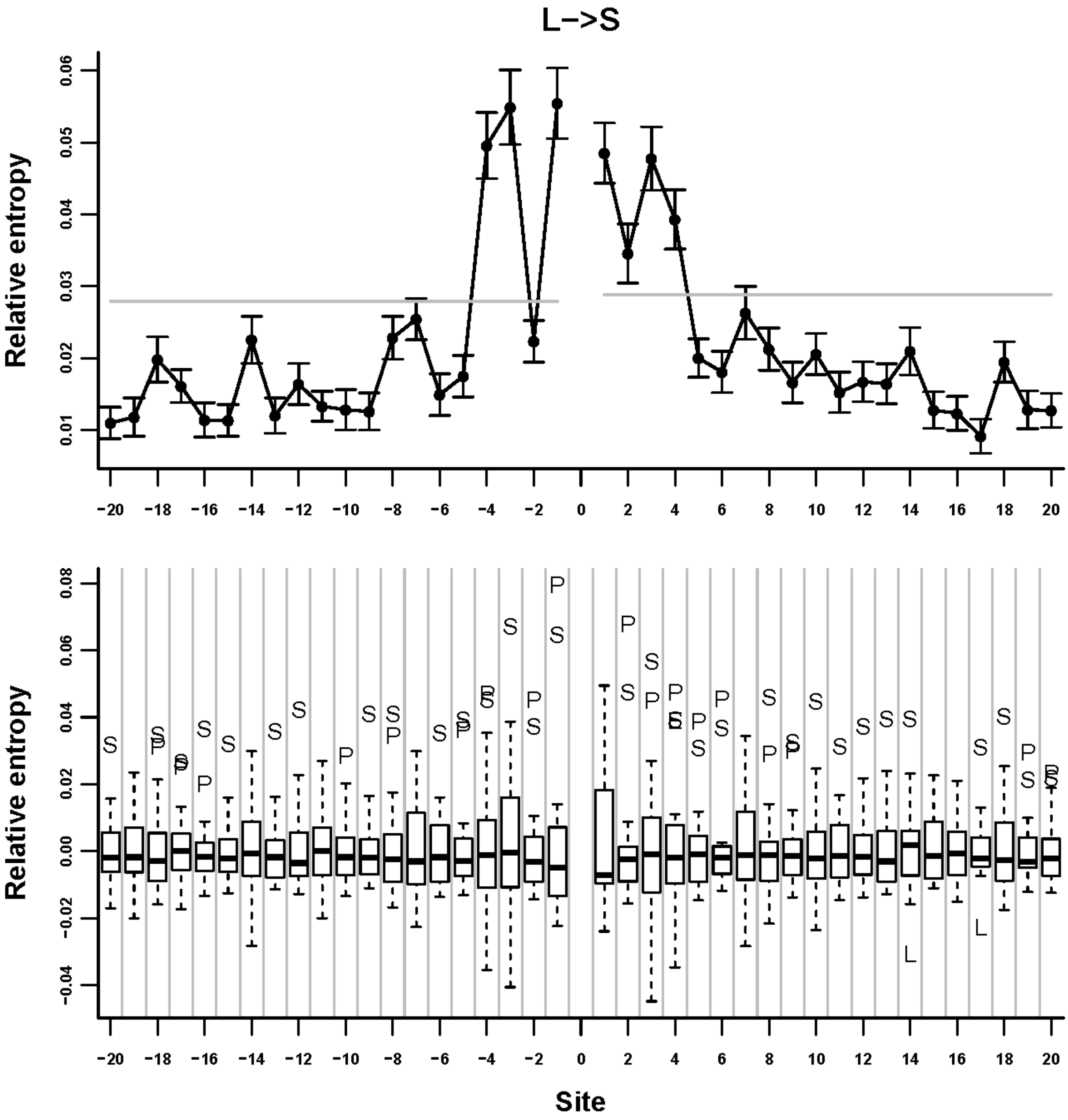
L−>S
Relative entropy
Relative entropy
Site

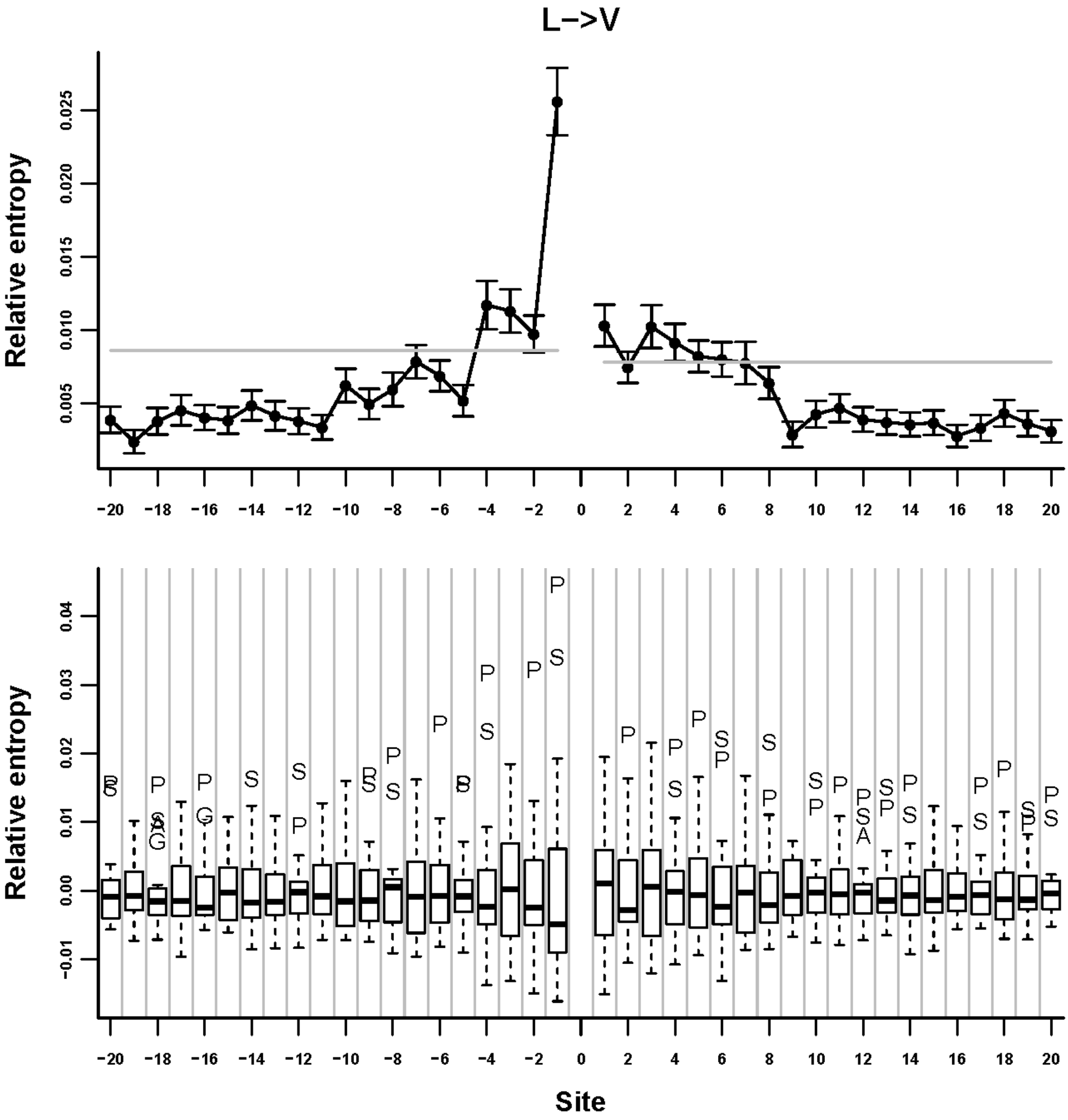
L−>V
Relative entropy
Relative entropy
Site

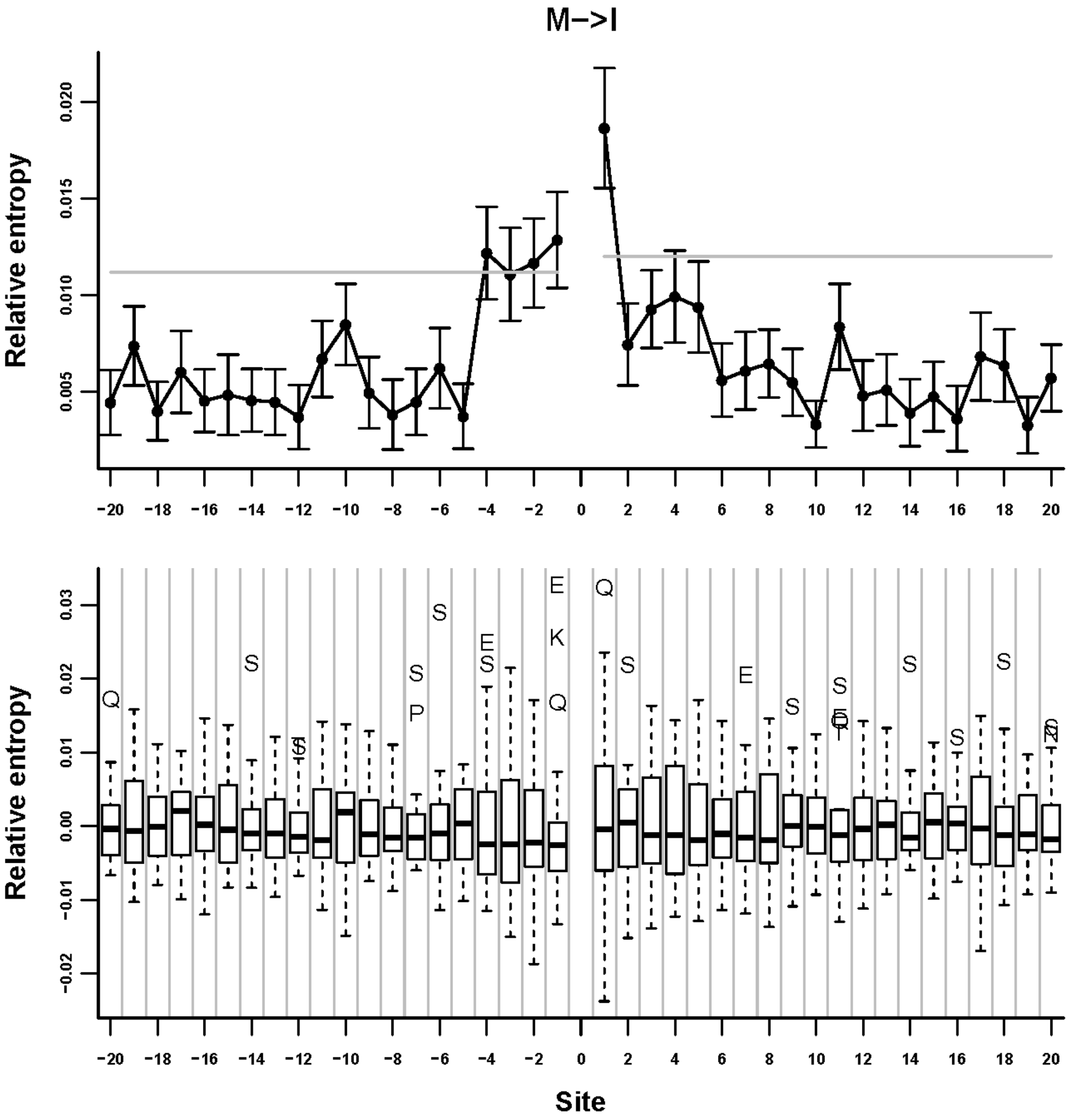
M−>I
Relative entropy
Site

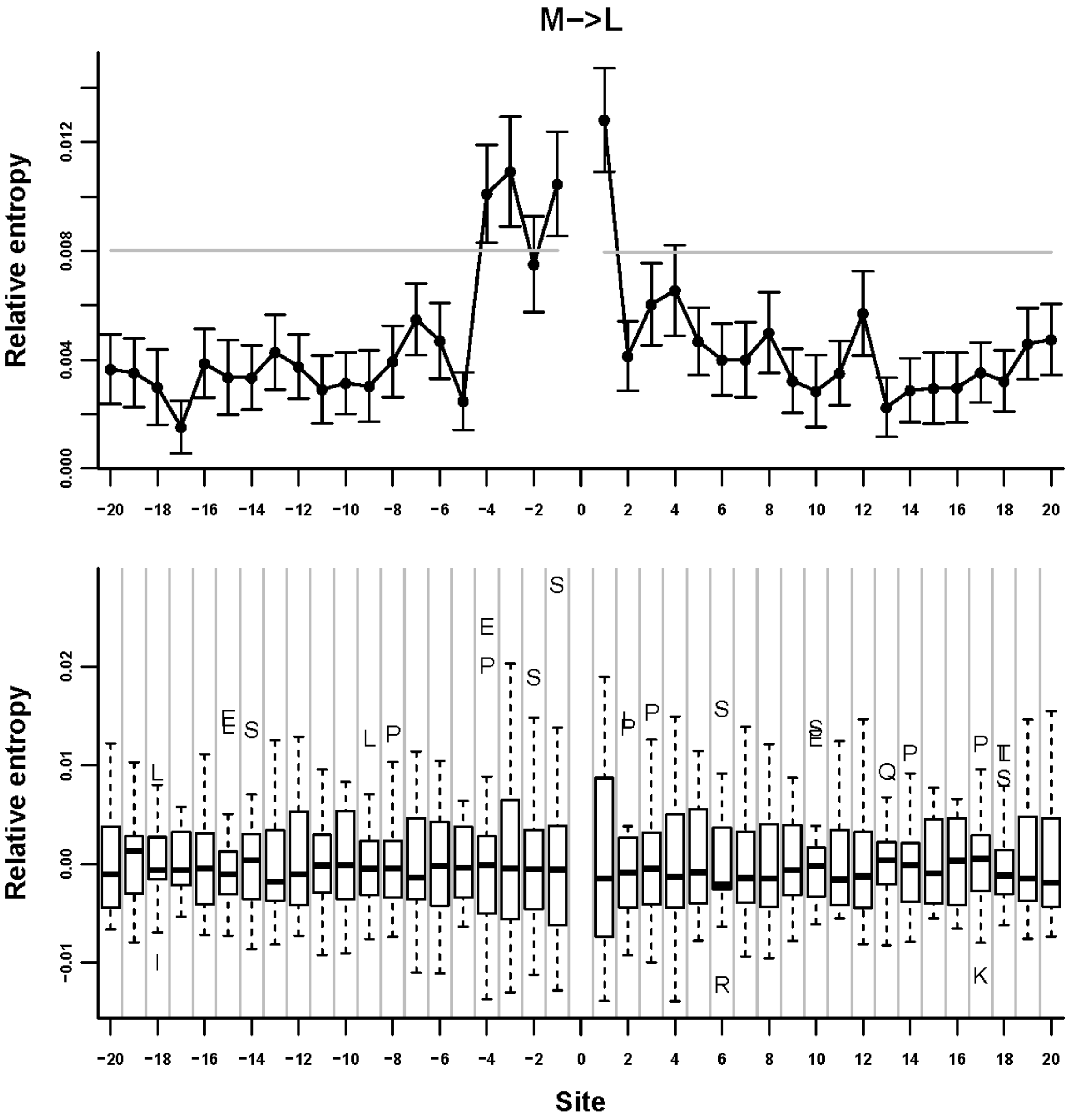
M−>L
Relative entropy
Relative entropy
Site

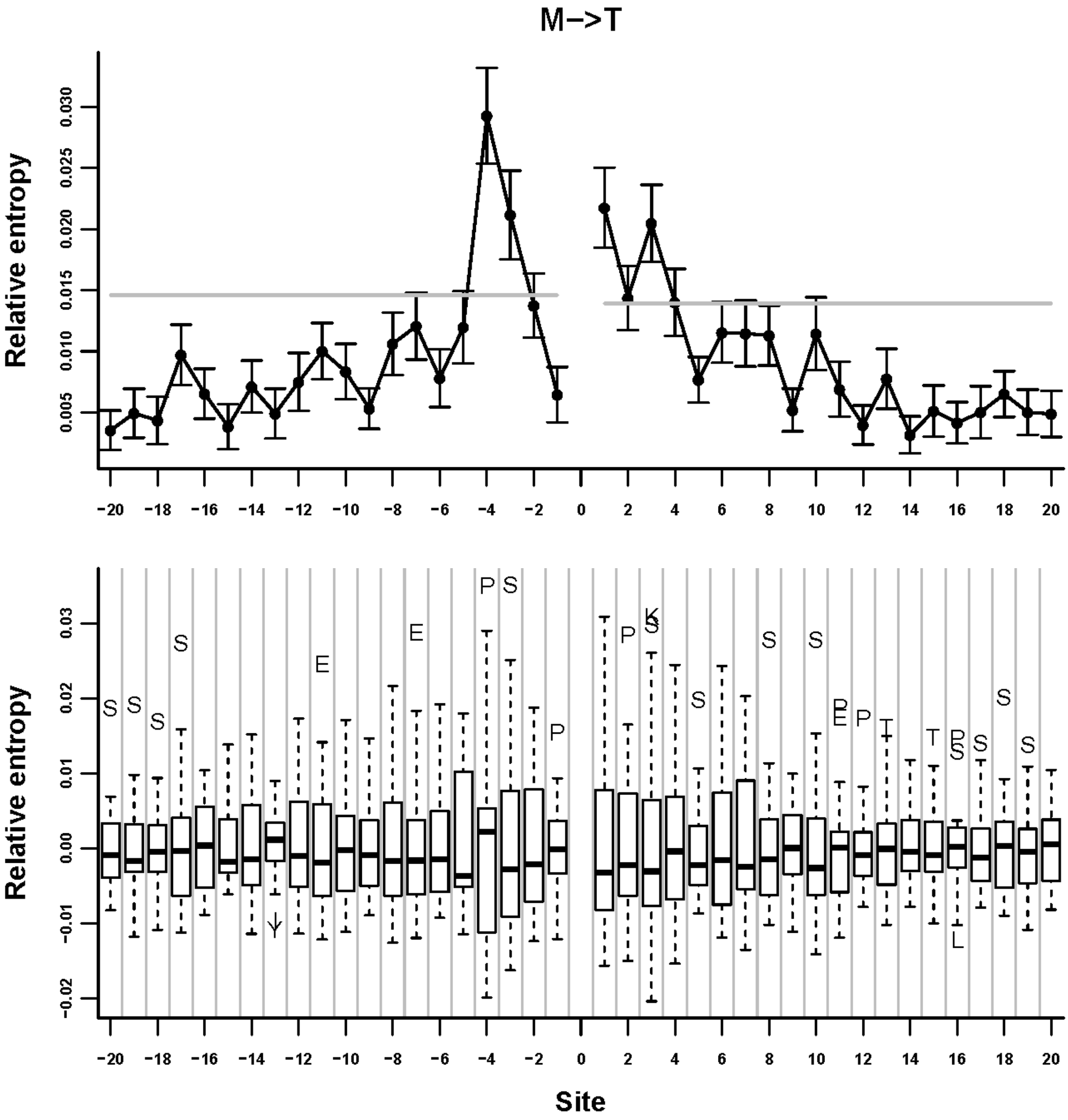
M->T
Relative entropy
Relative entropy
Site

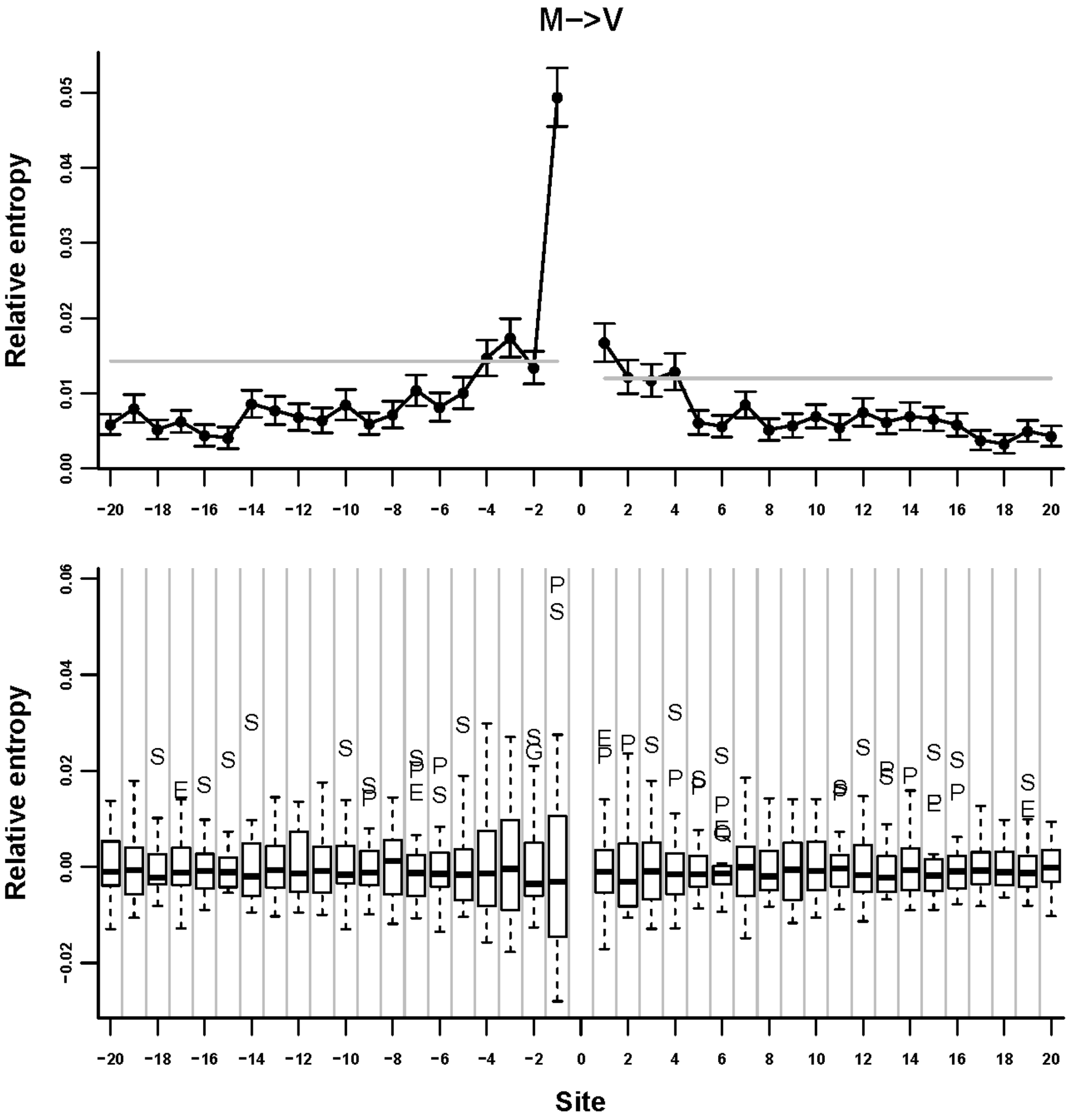
M−>V
Relative entropy
Relative entropy
Site

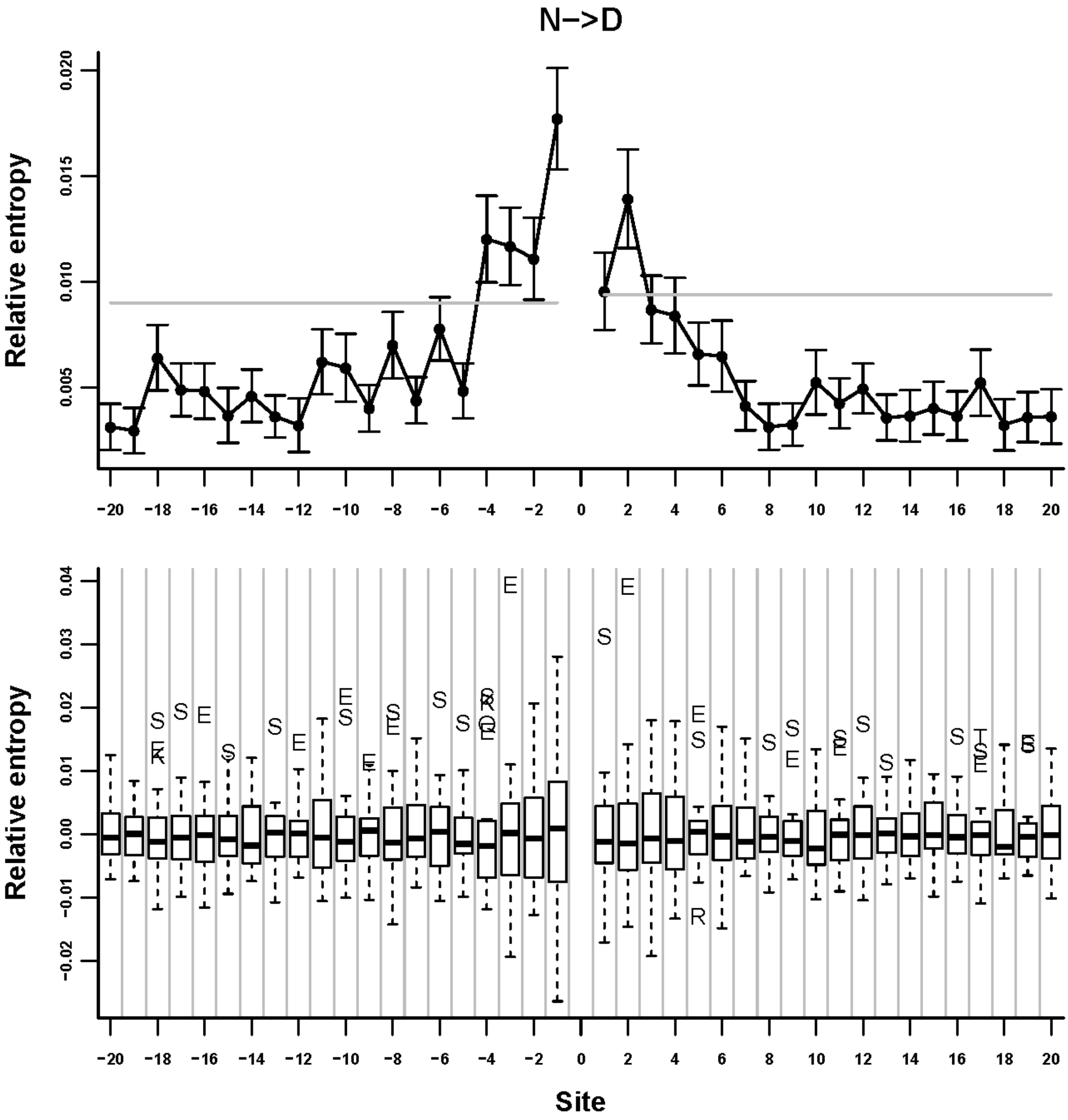

N−>D
Relative entropy
0.020
0.015
0.010
0.005
−20
−18
−16
−14
−12
−10
−8
−6
−4
−2
0
2
4
6
8
10
12
14
16
18
20
Relative entropy
0.04
0.03
0.02
0.01
0.00
−0.01
−0.02
Site

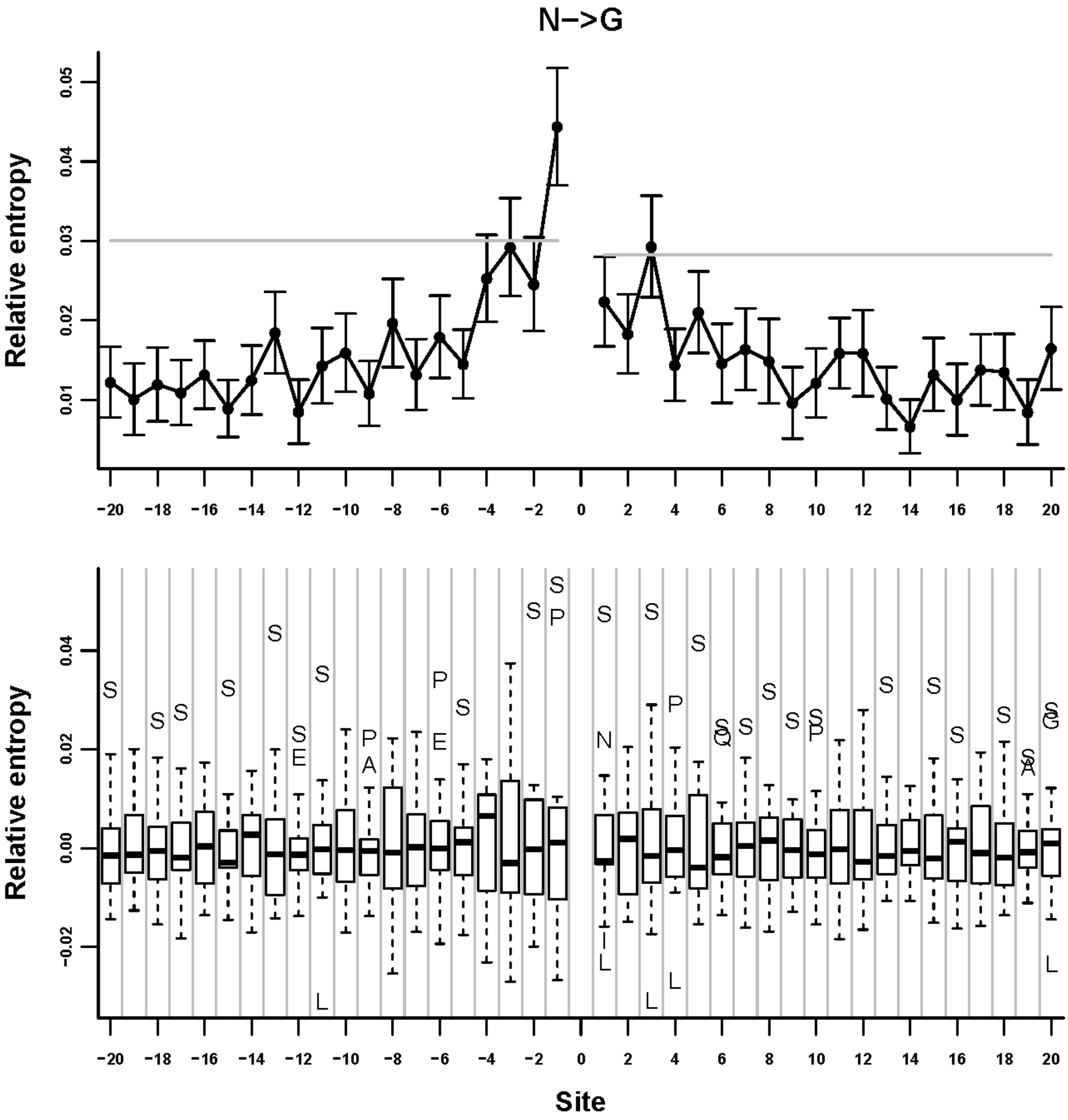
N−>G
Relative entropy
Relative entropy
Site

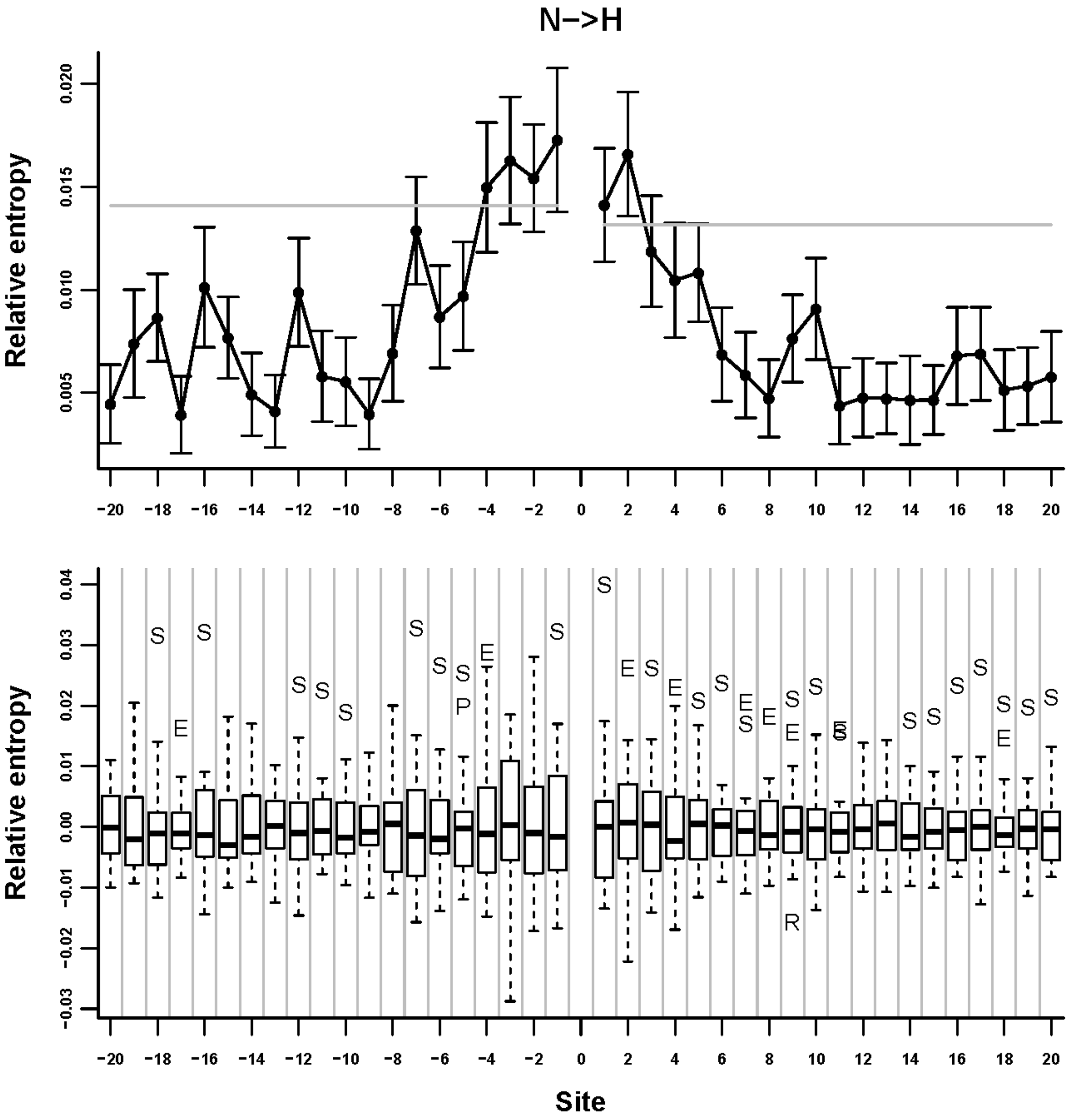
N->H
Relative entropy
Relative entropy
Site

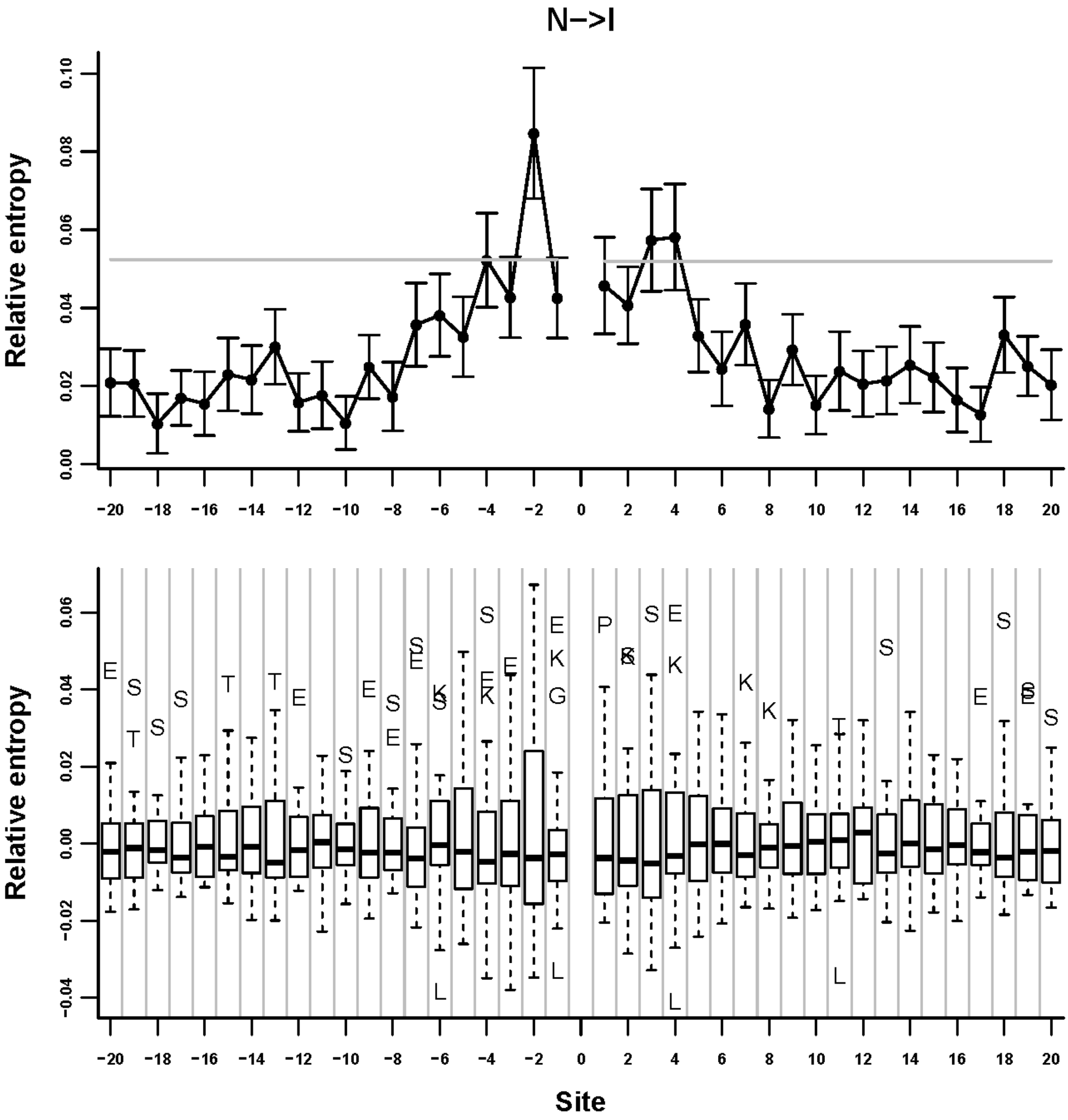

N−>I
Relative entropy
Relative entropy
Site

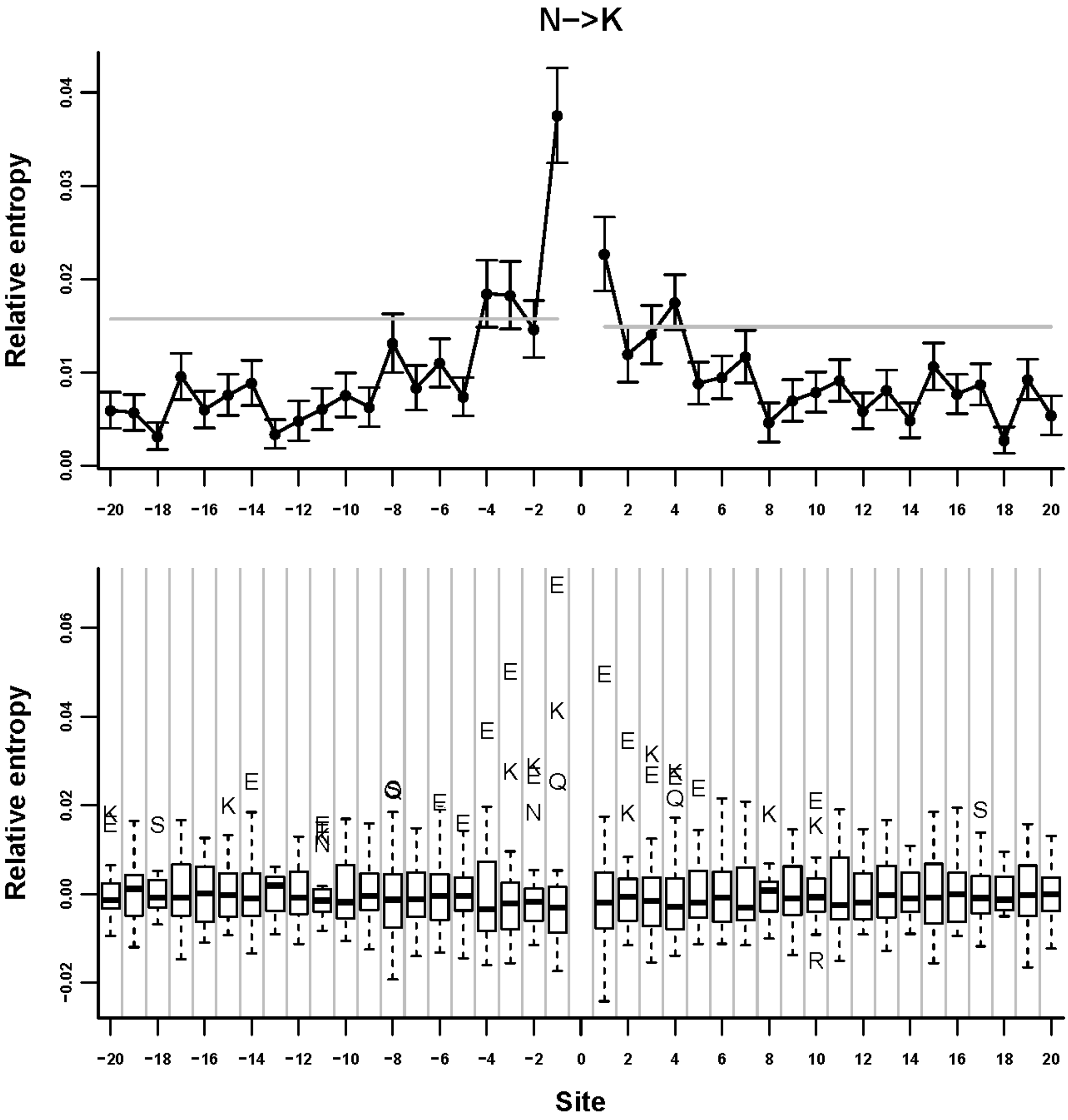
N−>K
Relative entropy
Relative entropy
Site

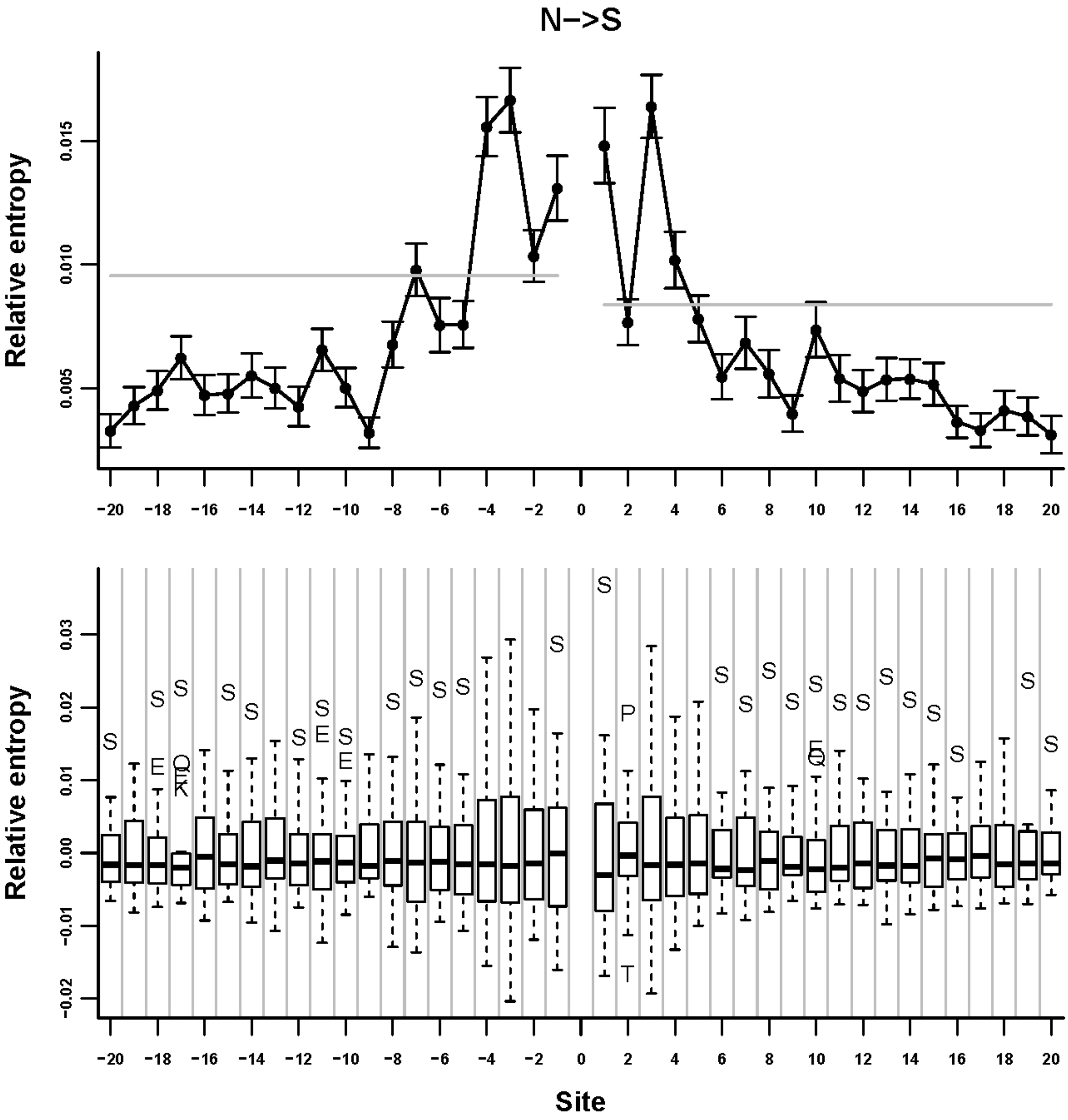
N−>S
Relative entropy
Relative entropy
Site

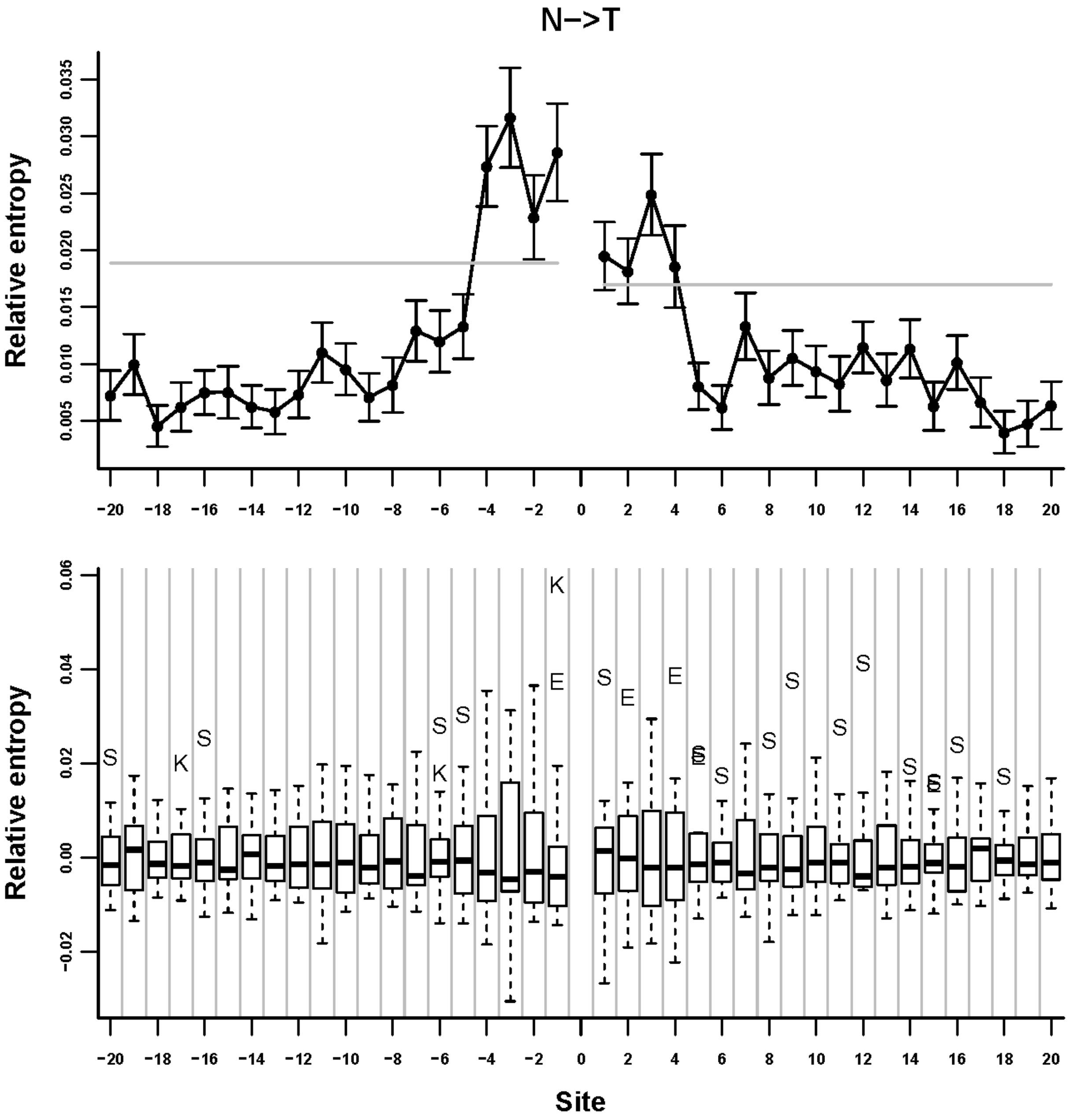
N−>T
Relative entropy
Relative entropy
Site

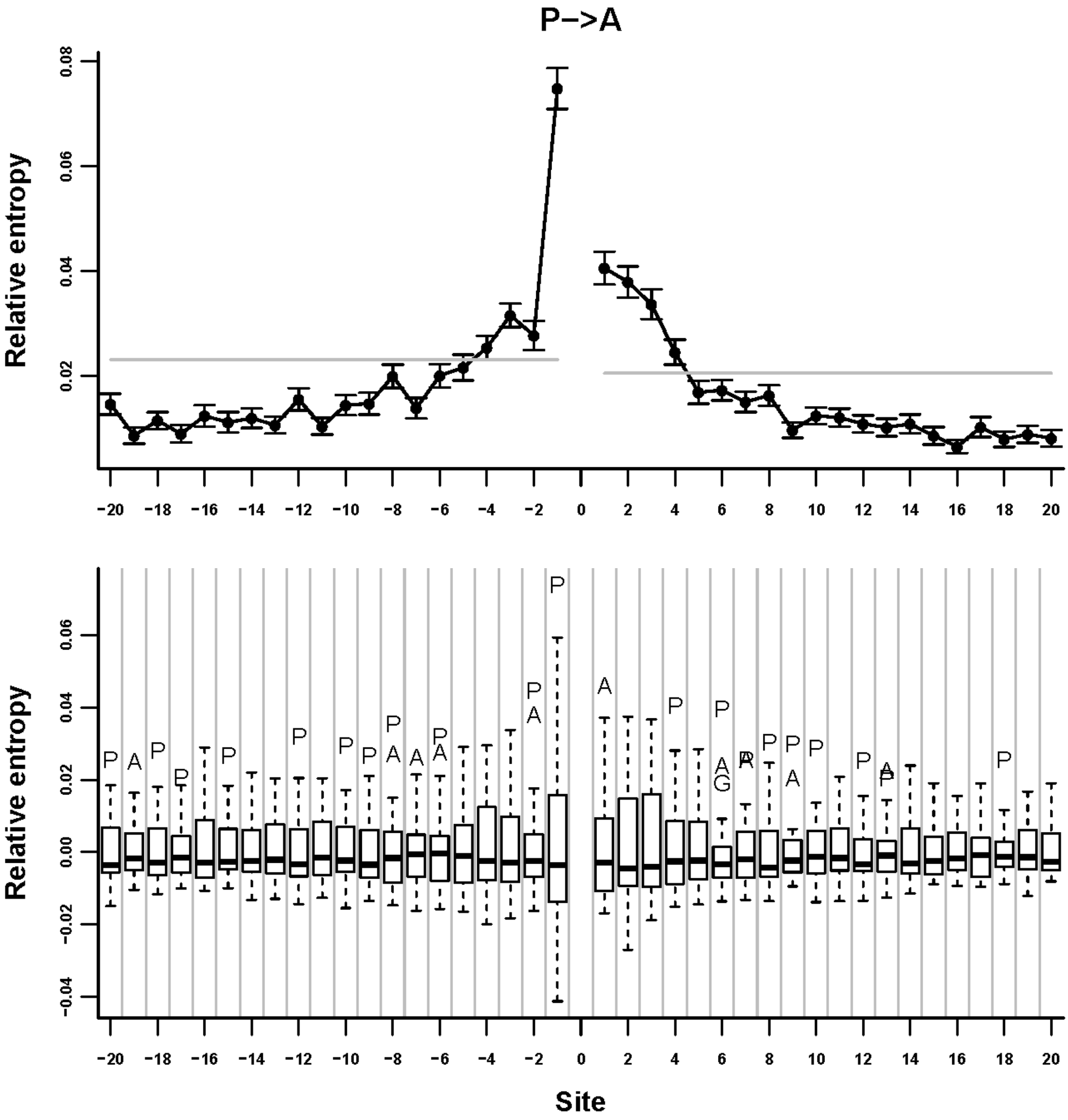
P−>A
Relative entropy
0.02
0.04
0.06
0.08
−20
−18
−16
−14
−12
−10
−8
−6
−4
−2
0
2
4
6
8
10
12
14
16
18
20
Relative entropy
−0.04
−0.02
0.00
0.02
0.04
0.06
Site

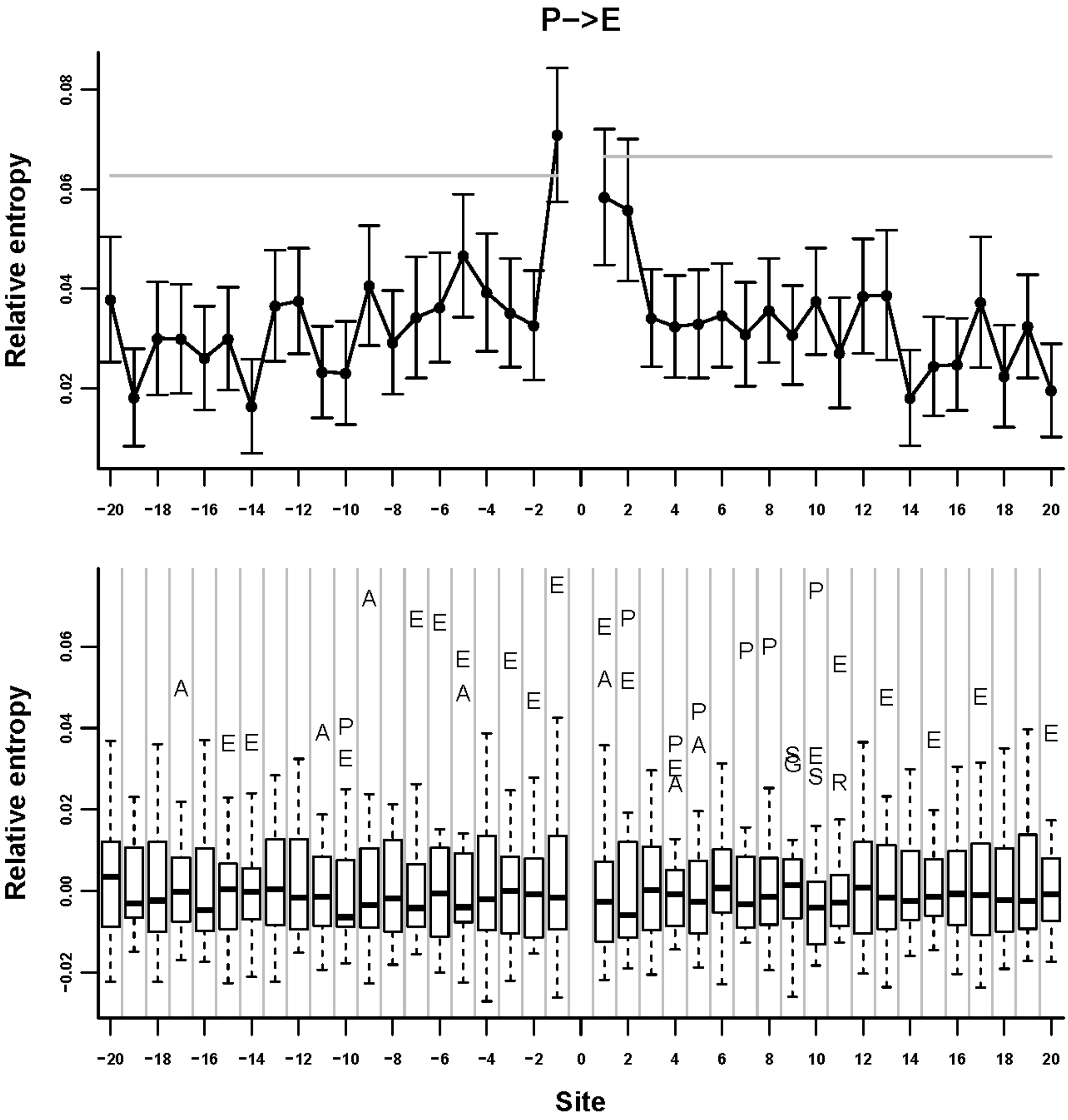
P−>E
Relative entropy
Relative entropy
Site

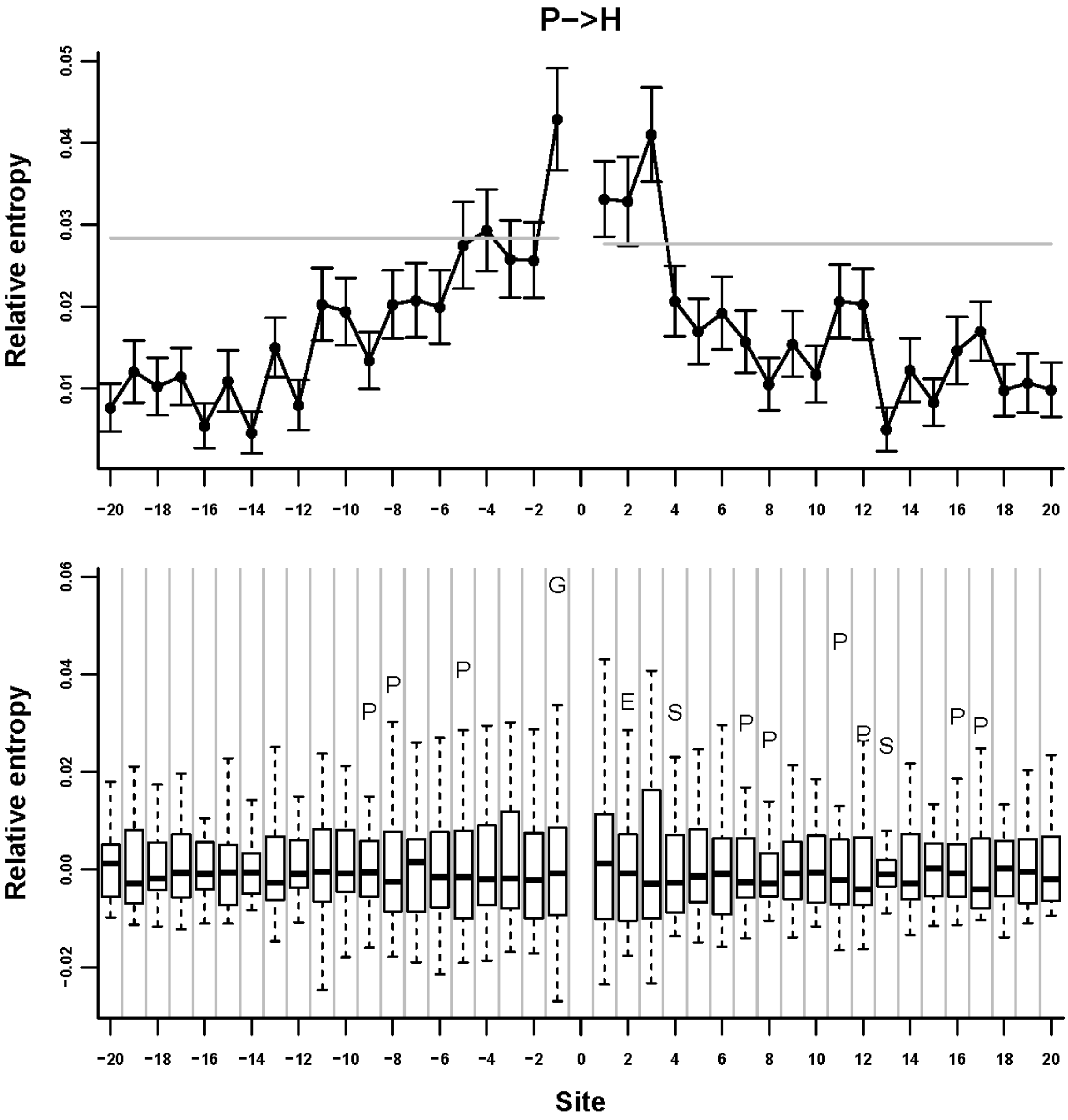
P−>H
Relative entropy
0.01
0.02
0.03
0.04
0.05
−20 −18 −16 −14 −12 −10 −8 −6 −4 −2 0 2 4 6 8 10 12 14 16 18 20
Relative entropy
−0.02
0.00
0.02
0.04
0.06
G
P
P
P
P
E
S
P
P
P
S
P
P
P
−20 −18 −16 −14 −12 −10 −8 −6 −4 −2 0 2 4 6 8 10 12 14 16 18 20
Site

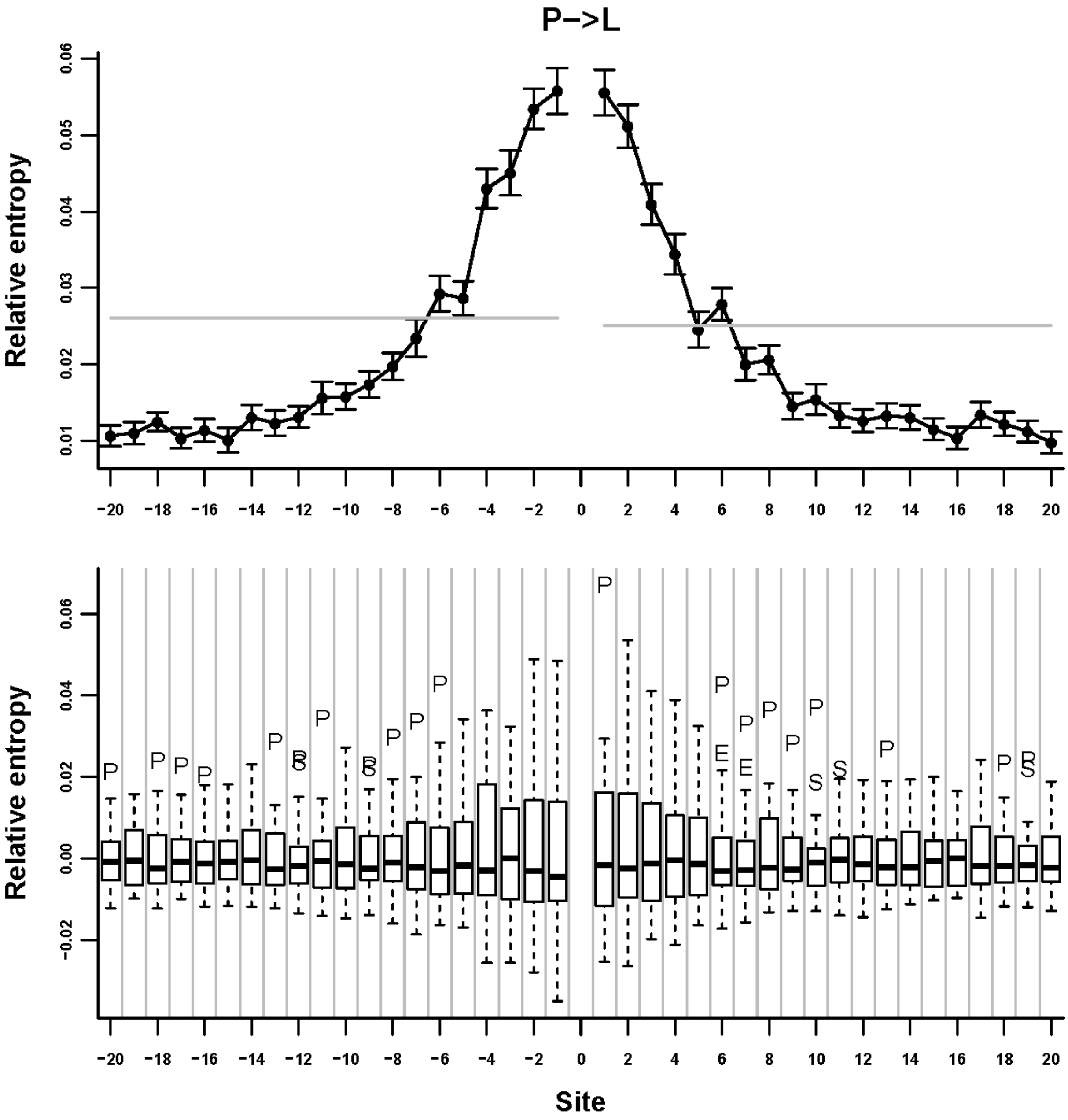
P−>L
Relative entropy
Relative entropy
Site

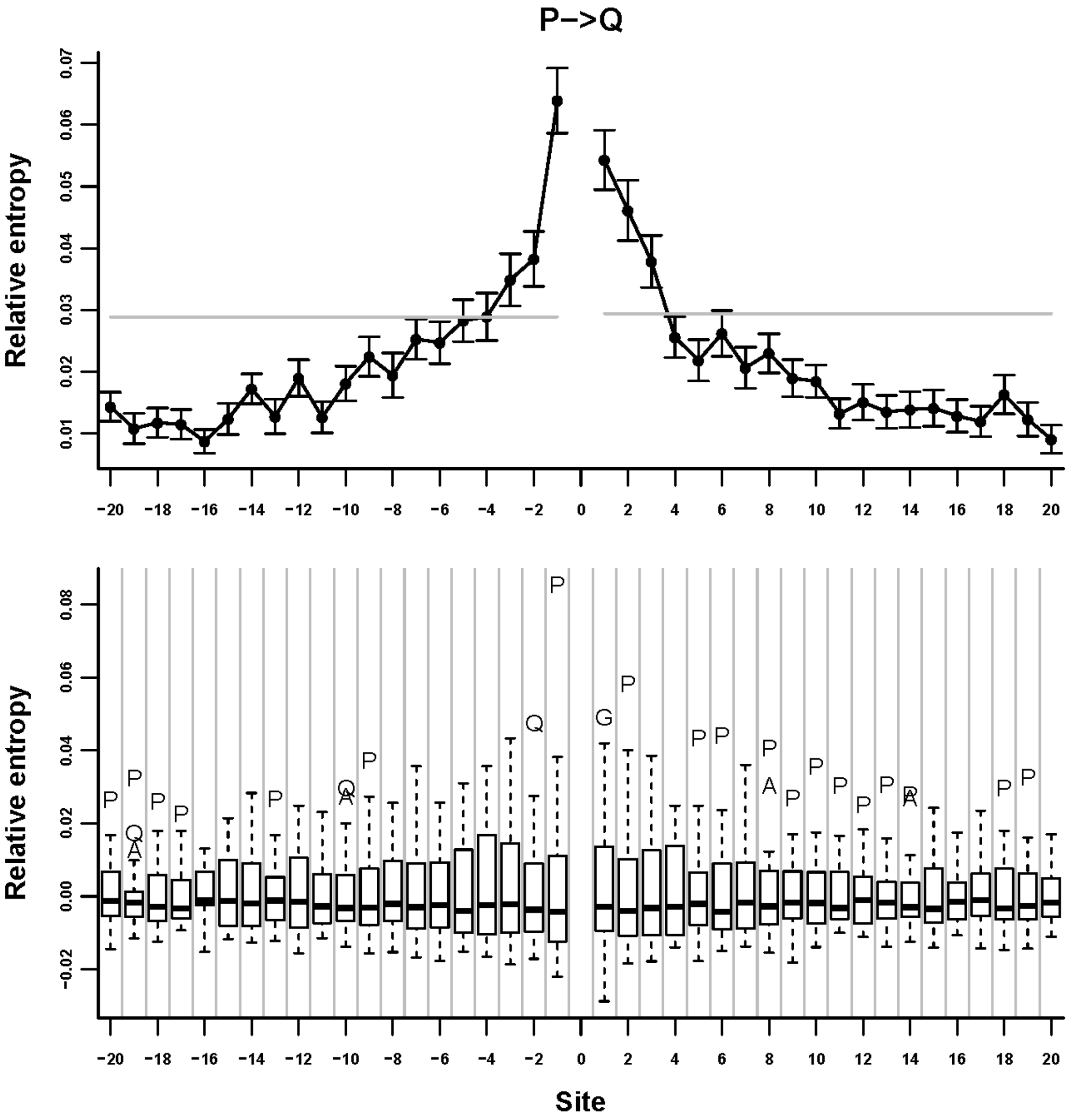
P−>Q
Relative entropy
Relative entropy
Site

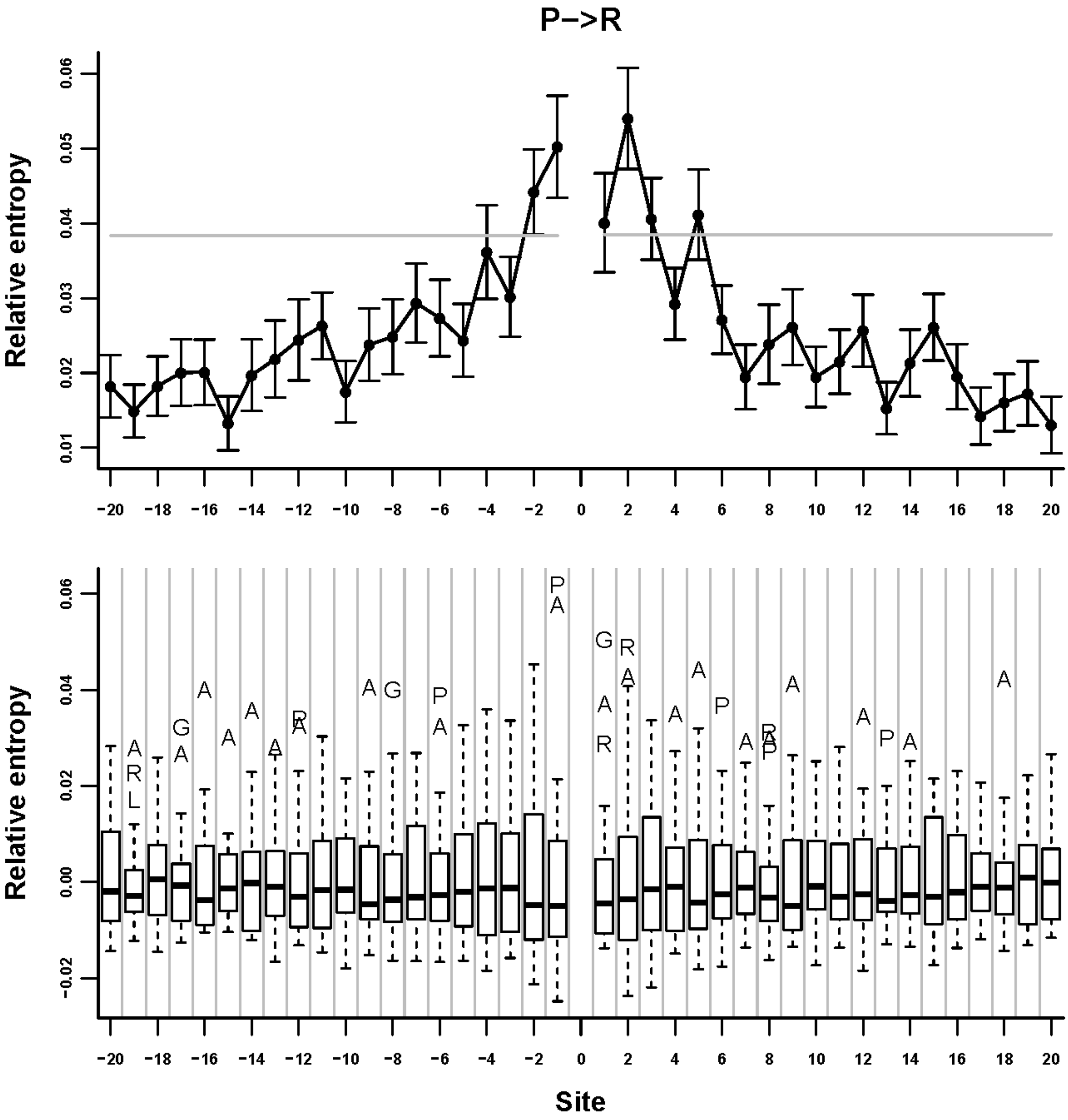
P−>R
Relative entropy
Relative entropy
Site

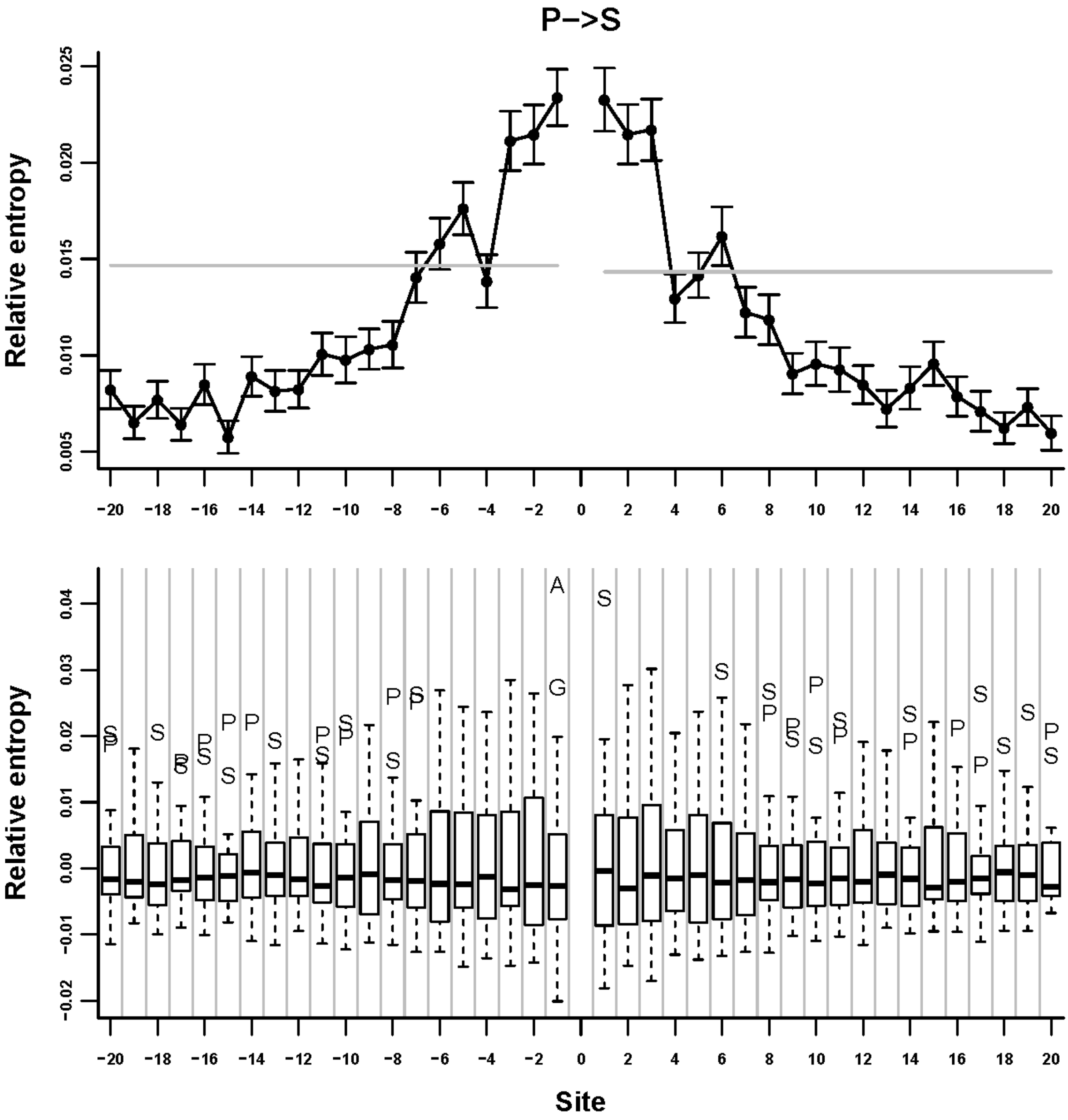

P−>S
Relative entropy
0.005
0.010
0.015
0.020
0.025
−20
−18
−16
−14
−12
−10
−8
−6
−4
−2
0
2
4
6
8
10
12
14
16
18
20
Relative entropy
−0.02
−0.01
0.00
0.01
0.02
0.03
0.04
A
G
S
Site

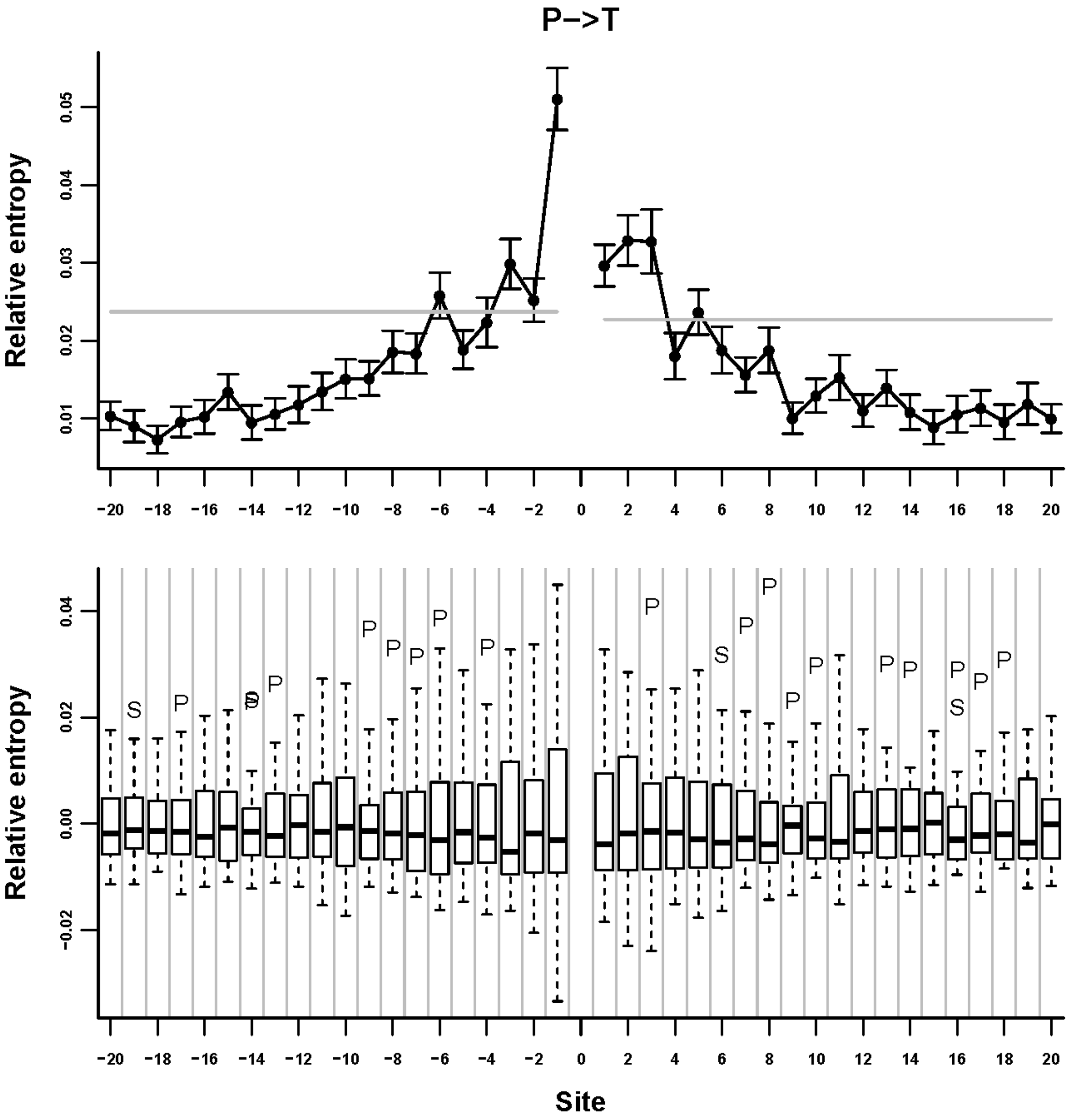
P−>T
Relative entropy
0.01
0.02
0.03
0.04
0.05
Relative entropy
−0.02
0.00
0.02
0.04
−20
−18
−16
−14
−12
−10
−8
−6
−4
−2
0
2
4
6
8
10
12
14
16
18
20
Site

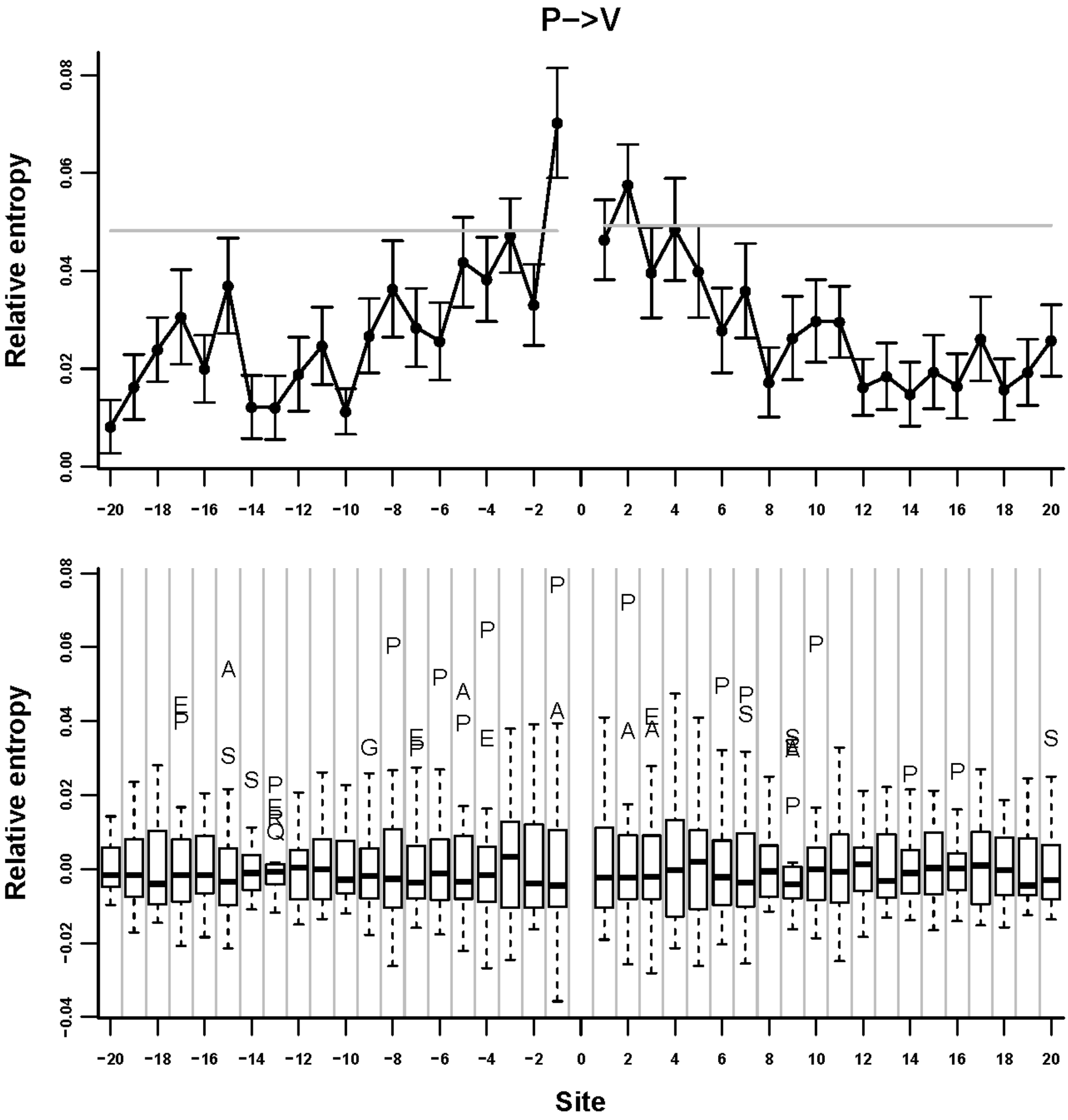

P−>V
Relative entropy
Relative entropy
Site

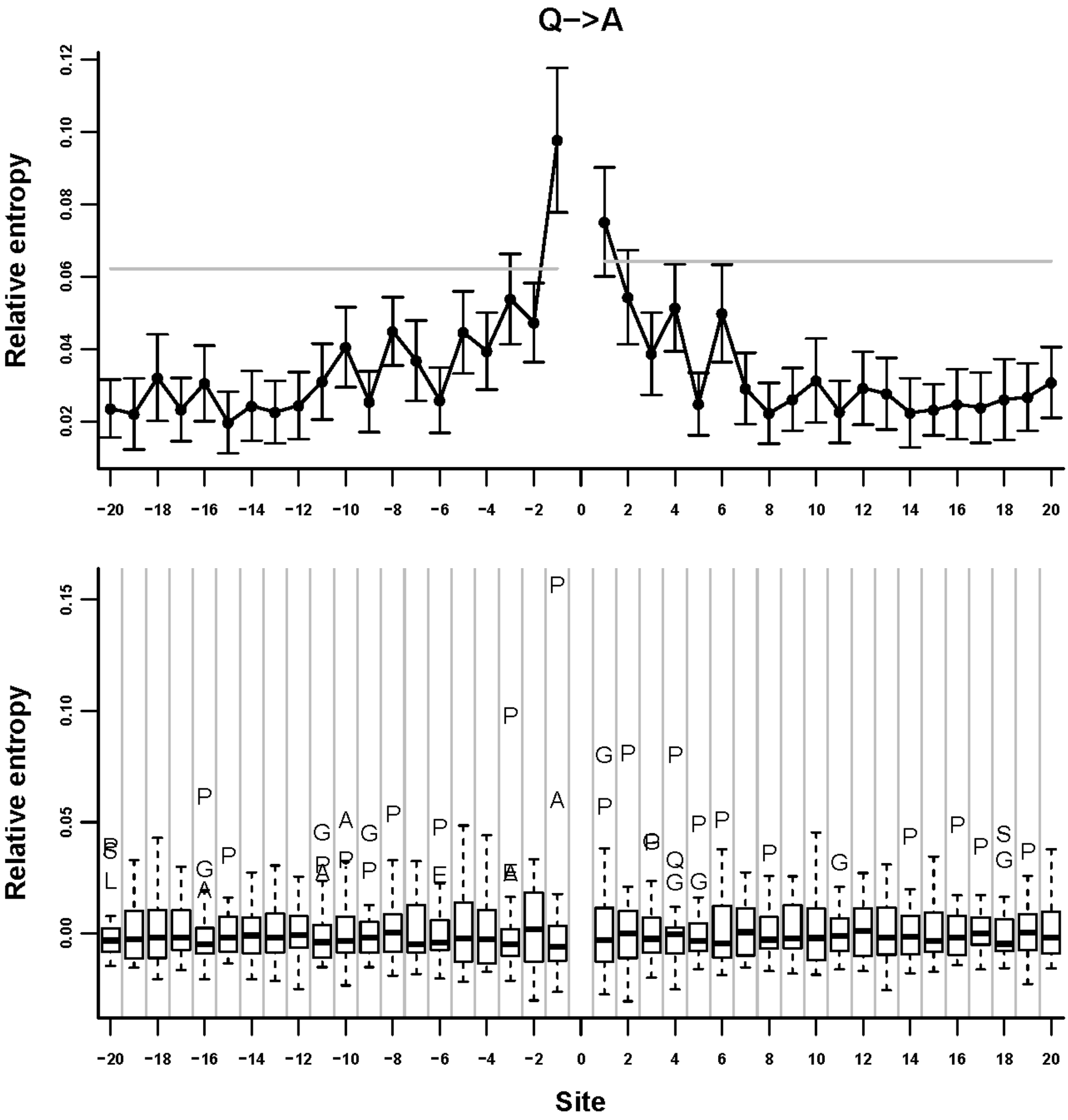
Q->A
Relative entropy
Relative entropy
Site

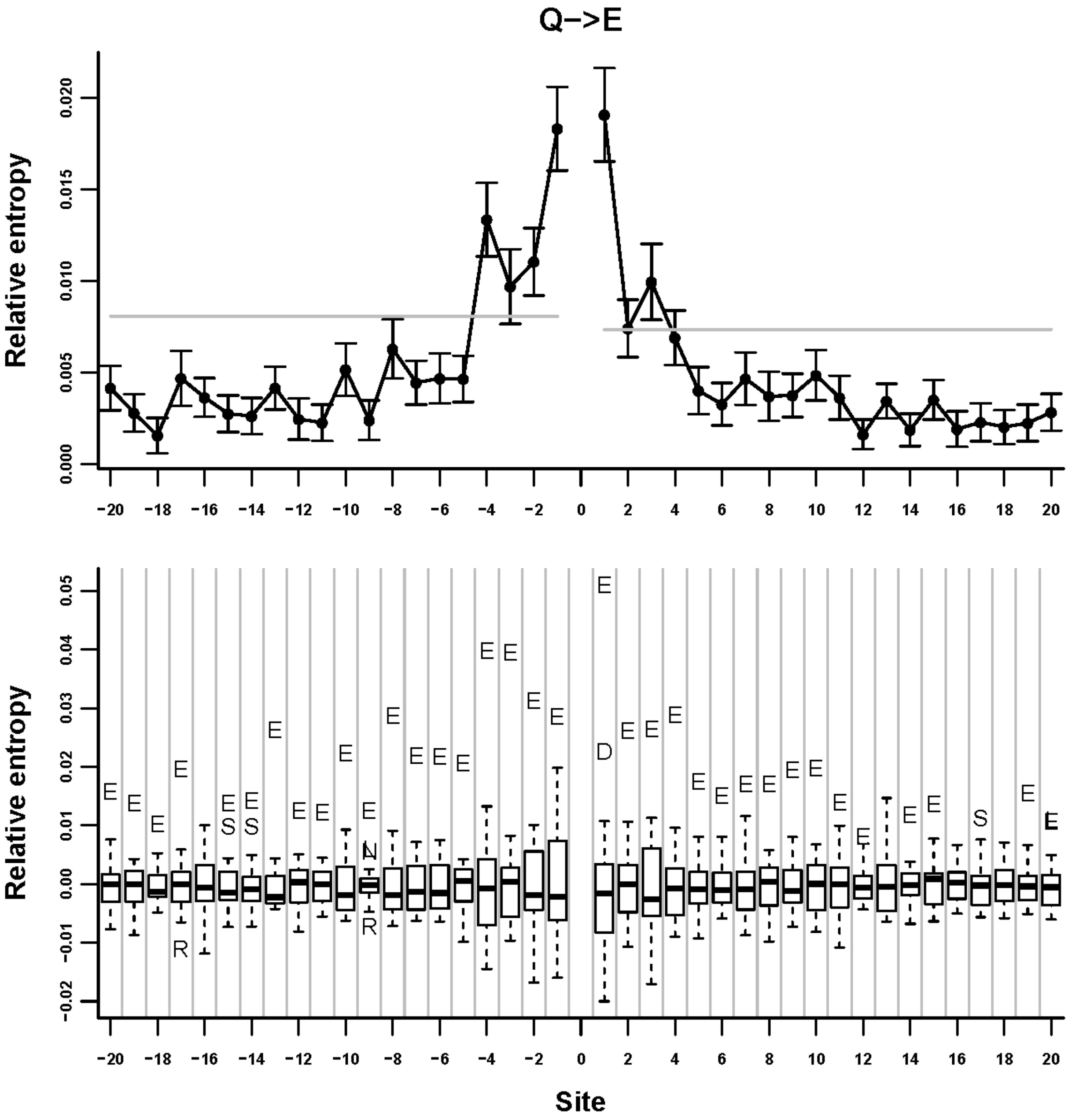
Q−>E
Relative entropy
Relative entropy
Site

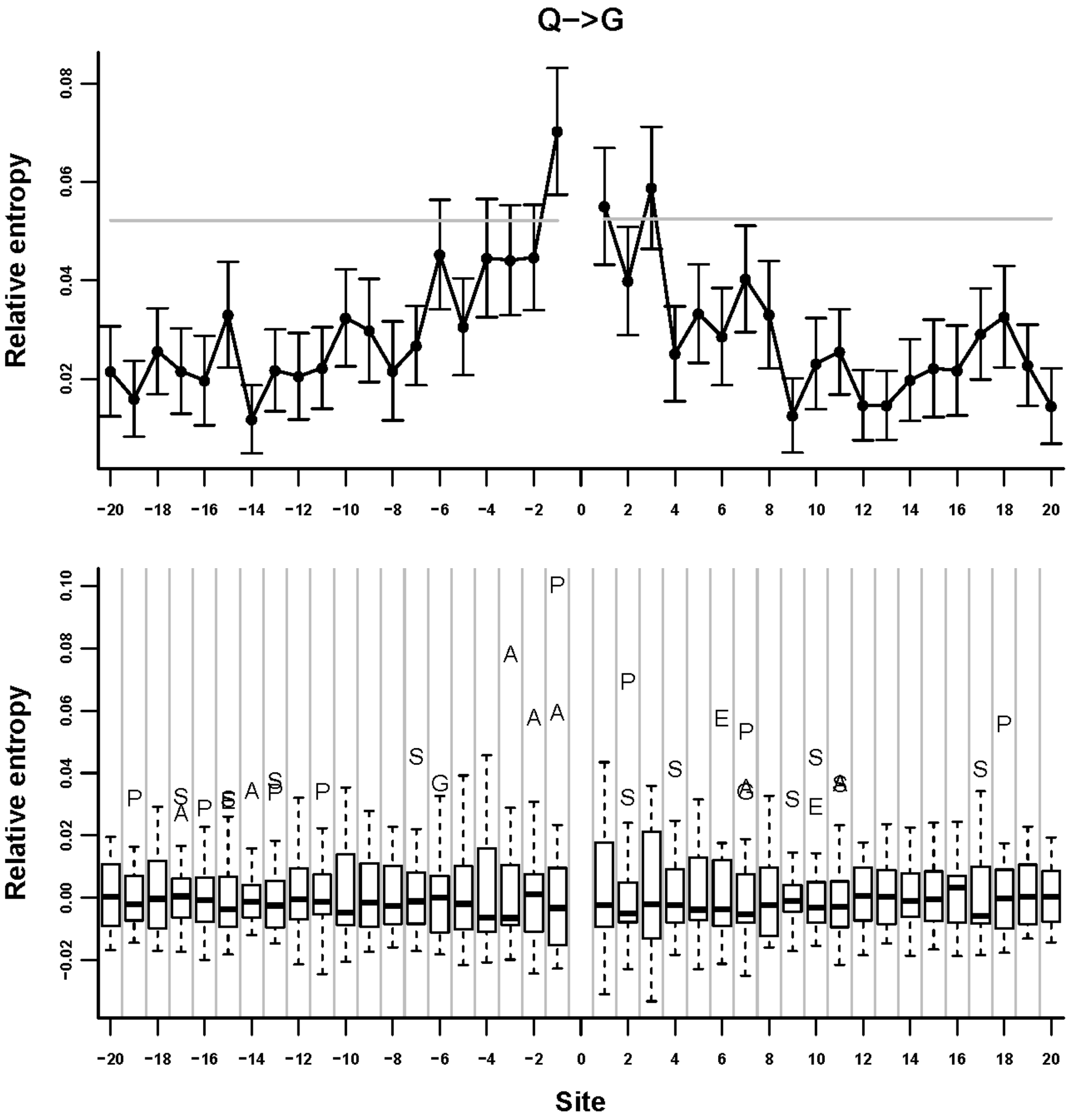
Q−>G
Relative entropy
Relative entropy
Site

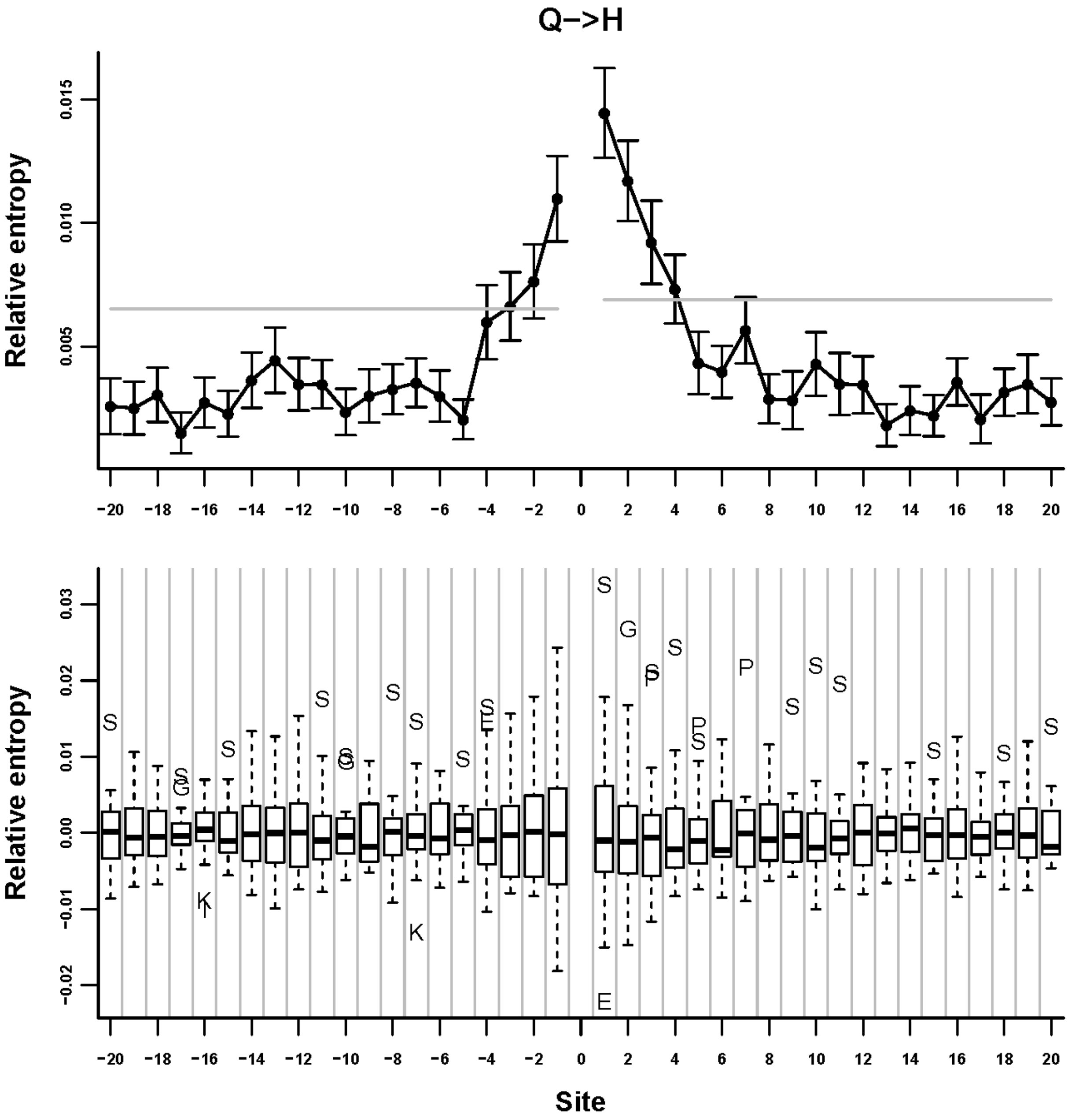

Q−>H
Relative entropy
0.015
0.010
0.005
−20 −18 −16 −14 −12 −10 −8 −6 −4 −2 0 2 4 6 8 10 12 14 16 18 20
Relative entropy
0.03
0.02
0.01
0.00
−0.01
−0.02
S G S P S E S K S S G S S S E S P S P S S S S S S S
−20 −18 −16 −14 −12 −10 −8 −6 −4 −2 0 2 4 6 8 10 12 14 16 18 20
Site

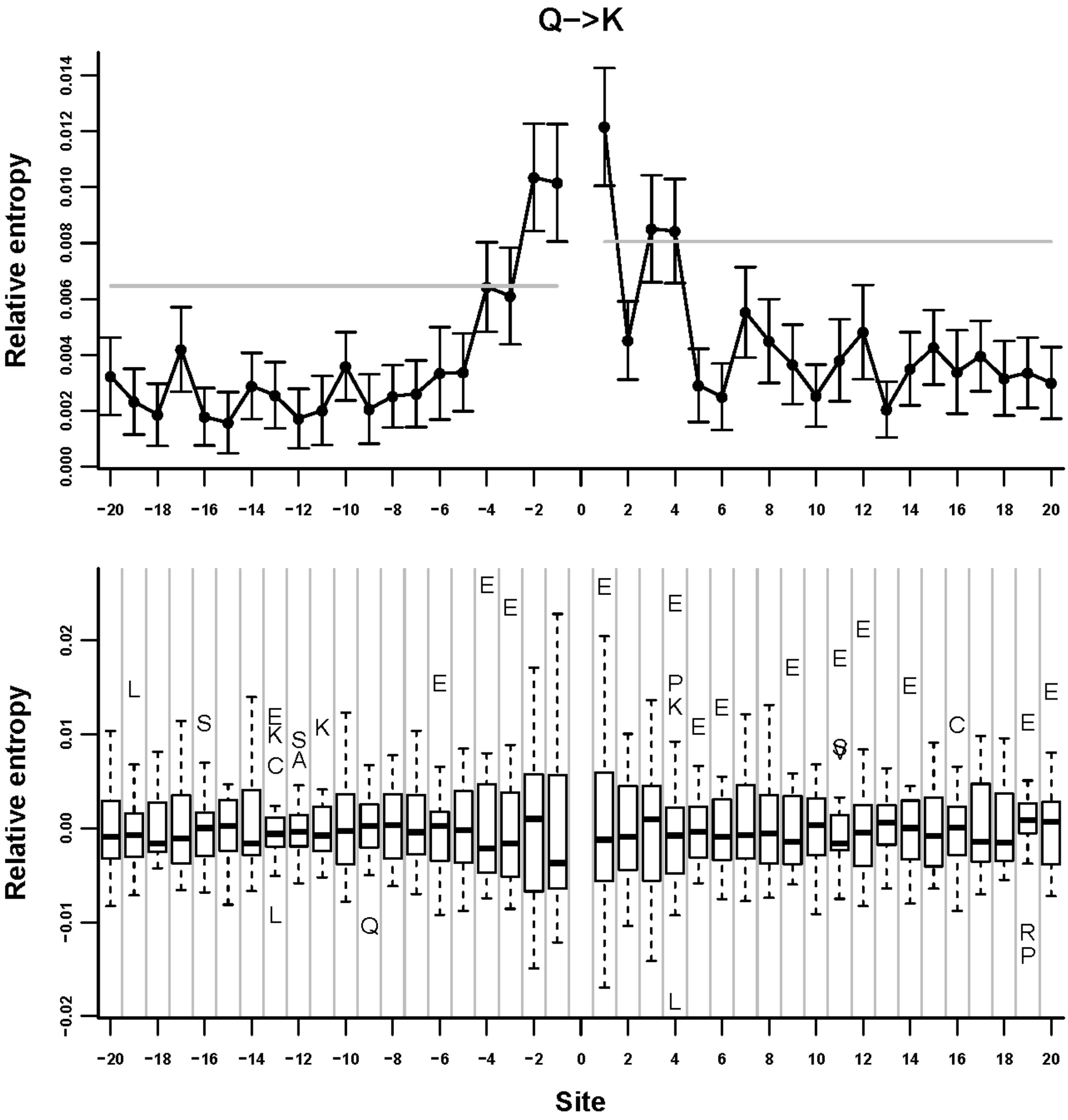
Q->K
Relative entropy
Relative entropy
Site

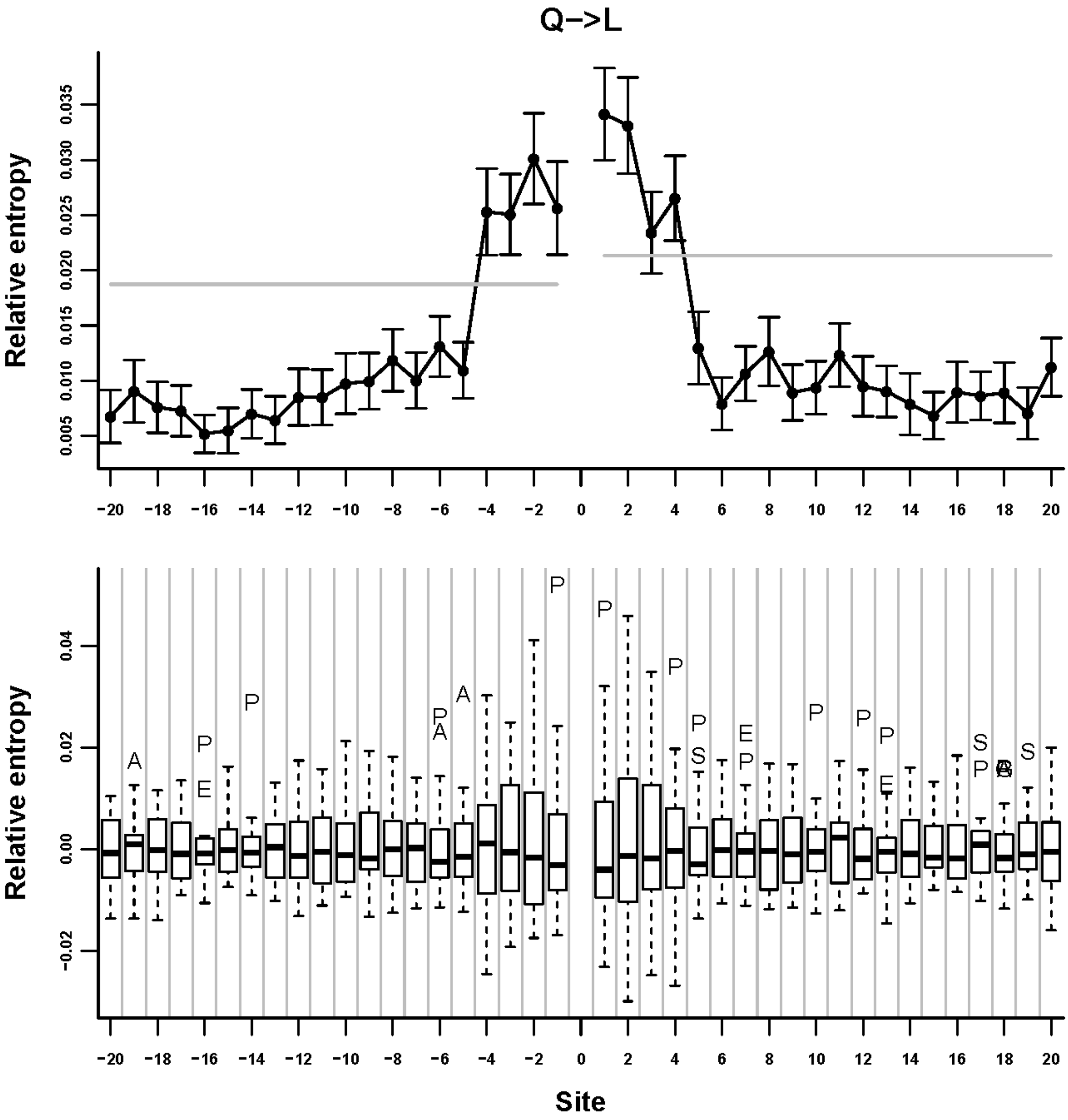
Q−>L
Relative entropy
Site

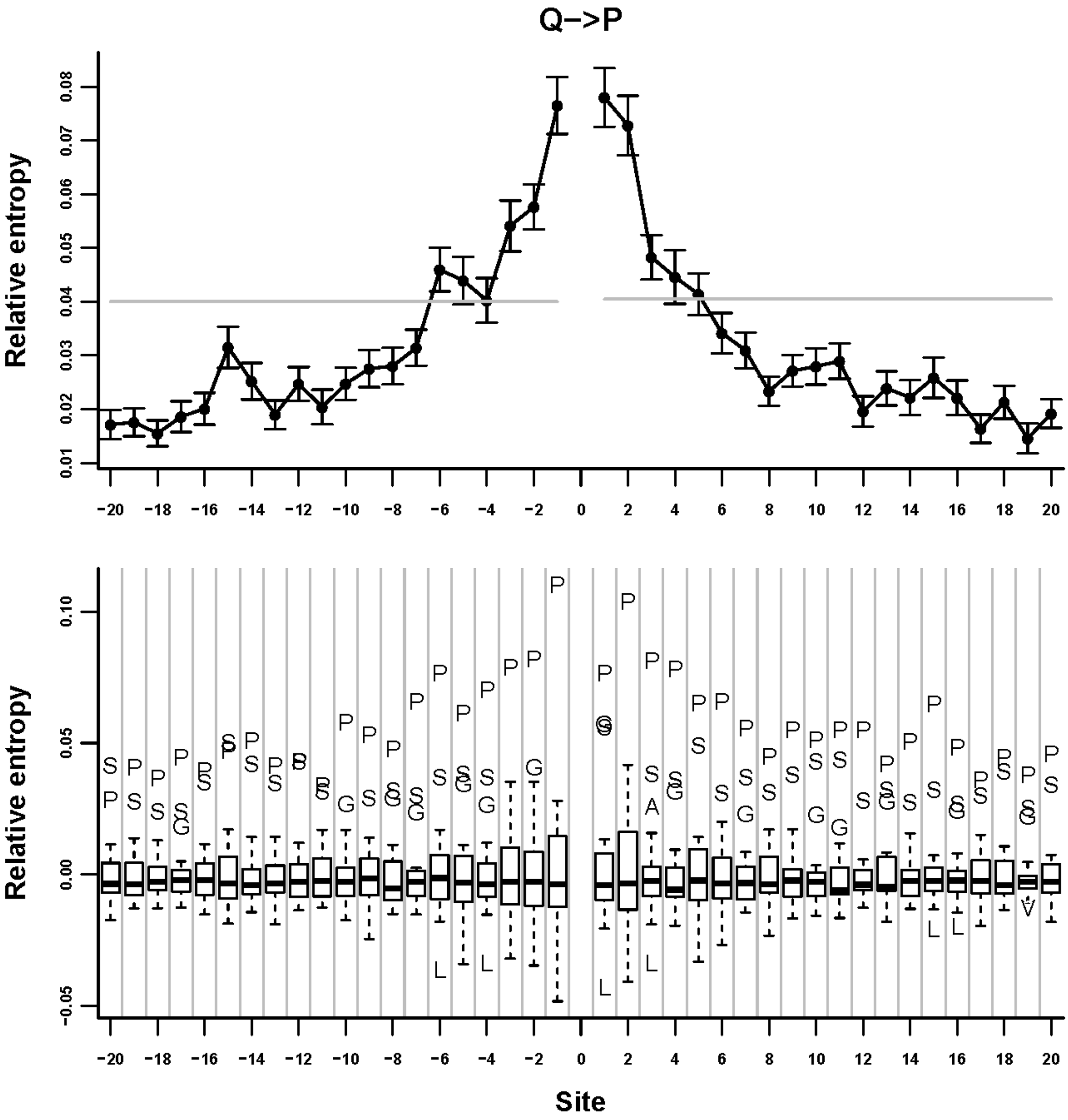
Q−>P
Relative entropy
Site

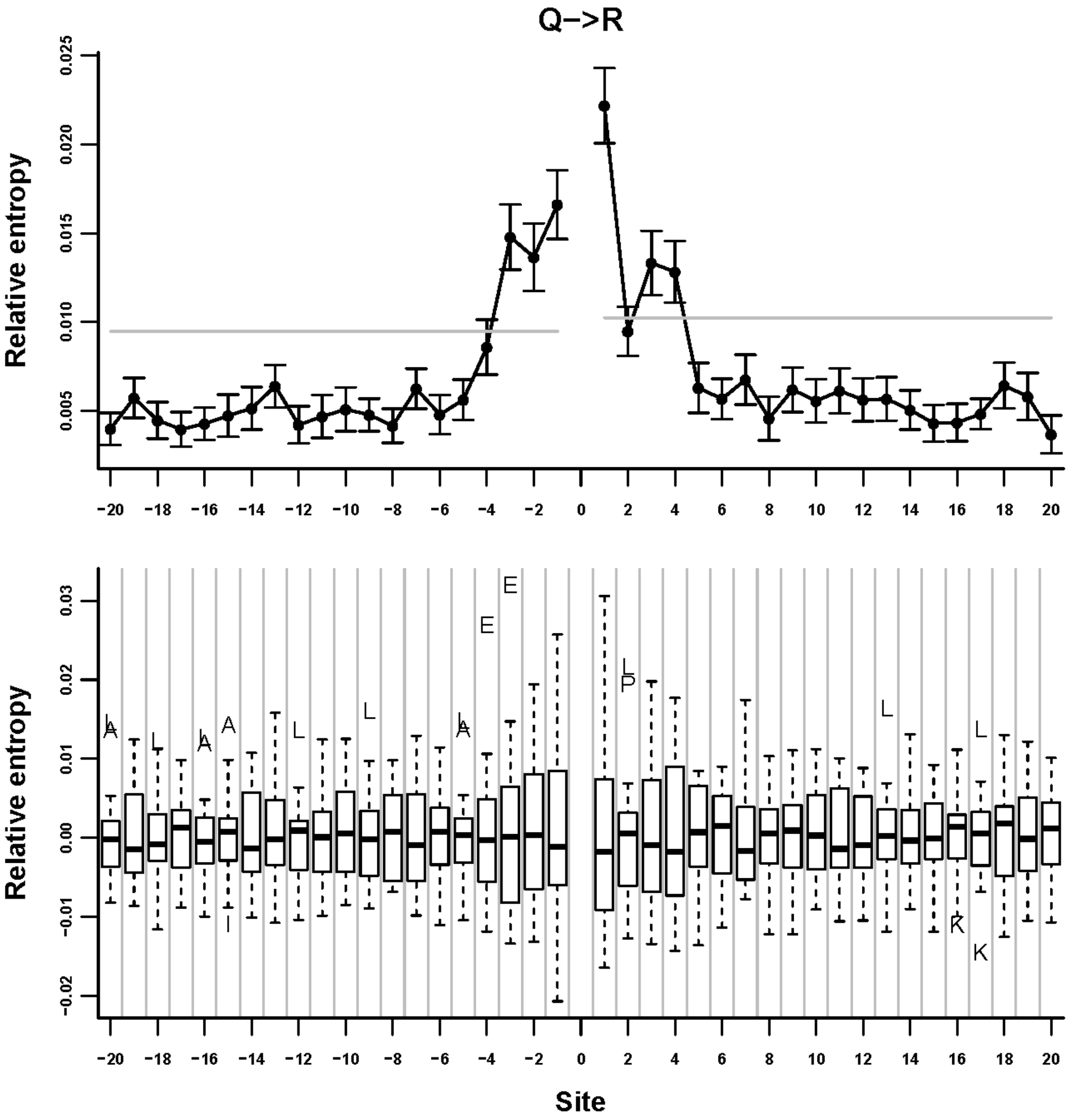

Q−>R
Relative entropy
0.005
0.010
0.015
0.020
0.025
−20
−18
−16
−14
−12
−10
−8
−6
−4
−2
0
2
4
6
8
10
12
14
16
18
20
Relative entropy
−0.02
−0.01
0.00
0.01
0.02
0.03
Site

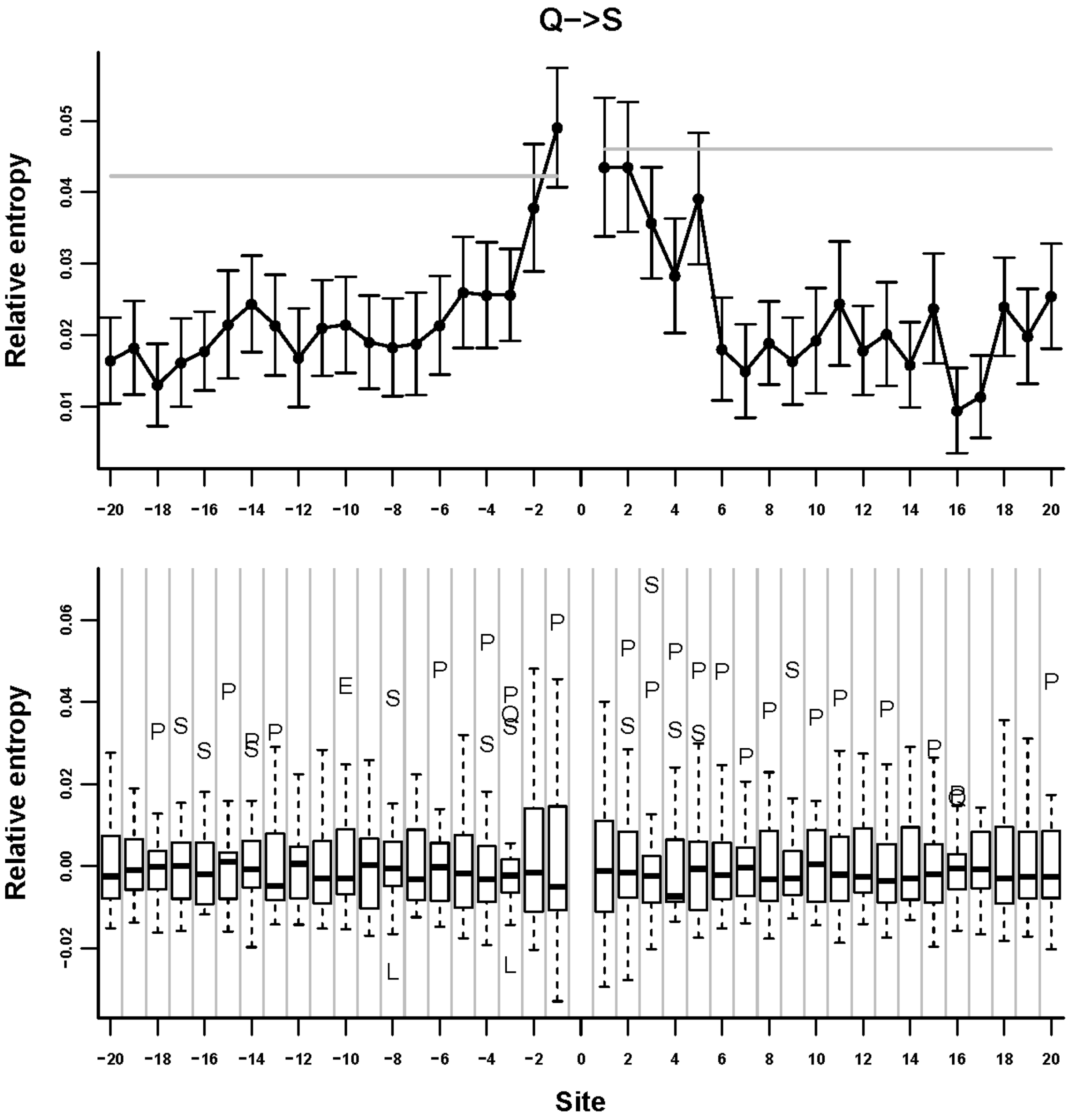
Q−>S
Relative entropy
Site

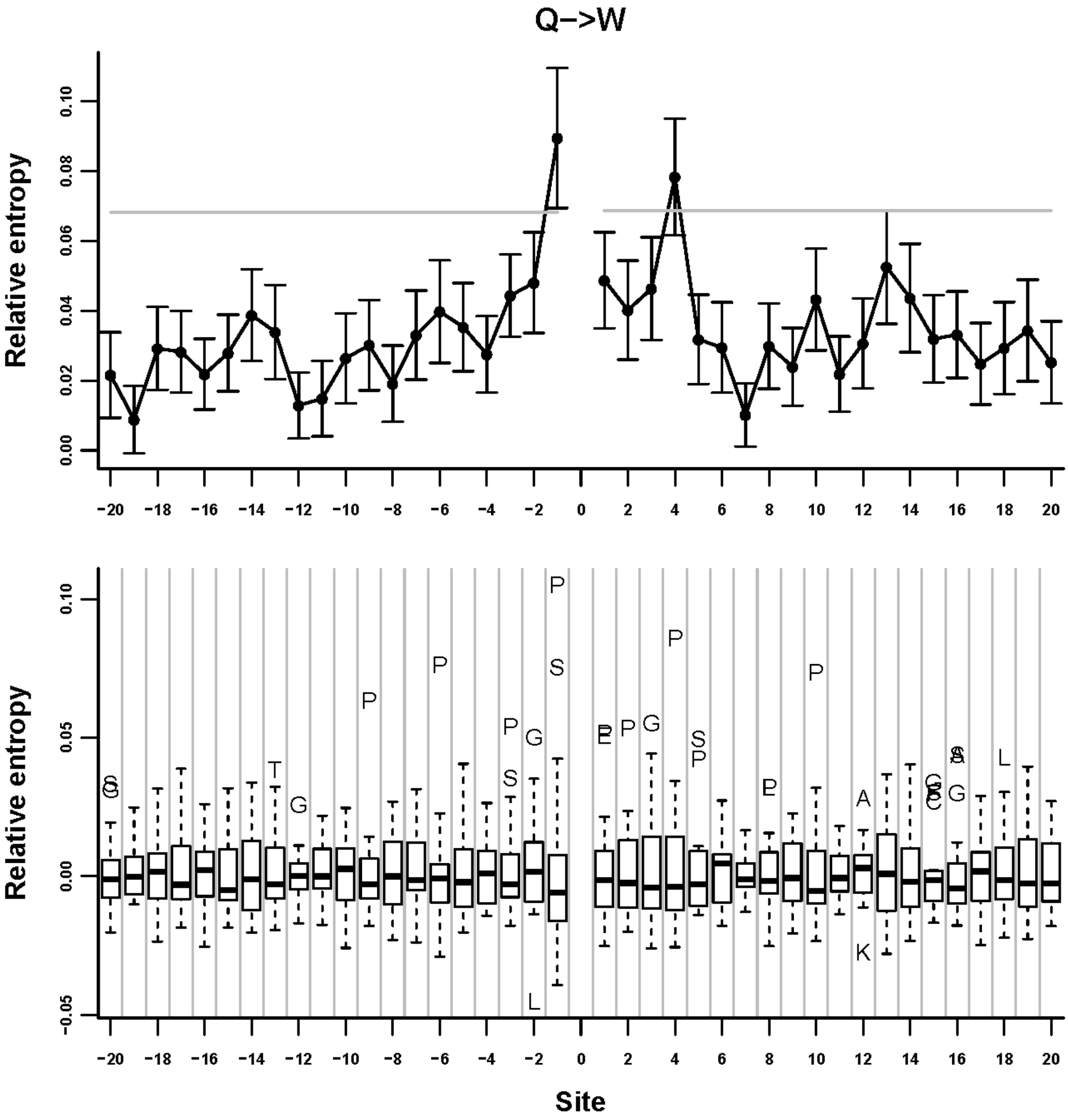
Q−>W
Relative entropy
0.00
0.02
0.04
0.06
0.08
0.10
−20
−18
−16
−14
−12
−10
−8
−6
−4
−2
0
2
4
6
8
10
12
14
16
18
20
−0.05
0.00
0.05
0.10
Site

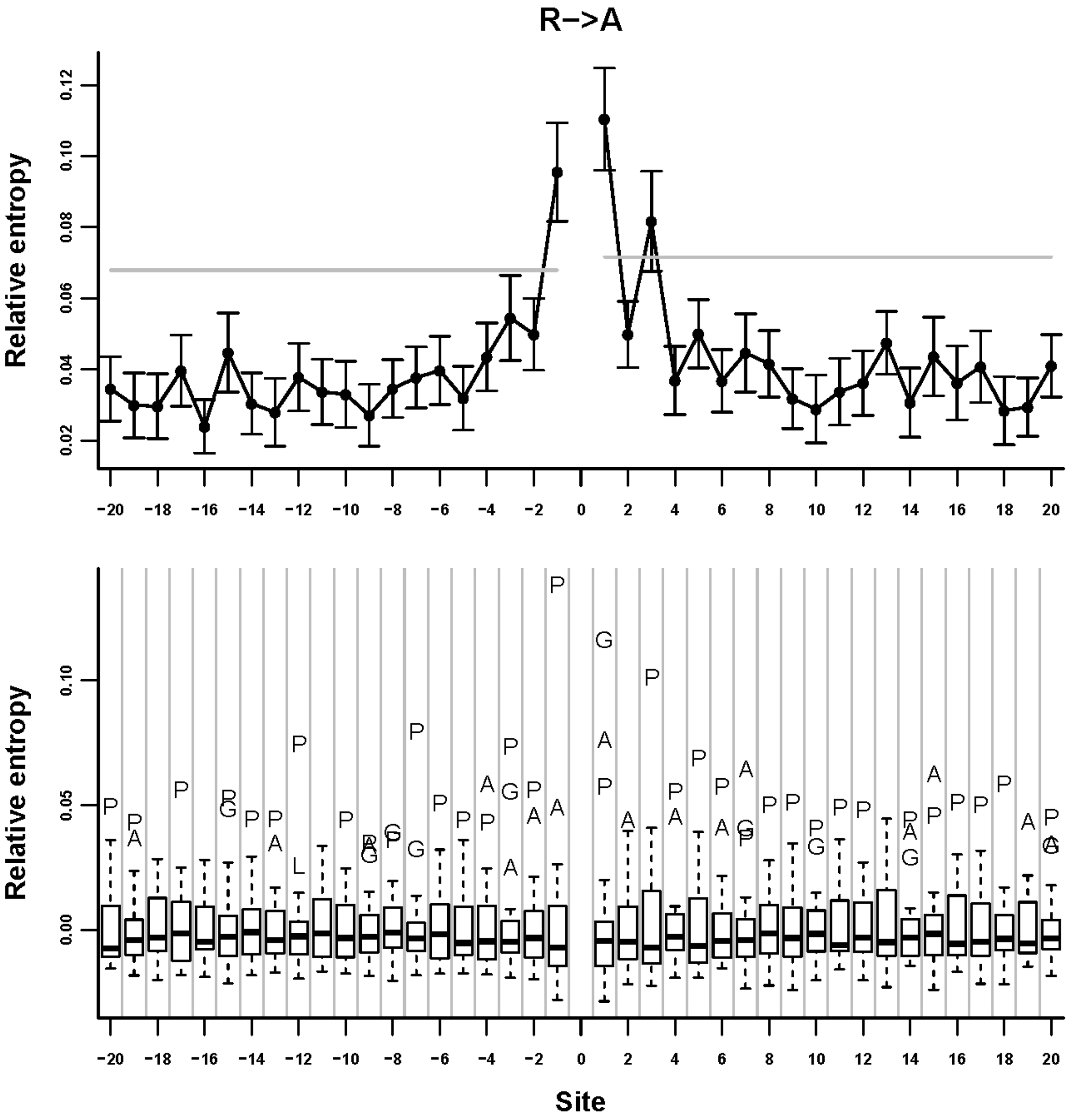
R−>A
Relative entropy
Relative entropy
Site

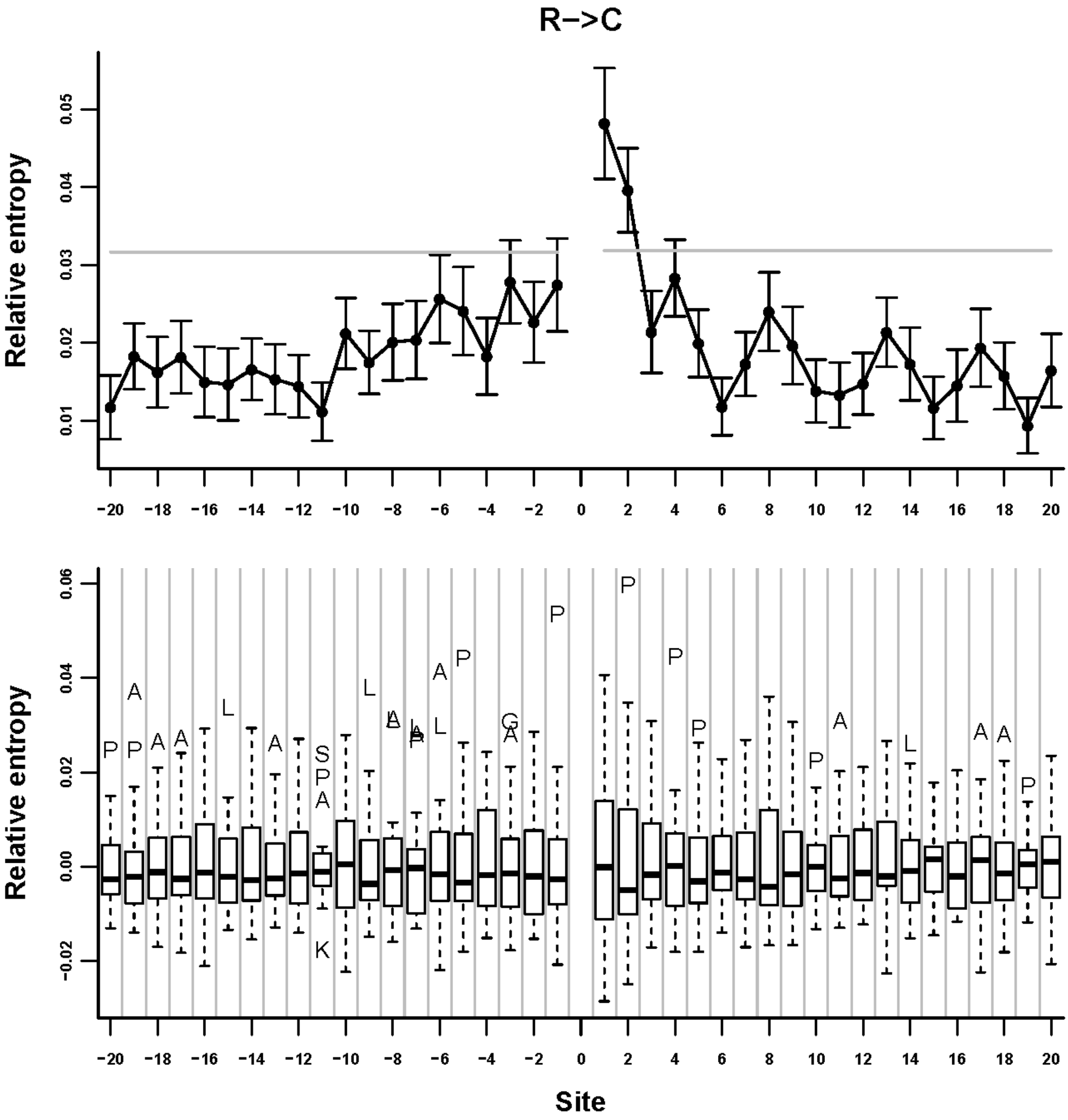
R−>C
Relative entropy
Relative entropy
Site

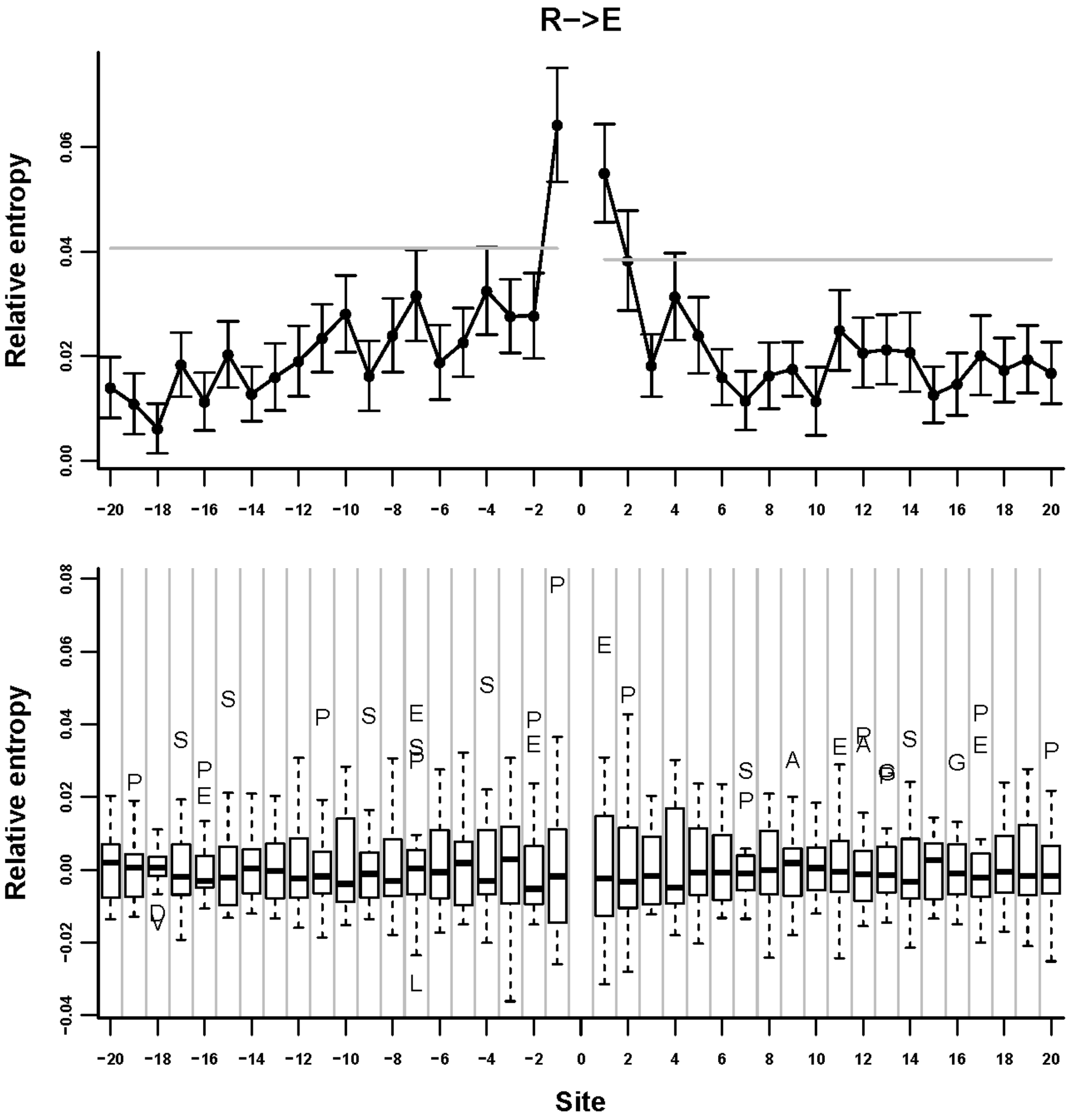
R->E
Relative entropy
Site

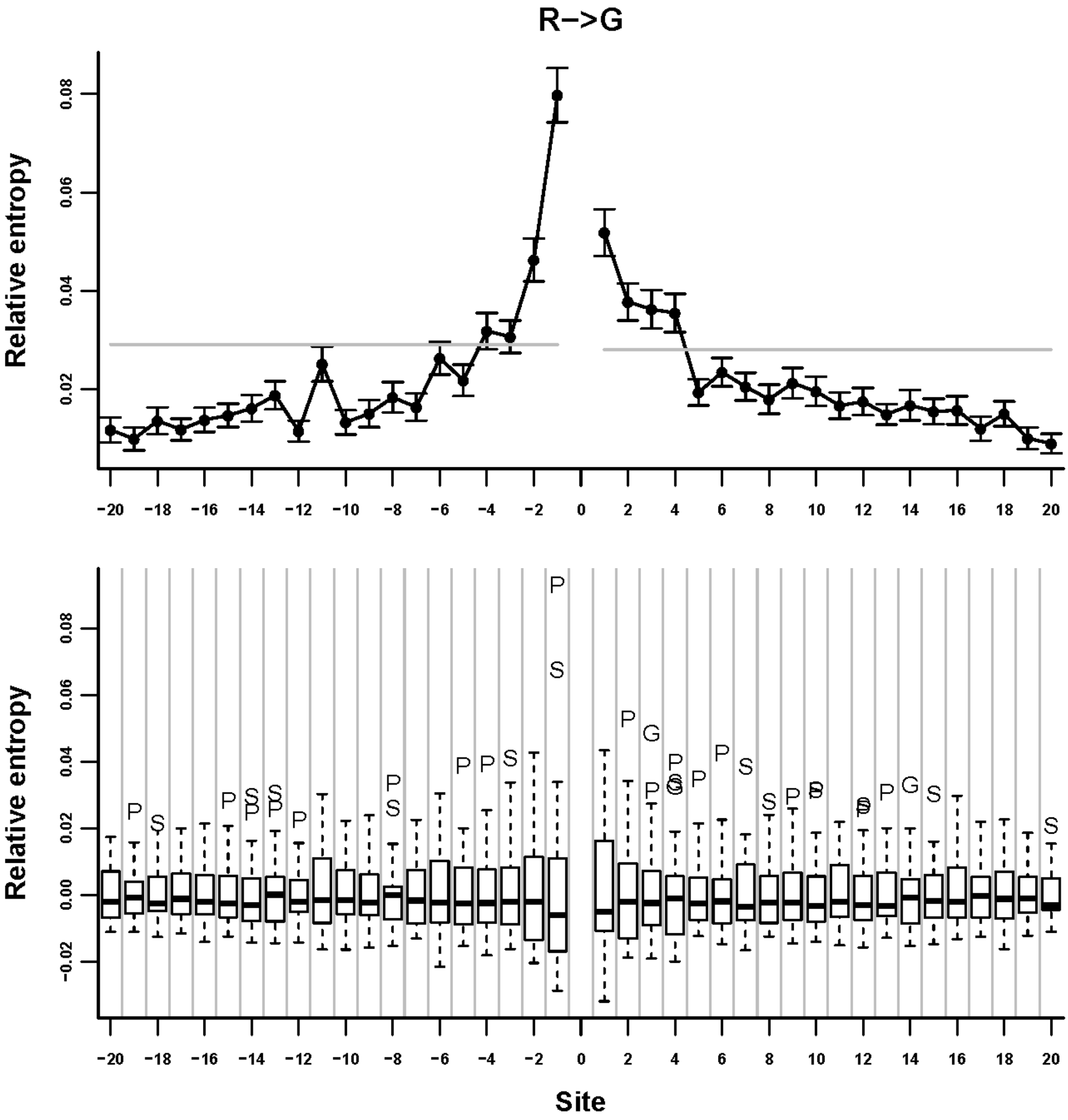

R->G
Relative entropy
0.08
0.06
0.04
0.02
-20 -18 -16 -14 -12 -10 -8 -6 -4 -2 0 2 4 6 8 10 12 14 16 18 20
Relative entropy
0.08
0.06
0.04
0.02
0.00
-0.02
P
S
P
G
S
Site

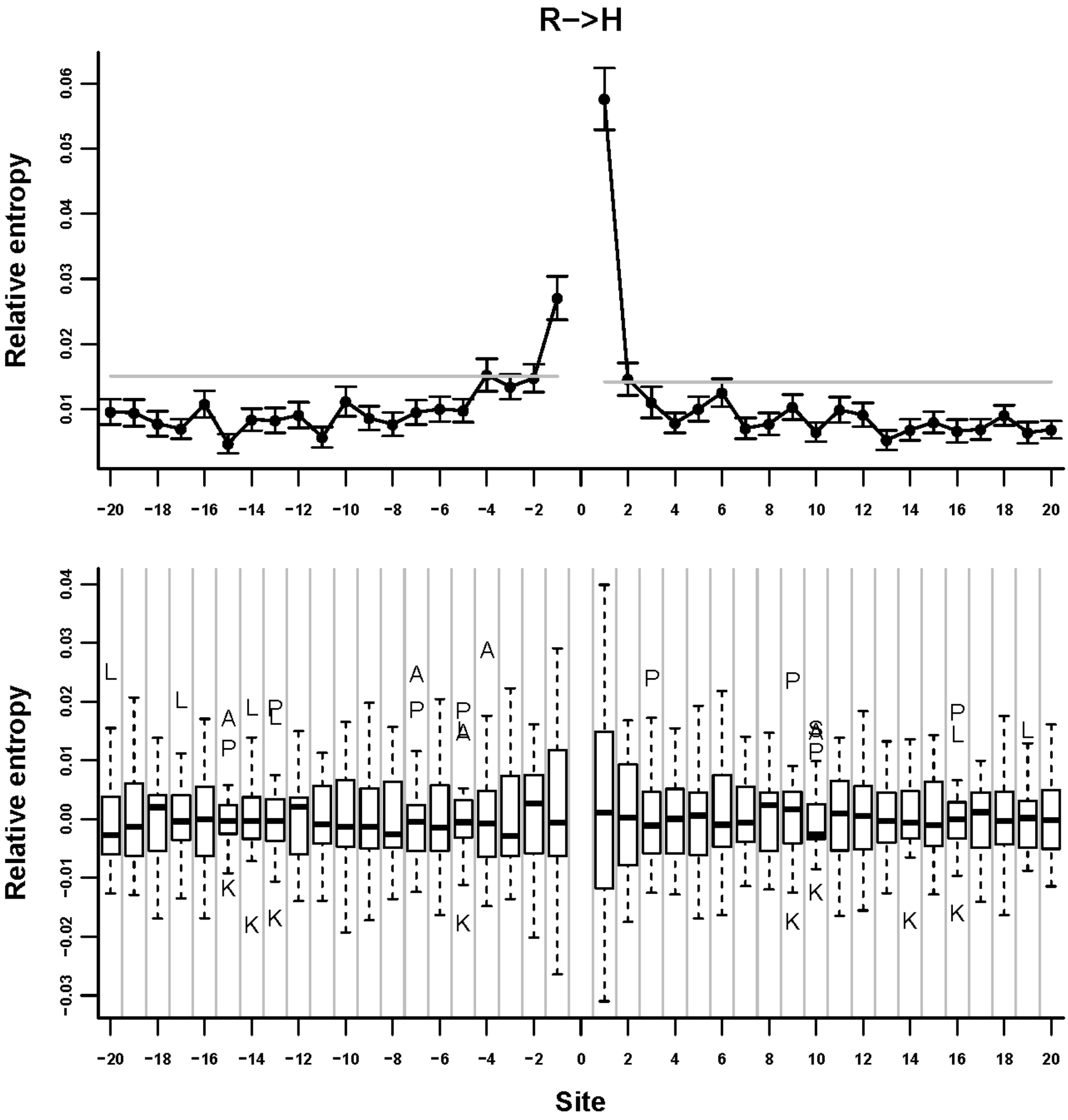
R−>H
Relative entropy
Relative entropy
Site

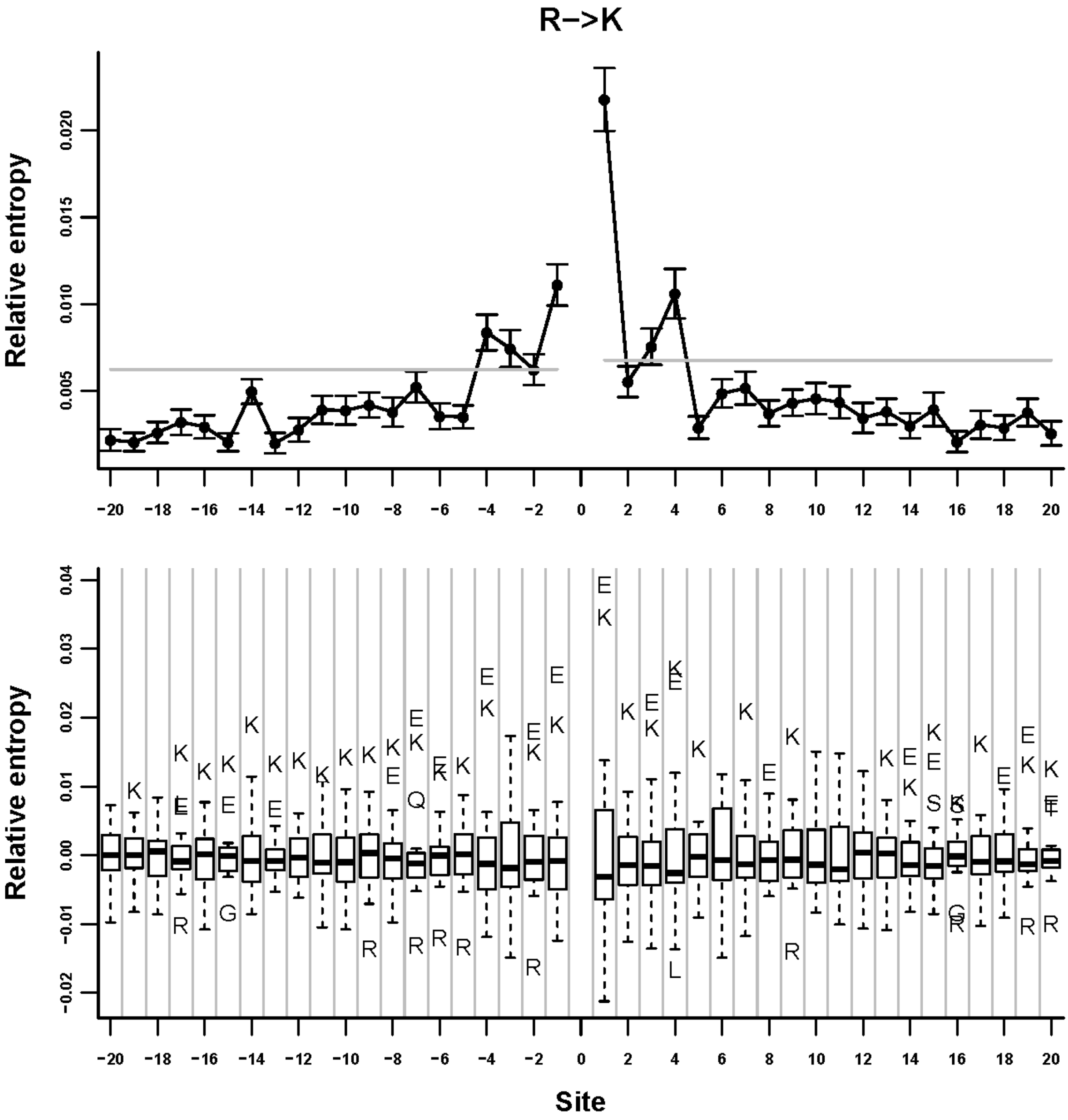
R−>K
Relative entropy
Site

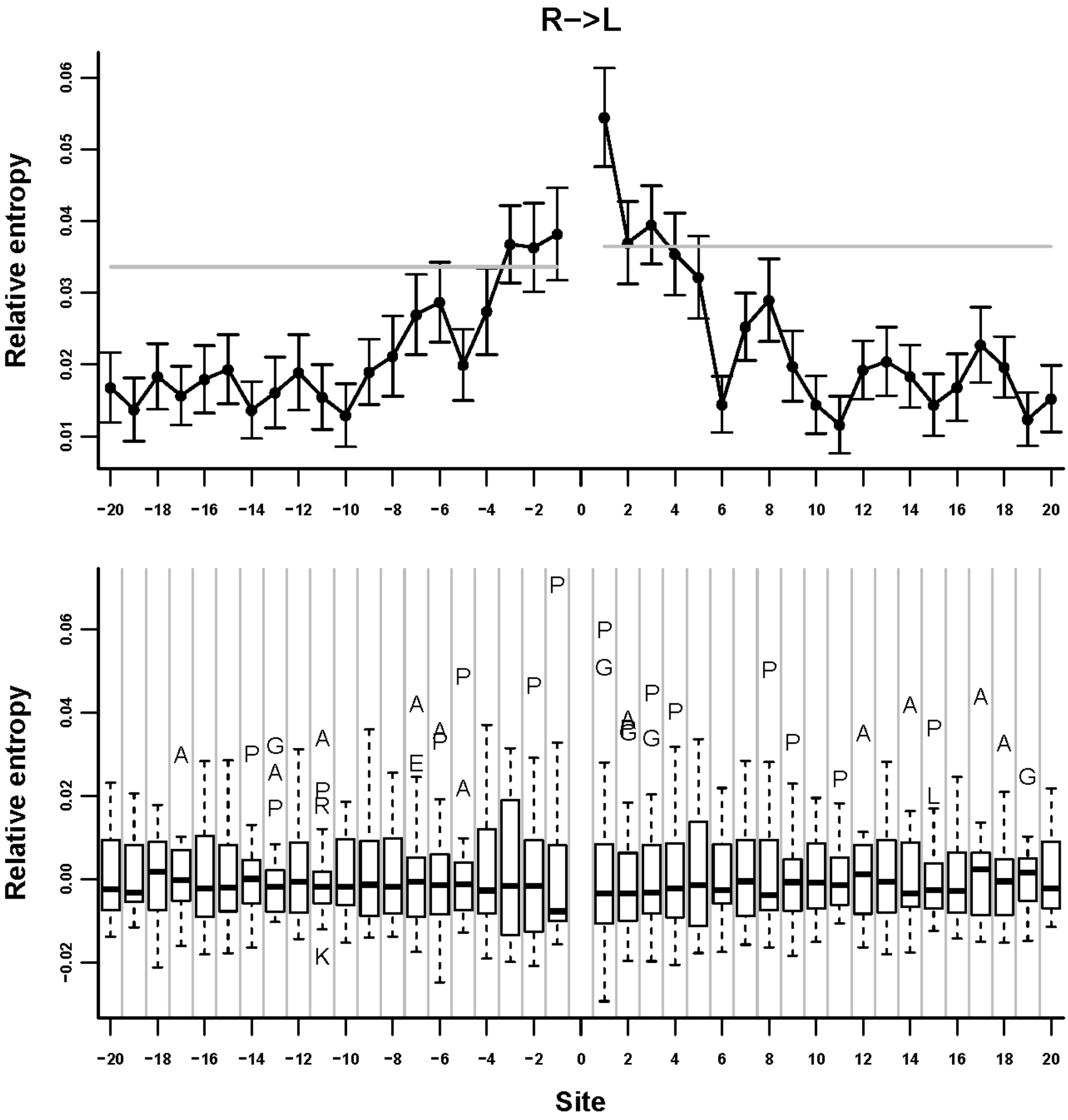
R−>L
Relative entropy
Site

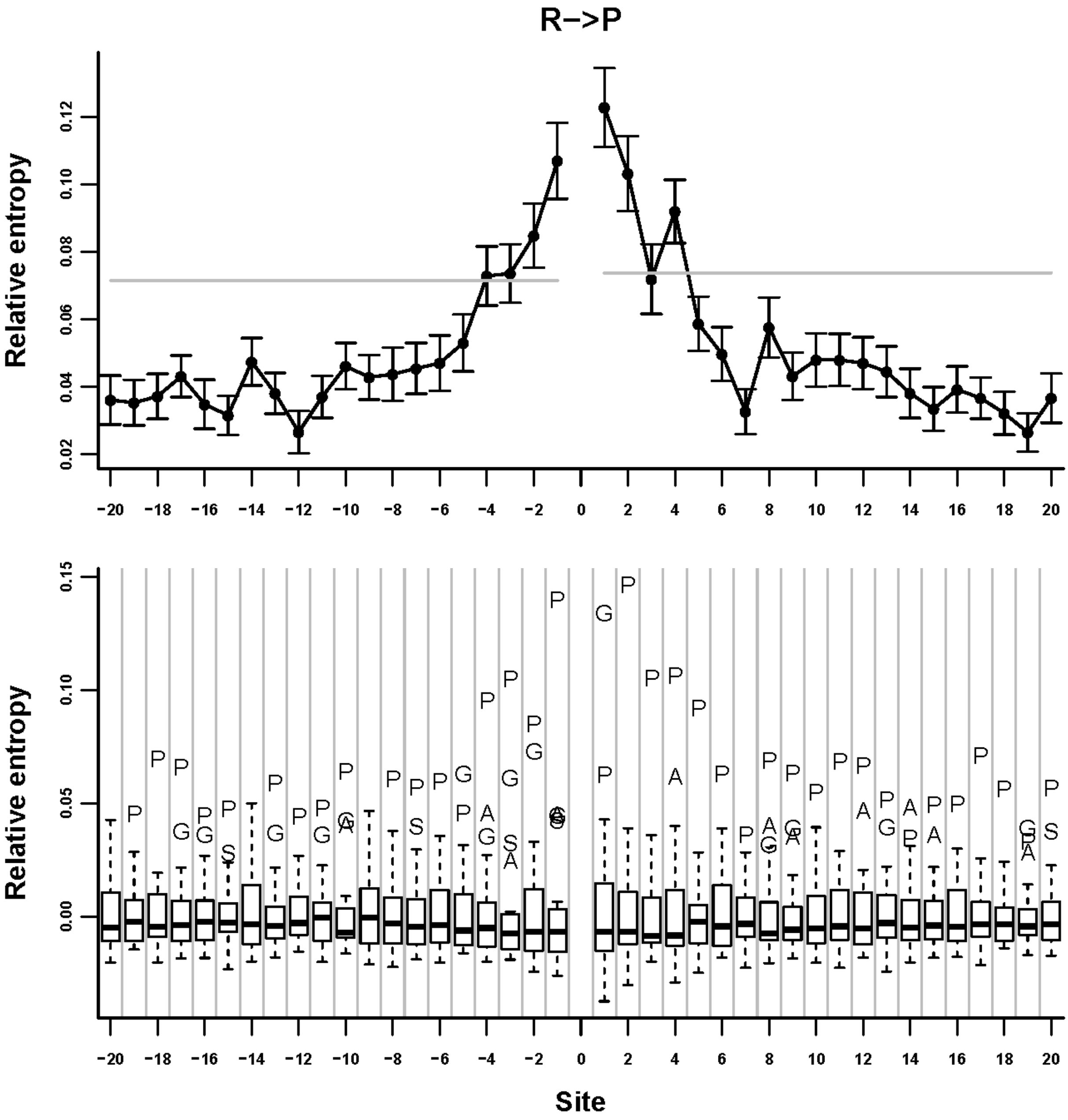
R−>P
Relative entropy
Relative entropy
Site

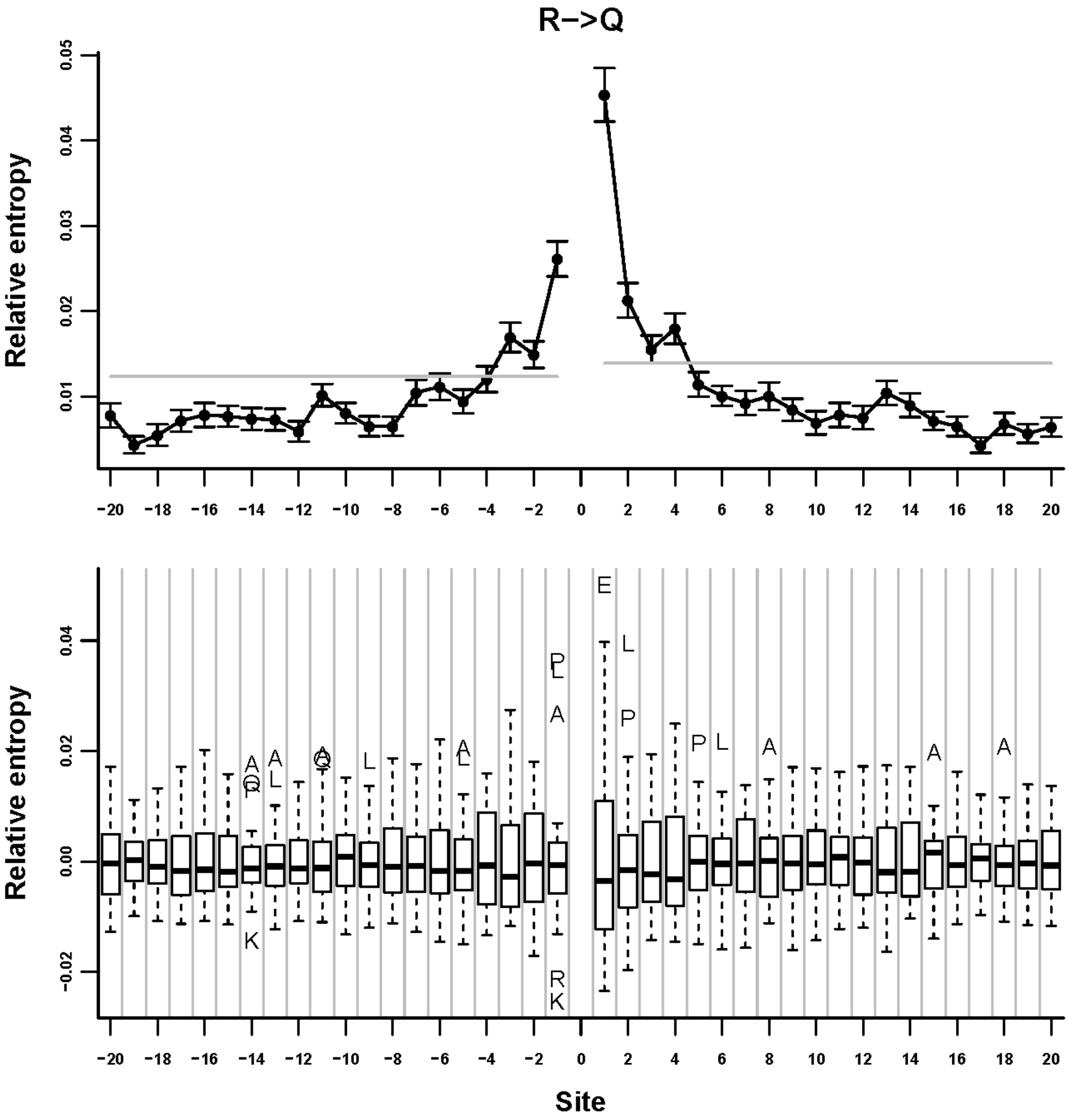
R−>Q
Relative entropy
0.01
0.02
0.03
0.04
0.05
−20 −18 −16 −14 −12 −10 −8 −6 −4 −2 0 2 4 6 8 10 12 14 16 18 20
Relative entropy
−0.02
0.00
0.02
0.04
−20 −18 −16 −14 −12 −10 −8 −6 −4 −2 0 2 4 6 8 10 12 14 16 18 20
Site

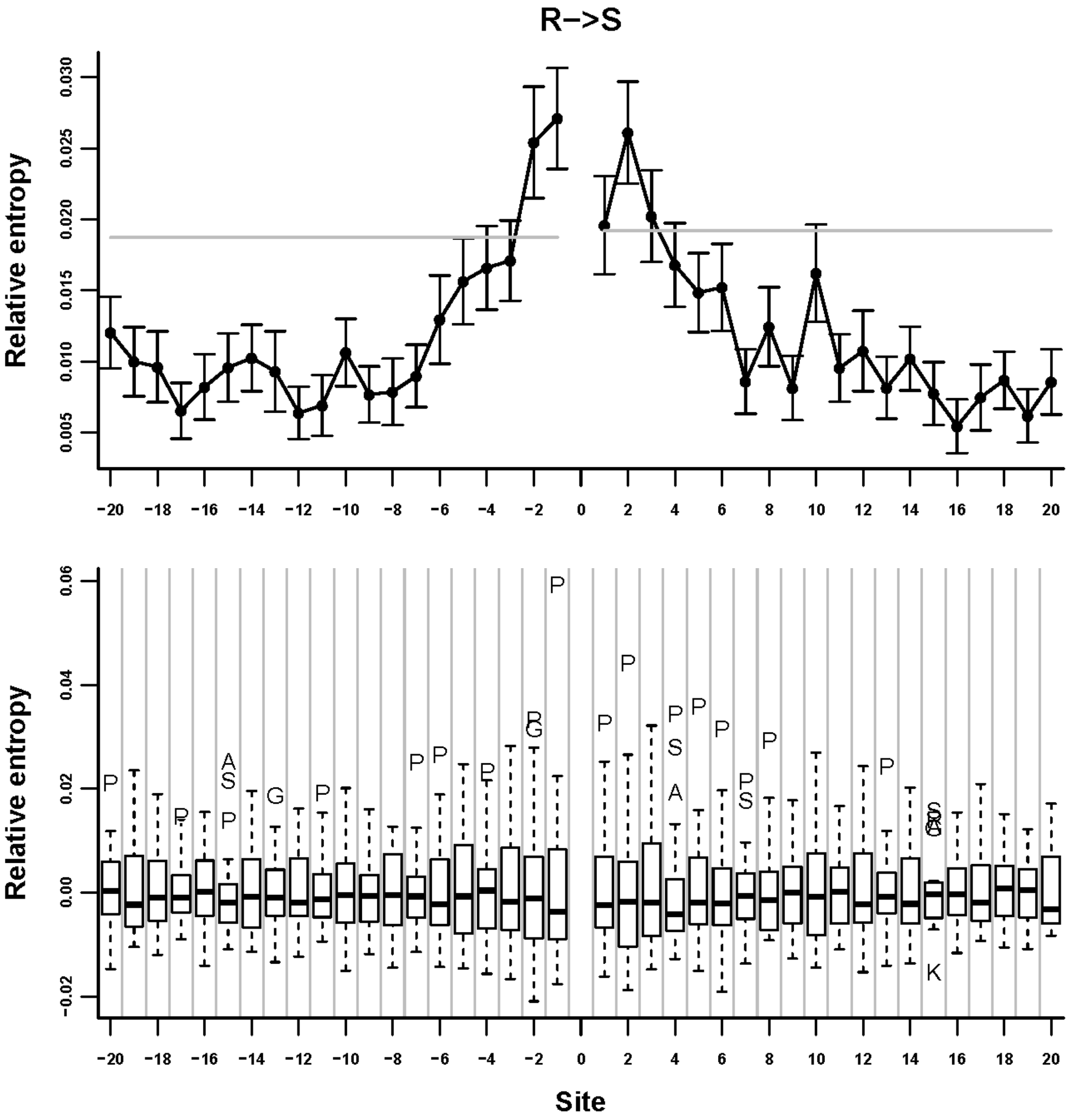
R−>S
Relative entropy
Relative entropy
Site

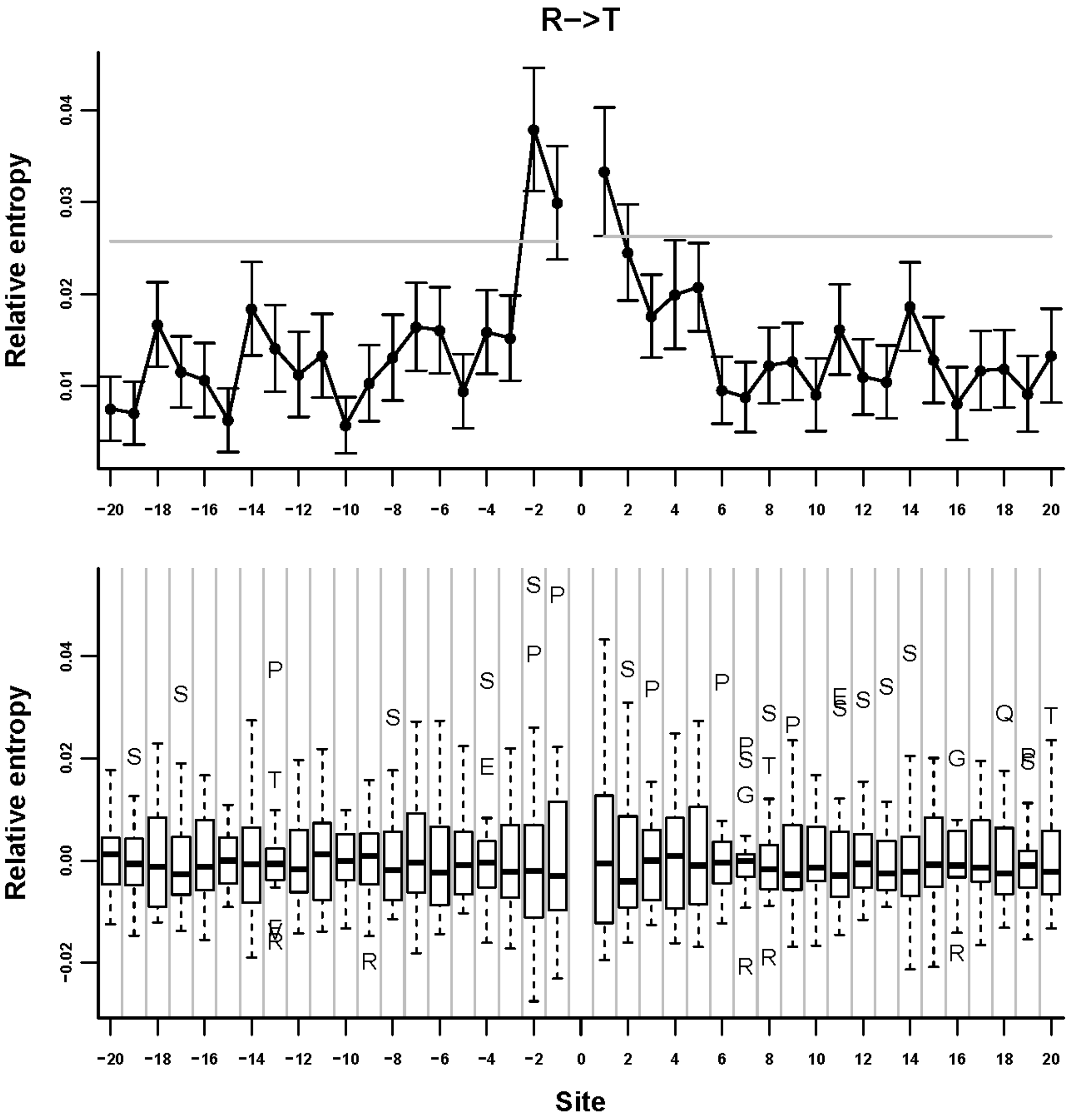
R−>T
Relative entropy
Relative entropy
Site

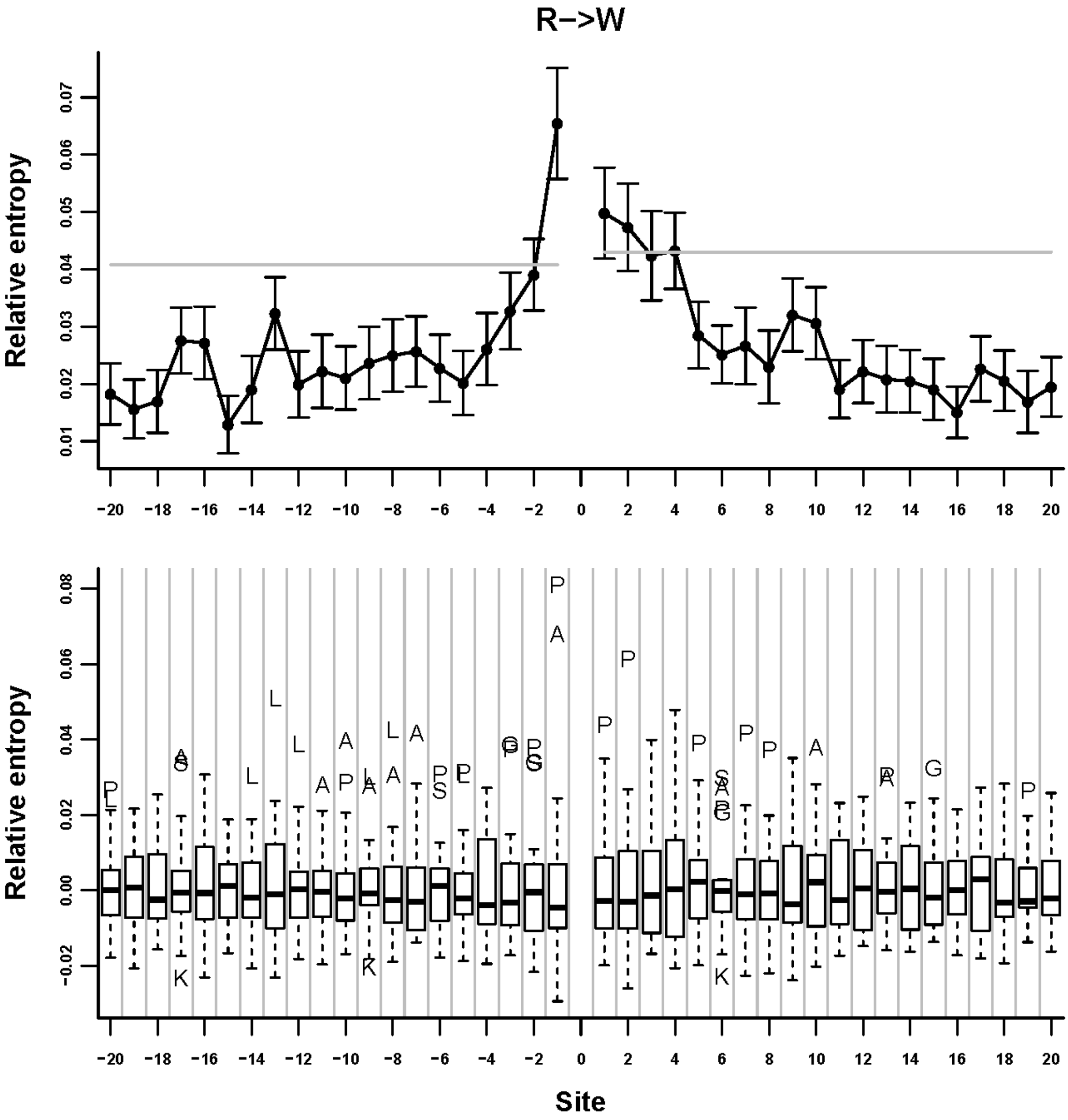
R−>W
Relative entropy
Relative entropy
Site

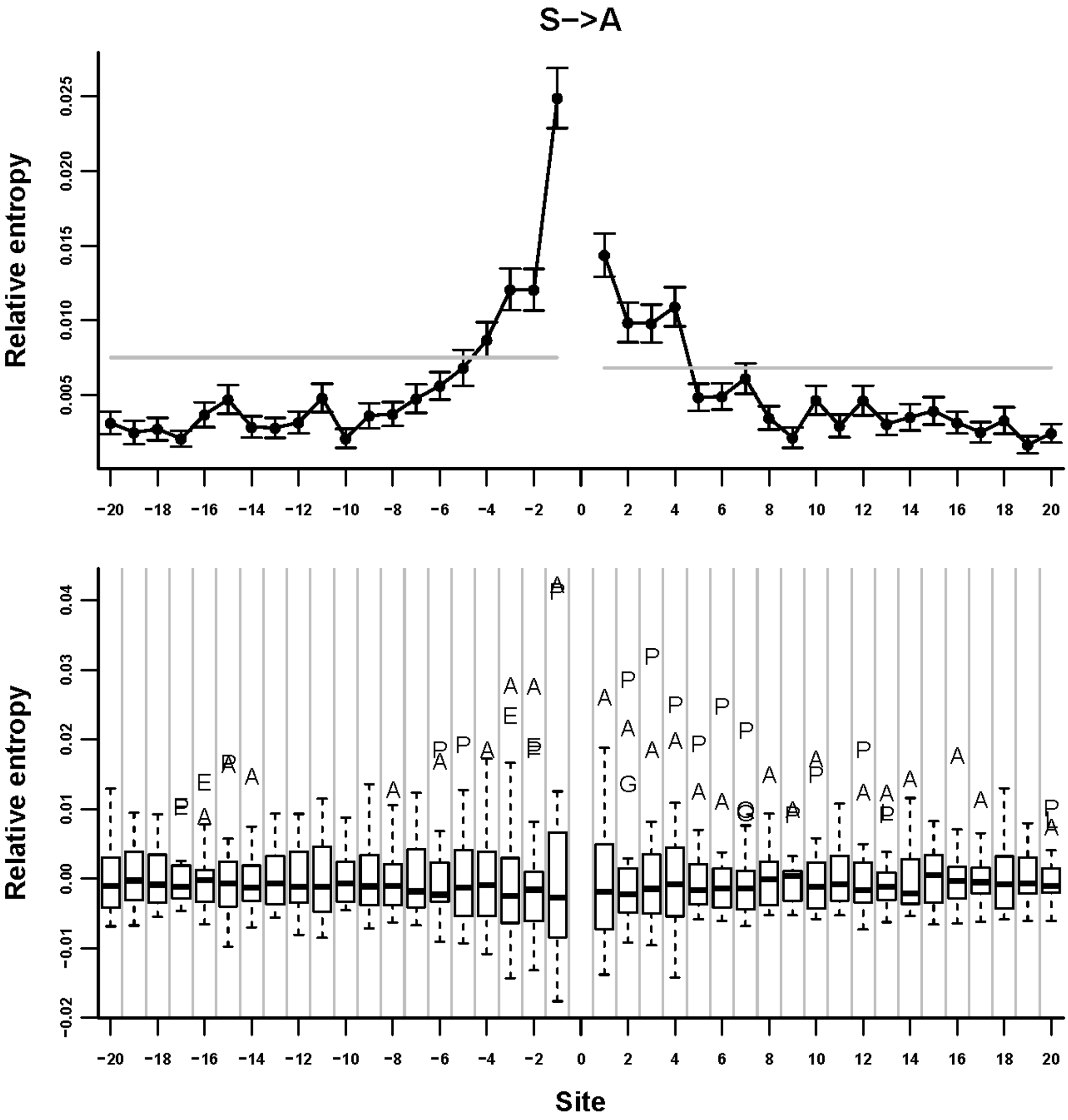
S−>A
Relative entropy
Site

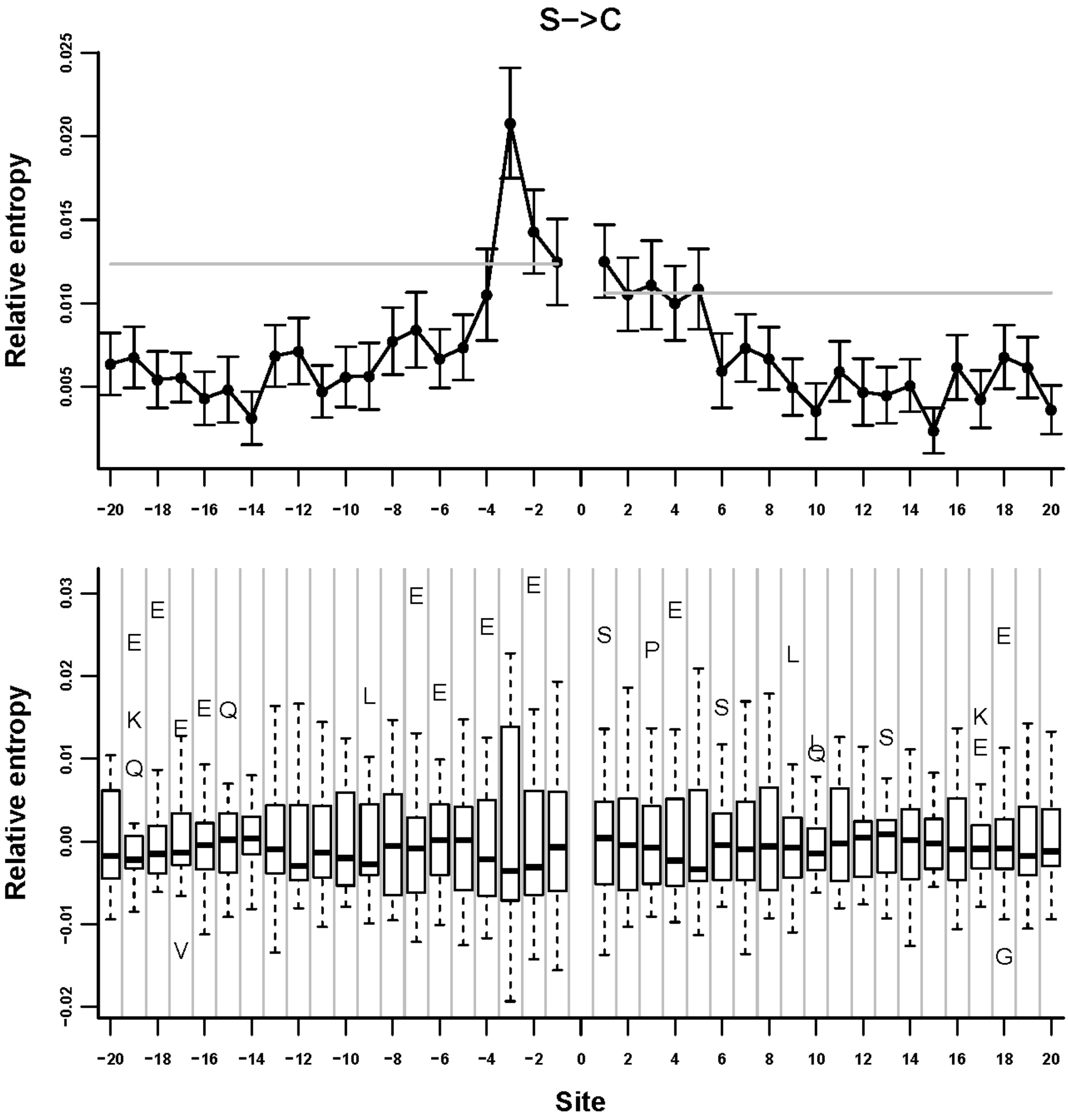
S−>C
Relative entropy
Site
E
K
Q
V
L
P
S
G

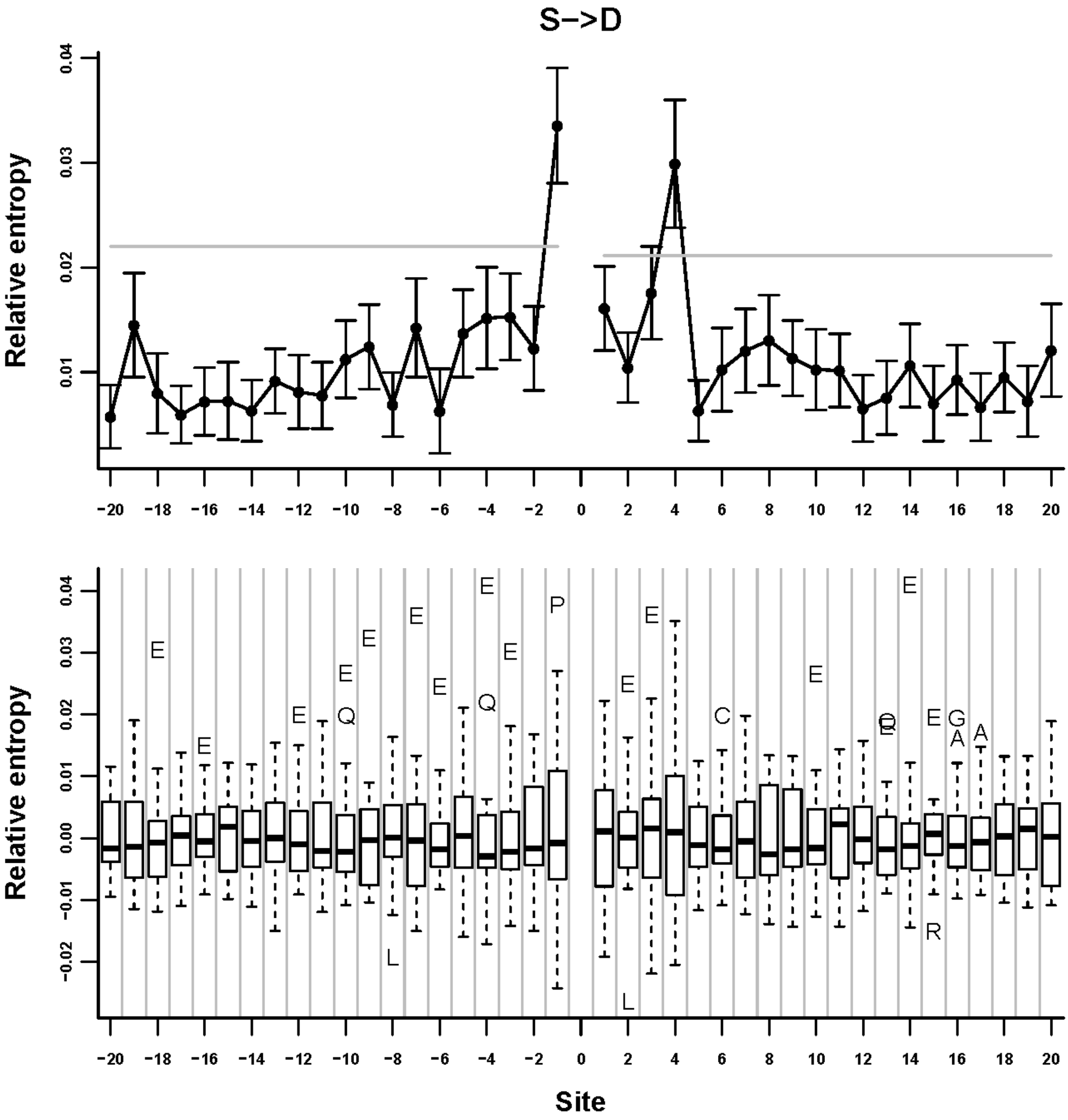

S−>D
Relative entropy
Site

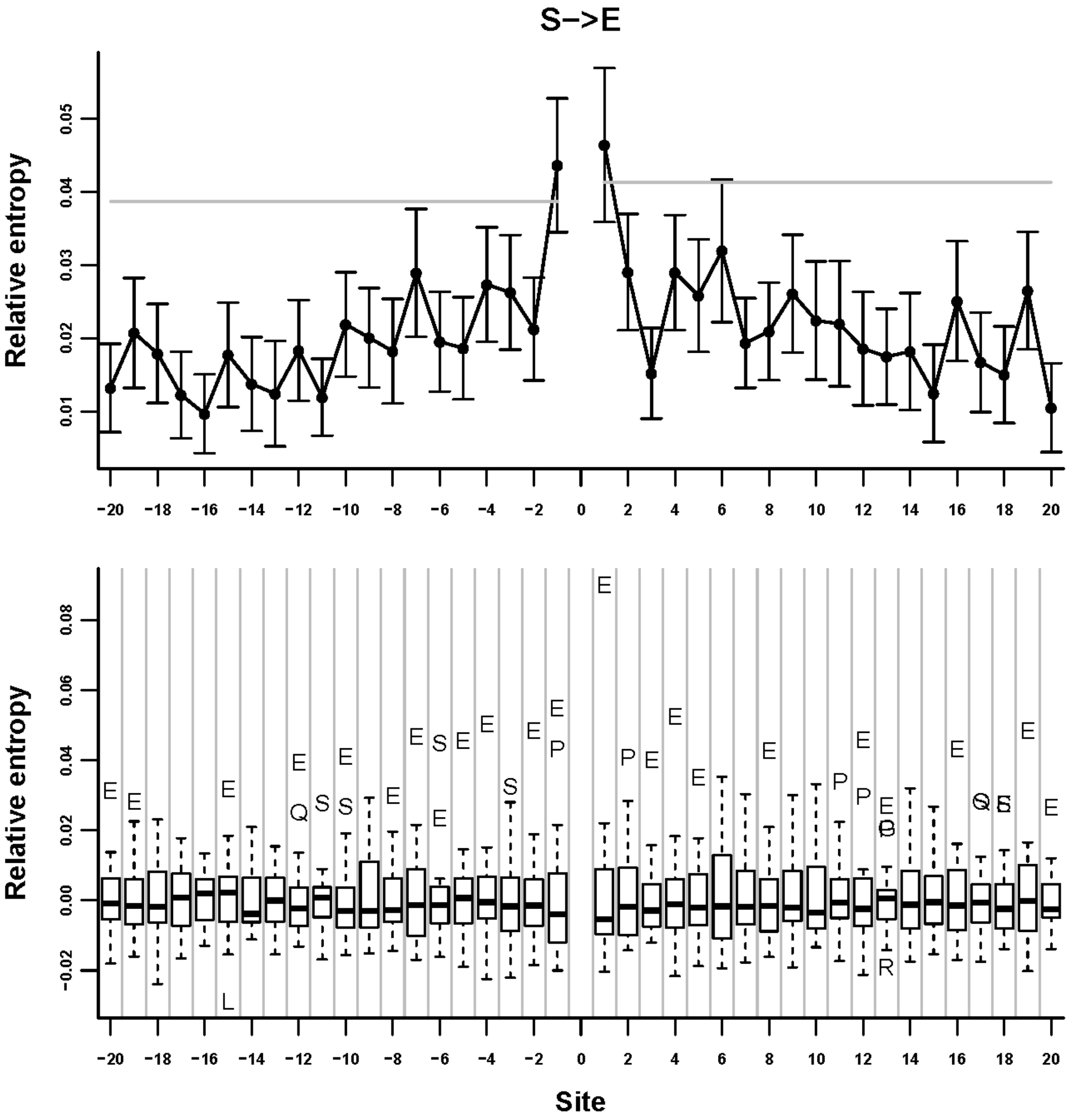
S−>E
Relative entropy
Site

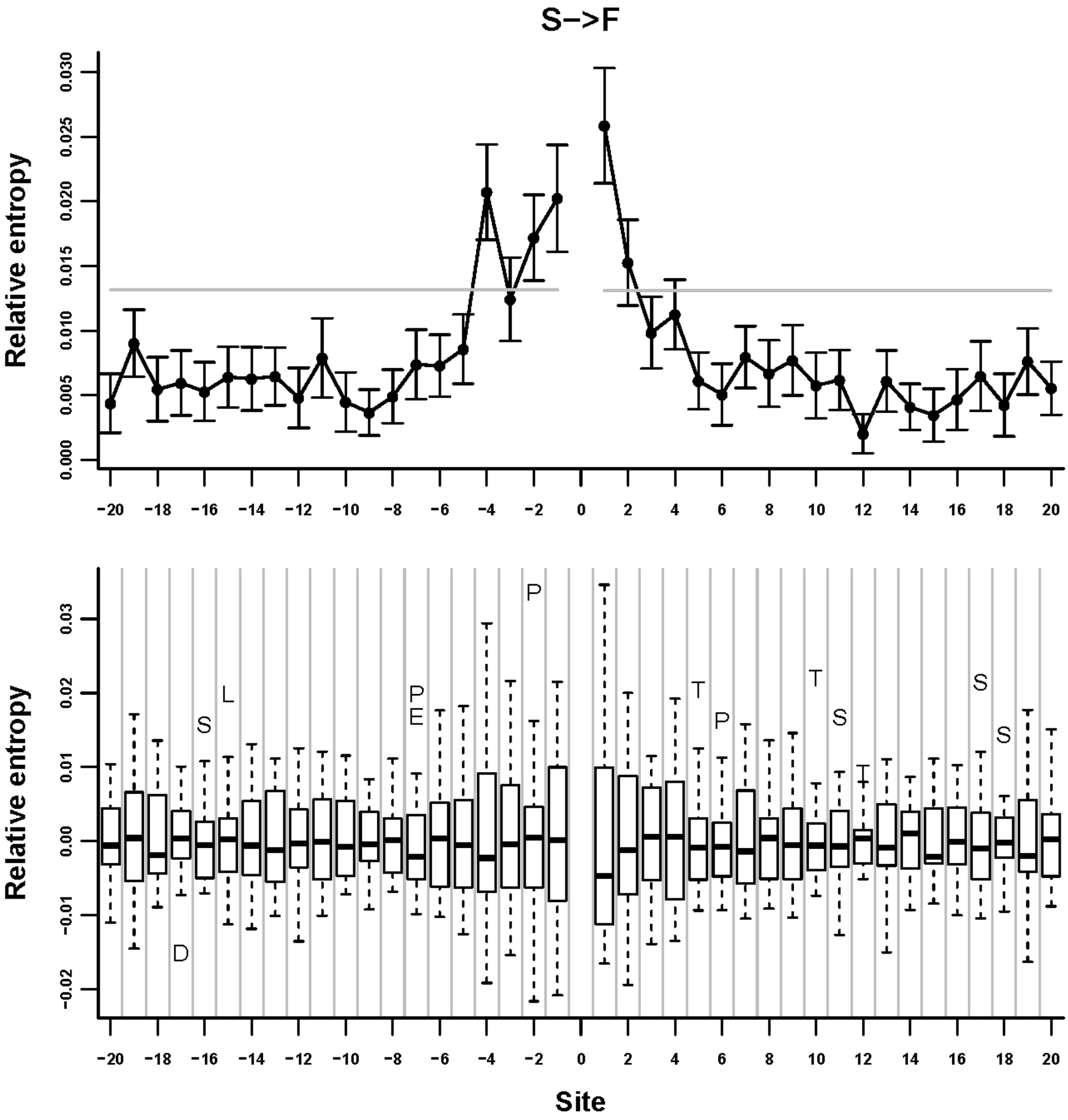
S−>F
Relative entropy
0.000
0.005
0.010
0.015
0.020
0.025
0.030
−20 −18 −16 −14 −12 −10 −8 −6 −4 −2 0 2 4 6 8 10 12 14 16 18 20
Relative entropy
−0.02
−0.01
0.00
0.01
0.02
0.03
S
L
D
P
E
P
T
P
T
S
S
S
−20 −18 −16 −14 −12 −10 −8 −6 −4 −2 0 2 4 6 8 10 12 14 16 18 20
Site

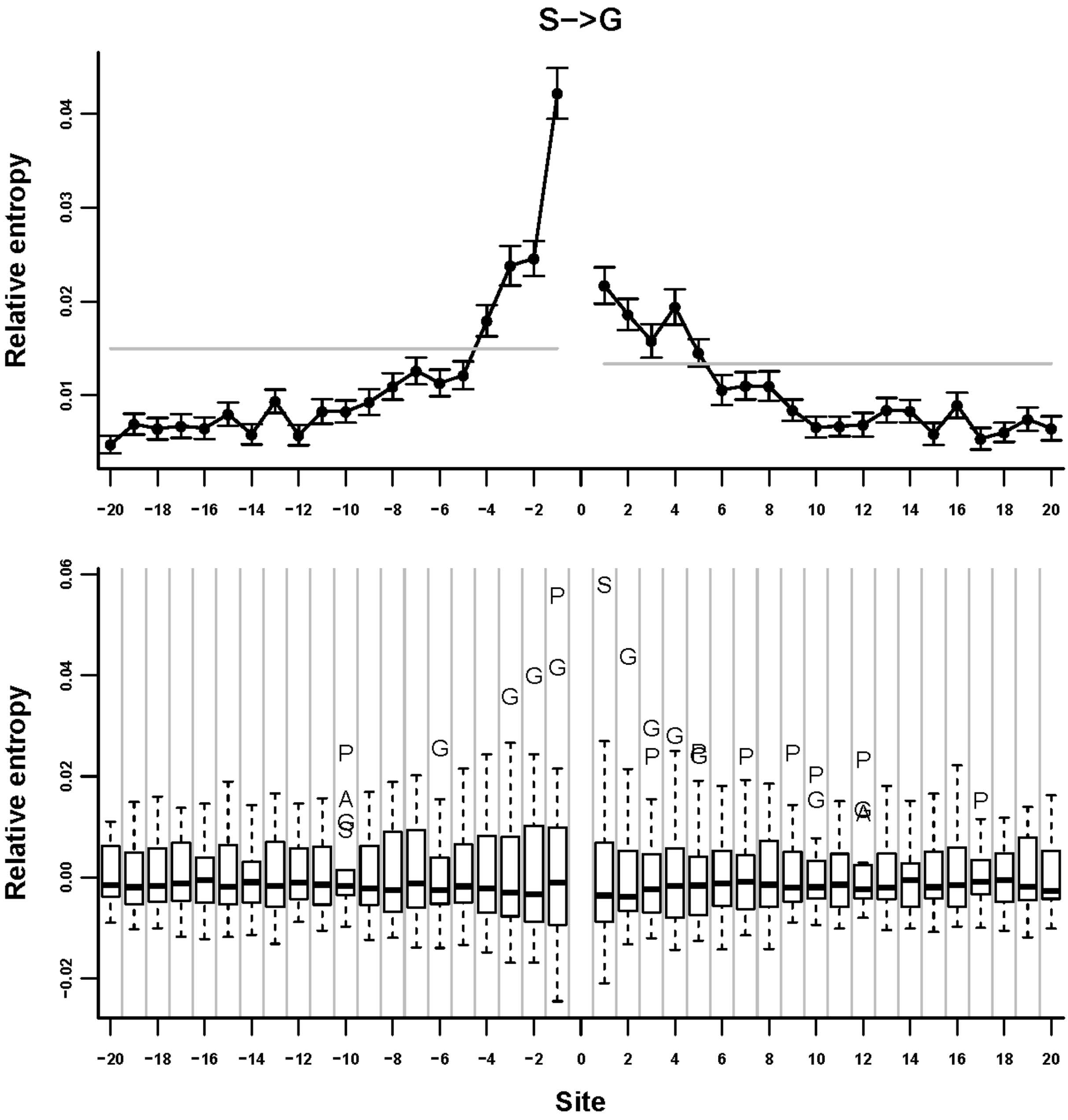
S−>G
Relative entropy
Relative entropy
Site

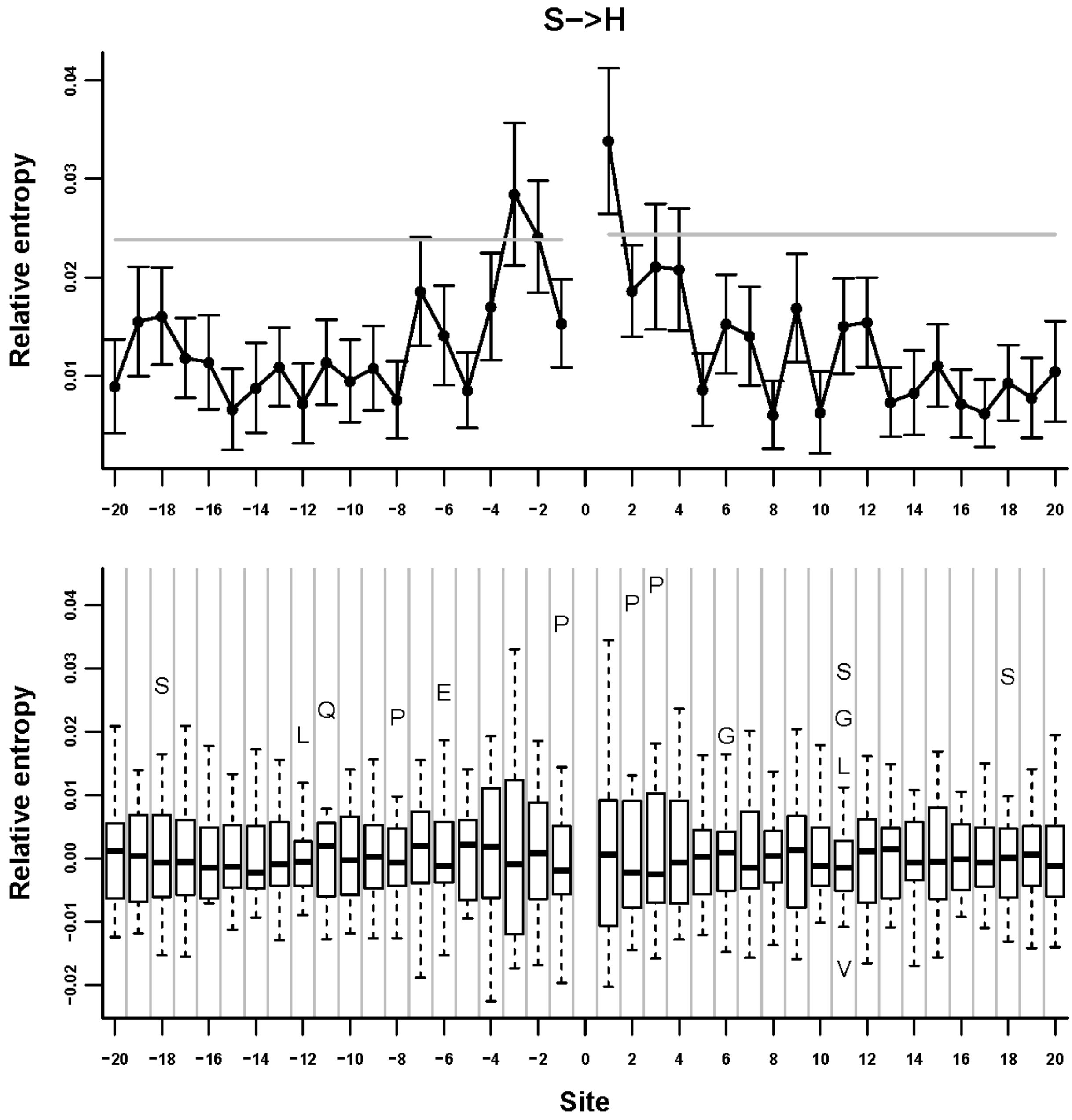
S−>H
Relative entropy
0.01
0.02
0.03
0.04
−20
−18
−16
−14
−12
−10
−8
−6
−4
−2
0
2
4
6
8
10
12
14
16
18
20
Relative entropy
−0.02
−0.01
0.00
0.01
0.02
0.03
0.04
S
L
Q
P
E
P
P
P
G
S
G
L
V
S
Site

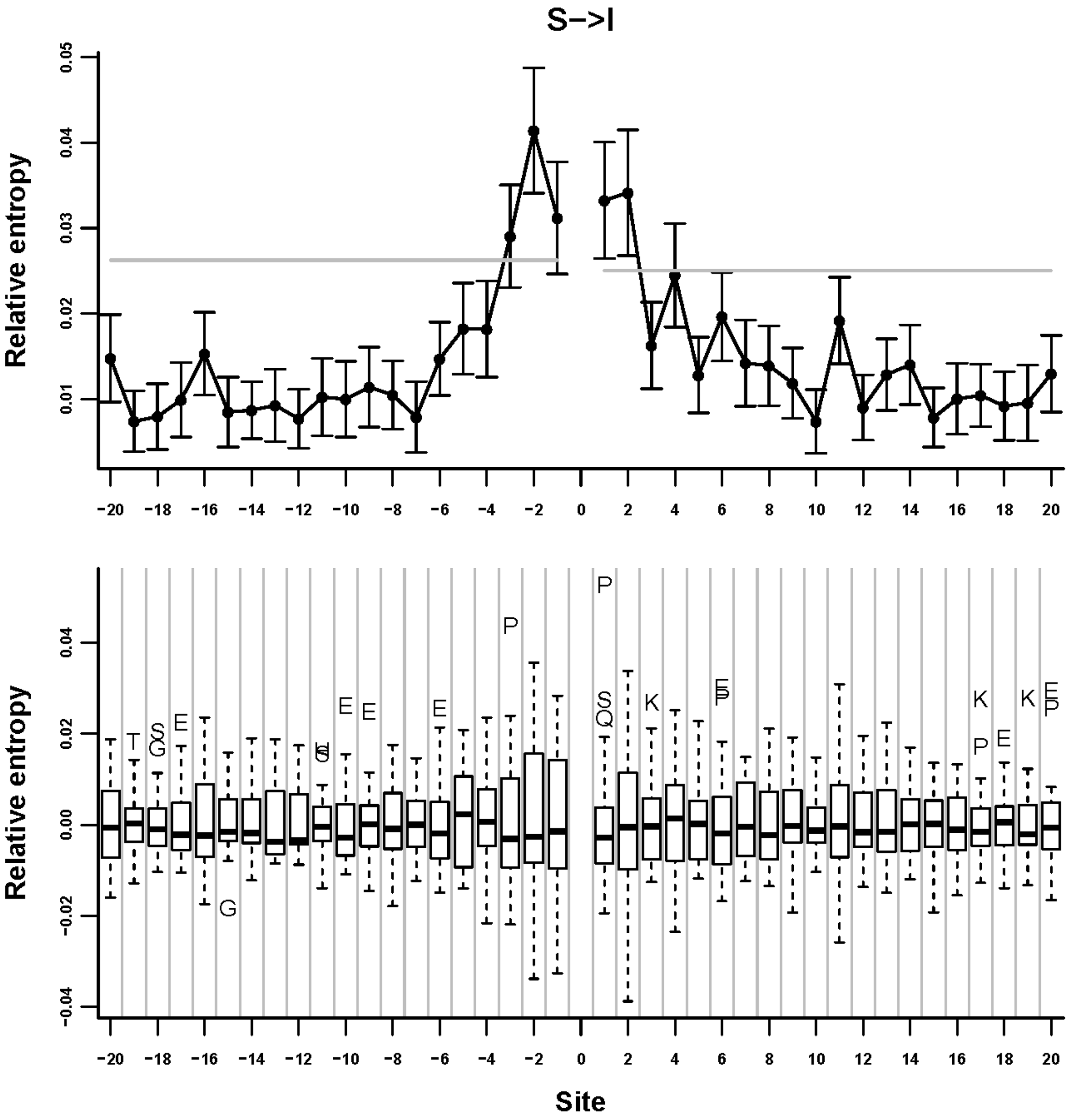

S−>I
Relative entropy
0.01
0.02
0.03
0.04
0.05
−20 −18 −16 −14 −12 −10 −8 −6 −4 −2 0 2 4 6 8 10 12 14 16 18 20
Relative entropy
−0.04
−0.02
0.00
0.02
0.04
Site

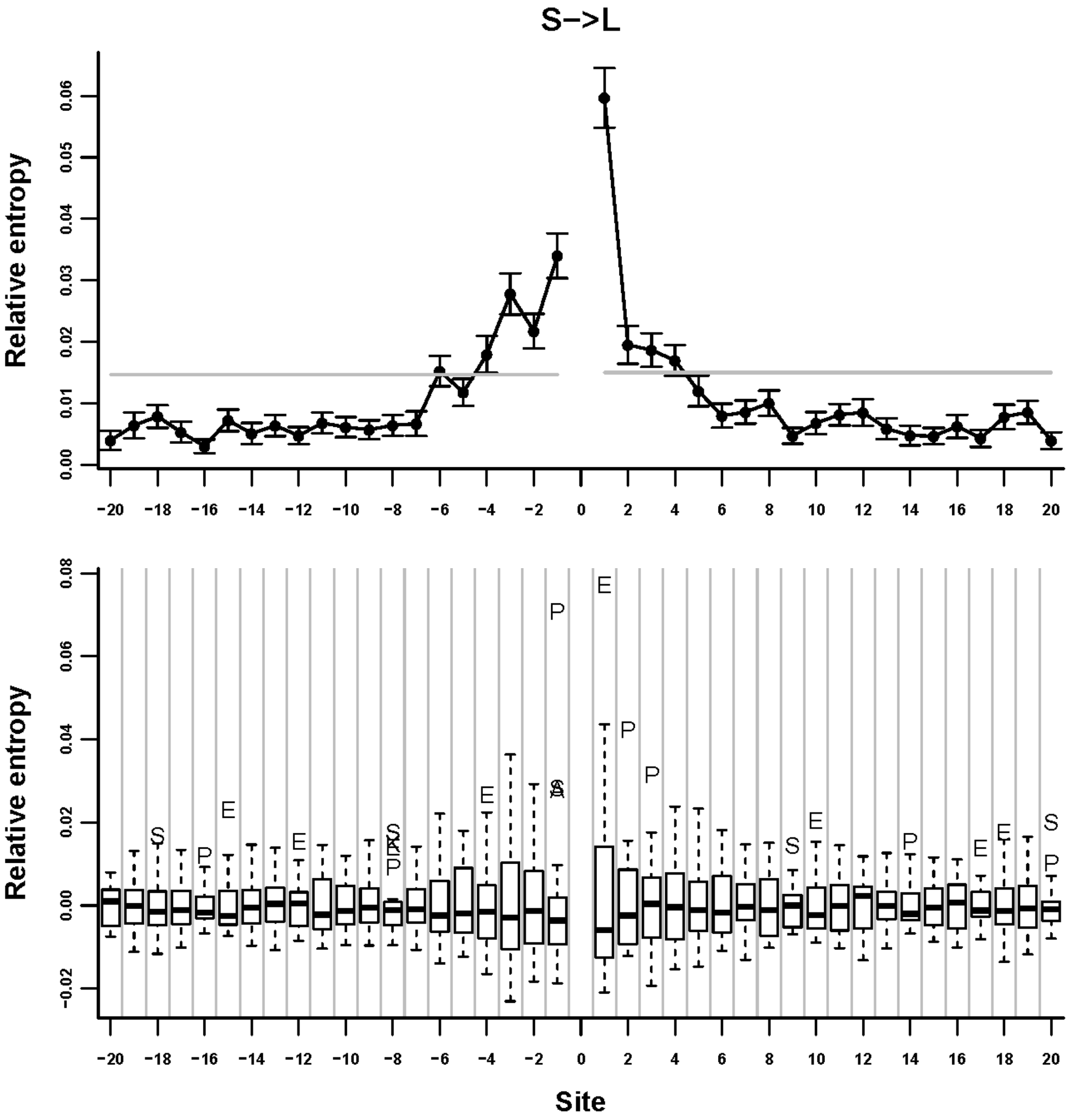
S−>L
Relative entropy
Relative entropy
Site

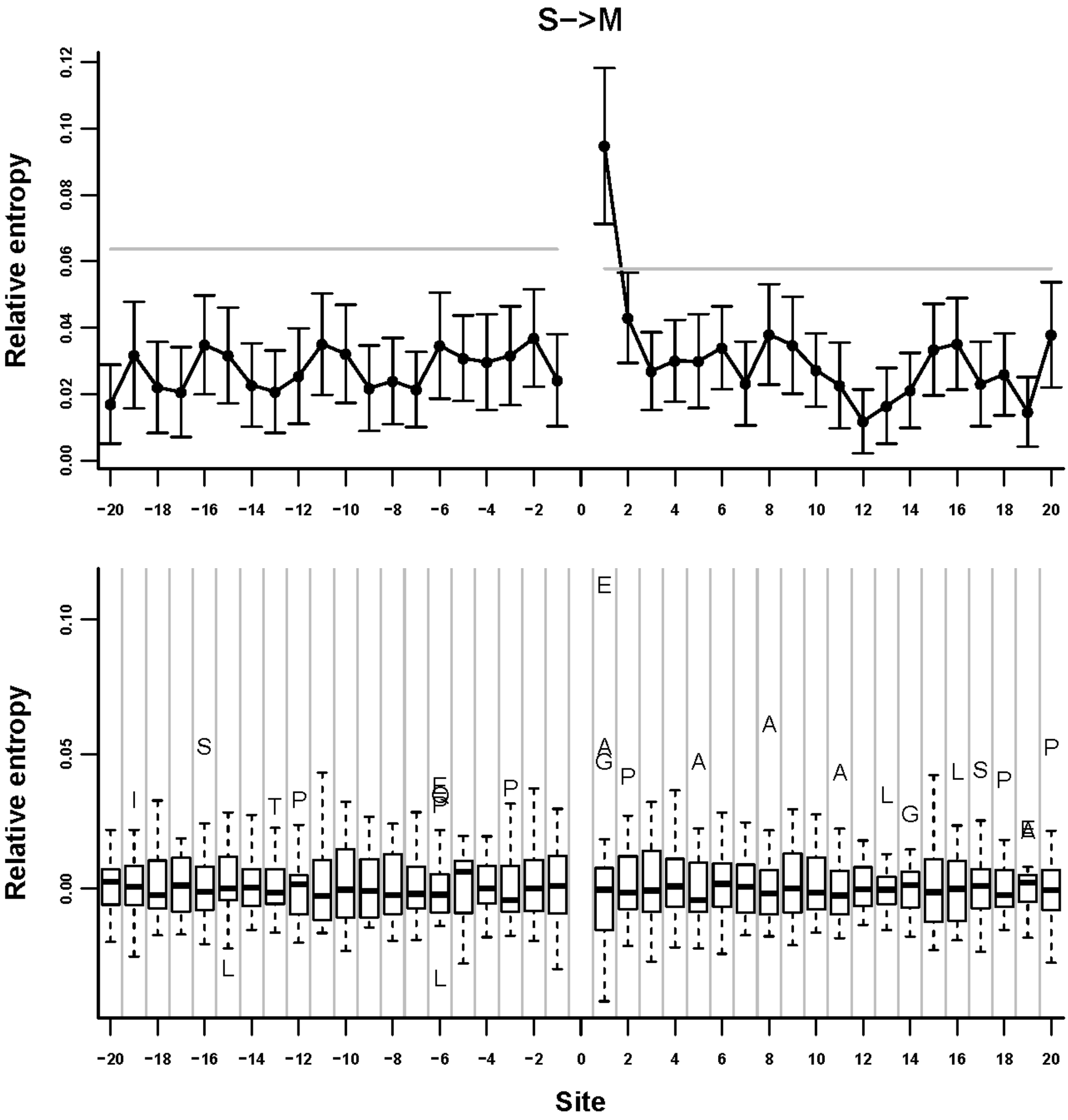
S−>M
Relative entropy
Relative entropy
Site

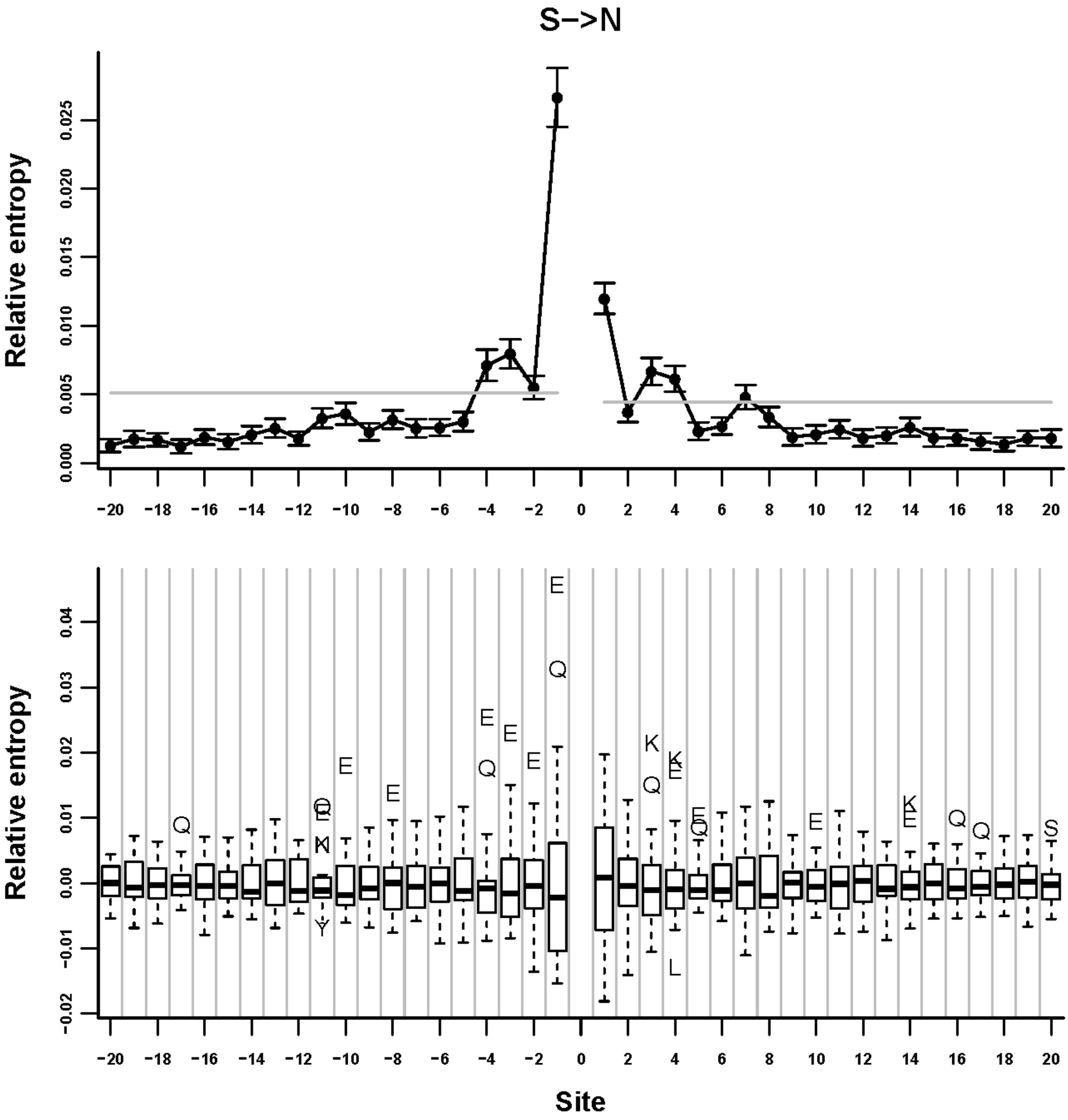
S−>N
Relative entropy
Relative entropy
Site

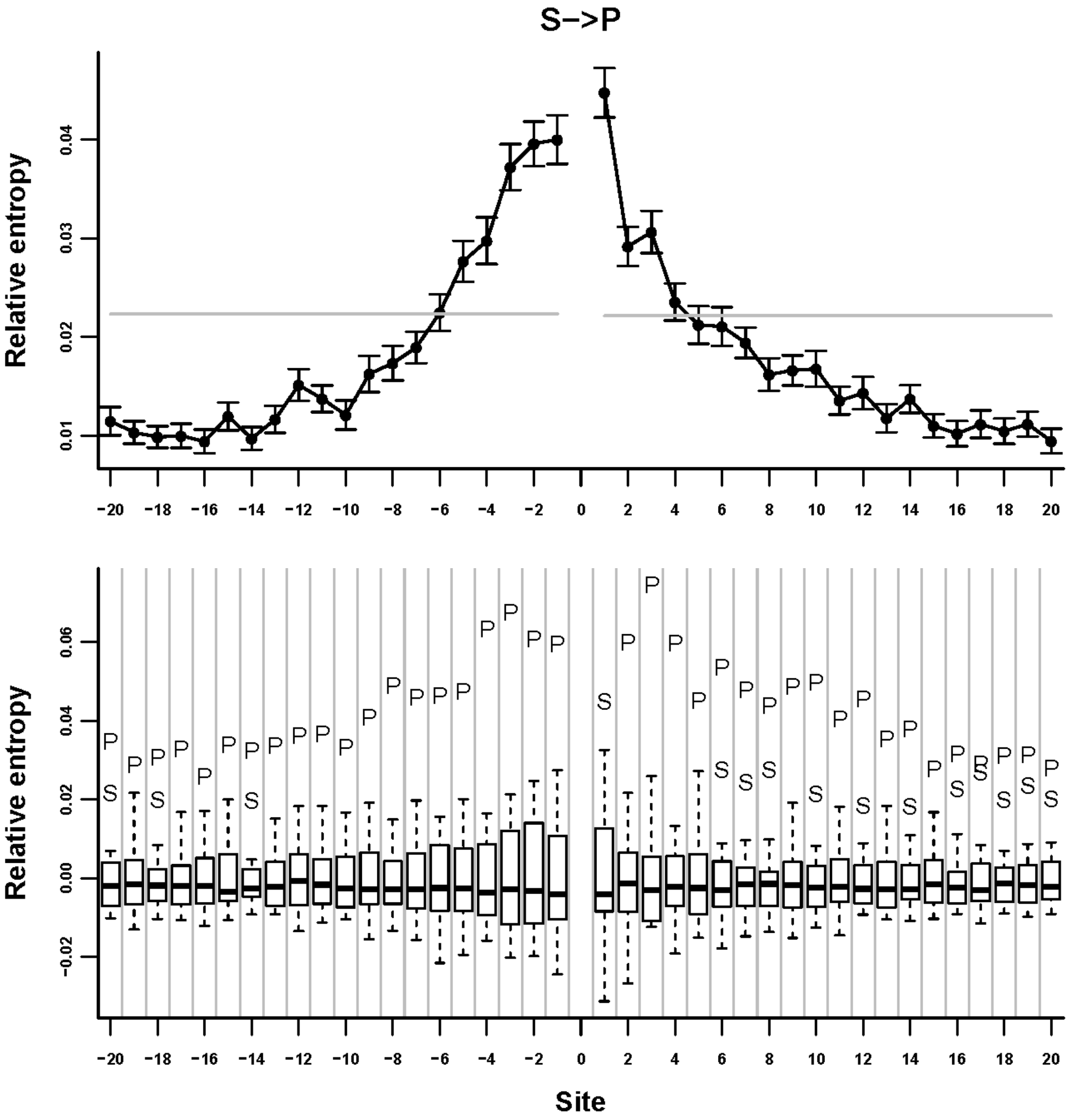
S−>P
Relative entropy
0.01
0.02
0.03
0.04
−20 −18 −16 −14 −12 −10 −8 −6 −4 −2 0 2 4 6 8 10 12 14 16 18 20
Relative entropy
−0.02
0.00
0.02
0.04
0.06
−20 −18 −16 −14 −12 −10 −8 −6 −4 −2 0 2 4 6 8 10 12 14 16 18 20
Site

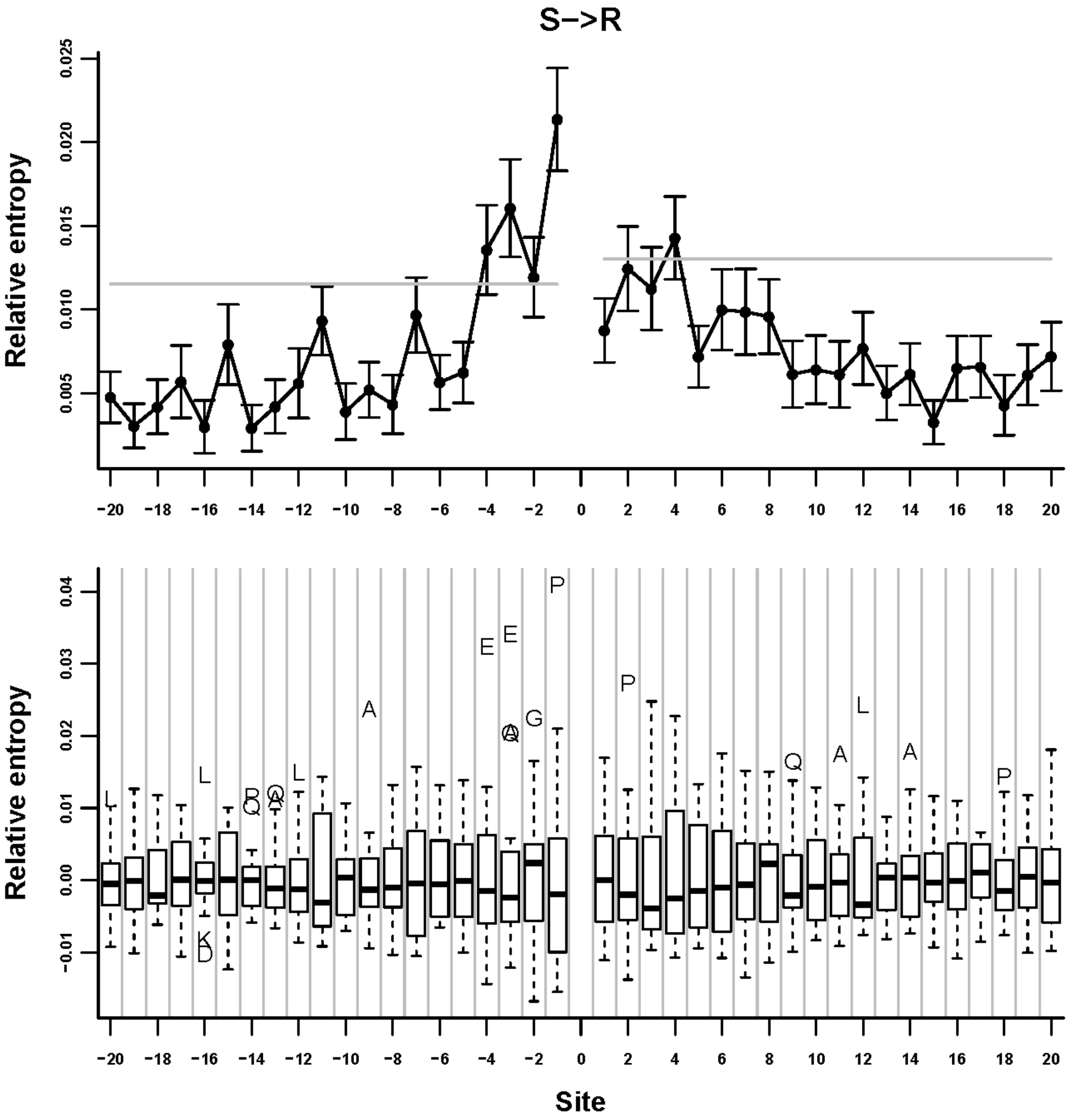
S−>R
Relative entropy
0.005
0.010
0.015
0.020
0.025
−20 −18 −16 −14 −12 −10 −8 −6 −4 −2 0 2 4 6 8 10 12 14 16 18 20
Relative entropy
-0.01
0.00
0.01
0.02
0.03
0.04
L L R Q Q A L A E E Q A G P P L Q A A A P K D
Site

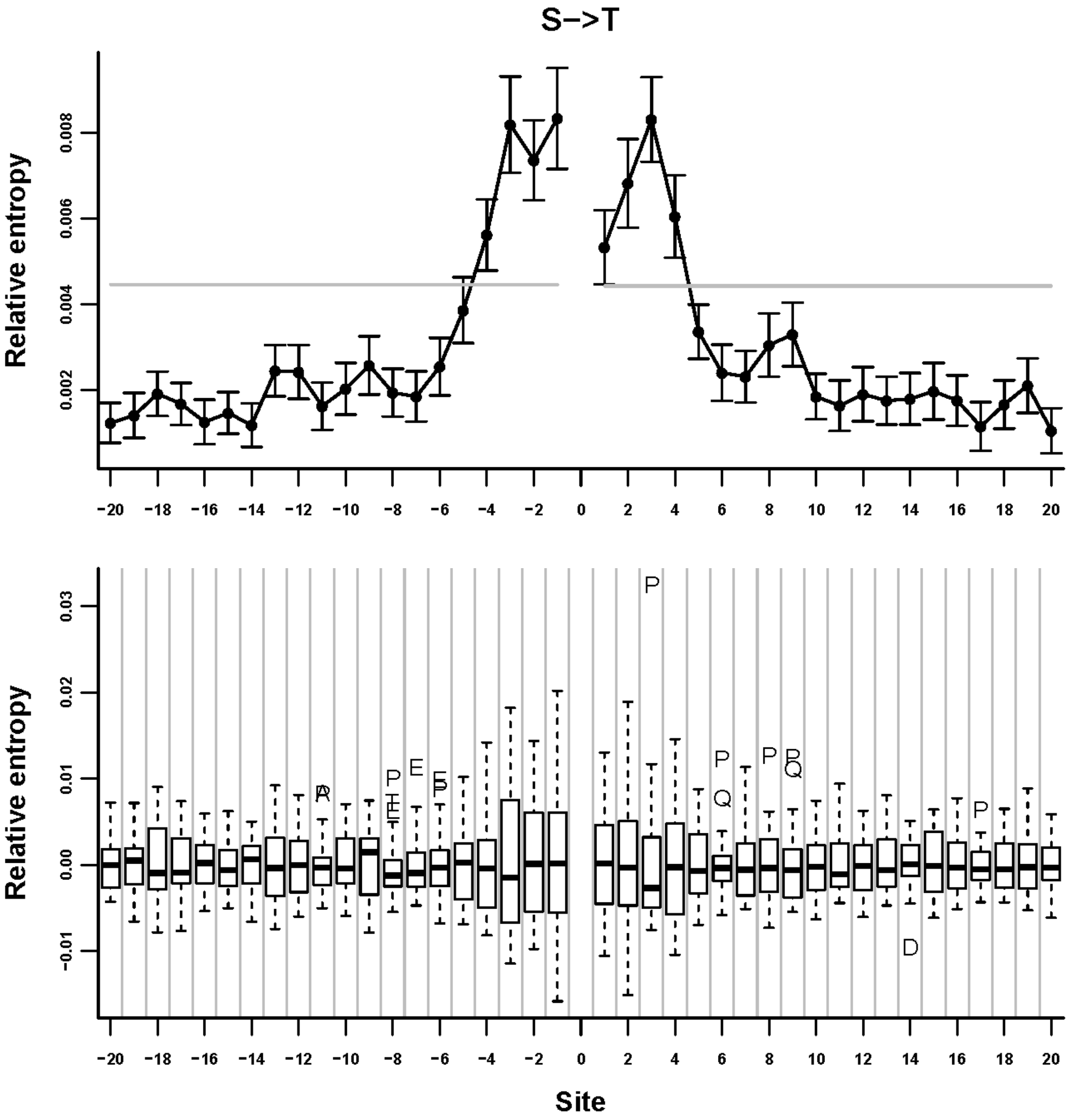
S−>T
Relative entropy
0.002
0.004
0.006
0.008
−20 −18 −16 −14 −12 −10 −8 −6 −4 −2 0 2 4 6 8 10 12 14 16 18 20
Relative entropy
-0.01
0.00
0.01
0.02
0.03
P
E
P
Q
D
Site

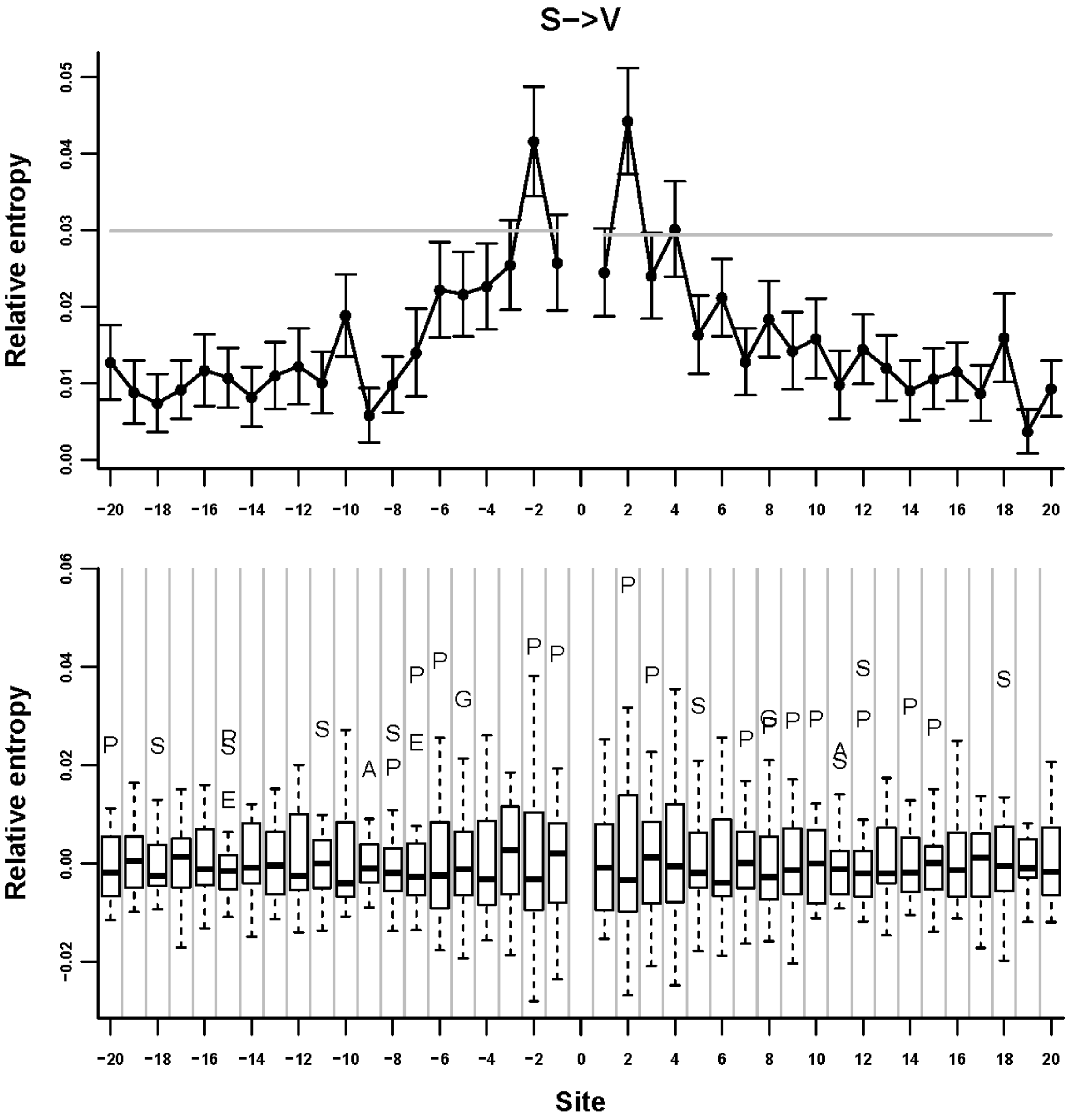
S−>V
Relative entropy
Relative entropy
Site

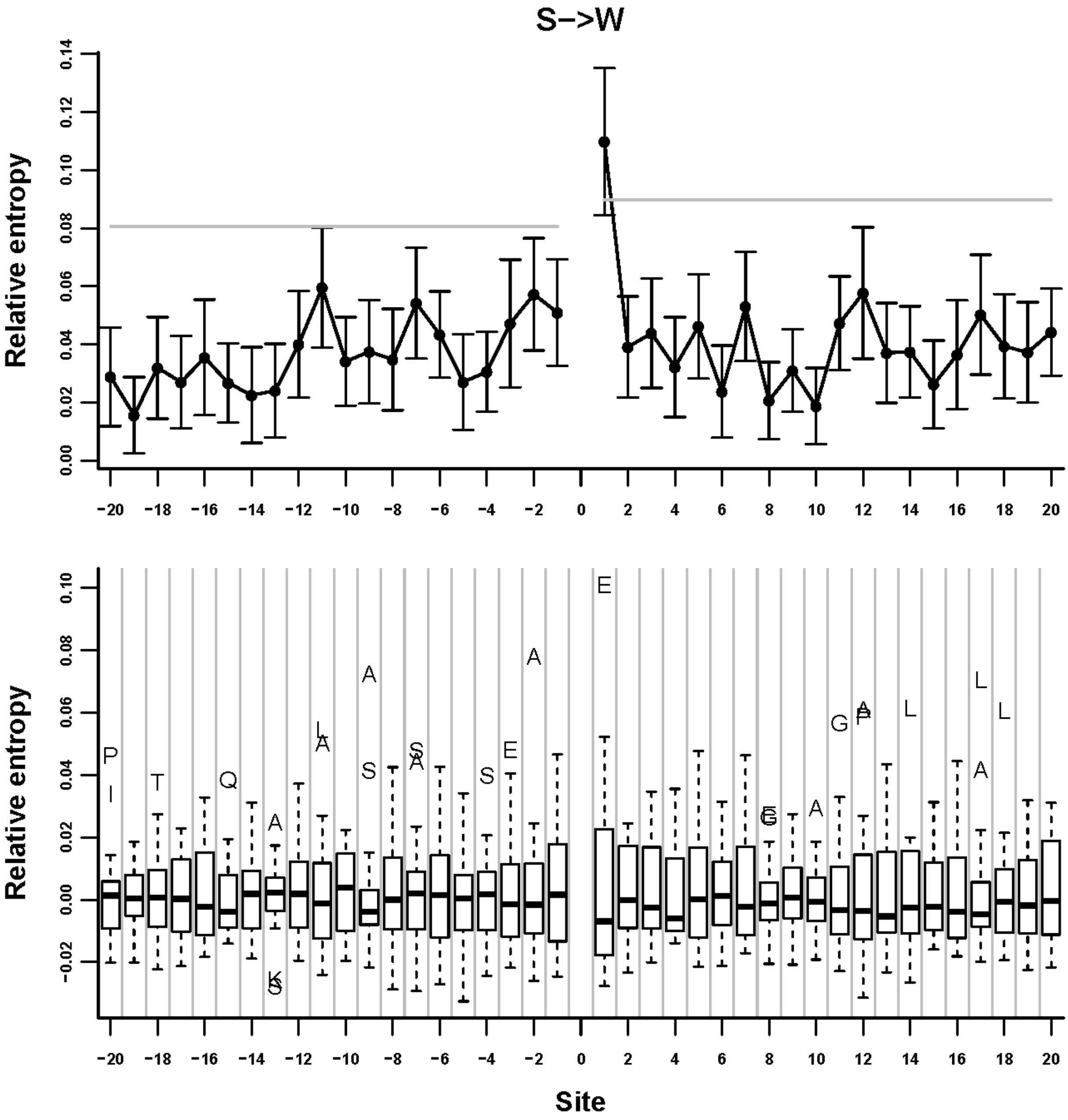
S−>W
Relative entropy
0.00
0.02
0.04
0.06
0.08
0.10
0.12
0.14
−20
−18
−16
−14
−12
−10
−8
−6
−4
−2
0
2
4
6
8
10
12
14
16
18
20
Relative entropy
−0.02
0.00
0.02
0.04
0.06
0.08
0.10
Site

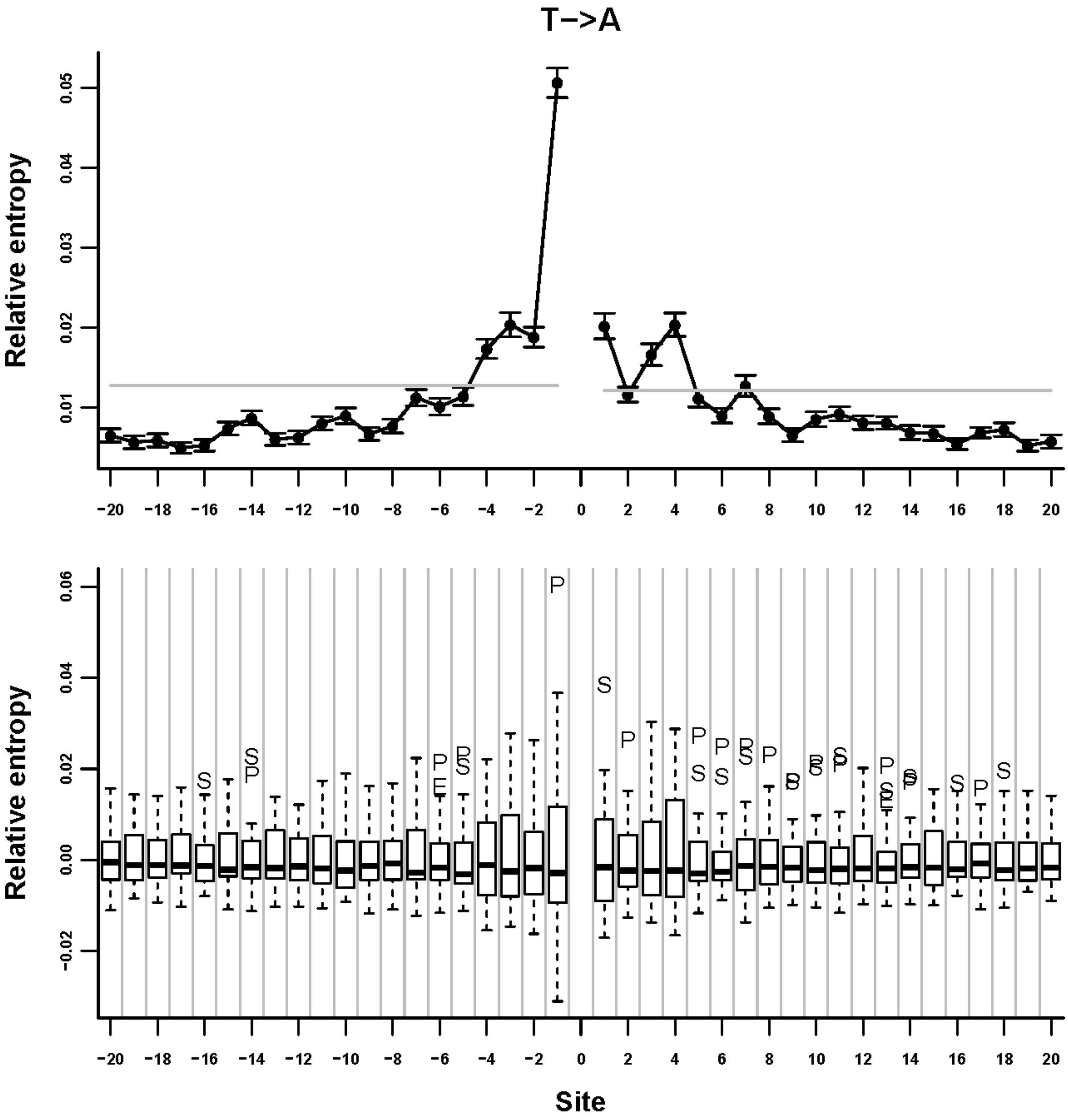
T−>A
Relative entropy
Relative entropy
Site

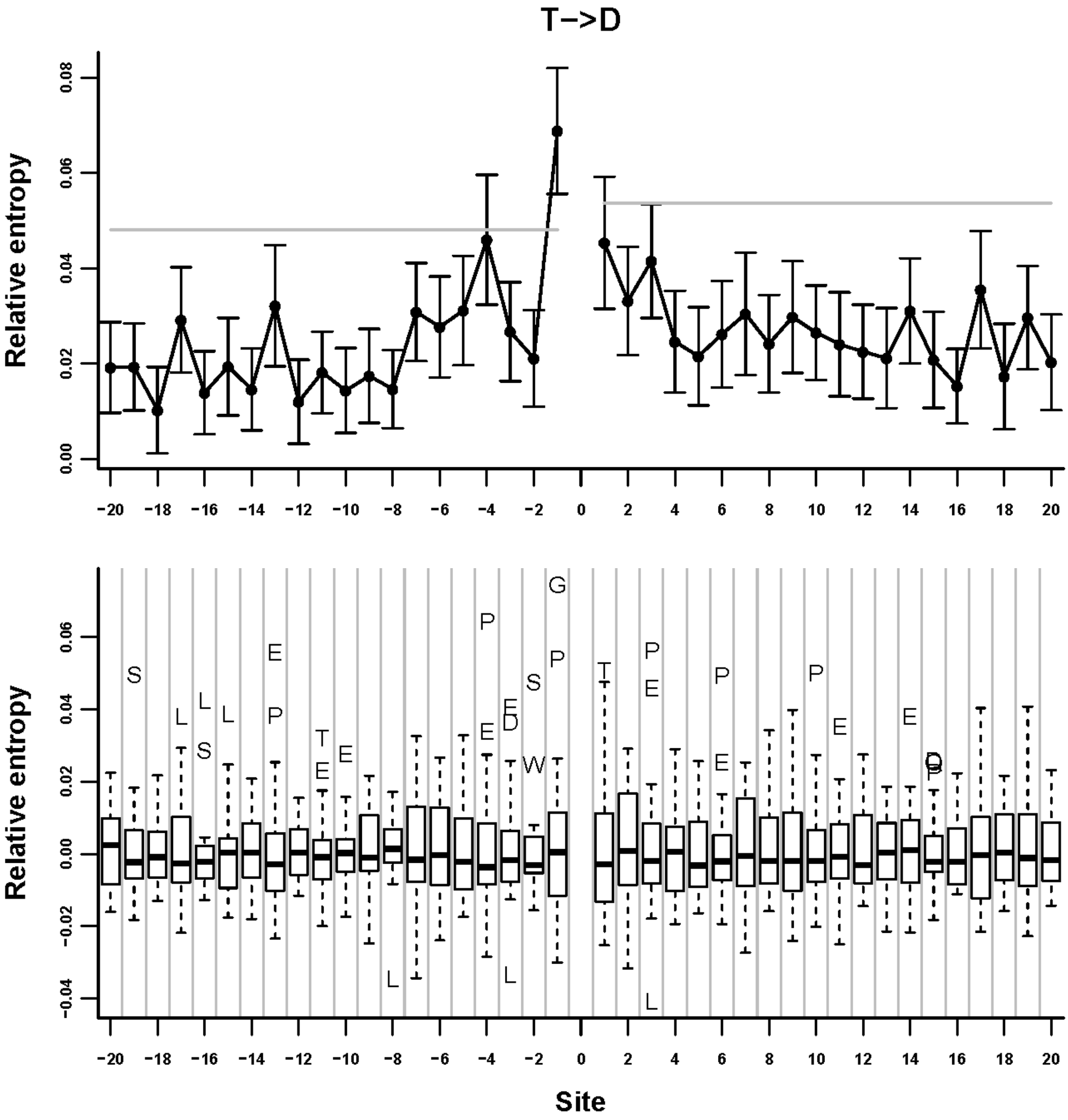
T−>D
Relative entropy
0.00
0.02
0.04
0.06
0.08
−20
−18
−16
−14
−12
−10
−8
−6
−4
−2
0
2
4
6
8
10
12
14
16
18
20
Relative entropy
Site

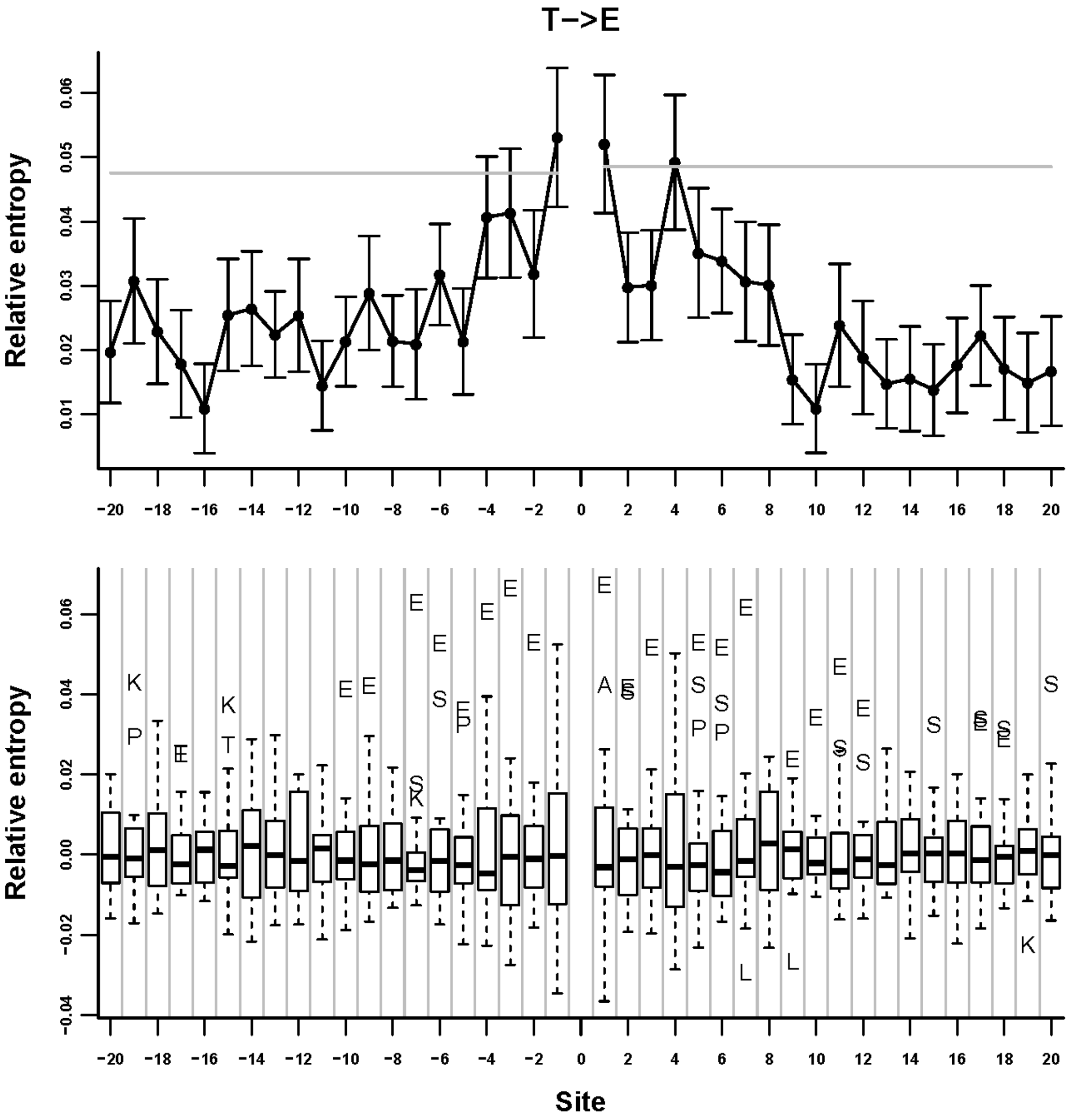
T->E
Relative entropy
Site

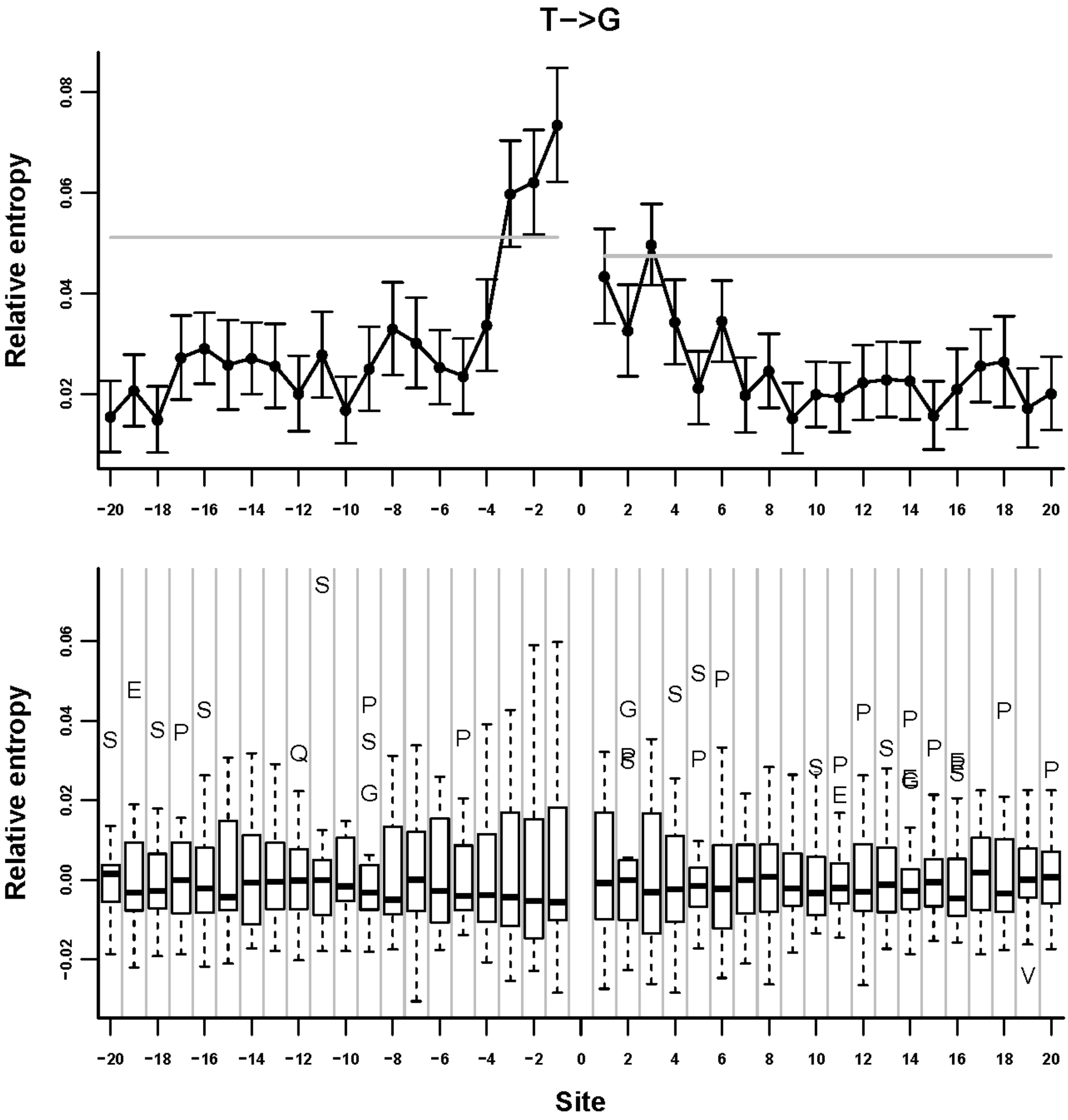
T−>G
Relative entropy
Relative entropy
Site

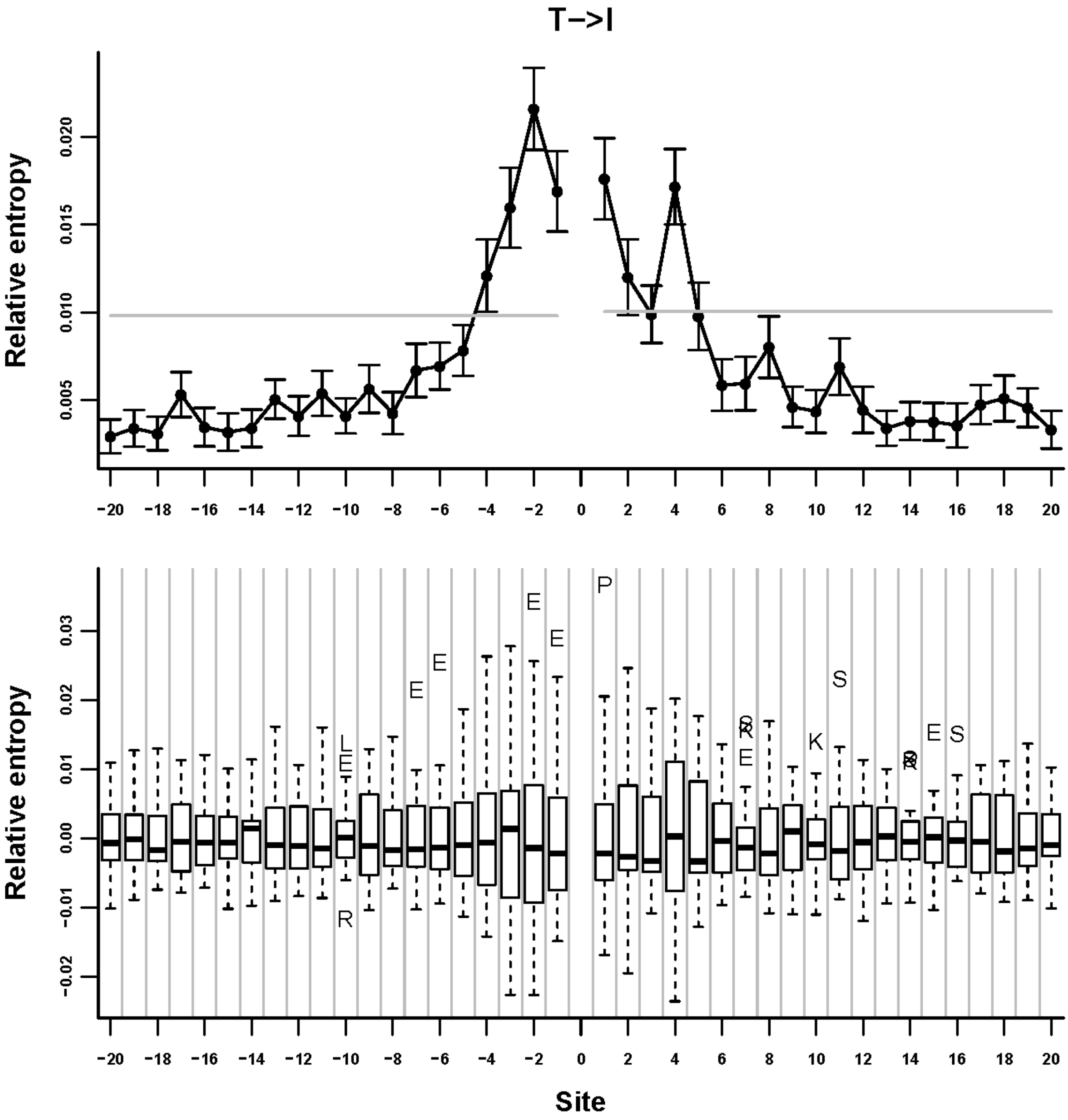
T−>I
Relative entropy
0.005
0.010
0.015
0.020
−20
−18
−16
−14
−12
−10
−8
−6
−4
−2
0
2
4
6
8
10
12
14
16
18
20
Relative entropy
−0.02
−0.01
0.00
0.01
0.02
0.03
P
E
E
E
E
L
E
R
S
K
E
S
Site

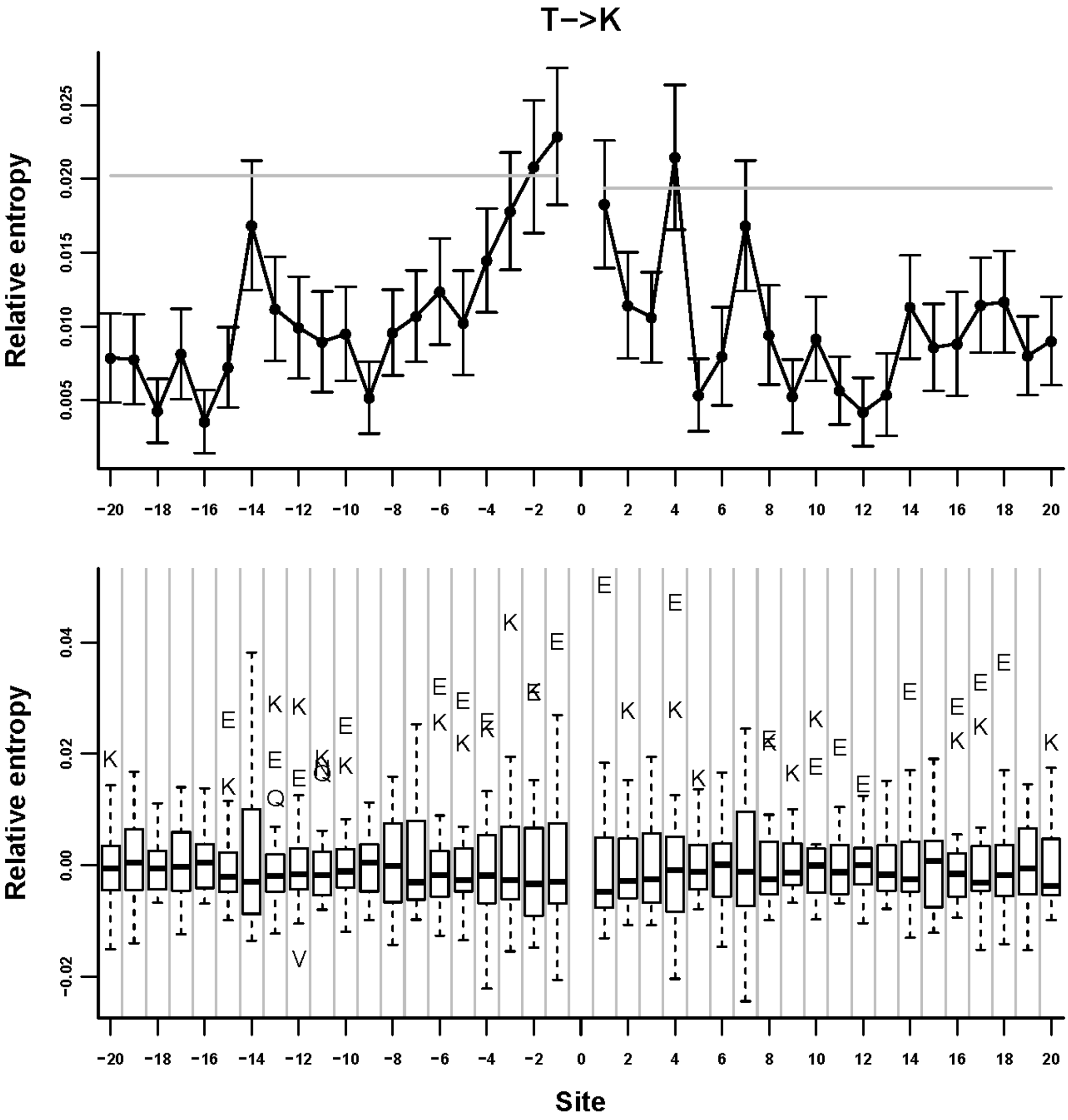
T−>K
Relative entropy
Relative entropy
Site

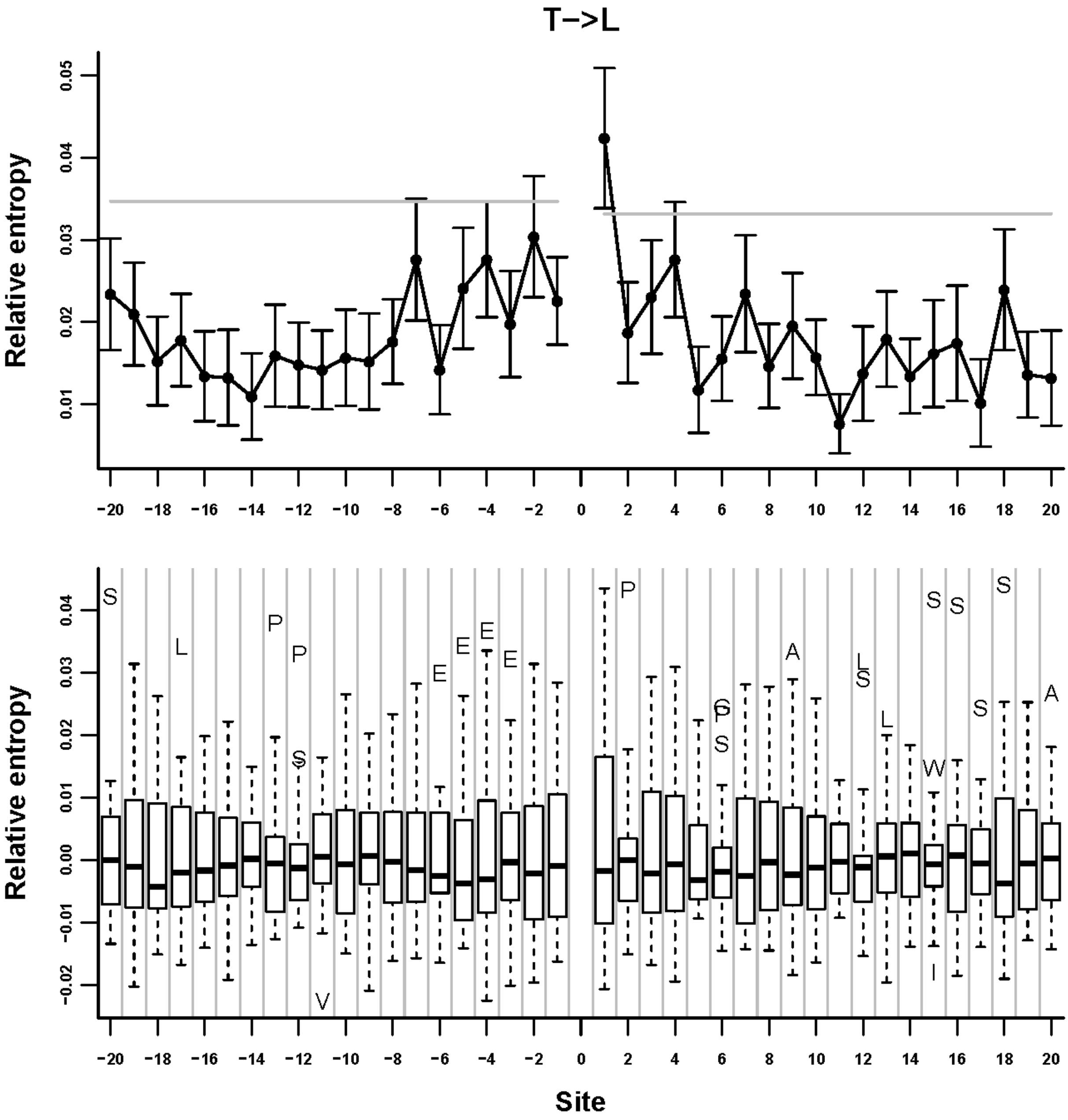
T−>L
Relative entropy
Relative entropy
Site

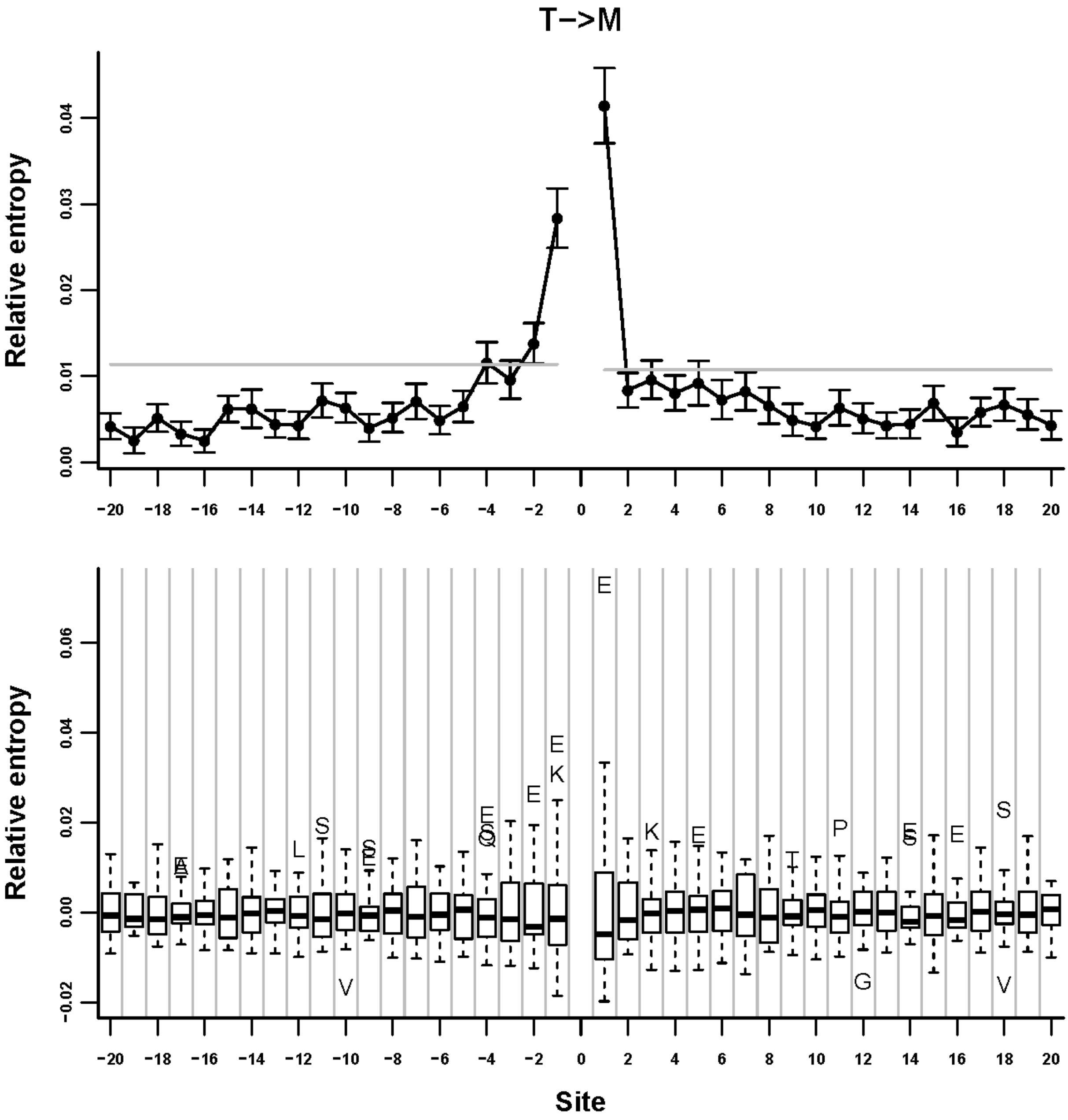
T->M
Relative entropy
0.00
0.01
0.02
0.03
0.04
-20
-18
-16
-14
-12
-10
-8
-6
-4
-2
0
2
4
6
8
10
12
14
16
18
20
Relative entropy
-0.02
0.00
0.02
0.04
0.06
Site

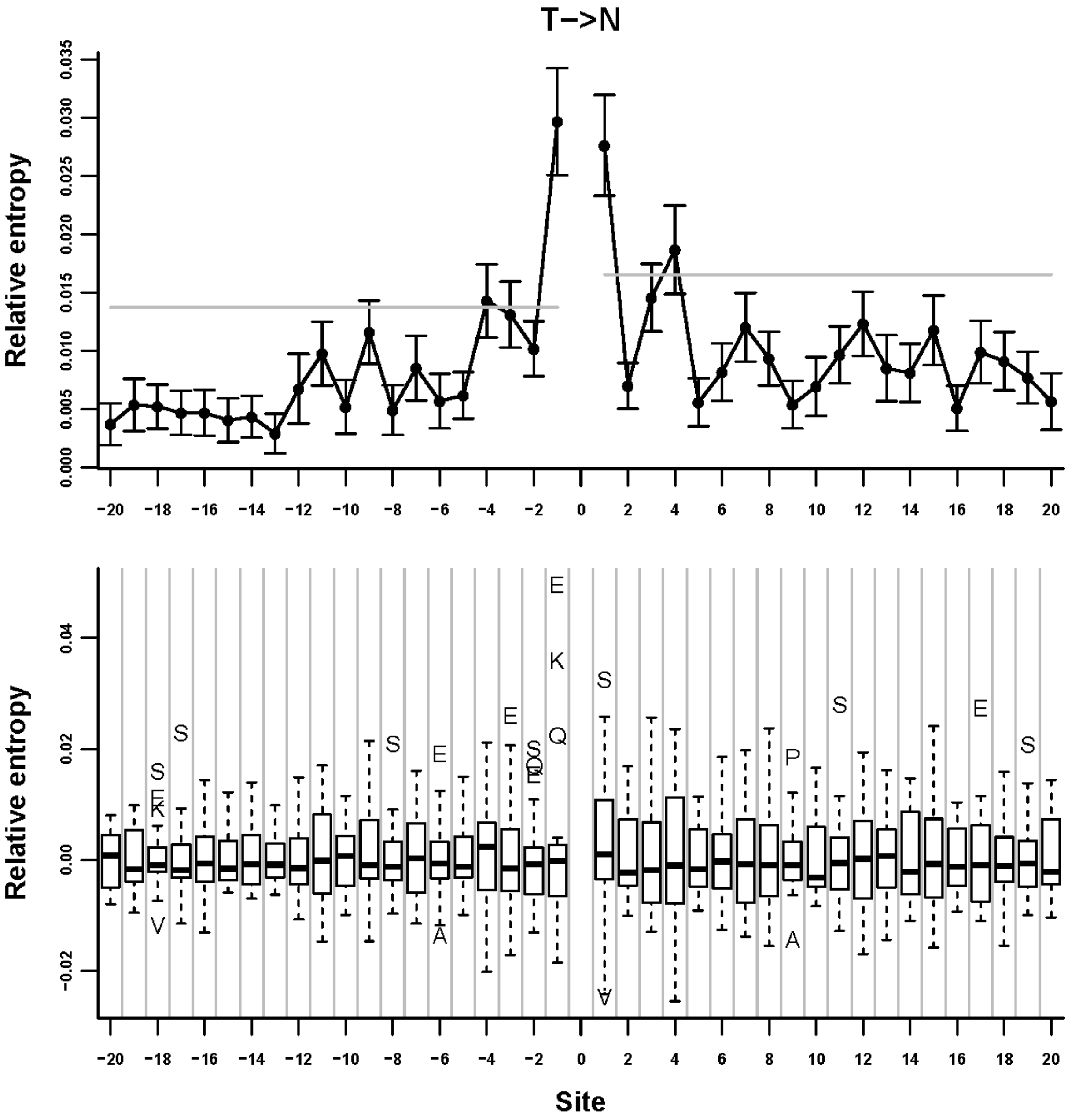
T−>N
Relative entropy
0.000
0.005
0.010
0.015
0.020
0.025
0.030
0.035
−20 −18 −16 −14 −12 −10 −8 −6 −4 −2 0 2 4 6 8 10 12 14 16 18 20
Relative entropy
−0.02
0.00
0.02
0.04
S
E
K
V
S
S
E
A
E
S
Q
E
E
K
Q
S
A
P
A
S
E
S
Site

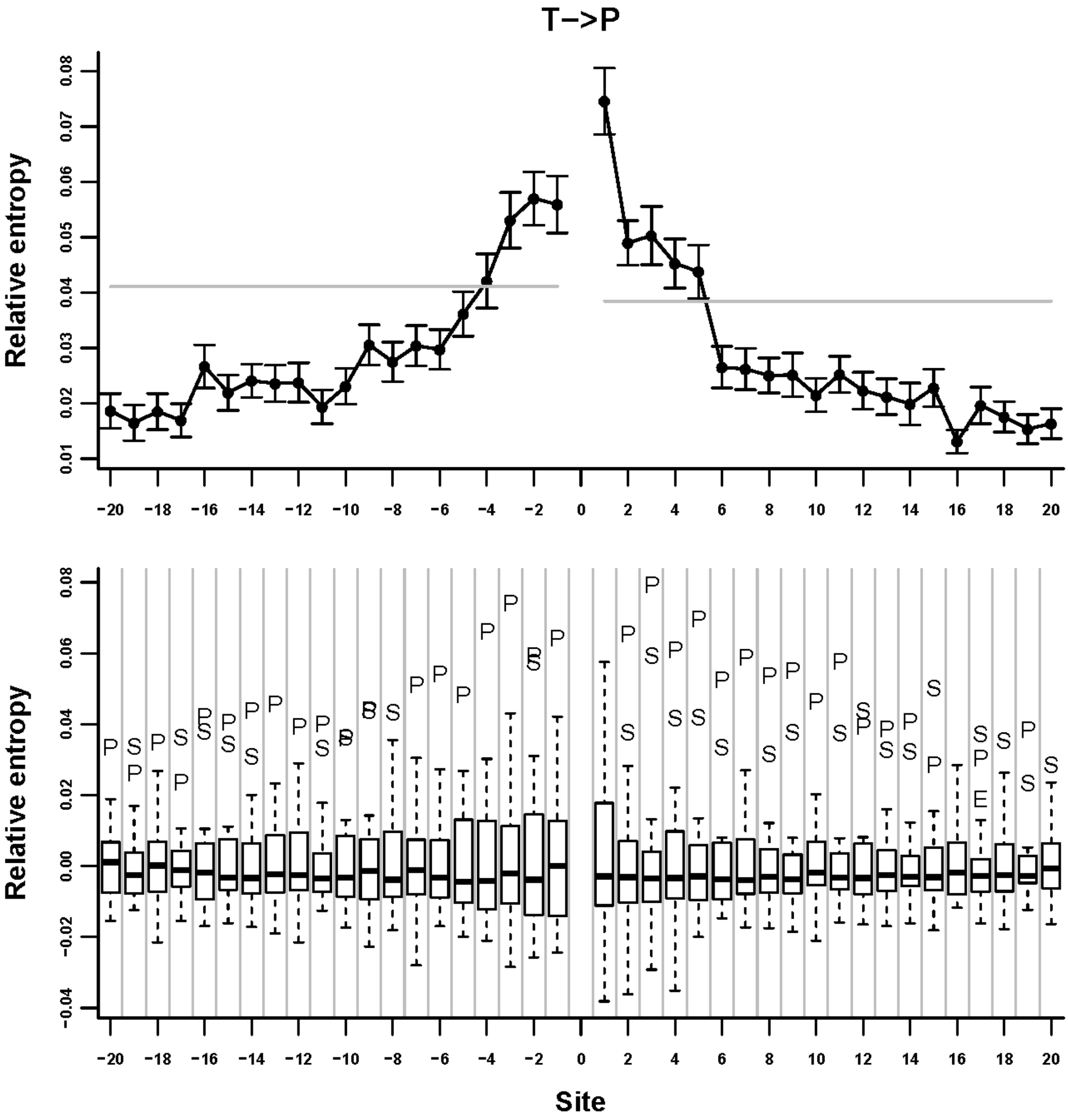
T−>P
Relative entropy
Relative entropy
Site

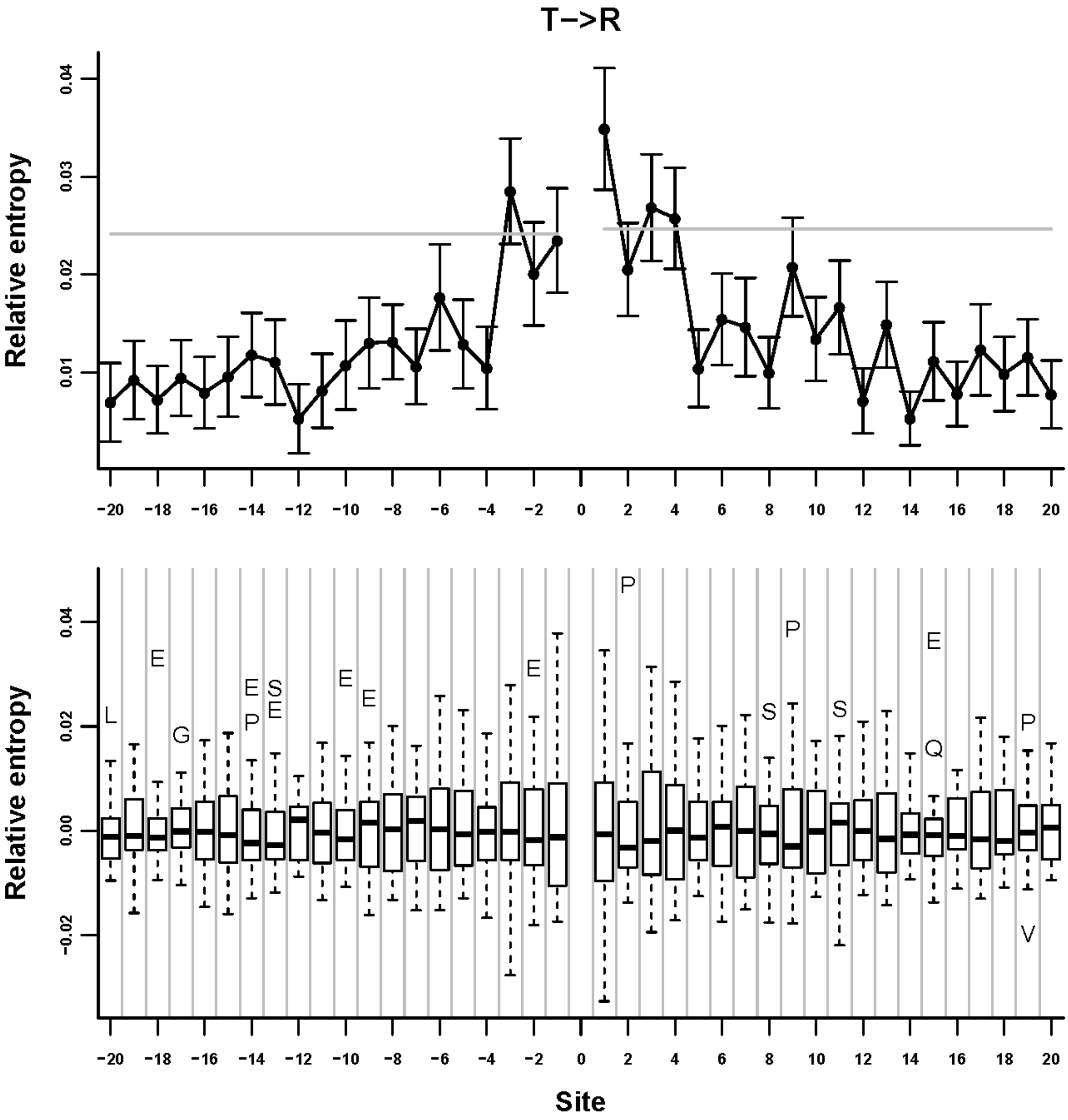
T−>R
Relative entropy
0.01
0.02
0.03
0.04
−20 −18 −16 −14 −12 −10 −8 −6 −4 −2 0 2 4 6 8 10 12 14 16 18 20
Relative entropy
−0.02
0.00
0.02
0.04
L E G E P S E E E E P P S S E Q P V
−20 −18 −16 −14 −12 −10 −8 −6 −4 −2 0 2 4 6 8 10 12 14 16 18 20
Site

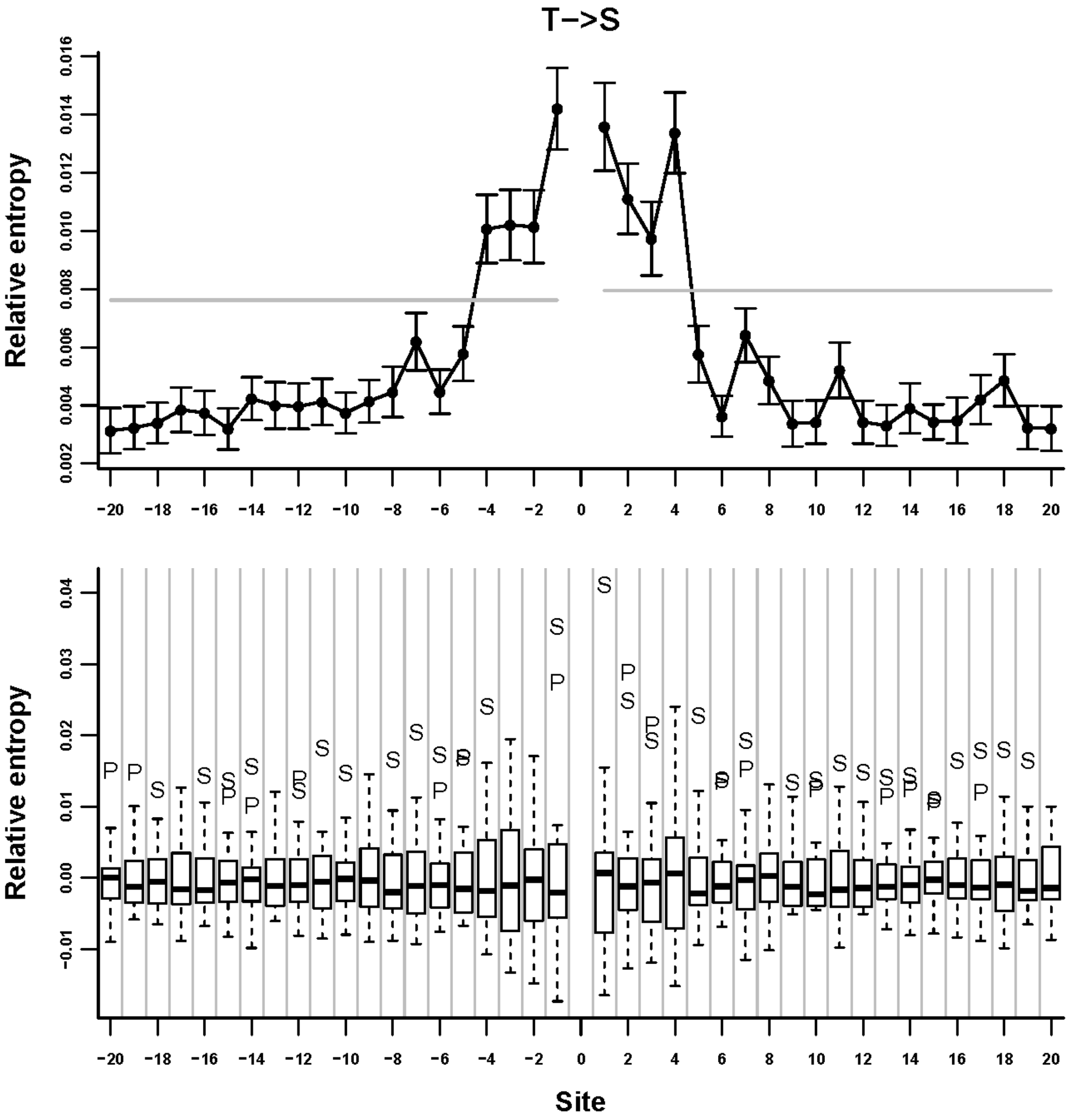
T−>S
Relative entropy
Relative entropy
Site

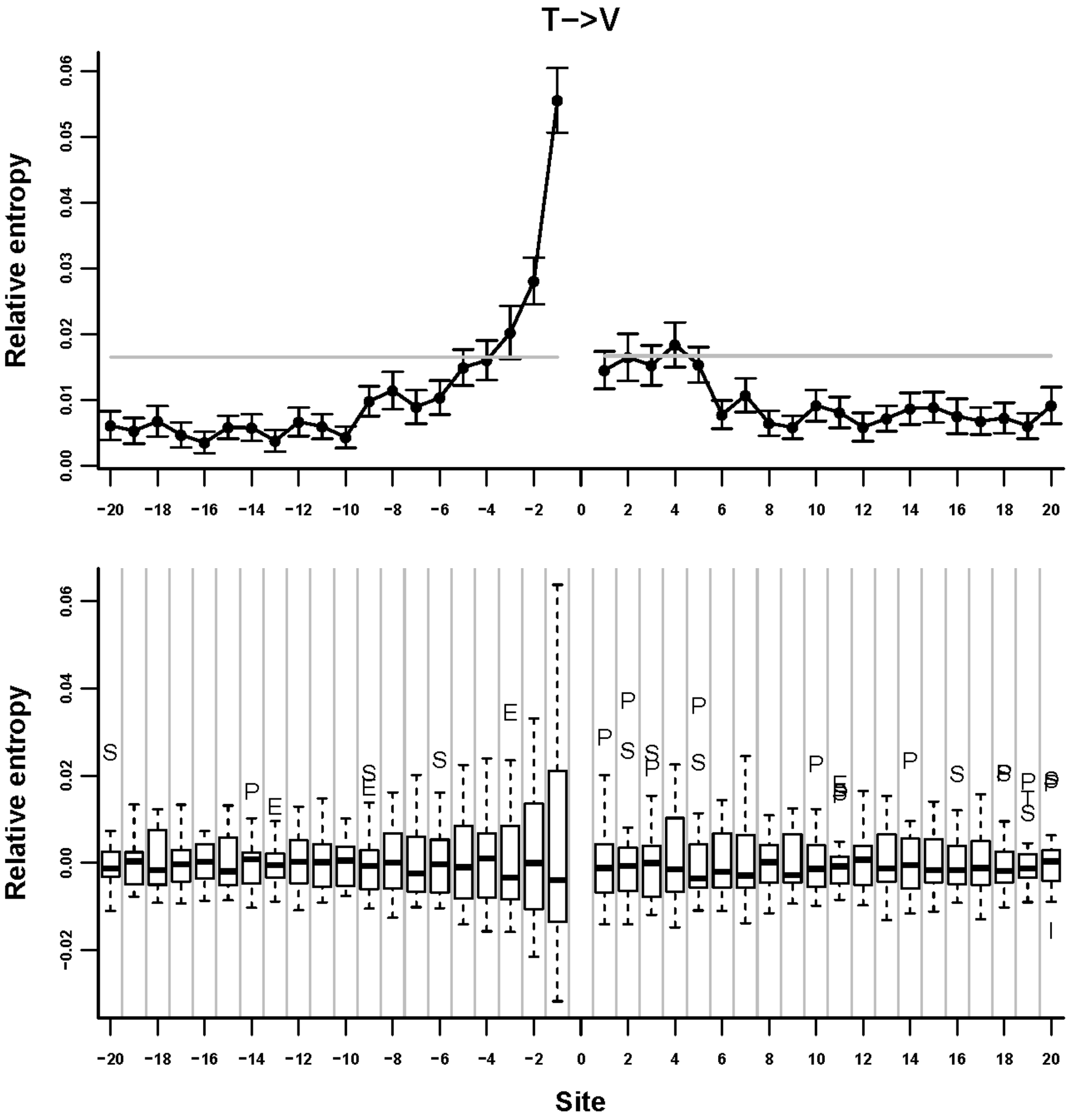
T−>V
Relative entropy
Relative entropy
Site

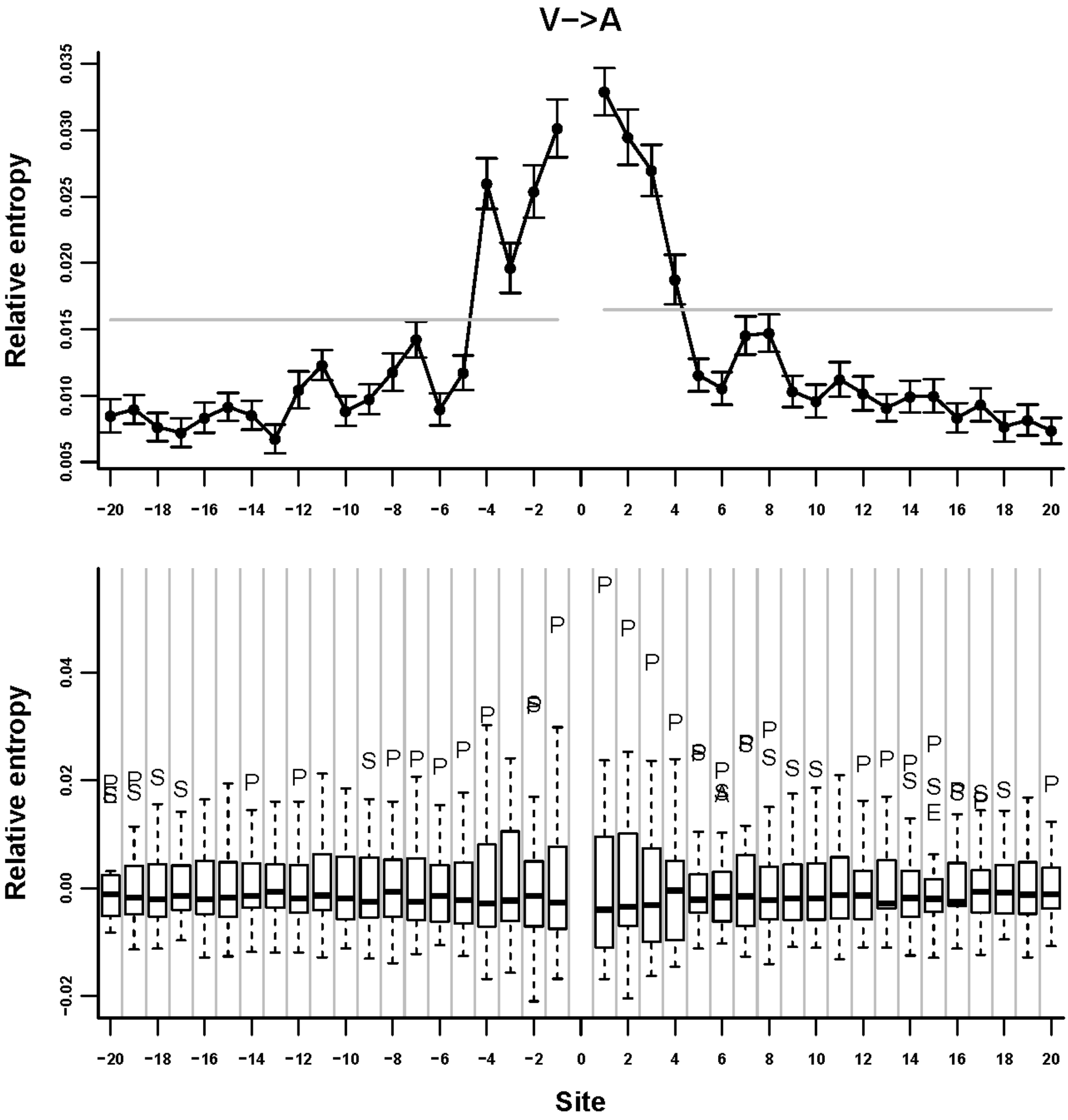
V->A
Relative entropy
Relative entropy
Site

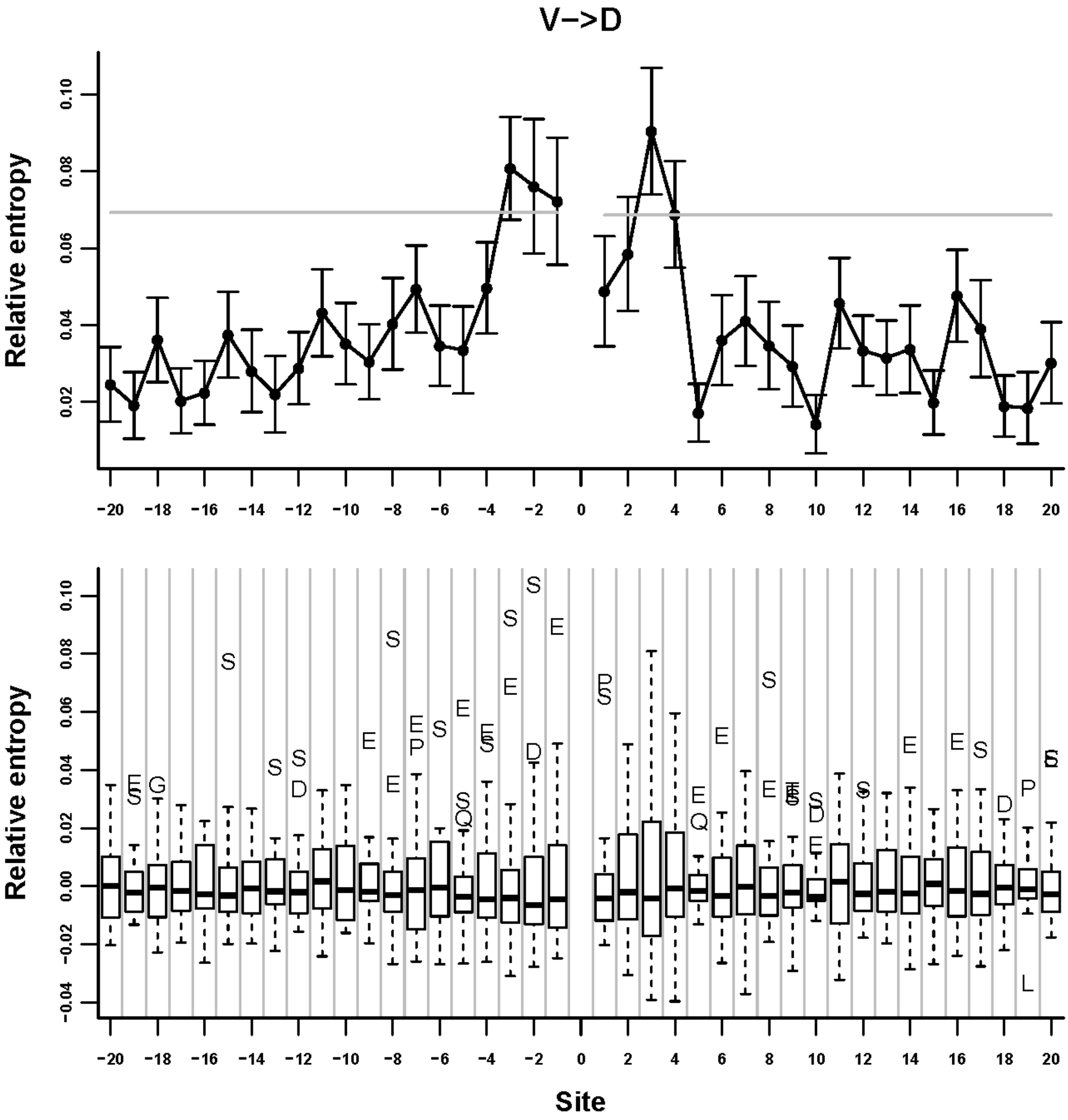
V−>D
Relative entropy
0.02
0.04
0.06
0.08
0.10
−20
−18
−16
−14
−12
−10
−8
−6
−4
−2
0
2
4
6
8
10
12
14
16
18
20
Relative entropy
−0.04
−0.02
0.00
0.02
0.04
0.06
0.08
0.10
Site

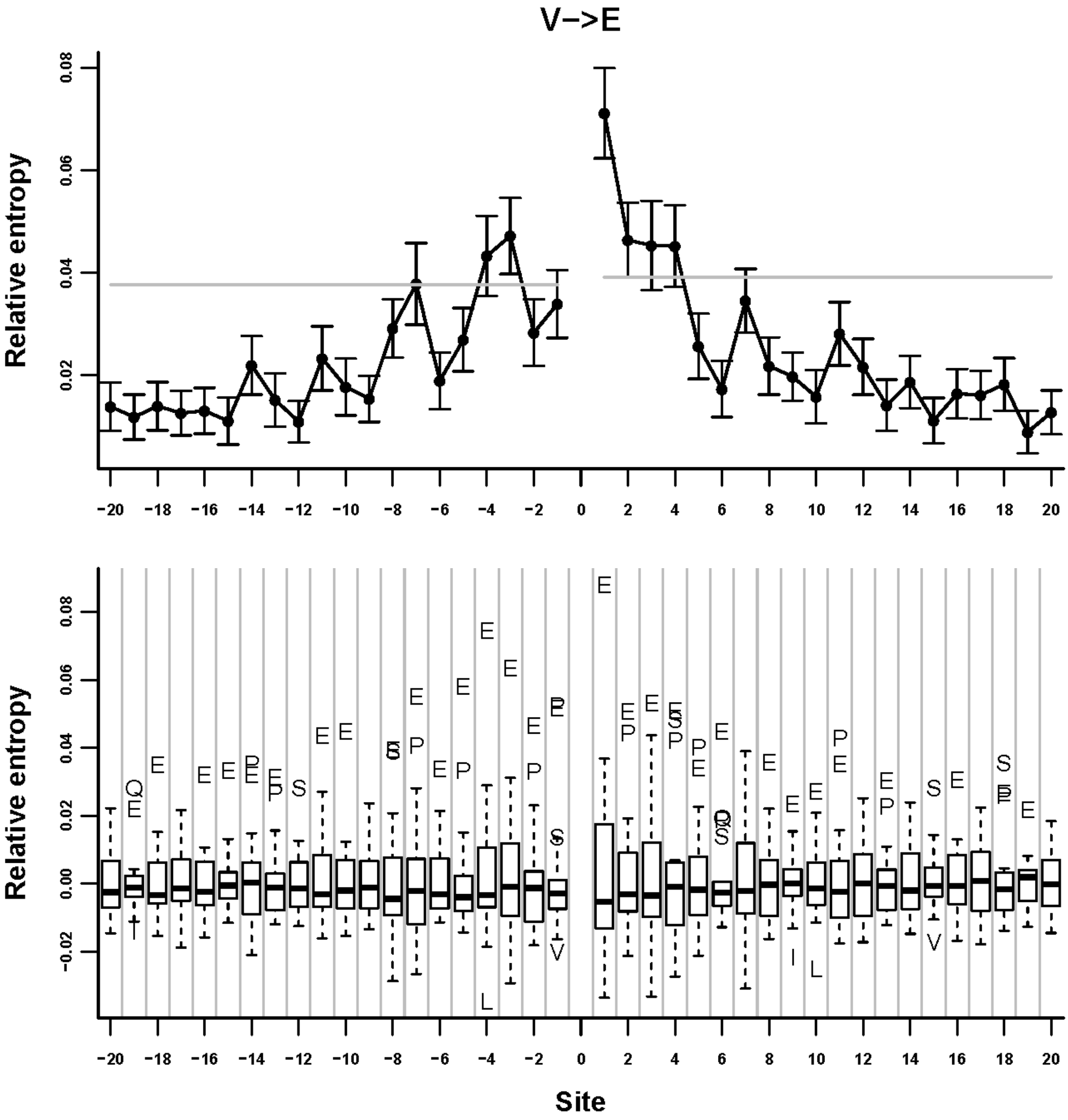
V−>E
Relative entropy
Relative entropy
Site

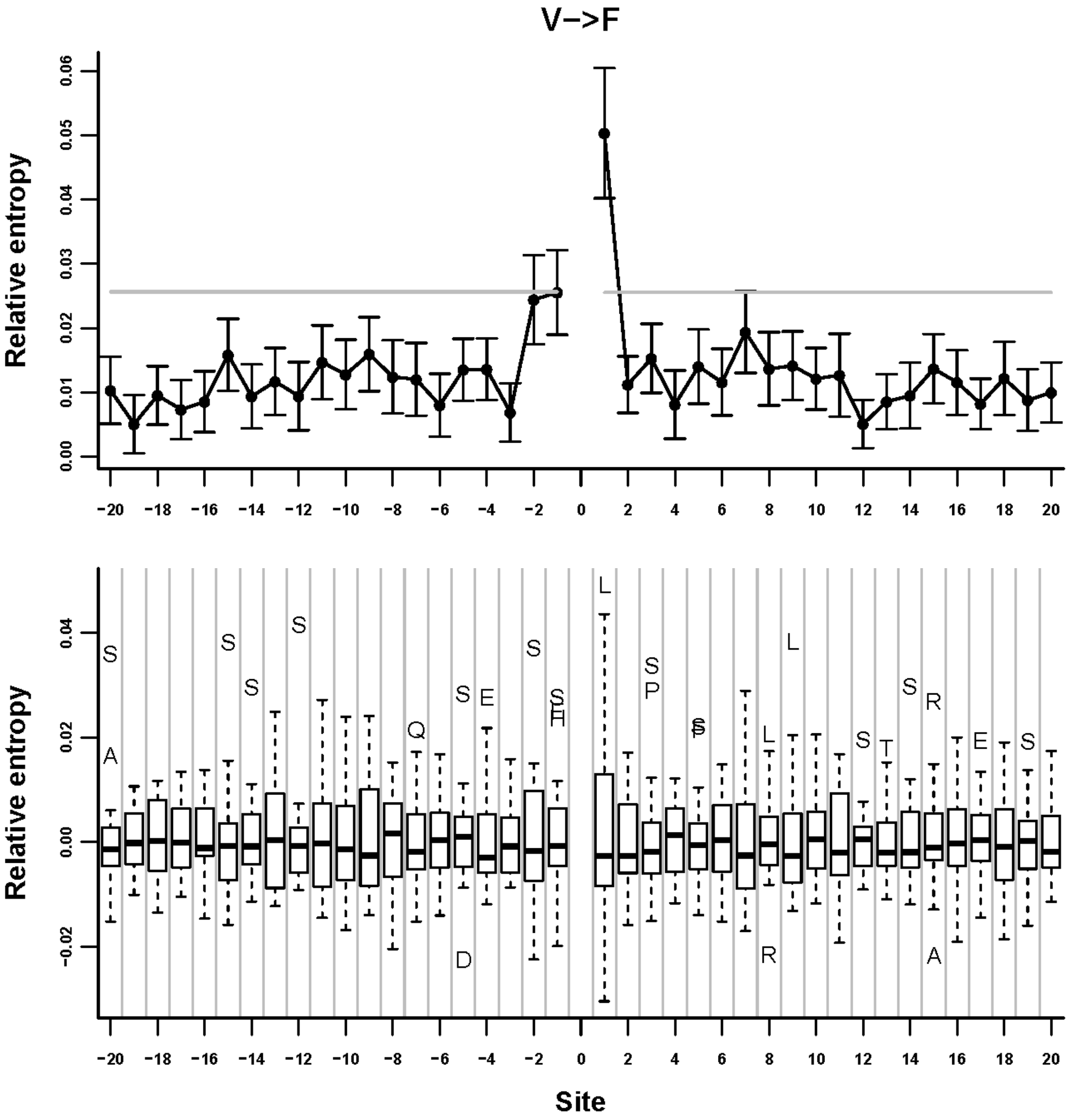
V->F
Relative entropy
0.00
0.01
0.02
0.03
0.04
0.05
0.06
−20 −18 −16 −14 −12 −10 −8 −6 −4 −2 0 2 4 6 8 10 12 14 16 18 20
Relative entropy
−0.02
0.00
0.02
0.04
Site

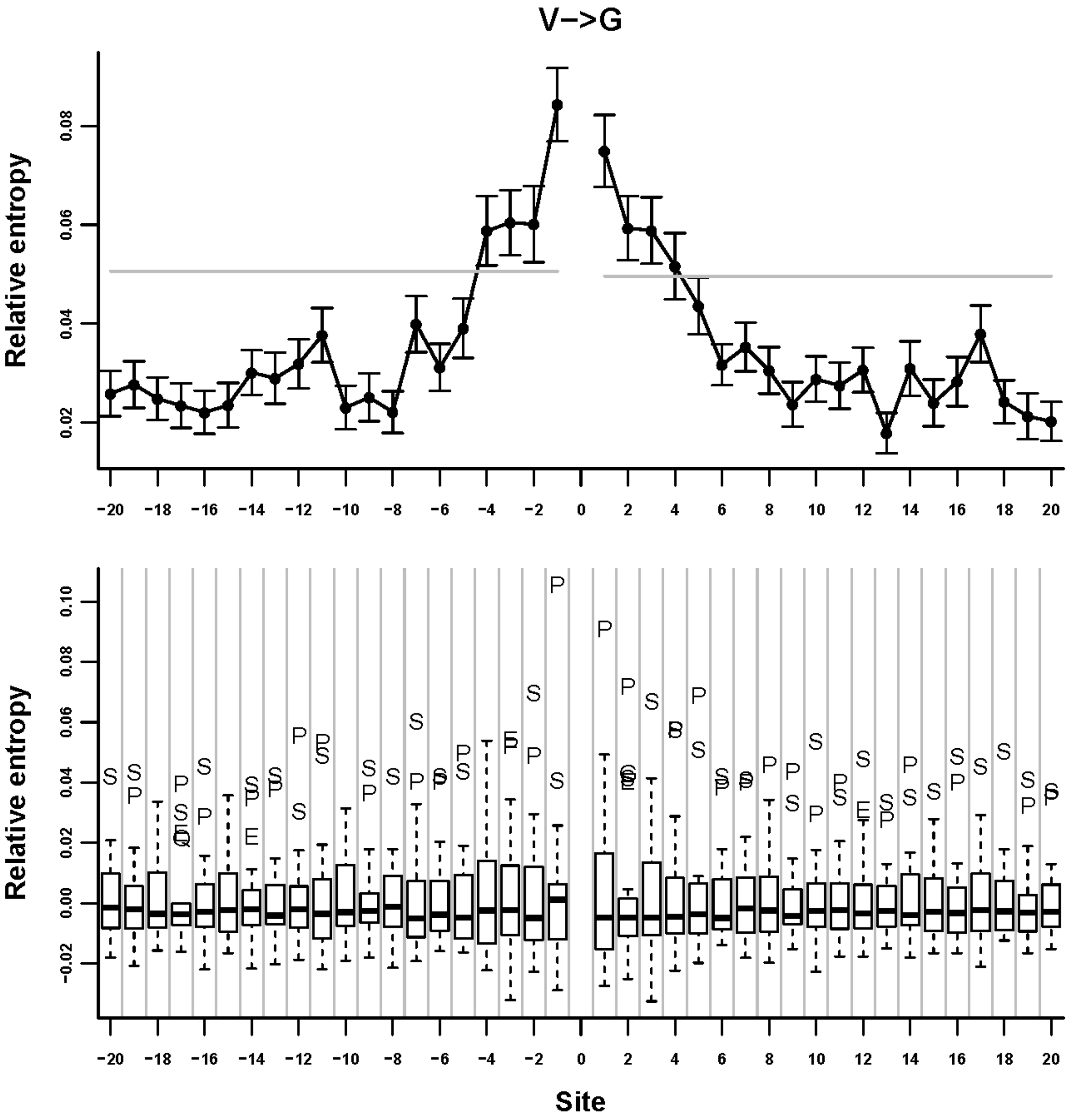
V−>G
Relative entropy
0.02
0.04
0.06
0.08
−20 −18 −16 −14 −12 −10 −8 −6 −4 −2 0 2 4 6 8 10 12 14 16 18 20
Relative entropy
−0.02
0.00
0.02
0.04
0.06
0.08
0.10
−20 −18 −16 −14 −12 −10 −8 −6 −4 −2 0 2 4 6 8 10 12 14 16 18 20
Site

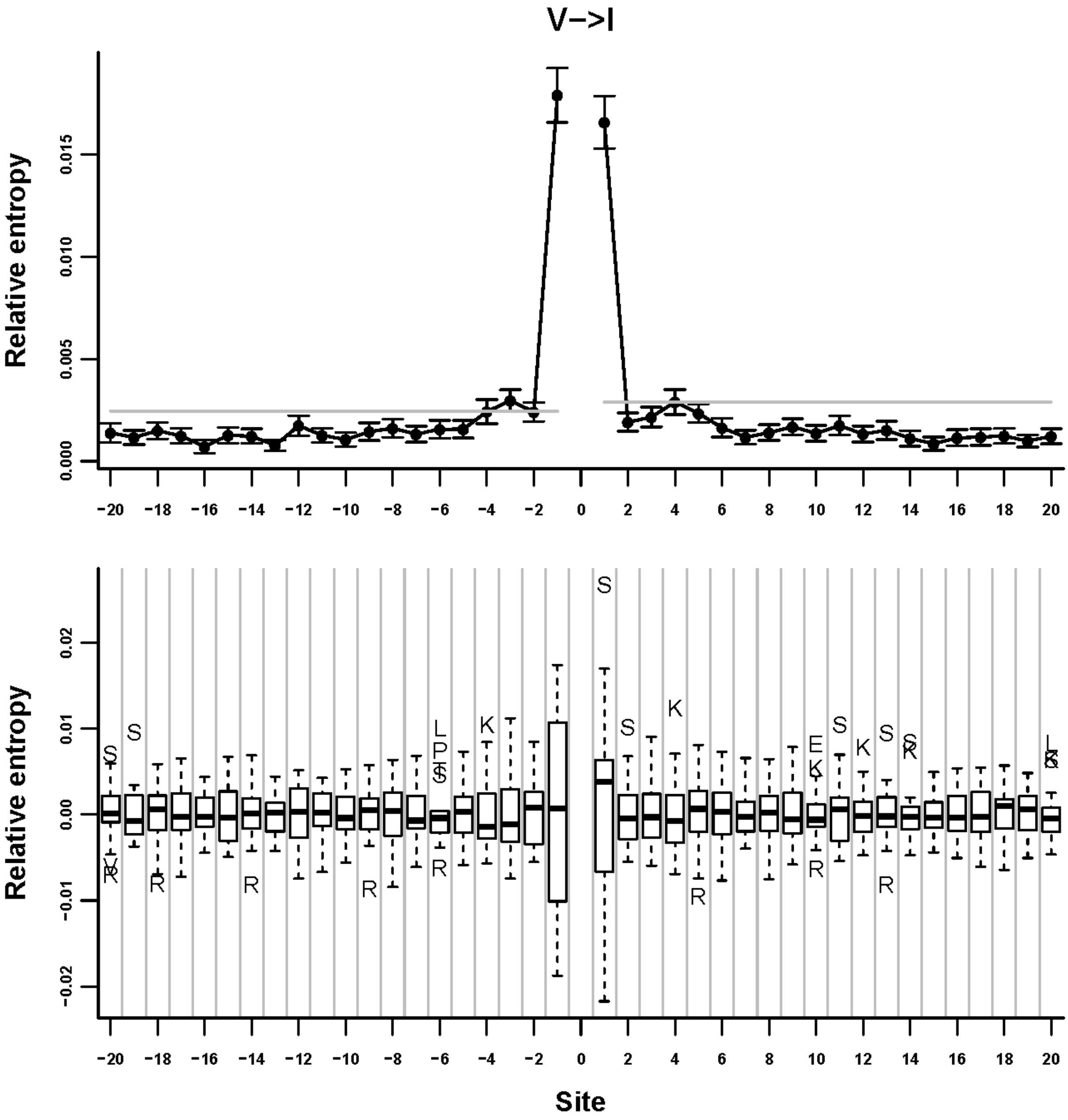
V−>I
Relative entropy
Site

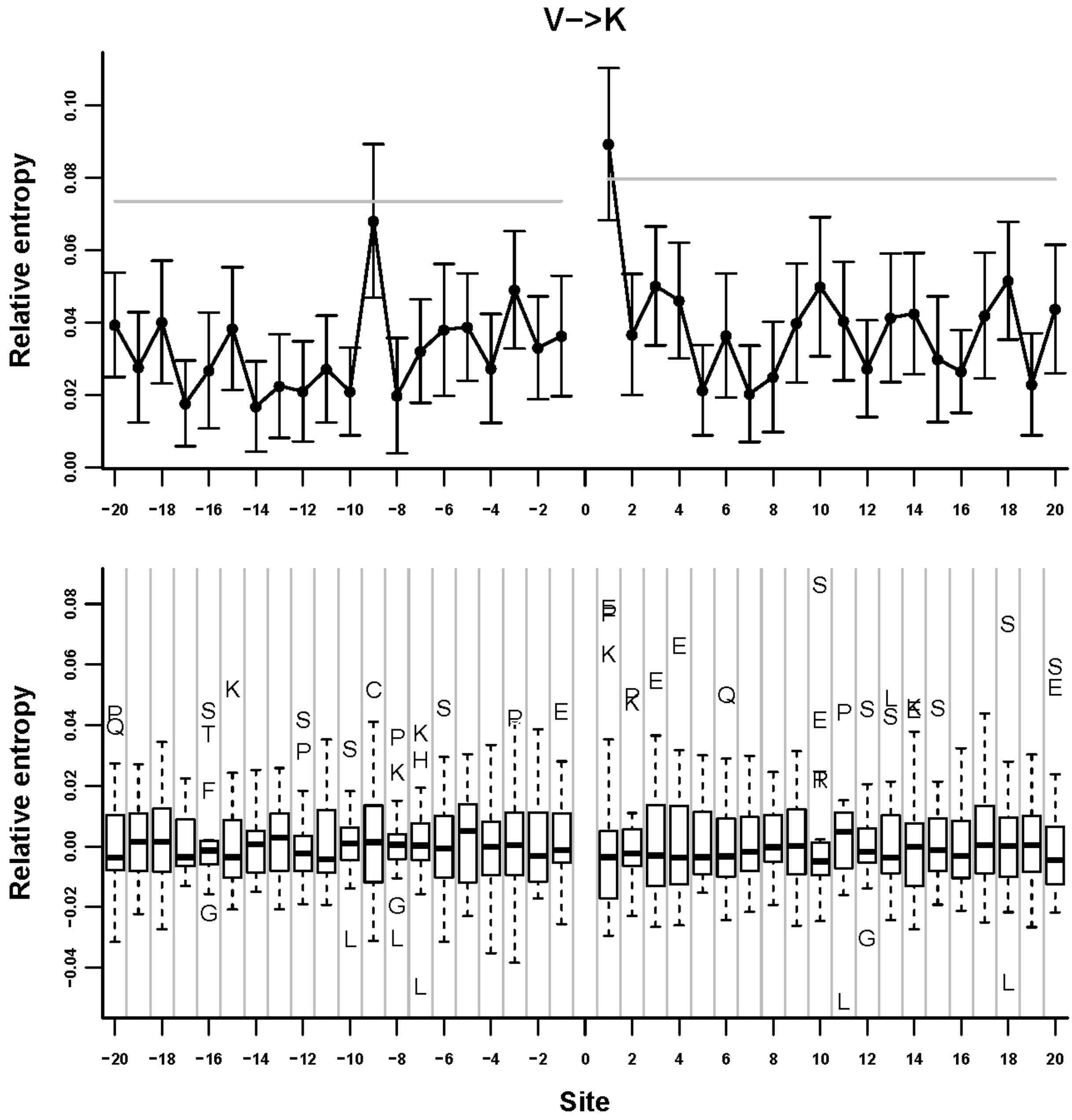
V−>K
Relative entropy
Relative entropy
Site

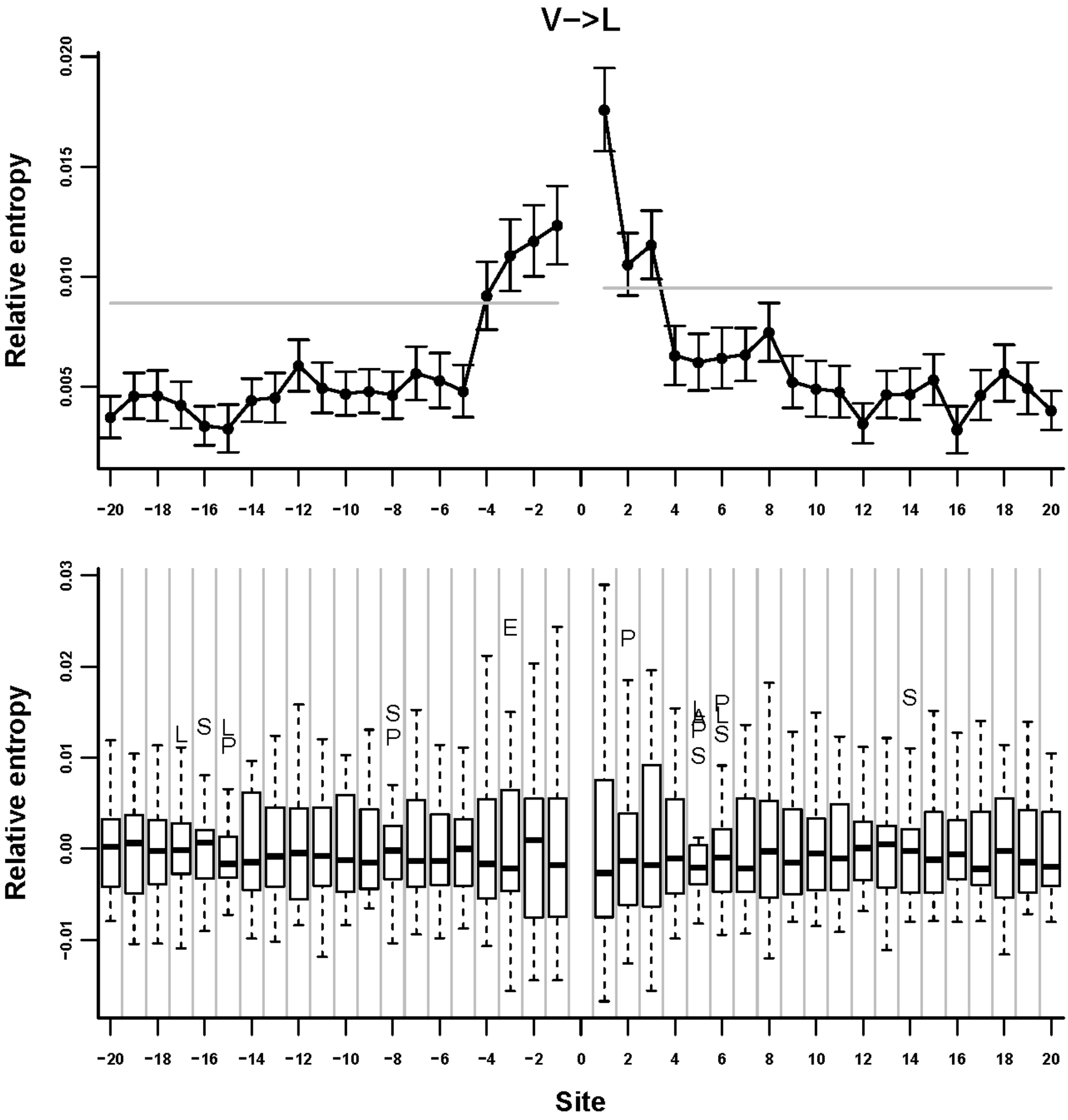
V−>L
Relative entropy
0.005
0.010
0.015
0.020
−20
−18
−16
−14
−12
−10
−8
−6
−4
−2
0
2
4
6
8
10
12
14
16
18
20
Relative entropy
−0.01
0.00
0.01
0.02
0.03
L
S
L
P
S
P
E
P
L
A
P
S
P
L
S
S
Site

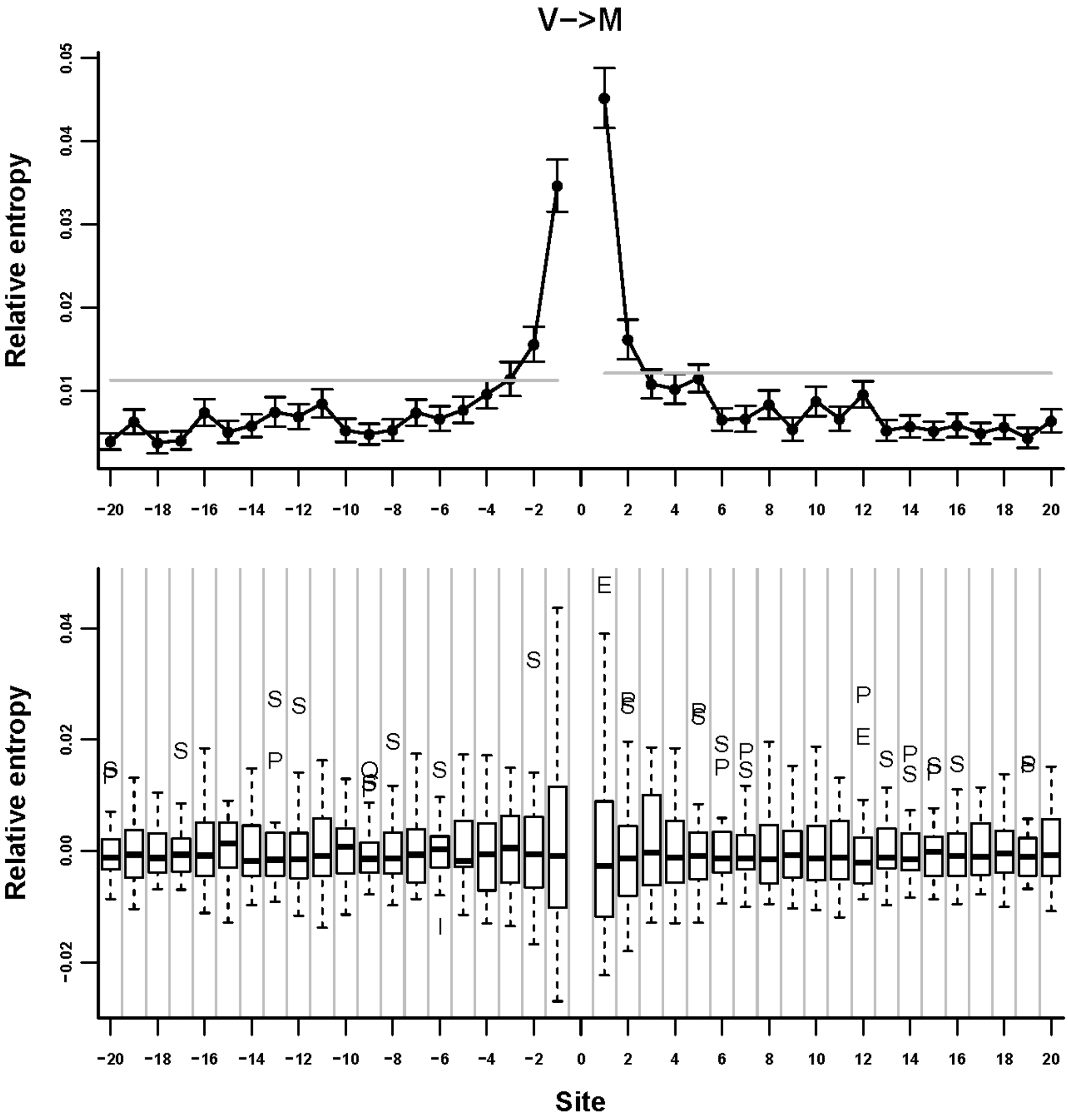

V−>M
Relative entropy
Relative entropy
Site

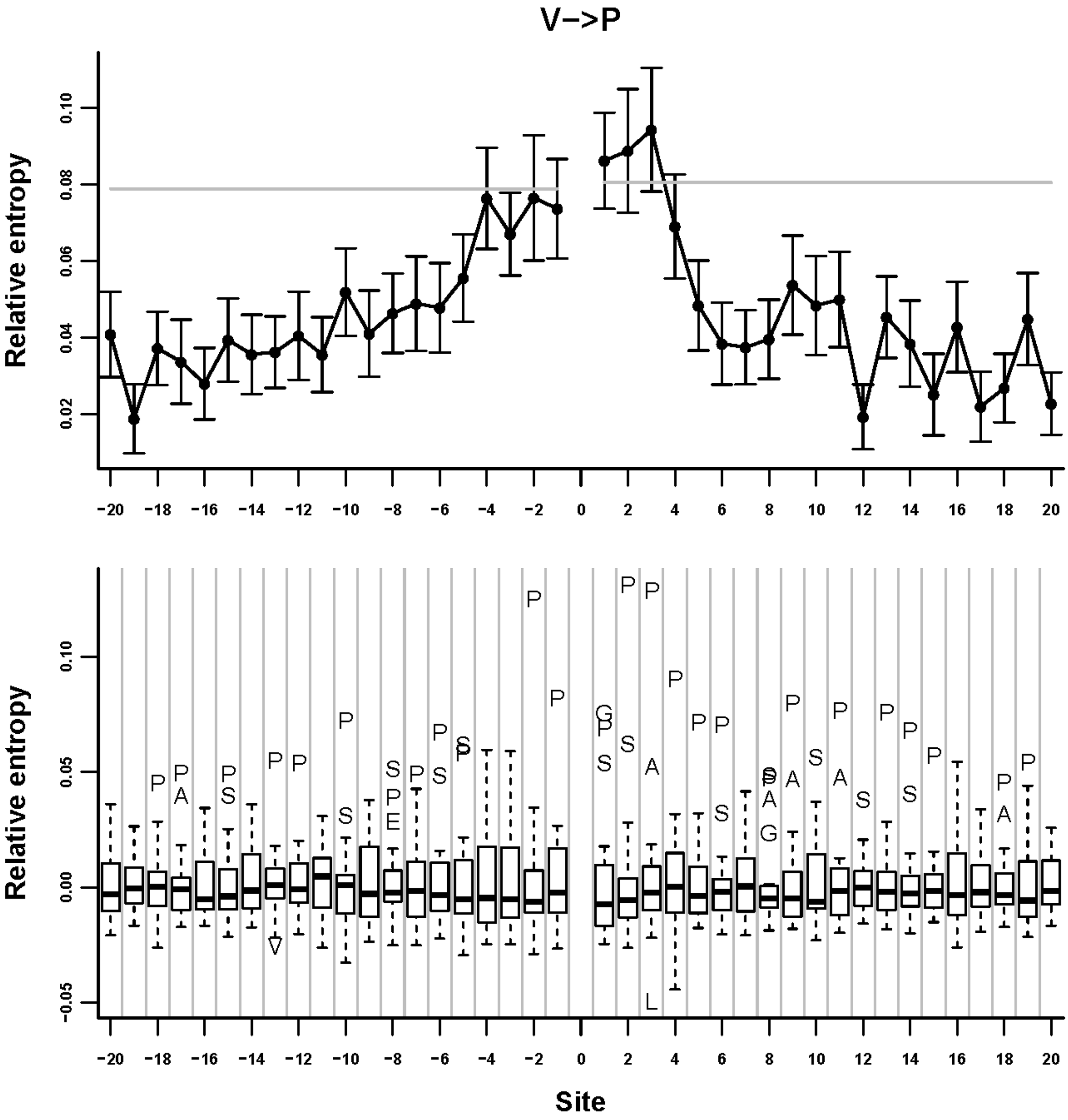
V−>P
Relative entropy
Relative entropy
Site

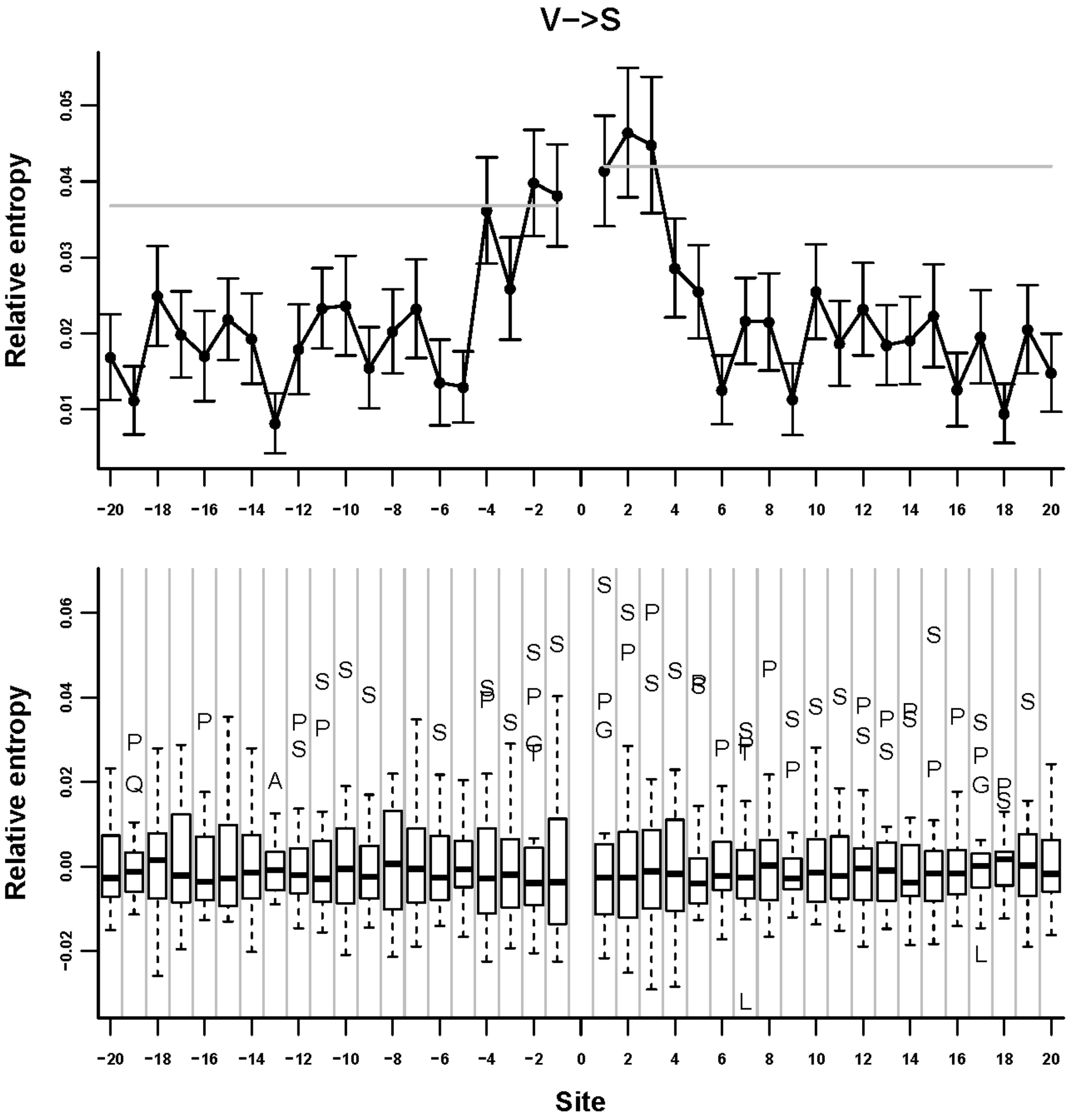
V−>S
Relative entropy
Relative entropy
Site

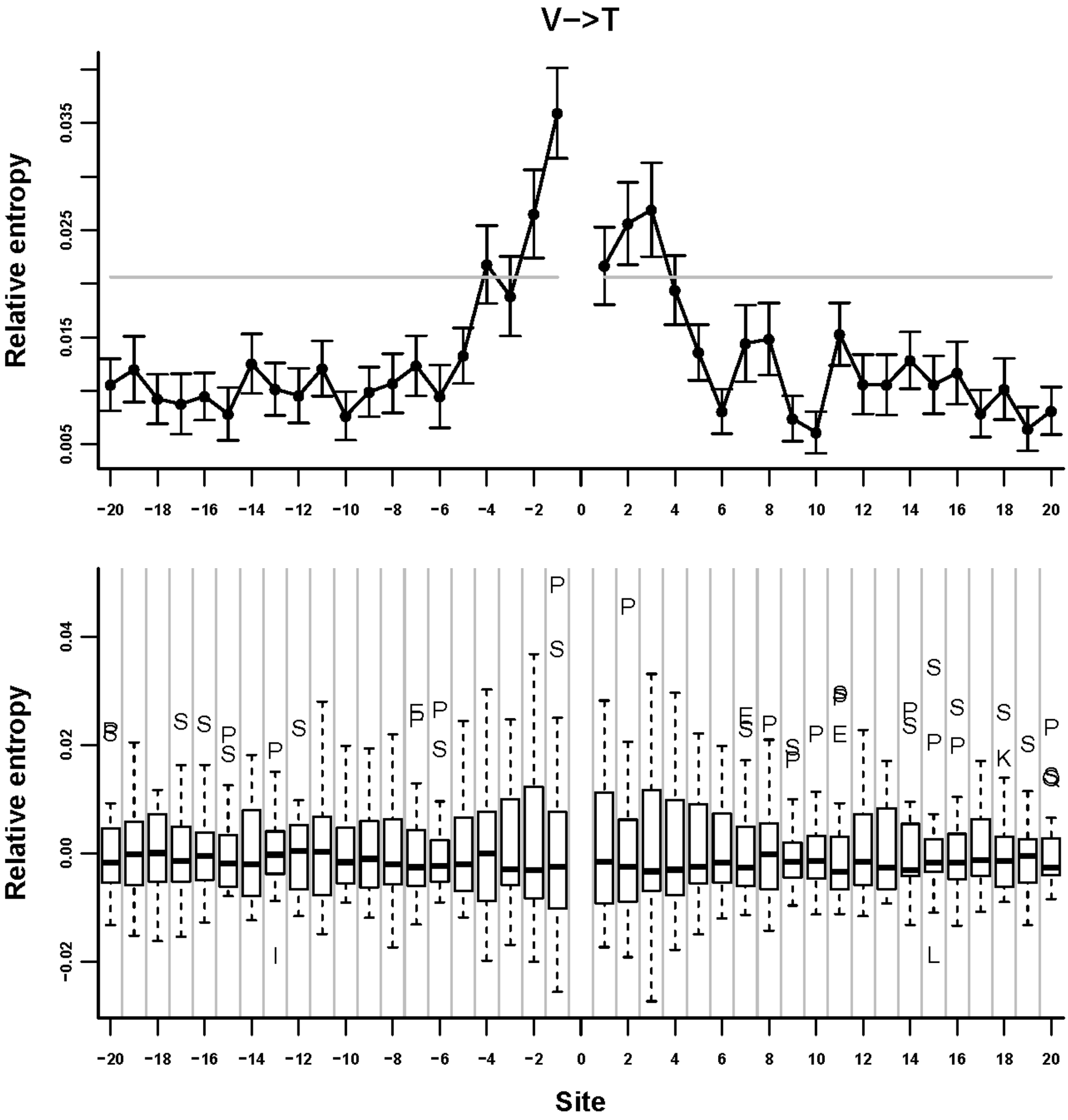
V->T
Relative entropy
Relative entropy
Site

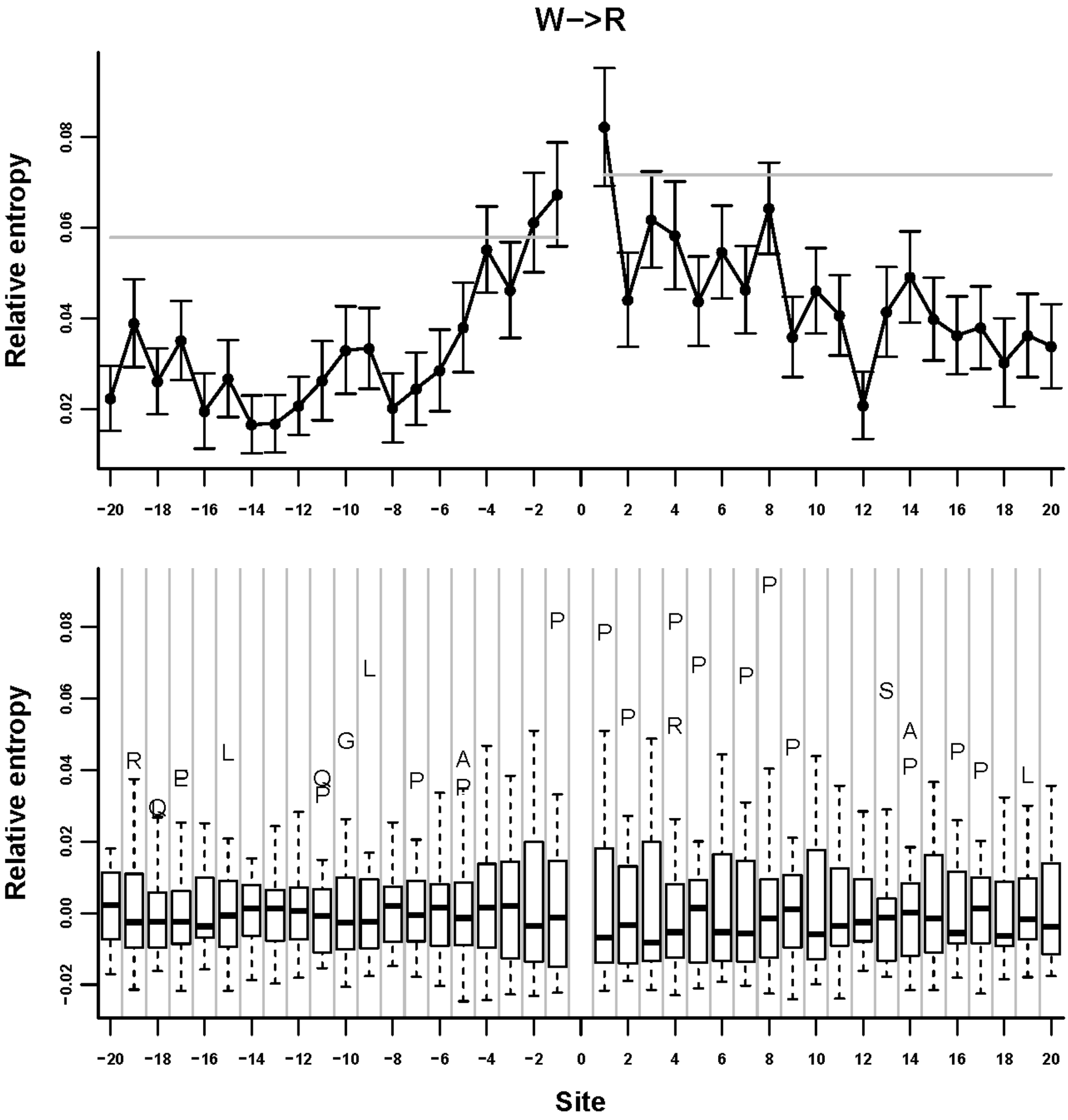
W−>R
Relative entropy
0.02
0.04
0.06
0.08
−20 −18 −16 −14 −12 −10 −8 −6 −4 −2 0 2 4 6 8 10 12 14 16 18 20
Relative entropy
−0.02
0.00
0.02
0.04
0.06
0.08
Site

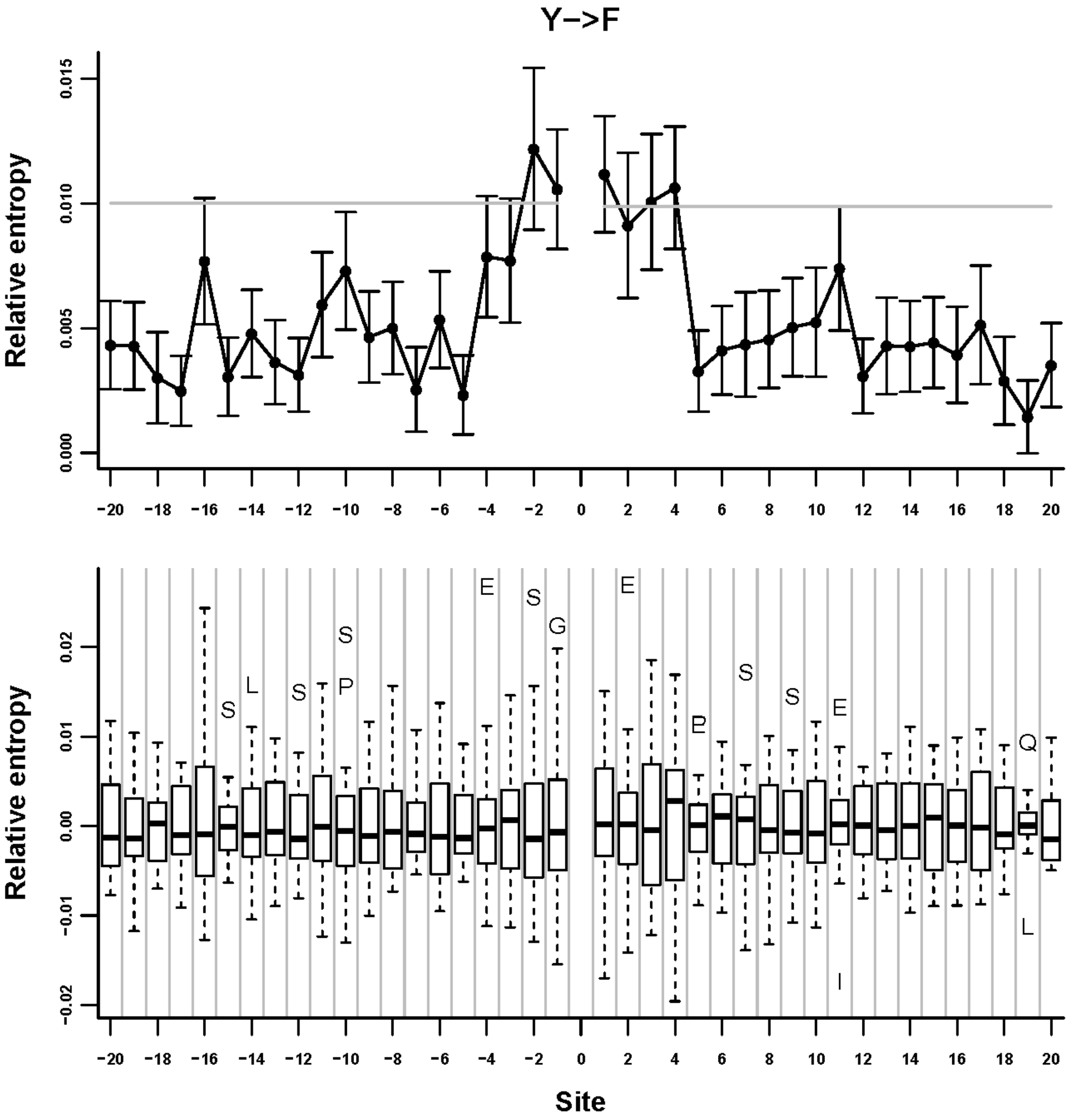
Y->F
Relative entropy
0.015
0.010
0.005
0.000
-20 -18 -16 -14 -12 -10 -8 -6 -4 -2 0 2 4 6 8 10 12 14 16 18 20
Relative entropy
0.02
0.01
0.00
-0.01
-0.02
E
S
G
E
S
S
L
S
P
S
S
S
E
P
Q
L
I
-20 -18 -16 -14 -12 -10 -8 -6 -4 -2 0 2 4 6 8 10 12 14 16 18 20
Site

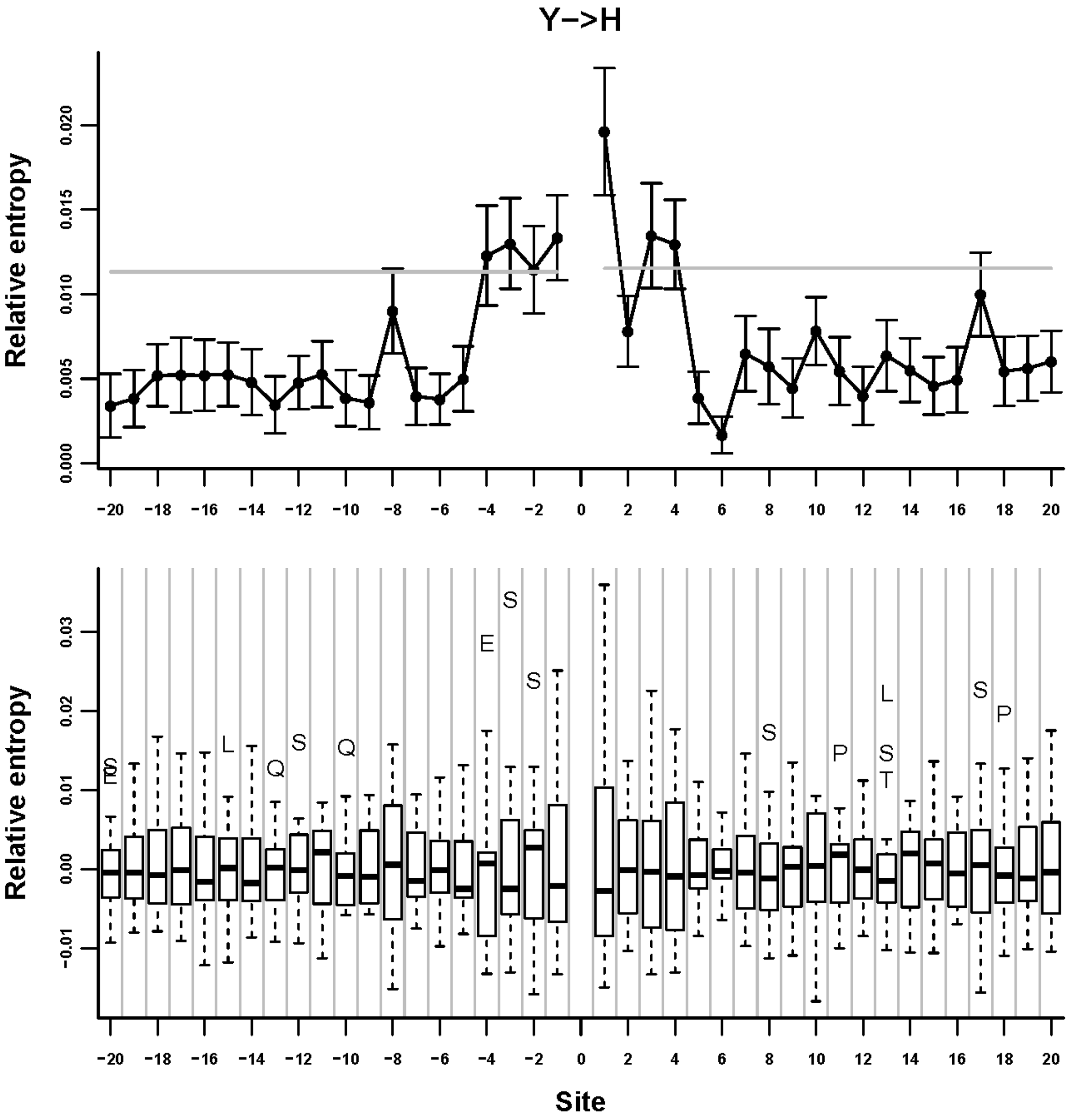

Y−>H
Relative entropy
0.000
0.005
0.010
0.015
0.020
−20
−18
−16
−14
−12
−10
−8
−6
−4
−2
0
2
4
6
8
10
12
14
16
18
20
Relative entropy
−0.01
0.00
0.01
0.02
0.03
Site

Figure S6. Context-dependence patterns of the amino acid substitutions in α-helix.

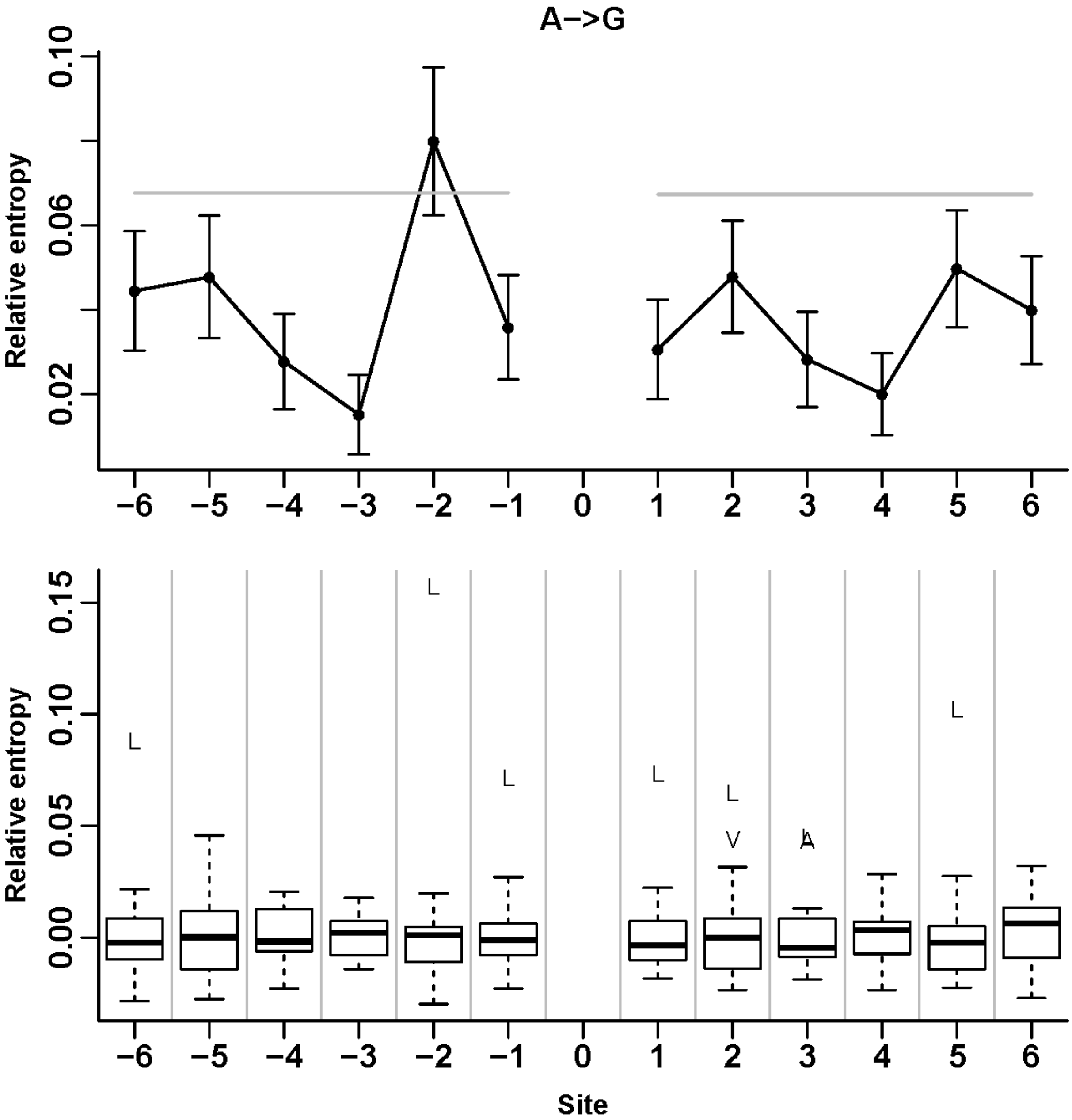

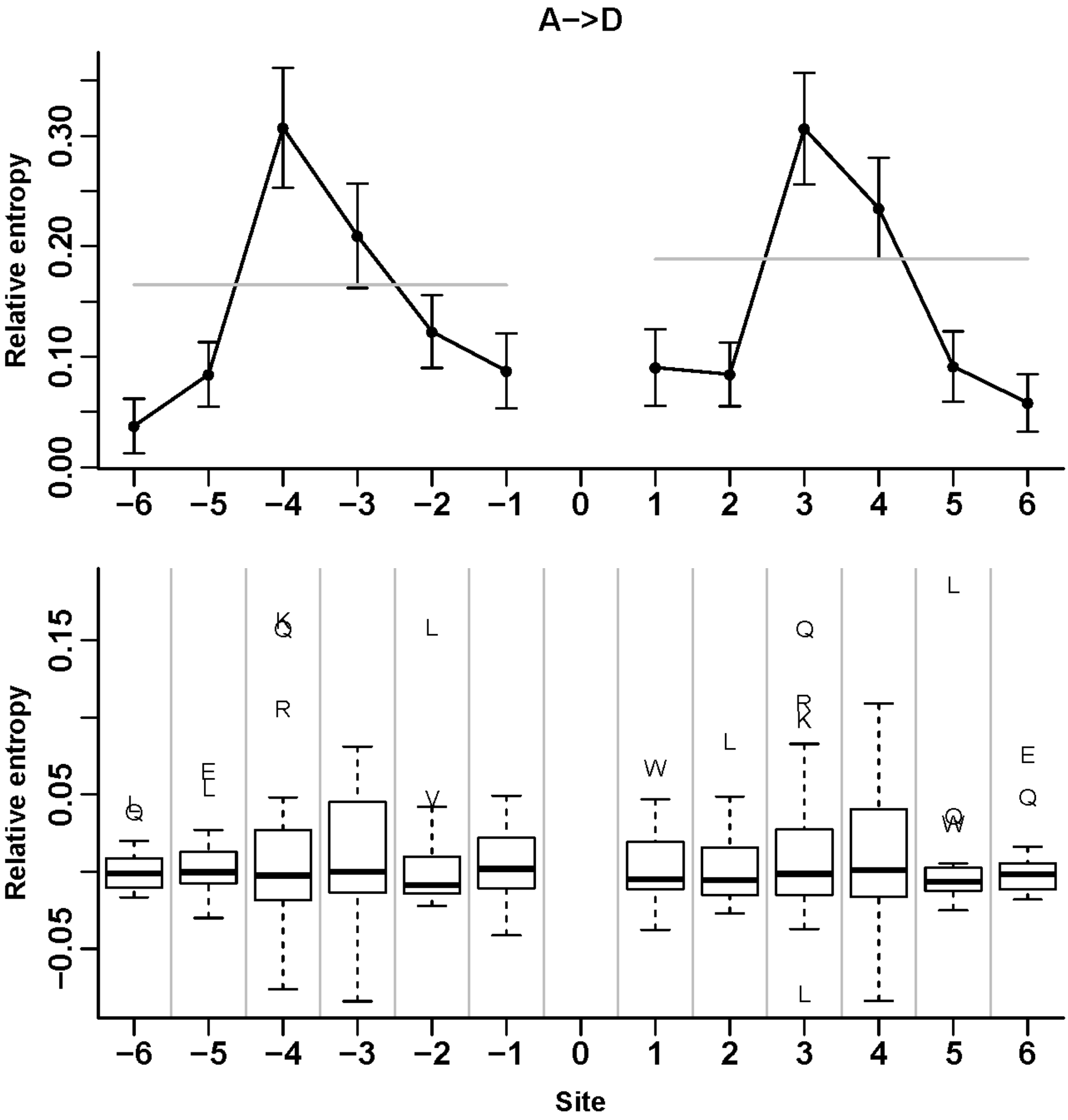
A−>D
Relative entropy
0.00
0.10
0.20
0.30
−6
−5
−4
−3
−2
−1
0
1
2
3
4
5
6
Relative entropy
−0.05
0.05
0.15
Site

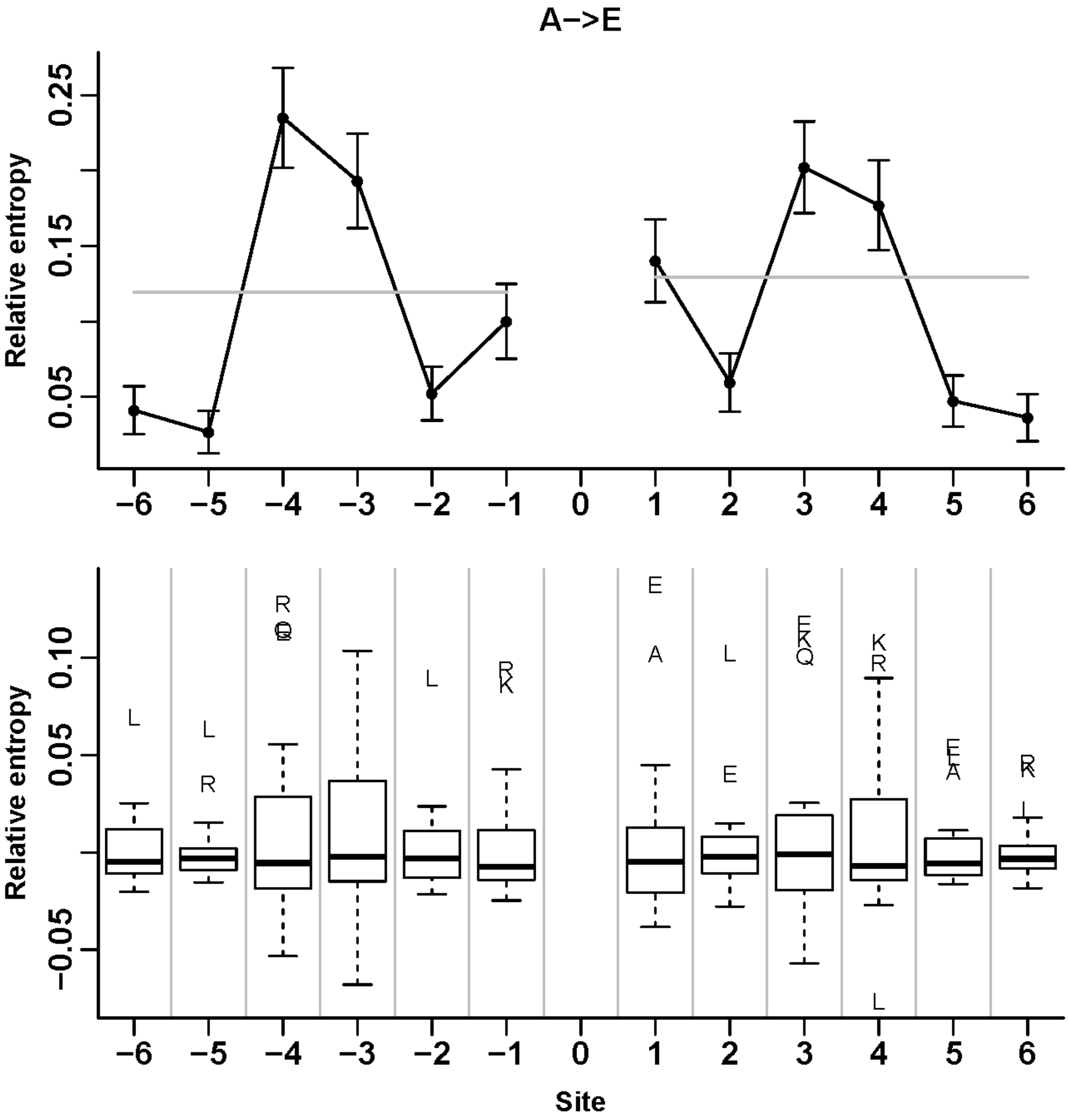

A−>E
Relative entropy
0.05
0.15
0.25
−6
−5
−4
−3
−2
−1
0
1
2
3
4
5
6
Relative entropy
−0.05
0.05
0.10
Site

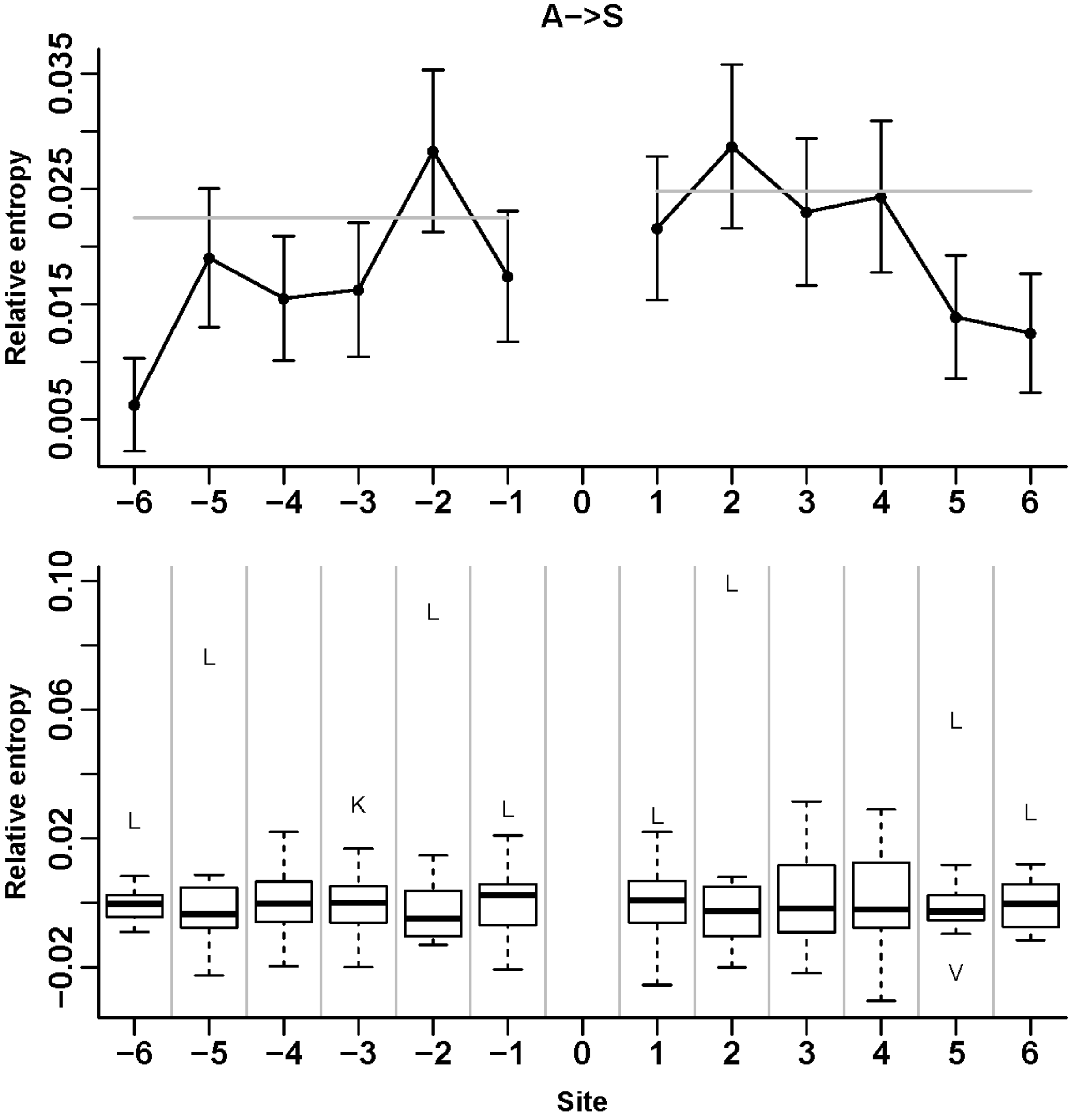
A−>S
Relative entropy
0.005
0.015
0.025
0.035
−6
−5
−4
−3
−2
−1
0
1
2
3
4
5
6
Relative entropy
−0.02
0.02
0.06
0.10
L
L
K
L
L
L
L
L
L
V
Site

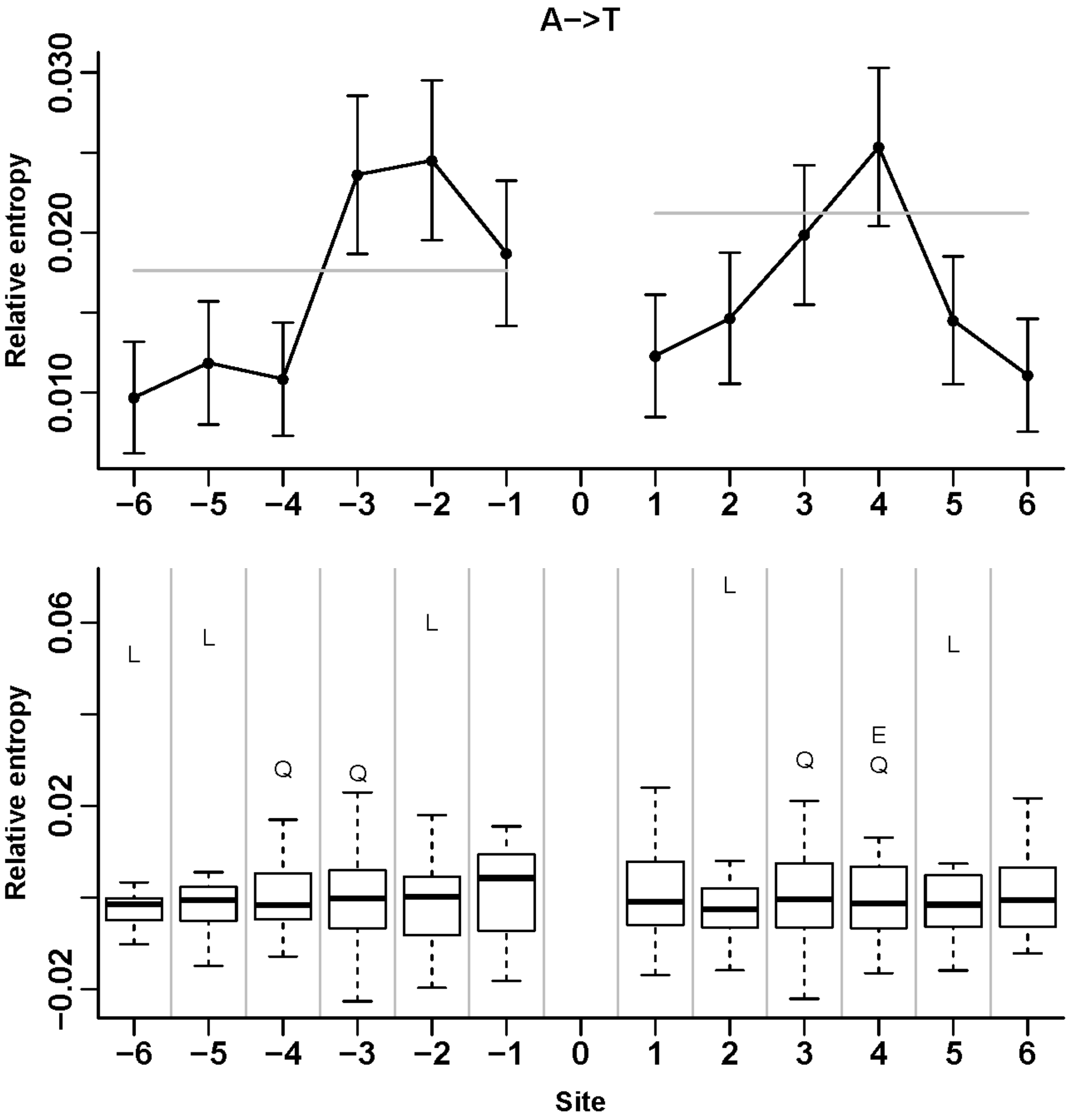
A−>T
Relative entropy
0.010
0.020
0.030
−6
−5
−4
−3
−2
−1
0
1
2
3
4
5
6
Relative entropy
−0.02
0.02
0.06
L
L
Q
Q
L
L
Q
E
Q
L
Site

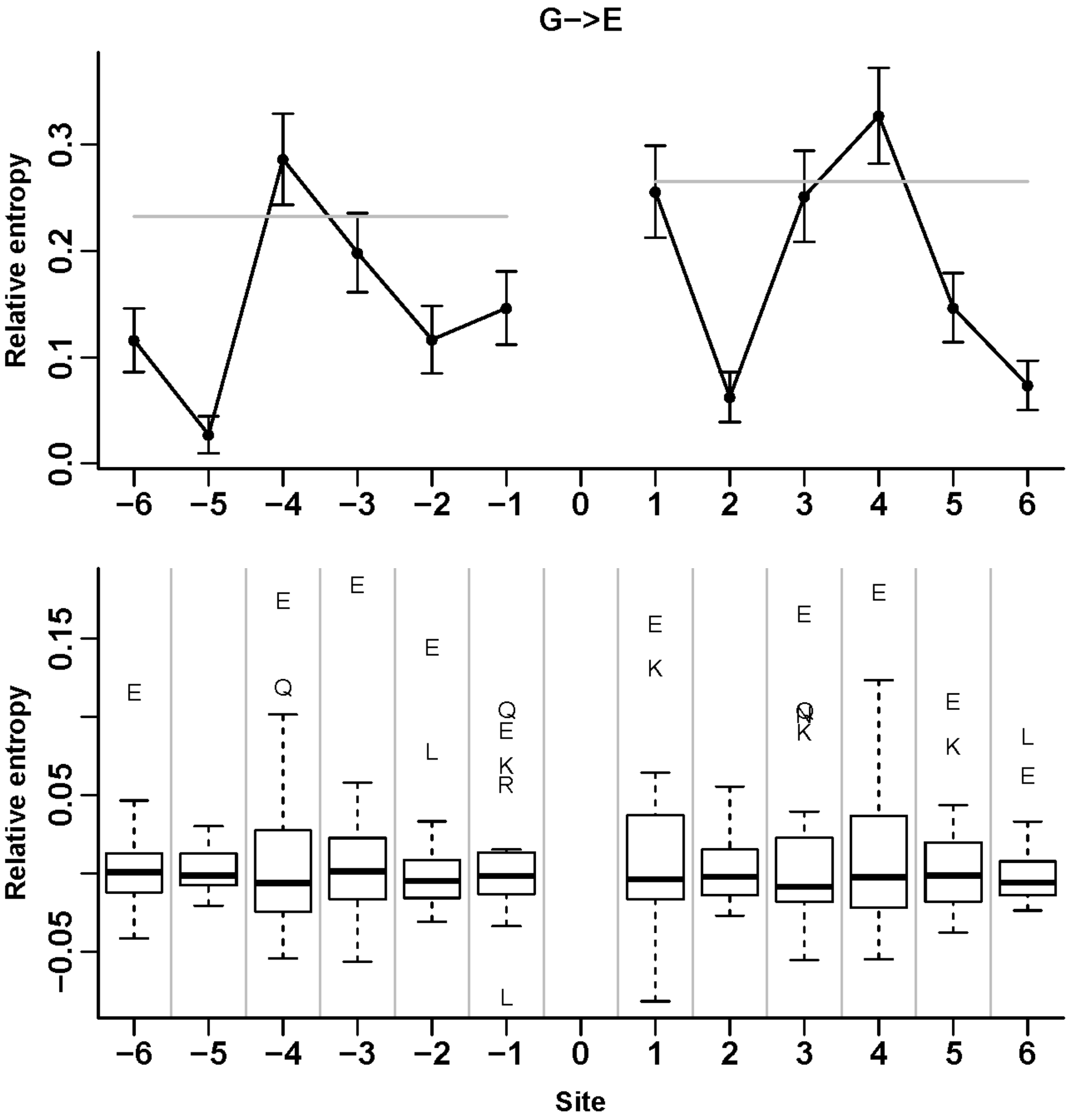
G−>E
Relative entropy
0.0
0.1
0.2
0.3
−6 −5 −4 −3 −2 −1 0 1 2 3 4 5 6
Relative entropy
−0.05
0.05
0.15
E
E Q
E
E L
Q E K R L
E K
E
Q N K
E
E K
L E
−6 −5 −4 −3 −2 −1 0 1 2 3 4 5 6
Site

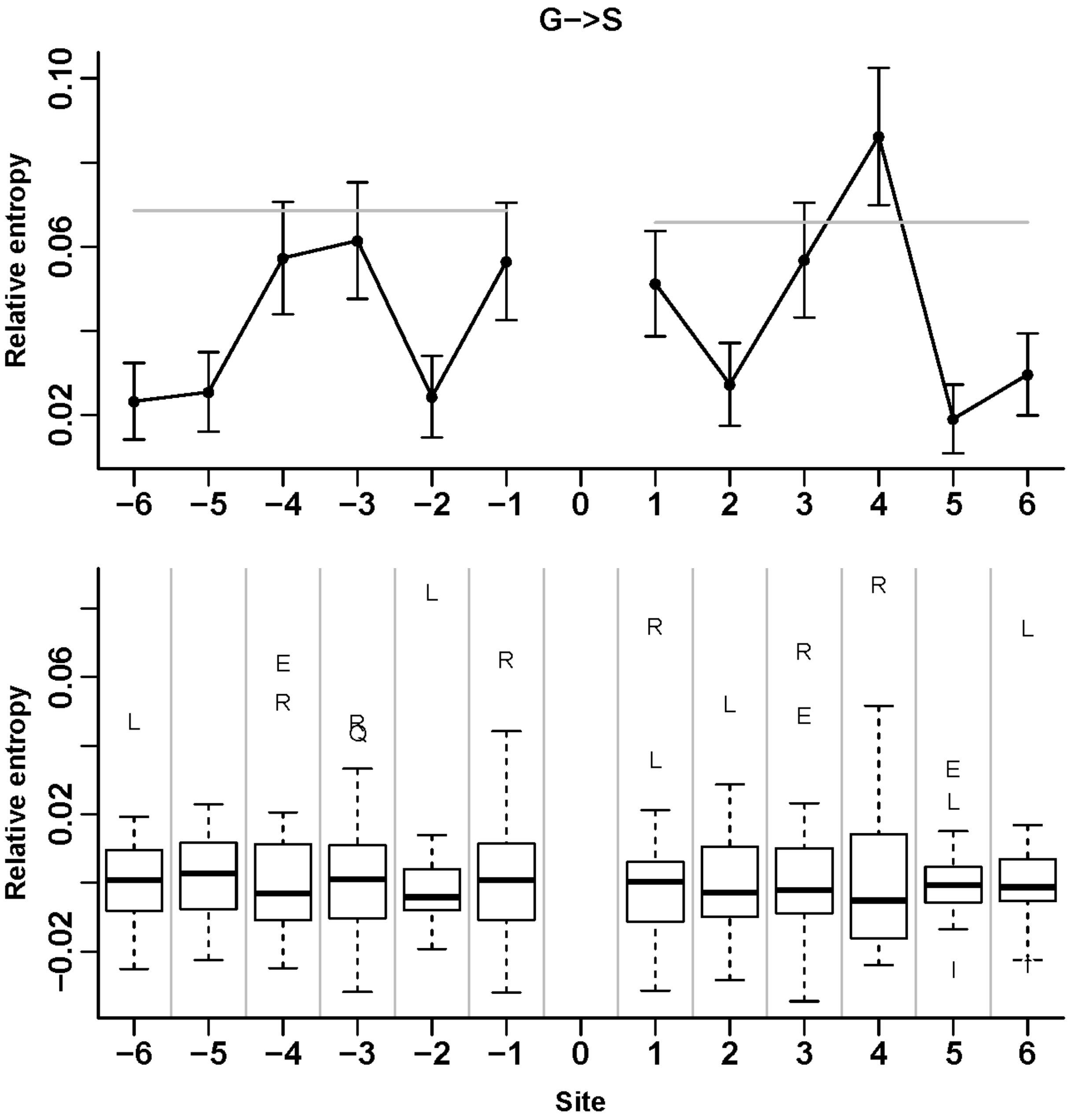
G−>S
Relative entropy
0.10
0.06
0.02
−6 −5 −4 −3 −2 −1 0 1 2 3 4 5 6
Relative entropy
0.06
0.02
−0.02
L
E
R
R
Q
L
R
R
L
L
R
E
R
E
L
L
I
Site

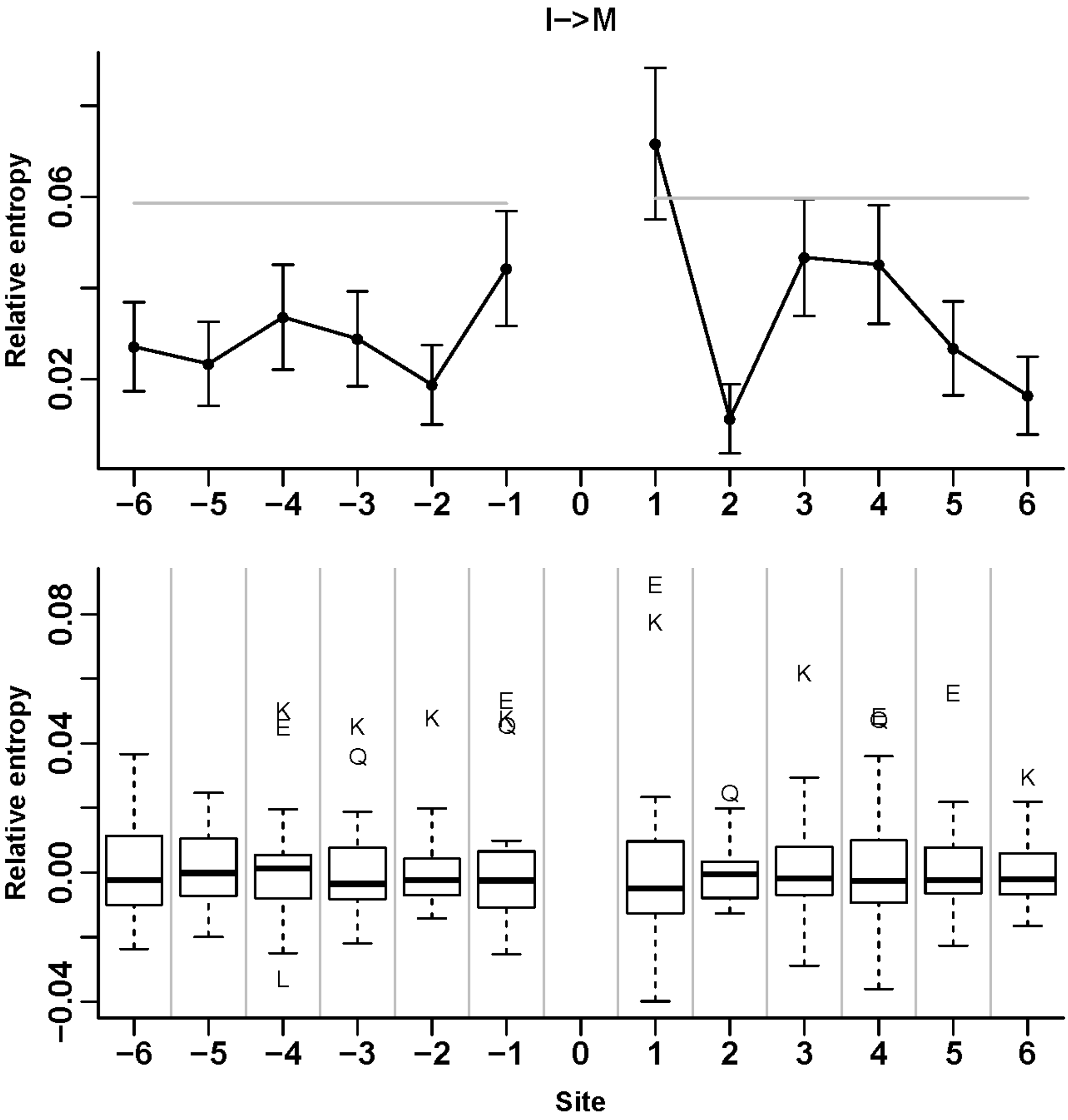
I−>M
Relative entropy
0.02
0.06
−6 −5 −4 −3 −2 −1 0 1 2 3 4 5 6
Relative entropy
−0.04
0.00
0.04
0.08
E
K
K
E
Q
K
K
E
Q
K
L
Q
K
E
K
−6 −5 −4 −3 −2 −1 0 1 2 3 4 5 6
Site

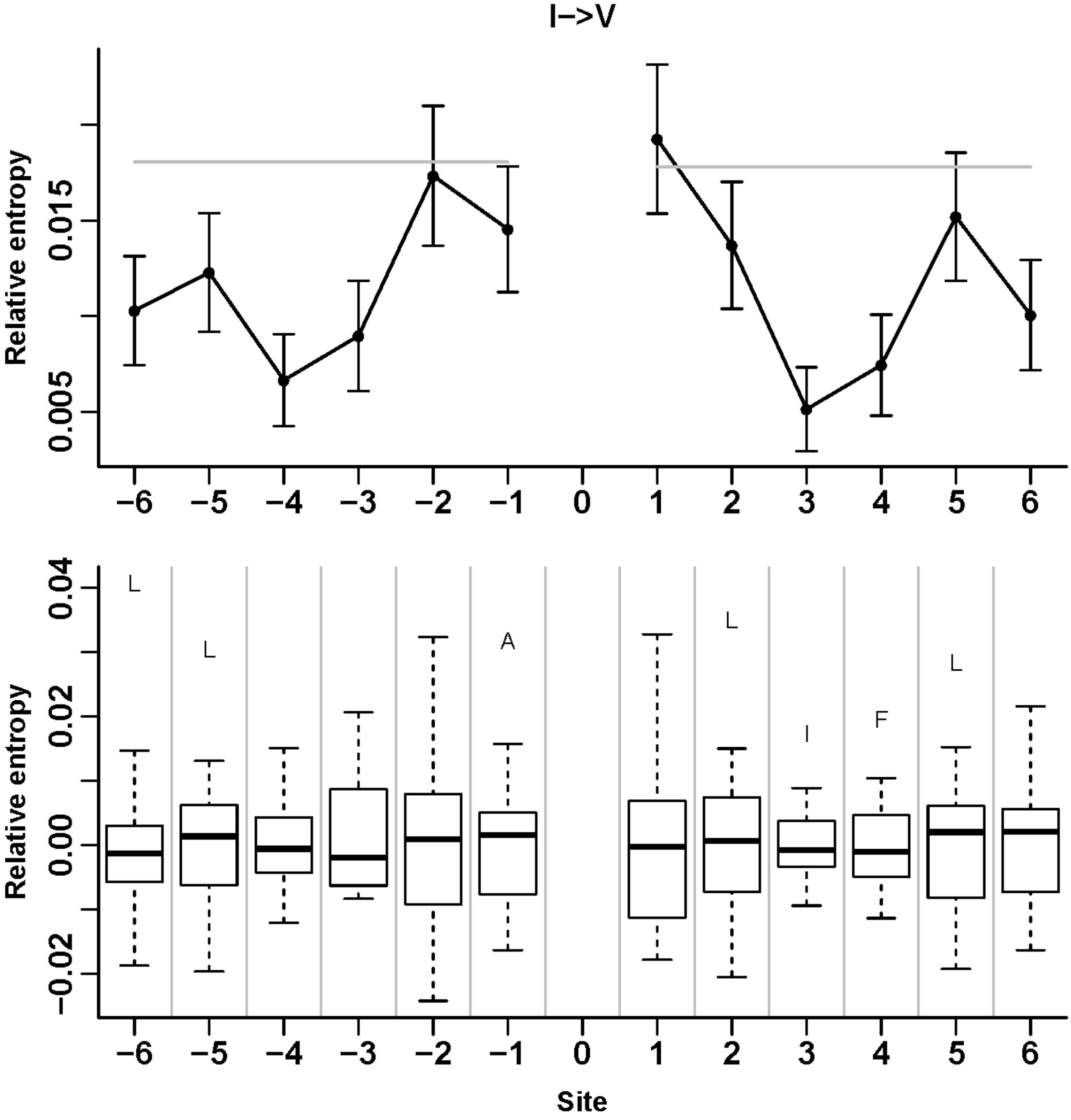
I−>V
Relative entropy
0.005
0.015
−6
−5
−4
−3
−2
−1
0
1
2
3
4
5
6
Relative entropy
−0.02
0.00
0.02
0.04
L
L
A
L
I
F
L
Site

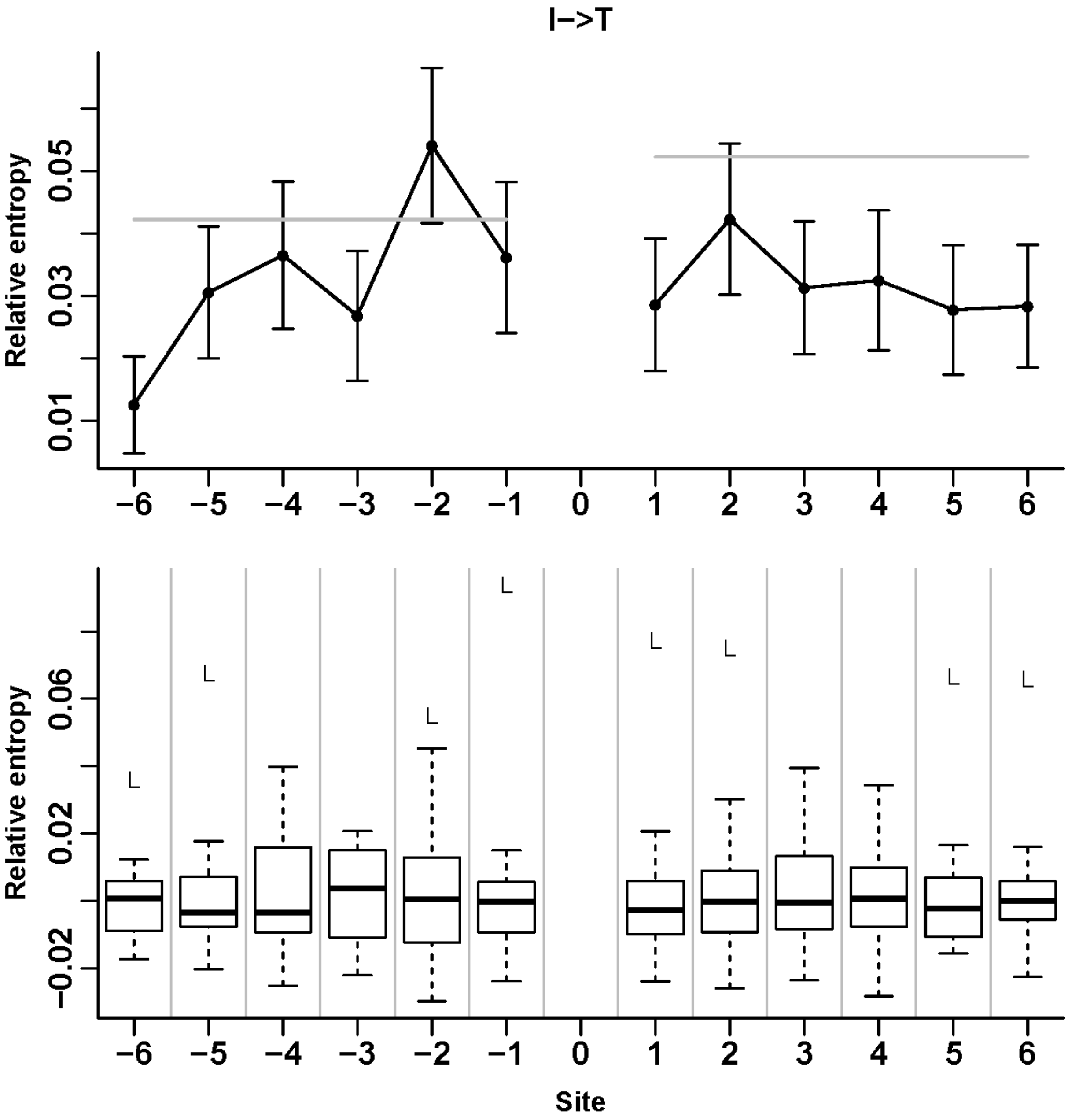
I−>T
Relative entropy
0.01
0.03
0.05
−6
−5
−4
−3
−2
−1
0
1
2
3
4
5
6
Relative entropy
−0.02
0.02
0.06
Site

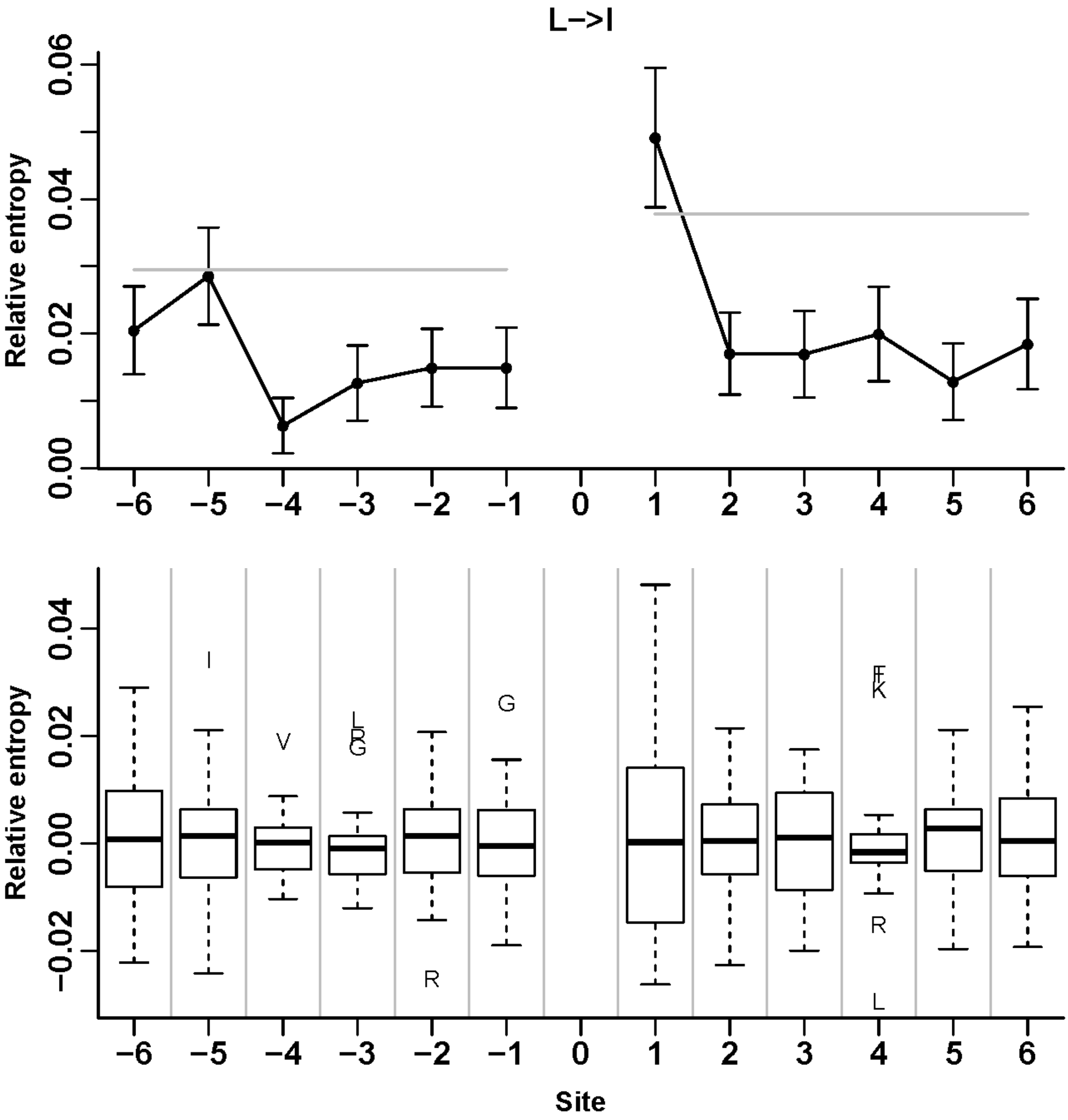
L−>I
Relative entropy
0.00
0.02
0.04
0.06
−6
−5
−4
−3
−2
−1
0
1
2
3
4
5
6
Relative entropy
−0.02
0.00
0.02
0.04
I
V
L
R
G
R
G
F
K
R
L
Site

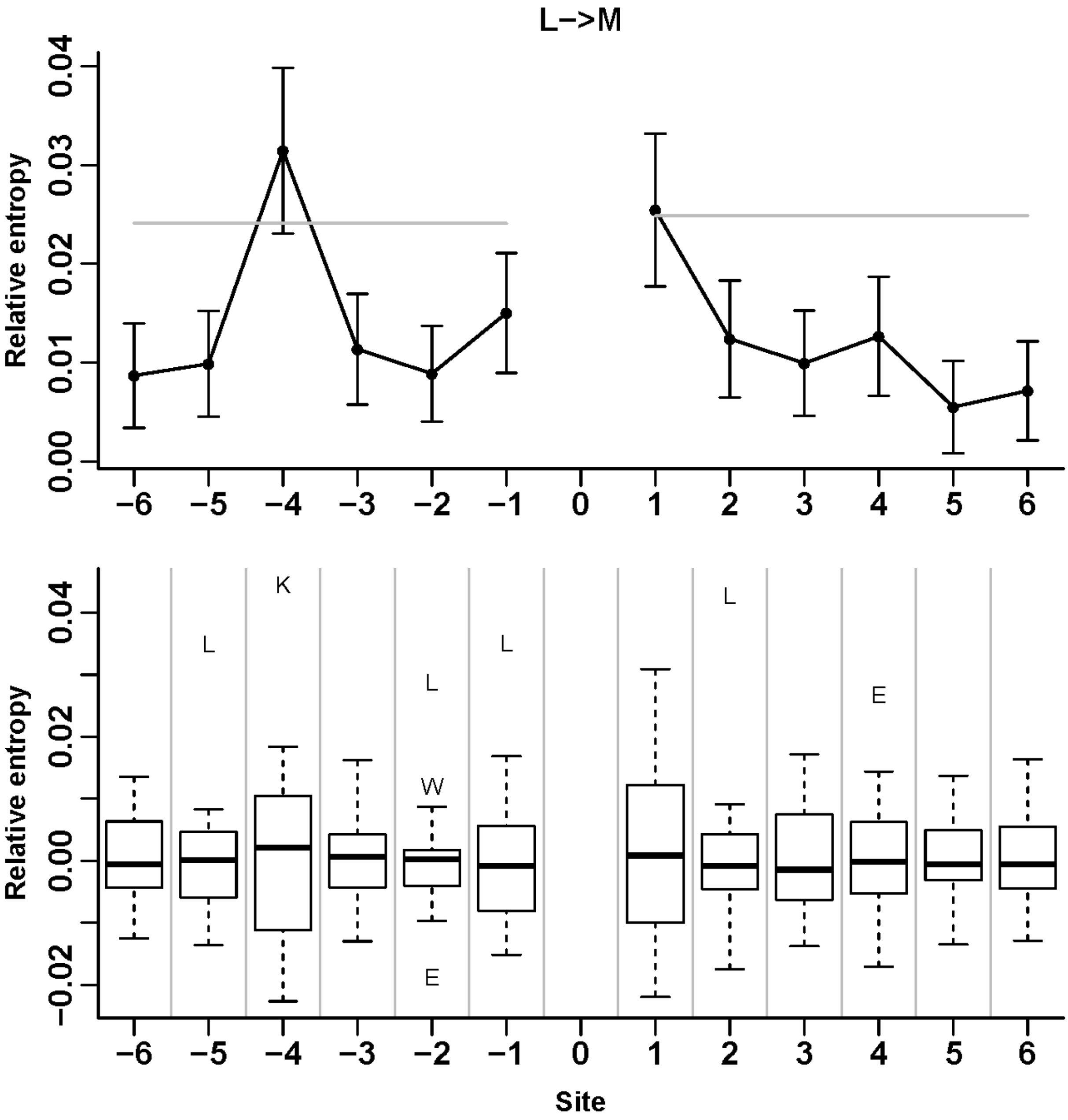
L->M
Relative entropy
0.00
0.01
0.02
0.03
0.04
−6
−5
−4
−3
−2
−1
0
1
2
3
4
5
6
K
L
L
L
L
E
W
E
L
−0.02
Site

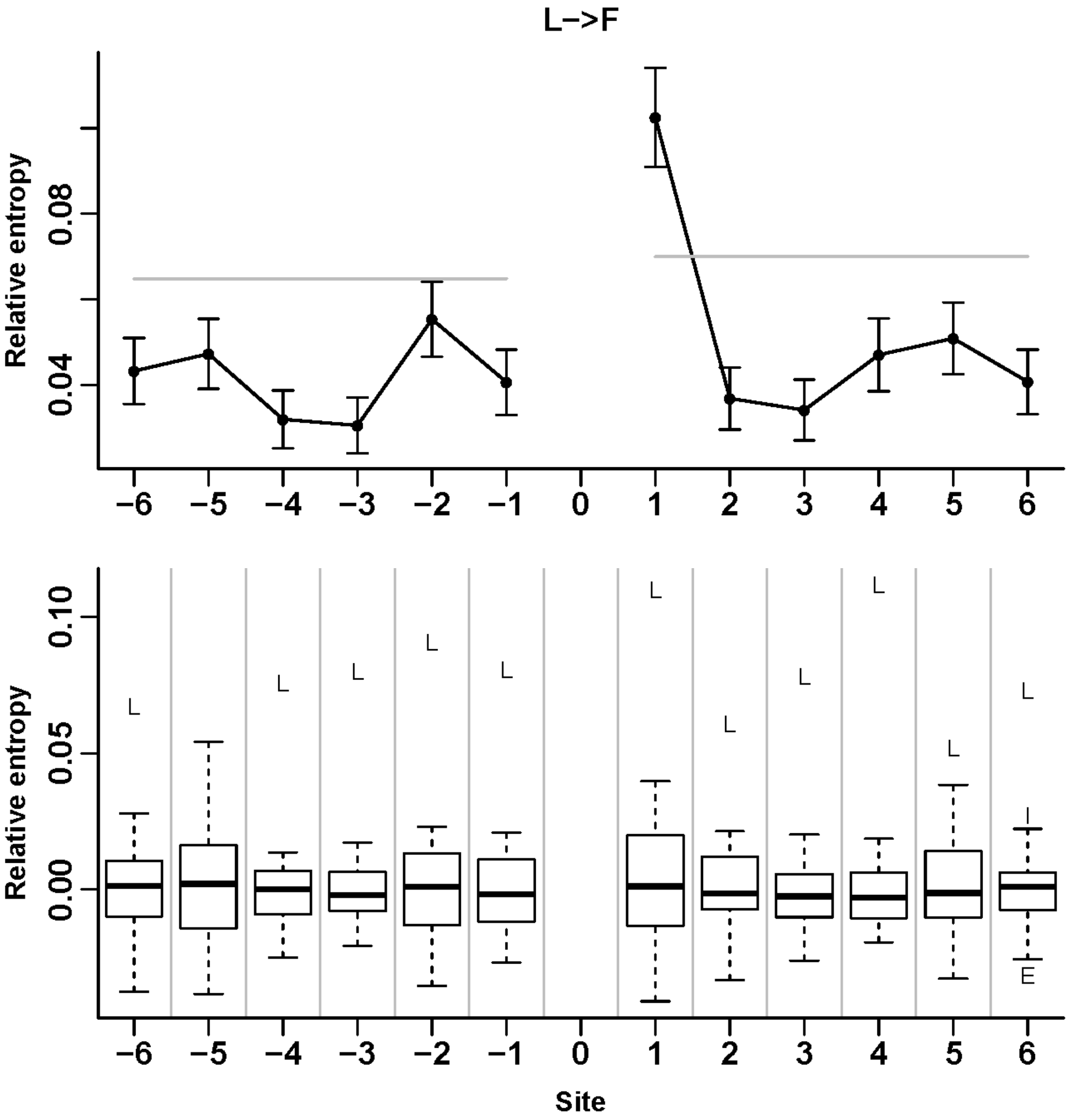

L−>F
Relative entropy
0.04
0.08
−6
−5
−4
−3
−2
−1
0
1
2
3
4
5
6
Relative entropy
0.00
0.05
0.10
L
E
Site

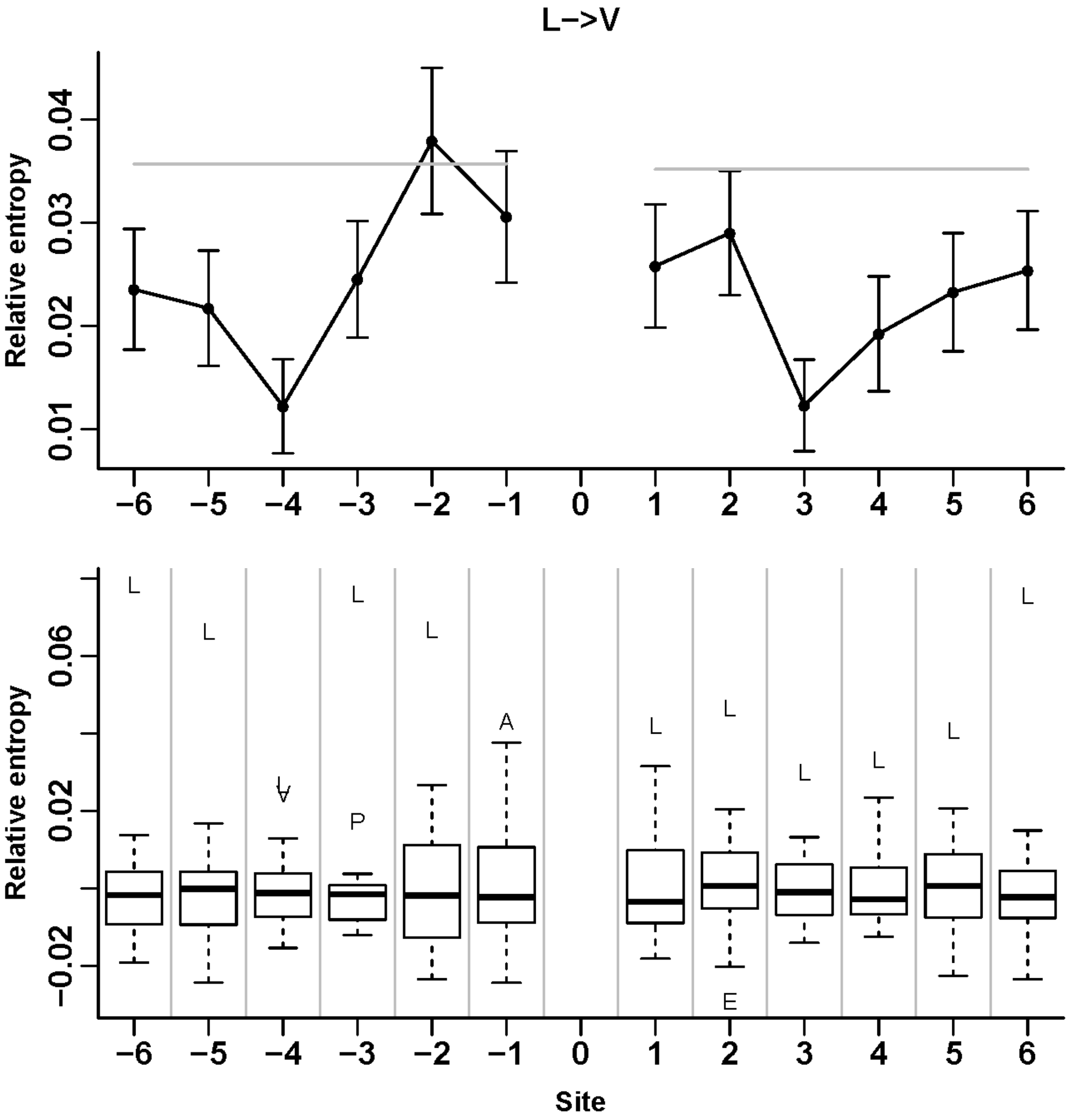
L−>V
Relative entropy
0.01
0.02
0.03
0.04
−6
−5
−4
−3
−2
−1
0
1
2
3
4
5
6
Relative entropy
−0.02
0.02
0.06
L
L
L
L
A
L
L
L
L
L
L
P
E
Site

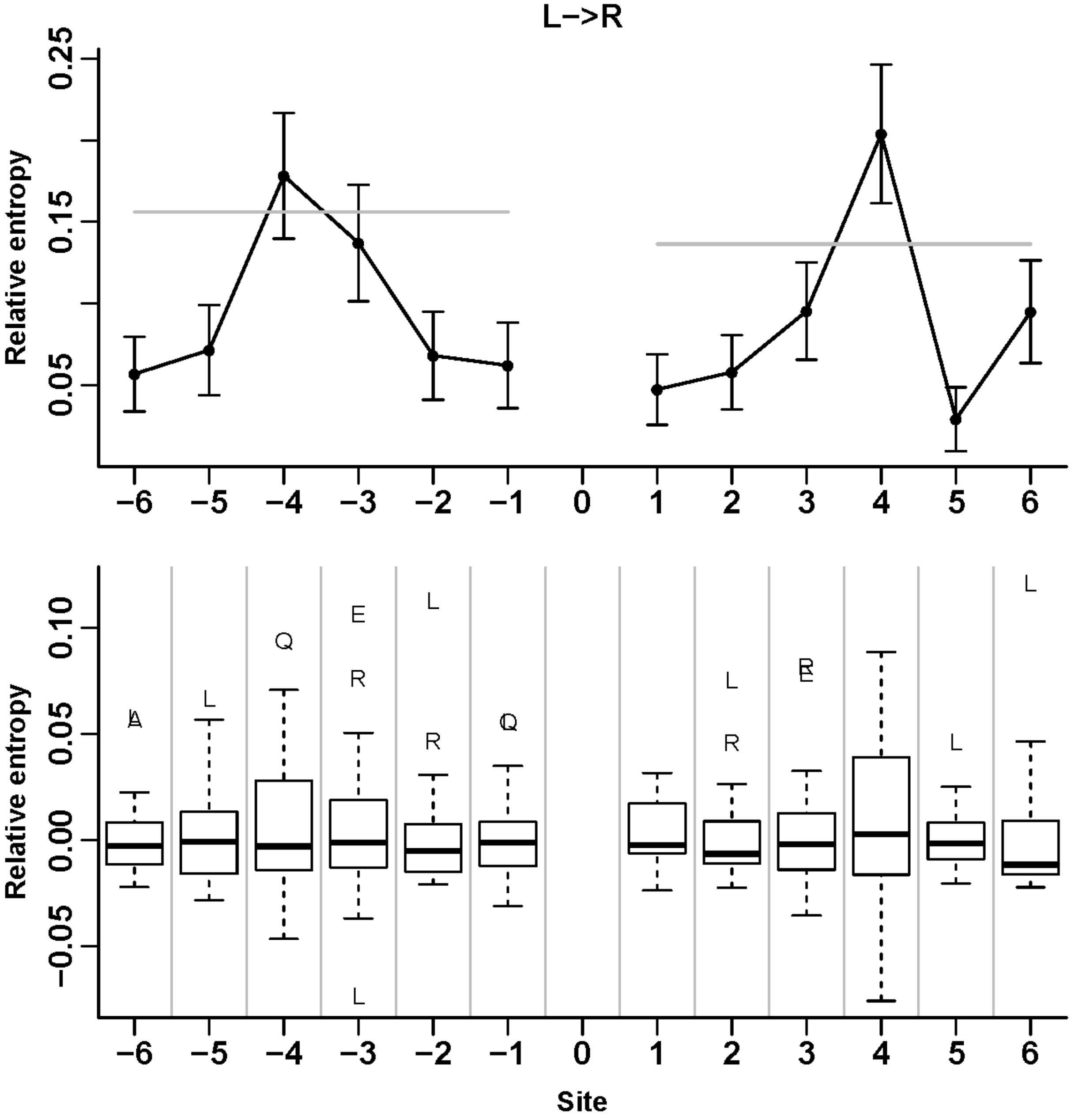
L−>R
Relative entropy
0.05
0.15
0.25
−6
−5
−4
−3
−2
−1
0
1
2
3
4
5
6
Relative entropy
−0.05
0.00
0.05
0.10
Site

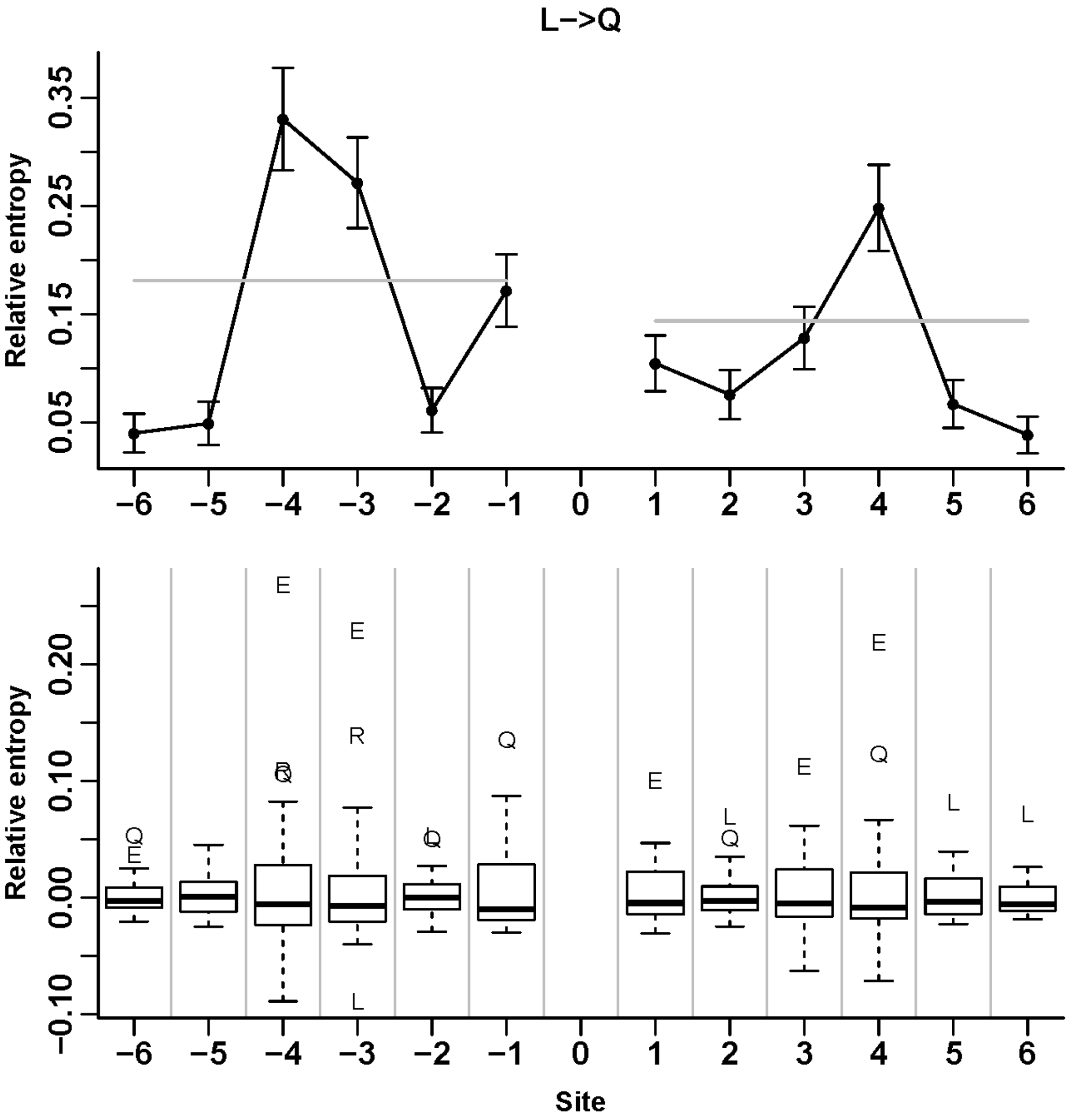
L−>Q
Relative entropy
0.05
0.15
0.25
0.35
−6
−5
−4
−3
−2
−1
0
1
2
3
4
5
6
Relative entropy
−0.10
0.00
0.10
0.20
E
E
R
Q
Q
E
Q
L
Q
E
Q
E
L
Q
L
L
Site

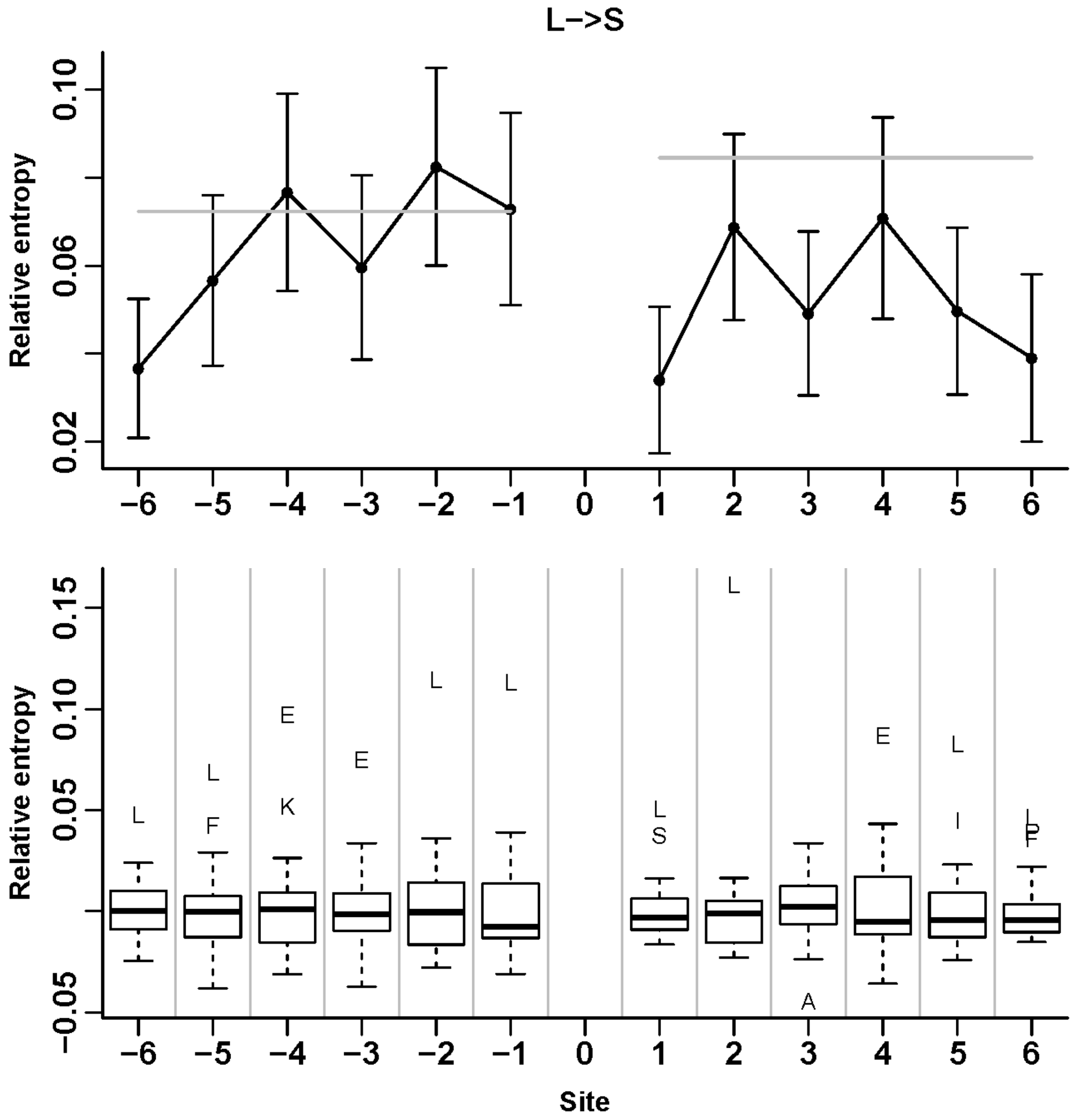
L−>S
Relative entropy
0.02
0.06
0.10
−6
−5
−4
−3
−2
−1
0
1
2
3
4
5
6
Relative entropy
−0.05
0.05
0.10
0.15
L
L
F
E
K
E
L
L
L
S
L
A
E
L
I
L
P
F
Site

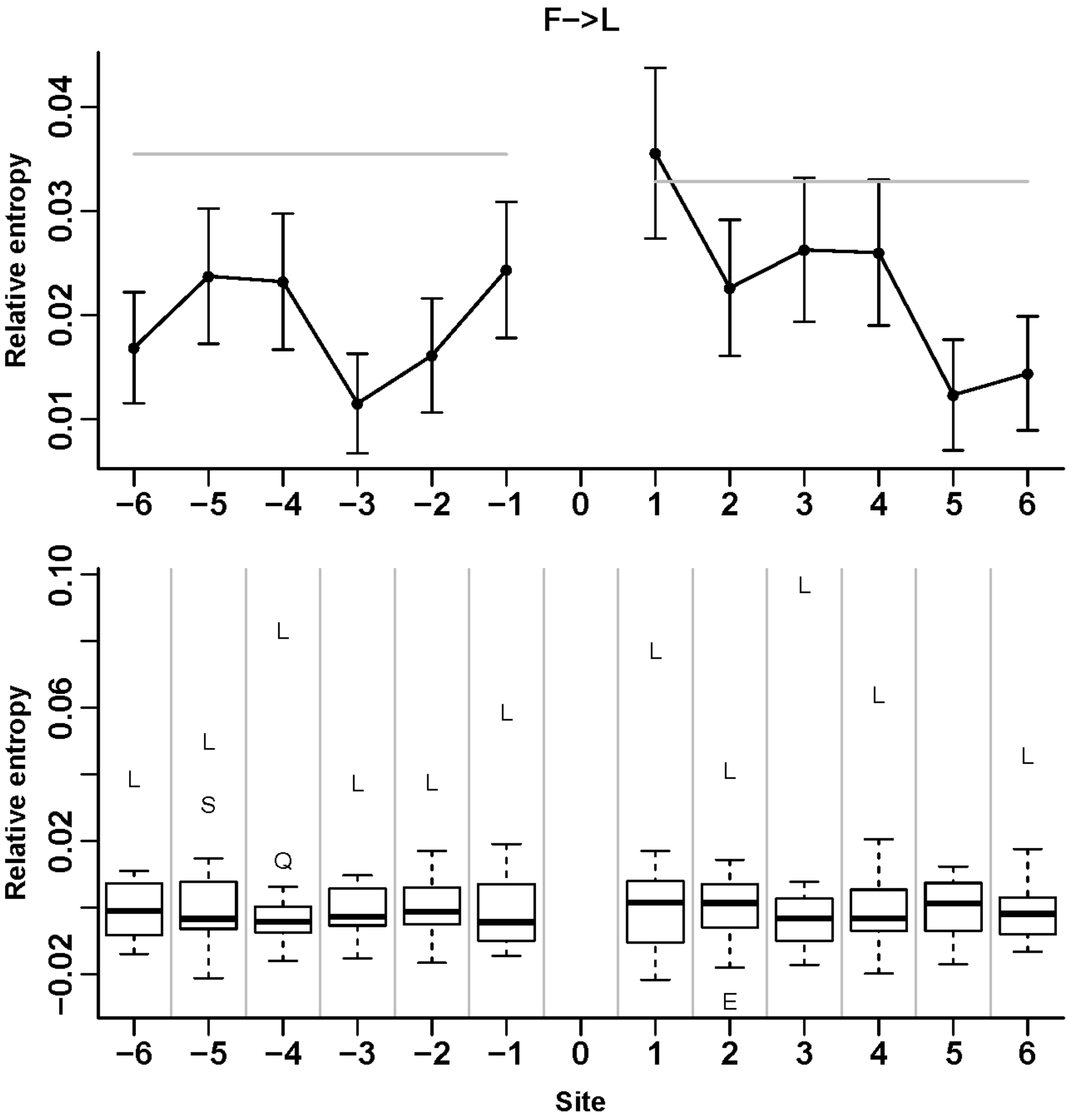
F−>L
Relative entropy
0.01
0.02
0.03
0.04
−6
−5
−4
−3
−2
−1
0
1
2
3
4
5
6
Relative entropy
−0.02
0.02
0.06
0.10
L
L
S
L
Q
L
L
L
L
L
L
E
L
L
L
Site

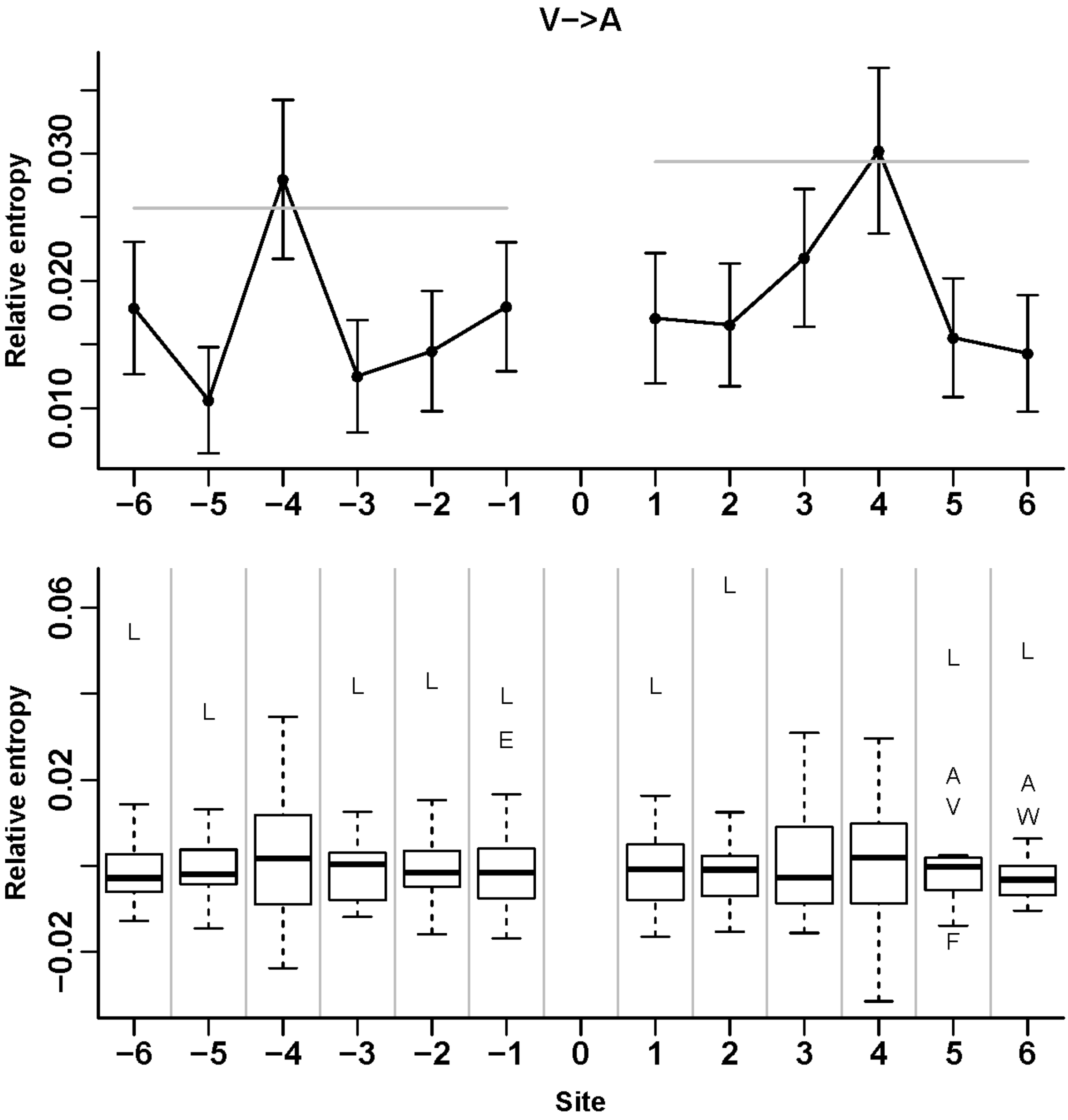
V−>A
Relative entropy
0.010
0.020
0.030
−6
−5
−4
−3
−2
−1
0
1
2
3
4
5
6
Relative entropy
−0.02
0.02
0.06
L
L
L
L
L
E
L
L
L
L
A
V
F
A
W
Site

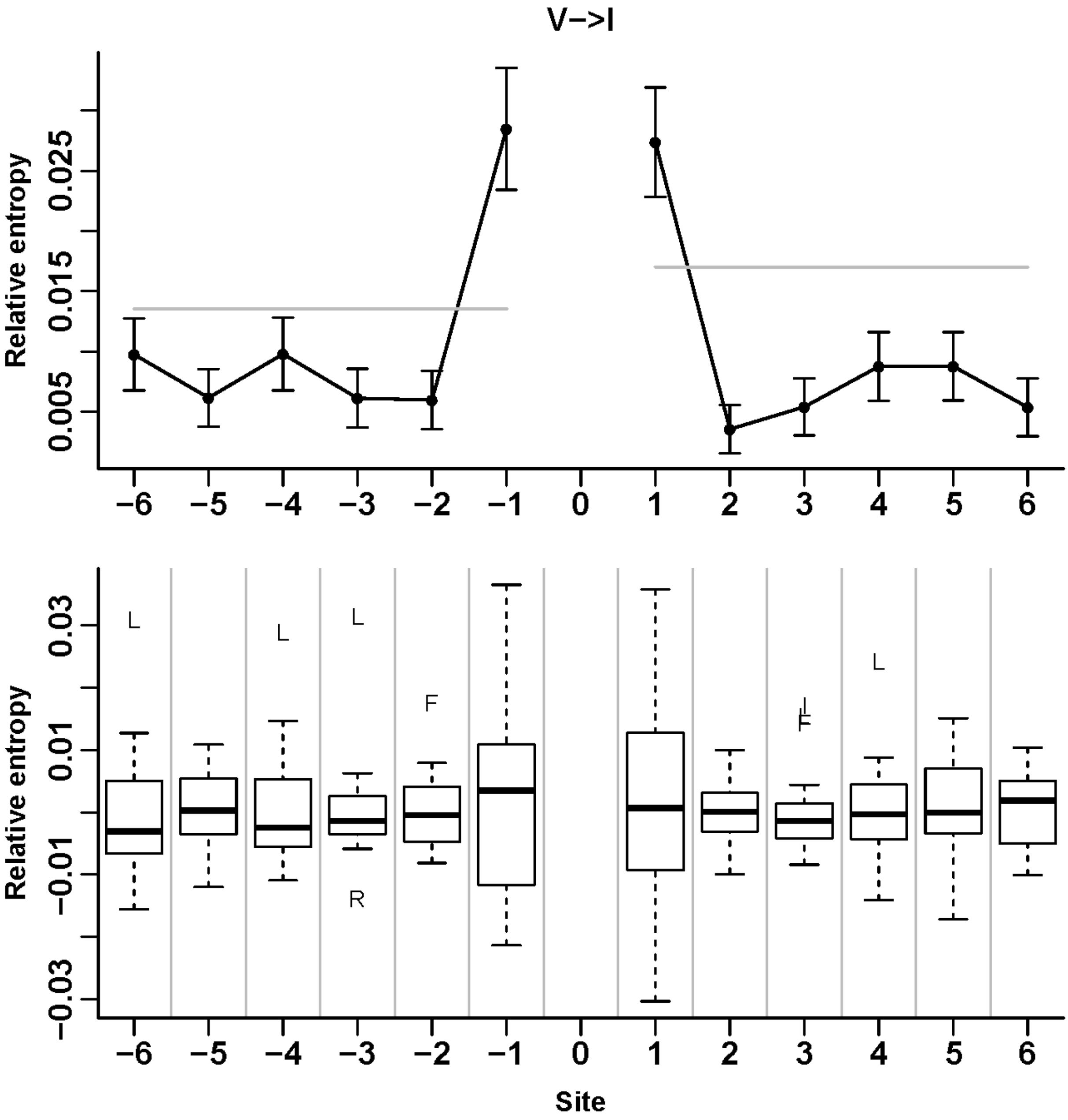
V−>I
Relative entropy
0.005
0.015
0.025
−6
−5
−4
−3
−2
−1
0
1
2
3
4
5
6
Relative entropy
−0.03
−0.01
0.01
0.03
L
L
L
F
R
L
F
L
Site

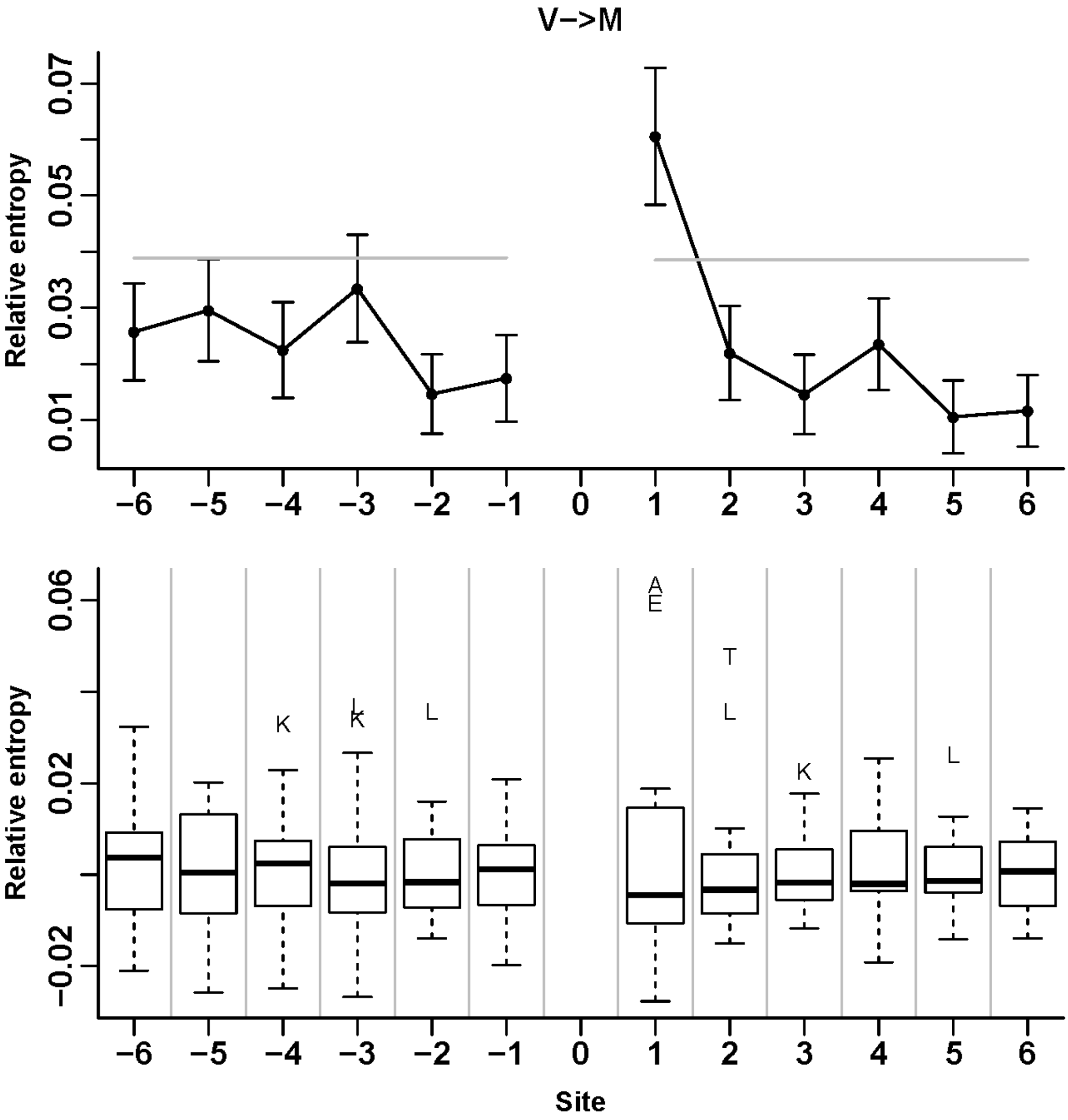
V−>M
Relative entropy
0.07
0.05
0.03
0.01
−6
−5
−4
−3
−2
−1
0
1
2
3
4
5
6
Relative entropy
0.06
0.02
−0.02
A
E
T
L
K
L
K
L
K
L
Site

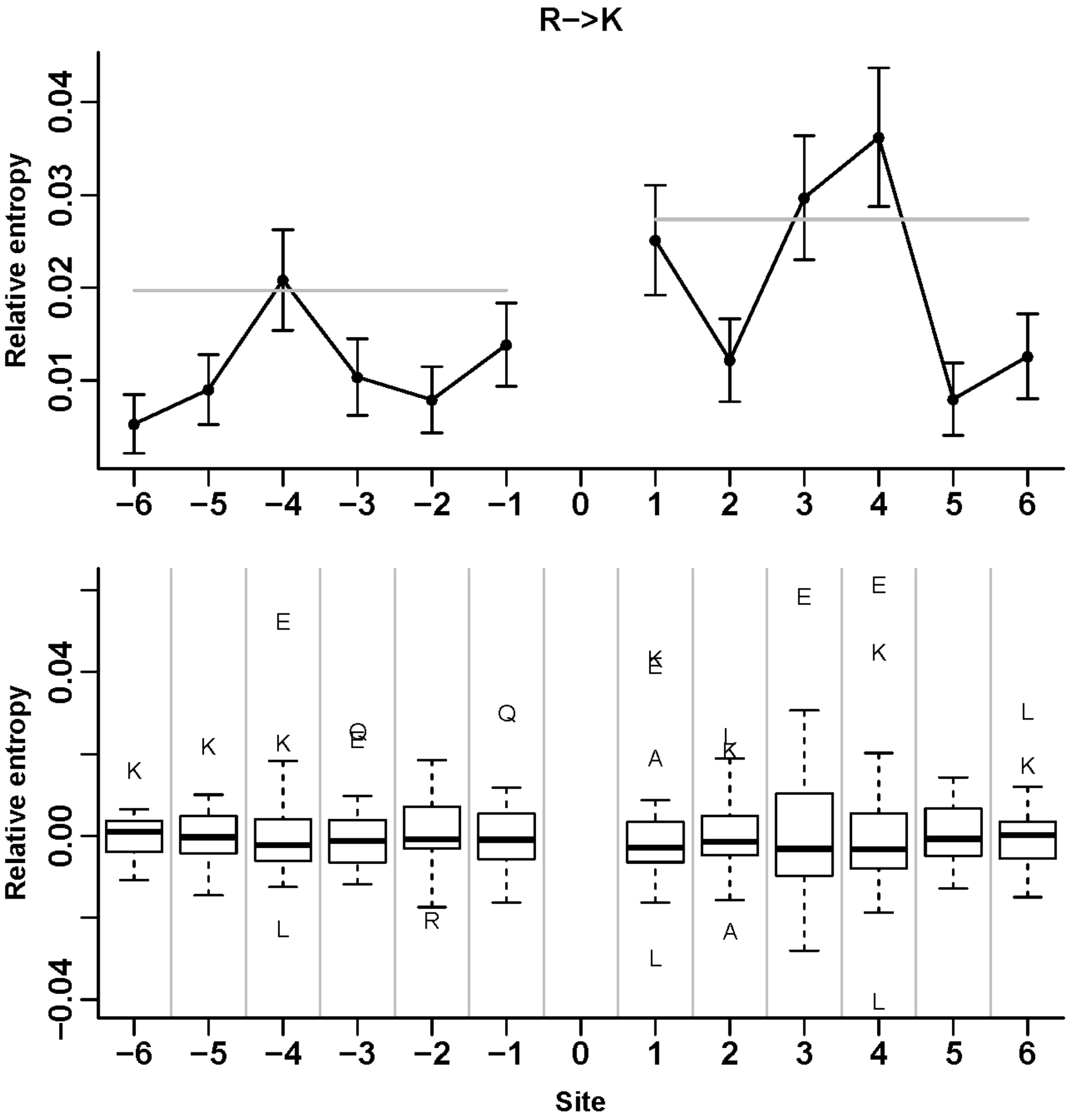
R−>K
Relative entropy
0.01
0.02
0.03
0.04
−6
−5
−4
−3
−2
−1
0
1
2
3
4
5
6
Relative entropy
−0.04
0.00
0.04
Site

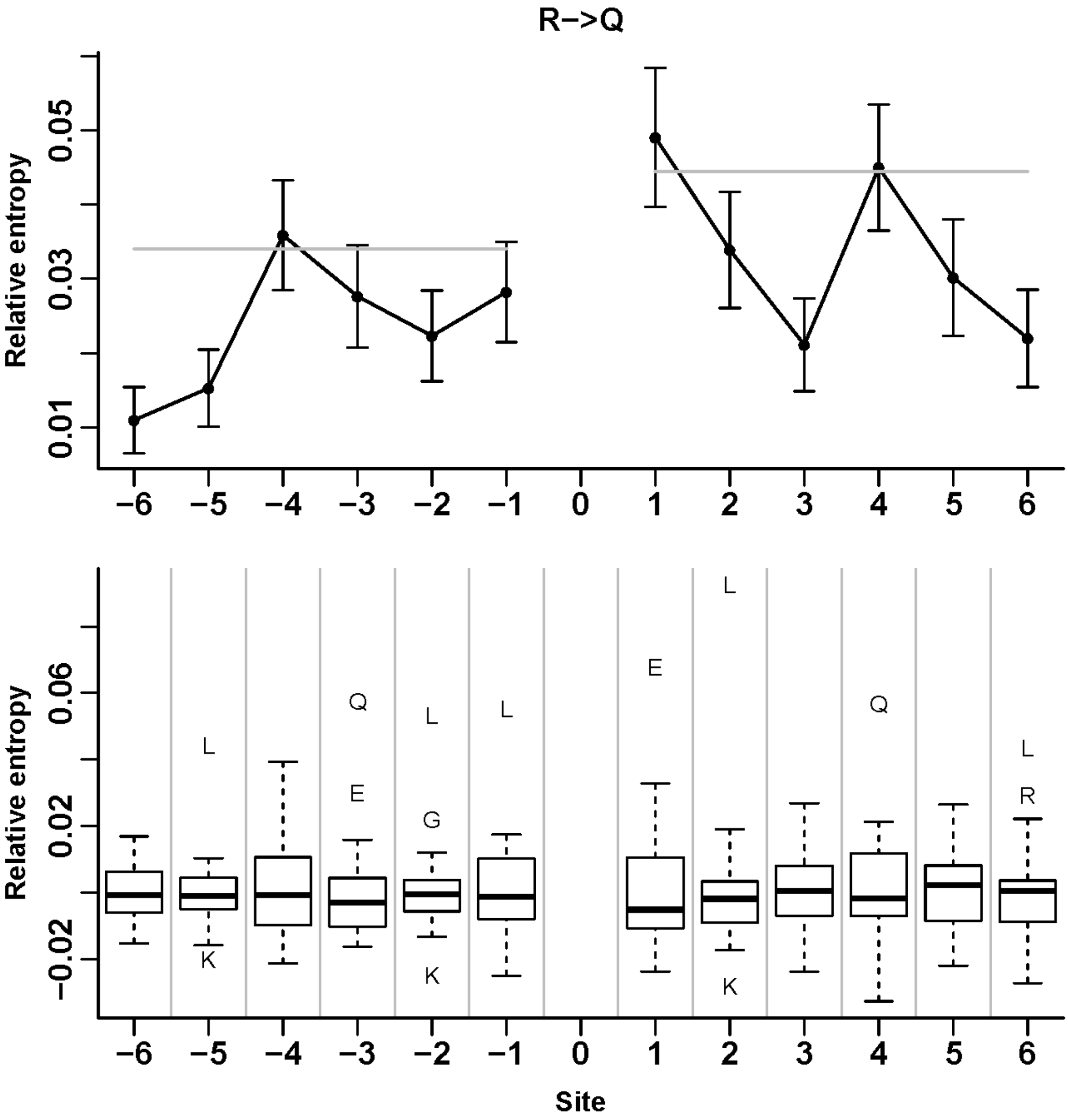
R−>Q
Relative entropy
0.01
0.03
0.05
−6
−5
−4
−3
−2
−1
0
1
2
3
4
5
6
Relative entropy
−0.02
0.02
0.06
L
K
Q
E
L
G
K
L
E
L
K
Q
L
R
Site

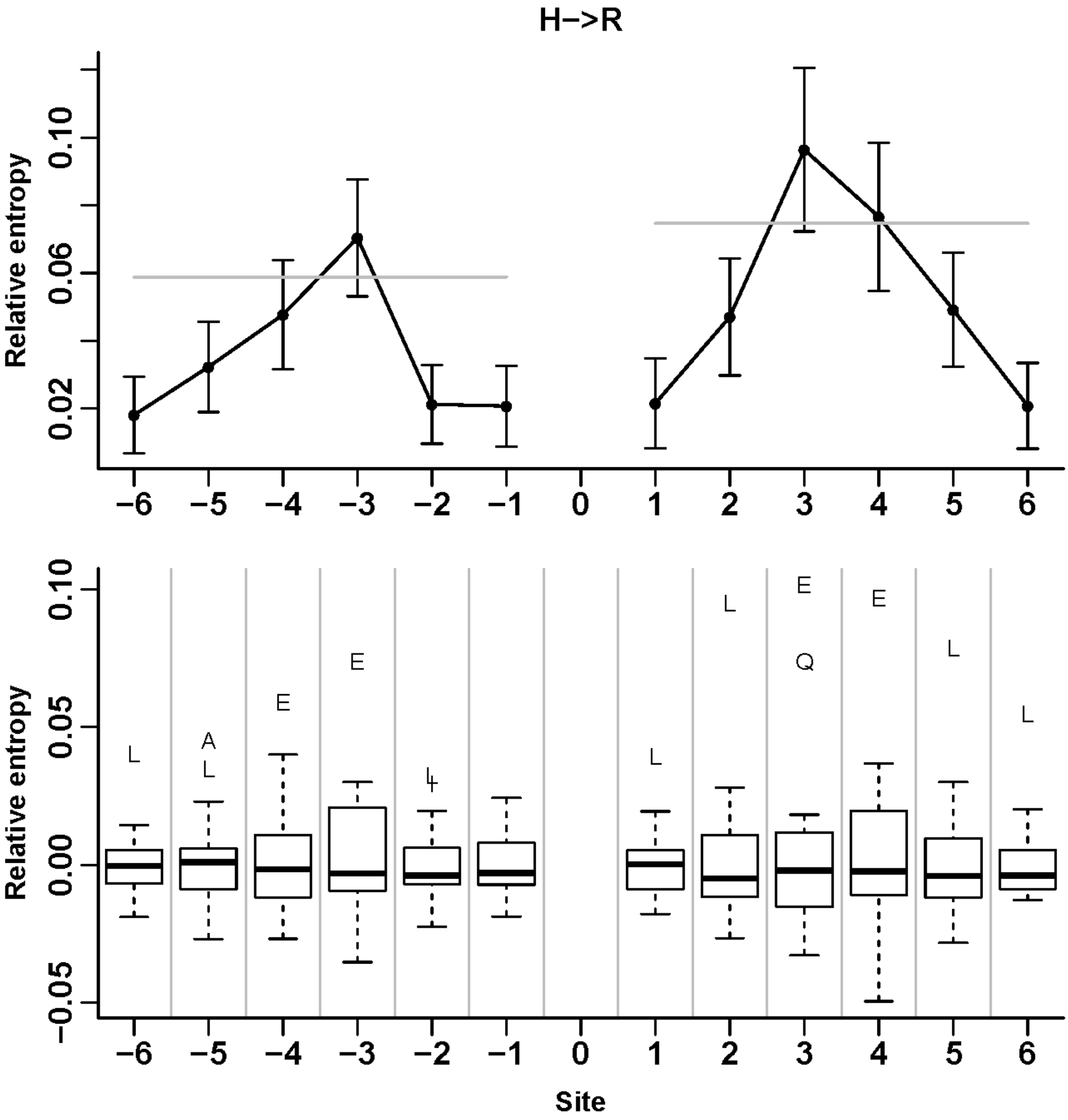
H−>R
Relative entropy
0.02
0.06
0.10
−6
−5
−4
−3
−2
−1
0
1
2
3
4
5
6
Relative entropy
−0.05
0.00
0.05
0.10
L
A
L
E
E
L
L
L
E
Q
E
L
L
Site

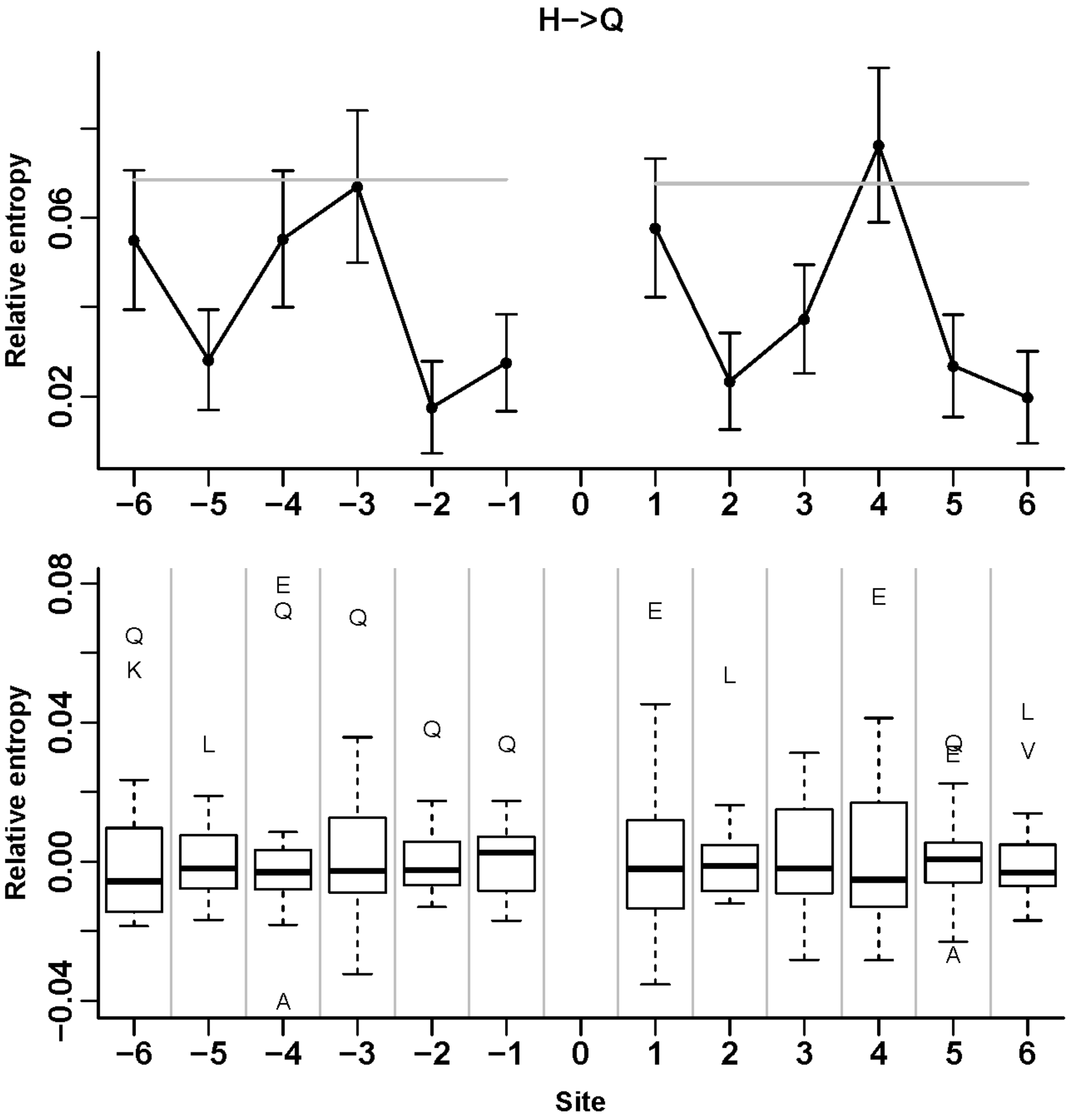
H−>Q
Relative entropy
0.02
0.06
−6
−5
−4
−3
−2
−1
0
1
2
3
4
5
6
Relative entropy
−0.04
0.00
0.04
0.08
Q
K
L
E
Q
A
Q
Q
Q
E
L
E
Q
E
A
L
V
Site

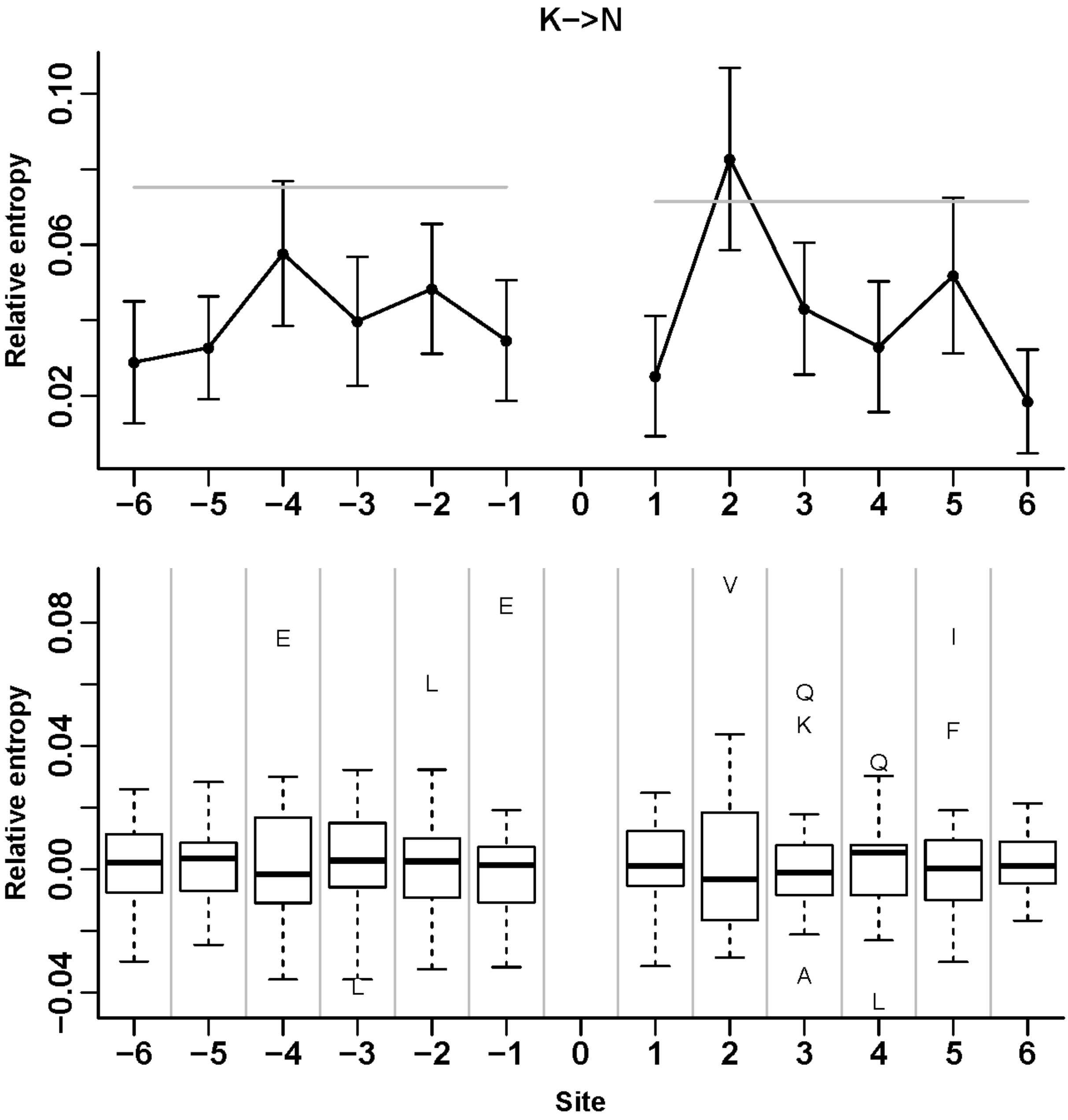
K−>N
Relative entropy
0.10
0.06
0.02
−6
−5
−4
−3
−2
−1
0
1
2
3
4
5
6
Relative entropy
0.08
0.04
0.00
−0.04
E
E
L
L
V
Q
K
A
Q
L
I
F
Site

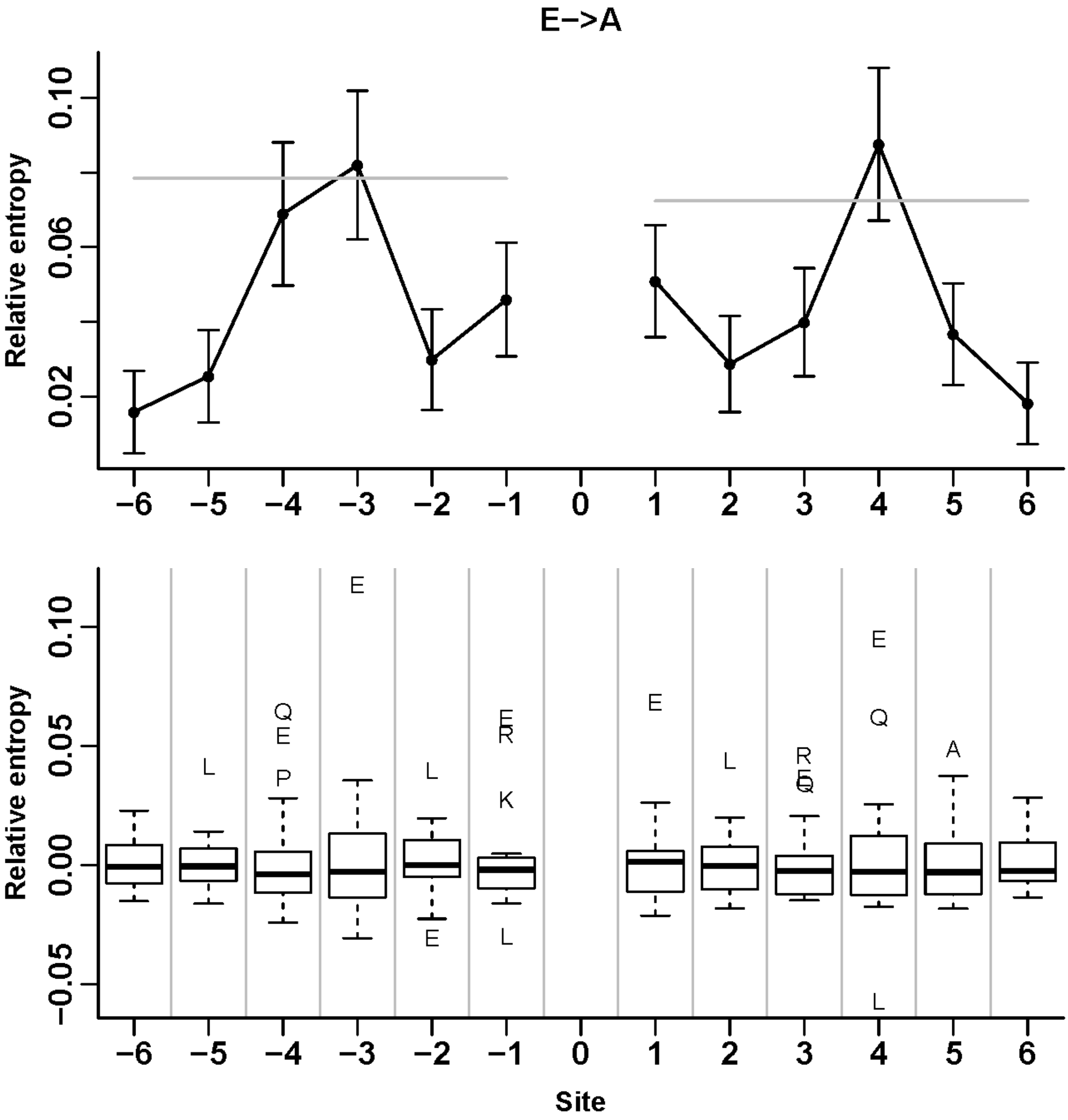

E−>A
Relative entropy
0.02
0.06
0.10
−6
−5
−4
−3
−2
−1
0
1
2
3
4
5
6
Relative entropy
−0.05
0.00
0.05
0.10
E
Q
E
P
L
L
E
E
R
K
L
E
E
L
R
E
Q
E
Q
L
A
Site

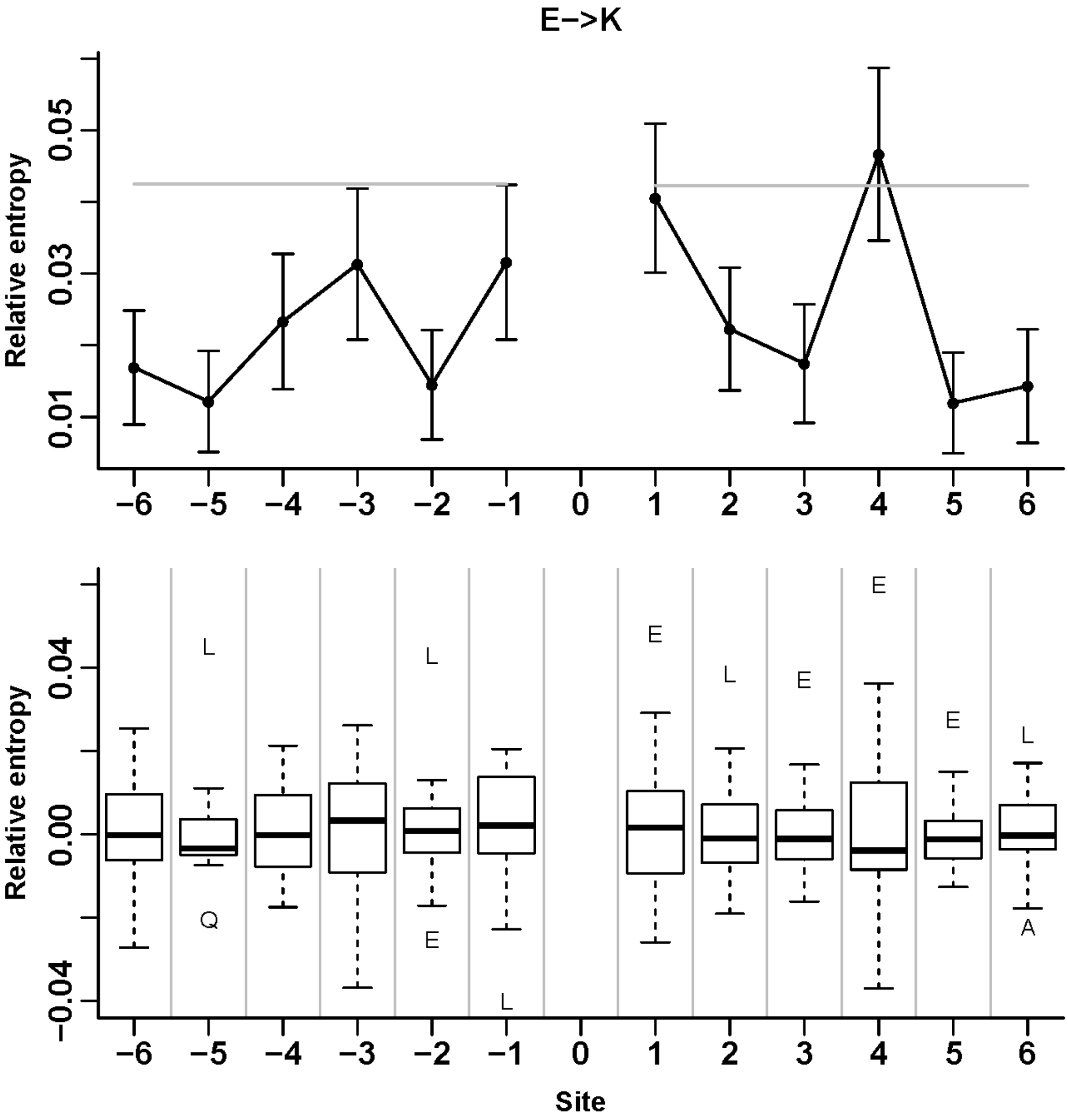
E−>K
Relative entropy
0.01
0.03
0.05
−6
−5
−4
−3
−2
−1
0
1
2
3
4
5
6
Relative entropy
−0.04
0.00
0.04
L
Q
L
E
L
E
L
E
E
E
L
A
Site

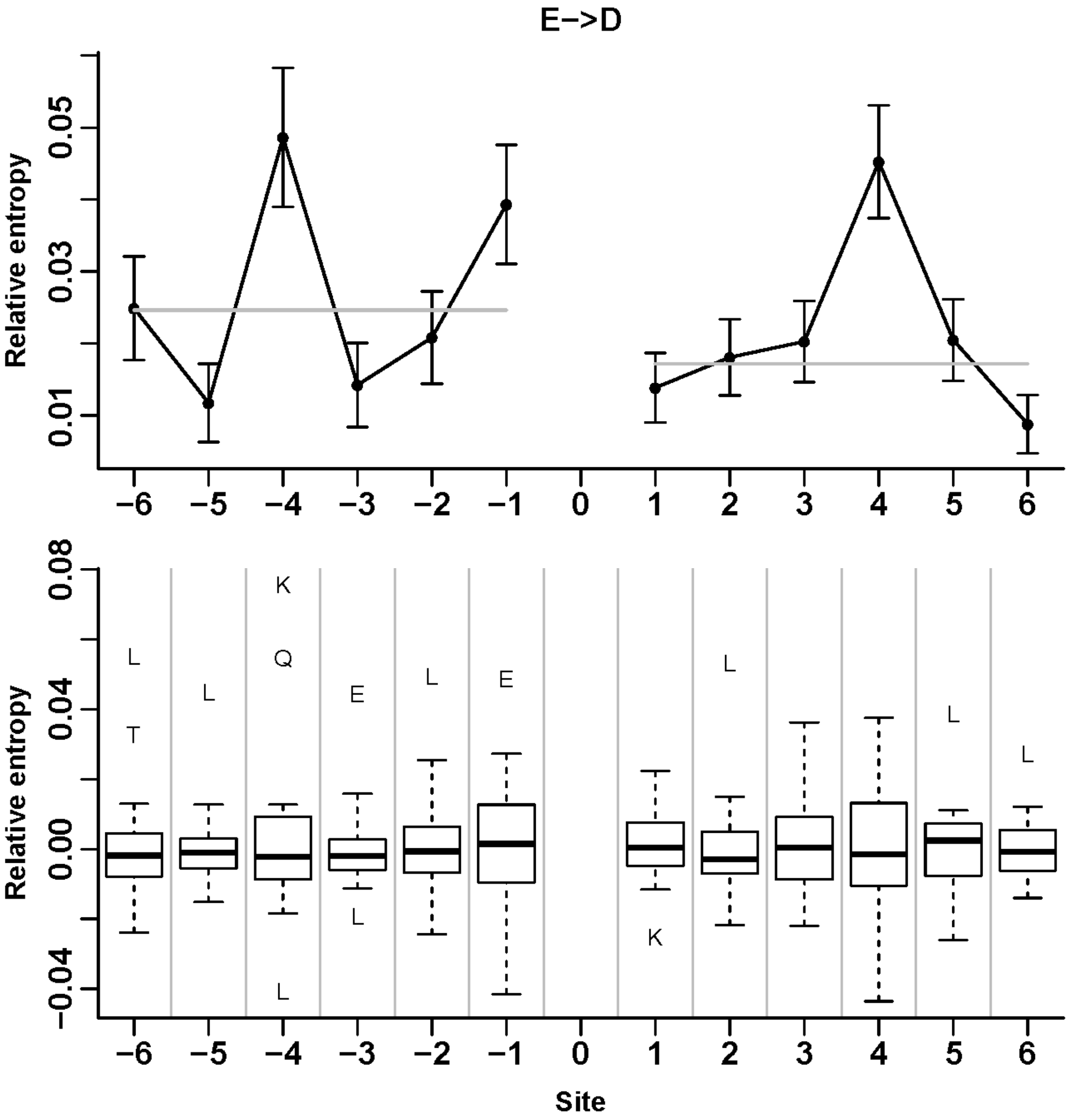
E−>D
Relative entropy
0.05
0.03
0.01
−6 −5 −4 −3 −2 −1 0 1 2 3 4 5 6
0.08
0.04
0.00
−0.04
L
T
L
K
Q
L
E
L
L
E
L
K
L
L
L
Site

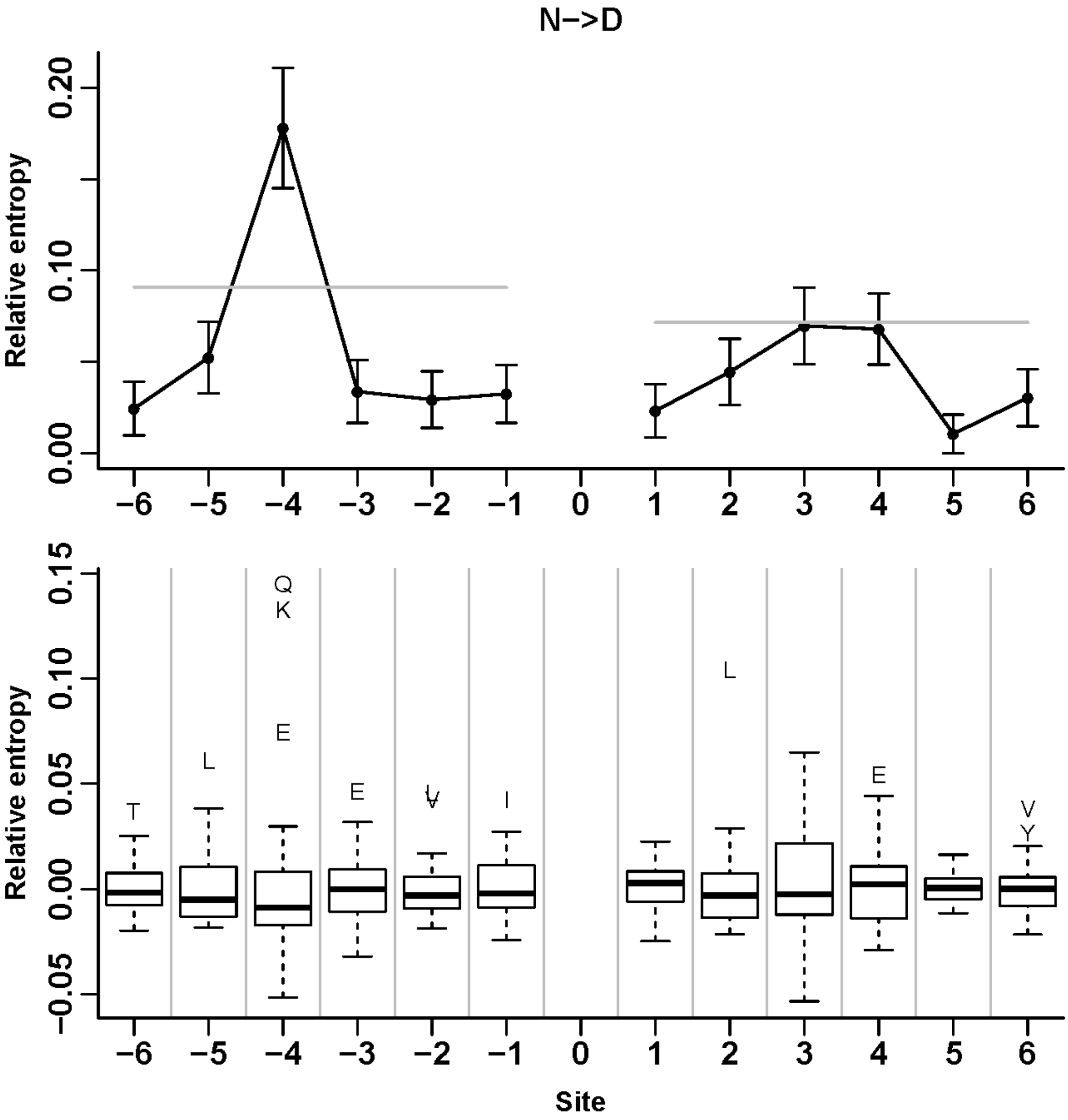
N−>D
Relative entropy
0.00
0.10
0.20
−6
−5
−4
−3
−2
−1
0
1
2
3
4
5
6
Relative entropy
−0.05
0.00
0.05
0.10
0.15
T
L
Q
K
E
E
L
V
I
L
E
V
Y
Site

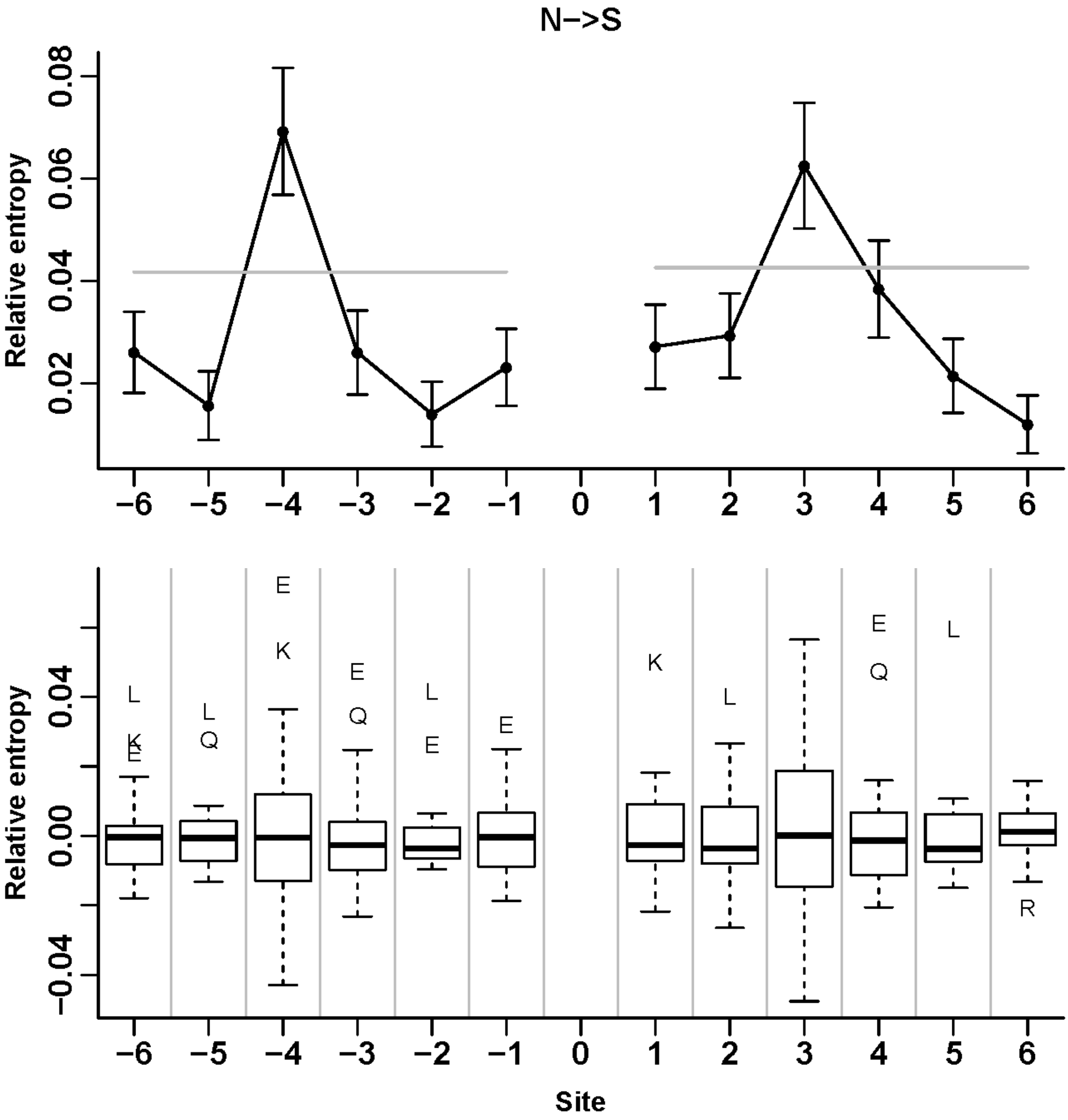
N−>S
Relative entropy
0.08
0.06
0.04
0.02
−6 −5 −4 −3 −2 −1 0 1 2 3 4 5 6
Relative entropy
0.04
0.00
−0.04
L K E
L Q
E K
E Q
L E
E
K
L
E Q
L
R
Site

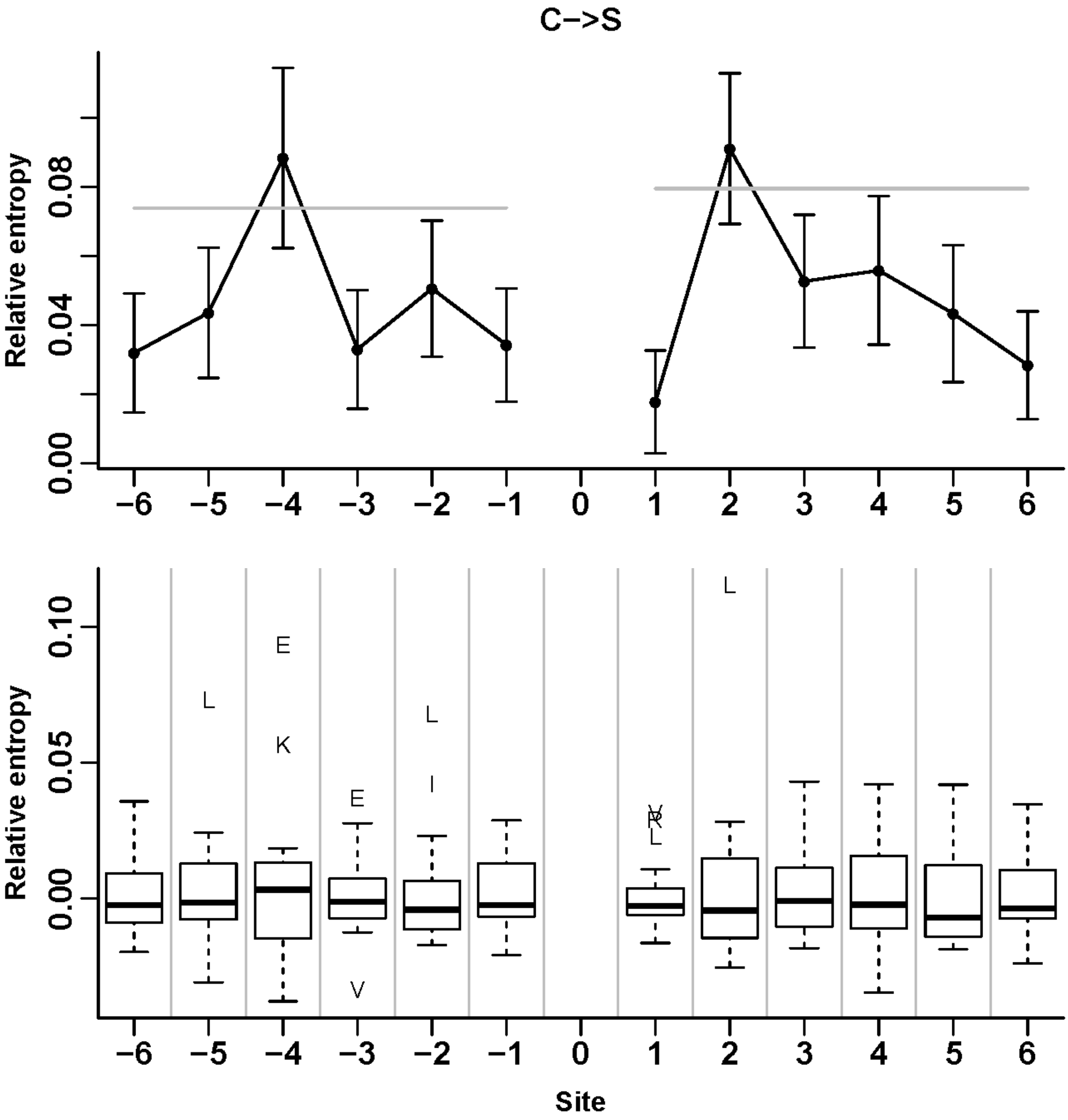
C−>S
Relative entropy
0.00
0.04
0.08
−6
−5
−4
−3
−2
−1
0
1
2
3
4
5
6
Relative entropy
0.00
0.05
0.10
L
E
K
L
E
I
V
V
R
L
L
Site

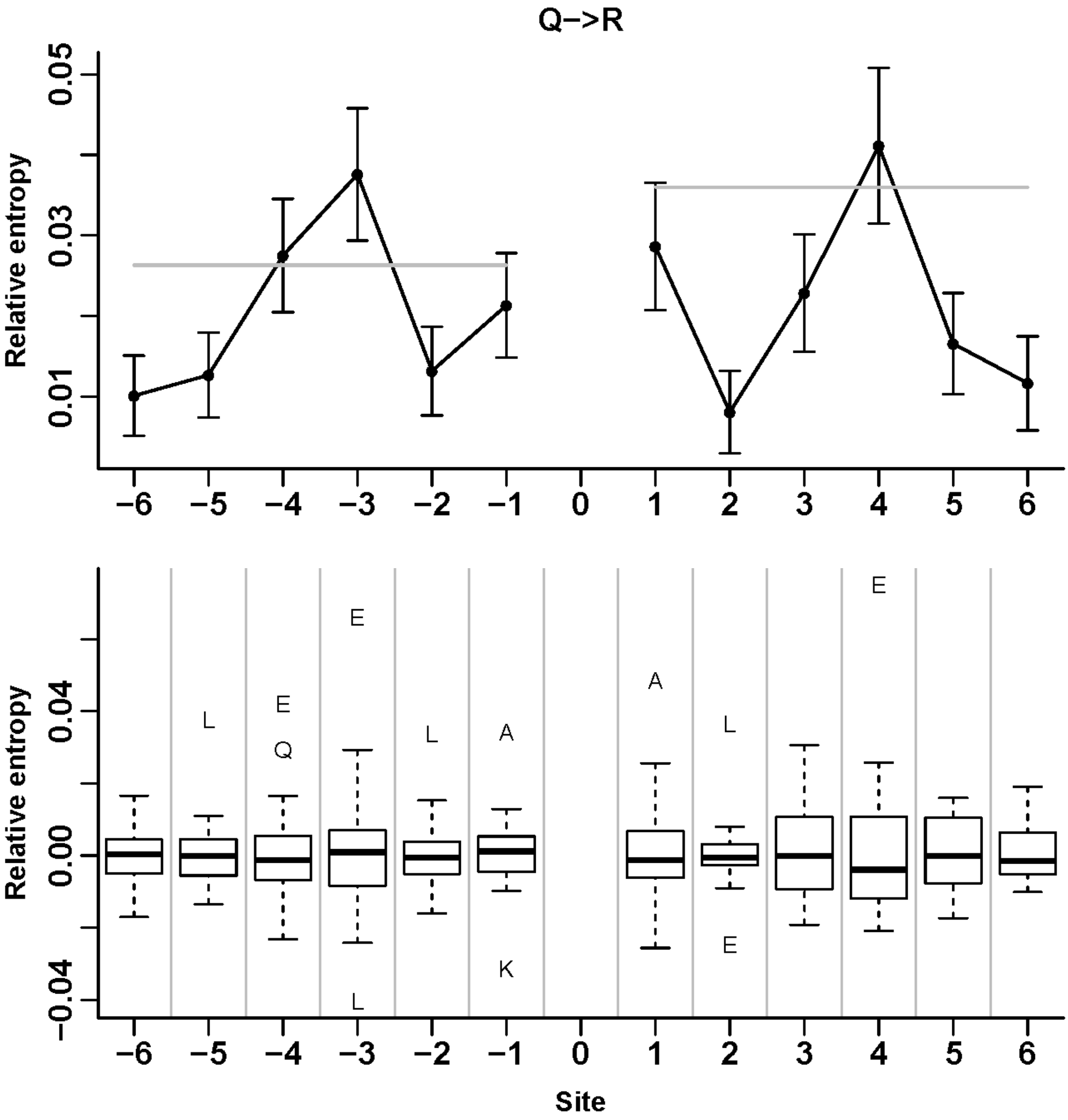
Q−>R
Relative entropy
0.01
0.03
0.05
−6
−5
−4
−3
−2
−1
0
1
2
3
4
5
6
Relative entropy
−0.04
0.00
0.04
E
L
E
Q
L
A
K
L
A
L
E
E
Site

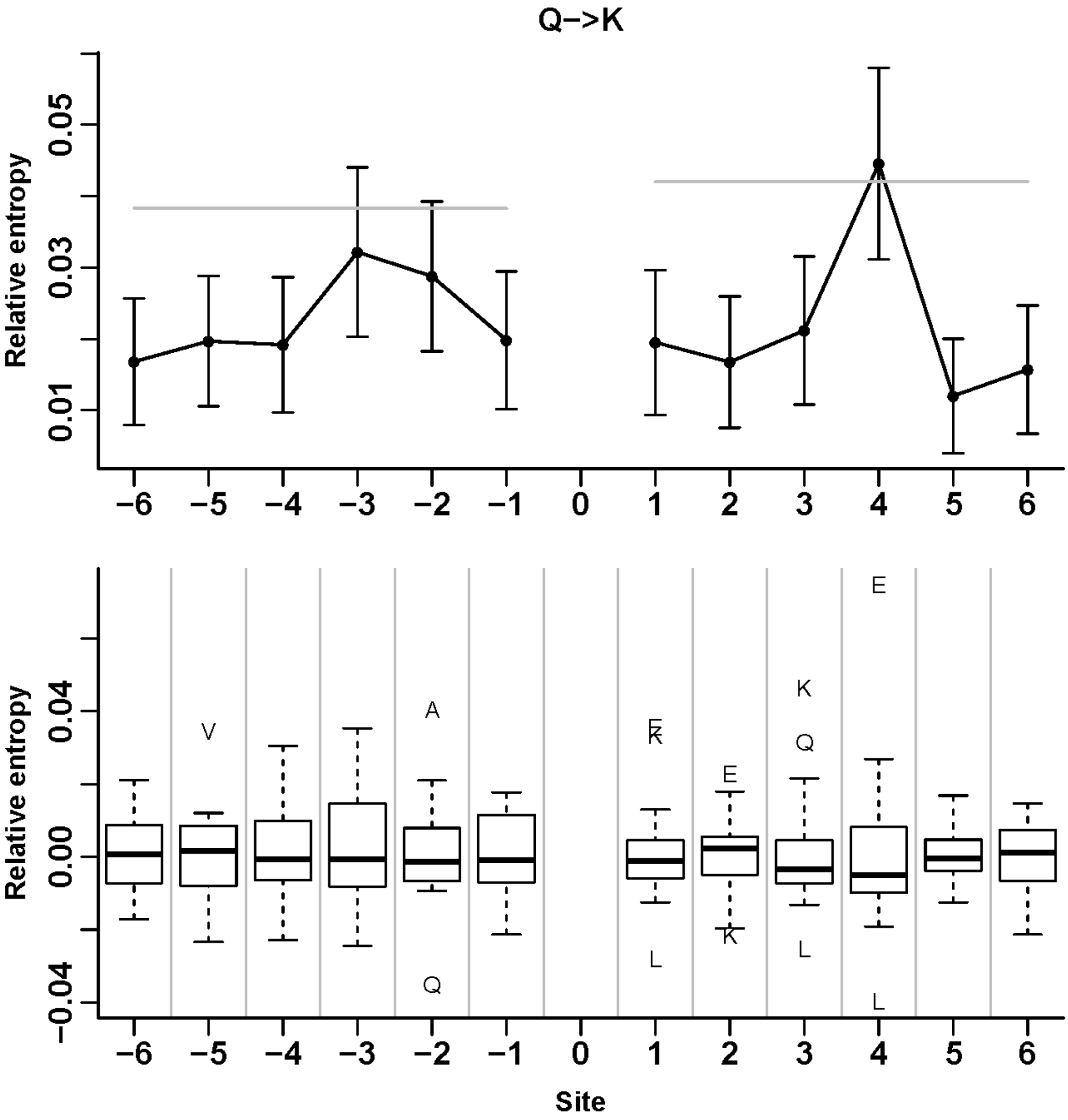
Q−>K
Relative entropy
0.01
0.03
0.05
−6
−5
−4
−3
−2
−1
0
1
2
3
4
5
6
Relative entropy
−0.04
0.00
0.04
V
A
Q
E
K
L
E
K
K
Q
L
E
L
Site

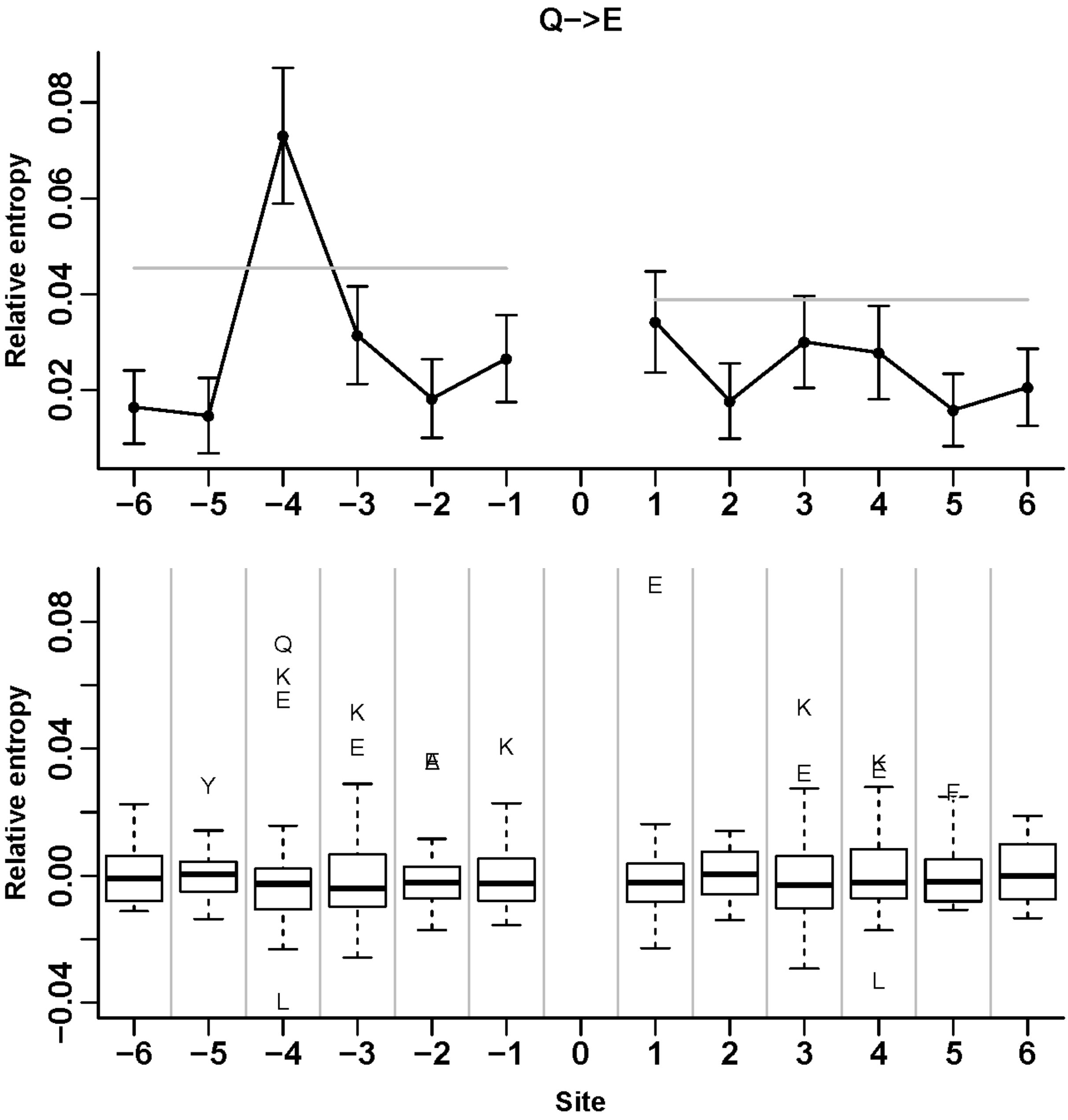

Q−>E
Relative entropy
0.02
0.04
0.06
0.08
−6 −5 −4 −3 −2 −1 0 1 2 3 4 5 6
Relative entropy
−0.04
0.00
0.04
0.08
Q
K
E
L
Y
K
E
K
E
K
E
K
E
L
E
Site

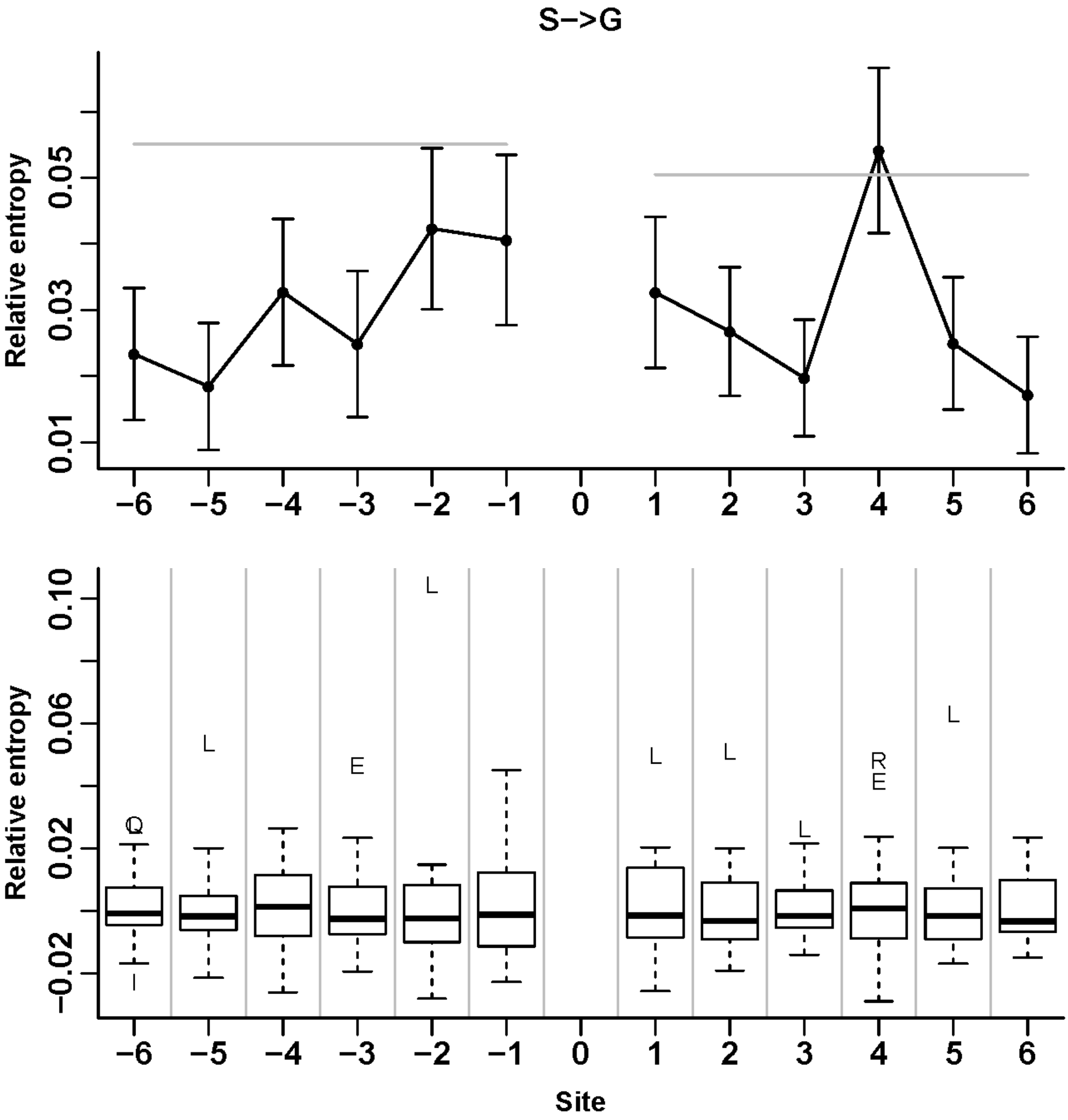
S−>G
Relative entropy
0.01
0.03
0.05
−6
−5
−4
−3
−2
−1
0
1
2
3
4
5
6
Relative entropy
−0.02
0.02
0.06
0.10
Q
I
L
E
L
L
L
L
R
E
L
Site

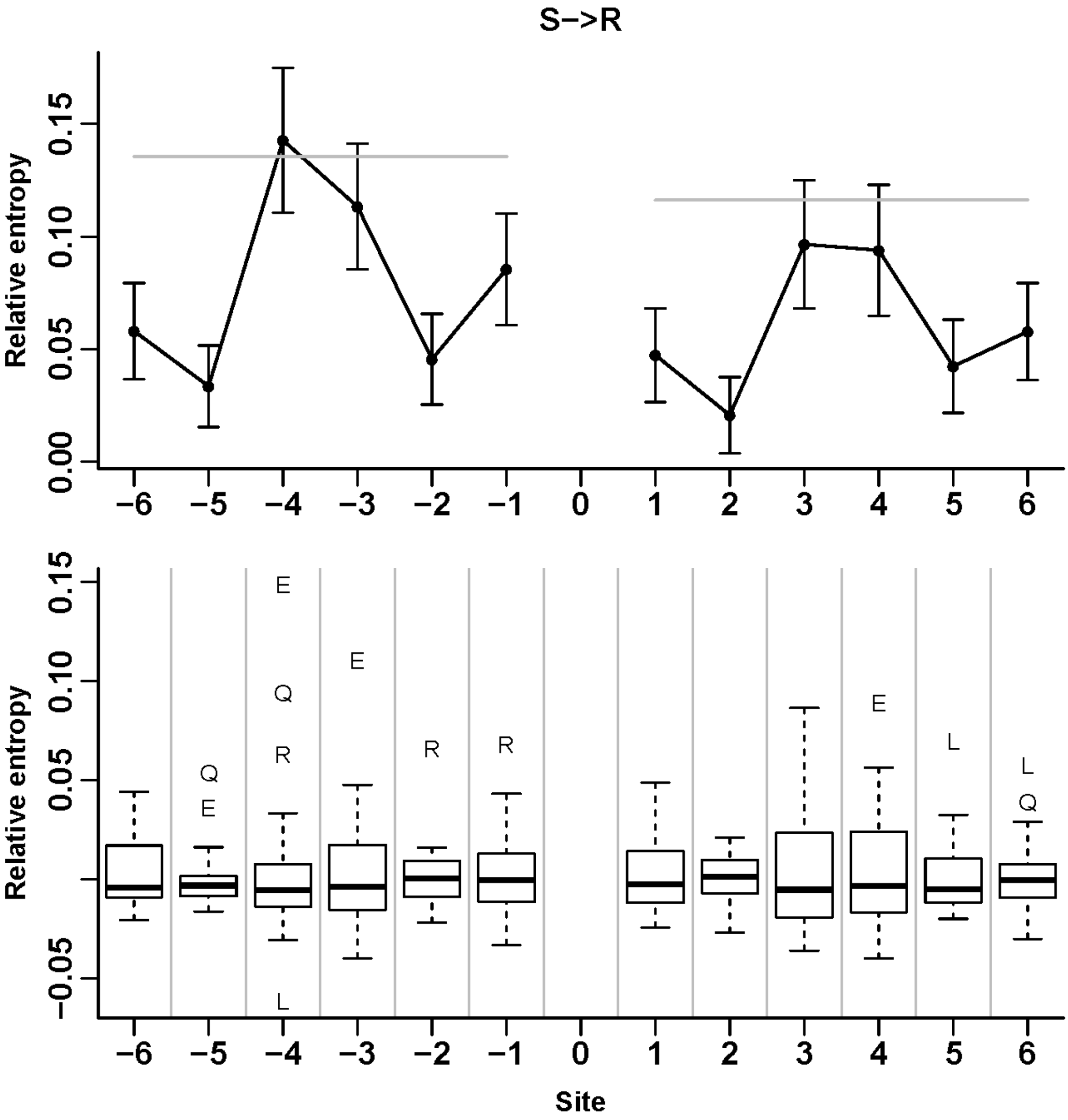
S−>R
Relative entropy
0.00
0.05
0.10
0.15
−6
−5
−4
−3
−2
−1
0
1
2
3
4
5
6
Relative entropy
−0.05
0.05
0.10
0.15
E
Q
R
L
Q
E
R
R
E
L
L
Q
Site

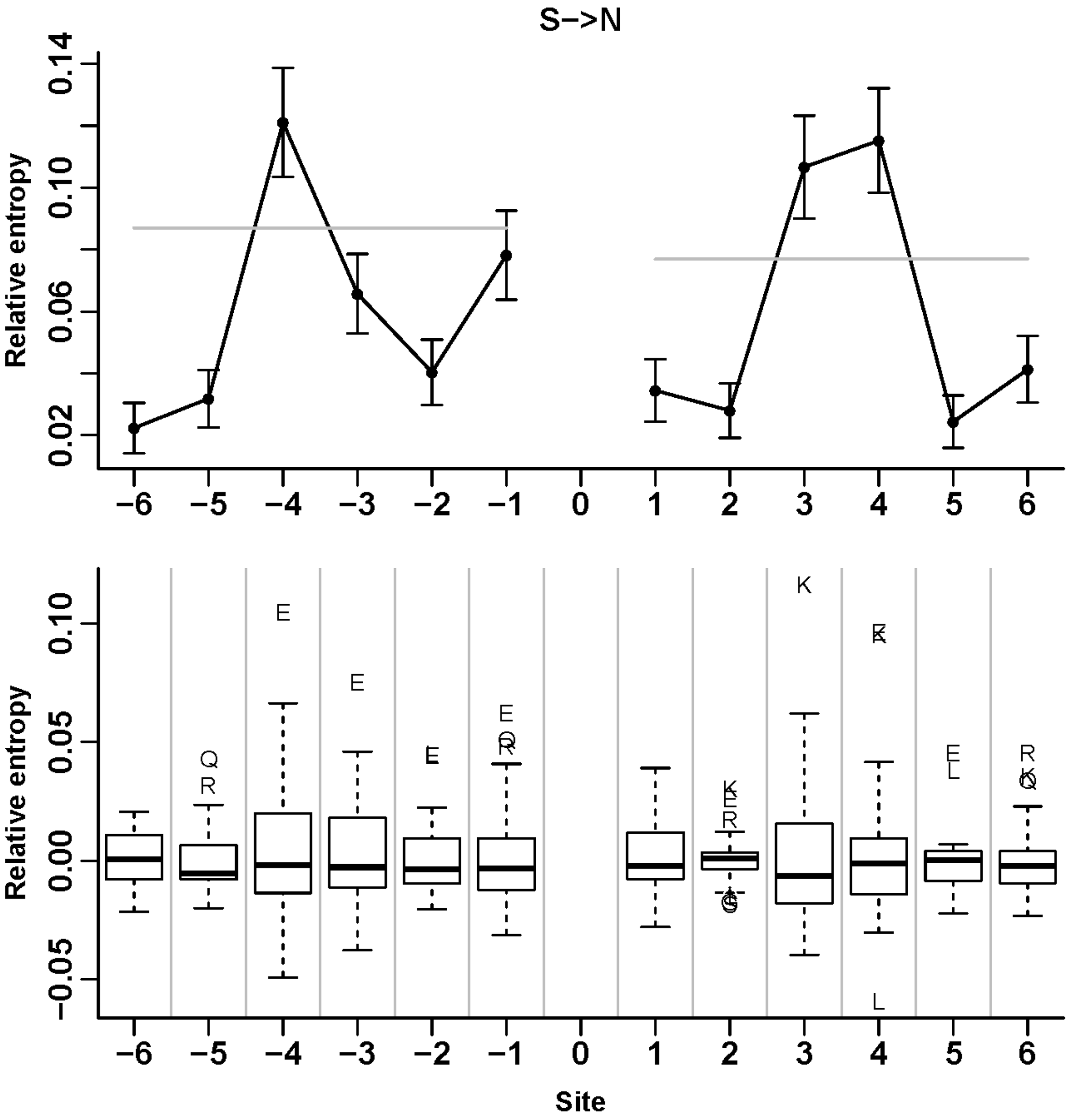

S−>N
Relative entropy
0.02
0.06
0.10
0.14
−6
−5
−4
−3
−2
−1
0
1
2
3
4
5
6
Relative entropy
−0.05
0.00
0.05
0.10
Site

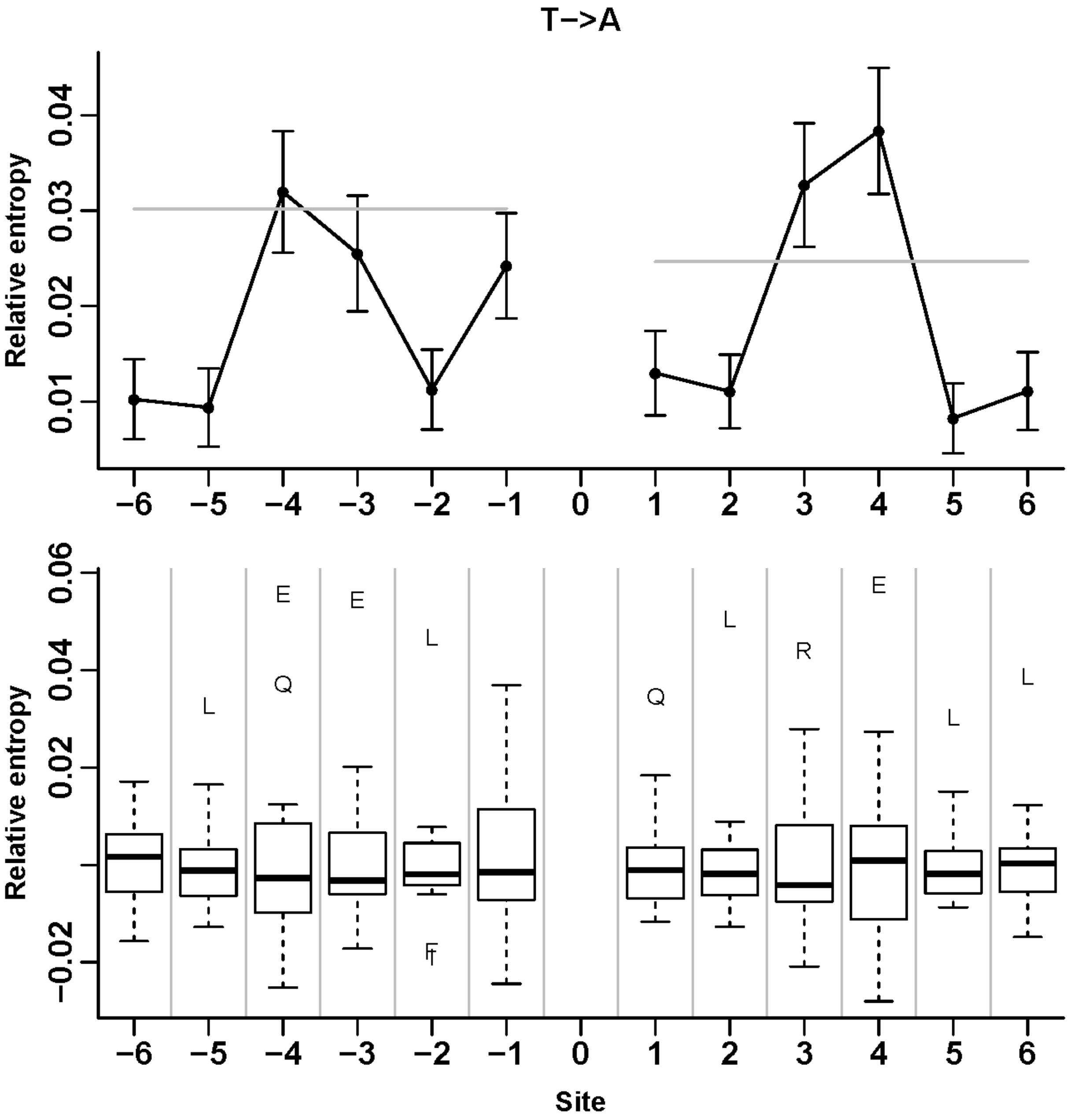
T−>A
Relative entropy
0.01
0.02
0.03
0.04
−6
−5
−4
−3
−2
−1
0
1
2
3
4
5
6
Relative entropy
−0.02
0.02
0.04
0.06
E
E
L
E
L
Q
L
R
Q
L
L
L
Site

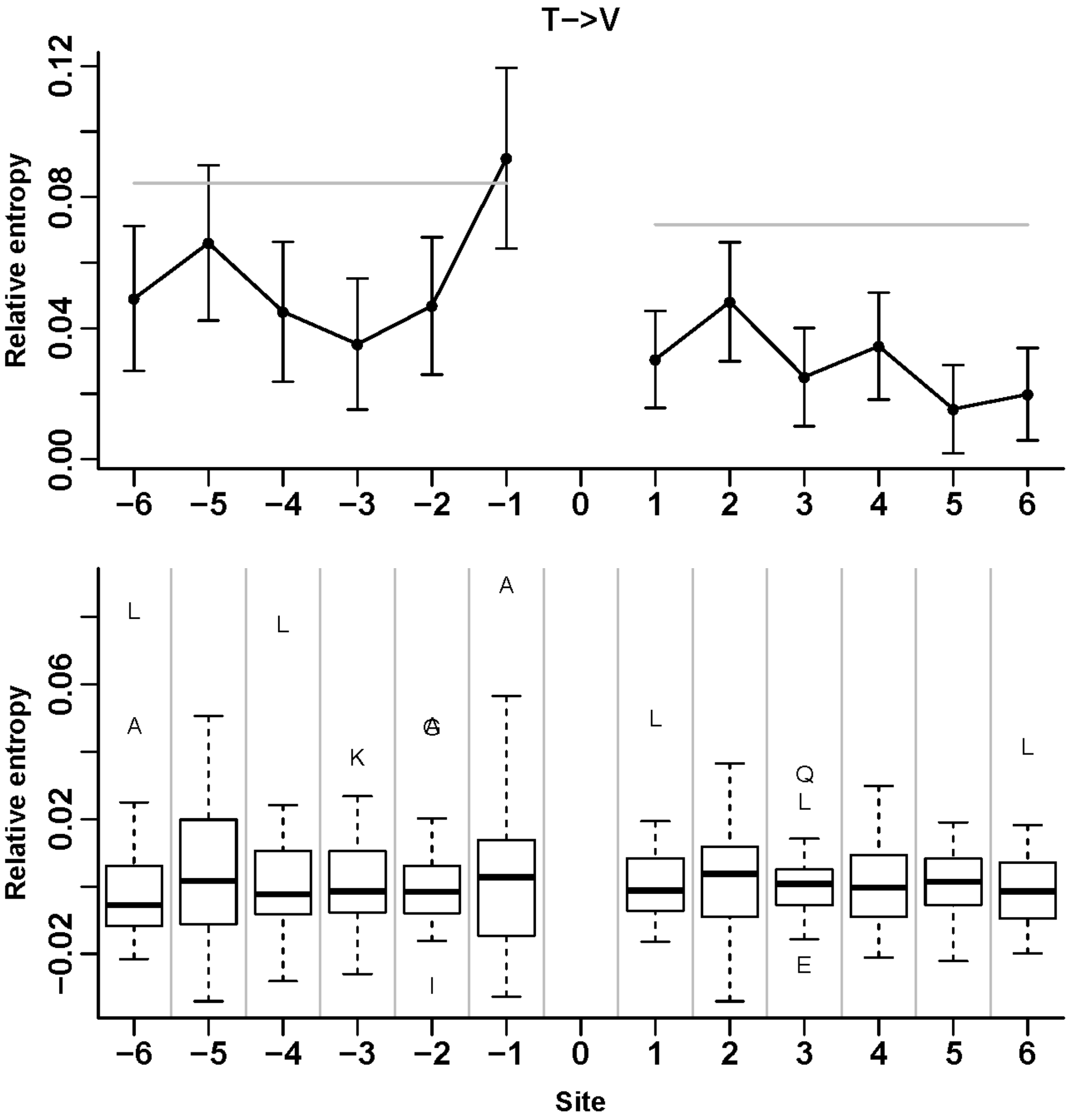
T−>V
Relative entropy
0.00
0.04
0.08
0.12
−6
−5
−4
−3
−2
−1
0
1
2
3
4
5
6
Relative entropy
−0.02
0.02
0.06
L
A
L
K
A
G
I
A
L
Q
L
E
L
Site

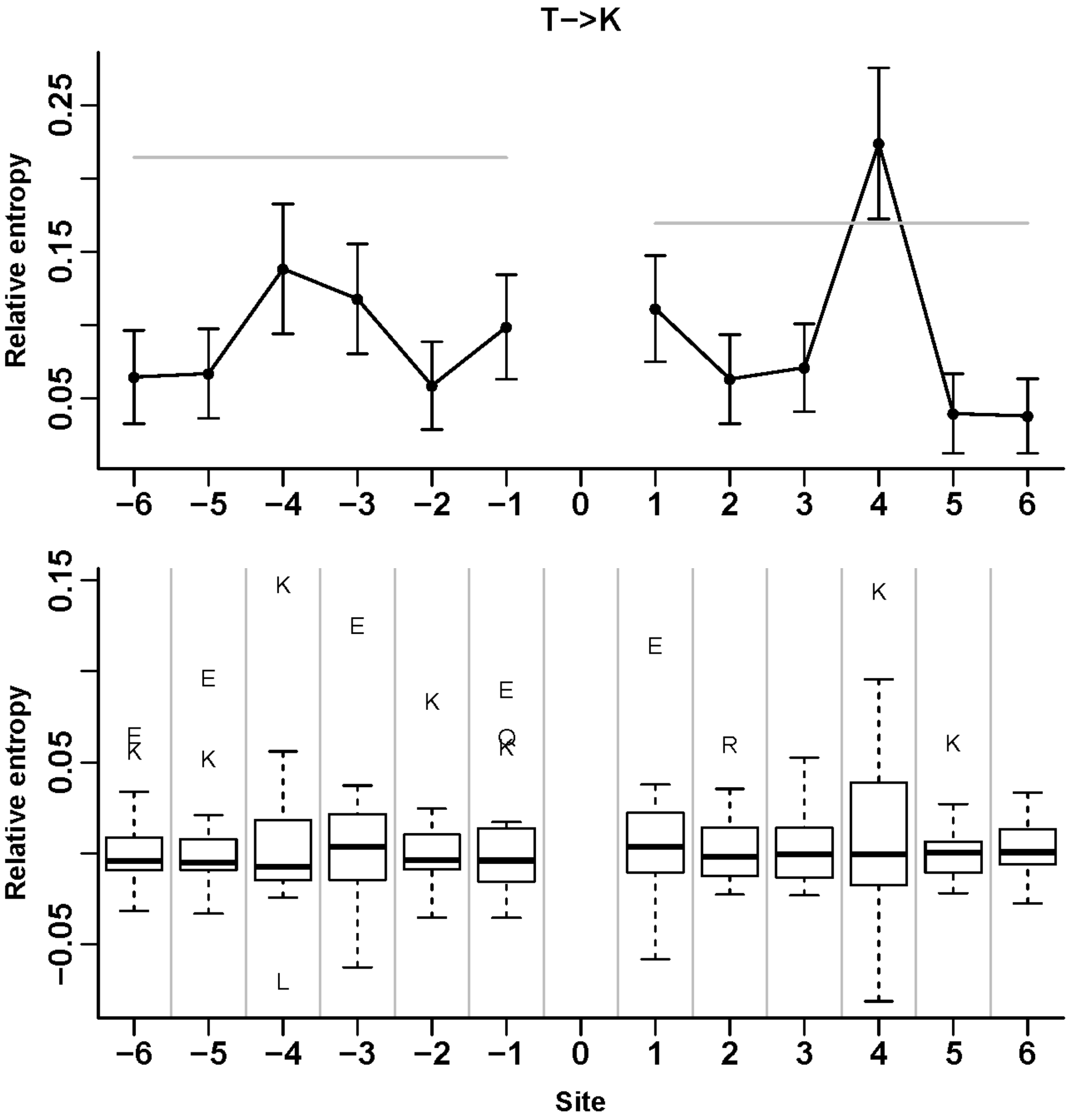

T−>K
Relative entropy
0.05
0.15
0.25
−6
−5
−4
−3
−2
−1
0
1
2
3
4
5
6
Relative entropy
−0.05
0.05
0.15
E
K
E
K
K
L
E
K
E
Q
K
E
R
K
K
Site

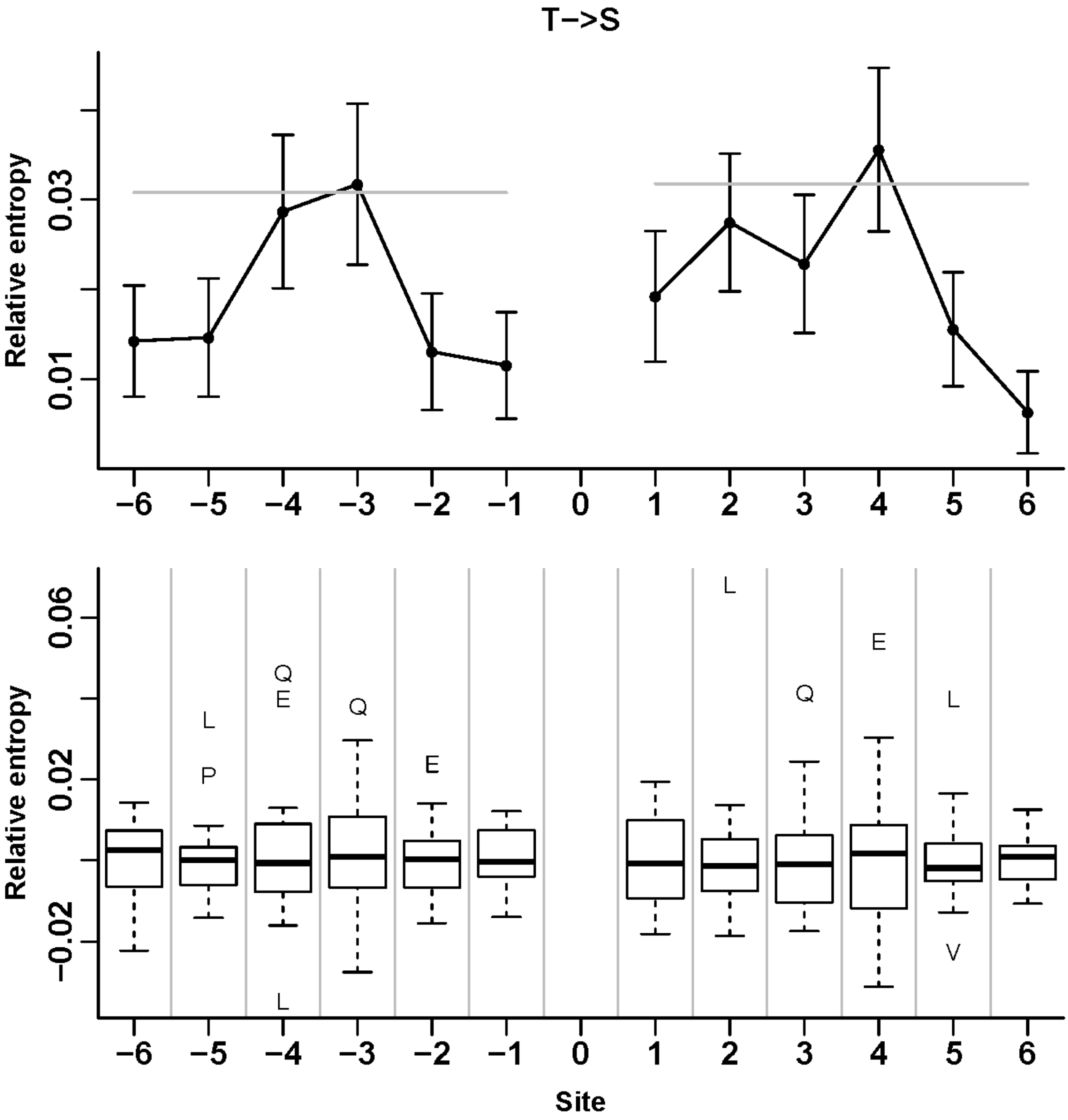
T−>S
Relative entropy
0.01
0.03
−6
−5
−4
−3
−2
−1
0
1
2
3
4
5
6
Relative entropy
−0.02
0.02
0.06
L
P
Q
E
L
Q
E
L
Q
E
L
V
Site

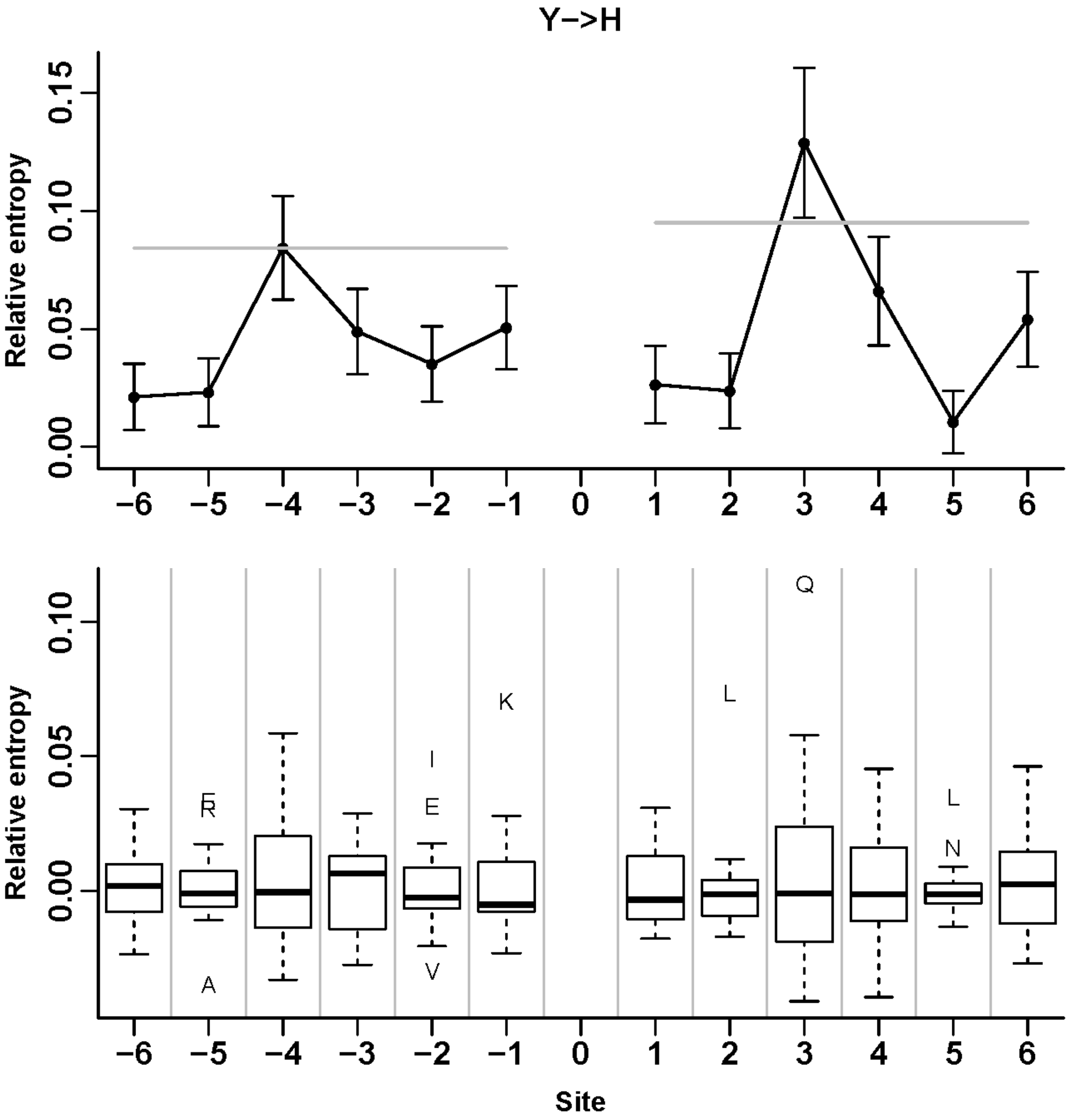
Y−>H
Relative entropy
0.00
0.05
0.10
0.15
−6
−5
−4
−3
−2
−1
0
1
2
3
4
5
6
Q
L
K
I
F
R
E
L
N
A
V
Site

Figure S7. Context-dependence patterns of the amino acid substitutions in coil.

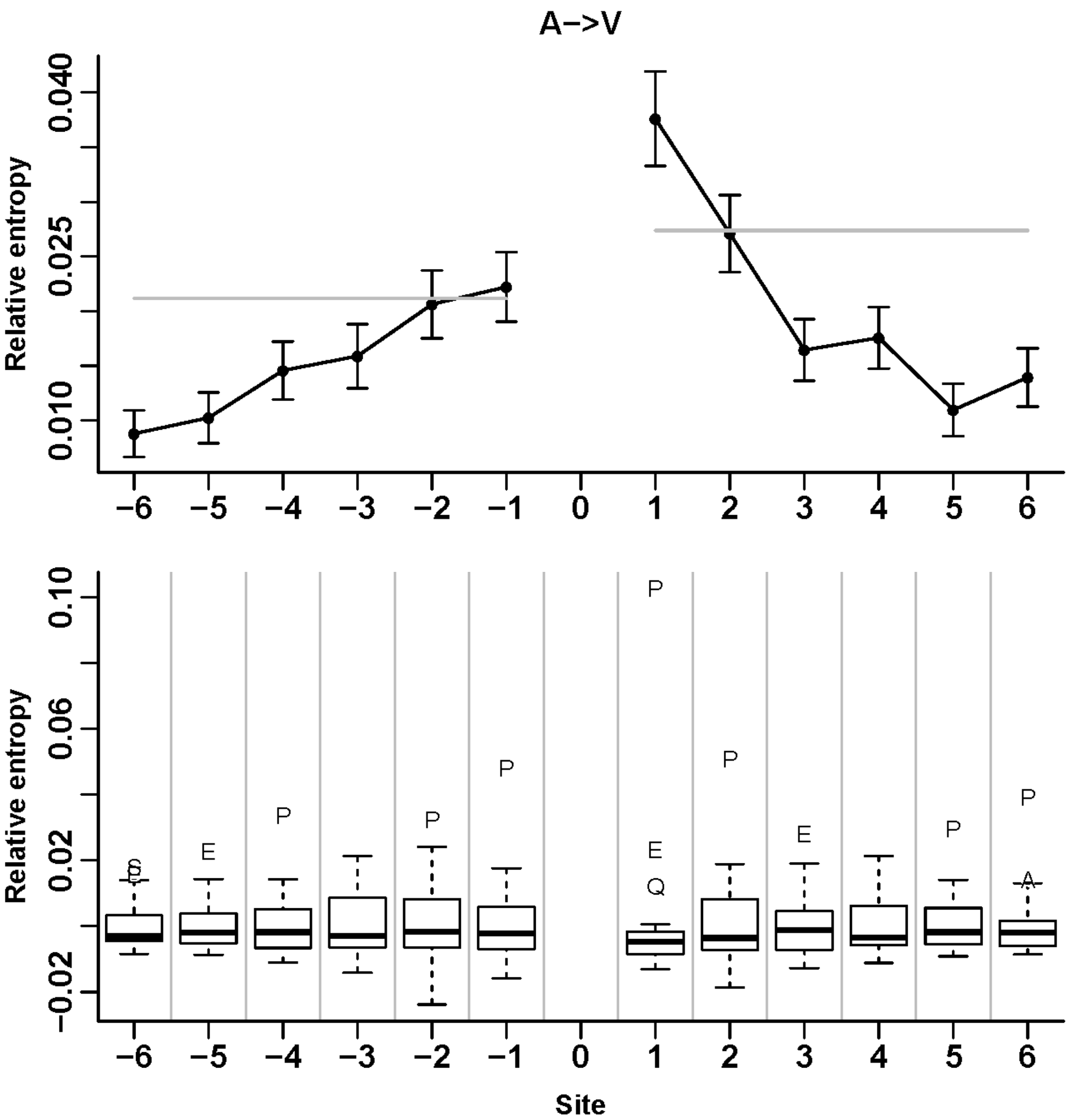

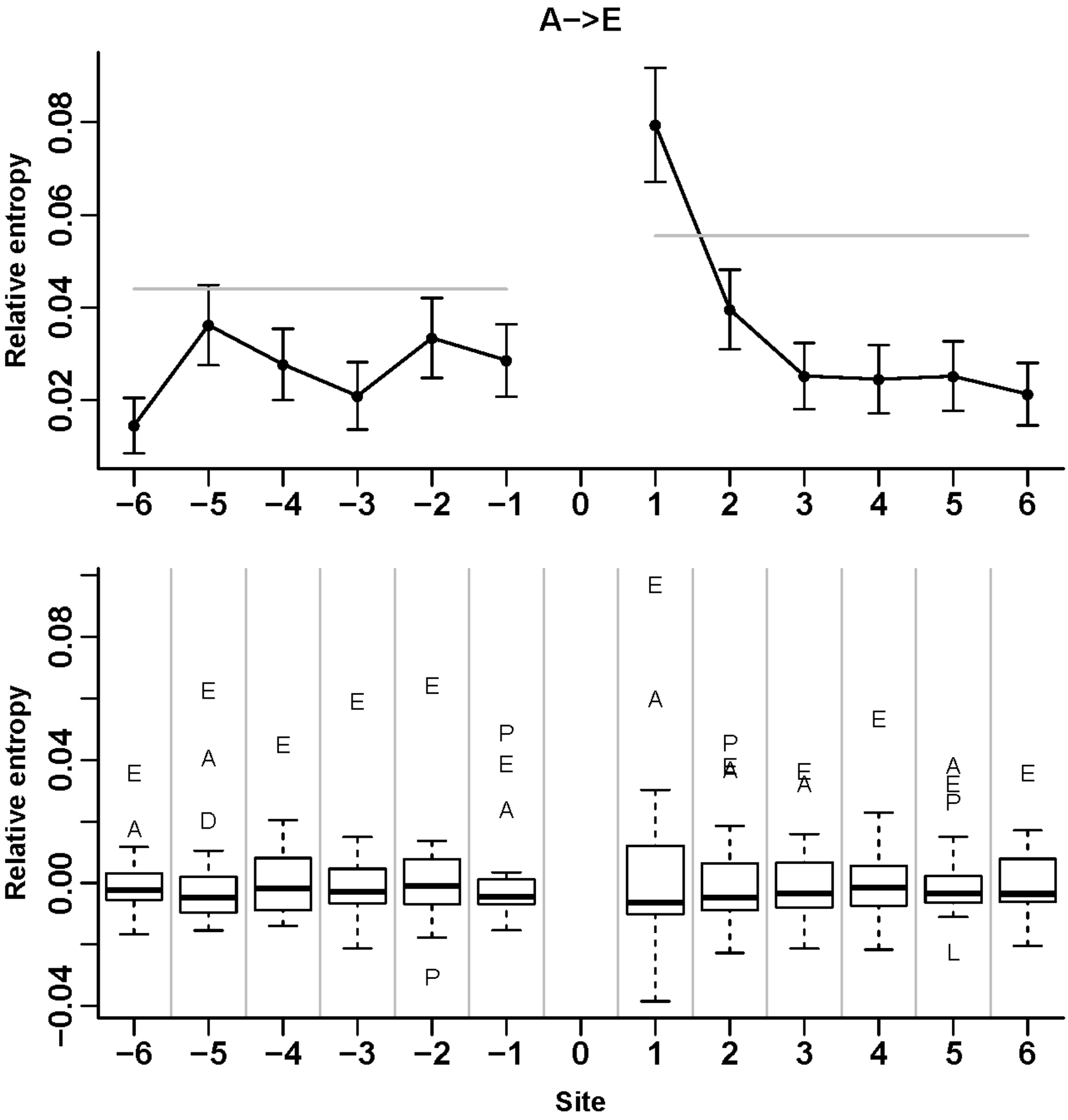

A−>E
Relative entropy
0.02
0.04
0.06
0.08
−6
−5
−4
−3
−2
−1
0
1
2
3
4
5
6
Relative entropy
−0.04
0.00
0.04
0.08
Site

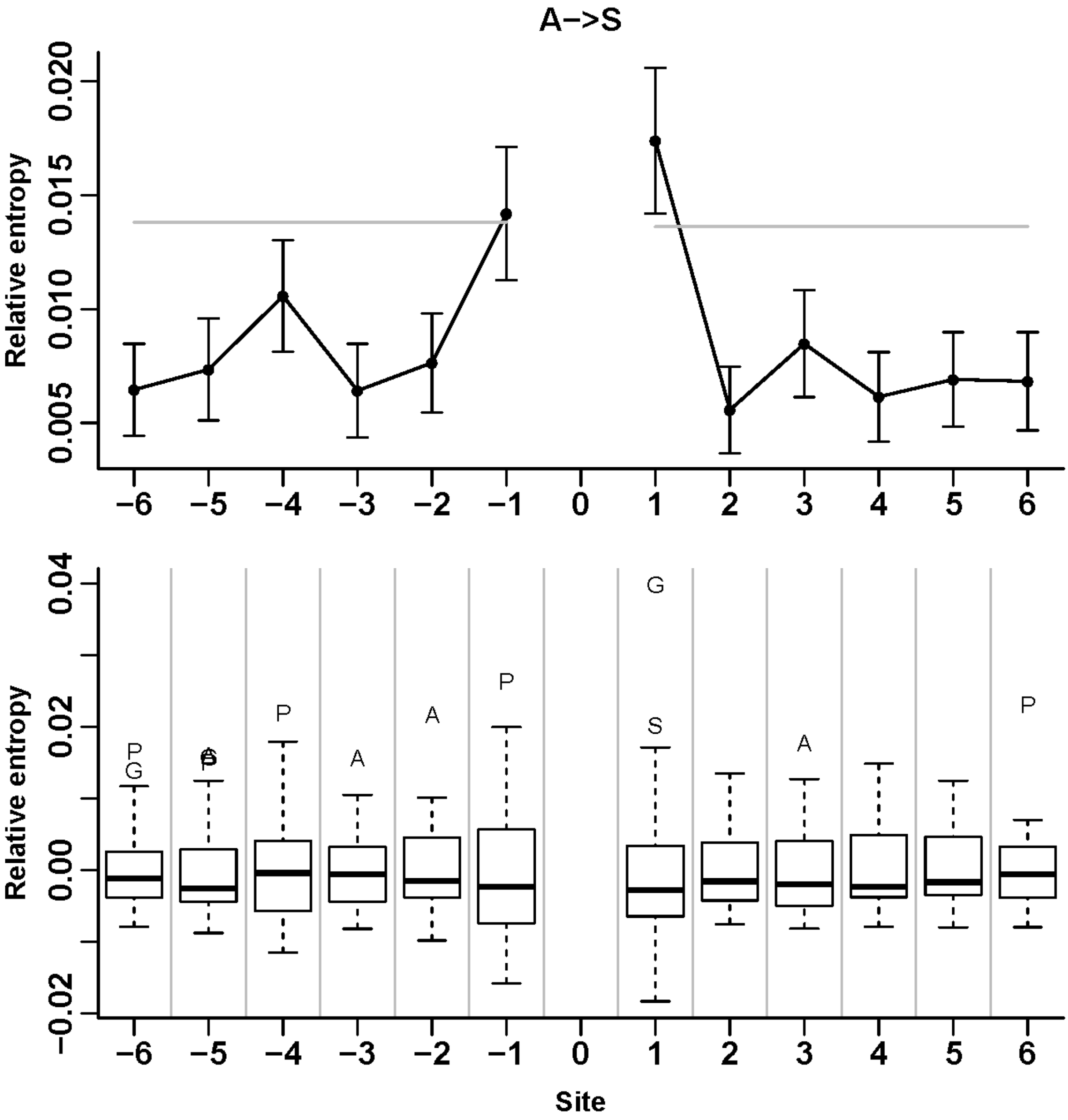
A−>S
Relative entropy
0.005
0.010
0.015
0.020
−6
−5
−4
−3
−2
−1
0
1
2
3
4
5
6
Relative entropy
−0.02
0.00
0.02
0.04
P
G
[illegible]
P
A
A
P
G
S
A
P
Site

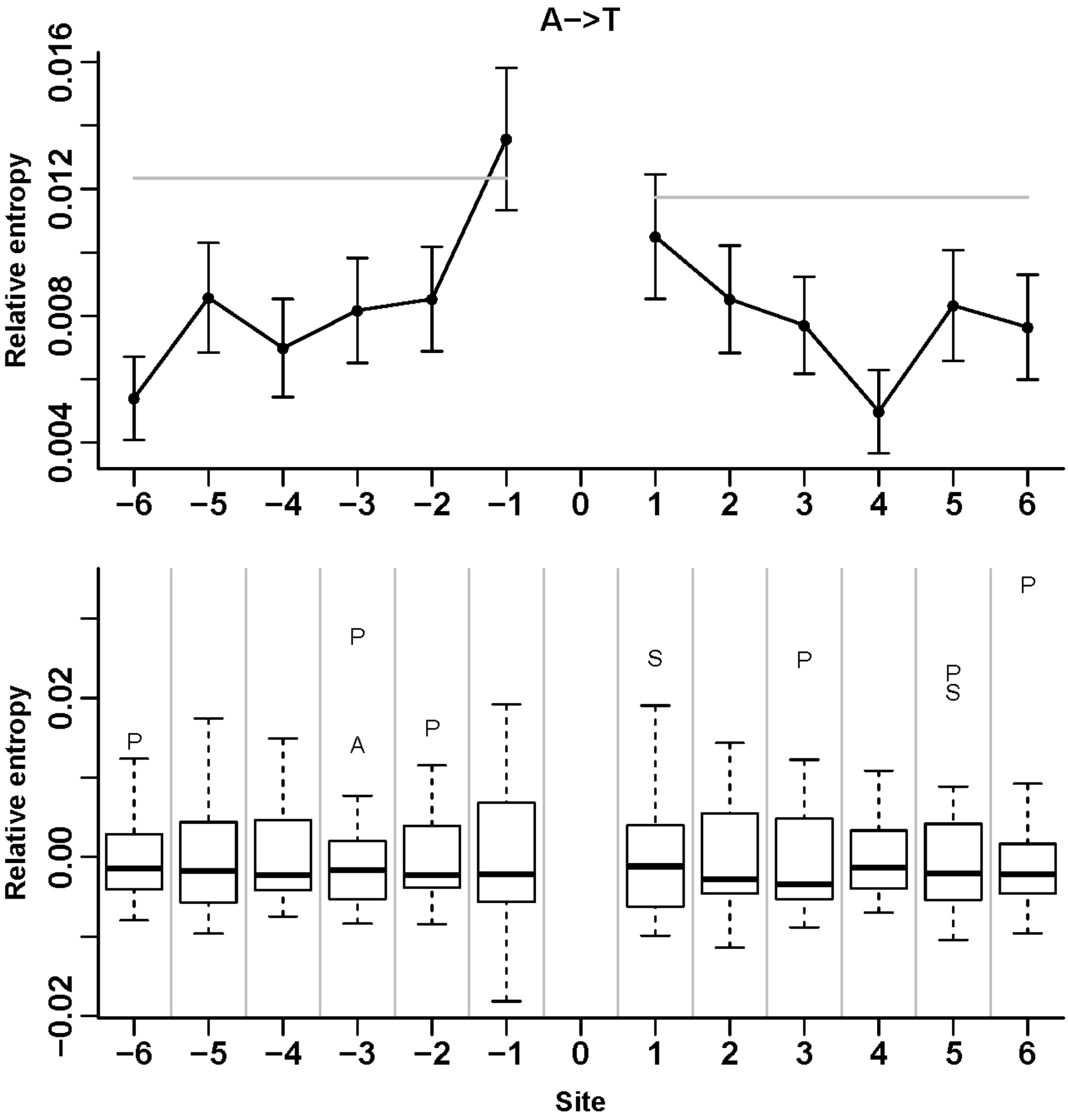
A->T
Relative entropy
0.004
0.008
0.012
0.016
−6
−5
−4
−3
−2
−1
0
1
2
3
4
5
6
Relative entropy
−0.02
0.00
0.02
P
P
A
P
S
P
P
S
P
Site

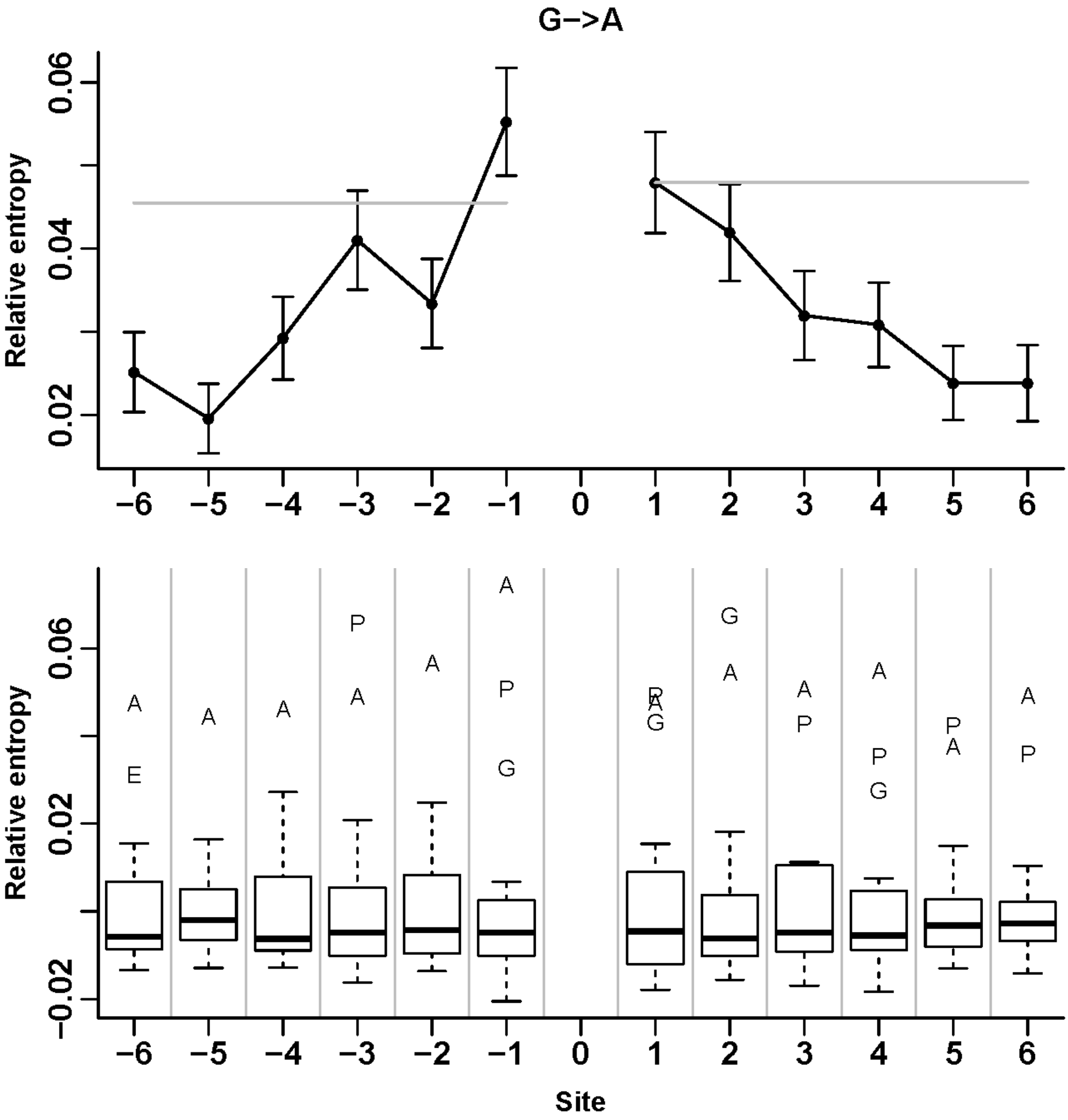
G−>A
Relative entropy
0.02
0.04
0.06
−6
−5
−4
−3
−2
−1
0
1
2
3
4
5
6
Relative entropy
−0.02
0.02
0.06
A
E
A
A
P
A
A
A
P
G
P
A
G
G
A
A
P
A
P
G
P
A
A
P
Site

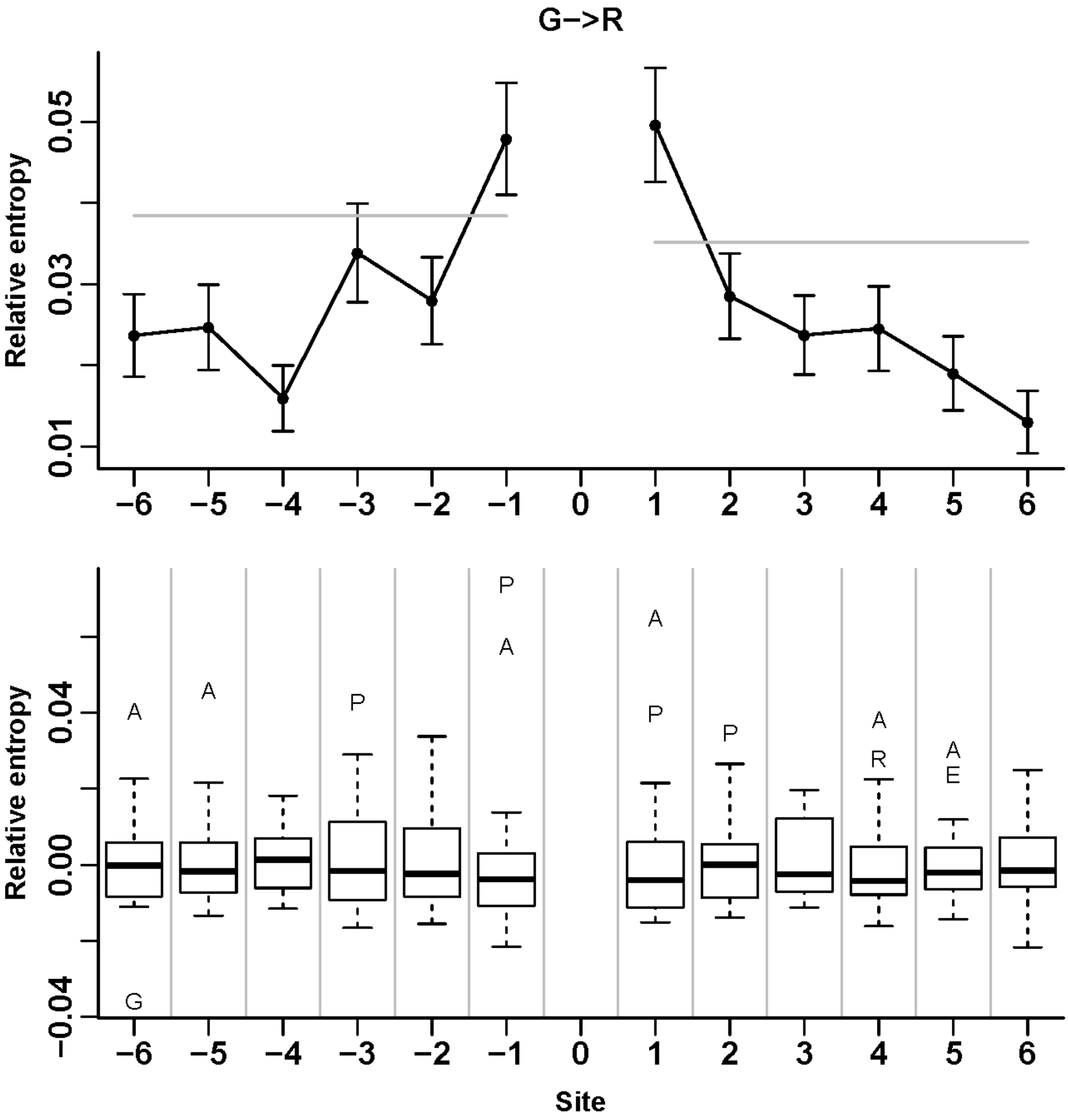
G−>R
Relative entropy
0.01
0.03
0.05
−6
−5
−4
−3
−2
−1
0
1
2
3
4
5
6
Relative entropy
−0.04
0.00
0.04
A
A
P
P
A
A
P
P
A
R
A
E
G
Site

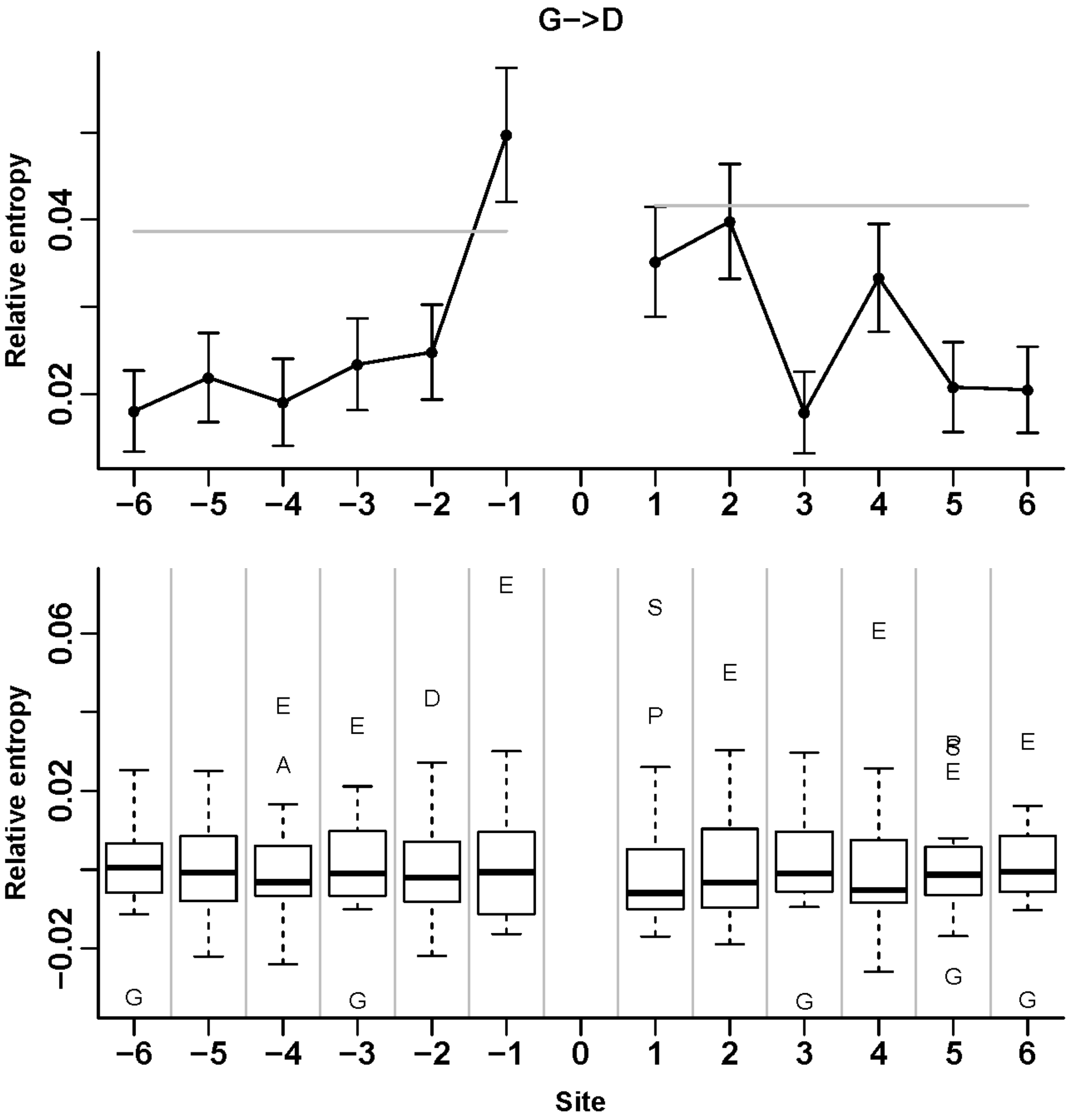
G−>D
Relative entropy
0.02
0.04
−6 −5 −4 −3 −2 −1 0 1 2 3 4 5 6
Relative entropy
−0.02
0.02
0.06
Site

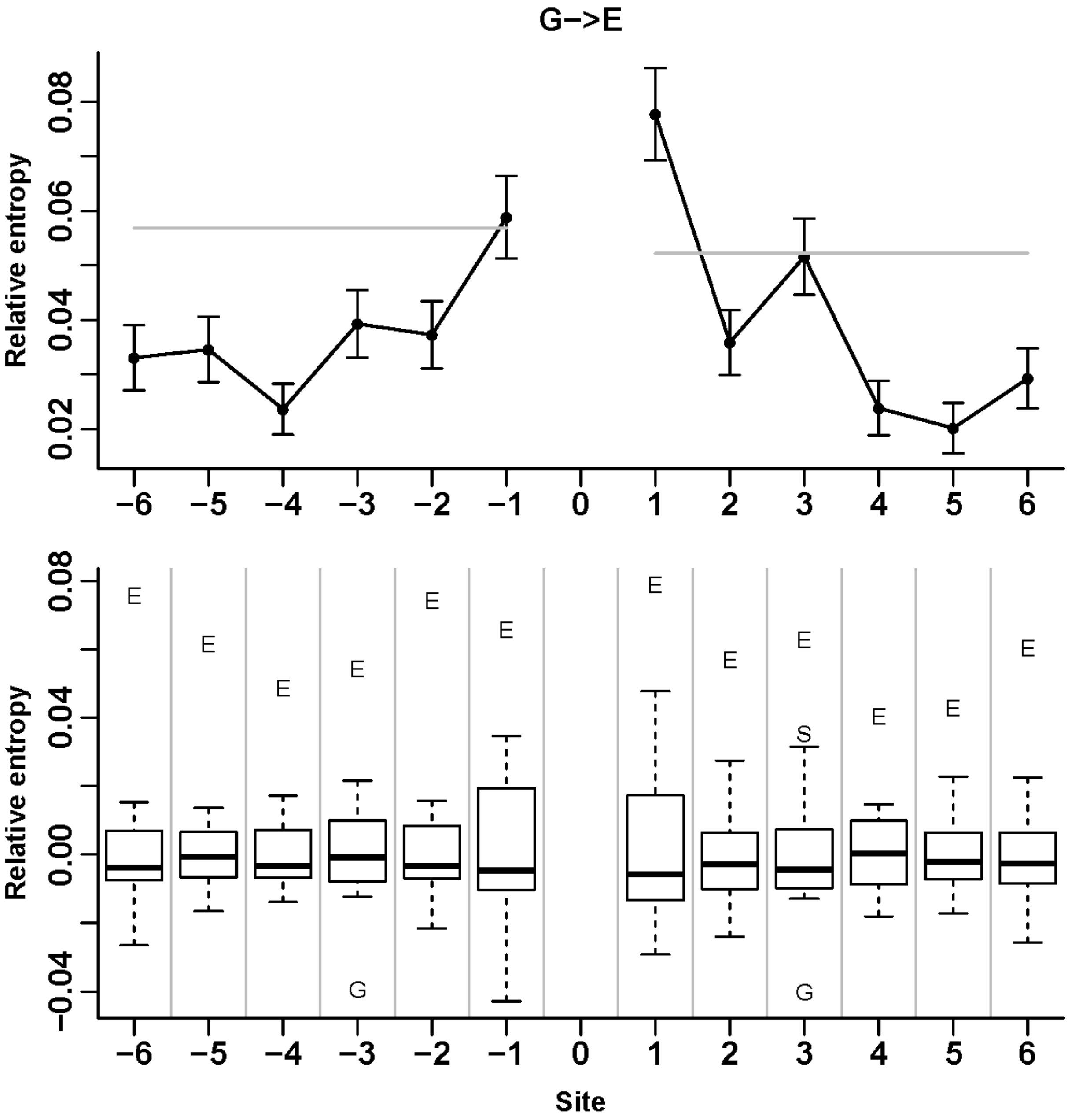
G−>E
Relative entropy
0.02
0.04
0.06
0.08
−6
−5
−4
−3
−2
−1
0
1
2
3
4
5
6
Relative entropy
−0.04
0.00
0.04
0.08
E
S
G
Site

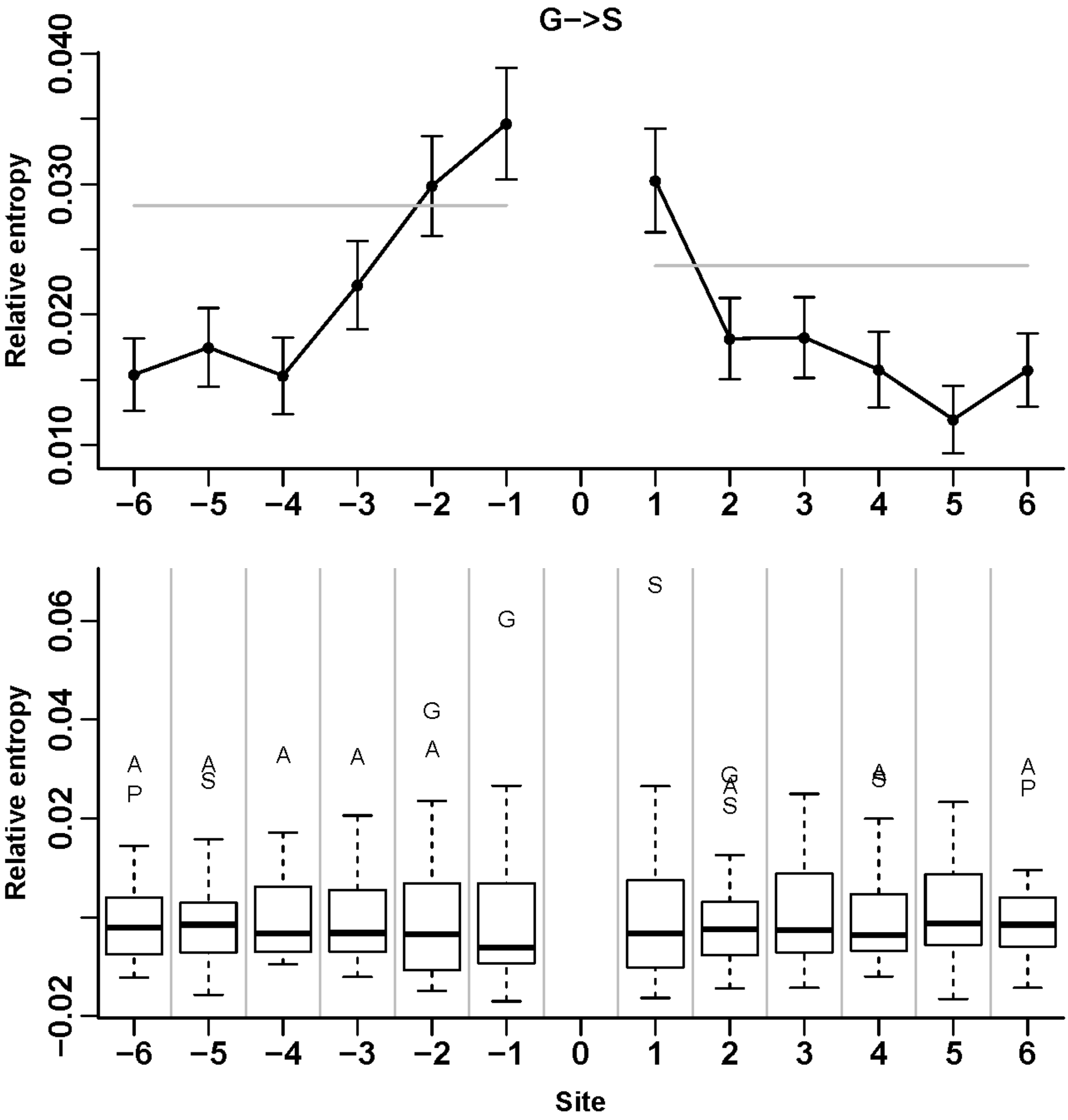
G->S
Relative entropy
0.010
0.020
0.030
0.040
−6 −5 −4 −3 −2 −1 0 1 2 3 4 5 6
Relative entropy
−0.02
0.02
0.04
0.06
Site

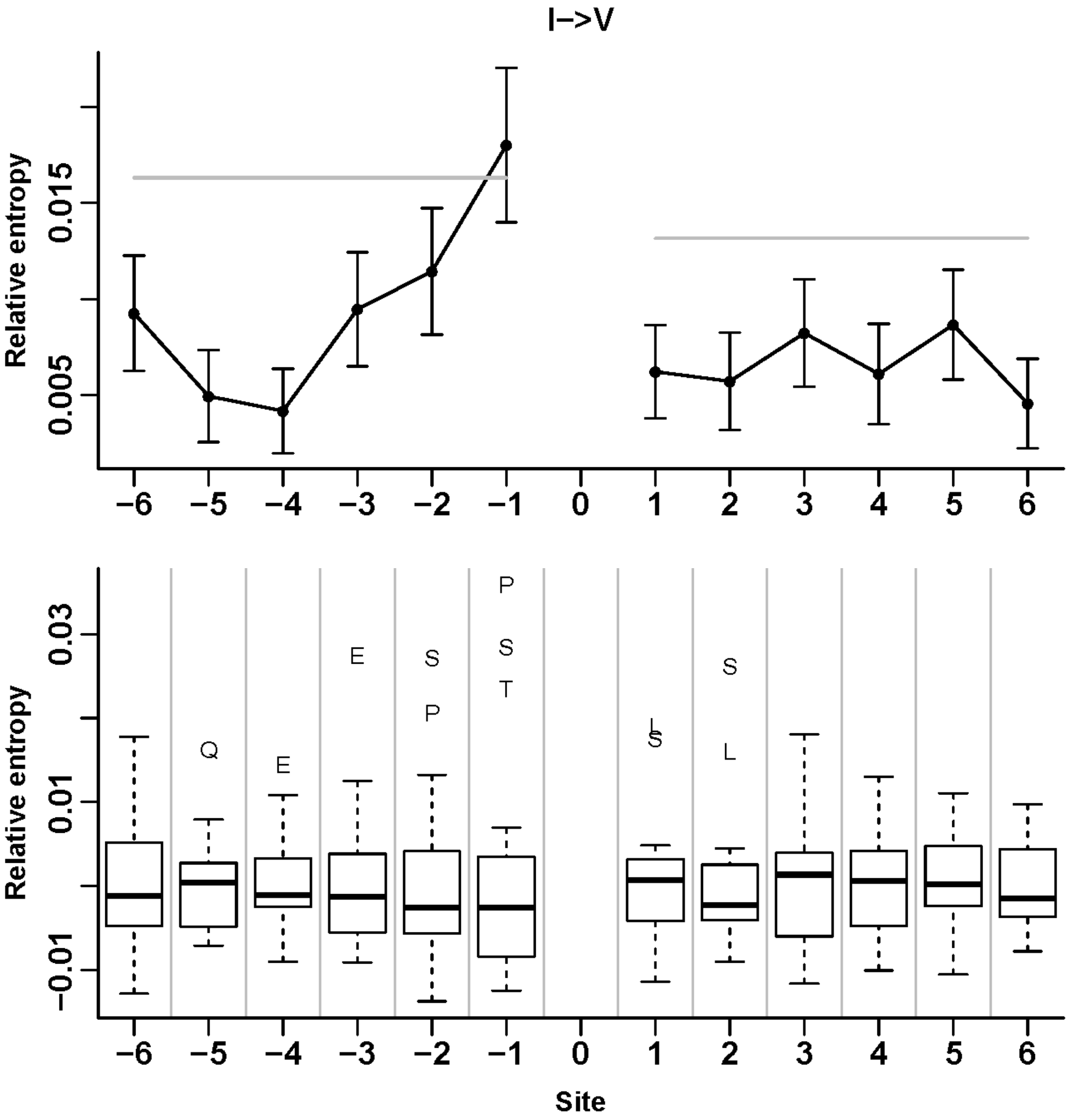
I−>V
Relative entropy
0.005
0.015
−6
−5
−4
−3
−2
−1
0
1
2
3
4
5
6
Relative entropy
−0.01
0.01
0.03
Q
E
E
S
P
P
S
T
L
S
S
L
Site

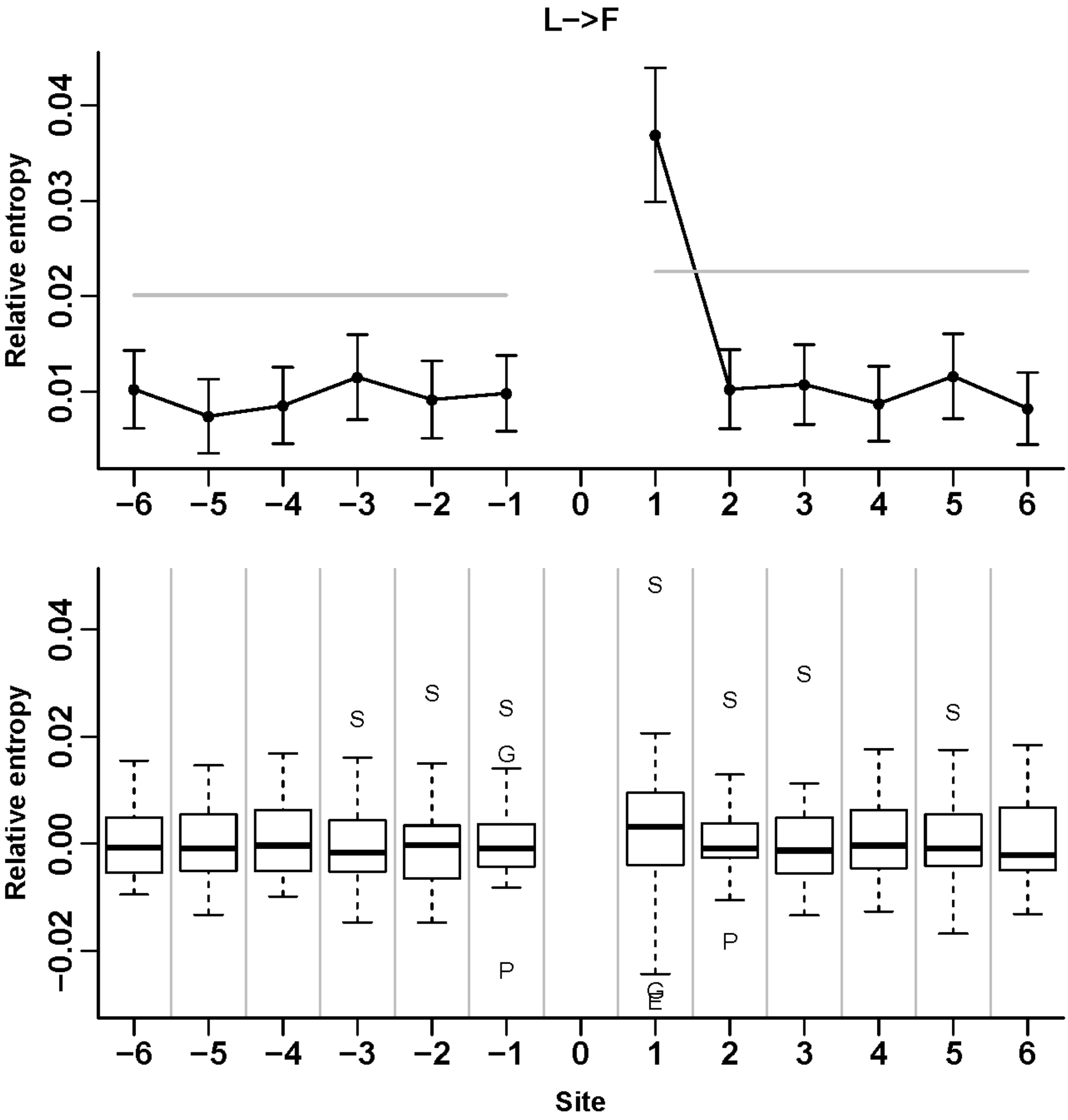
L−>F
Relative entropy
0.01
0.02
0.03
0.04
−6
−5
−4
−3
−2
−1
0
1
2
3
4
5
6
Relative entropy
−0.02
0.00
0.02
0.04
S
G
P
E
Site

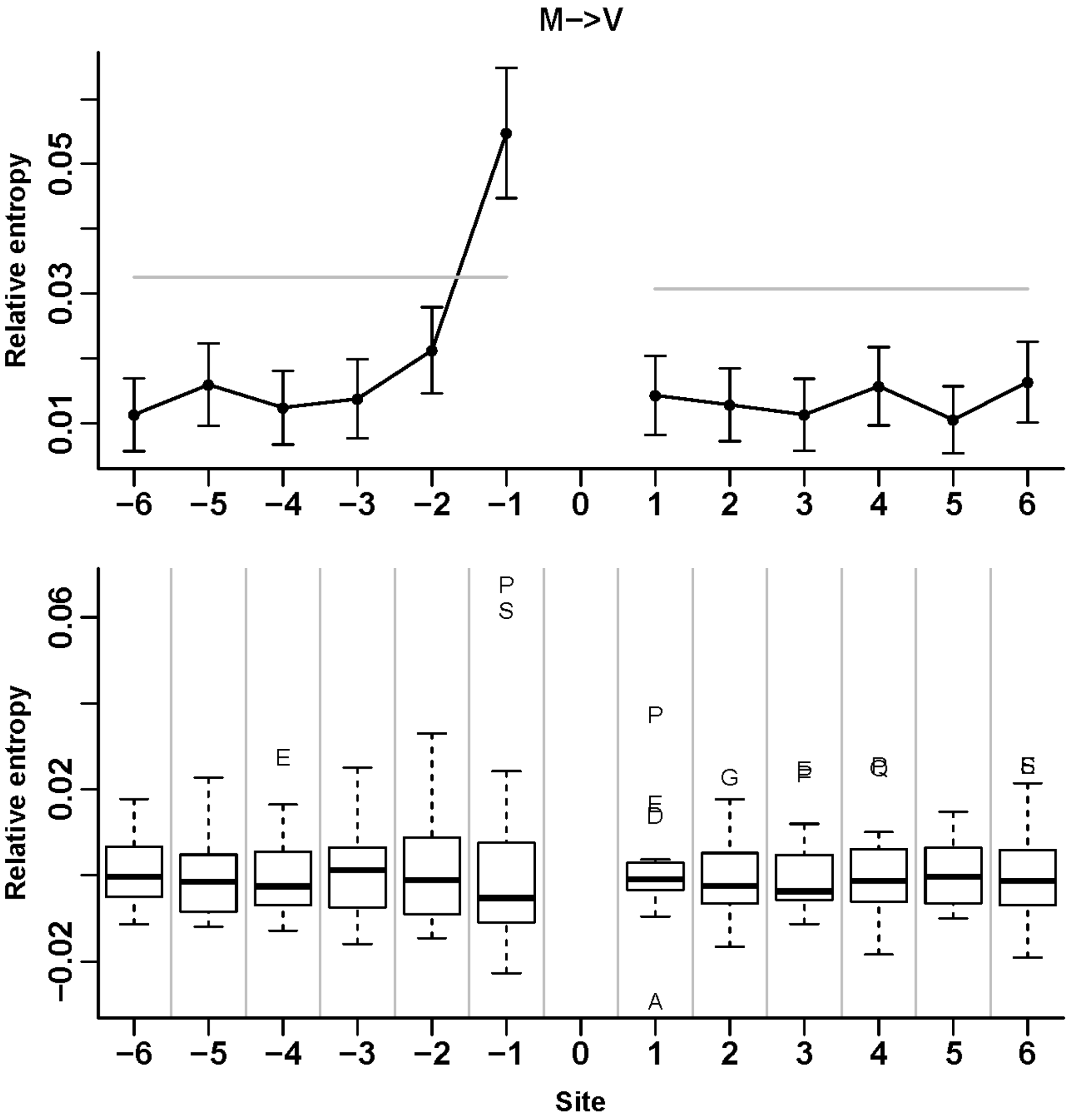
M−>V
Relative entropy
0.01
0.03
0.05
−6
−5
−4
−3
−2
−1
0
1
2
3
4
5
6
Relative entropy
−0.02
0.02
0.06
P
S
E
P
E
D
G
A
Site

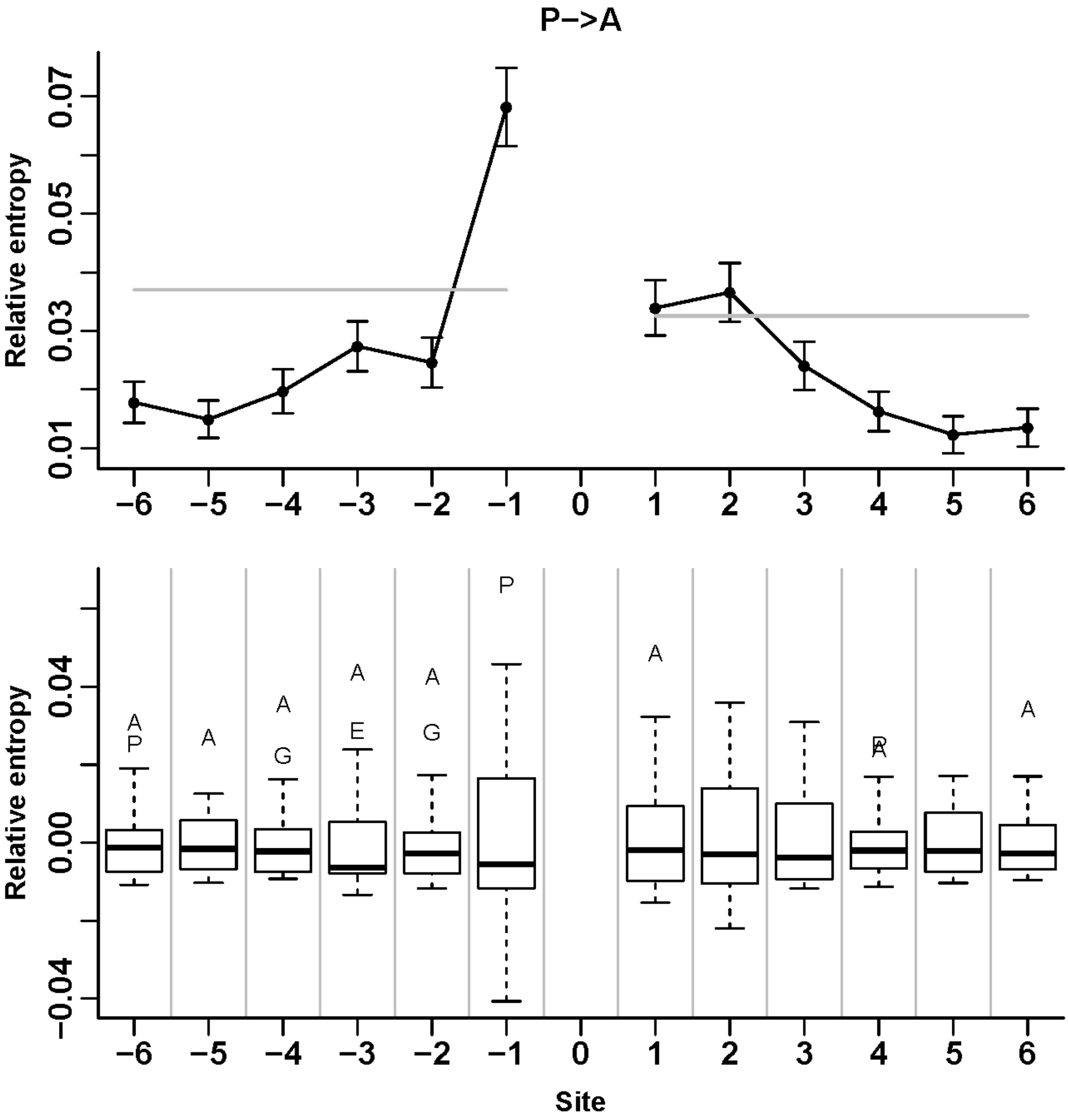

P–>A
Relative entropy
0.01
0.03
0.05
0.07
−6
−5
−4
−3
−2
−1
0
1
2
3
4
5
6
Relative entropy
−0.04
0.00
0.04
A
P
A
A
G
A
E
A
G
P
A
A
A
Site

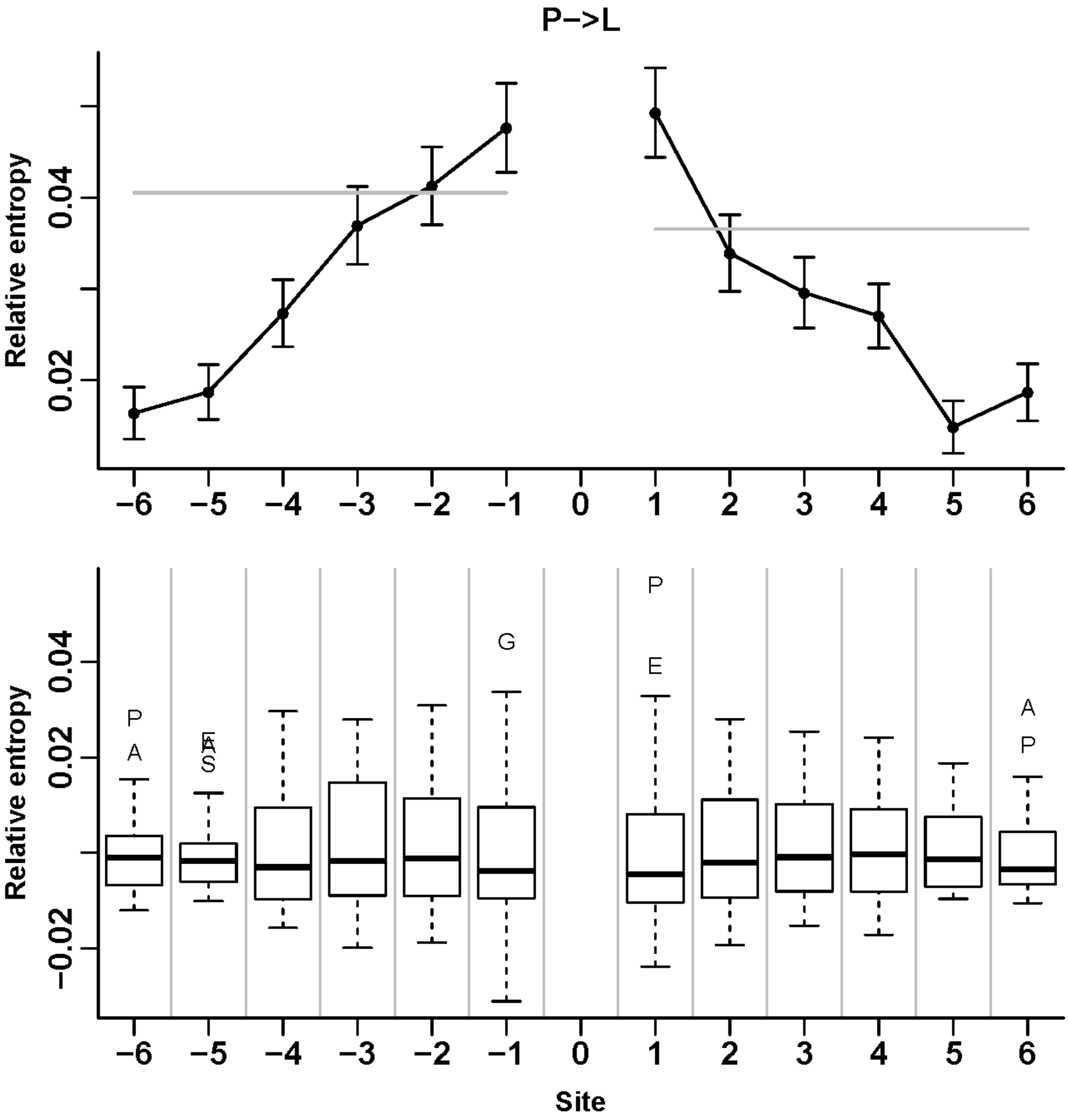
P−>L
Relative entropy
0.02
0.04
−6
−5
−4
−3
−2
−1
0
1
2
3
4
5
6
Relative entropy
−0.02
0.02
0.04
P
A
E
A
S
G
P
E
A
P
Site

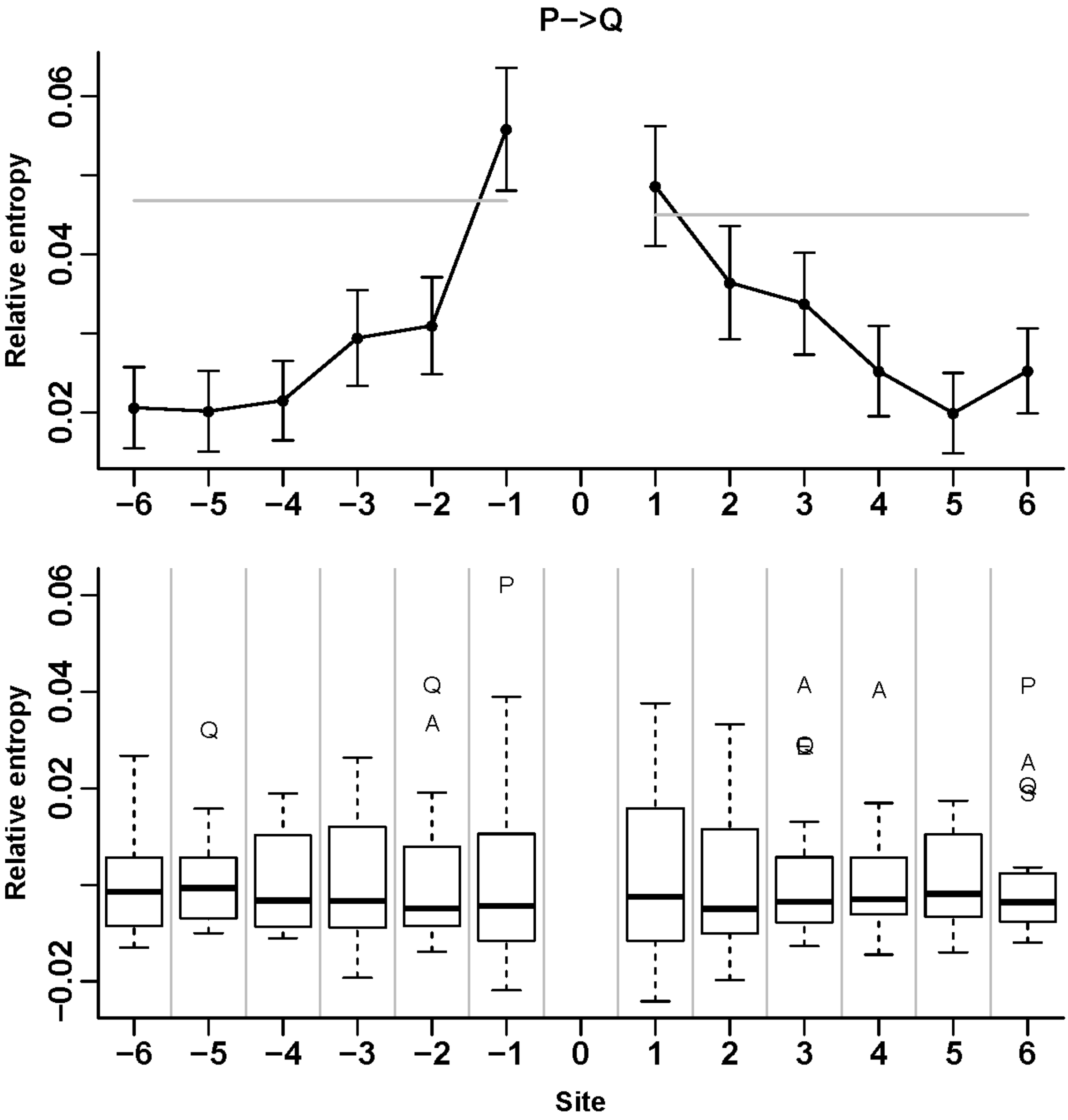
P−>Q
Relative entropy
0.02
0.04
0.06
−6
−5
−4
−3
−2
−1
0
1
2
3
4
5
6
Q
Q
A
P
A
A
A
P
A
S
−0.02
Site

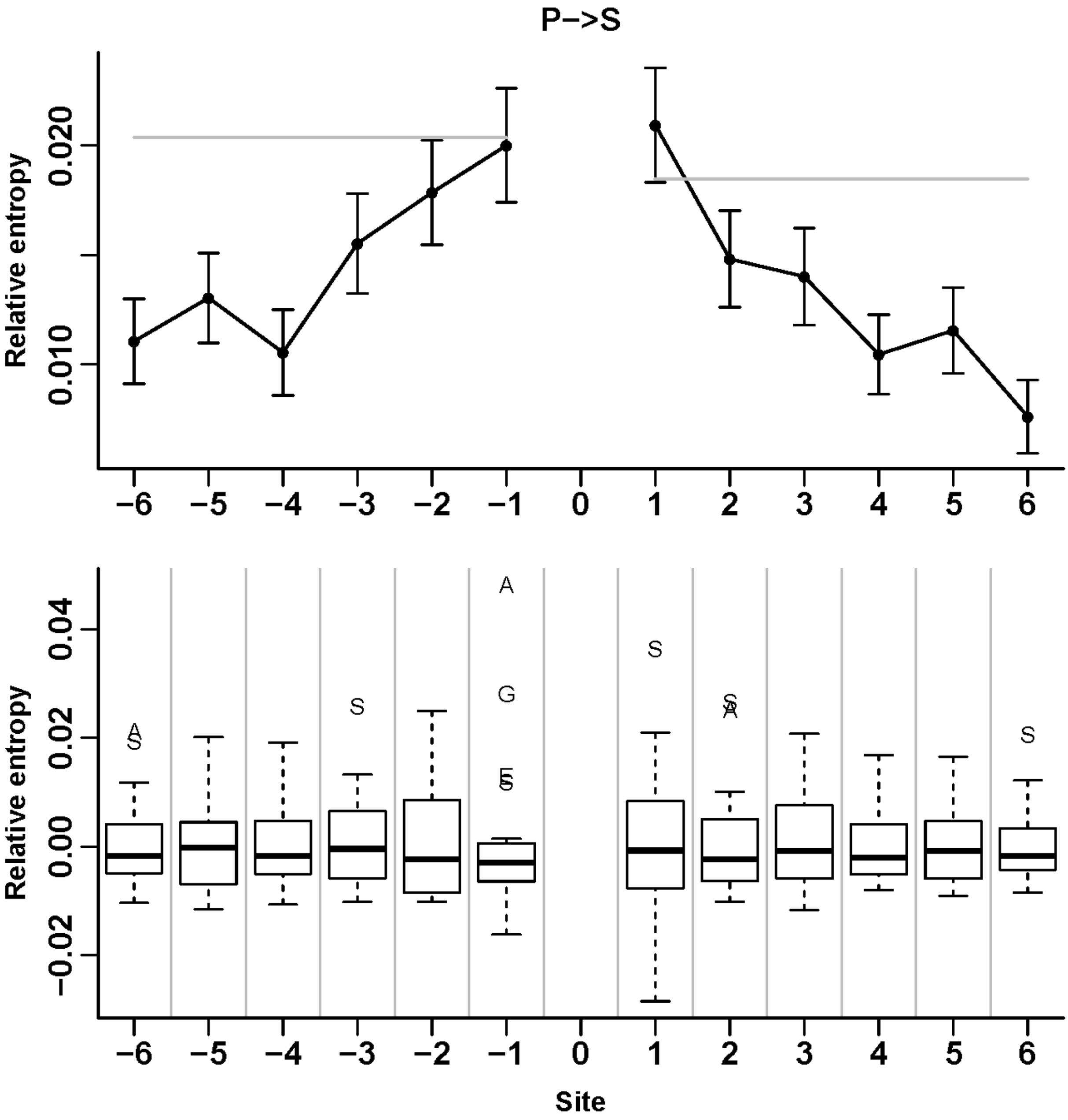
P−>S
Relative entropy
0.010
0.020
−6
−5
−4
−3
−2
−1
0
1
2
3
4
5
6
Relative entropy
−0.02
0.00
0.02
0.04
A
S
G
Site

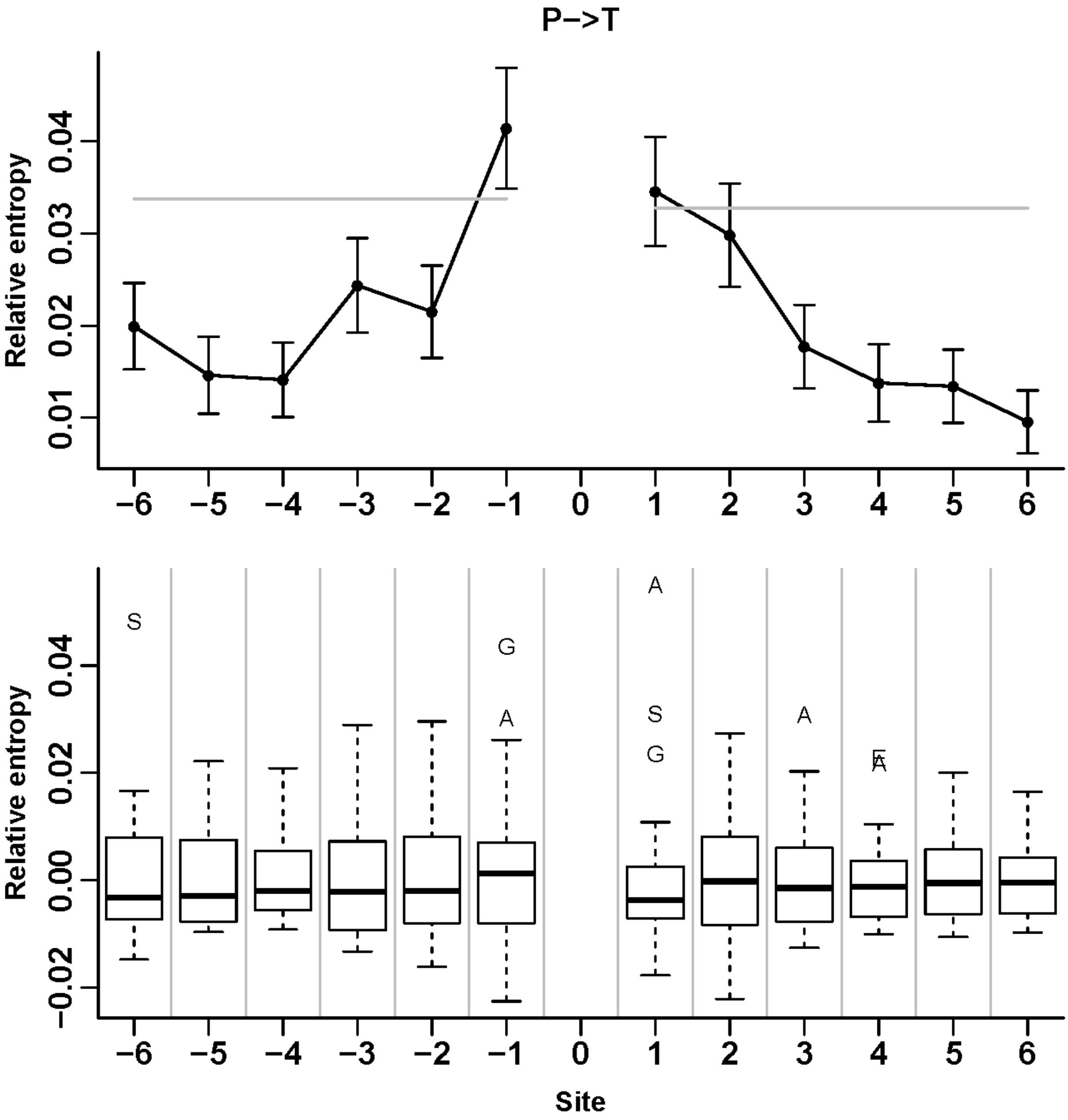
P−>T
Relative entropy
0.01
0.02
0.03
0.04
−6
−5
−4
−3
−2
−1
0
1
2
3
4
5
6
Relative entropy
−0.02
0.00
0.02
0.04
S
G
A
A
S
G
A
Site

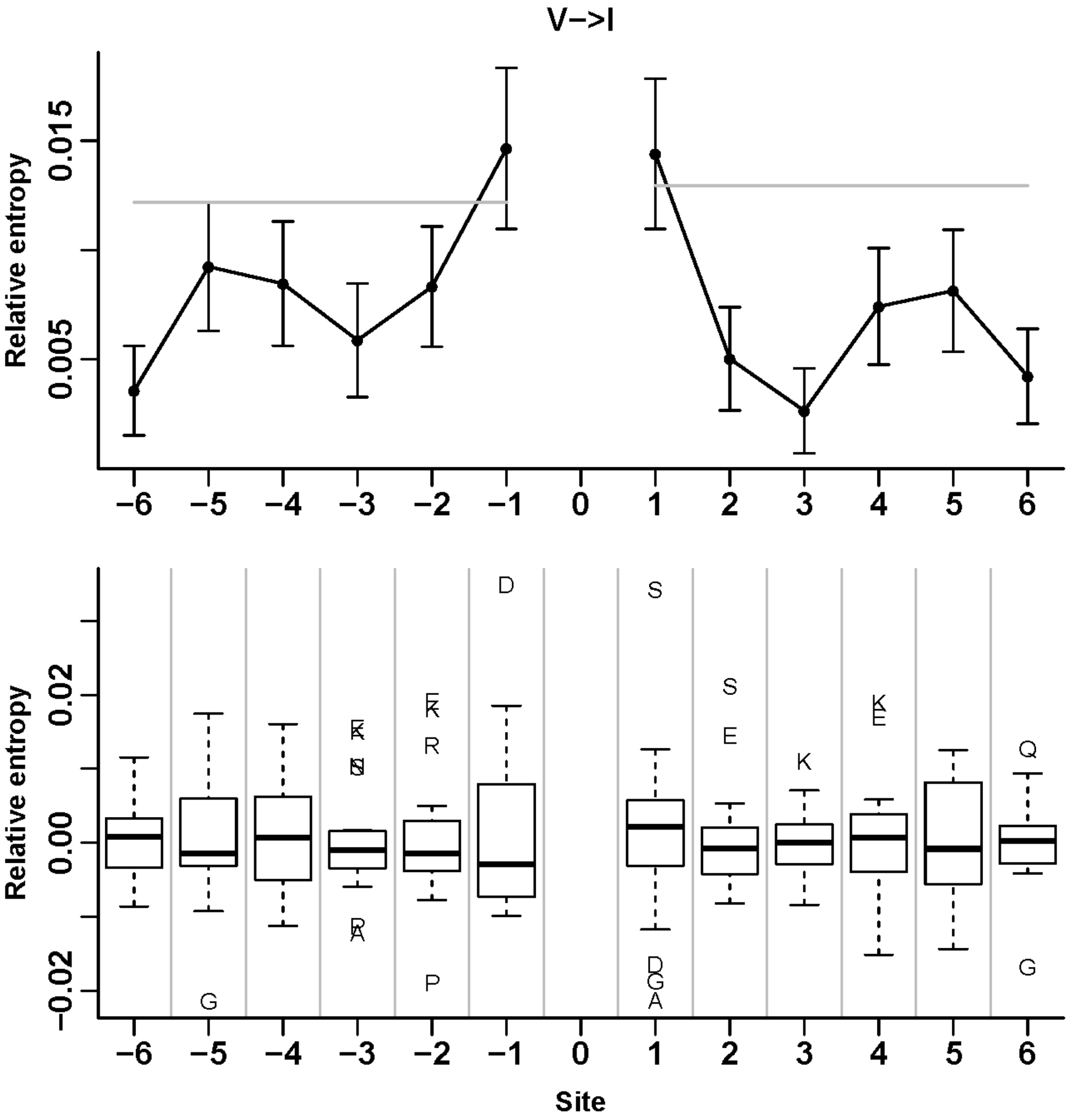
V−>I
Relative entropy
0.015
0.005
−6 −5 −4 −3 −2 −1 0 1 2 3 4 5 6
Relative entropy
0.02
0.00
−0.02
D
S
S
E
K
K
E
Q
R
P
G
D
G
A
G
−6 −5 −4 −3 −2 −1 0 1 2 3 4 5 6
Site

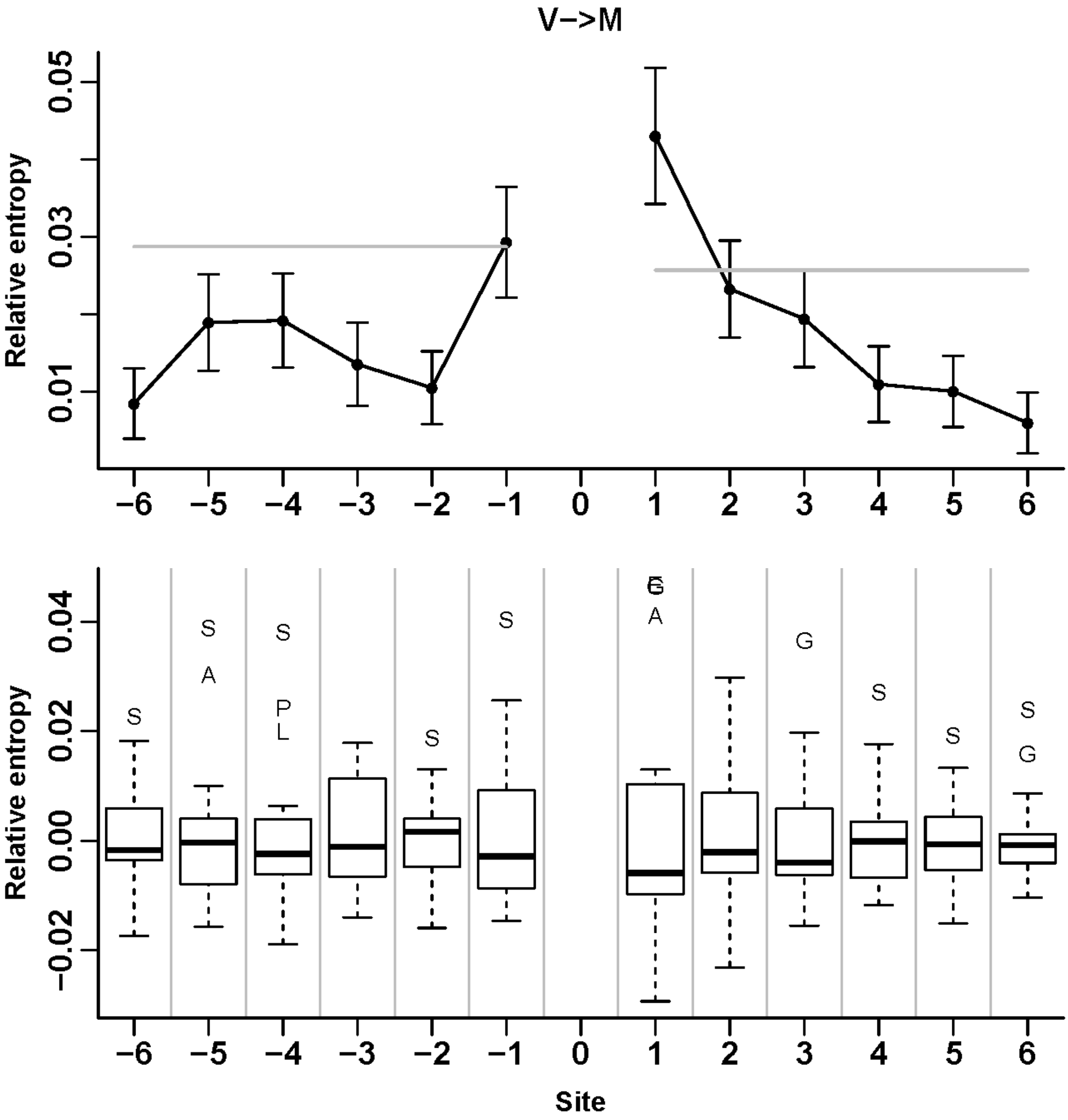
V−>M
Relative entropy
0.05
0.03
0.01
−6
−5
−4
−3
−2
−1
0
1
2
3
4
5
6
Relative entropy
0.04
0.02
0.00
−0.02
S
S
A
S
P
L
S
S
A
G
S
S
S
G
Site

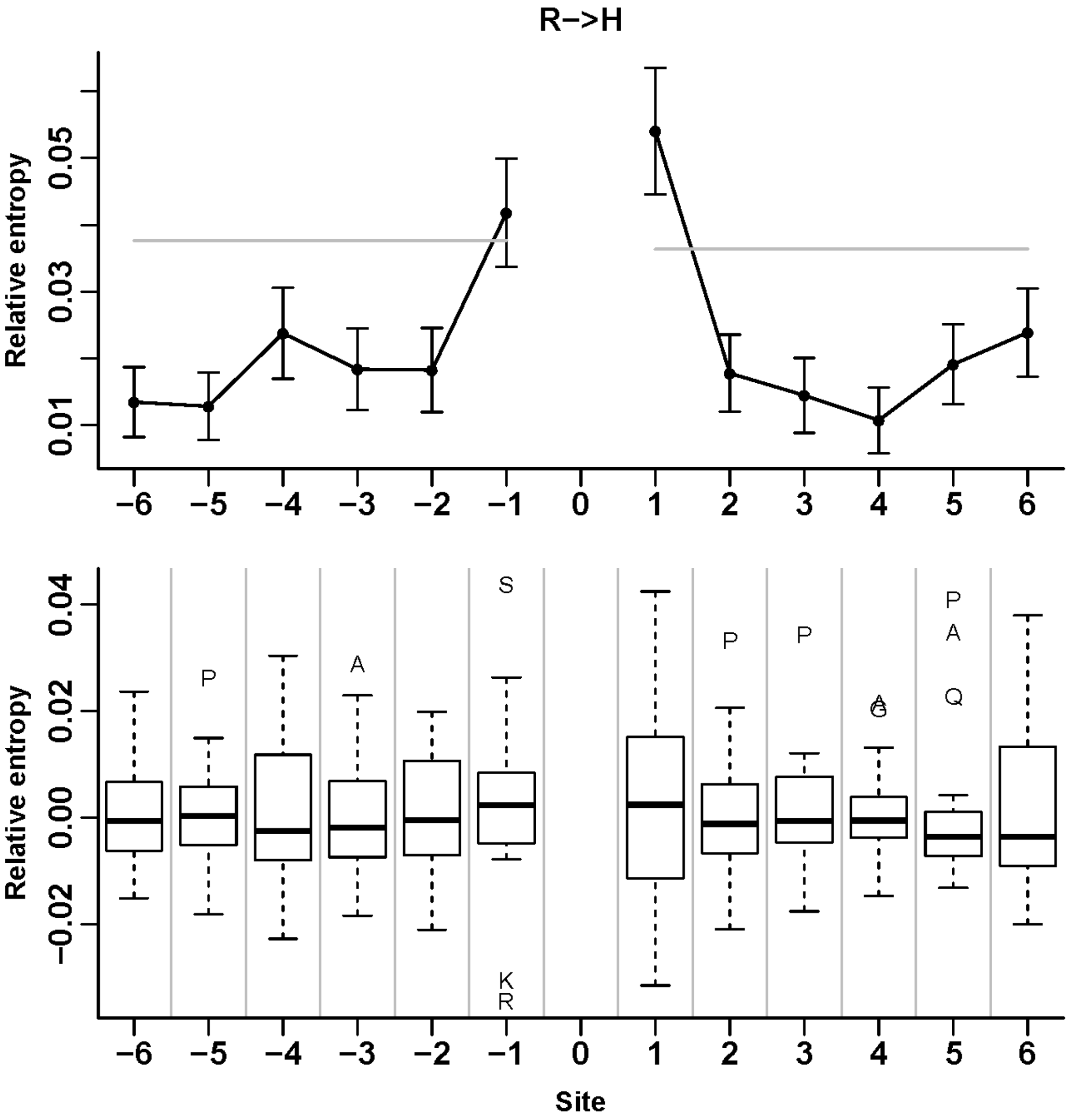
R−>H
Relative entropy
0.01
0.03
0.05
−6
−5
−4
−3
−2
−1
0
1
2
3
4
5
6
Relative entropy
−0.02
0.00
0.02
0.04
P
A
S
K
R
P
P
A
G
P
A
Q
Site

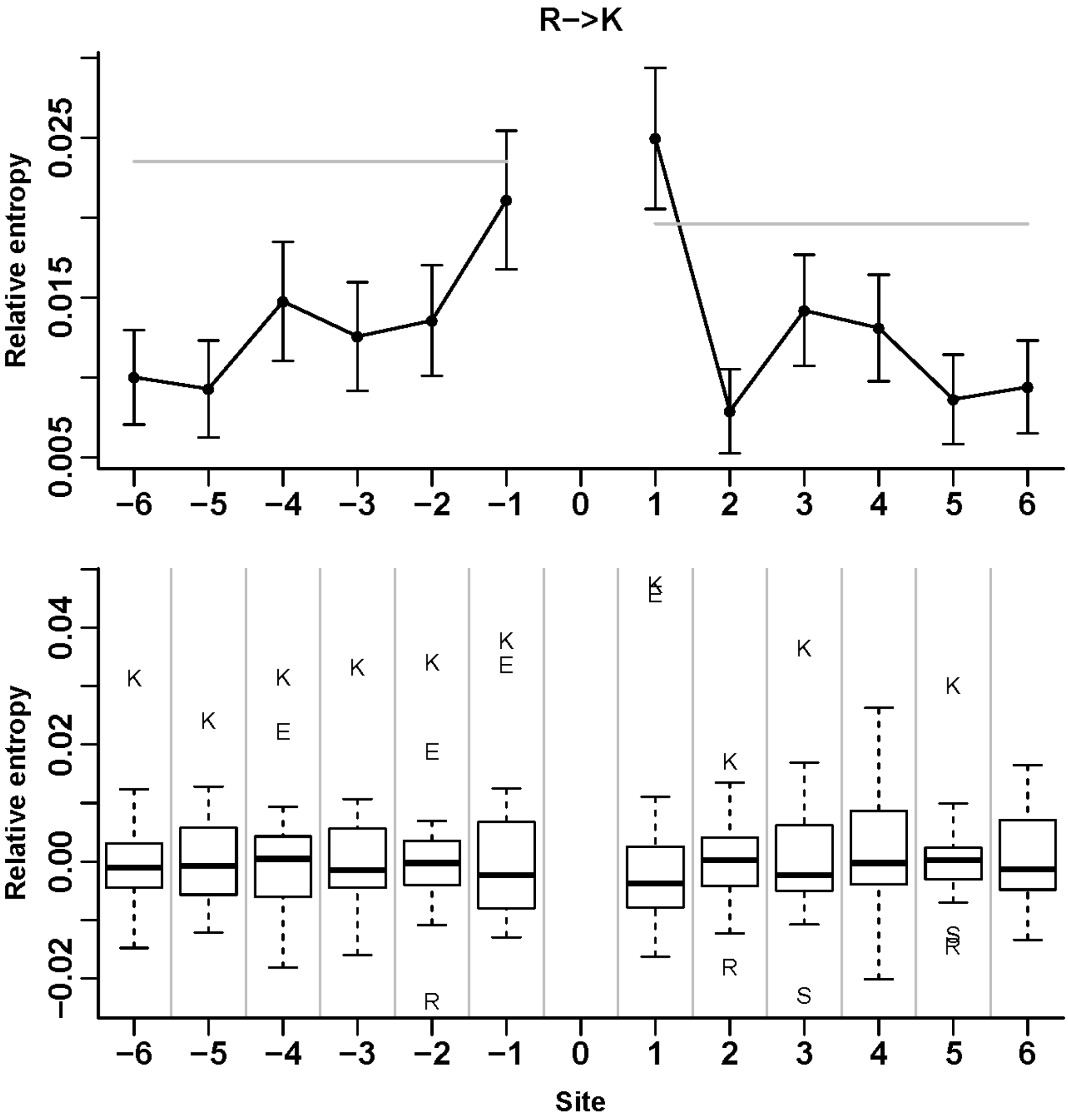
R−>K
Relative entropy
0.005
0.015
0.025
−6
−5
−4
−3
−2
−1
0
1
2
3
4
5
6
Relative entropy
−0.02
0.00
0.02
0.04
K
E
R
S
Site

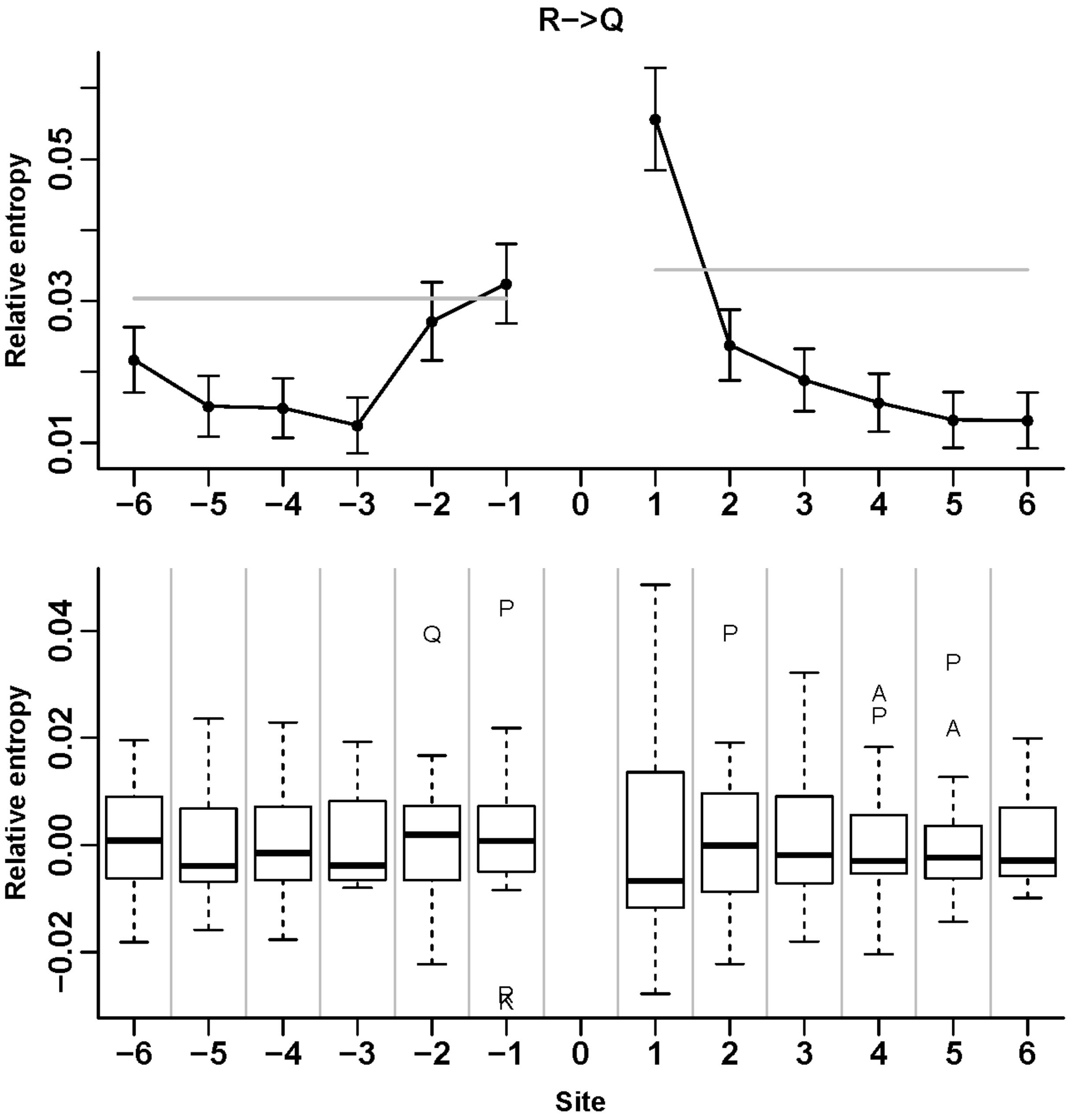
R−>Q
Relative entropy
0.01
0.03
0.05
−6
−5
−4
−3
−2
−1
0
1
2
3
4
5
6
Relative entropy
−0.02
0.00
0.02
0.04
Q
P
P
A
P
P
A
R
K
Site

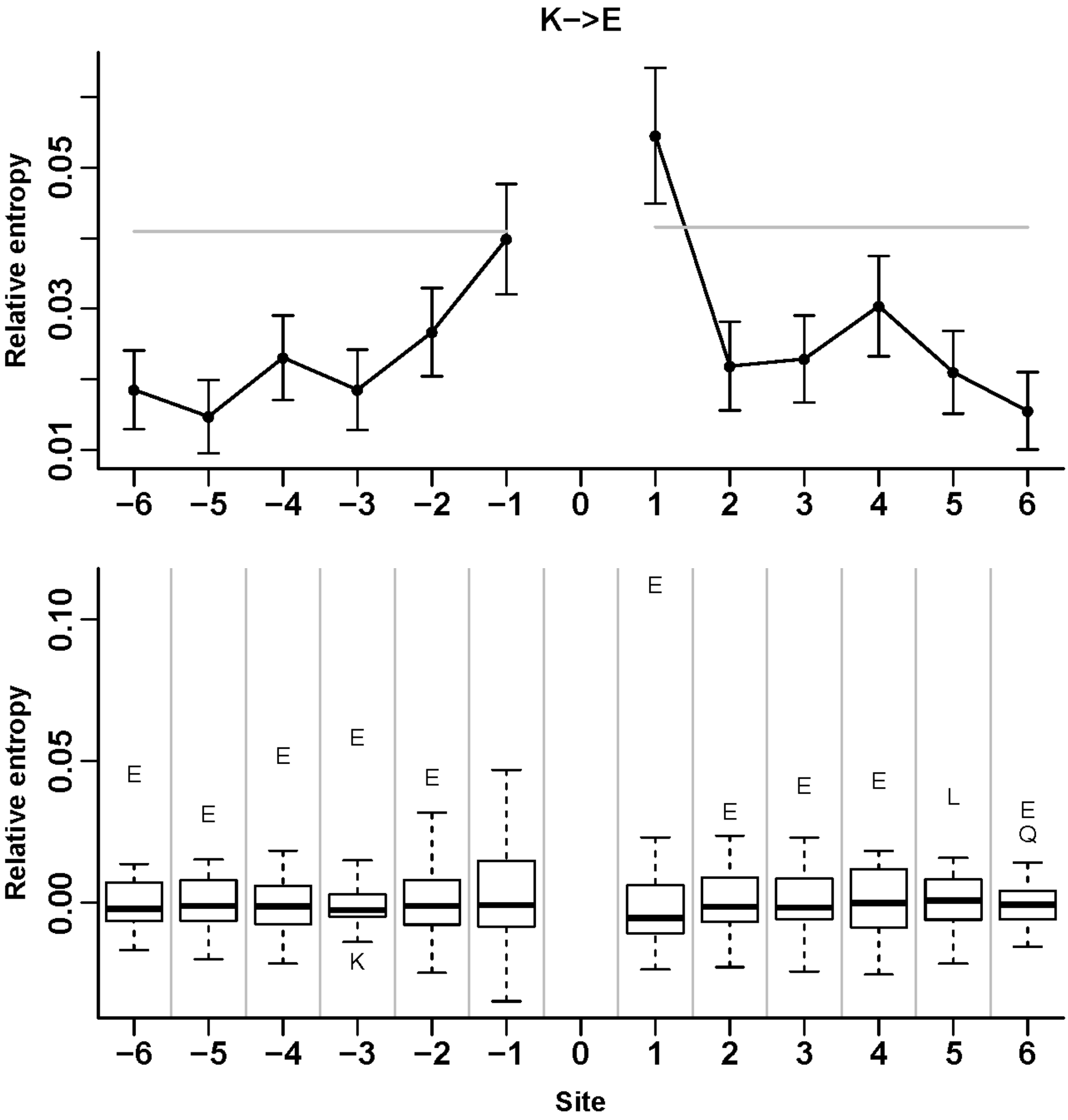
K−>E
Relative entropy
0.01
0.03
0.05
−6
−5
−4
−3
−2
−1
0
1
2
3
4
5
6
Relative entropy
0.00
0.05
0.10
E
E
E
E
K
E
E
E
E
E
L
E
Q
Site

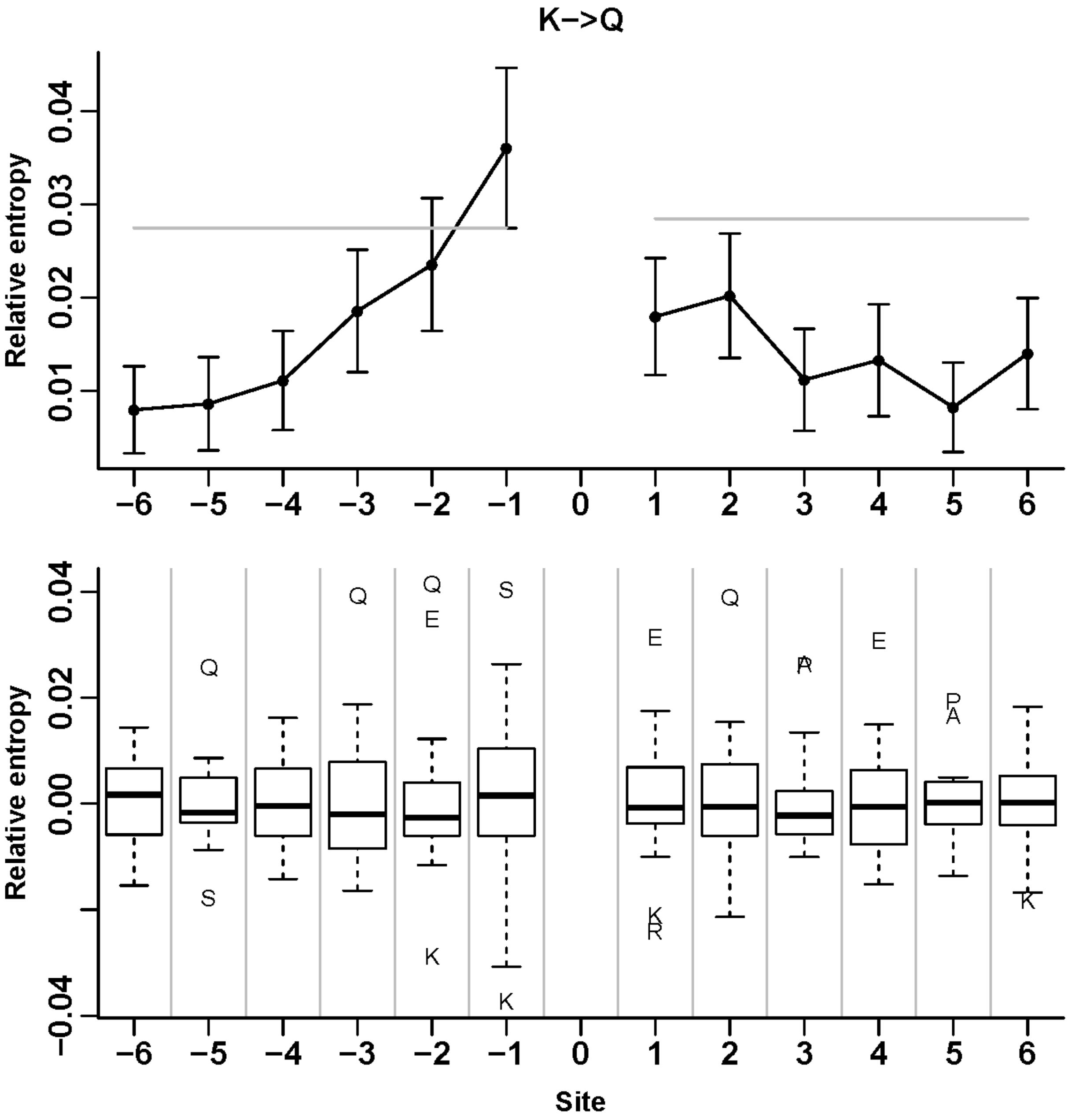
K−>Q
Relative entropy
0.01
0.02
0.03
0.04
−6
−5
−4
−3
−2
−1
0
1
2
3
4
5
6
Relative entropy
−0.04
0.00
0.02
0.04
Q
S
Q
Q
E
K
S
K
E
K
R
Q
A
R
E
P
A
K
Site

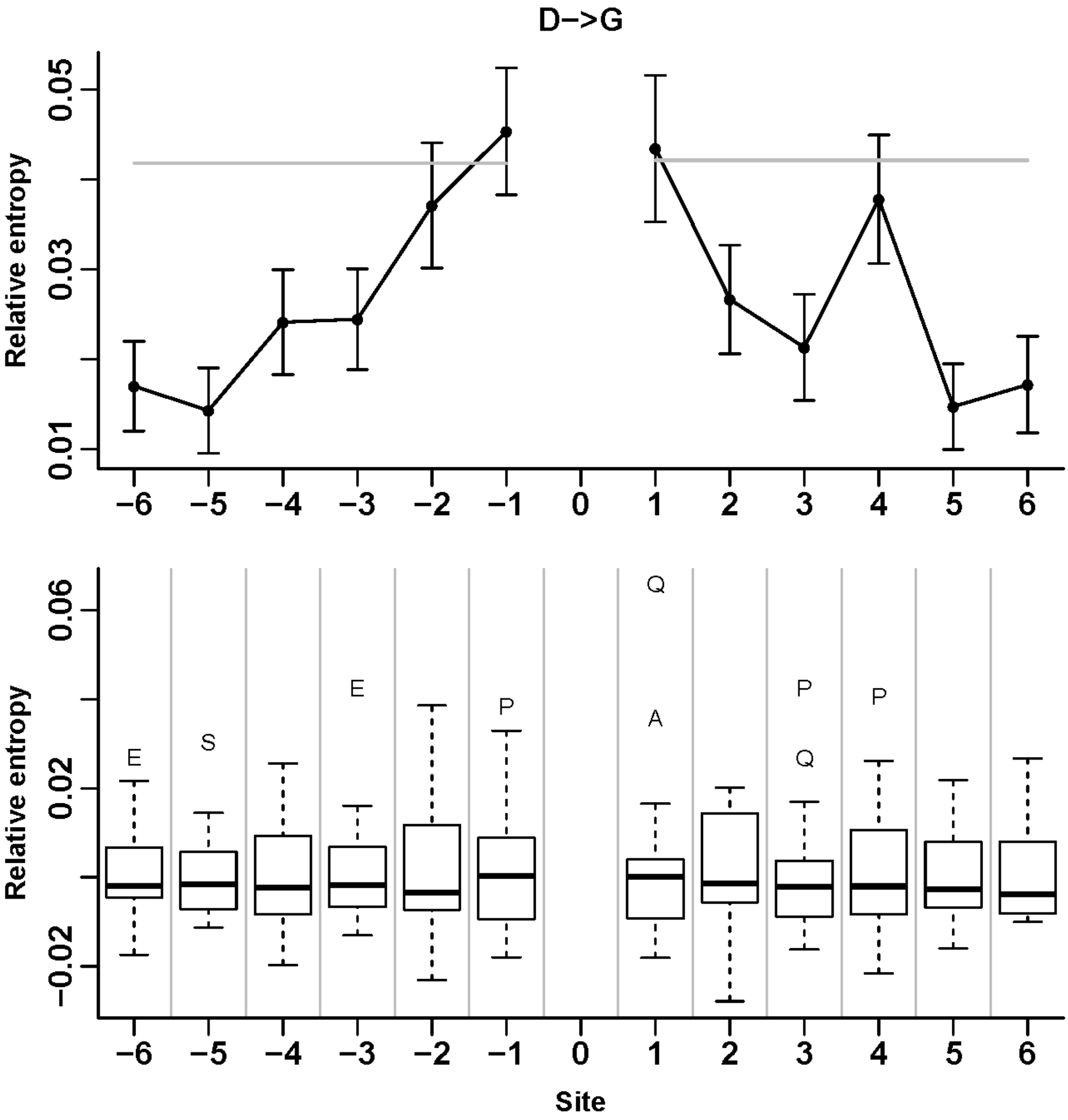
D−>G
Relative entropy
0.01
0.03
0.05
−6
−5
−4
−3
−2
−1
0
1
2
3
4
5
6
Relative entropy
−0.02
0.02
0.06
E
S
E
P
Q
A
P
Q
P
Site

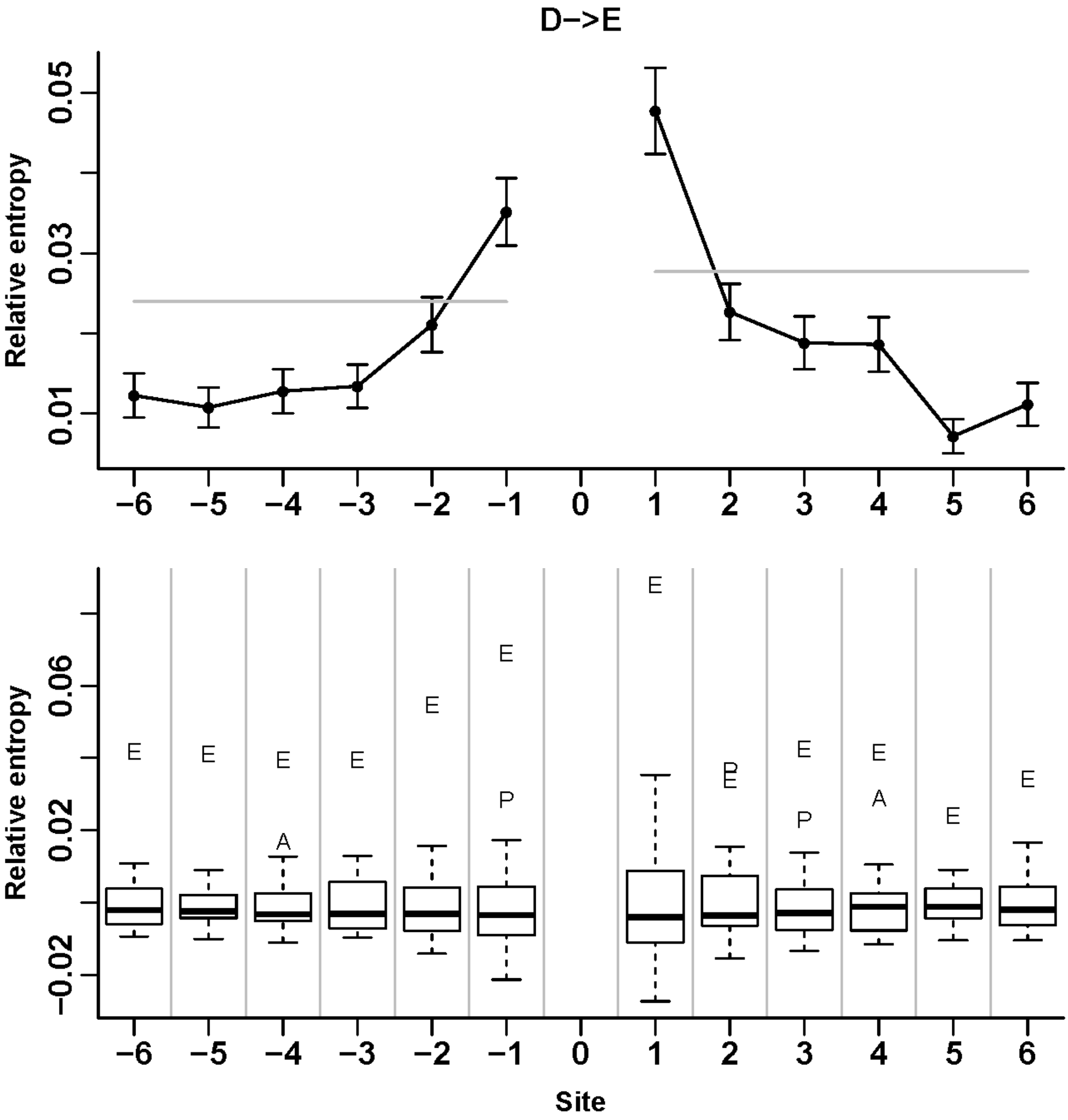
D−>E
Relative entropy
0.01
0.03
0.05
−6
−5
−4
−3
−2
−1
0
1
2
3
4
5
6
Relative entropy
−0.02
0.02
0.06
E
E
E
E
E
E
E
E
E
E
E
E
E
A
P
P
P
A
Site

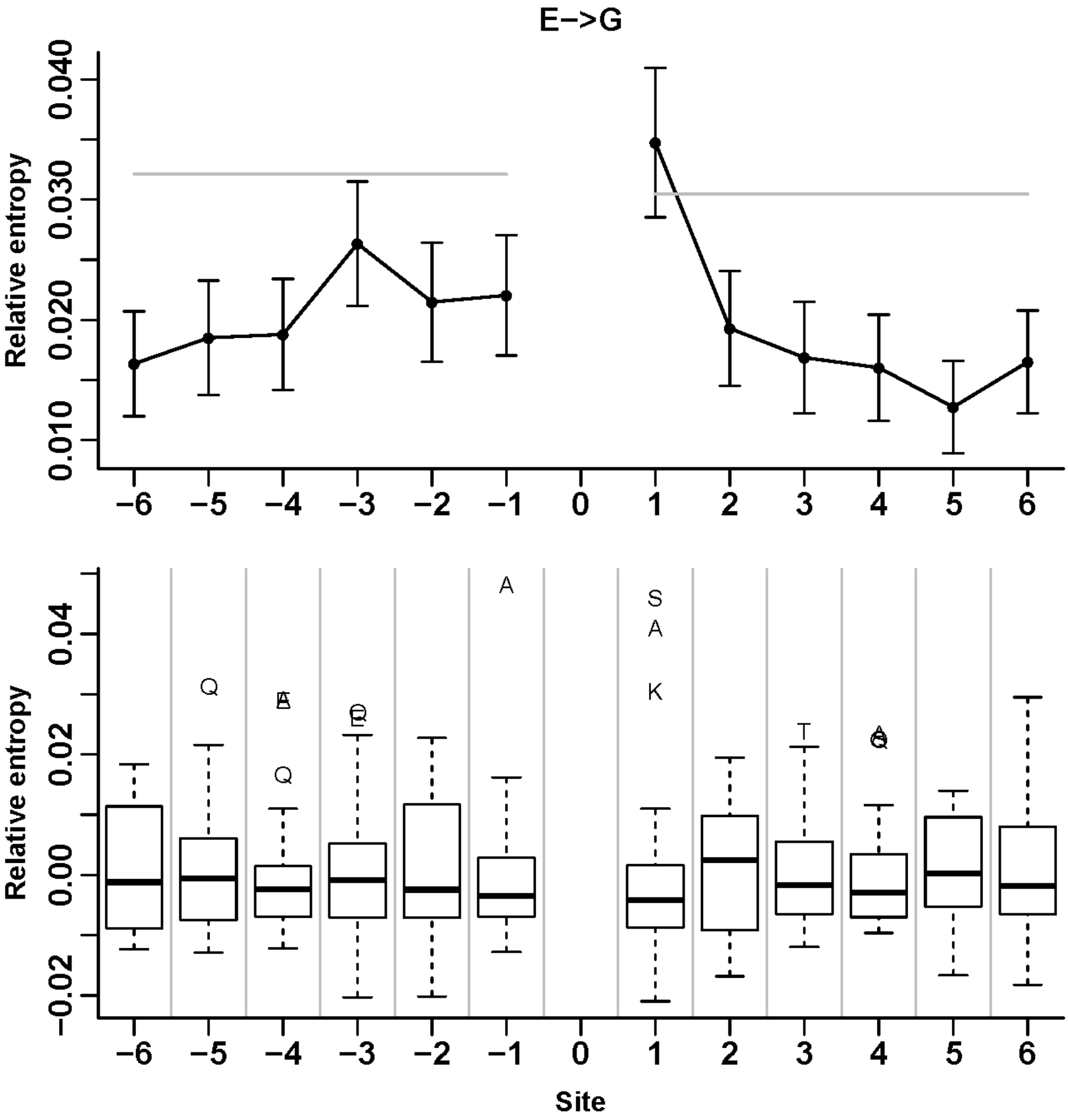
E−>G
Relative entropy
0.010
0.020
0.030
0.040
−6
−5
−4
−3
−2
−1
0
1
2
3
4
5
6
Relative entropy
−0.02
0.00
0.02
0.04
Q
Q
A
S
A
K
T
Site

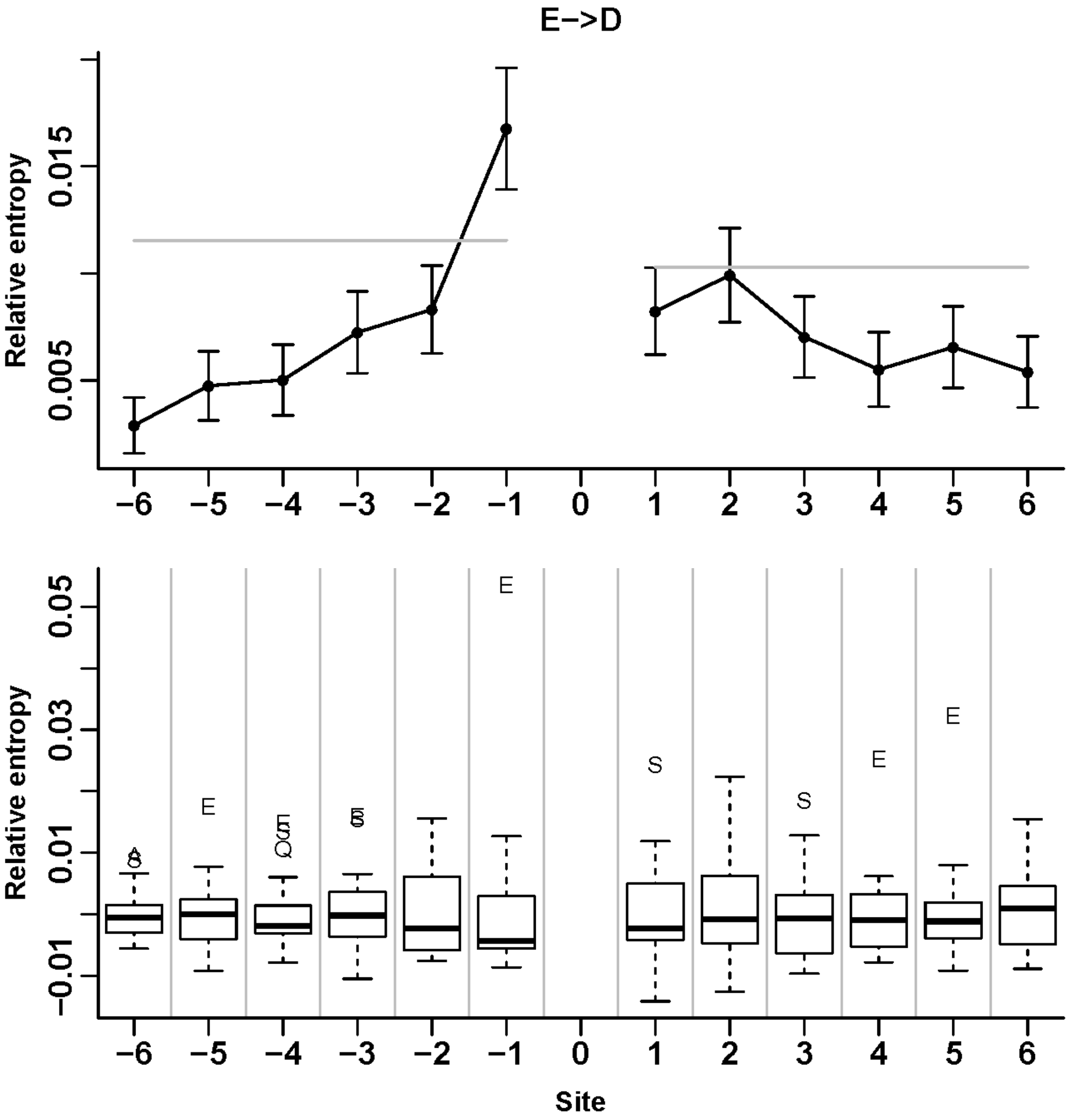
E−>D
Relative entropy
0.005
0.015
−6 −5 −4 −3 −2 −1 0 1 2 3 4 5 6
Relative entropy
−0.01
0.01
0.03
0.05
E
E
S
S
E
E
−6 −5 −4 −3 −2 −1 0 1 2 3 4 5 6
Site

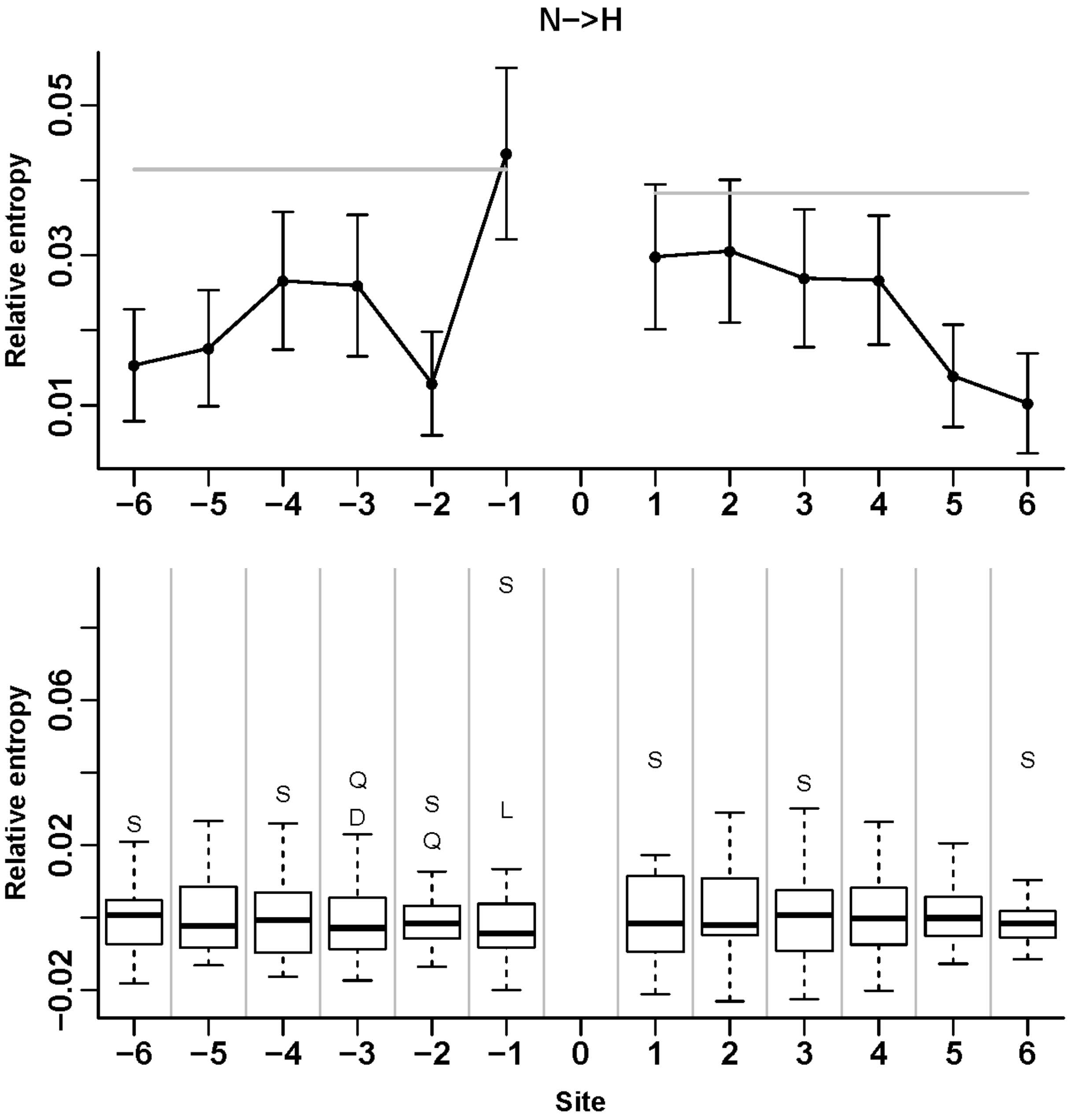
N−>H
Relative entropy
0.01
0.03
0.05
−6 −5 −4 −3 −2 −1 0 1 2 3 4 5 6
Relative entropy
−0.02
0.02
0.06
S
S
Q
D
S
Q
S
L
S
S
S
S
−6 −5 −4 −3 −2 −1 0 1 2 3 4 5 6
Site

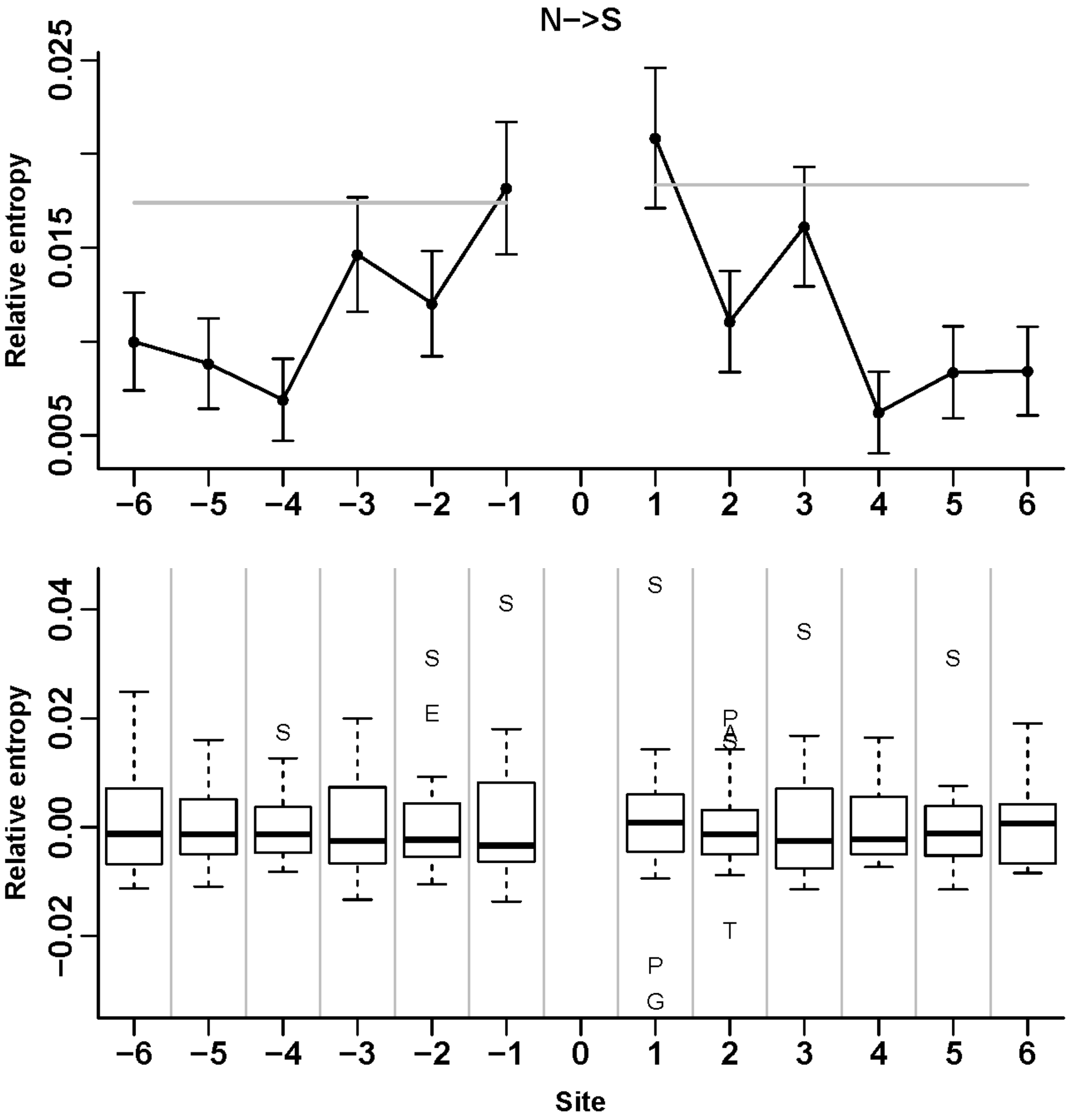
N−>S
Relative entropy
0.005
0.015
0.025
−6
−5
−4
−3
−2
−1
0
1
2
3
4
5
6
Relative entropy
−0.02
0.00
0.02
0.04
S
S
E
S
S
P
A
S
T
P
G
S
S
Site

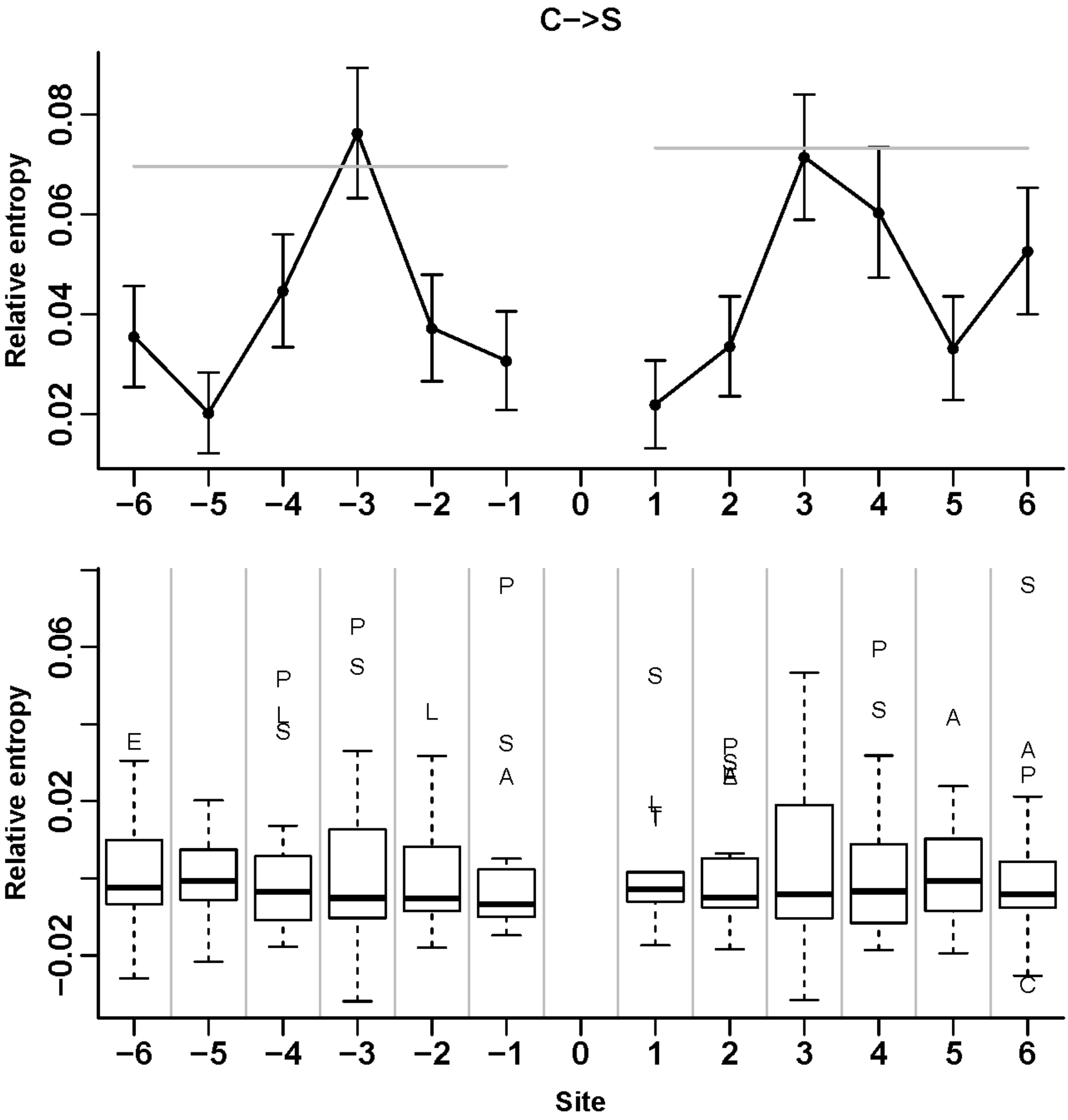
C−>S
Relative entropy
0.02
0.04
0.06
0.08
−6
−5
−4
−3
−2
−1
0
1
2
3
4
5
6
Relative entropy
−0.02
0.02
0.06
Site

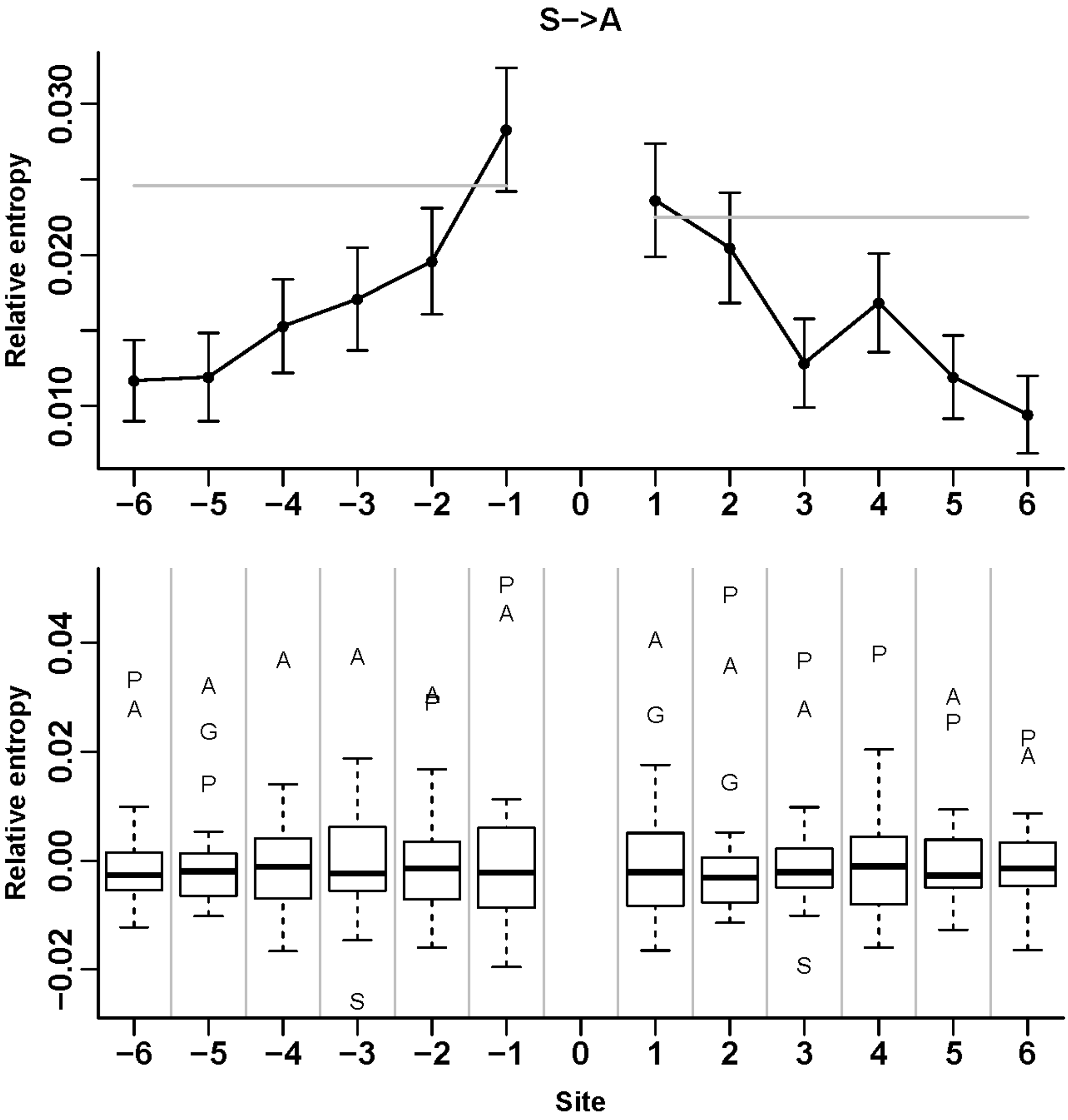
S−>A
Relative entropy
0.010
0.020
0.030
−6
−5
−4
−3
−2
−1
0
1
2
3
4
5
6
Relative entropy
−0.02
0.00
0.02
0.04
Site

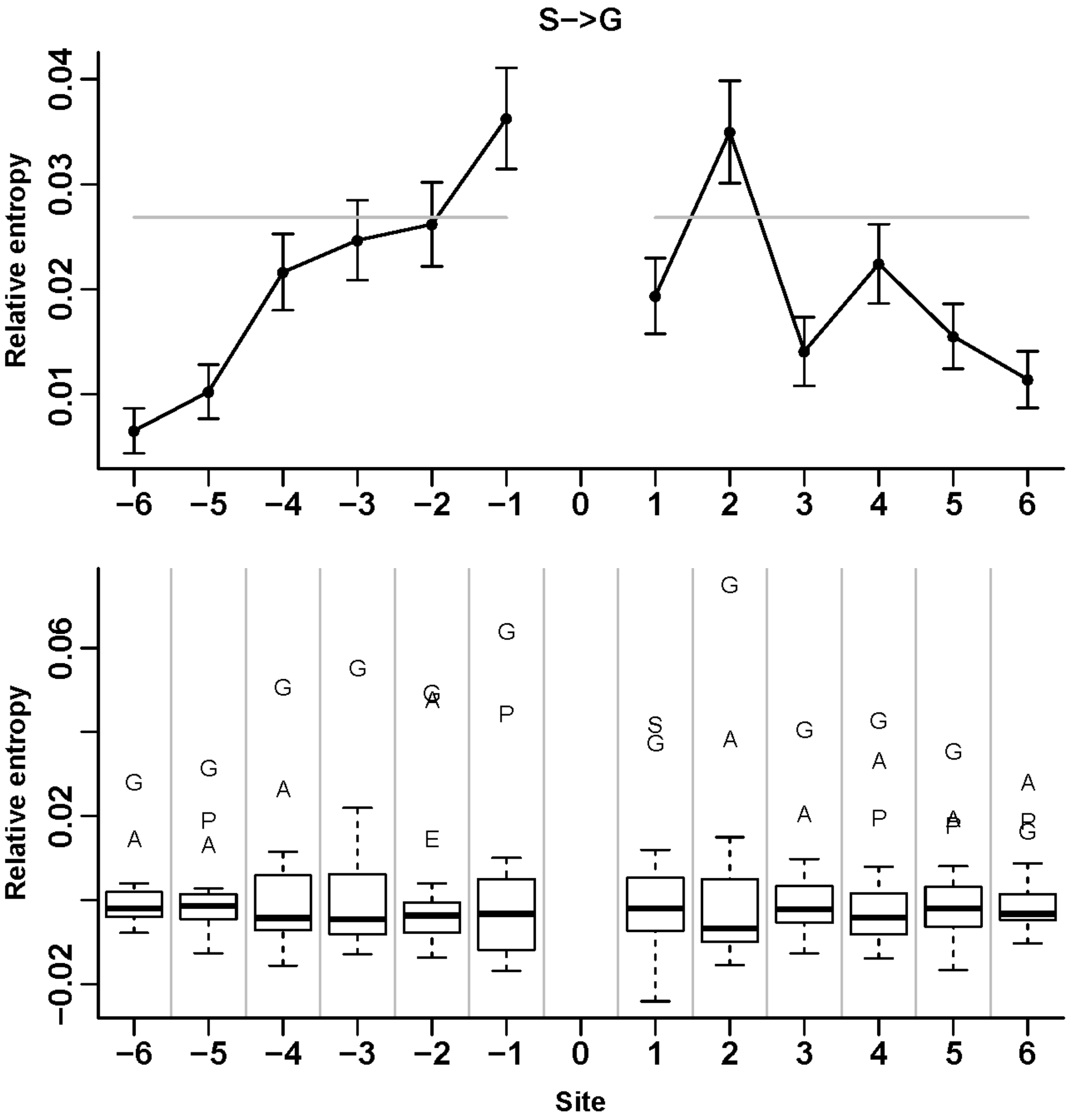
S−>G
Relative entropy
0.01
0.02
0.03
0.04
−6
−5
−4
−3
−2
−1
0
1
2
3
4
5
6
Relative entropy
−0.02
0.02
0.06
Site

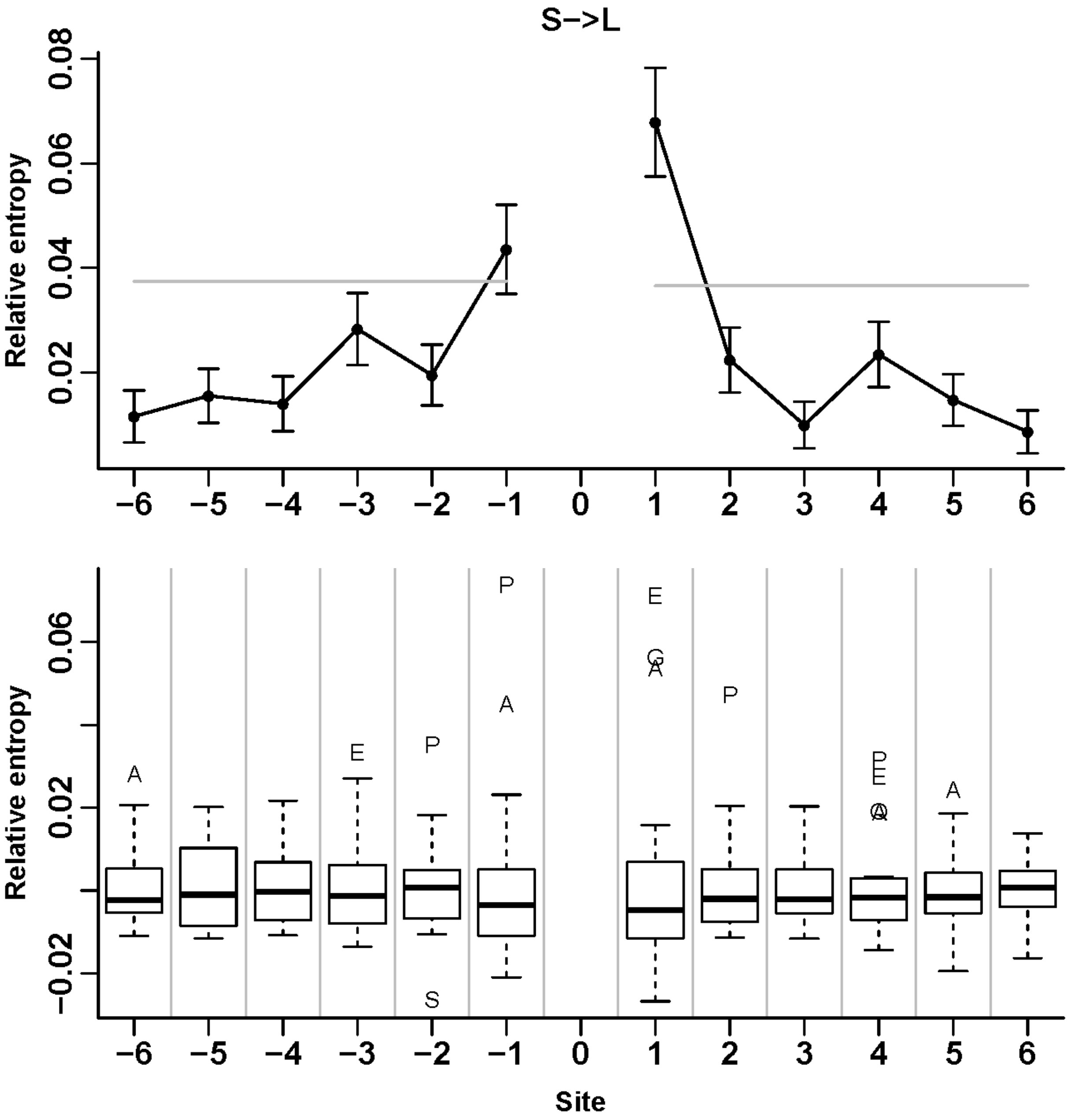
S−>L
Relative entropy
0.08
0.06
0.04
0.02
−6 −5 −4 −3 −2 −1 0 1 2 3 4 5 6
Relative entropy
0.06
0.02
−0.02
A
E
P
S
P
A
E
G
A
P
P
E
A
A
Site

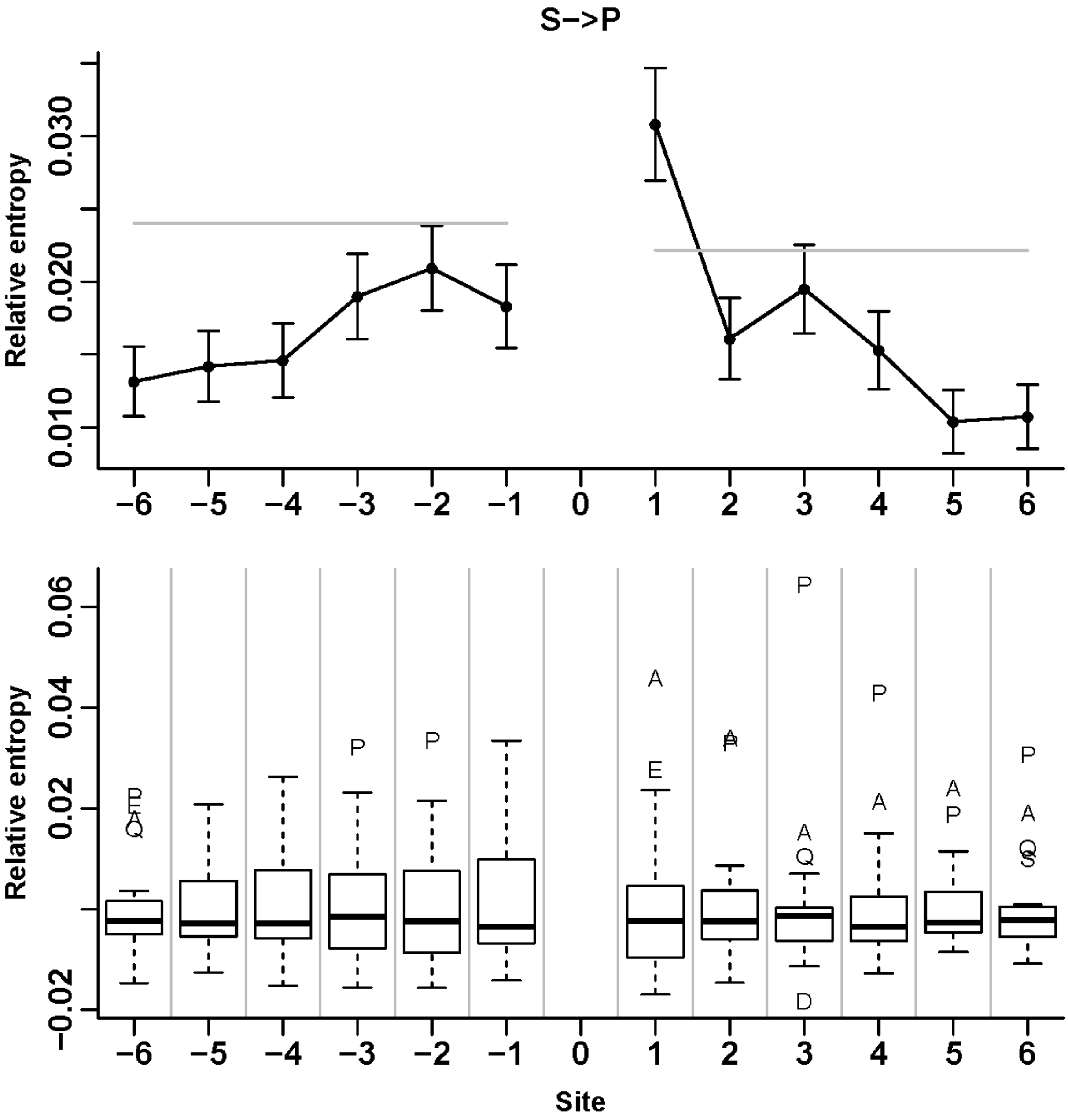
S−>P
Relative entropy
0.010
0.020
0.030
−6
−5
−4
−3
−2
−1
0
1
2
3
4
5
6
Relative entropy
−0.02
0.02
0.04
0.06
P
P
P
A
E
P
A
Q
D
P
A
A
P
P
A
Q
S
Site

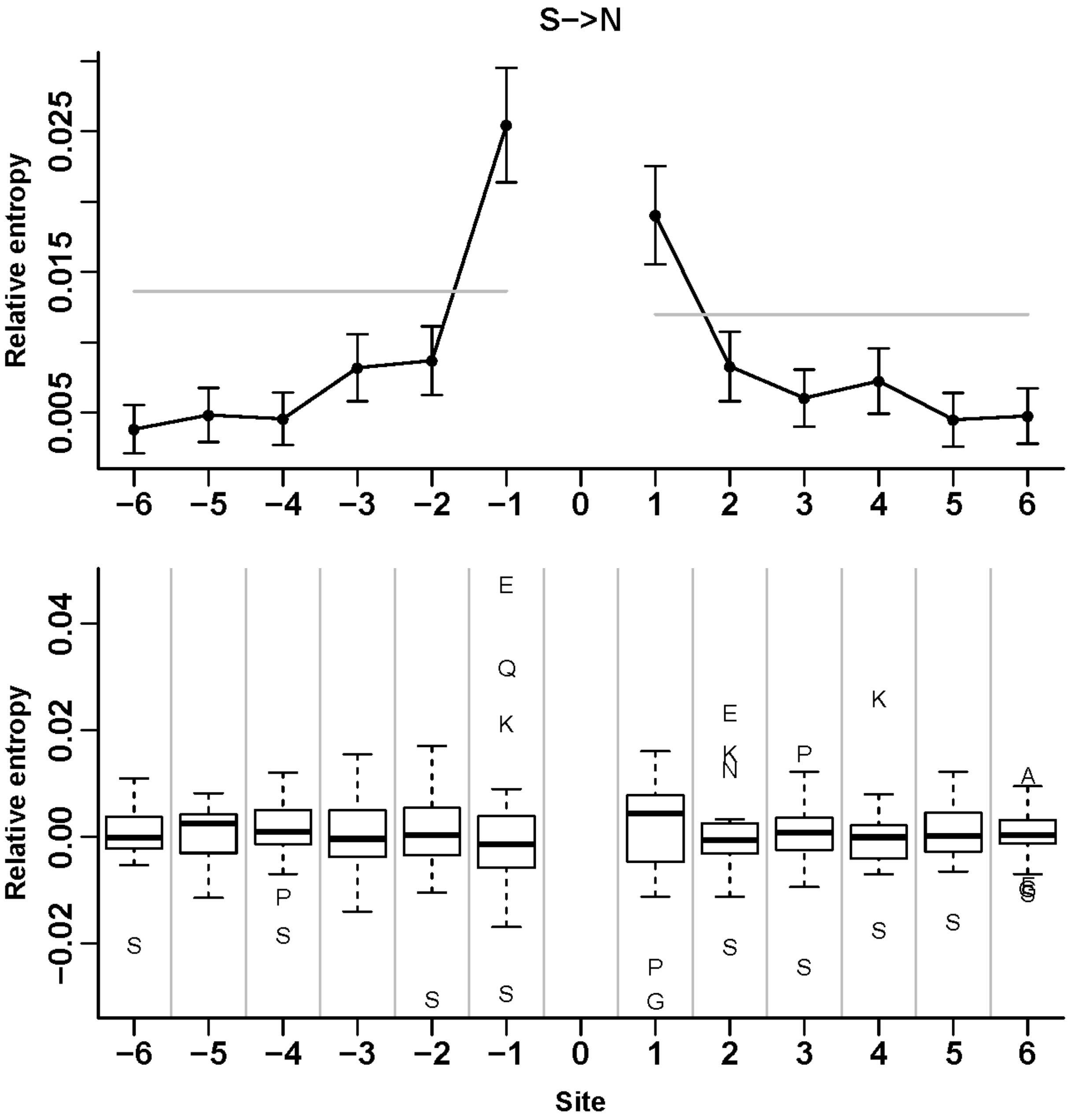
S−>N
Relative entropy
0.005
0.015
0.025
−6
−5
−4
−3
−2
−1
0
1
2
3
4
5
6
Relative entropy
−0.02
0.00
0.02
0.04
E
Q
K
E
K
N
P
K
A
S
P
S
S
S
P
G
S
S
S
S
E
S
Site

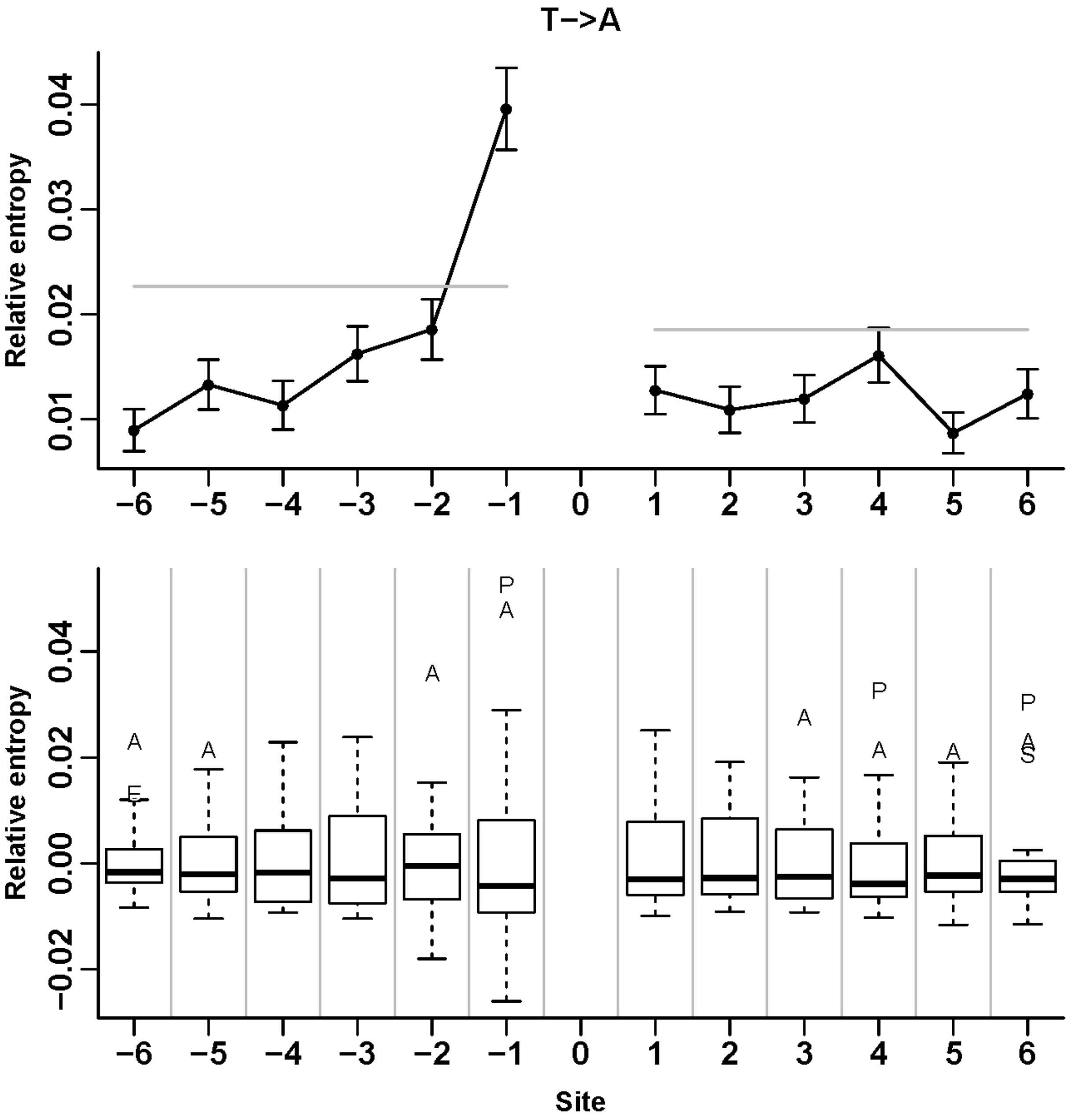
T−>A
Relative entropy
0.01
0.02
0.03
0.04
−6
−5
−4
−3
−2
−1
0
1
2
3
4
5
6
Relative entropy
−0.02
0.00
0.02
0.04
A
E
A
A
P
A
A
P
A
A
P
A
S
Site

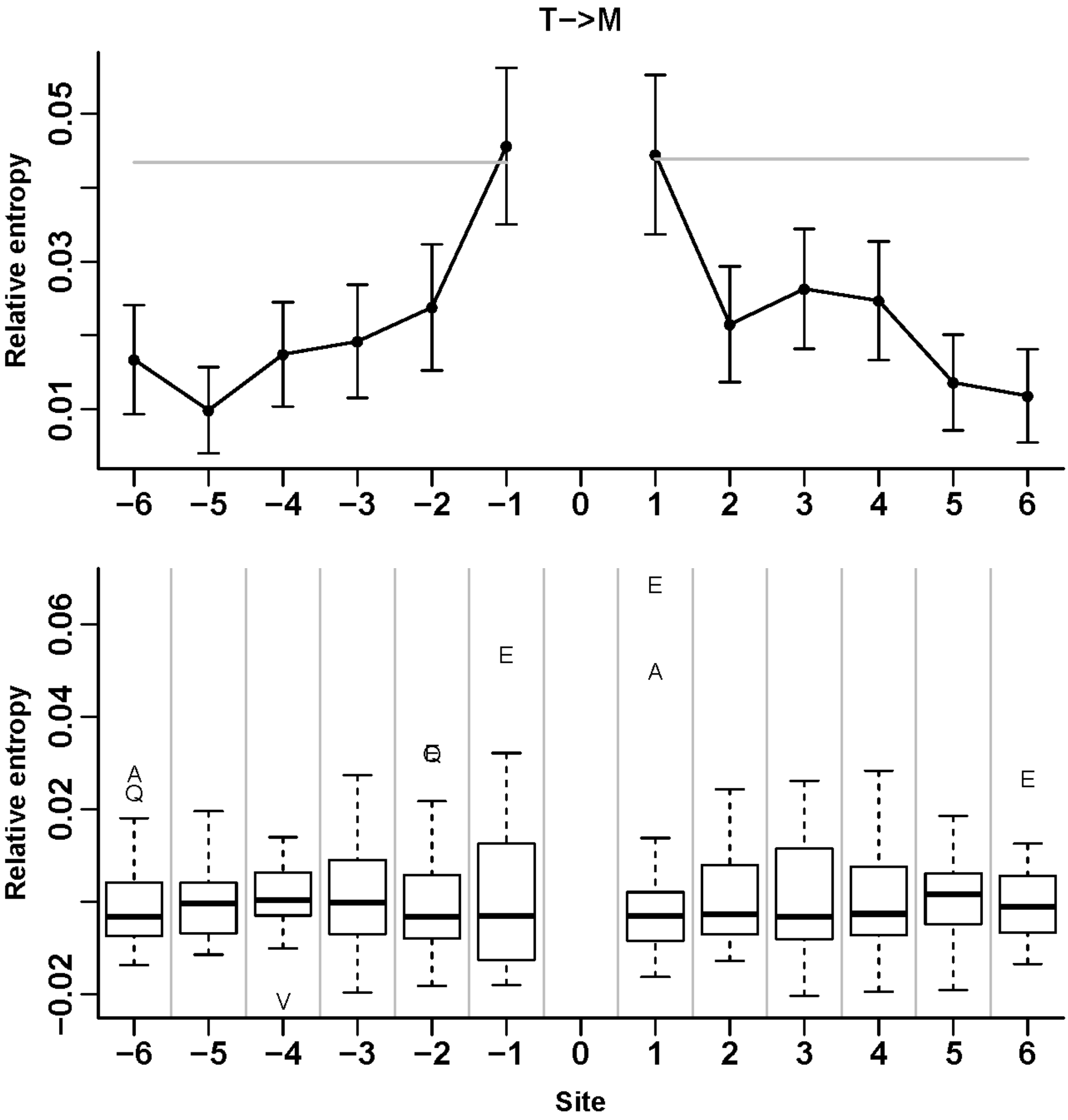
T−>M
Relative entropy
0.01
0.03
0.05
−6
−5
−4
−3
−2
−1
0
1
2
3
4
5
6
Relative entropy
−0.02
0.02
0.04
0.06
A
Q
V
E
A
E
Site

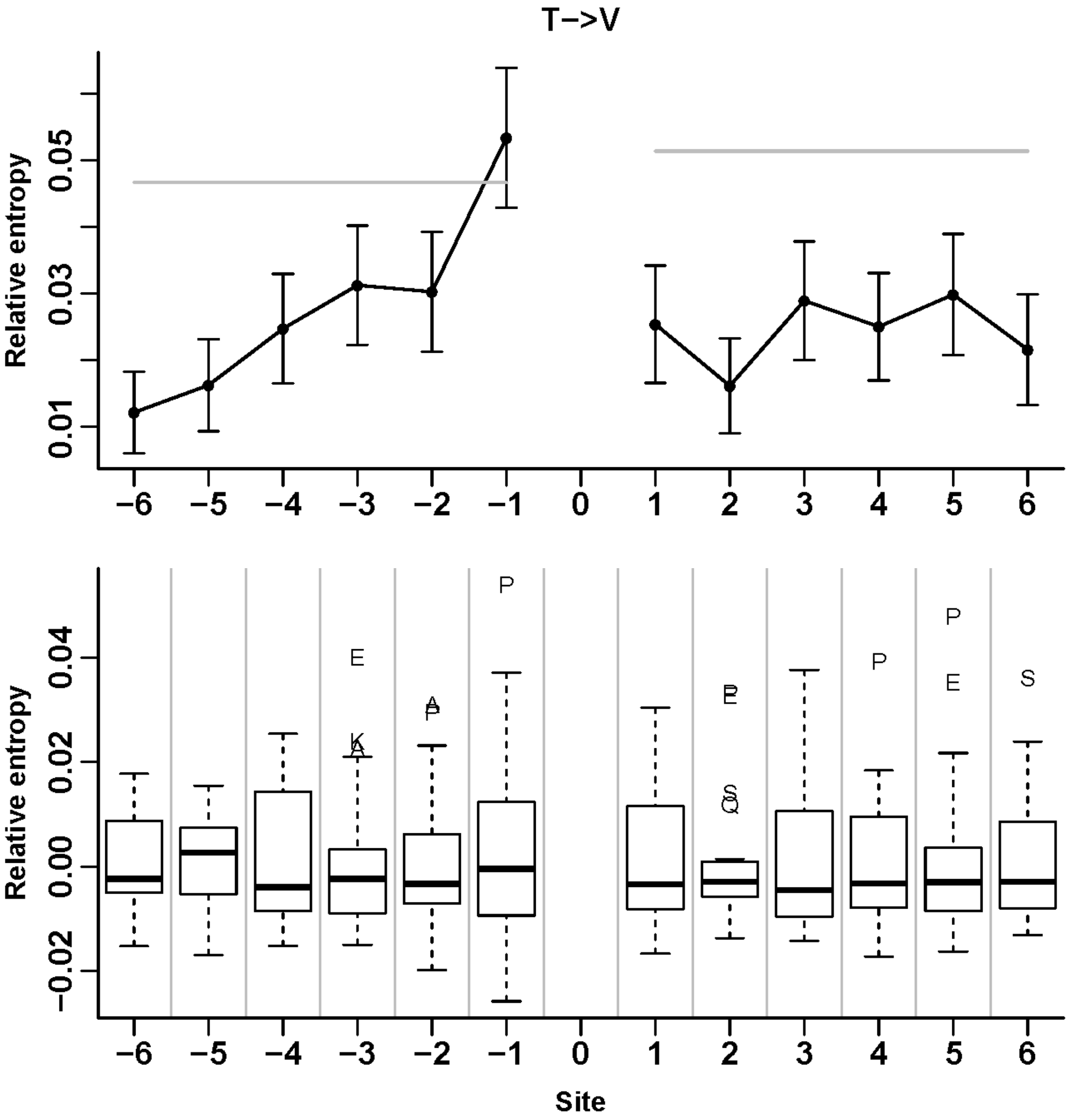
T−>V
Relative entropy
0.01
0.03
0.05
−6
−5
−4
−3
−2
−1
0
1
2
3
4
5
6
Relative entropy
−0.02
0.00
0.02
0.04
P
E
K
A
P
P
E
S
S
Q
P
P
E
Site

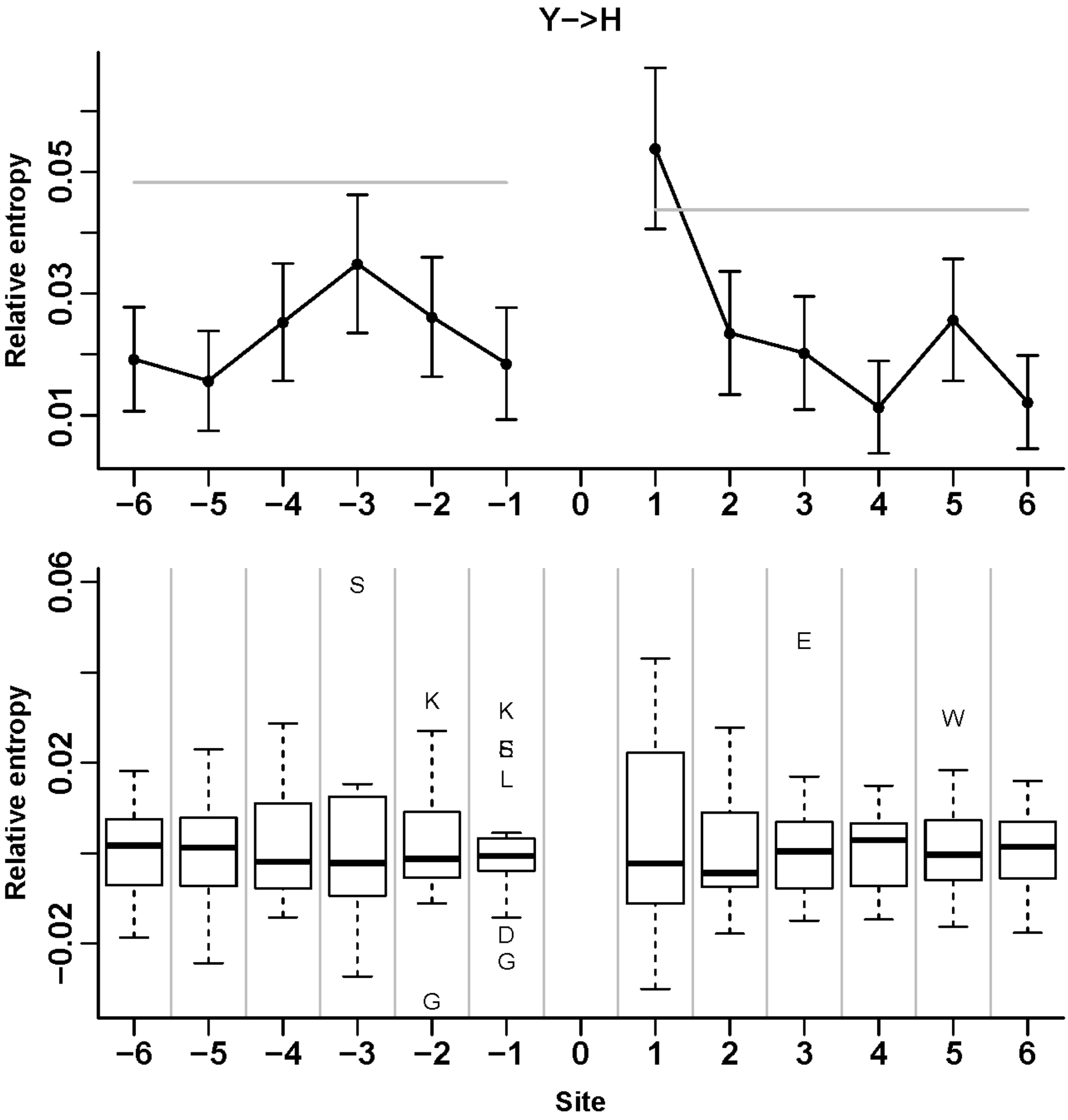
Y−>H
Relative entropy
0.01
0.03
0.05
−6
−5
−4
−3
−2
−1
0
1
2
3
4
5
6
Relative entropy
−0.02
0.02
0.06
S
K
K
L
D
G
G
E
W
Site

Figure S8. Neighbor preference patterns of G in collagen and other proteins.

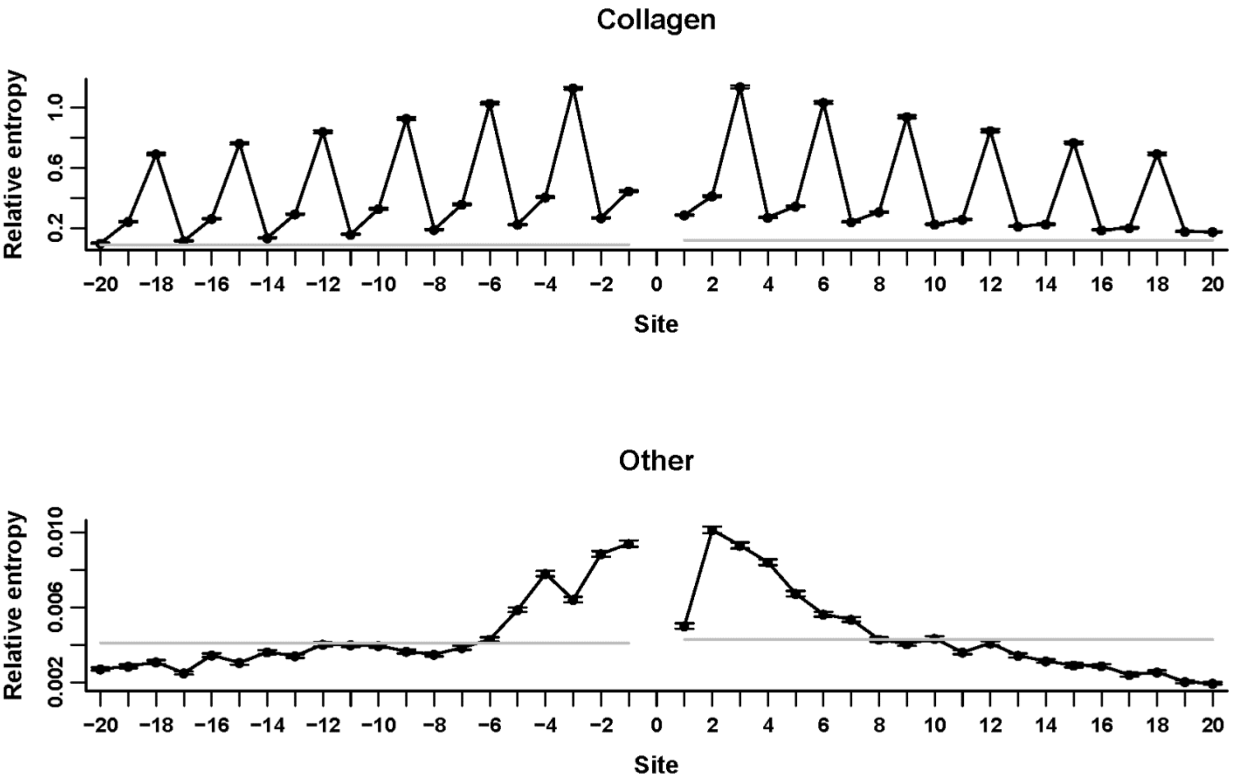

Figure S9. Neighbor preference patterns of C in different cellular locations.

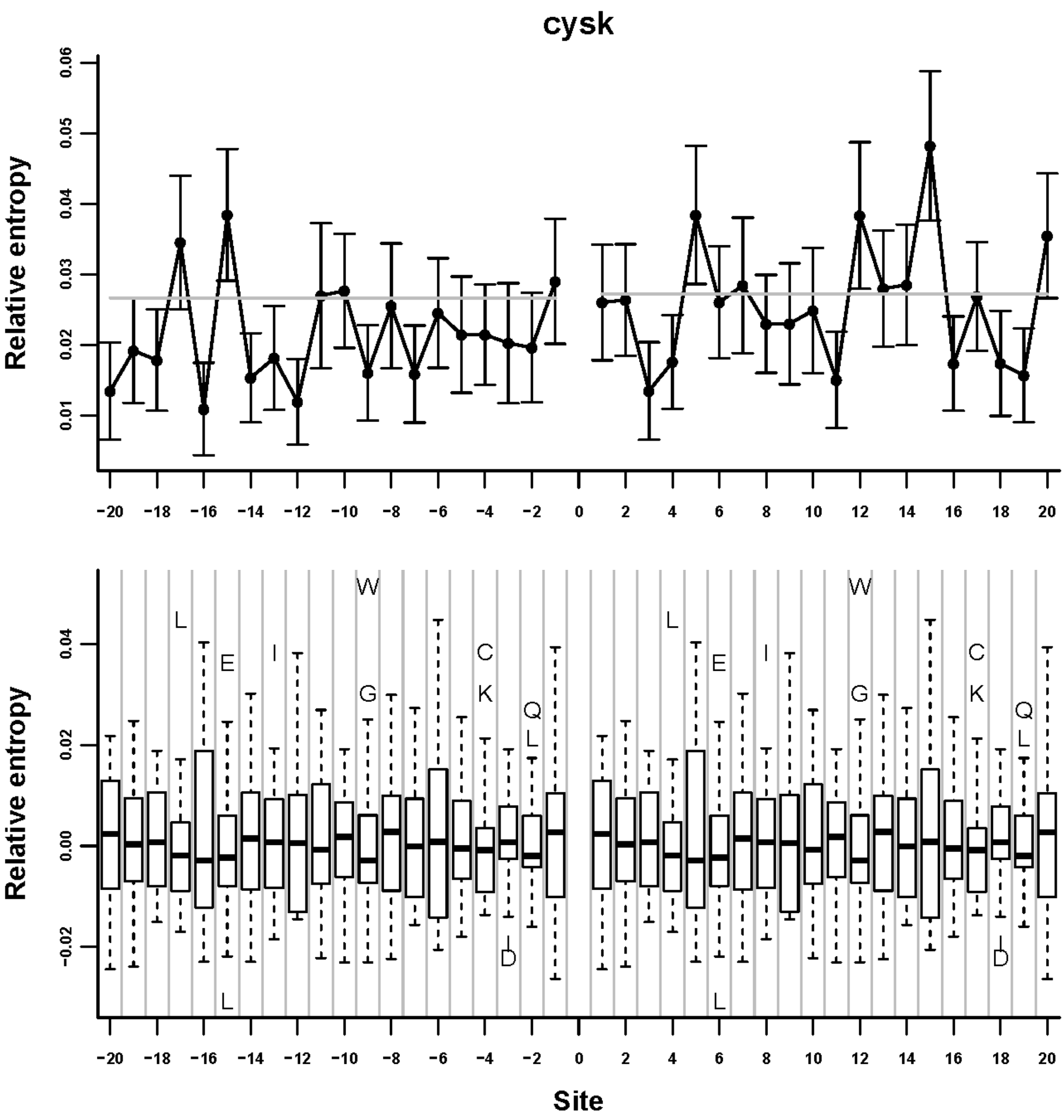

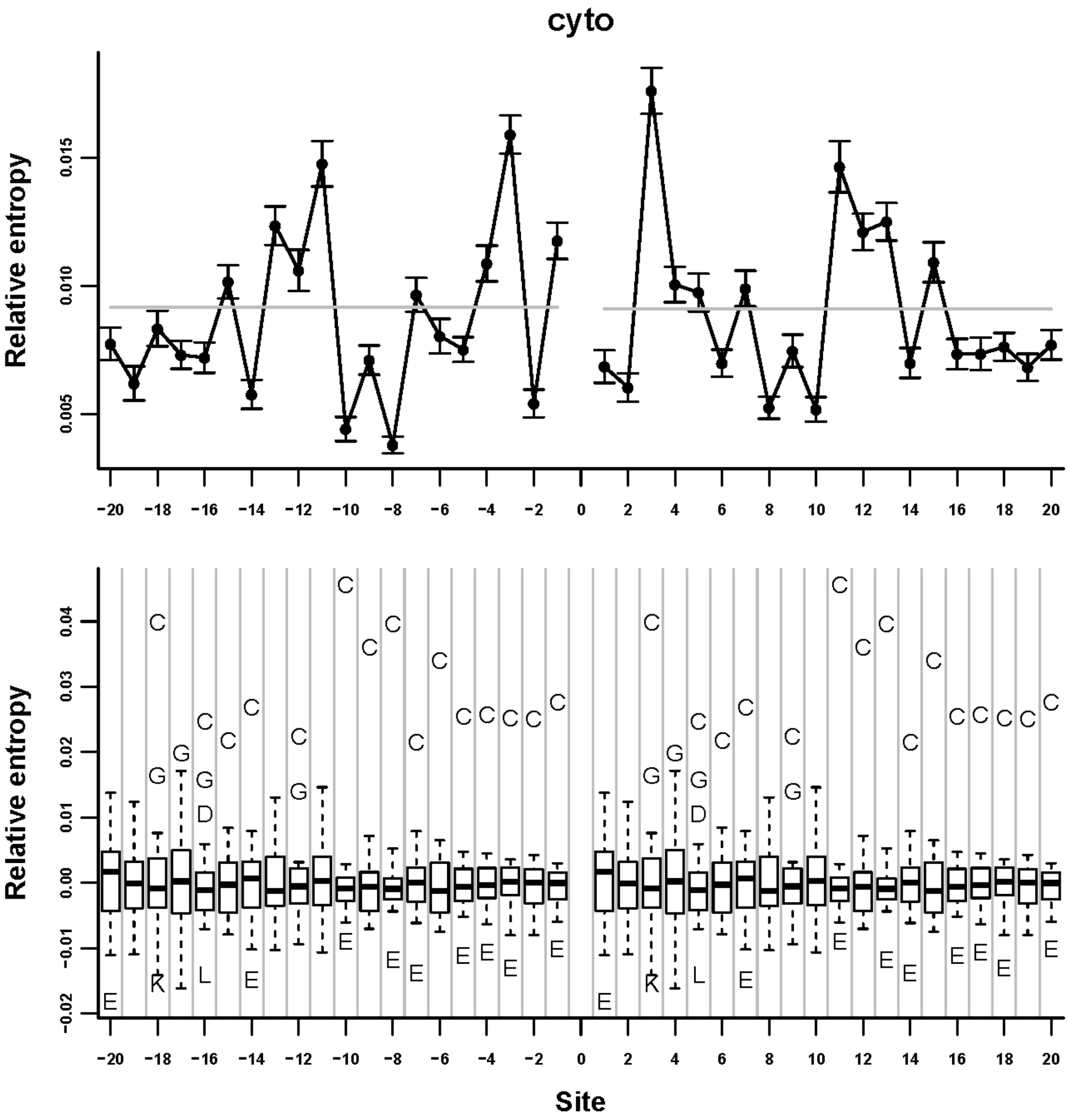
cyto
Relative entropy
Relative entropy
Site

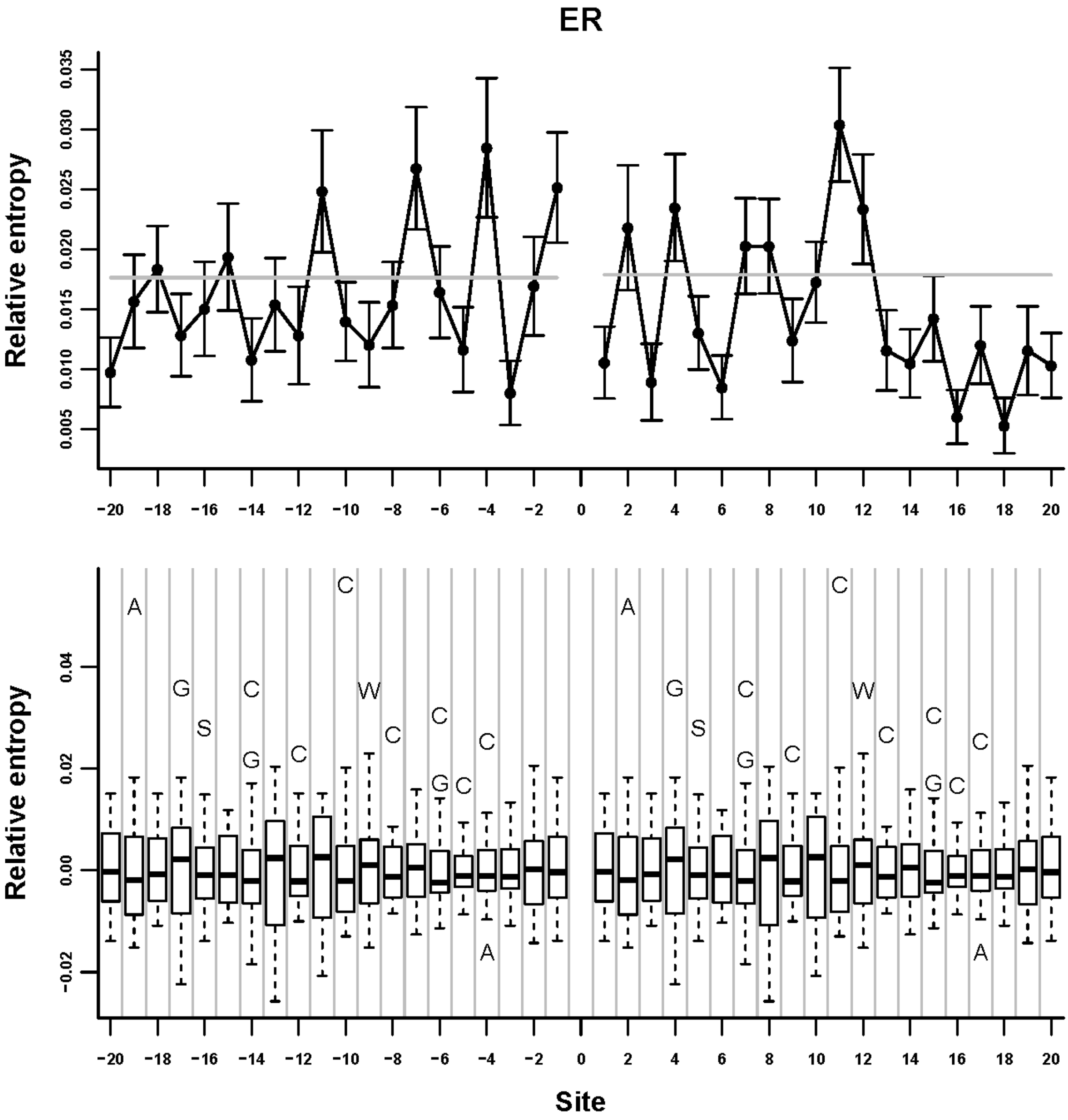
ER
Relative entropy
0.005
0.010
0.015
0.020
0.025
0.030
0.035
-20
-18
-16
-14
-12
-10
-8
-6
-4
-2
0
2
4
6
8
10
12
14
16
18
20
Relative entropy
-0.02
0.00
0.02
0.04
A
C
G
S
C
G
C
W
C
C
G
C
C
A
A
G
S
C
G
C
C
W
C
C
G
C
C
A
Site

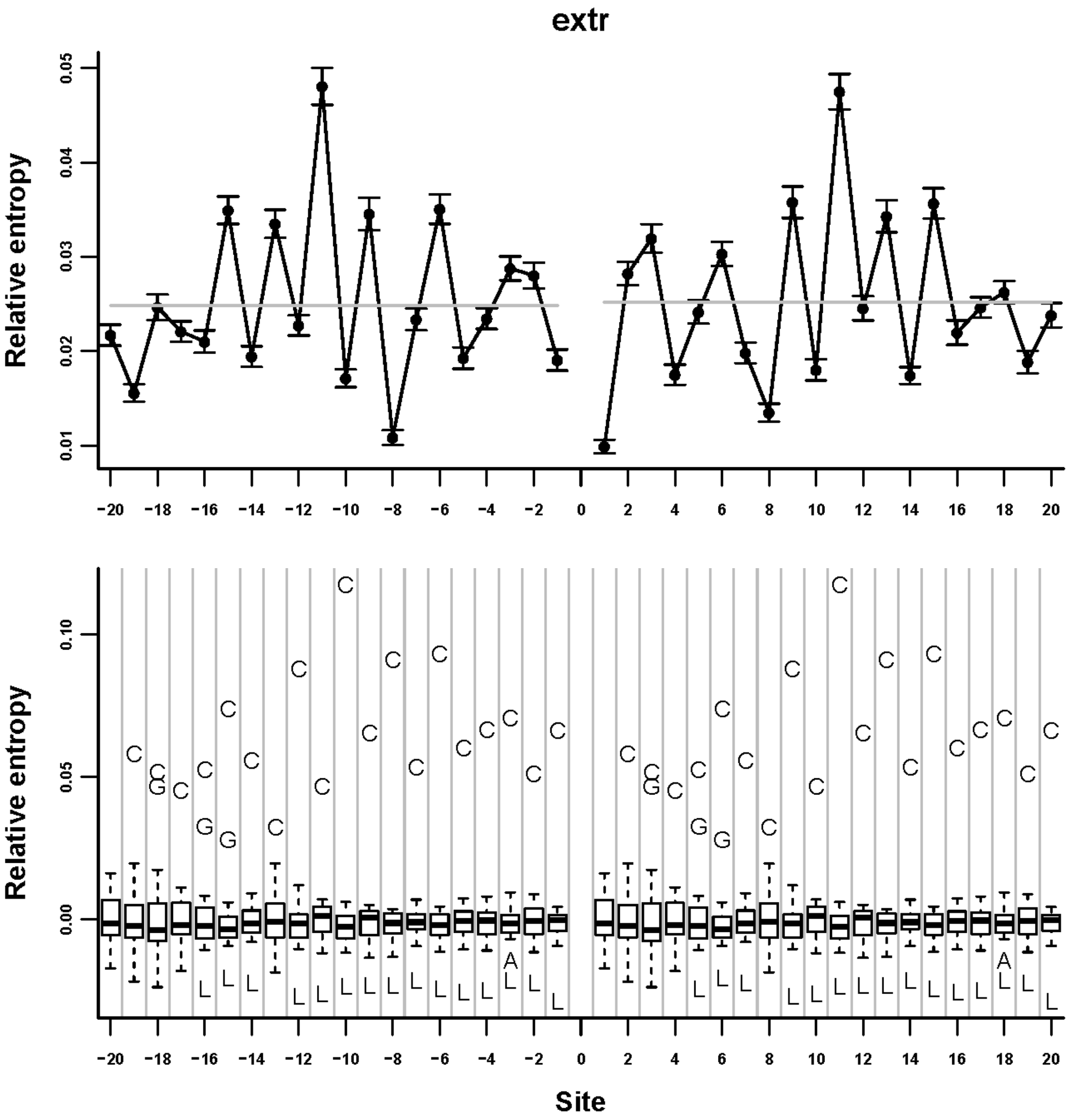
extr
Relative entropy
0.01
0.02
0.03
0.04
0.05
-20 -18 -16 -14 -12 -10 -8 -6 -4 -2 0 2 4 6 8 10 12 14 16 18 20
Relative entropy
0.00
0.05
0.10
-20 -18 -16 -14 -12 -10 -8 -6 -4 -2 0 2 4 6 8 10 12 14 16 18 20
Site

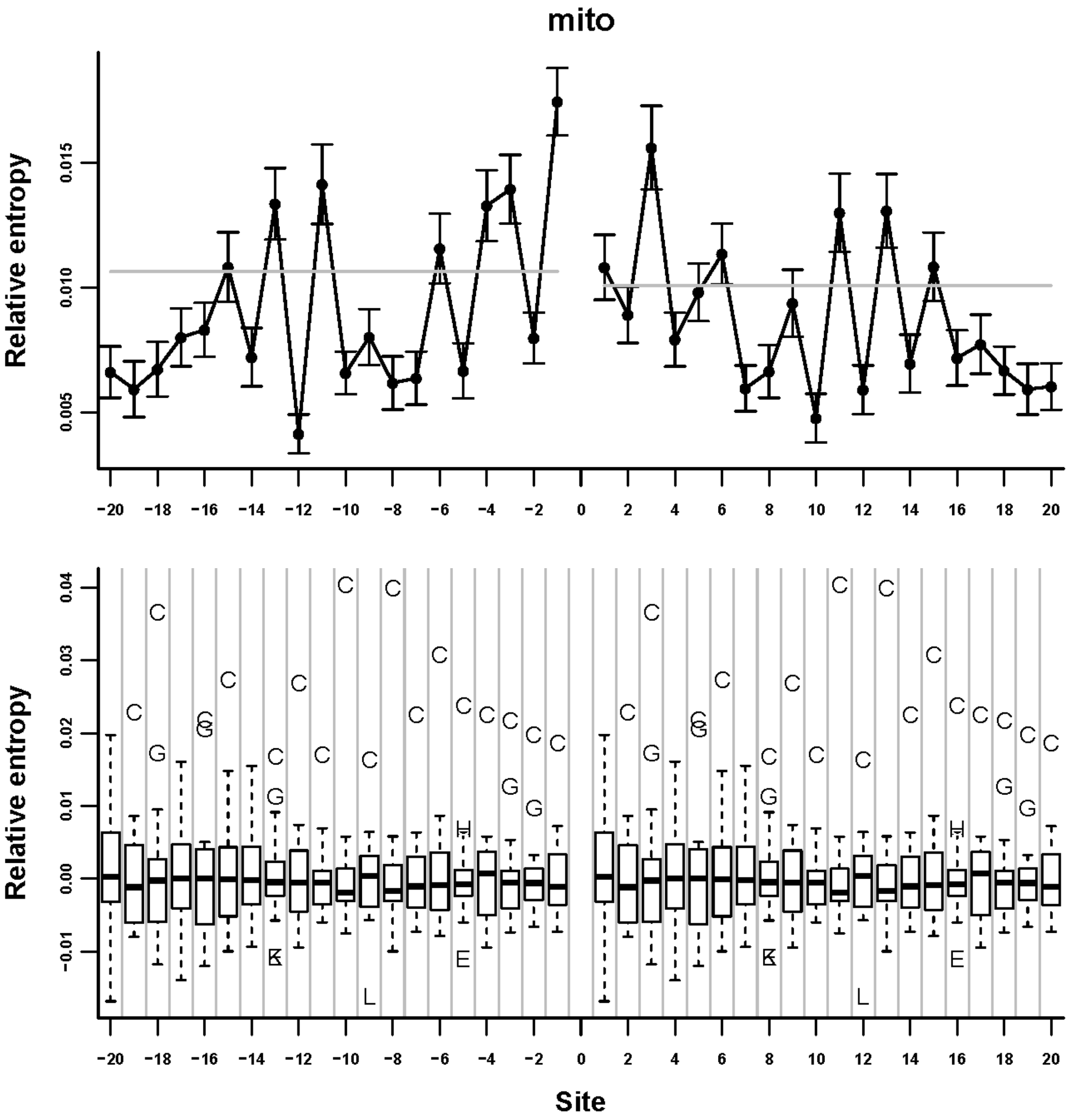
mito
Relative entropy
0.005
0.010
0.015
-20
-18
-16
-14
-12
-10
-8
-6
-4
-2
0
2
4
6
8
10
12
14
16
18
20
Relative entropy
-0.01
0.00
0.01
0.02
0.03
0.04
Site

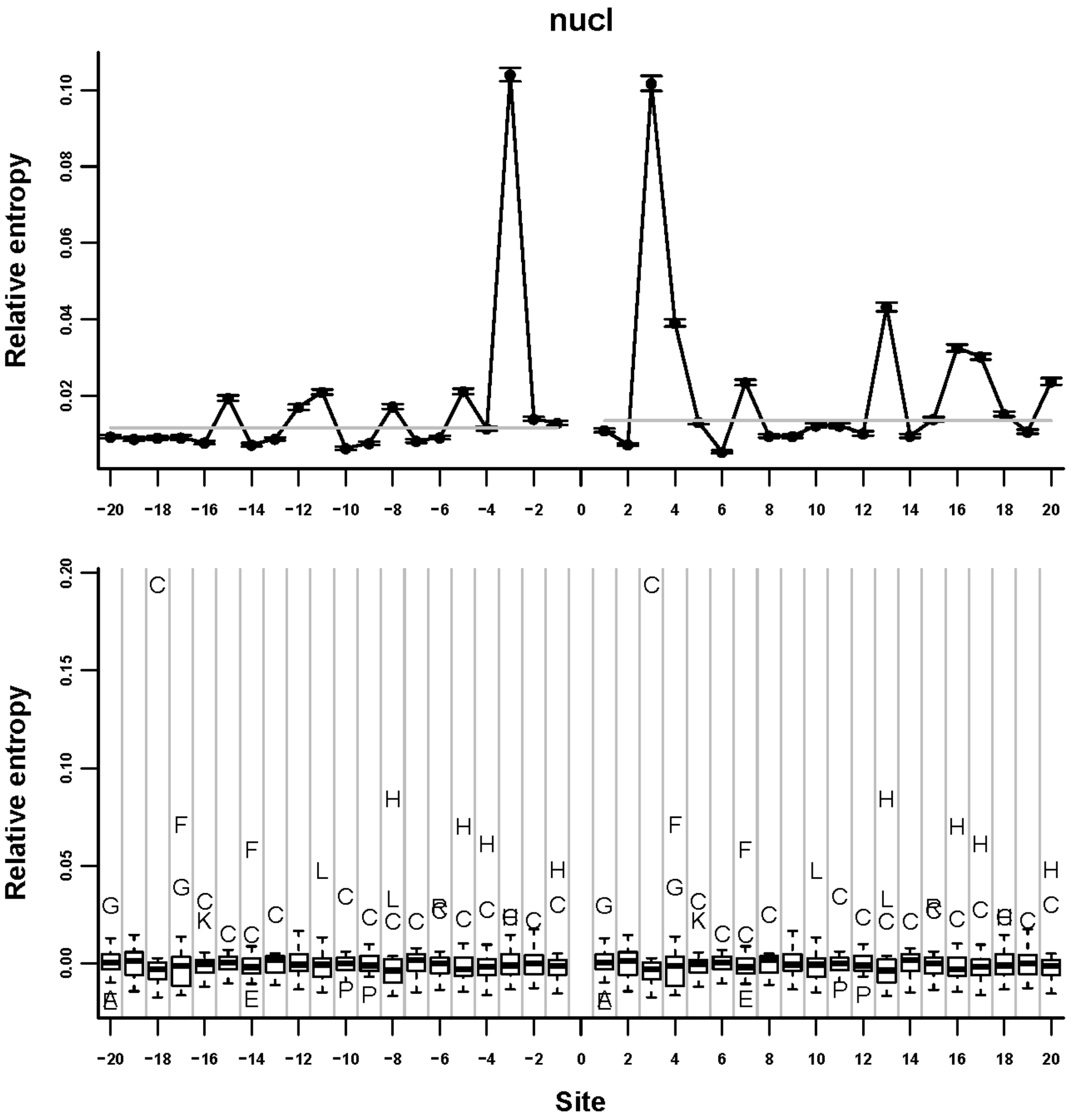

nucl
Relative entropy
Relative entropy
Site

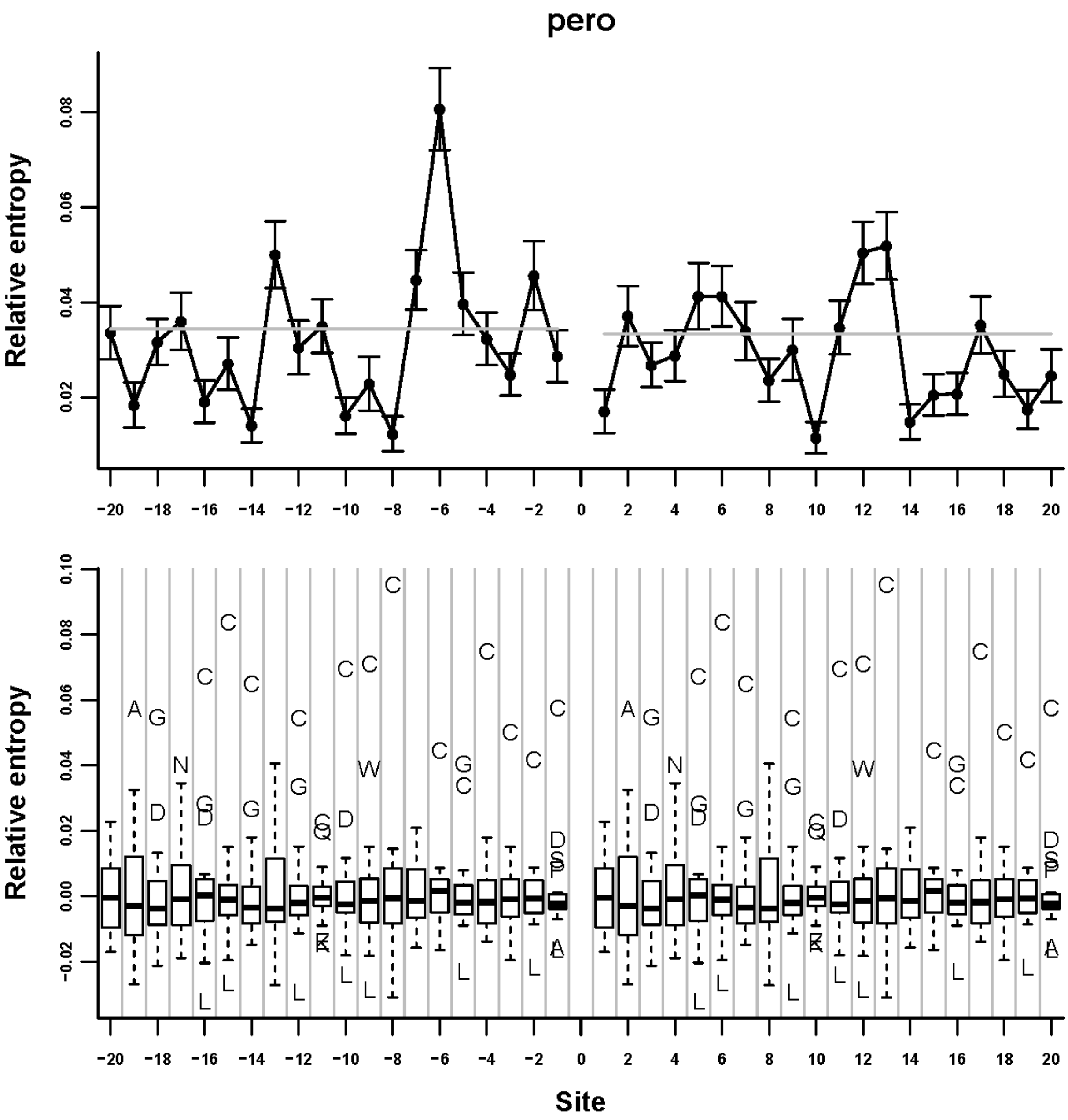
pero
Relative entropy
Relative entropy
Site

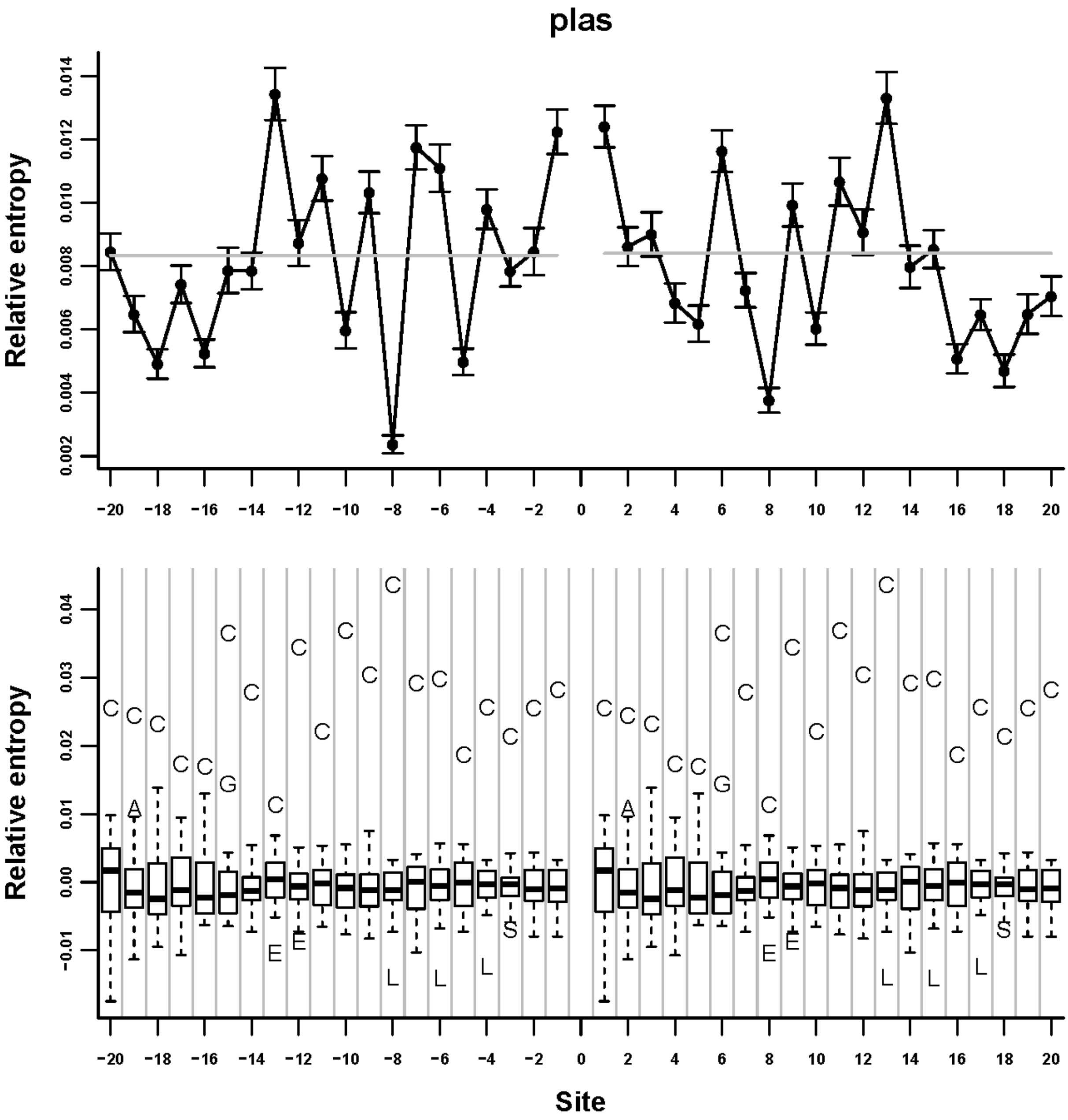
plas
Relative entropy
Relative entropy
Site

Figure S10. Neighbor preference patterns of G in different cellular locations.

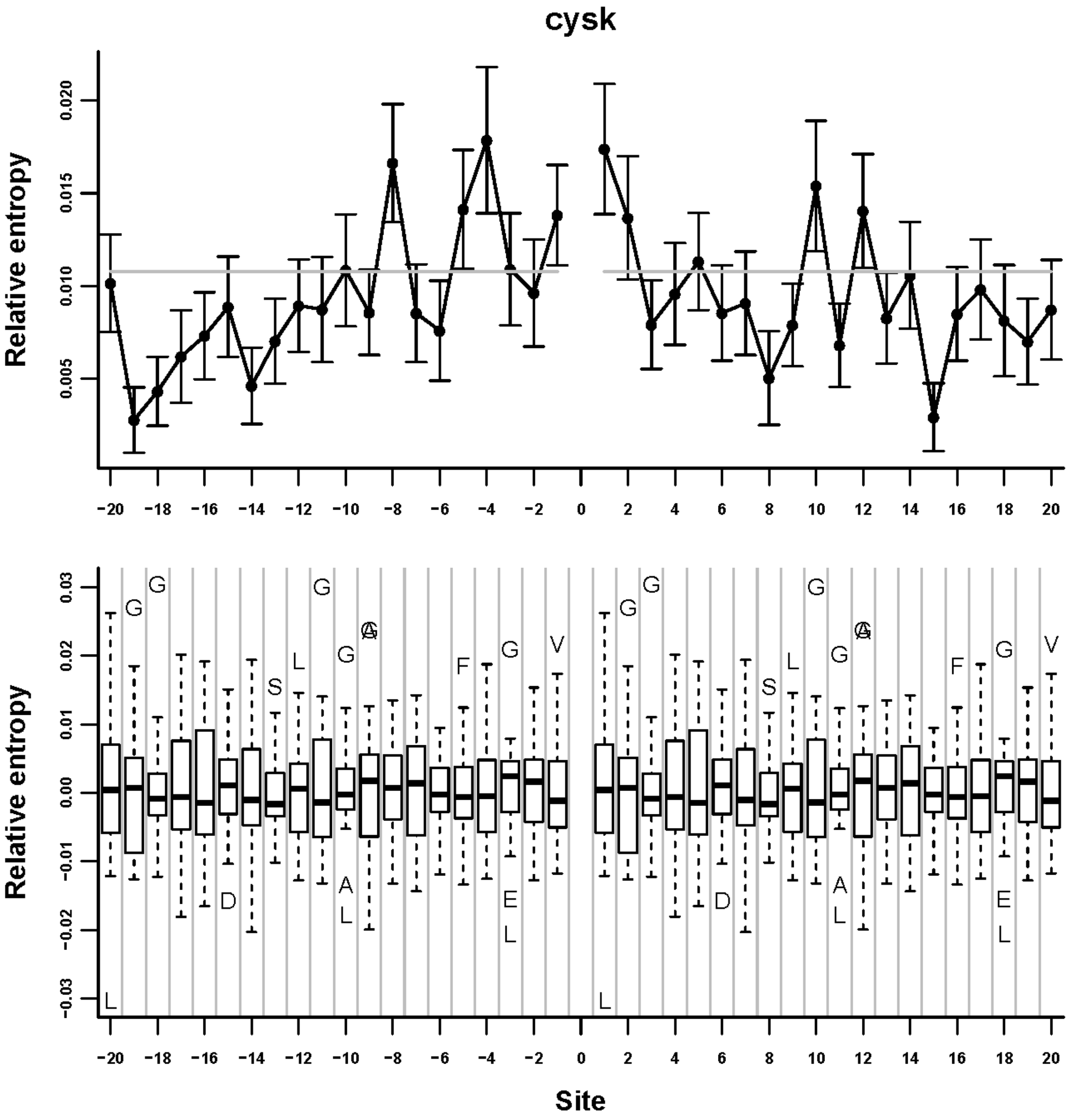

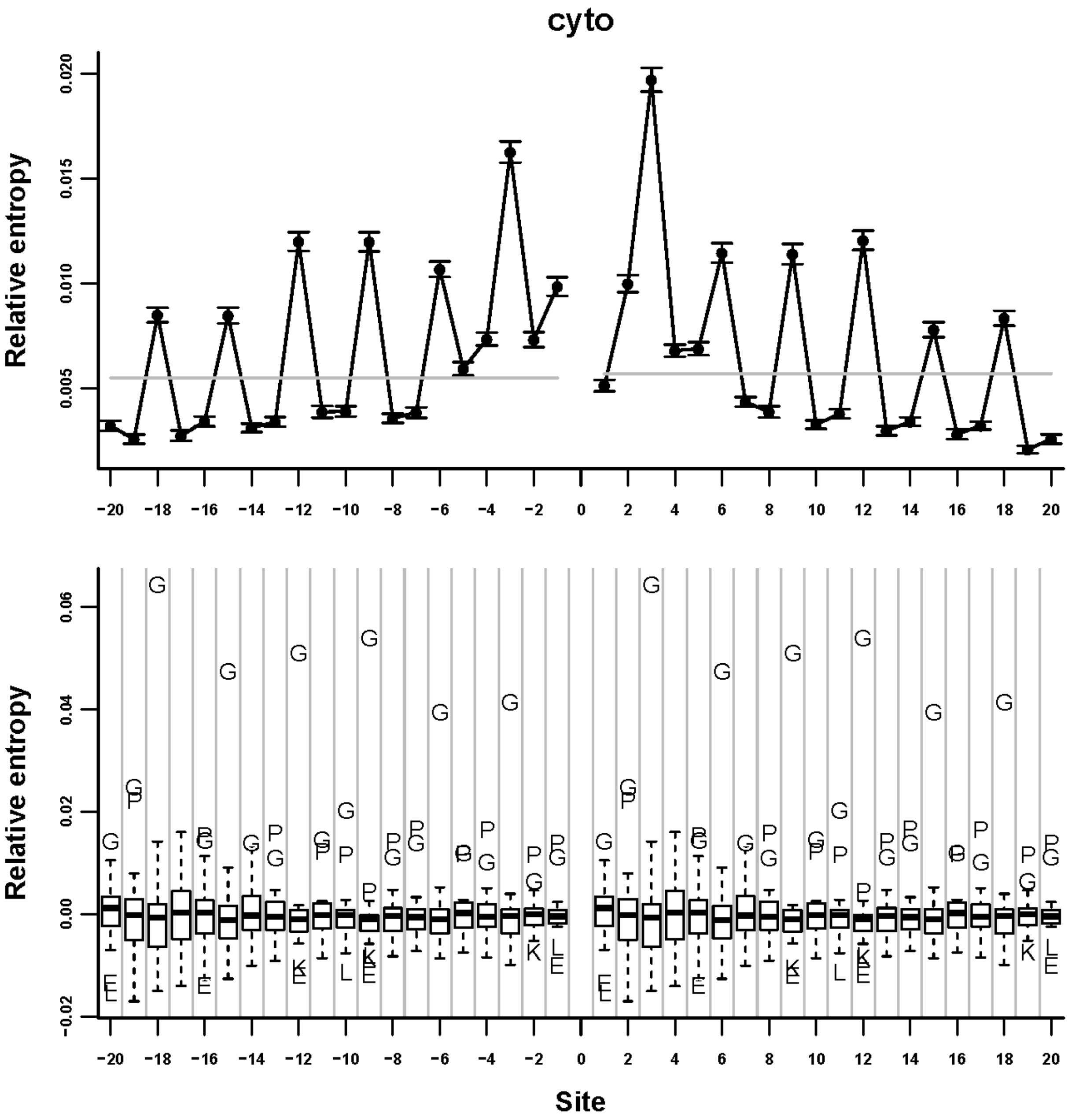
cyto
Relative entropy
0.020
0.015
0.010
0.005
−20
−18
−16
−14
−12
−10
−8
−6
−4
−2
0
2
4
6
8
10
12
14
16
18
20
Relative entropy
0.06
0.04
0.02
0.00
−0.02
Site

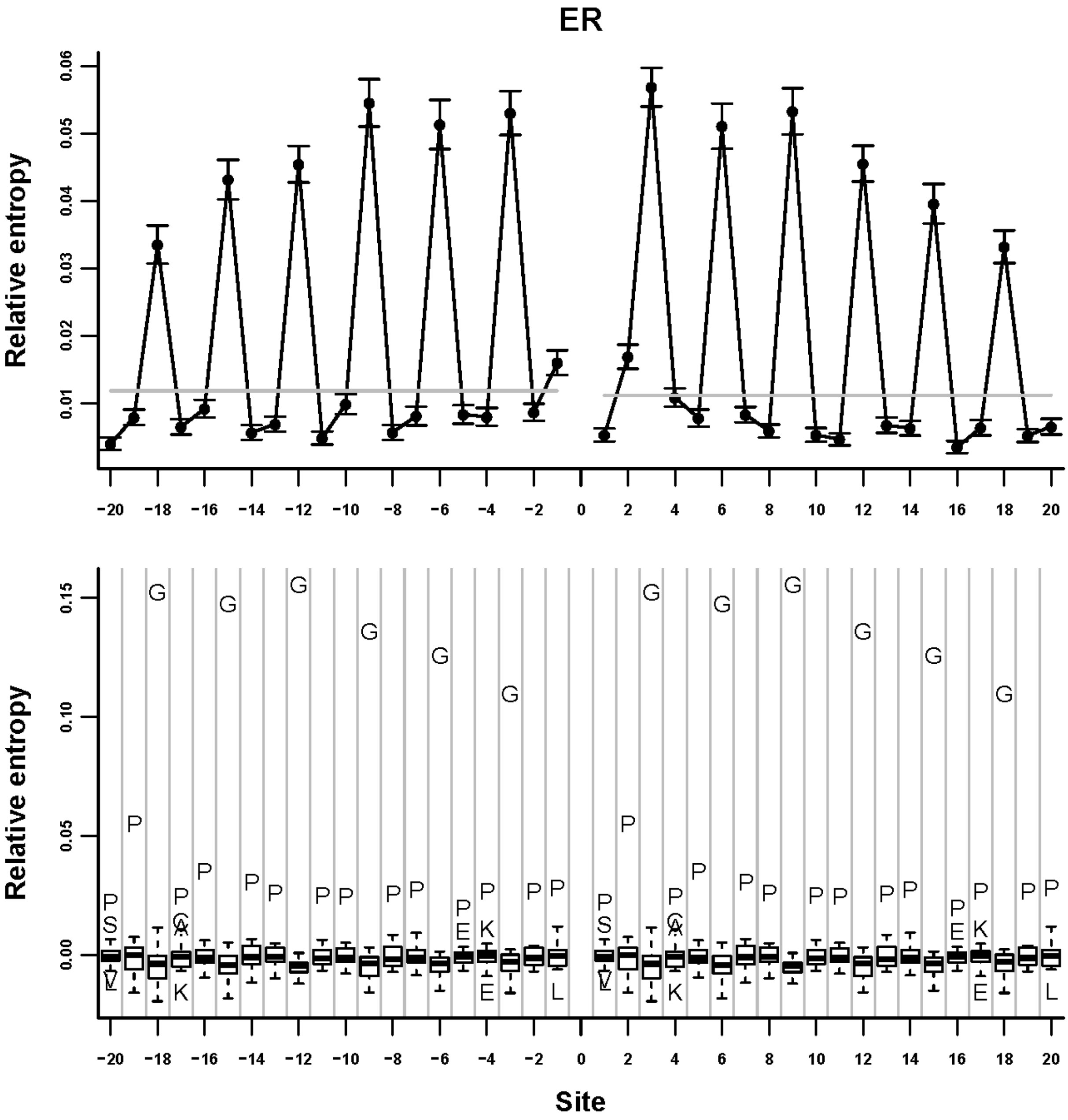
ER
Relative entropy
Relative entropy
Site

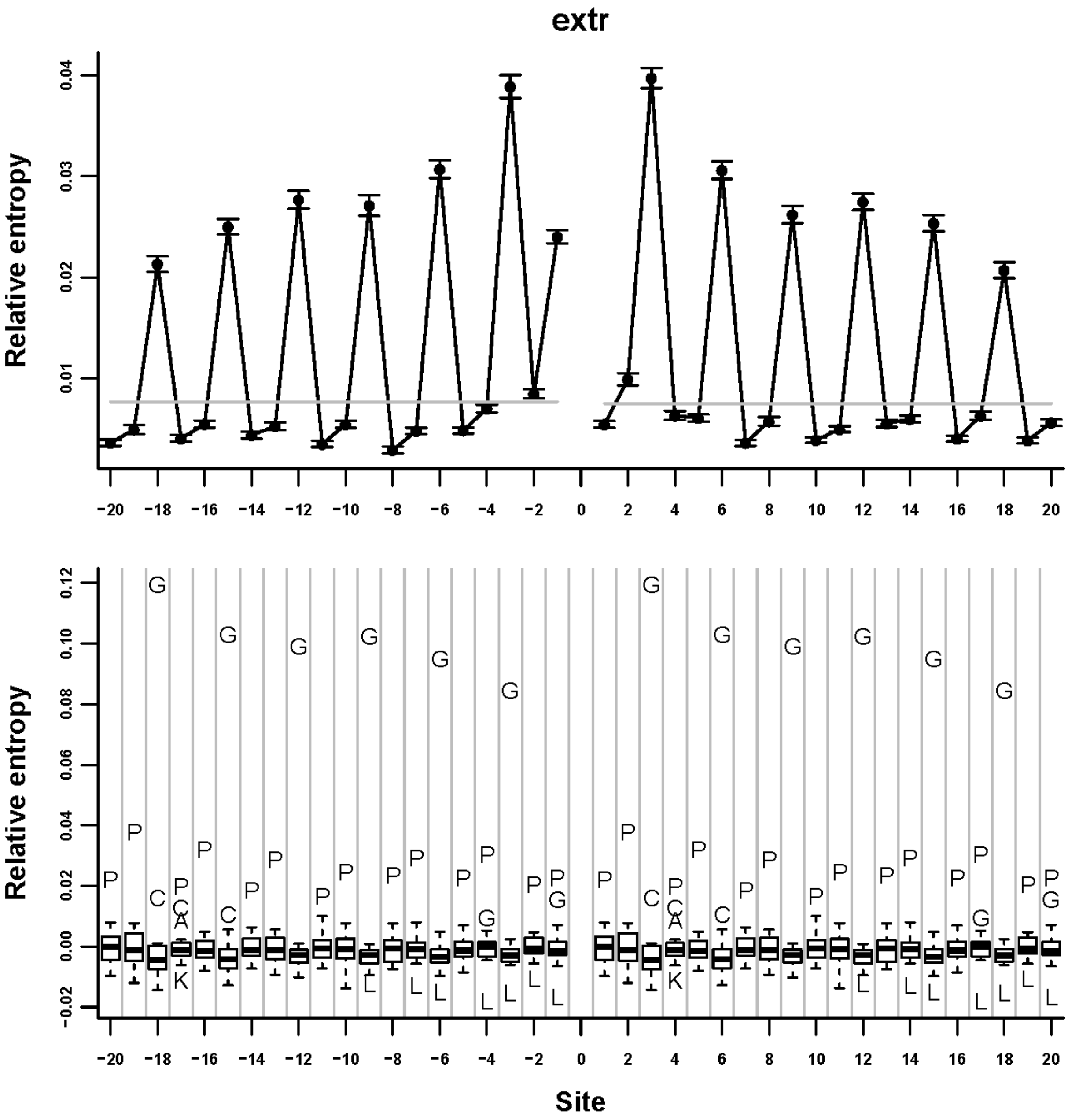
extr
Relative entropy
Site

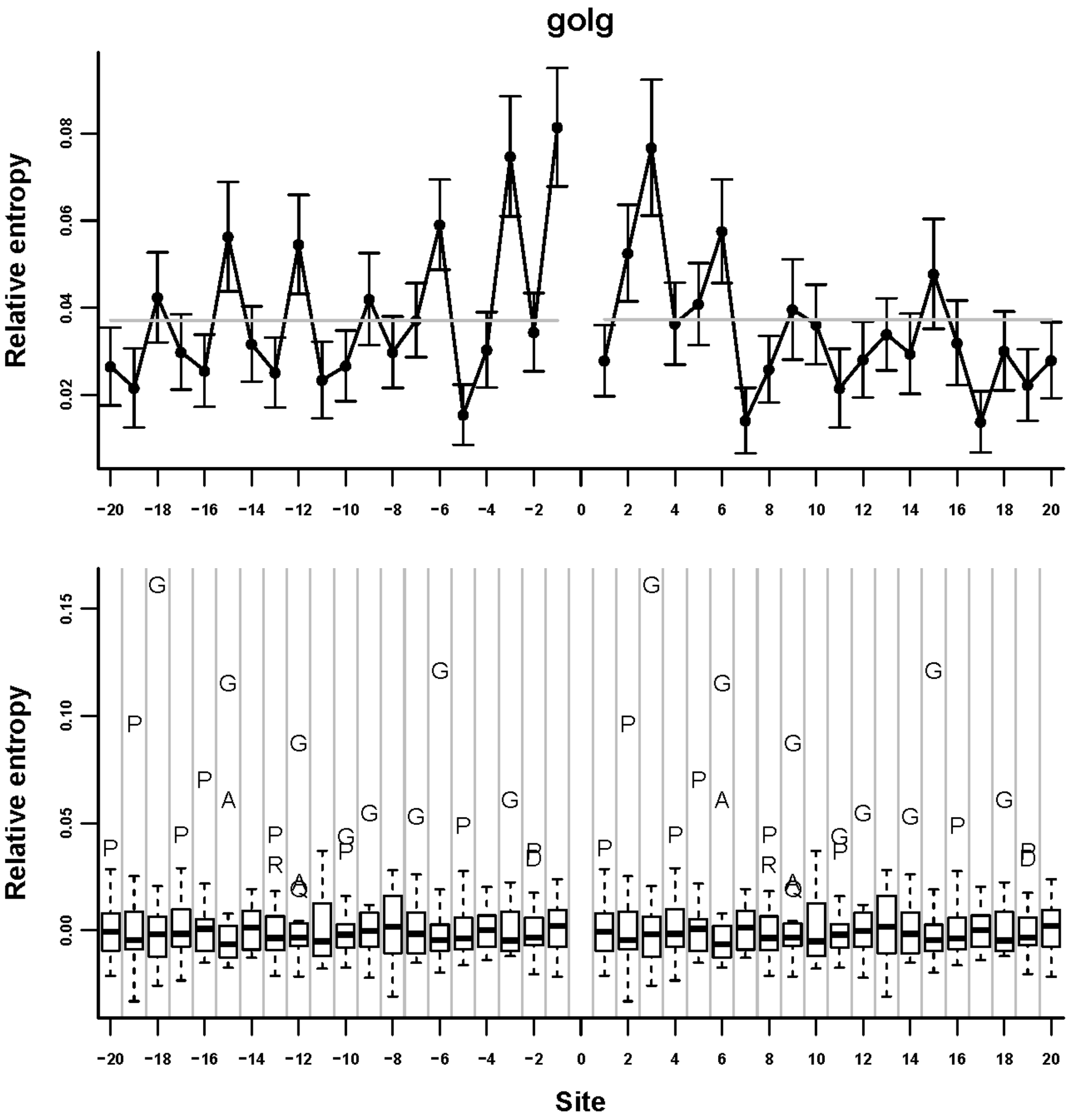
golg
Relative entropy
0.02
0.04
0.06
0.08
−20 −18 −16 −14 −12 −10 −8 −6 −4 −2 0 2 4 6 8 10 12 14 16 18 20
Relative entropy
0.00
0.05
0.10
0.15
−20 −18 −16 −14 −12 −10 −8 −6 −4 −2 0 2 4 6 8 10 12 14 16 18 20
Site

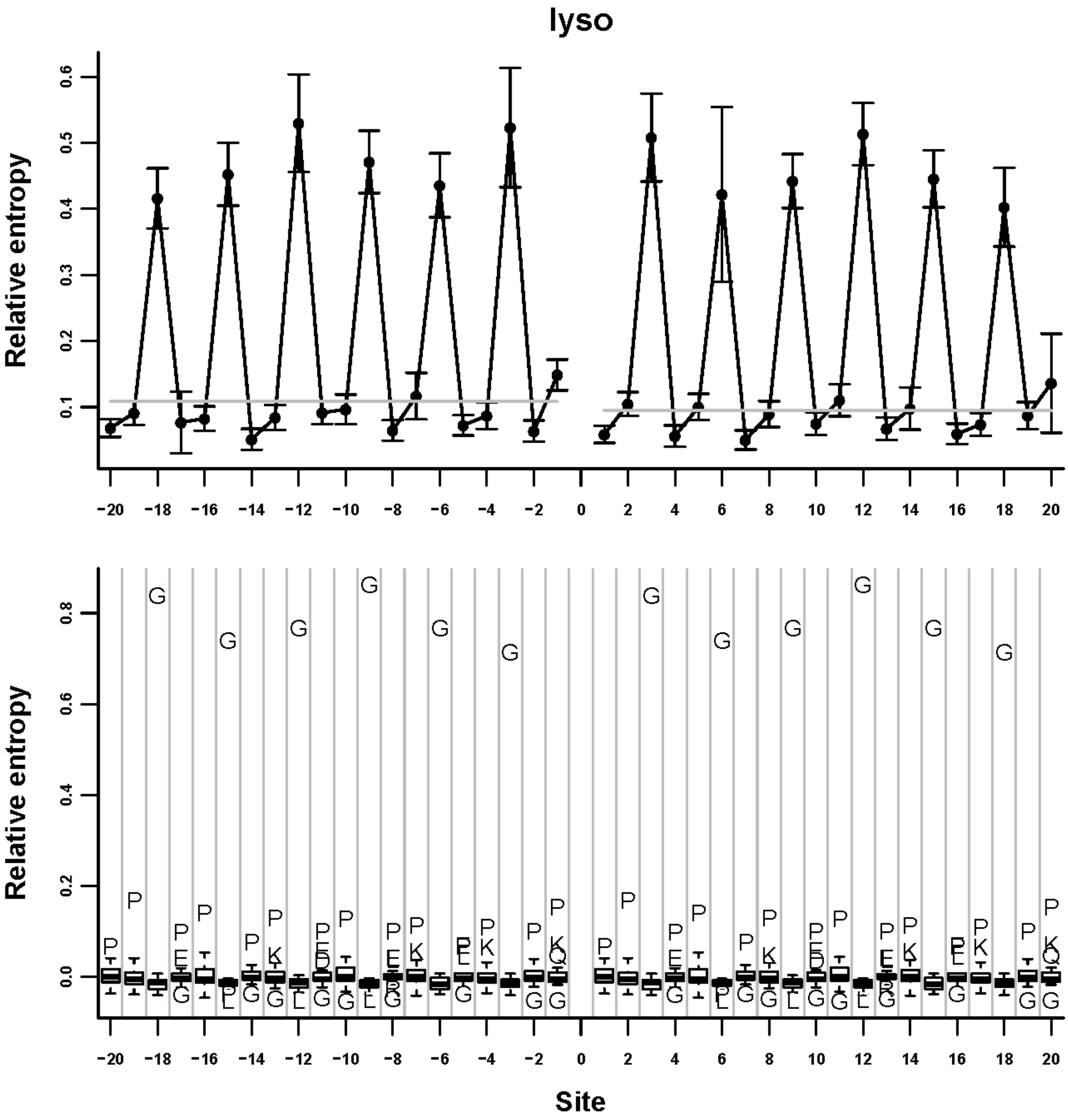
lyso
Relative entropy
Relative entropy
Site

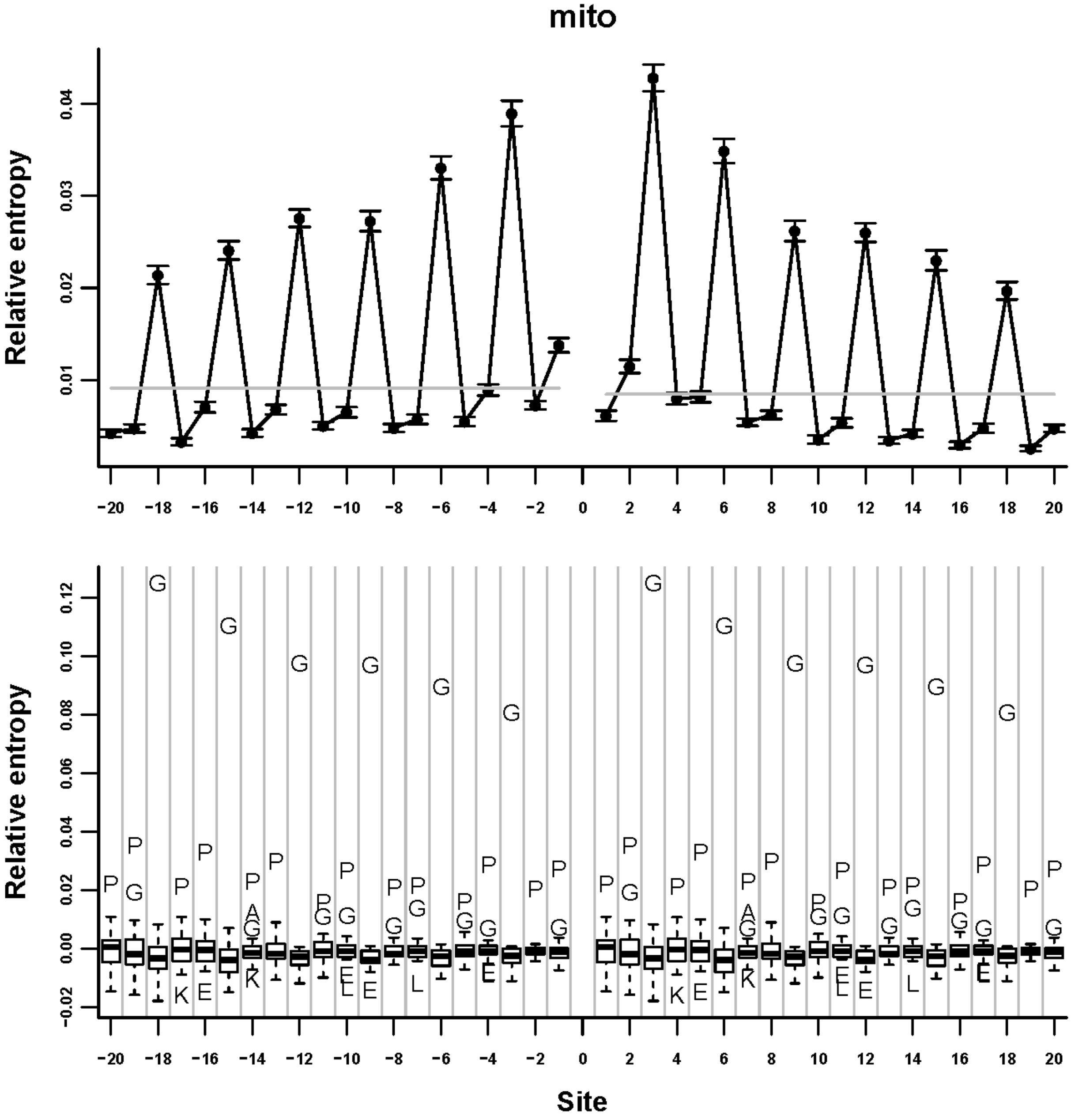
mito
Relative entropy
Site

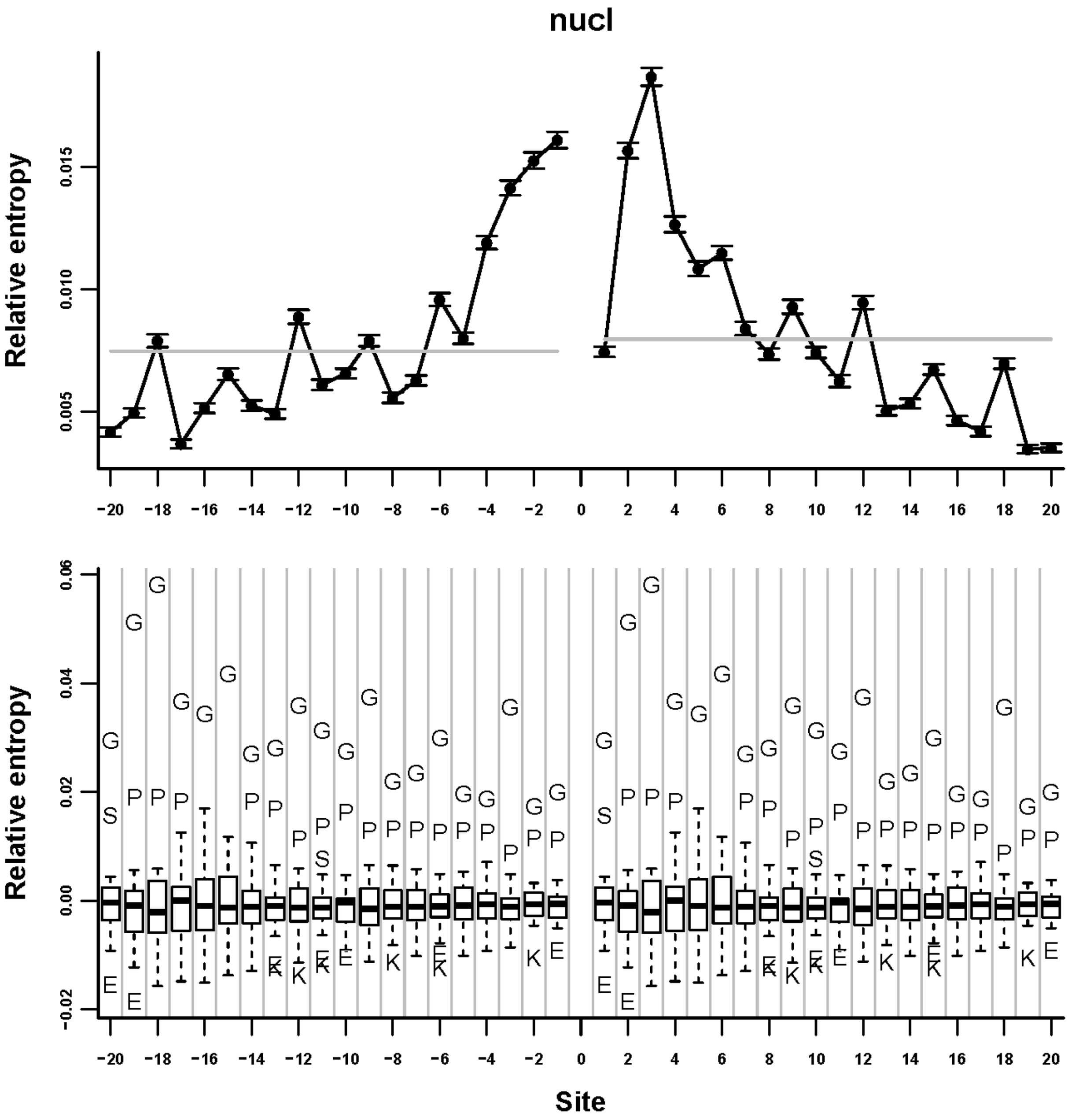
nucl
Relative entropy
Relative entropy
Site

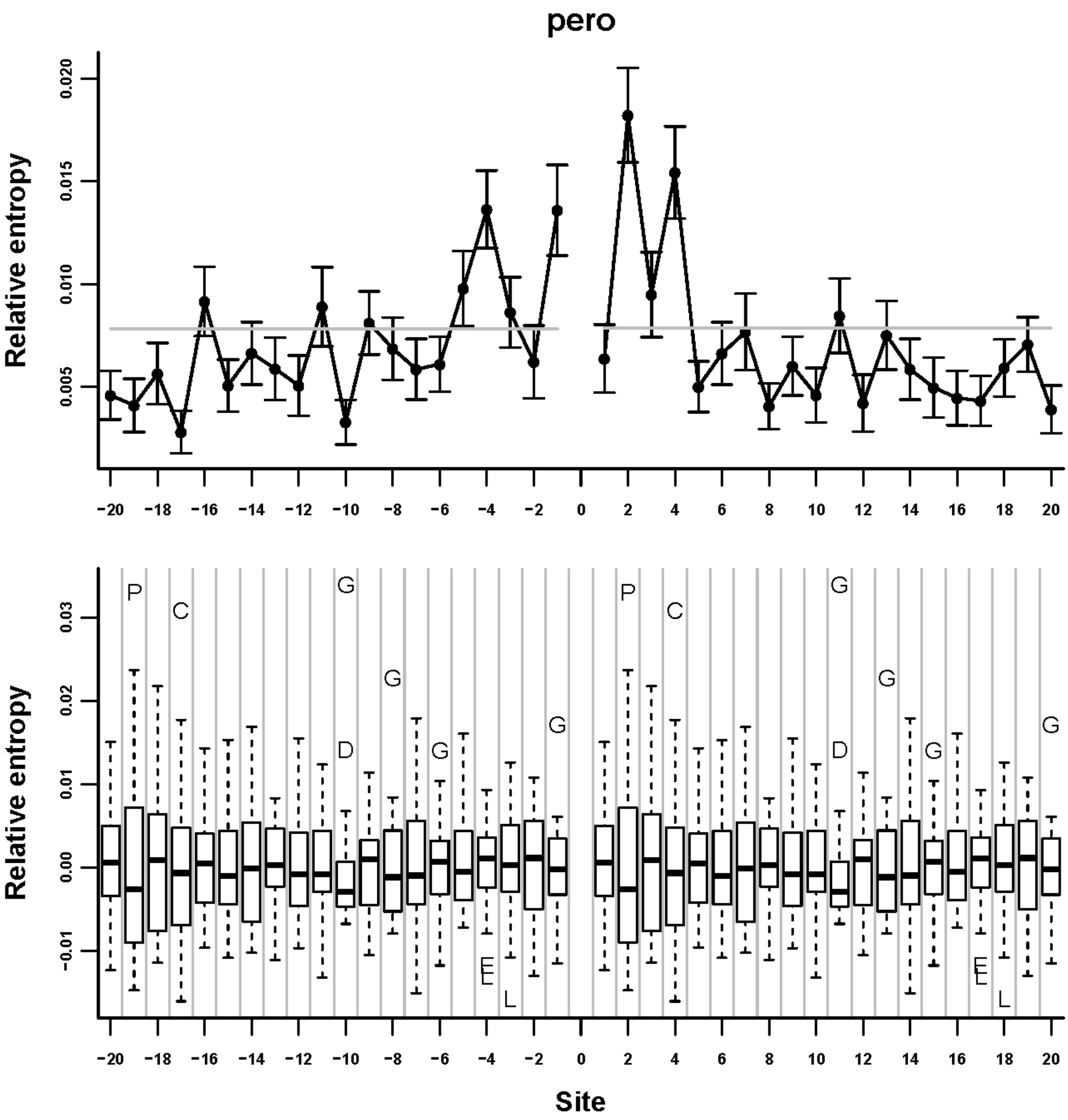

pero
Relative entropy
0.005
0.010
0.015
0.020
-20 -18 -16 -14 -12 -10 -8 -6 -4 -2 0 2 4 6 8 10 12 14 16 18 20
Relative entropy
-0.01
0.00
0.01
0.02
0.03
P
C
G
G
D
G
G
E
E
L
P
C
G
G
D
G
E
E
L
G
-20 -18 -16 -14 -12 -10 -8 -6 -4 -2 0 2 4 6 8 10 12 14 16 18 20
Site

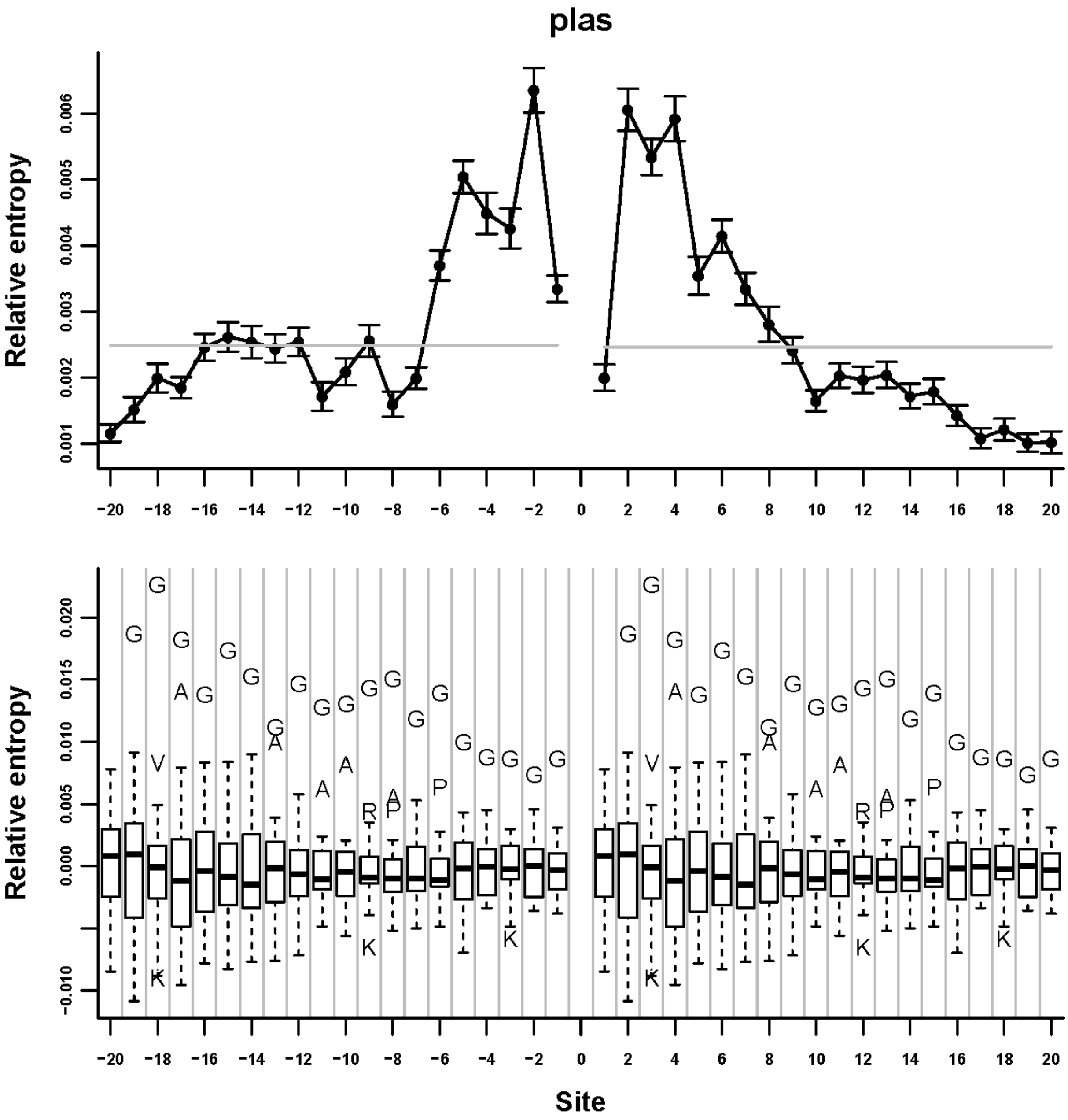
plas
Relative entropy
Relative entropy
Site
-20 -18 -16 -14 -12 -10 -8 -6 -4 -2 0 2 4 6 8 10 12 14 16 18 20

Figure S11. Comparison of the neighbor preference patterns of C and G in predicted and GO datasets.

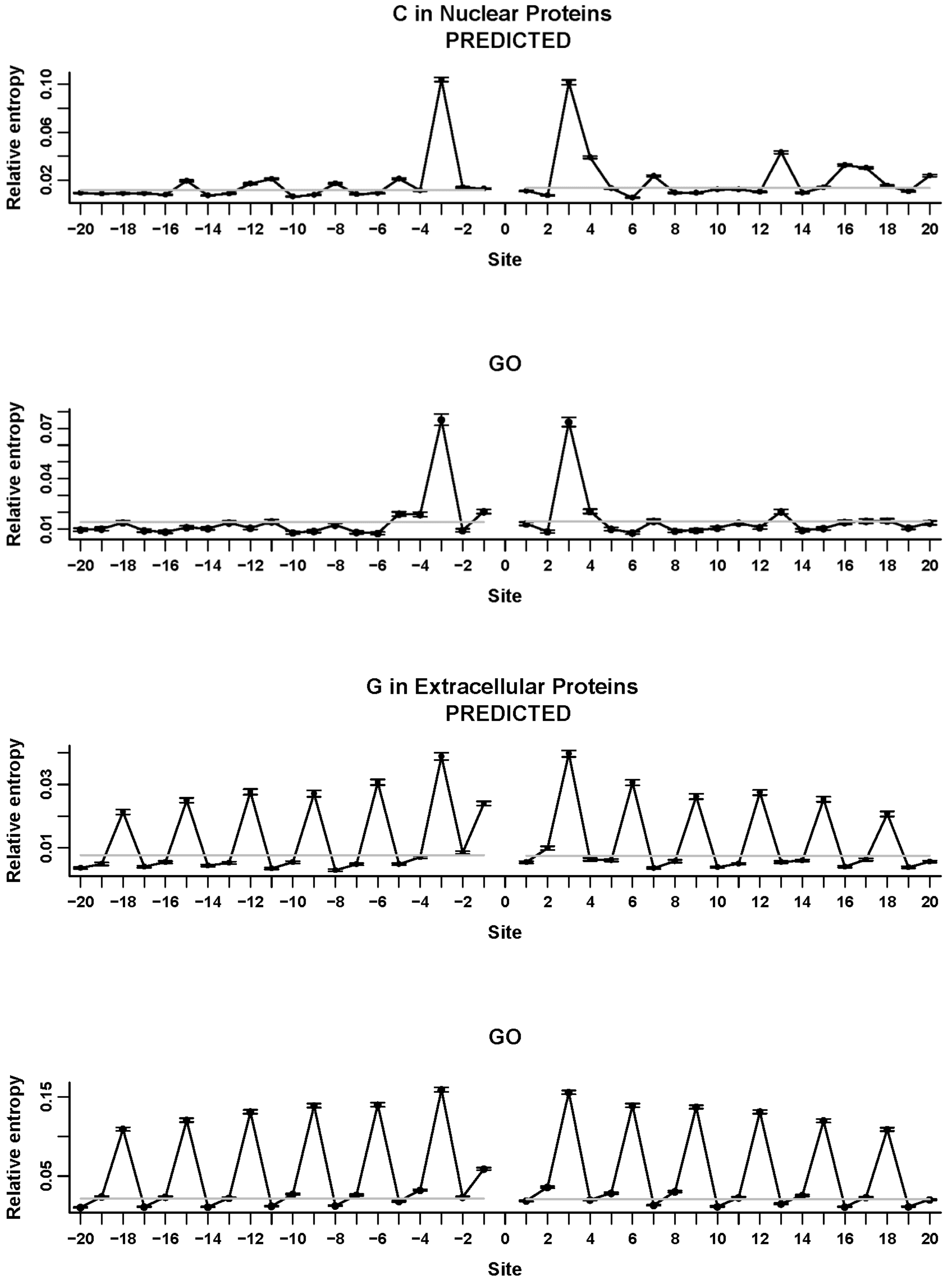

Figure S12. The phylogenetic tree of human, mouse and dog.

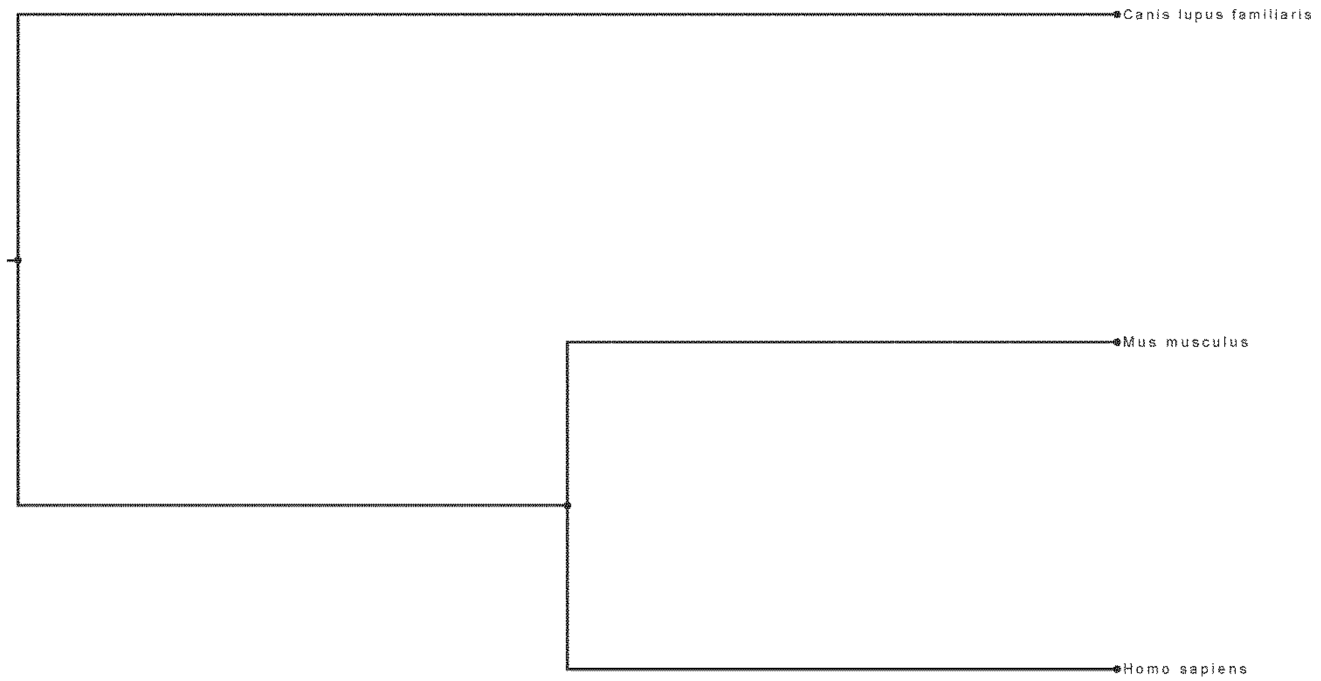

Supplement: Supplementary File 1 [file ijms-15-15963-s001.pdf]
